# Genomic characterization of the *Yersinia *genus

**DOI:** 10.1186/gb-2010-11-1-r1

**Published:** 2010-01-04

**Authors:** Peter E Chen, Christopher Cook, Andrew C Stewart, Niranjan Nagarajan, Dan D Sommer, Mihai Pop, Brendan Thomason, Maureen P Kiley Thomason, Shannon Lentz, Nichole Nolan, Shanmuga Sozhamannan, Alexander Sulakvelidze, Alfred Mateczun, Lei Du, Michael E Zwick, Timothy D Read

**Affiliations:** 1 Biological Defense Research Directorate, Naval Medical Research Center, 503 Robert Grant Avenue, Silver Spring, Maryland 20910, USA; 2 University of Maryland Institute for Advanced Computer Sciences, Center for Bioinformatics and Computational Biology, University of Maryland, College Park, Maryland 20742, USA; 3 Emerging Pathogens Institute and Department of Molecular Genetics and Microbiology, University of Florida College of Medicine, Gainesville, Florida 32610, USA; 4 454 Life Sciences Inc., 15 Commercial Street, Branford, Connecticut 06405, USA; 5 Department of Human Genetics, Emory University School of Medicine, 615 Michael Street, Atlanta, Georgia 30322, USA; 6 Division of Infectious Diseases, Emory University School of Medicine, 615 Michael Street, Atlanta, Georgia 30322, USA; 7 Current address: Computational and Mathematical Biology, Genome Institute of Singapore, Singapore-127726

## Abstract

**Background:**

New DNA sequencing technologies have enabled detailed comparative genomic analyses of entire genera of bacterial pathogens. Prior to this study, three species of the enterobacterial genus *Yersinia *that cause invasive human diseases (*Yersinia pestis*, *Yersinia pseudotuberculosis*, and *Yersinia enterocolitica*) had been sequenced. However, there were no genomic data on the *Yersinia *species with more limited virulence potential, frequently found in soil and water environments.

**Results:**

We used high-throughput sequencing-by-synthesis instruments to obtain 25- to 42-fold average redundancy, whole-genome shotgun data from the type strains of eight species: *Y. aldovae*, *Y. bercovieri*, *Y. frederiksenii*, *Y. kristensenii*, *Y. intermedia*, *Y. mollaretii, Y. rohdei*, and *Y. ruckeri*. The deepest branching species in the genus, *Y. ruckeri*, causative agent of red mouth disease in fish, has the smallest genome (3.7 Mb), although it shares the same core set of approximately 2,500 genes as the other members of the species, whose genomes range in size from 4.3 to 4.8 Mb. *Yersinia *genomes had a similar global partition of protein functions, as measured by the distribution of Cluster of Orthologous Groups families. Genome to genome variation in islands with genes encoding functions such as ureases, hydrogeneases and B-12 cofactor metabolite reactions may reflect adaptations to colonizing specific host habitats.

**Conclusions:**

Rapid high-quality draft sequencing was used successfully to compare pathogenic and non-pathogenic members of the *Yersinia *genus. This work underscores the importance of the acquisition of horizontally transferred genes in the evolution of *Y. pestis *and points to virulence determinants that have been gained and lost on multiple occasions in the history of the genus.

## Abstract 2

Comparative *Yersinia *genomics identifies features responsible for the colonization of specific host habitats and the horizontal transfer of virulence determinants.

## Background

Of the millions of species of bacteria that live on this planet, only a very small percentage cause serious human diseases [1]. Comparative genetic studies are revealing that many pathogens have only recently emerged from protean environmental, commensal or zoonotic populations [2-5]. For a variety of reasons, most research effort has been focused on characterizing these pathogens, while their closely related non-pathogenic relatives have only been lightly studied. As a result, our understanding of the population biology of these clades remains biased, limiting our knowledge of the evolution of virulence and our ability to design reliable assays that distinguish pathogen signatures from the background in the clinic and environment [6].

The recent development of second generation sequencing platforms (reviewed by Mardis [7,8] and Shendure [7,8]) offers an opportunity to change the direction of microbial genomics, enabling the rapid genome sequencing of large numbers of strains of both pathogenic and non-pathogenic strains. Here we describe the deployment of new sequencing technology to extensively sample eight genomes from the *Yersinia *genus of the family Enterobacteriaceae. The first published sequencing studies on the *Yersinia *genus have focused exclusively on invasive human disease-causing species that included five *Yersinia pestis *genome sequences (one of which, strain 91001, is from the avirulent 'microtus' biovar) [9-12], two *Yersinia pseudotuberculosis *[13,14] and one *Yersinia enterocolitica *biotype 1B [15]. Primarily a zoonotic pathogen, *Y. pestis*, the causative agent of bubonic plague and a category A select agent, is a recently emerged lineage that has since undergone global expansion [2]. Following introduction into a human through flea bite [16], *Y. pestis *is engulfed by macrophages and taken to the regional lymph nodes. *Y. pestis *then escapes the macrophages and multiplies to cause a highly lethal bacteremia if untreated with antibiotics. *Y. pseudotuberculosis *and *Y. enterocolitica *(primarily biotype 1B) are enteropathogens that cause gastroenteritis following ingestion and translocation of the Peyer's patches. Like *Y. pestis*, the enteropathogenic Yersiniae can escape macrophages and multiply outside host cells, but unlike their more virulent cogener, they only usually cause self-limiting inflammatory diseases.

The generally accepted pathway for the evolution of these more severe disease-causing Yersiniae is memorably encapsulated by the recipe, 'add DNA, stir, reduce' [17]. In each species DNA has been 'added' by horizontal gene transfer in the form of plasmids and genomic islands. All three human pathogens carry a 70-kb pYV virulence plasmid (also known as pCD), which carries the Ysc type III secretion system and Yops effectors [18-20], that is not detected in non-pathogenic species. *Y. pestis *also has two additional plasmids, pMT (also known as pFra), containing the F1 capsule-like antigen and murine toxin, and pPla (also known as pPCP1), which carries plasminogen-activating factor, Pla. *Y. pestis*, *Y. pseudotuberculosis*, and biotype 1B *Y. enterocolitica *also contain a chromosomally located, mobile, high-pathogenicity island (HPI) [21]. The HPI includes a cluster of genes for biosynthesis of yersiniabactin, an iron-binding siderophore necessary for systemic infection [22]. 'Stir' refers to intra-genomic change, notably the recent expansion of insertion sequences (IS) within *Y. pestis *(3.7% of the *Y. pestis *CO92 genome [9]) and a high level of genome structural variation [23]. 'Reduce' describes the loss of functions via deletions and pseudogene accumulation in *Y. pestis *[9,13] due to shifts in selection pressure caused by the transition from *Y. pseudotuberculosis*-like enteropathogenicity to a flea-borne transmission cycle. This description of *Y. pestis *evolution is, of course, oversimplified. *Y. pestis *strains show considerable diversity at the phenotypic level and there is evidence of acquisition of plasmids and other horizontally transferred genes [[12,24,25] DNA microarray, [26,27]].

While most attention is focused on the three well-known human pathogens, several other, less familiar *Yersinia *species have been split off from *Y. enterocolitica *over the past 40 years based on biochemistry, serology and 16S RNA sequence [28,29]. *Y. ruckeri *is an agriculturally important fish pathogen that is a cause of 'red mouth' disease in salmonid fish. The species has sufficient phylogenetic divergence from the rest of the *Yersinia *genus to stir controversy about its taxonomic assignment [30]. *Y. fredricksenii*, *Y. kristensenii*, *Y. intermedia*, *Y. mollaretii*, *Y. bercovieri*, and *Y. rohdei *have been isolated from human feces, fresh water, animal feces and intestines and foods [28]. There have been reports associating some of the species with human diarrheal infections [31] and lethality for mice [32]. *Y. aldovae *is most often isolated from fresh water but has also been cultured from fish and the alimentary tracts of wild rodents [33]. There is no report of isolation of *Y. aldovae *from human feces or urine [28].

Using microbead-based, massively parallel sequencing by synthesis [34] we rapidly and economically obtained high redundancy genome sequence of the type strains of each of these eight lesser known *Yersinia *species. From these genome sequences, we were able to determine the core gene set that defines the *Yersinia *genus and to look for clues to distinguish the genomes of human pathogens from less virulent strains.

## Results

### High-redundancy draft genome sequences of eight *Yersinia *species

Whole genome shotgun coverage of eight previously unsequenced *Yersinia *species (Table 1) was obtained by single-end bead-based pyrosequencing [34] using the 454 Life Sciences GS-20 instrument. Each of the eight genomes was sequenced to a high level of redundancy (between 25 and 44 sequencing reads per base) and assembled *de novo *into large contigs (Table 2; Additional file 1). Excluding contigs that covered repeat regions and therefore had significantly increased copy number, the quality of the sequence of the draft assemblies was high, with less than 0.1% of the sequence of each genome having a consensus quality score [35] less than 40. Moreover, a more recent assessment of quality of GS-20 data suggests that the scores generated by the 454 Life Sciences software are an underestimation of the true sequence quality [36]. The most common sequencing error encountered when assembling pyrosequencing data is the rare calling of incorrect numbers of homopolymers caused by variation in the intensity of fluorescence emitted upon extension with the labeled nucleoside [34].

**Table 1:** Strains sequenced in this study

Species	ATCC number	Other designations	Year isolated	Location isolated	Description	Optimum growth temperature	Reference
*Y. aldovae*	35236T	CNY 6065	NR	Czechoslovakia	Drinking water	26°C	[100]
*Y. bercovieri*	43970T	CDC 2475-87	NR	France	Human stool	26°C	[101]
*Y. frederiksenii*	33641T	CDC 1461-81, CIP 80-29	NR	Denmark	Sewage	26°C	[102]
*Y. intermedia*	29909T	CIP 80-28	NR	NR	Human urine	37°C	[103]
*Y. kristensenii*	33638T	CIP 80-30	NR	NR	Human urine	26°C	[104]
*Y. mollaretii*	43969T	CDC 2465-87	NR	USA	Soil	26°C	[101]
*Y. rohdei*	43380T	H271-36/78, CDC 3022-85	1978	Germany	Dog feces	26°C	[105]
*Y. ruckeri*	29473T	2396-61	1961	Idaho, USA	Rainbow trout (*Oncorhynchus mykiss*) with red mouth disease	26°C	[67]

NR, not reported in reference publication.

**Table 2:** Genomes summary

Species	Type strain	NCBI project ID	GenBank accession number	Total reads	Number of contigs >500 nt	Total length of large contigs	% large contigs <Q40	Number of contigs aligned to chromosomal scaffold
*Y. rohdei*	ATCC_43380	29767	[Genbank:ACCD00000000]	991,106	83	4,303,720	0.11	60
*Y. ruckeri*	ATCC_29473	29769	[Genbank:ACCC00000000]	1,347,304	103	3,716,658	0.004	68
*Y. aldovae*	ATCC_35236	29741	[Genbank:ACCB00000000]	1,125,002	104	4,277,123	0.006	60
*Y. kristensenii*	ATCC_33638	29761	[Genbank:ACCA00000000]	1,374,452	86	4,637,246	0.003	63
*Y. intermedia*	ATCC_29909	29755	[Genbank:AALF00000000]	1,768,909	74	4,684,150	0.003	68
*Y. frederiksenii*	ATCC_33641	29743	[Genbank:AALE00000000]	1,504,985	90	4,864,031	0.005	56
*Y. mollaretii*	ATCC_43969	16105	[Genbank:AALD00000000]	1,825,876	110	4,535,932	0.003	80
*Y. bercovieri*	ATCC_43970	16104	[Genbank:AALC00000000]	1,263,275	144	4,316,521	0.006	91

Previous studies and our experience suggest that at this level of sequence coverage the assembly gaps fall in repeat regions that cannot be spanned by single-end sequence reads (average length 109 nucleotides in this study) [34]. Fewer RNA genes are observed compared to published *Yersinia *genomes finished using traditional Sanger sequencing technology (Additional file 1), reflecting the greater difficulty of uniquely assembling repetitive sequences with single-end reads. We assessed the quality of our assemblies using metrics implemented in the *amosvalidate *package [37]. Specifically, we focused on three measures frequently correlated with assembly errors: density of polymorphisms within assembled reads, depth of coverage, and breakpoints in the alignment of unassembled reads to the final assembly. Regions in each genome where at least one measure suggested a possible mis-assembly were validated by manual inspection (Additional file 2). Many of the suspect regions corresponded to collapsed repeats, where the location of individual members of the repeat family within the genome could not be accurately determined. Based on the results of the *amosvalidate *analysis and the optical map alignment we found no evidence of mis-assemblies leading to chimeric contigs in the eight genomes we sequenced. Genomic regions flagged by the *amosvalidate *package are made available in GFF format (compatible with most genome browsers) in Additional file 3.

Genome sizes were estimated initially as the sum of the sizes of the contigs from the shotgun assembly, with corrections for contigs representing collapsed repeats (Table 2). We also derived an independent estimate for the genome size from the whole-genome optical restriction mapping of the species [38] (Additional file 4). Alignment of contigs to the optical maps [39] suggested that the optical maps consistently overestimated sizes (2 to 10% on average). After correction, the map-based estimates and sequence-based estimates agreed well (within 7%). Two species, *Y. aldovae *(4.22 to 4.33 Mbp) and *Y. ruckeri *(3.58 to 3.89 Mbp), have a substantially reduced total genome size compared with the 4.6 to 4.8 Mbp seen in the genus generally. The agreement between the optical maps and sequence-based estimates of genome sizes tallied with experimental evidence for the lack of large plasmids in the sequenced genomes (Additional file 5). A screen for matches to known plasmid genes produced only a few candidate plasmid contigs, totaling less than 10 kbp of sequence in each genome.

The number of IS elements per genome for the eight species (12 to 167 matches) discovered using the IS finder database [40] was much lower than in the *Y. pestis *genome (1,147 matches; copy numbers estimates took into account the possibility of mis-assembly and were accordingly adjusted; see Methods). Furthermore, the non-pathogenic species with the most IS matches, namely *Y. bercovieri *(167 matches), *Y. aldovae *(143 matches) and *Y. ruckeri *(136 matches), have comparatively smaller genomes. We also searched for novel repeat families using a *de novo *repeat-finder [41] and collected a non-redundant set of 44 repeat sequence families in the *Yersinia *genus (Table 3; Additional file 6). Interestingly, the well-known ERIC element [42] was recovered by our *de novo *search and was found to be present in many copies in all the pathogenic species, but was relatively rare in the non-pathogenic ones. On the other hand, a similar and recently discovered element, YPAL [43] (also recovered by the *de novo *search), was abundant in all the *Yersinia *genomes except the fish pathogen *Y. ruckeri*. Insertion sequence IS1541C in the IS finder database, which has expanded in *Y. pestis *(to more than 60 copies), had only a handful of strong matches in *Y. enterocolitica*, *Y. pseudotuberculosis*, and *Y. bercovieri *and no discernable matches in the other *Yersinia *genomes.

**Table 3:** Distribution of common repeat sequences

	ERIC (127 bp)	YPAL (167 bp)	*Kristensenii *39 (142 bp)	IS1541C (708 bp)	Aldovae3 (154 bp)
*E. coli*	0	3	5	0	5
*Y. pestis*	54	43	33	61	38
*Y. pseudotuberculosis*	55	52	29	5	36
*Y. enterocolitica*	63	144	100	3	75
*Y. aldovae*	6	84	46	0	40
*Y. bercovieri*	9	45	6	9	13
*Y. frederiksenii*	0	57	6	0	5
*Y. intermedia*	2	91	48	0	43
*Y. kristensenii*	2	99	70	0	59
*Y. mollaretii*	6	62	26	0	20
*Y. rohdei*	0	37	8	0	7
*Y. ruckeri*	45	2	0	0	2

Three of the repeat sequences found using *de novo *searches matched the known repeat elements ERIC, YPAL, and IS1541C and are identified as such. *Kristensenii*39 and Aldovae3 are elements found from *de novo *searches in the *Y. kristensenii *and *Y. aldovae *genomes, respectively.

### New *Yersinia *genome data reduce the pool of unique detection targets for *Y. pestis *and *Y. enterocolitica*

The sequences generated in this study provide new background information for validating genus detection and diagnosis assays targeting pathogenic members of the *Yersinia *genus. The assay design process commonly starts by computationally identifying genomic regions that are unique to the targeted genus ('signatures') - an ideal signature is shared by all targeted pathogens but not found in a background comprising non-pathogenic near neighbors or in other unrelated microbes. While many pathogens are well characterized at the genomic level, the background set is only sparsely represented in genomic databases, thereby limiting the ability to computationally screen out non-specific candidate assays (false positives). As a result, many assays may fail experimental field tests, thereby increasing the costs of assay development efforts. To evaluate whether the new genomic sequences generated in our study can reduce the incidence of false positives in assay development, we computed signatures for the *Y. pestis *and *Y. enterocolitica *genera using the Insignia pipeline [44], the system previously used to successfully develop assays for the detection of *V. cholerae *[44]. We identified 171 and 100 regions within the genomes of *Y. pestis *and *Y. enterocolitica*, respectively, that represent good candidates for the design of detection assays. In *Y. pestis *these regions tended to cluster around the origin of replication, whereas in *Y. enterocolitica *there was a more even distribution. The average G+C content of the regions for the unique sequences in both species was close to the *Yersinia *average (47%) and there was not a strong association with putative genome islands (Additional files 7, 8, 9, 10, 11, 12, [45]). For both species, most regions overlapped predicted genes (161 of 171 (94%) and 96 of 100 (96%) in *Y. pestis *and *Y. pseudotuberculosis*, respectively). Interestingly, 171 *Y. pestis *gene regions were spread over only 70 different genes, whereas the 96 *Y. enterocolitica *regions were found overlapping only 90 genes. There was no obvious trend in the nature of the genes harboring these putative signals except that many could be arguably classed as 'non-core' functions, encoding phage endonucleases, invasins, hemolysins and hypothetical proteins.

Ten *Y. pestis*-specific and 31 *Y. enterocolitica*-specific putative signatures have significant matches in the new genome sequence data (Additional files 7, 8, 9, 10), indicating assays designed within these regions would result in false positive results. This result underscores the need for a further sampling of genomes of the *Yersinia *genus in order to assist the design of diagnostic assays.

### *Yersinia *whole-genome comparisons

We performed a multiple alignment of the 11 *Yersinia *species using the MAUVE algorithm [46] (from here on *Y. pestis *CO92 and *Y. pseudotuberculosis *IP32953 were used as the representative genomes of their species) and obtained 98 locally collinear blocks (LCBs; Additional files 13, 14, [47]). The mean length of the LCBs was 23,891 bp. The shortest block was 1,570 bp, and the longest was 201,130 bp. This multiple alignment of the 'core' region on average covered 52% of each *Yersinia *genome. The nucleotide diversity (Π) for the concatenated aligned region was 0.27, or an approximate genus-wide nucleotide sequence homology of 73%. As expected for a set of bacteria with this level of diversity, the alignment of the genomes shows evidence of multiple large genome rearrangements [23] (Additional file 13).

**Figure 1:**
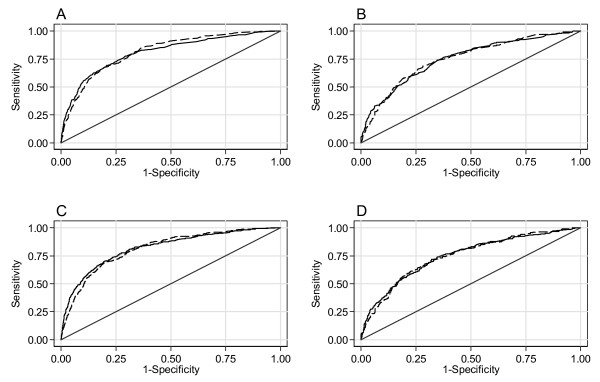
***Yersinia *whole-genome phylogeny**. The phylogeny of the *Yersinia *genus was constructed from a dataset of 681 concatenated, conserved protein sequences using the Neighbor-Joining (NJ) algorithm implemented by PHYLIP [51]. The tree was rooted using *E. coli*. The scale measures number of substitutions per residue. Tree topologies computed using maximum likelihood and parsimony estimates are identical with each other and the NJ tree (Additional file 20). The only branches not supported in more than 99% of the 1,000 bootstrap replicates using both methods are marked with asterisks. Both these branches were supported by >57% of replicates.

Using an automated pipeline for annotation and clustering of protein orthologs based on the Markov chain clustering tool MCL [48], we estimated the size of the *Yersinia *protein core set to be 2,497 and the pan-genome [49] to be 27,470 (Additional files 15, 16, 17, 18). The core number falls asymptotically as genomes are introduced and hence this estimate is somewhat lower than the recent analysis of only the *Y. enterocolitica*, *Y. pseudotuberculosis *and *Y. pestis *genomes (2,747 core proteins) [15]. We found 681 genes to be in exactly one copy in each *Yersinia *genome and to be nearly identical in length. We used ClustalW [50] to align the members of this highly conserved set, and concatenated individual gene product alignments to make a dataset of 170,940 amino acids for each of the species. Uninformative characters were removed from the dataset and a phylogeny of the genus was computed using Phylip [51] (Figure 1). The topology of this tree was identical whether distance or parsimony methods were used (Additional files 19, 20) and was also identical to a tree based on the nucleotide sequence of the approximately 1.5 Mb of the core genome in LCBs (see above). The genus broke down into three major clades: the outlying fish pathogen, *Y. ruckeri*; *Y. pestis*/*Y. pseudotuberculosis*; and the remainder of the 'enterocolitica'-like species. *Y. kristensenii *ATCC33638T was the nearest neighbor of *Y. enterocolitica *8081. The outlying position of *Y. ruckeri *was confirmed further when we analyzed the contribution of the genome to reducing the size of the *Yersinia *core protein families set. If *Y. ruckeri *was excluded, the *Yersinia *core would be 2,232 protein families of N = 2 rather than 2,072 (Table 4). In contrast, omission of any one of the 10 other species only reduced the set by a maximum of 22 families.

**Table 4:** *Yersinia *core size reduction by exclusion of one species

Species excluded	Core protein families
None	2,072
*Y. enterocolitica*	2,074
*Y. aldovae*	2,085
*Y. bercovieri*	2,079
*Y. frederiksenii*	2,077
*Y. intermedia*	2,080
*Y. kristensenii*	2,076
*Y. mollaretii*	2,078
*Y. rohdei*	2,091
*Y. ruckeri*	2,232
*Y. pseudotuberculosis*	2,076
*Y. pestis*	2,094

The core protein families with number of members 2 or greater were recalculated in each case (see Materials and methods) with the protein set from one genome missing.

**Figure 2:**
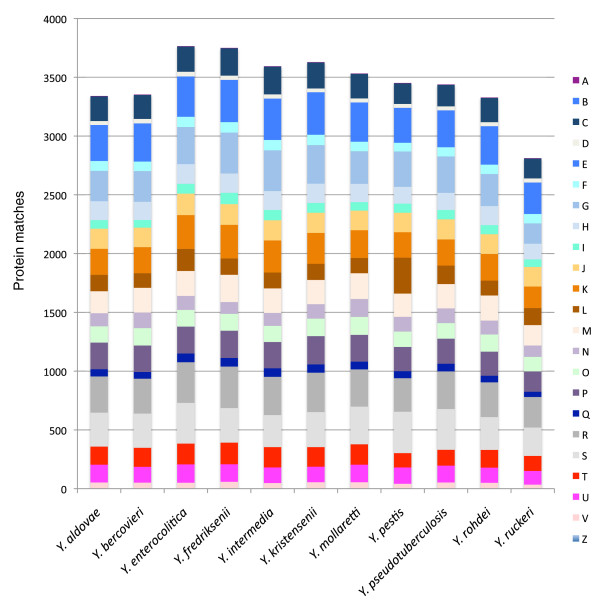
**Comparison of major COG groups in *Yersinia *genomes**. Bars represent the number of proteins assigned to COG superfamilies [52] for each genome, based on matches to the Conserved Domain Database [95] database with an E-value threshold <10^-10^. The COG groups are: U, intracellular trafficking; G, carbohydrate transport and metabolism; R, general function prediction; I, lipid transport and metabolism; D, cell cycle control; H, coenzyme transport and metabolism; B, chromatin structure; P, inorganic ion transport and metabolism; W, extracellular structures; O, post-translational modification; J, translation; A, RNA processing and editing; L, replication, recombination and repair; C, energy production; M, cell wall/membrane biogenesis; Q, secondary metabolite biosynthesis; Z, cytoskeleton; V, defense mechanisms; E, amino acid transport and metabolism; K, transcription; N, cell motility; T, signal transduction; F, nucleotide transport; S, function unknown.

Clustering the significant Cluster of Orthologous Groups (COG) hits [52] for each genome hierarchically (Figure 2) yielded a similar pattern for the three basic clades. The overall composition of the COG matches in each genome, as measured by the proportion of the numbers in each COG supercategory, was similar throughout the genus, with the notable exceptions of the high percentage of group L COGs in *Y. pestis *due to the expansion of IS recombinases and the relatively low number of group G (sugar metabolism) COGs in *Y. ruckeri *(Figure 2).

### Shared protein clusters in pathogenic *Yersinia*: yersiniabactin biosynthesis is the key chromosomal function specific to high virulence in humans

**Figure 3:**
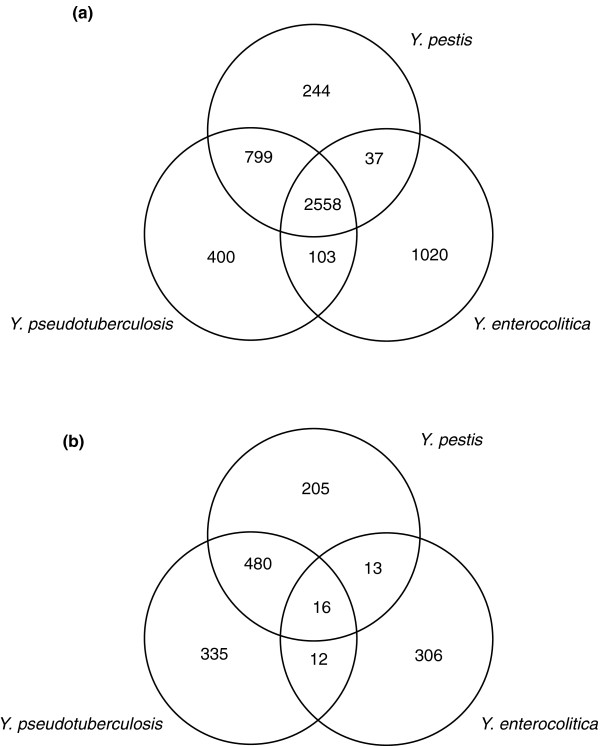
**Distribution of protein clusters across *Y. enterocolitica *8081, *Y. pestis *CO92, and *Y. pseudotuberculosis *IP32953**. **(a) **The Venn diagram shows the number of protein clusters unique or shared between the two other high virulence *Yersinia *species (see Materials and methods). **(b) **The number of shared and unique clusters that do not contain a single member of the eight low human virulence genomes sequenced in this study.

The *Yersinia *proteomes were investigated for common clusters in the three high virulence species missing from the low human virulence genomes (Figure 3). Because of the close evolutionary relationship of the 'enterocolictica' clade strains, the number of unique protein clusters in *Y. enterocolitica *was reduced to a greater degree than the more phylogentically isolated *Y. pestis *and *Y. pseudotuberculosis*. Many of the same genome islands identified as recent horizontal acquisition by *Y. pestis *and/or *Y. pseudotuberculosis *[9,13,15] were not present in any of the newly sequenced genomes. However, some genes, interesting from the perspective of the host specificity of the *Y. pestis*/*Y. pseutoberculosis *ancestor, were detected in other *Yersinia *species for the first time. These included orthologs of YPO3720/YPO3721, a hemolysin and activator protein in *Y. intermedia*, *Y. bercovieri *and *Y. fredricksenii*; YPO0599, a heme utilization protein also found in *Y. intermedia*; and YPO0399, an enhancin metalloprotease that had an ortholog in *Y. kristensenii *(ykris0001_41250). Enhancin was originally identified as a factor promoting baculovirus infection of gypsy moth midgut by degradation of mucin [53]. Other loci in *Y. pestis*/*Y. pseudotuberculosis *linked with insect infection, the TccC and TcABC toxin clusters [54], were also found in *Y. mollaretti*. In *Y. mollaretti *the Tca and Tcc proteins show about 90% sequence identity to *Y. pestis*/*Y. pseudotuberculsis *and share identical flanking chromosomal locations. Further work will need to be undertaken to resolve whether the insertion of the toxin genes in *Y. mollaretti *is an independent horizontal transfer event or occurred prior to divergence of the species.

After comparison of the new low virulence genomes, the number of protein clusters shared by *Y. enterocolitica *and the other two pathogens was reduced to 12 and 13 for *Y. pseudotuberculosis *and *Y. pestis*, respectively (Figure 3). The remaining shared proteins were either identified as phage-related or of unknown role, providing few clues to possible functions that might define distinct pathogenic niches. Performing a similar analysis strategy between others genome of the 'enterocolitica' clade and *Y. pestis *or *Y. pseudotuberculosis *gave a similar result in terms of numbers and types of shared protein clusters.

Only sixteen clusters of chromosomal proteins were found to be common to all three high-virulence species but absent from all eight non-pathogens (Figure 3). Eleven of these are components of the yersiniabactin biosynthesis operon (Additional file 21), further highlighting the critical importance of this iron binding siderophore for invasive disease. The other proteins are generally small proteins that are likely included because they fall in unassembled regions of the eight draft genomes. One other small island of three proteins constituting a multi-drug efflux pump (YE0443 to YE0445) was common to the high-virulence species but missing from the eight draft low-virulence species.

### Variable regions of *Y. enterocolitica *clade genomes

**Figure 4:**
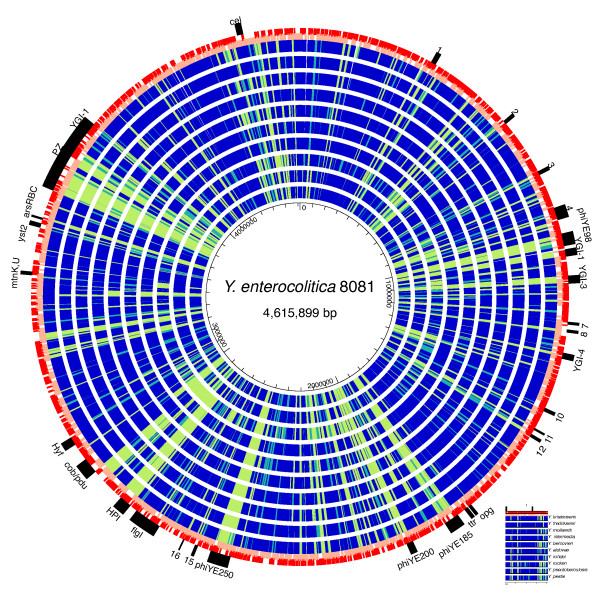
**Protein-based comparison of *Y. enterocolitica *8081 to the *Yersinia *genus**. The map represents the blast score ratio (BSR) [98,99] to the protein encoded by *Y. enterocolitica *[15]. Blue indicates a BSR >0.70 (strong match); cyan 0.69 to 0.4 (intermediate); green <0.4 (weak). Red and pink outer circles are locations of the *Y. enterocolitica *genes on the + and - strands. The genomes are ordered from outside to inside based on the greatest overall similarity to *Y. enterocolitica*: *Y. kristensenii*, *Y. frederiksenii*, *Y. mollaretii*, *Y. intermedia*, *Y. bercovieri, Y. aldovae*, *Y. rohdei*, *Y. ruckeri*, *Y. pseudotuberculosis*, and *Y. pestis*. The black bars on the outside refer to genome islands in *Y. enterocolitica *identified by Thomson *et al*. [15].

The basic metabolic similarities of *Y. enterocolitica *and the seven species on the main branch of the *Yersinia *genus phylogenetic tree are further illustrated in Figure 4, where the best protein matches against each *Y. enterocolitica *8081 gene product [15] are plotted against a circular genome map. Very few genes exclusive to *Y. enterocolitica *8081 were found outside of prophage regions, which is a typical result when groups of closely related bacterial genomes are compared [55]. One of the largest islands found in *Y. enterocolitica *8081 was the 66-kb *Y. pseudotuberculosis *adhesion pathogenicity island (YAPI_ye_) [15,56,57], a unique feature of biotype 1B strains. YAPI_ye_, containing a type IV pilus gene cluster and other putative virulence determinants, such as arsenic resistance, is similar to a 99-kb YAPI_pst _that is found in several other serotypes of *Y. pseudotuberculosis *[14,57] but is missing in *Y. pestis *and the serotype I *Y. pseudotuberculosis *strain IP32953 [14]. A model has been proposed for the acquisition of YAPI in a common ancestor of *Y. pseudotuberculosis *and *Y. enterocolitica *and subsequent degradation to various degrees within the *Y. pseudotuberculosis *clade. However, the complete absence of YAPI from any of the seven species in the *Y. enterocolitica *branch (Figure 4), as well as from most strains of *Y. enterocolitica *[15], argues against an ancient acquisition of YAPI, but instead suggests the recent independent acquisition of related islands by both *Y. enterocolitica *biogroup 1B and *Y. pseudotuberculosis*.

Many genes previously thought to be unique to *Y. enterocolitica* in general and biotype 1B in particular turned out to have orthologs in the low human virulence species sequenced in this study. These included several putative biotype 1B-specific genes identified by microarray-based screening [58], including YE0344 HylD hemophore (yinte0001_41550 has 78% nucleotide identity), YE4052 metalloprotease (yinte0001_36030 has 95% nucleotide identity), and YE4088, a two-component sensor kinase, which had orthologs in all species. Large portions of the biogroup 1B-specific island containing the Yts1 type II secretion system were found in *Y. ruckeri*, *Y. mollaretii*, and *Y. aldovae*. *Y. aldovae* and *Y. mollaretii* also had islands containing *ysa* type three secretion systems (TTSS) with 75 to 85% nucleotide identity to the homolog in *Y. enterocolitica* 1B. The *ysa*genes are a chromosomal cluster [9,13,15] that in *Y. enterocolitica*, at least, appears to play a role in virulence [59]. The *Y. enterocolitica ysa *genes are found in the plasticity zone (Figure 4) and have very low similarity to the *Y. pestis *and *Y. pseudotuberculosis ysa *genes (which are more similar to the *Salmonella *SPI-2 island [60,61]) and are found between orthologs of YPO0254 and YPO0274 [9]. Species within the *Yersinia *genus had either the *Y. enterocolitica *type of *ysa *TTSS locus or the *Y. pestis*/SPI-2 type (with the exception of *Y. aldovae*, which has both; Additional file 22). This suggested the exchange of chromosomal TTSS genes within *Yersinia*.

The modular nature of the islands found in the *Y. enterocolitica *genome was demonstrated further by two examples gleaned from comparison with the evolutionarily closest low human virulence genome, *Y. kristensenii *ATCC 33638T (Figure 1). The YGI-3 island [15] in *Y. enterocolitica *8081 is a degraded integrated plasmid; at the same chromosomal locus in *Y. kristensenii *ATCC 33638T a prophage was found, suggesting that the YGI-3 location may be a recombinational hotspot. Another *Y. enterocolitica *8081 island, YGI-1, encodes a 'tight adherence' (*tad*) locus responsible for non-specific surface binding. *Y. kristensenii *ATCC 33638T had an identical 13 gene *tad *locus in the same position, but the nucleotide sequence identity of the region to *Y. enterocolitica *8081 was uniformly lower than that found for the rest of the genome, suggesting there had been either a gene conversion event replacing the *tad *locus with a set of new alleles in the recent history of *Y. kristensenii *or *Y. enterocolitica *or the locus was under very high positive selective pressure.

### Niche-specific metabolic adaptations in the *Yersinia *genus

Comparison of the *Y. enterocolitica *genome to *Y. pestis *and *Y. pseudotuberculosis *revealed some potentially significant metabolic differences that may account for varying tropisms in gastric infections [62]. *Y. enterocolitica *8081 alone contained entire gene clusters for cobalamin (vitamin B12) biosynthesis (*cbi*), 1,2-propanediol utilization (*pdu*), and tetrathionate respiration (*ttr*). In *Y. enterocolitica *and *Salmonella typhimurium *[63,64], vitamin B12 is produced under anaerobic conditions where it is used as a cofactor in 1,2-propanediol degradation, with tetrathionate serving as an electron acceptor. This study showed the genes for this pathway to be a general feature of species in the 'enterocolitica' branch of the *Yersinia *genus (with the caveat that some portions are missing in some species; for example, *Y. rohdei *is missing the *pdu *cluster (Table 5). Additionally, *Y. intermedia*, *Y. bercovieri*, and *Y. mollaretii *contained gene clusters encoding degradation of the membrane lipid constituent ethanolamine. Ethanolamine metabolism under anaerobic conditions also requires the B12 cofactor. *Y. intermedia *contained the full 17-gene cluster reported in *S. typhimurium *[65], including structural components of the carboxysome organelle. Another discovery from the *Y. enterocolitica *genome analysis was the presence of two compact hydrogenase gene clusters, Hyd-2 and Hyd-4 [15]. Hydrogen released from fermentation by intestinal microflora is imputed to be an important energy source for enteric gut pathogens [66]. Both gene clusters are conserved across all the other seven enterocolitica-branch species, but are missing from *Y. pestis *and *Y. pseudotuberculosis*. *Y. ruckeri *contained a single [NiFe]-containing hydrogenase complex.

**Table 5:** Key niche-specific genes in *Yersinia*

	*cbi*	*pdu*	*ttr*	*eut*	*hyd-2*	*hyd-4*	*ure*	*mtn*	*opg*
*Y. enterocolitica*	+	+	+	-	+	+	+	+	+
*Y. aldovae*	+	+	-	-	+	+	+	+	+
*Y. bercovieri*	+	+	+	*eutABC*	+	+	+	+	+
*Y. frederiksenii*	+	+	+	-	+	+	+	+	+
*Y. intermedia*	+	+	+	*eutSPQTDMNEJGHABCLKR*	+	+	+	+	+
*Y. kristensenii*	+	+	+	-	+	+	+	+	+
*Y. mollaretii*	+	+	-	*eutABC*	+	+	+	+	+
*Y. rohdei*	+	-	+	-	+	+	+	+	+
*Y. ruckeri*	-	-	-	-	+/- hyfABCGHINfdhF	+/- (hyaD, hypEDB)	-	-	+
*Y. pseudotuberculosis*	-	-	-	-	-	-	+	+	+
*Y. pestis*	-	-	-	-	-	-	+/-	-	-

Abbreviations: *cbi*, cobalamin (vitamin B12) biosynthesis; *pdu*, 1,2-propanediol utilization; *ttr*, tetrathionate respiration; *eut*, ethanolamine degradation; *hyd-2 *and *hyd-4*, hydrogenases 2 and 4, respectively; *ure*, urease; *mtn*, methionine salvage pathway; *opg*, osmoprotectant (synthesis of periplasmic branched glucans).

*Y. ruckeri*, the most evolutionarily distant member of the genus (Figure 1) with the smallest genome (3.7 Mb), had several features that were distinctive from its cogeners. The *Y. ruckeri *O-antigen operon contained a *neuB *sialic acid synthase gene, therefore the bacterium was predicted to produce a sialated outer surface structure. Among the common *Yersinia *genes that are missing only in *Y. ruckeri *were those for xylose utilization and urease activity, consistent with phenotypes that have long been known in clinical microbiology [67] (Table 3). Surprisingly, we discovered that *Y. ruckeri *was also missing the *mtnKADCBEU *gene cluster that comprises the majority of the methionine salvage pathway [68] found in most other Yersiniae. These genes have also been deleted from *Y. pestis*, but as with *Y. ruckeri*, the *mtnN *(methylthioadenosine nucleosidase) is maintained. The loss of these genes in *Y. pestis *has been interpreted as a consequence of adaptation to an obligate host-dwelling lifecycle, where the availability of the sulfur-containing amino acids is not a nutritional limitation [15].

## Discussion

Whole-genome shotgun sequencing by high-throughput bead-based pyrosequencing has proved remarkably useful for the large-scale sequencing of closely related bacteria [49,69-74]. High-quality *de novo *assemblies can be obtained with relatively few errors and gaps when the sequence read coverage redundancy is 15-fold or greater. Closing all the gaps in each genome sequence is time-consuming and costly; therefore, in the near future there will be an excess of draft bacterial sequences versus closed genomes in public databases. Our analysis strategy here melds both draft and complete genomes using consistent automated annotation that is scalable to encompass potentially much larger datasets. High quality draft sequencing is likely to shortly supersede comparative genome hybridization using microarrays [25,58,75,76] as the most popular strategy for genome-wide bacterial comparisons. Genome sequence datasets can be used to shed light on the novel functions in close relatives that may have been lost in the pathogen of interest, as well as orthologs in genomes that fall below the threshold for hybridization-based detection. The problems of using microarrays for comparisons of more diverse bacterial taxa are illustrated in a study of the *Yersinia *genus, using many of the strains sequenced in this work, where the estimated number of core genes was found to be only 292 [25].

We cannot claim complete coverage of all the type strains of the *Yersinia *genus, as three new species have been created [77-79] since our work began. Nonetheless, from this extensive genomic survey we have attempted to categorize the features that define *Yersinia*. The core of about 2,500 proteins present in all 11 species is not a subset of any other enterobacterial genome. Species of the *Y. enterocolitica *clade (Figure 1) have overall a similar array of protein functions and contain a number of conserved gene clusters (cobalamin, hydrogenases, ureases, and so on) found in other bacteria (*Helicobacter*, *Campylobacte*r, *Salmonella*, *Escherichia coli*) that colonize the mammalian gut. *Y. pestis *has lost many of these genes by deletion or disruption since its split from the enteric pathogen *Y. pseudotuberculosis *and adoption of an insect vector-mediated pathogenicity mode. The smaller *Y. ruckeri *chromosome does not appear to result from recent reductive evolution (as is the case of *Y. pestis*), evidenced by the relatively low number of frameshifts and pseudogenes, and the normal amount of repetitive contigs in the *newbler *genome assembly. Like *Y. pestis*, *Y. ruckeri *lacks urease, methionine salvage genes, and B12-related metabolism. The prevailing consensus is that the pathway of transmission of red mouth disease in fish is gastrointestinal yet the similarities of *Y. ruckeri *genome reduction to *Y. pestis *hint at an alternative mode of infection for *Y. ruckeri*.

This comparative genomic study reaffirms that the distinguishing features of the high-level mammalian pathogens is the acquisition of a particular set of mobile elements: HPI, the pYV, pMT1 and pPCP plasmids, and the YADI island. However, the eight species sequenced in this study believed to have either low or zero potential for human infection, contain numerous, apparently horizontally transferred genes that would be considered putative virulence determinants if discovered in the genome of a more serious pathogen. Two examples are yaldo0001_40900 (bile salt hydrolase) and yfred0001_36480, an ortholog of the TibA adhesin of enterotoxigenic *E. coli*. Bile salt hydrolase in pathogenic *Brucella abortus *has been shown to enhance bile resistance during oral mouse infections [80] and the TibA adhesin forms a biofilm that mediates human cell invasion [81]. The low-virulence species contain a similar (and in some cases greater) number of matches to known drug resistance mechanisms that have been curated in the Antibiotic Resistance Genes Database [82] (Additional file 23, [83]). Adding DNA, stirring and reducing [17] is, therefore, the general recipe for *Yersinia *genome evolution rather than a formula specific to pathogens. Comparative genomic studies such as these can be used to enhance our ability to rapidly assess the virulence potential of a genome sequence of an emerging pathogen and we plan to continue to build more extensive databases of non-pathogenic *Yersinia *genomes that will allow us to draw conclusions with more statistical power possible than just 11 representative species.

## Conclusions

Genomes of the 11 *Yersinia *species studied range in estimated size from 3.7 to 4.8 Mb. The nucleotide diversity (Π) of the conserved backbone based on large collinear conserved blocks was calculated to be 0.27. There were no orthologs of genes and predicted proteins in the virulence-associated plasmids pYV, pMT1, and pPla, and the HPI of *Y. pestis *in the genomes of the type strains - eight non- or low-pathogenic *Yersinia *species

Apart from functions encoded on the aforementioned plasmids, HPI and YAPI regions, only nine proteins detected as common to all three *Yersinia *pathogen species (*Y. pestis*, *Y. enterocolitica *and *Y. pseudotuberculosis*) were not found on at least one of the other eight species. Therefore, our study is in agreement with the hypothesis that genes acquired by recent horizontal transfer effectively define the members of the *Yersinia *genus virulent for humans.

The core proteome of the 11 *Yersinia *species consists of approximately 2,500 proteins. *Yersinia *genomes had a similar global partition of protein functions, as measured by the distribution of COG families. Genome to genome variation in islands with genes encoding functions such as ureases, hydrogenases and B12 cofactor metabolite reactions may reflect adaptations to colonizing specific host habitats.

*Y. ruckeri*, a salmonid fish pathogen, is the earliest branching member of the genus and has the smallest genome (3.7 Mb). Like *Y. pestis*, *Y. ruckeri *lacks functional urease, methionine salvage genes, and B12-related metabolism. These losses may reflect adaptation to a lifestyle that does not include colonization of the mammalian gut.

The absence of the YAPI island in any of the seven '*Y. enterocolitica *clade' genomes likely indicates that YAPI was acquired independently in *Y. enterocolitica *and *Y. pseudotuberculosis*.

We identified 171 and 100 regions within the genomes of *Y. pestis *and *Y. enterocolitica*, respectively, that represented potential candidates for the design of nucleotide sequence-based assays for unique detection of each pathogen.

## Materials and methods

### Bacterial strains

Type strains of the eight *Yersinia *species sequenced in this study (Table 1) were acquired from the American Type Culture Collection (ATCC) and propagated at 37°C or 25°C (*Y. ruckeri*) on Luria media. DNA for genome sequencing was prepared from overnight broth cultures propagated from single colonies streaked on a Luria agar plate using the Promega Wizard Maxiprep System (Promega, Madison, WI, USA).

### Genome sequencing and assembly

Genomes were sequenced using the Genome Sequencer 20 Instrument (454 Life Sequencing Inc., Branford, CT) [34]. Libraries for sequencing were prepared from 5 μg of genomic DNA. The sequencing reads for each project were assembled *de novo *using the *newbler *program (version 01.51.02; 454 Life Sciences Inc).

### Optical mapping

Optical maps [38] for each genome using the restriction enzymes *Afl*II and *Nhe*I (*Y. aldovae *and *Y. kristensenii *only have maps for *Afl*II) were constructed by Opgen Inc. (Madison, WI). The *newbler *assemblies for each genome were scaffolded using the optical maps and the SOMA package [39] (Additional file 4). Assemblies that did not align against the optical map were tested for high read coverage, unusual GC content, and good matches to plasmid-associated genes from the ACLAME database [84] (BLAST E-value less than 10^-20^) to identify sequences that could potentially be part of an extrachromosomal element.

### Detection of disrupted genes

We used two methods for detecting disrupted proteins used. In the first method clustered protein groups were used to adduce evidence for possible gene disruption events. The clusters were parsed for pairs of proteins that met the following criteria: both from the same genome; encoded by genes located on the same strand with less than 200 bp separating their frames; and total length of the combined genes was not greater than 120% of the longest gene in the cluster. The second method used was the FSFIND algorithm [85] with a standard bacterial gene model to compare the accumulation of predicted frameshifts across different genomes.

### Assembly validation

In order to rule out artifacts due to undocumented features of the *newbler *assemblies, new assemblies were generated for validation purposes by re-mapping all the shotgun reads to the sequence of the assembled contigs using AMOScmp [86]. The resulting assembly was then subjected to analysis using the *amosvalidat*e package [37]. The output of this program includes a list of genomic regions that contain inconsistencies highlighting possible misassemblies. The resulting regions were manually inspected to reduce the possibility of assembly errors. The regions flagged by the *amosvalidate *package are provided in GFF (general feature format), compatible with most genome browsers (Additional file 3).

### Insertion sequences and *de novo *repeat finding

The presence of repeats is known to confound assembly programs and the *newbler *assembler is known to collapse high-fidelity repeat instances into a single contig. To account for the possibility of such misassemblies, we computed the copy number of contigs based on coverage statistics and used this information to correct our estimates for the abundance of classes of repeats (Additional file 3). To find known insertion sequences, the genomes were scanned for matches using the IS finder web service [40] with a BLAST E-value threshold of 10^-10 ^(matches to known repeat contigs were counted as multiple matches based on the coverage of the contig). In addition, we searched for common repeat sequences in the genome using the RepeatScout program [41] after duplicating known repeat contigs. The repeats found in each genome were collected (64 sequences) and transformed into a non-redundant set of 44 sequences using the CD-HIT program [87] (Additional file 6). The repeats found were then searched against all the genomes using BLAST with an E-value threshold of 10^-10 ^to record matches. The resultant figures for repeat content are estimations that may be lower than the true number found in the genomes.

### Finding unique DNA signatures in *Y. pestis *and *Y. enterocolitica*

DNA signatures for the *Y. pestis *and the *Y. enterocolitica *genomes were identified using the Insignia pipeline [44]. Signatures of 100 bp or longer were considered good candidates for the design of detection assays. These signatures were then compared with the genomes of the *Yersinia *strains sequenced during the current study using the MUMmer package [88] with default parameters. Signatures that matched by more than 40 bp were deemed invalidated, as they would likely lead to false-positive results.

### Automated annotation

We used DIYA [89] for automated annotation, which is a pipeline for integrating bacterial analysis tools. Using DIYA, the assemblies generated by *newbler *were scaffolded based on the optical map, concatenated, and used as a template for the programs GLIMMER [90], tRNASCAN-SE [91], and RNAmmer [92] for prediction of open reading frames and RNA genes, respectively. All predicted proteins encoded by each coding sequence were compared against a database of all proteins predicted from the canonical annotation of *Y. pestis *CO92 [9] as a preliminary screen for potentially novel functions. The GenBank format files created from the eight genomes sequenced in this study were combined with other DIYA-annotated, published whole genomes to form a dataset for analysis. All proteins were searched against the UniRef50 database (July 2008) [93] using BLASTP [94] and against the Conserved Domain Database [95] using RPSBLAST [96] with an E-value threshold of 10^-10 ^to record matches.

### Database accession numbers

The annotated genome data were submitted to NCBI GenBank and the sequence data submitted to the NCBI Short Read Archive (SRA). The accession numbers are: *Y. rohdei*, ATCC_43380: [Genbank:ACCD00000000]/[SRA:SRA009766.1]; *Y. ruckeri *ATCC_29473: [Genbank:ACCC00000000]/[SRA:SRA009767.1]; *Y. aldovae *ATCC_35236: [Genbank:ACCB00000000]/[SRA:SRA009760.1]; *Y. kristensenii *ATCC_33638: [Genbank:ACCA00000000]/[SRA:SRA009764.1]; *Y. intermedia *ATCC_29909: [Genbank:AALF00000000]/[SRA:SRA009763.1]; *Y. frederiksenii *ATCC_33641: [Genbank: AALE00000000]/[SRA:SRA009762.1]; *Y. mollaretii *ATCC_43969: [Genbank:AALD00000000]/[SRA:SRA009765.1]; *Y. bercovieri *ATCC_43970: [Genbank:AALC00000000]/[SRA:SRA009761.1].

### Whole-genome alignment using MAUVE

*Yersinia *genomes were aligned using the standard MAUVE [46] algorithm with default settings. A cutoff for 1,500 bp was set as the minimum LCB length. LCBs for each genome were extracted from the output of the program and concatenated. From the alignment nucleotide diversity was calculated by an in-house script using positions where there was a base in all 11 genomes. Because of the size of the dataset, the calculated value of Π is very robust in terms of sequence error. We calculated that 112,696 nucleotides of sequence in the concatenated core would have to be wrong to alter the estimation of *P *by ± 5% (Additional file 24). PHYLIP [51] programs were used to build a consensus tree of the MAUVE alignment with bootstrapping 1,000 replicates. The underlying model for each replicate was Fitch-Margoliash. The final phylogeny was resolved according to the majority consensus rule.

### Clustering protein orthologs

The complete predicted proteome from all genomes annotated in this study was searched against itself using BLASTP with default parameters. We removed short, spurious, and non-homologous hits by setting a bitscore/alignment length filtering threshold of 0.4 and minimum protein length of 30. Predicted proteins passing this filter were clustered into families based on these normalized distances using the MCL algorithm [48] with an inflation parameter value of 4. These parameters were based on an investigation of clustering 12 completed *E. coli *genomes, which produced very similar results to a previous study [42].

### Whole genome phylogenetic reconstruction

From the results of clustering analysis, 681 proteins were found that had exactly one member in each of the genomes and the length of each protein in the cluster was nearly identical. These protein sequences were aligned using ClustalW [50], and individual gene alignments were concatenated into a string of 170,940 amino acids for each genome. Uninformative characters were removed from the dataset using Gblocks [97] and a phylogeny reconstructed with PHYLIP [51] under a neighbor-joining model. To evaluate node support, a majority rule-consensus tree of 1,000 bootstrap replicates was computed.

## Abbreviations

ATCC: American Type Culture Collection; COG: Cluster of Orthologous Groups; HPI: high-pathogenicity island; IS: insertion sequence; LCB: locally collinear block; SRA: Short Read Archive; TTSS: type III secretion system; YAPI: *Y. pseudotuberculosis *adhesion pathogenicity island.

## Authors' contributions

TDR, MEZ, LD, and SS were involved in study design. AS, and AM were involved in materials. LD, MPKT, SL, and NNo were involved in 454 sequencing. SS, MPKT, and CC were involved in additional experiments. PEC, TDR, CC, MEZ, ACS, NN, MP, BT, and DDS were involved in data analysis. TDR, MP, and NN wrote the paper.

## Supplementary Material

Additional file 1 Statistics from running DIYA [89] and frameshift detection programs on the eight genomes sequenced in this study and various other enterobacterial genomes downloaded from NCBI. Click here for file

Additional file 2 Results of *amosvalidate *[37] analysis on the eight genomes of this study. Click here for file

Additional file 3 These consist of ISfinder [40], RepeatScout [41]and *amosvalidate *[37] results (GFF format); repeats found by RepeatScout in fasta format, scaffold files (NCBI AGP format); and information about length of contigs, read count, estimated repeat number, count in scaffold and whether or not the contig was placed by SOMA [39]. Click here for file

Additional file 4 Estimates for genome sizes (in Mbp) based on optical map data. Click here for file

Additional file 5 An *E. coli *strain with known plasmids was a positive control. Click here for file

Additional file 6 Sequences of the detected repeat families. Click here for file

Additional file 7 *Y. pestis *CO92 signatures longer than 100 bp computed by the Insignia [44] pipeline. Click here for file

Additional file 8 Sequences of the new genomes that match (that is, invalidate) the *Y. pestis *CO92 signatures listed in Additional file 7. Click here for file

Additional file 9 *Y. enterocolitica *signatures longer than 100 bp computed by the Insignia pipeline. Click here for file

Additional file 10 Sequences of the new genomes that match (that is, invalidate) the *Y. enterocolitica *signatures. Click here for file

Additional file 11 *Y. pestis *genome with the Insiginia-indentified repeats and genome islands identified using IslandViewer [45] plotted. The figure was created using DNAPlotter [106]. Click here for file

Additional file 12 *Y. enterocolitica *genome with the Insiginia-indentified repeats and genome islands identified using IslandViewer [45] plotted. The figure was created using DNAPlotter [106]. Click here for file

Additional file 13 The eight genomes sequenced in this study are represented as pseudocontigs, ordered by a combination of optical mapping and alignment to the closest completed reference genome. Click here for file

Additional file 14 Whole genome multiple alignment produced by MAUVE of the 11 *Yersinia *genomes in XMFA format [106]. Click here for file

Additional file 15 The top level directory consists of a directory called Additional_cluster_files and 5010 directories, one for each multi-protein cluster family. (This top level directory has been split into three data files for uploading purposes (Additional files 15, 16, 17).) Within the directory are the following files: PGL1_unique_*Yersinia*_unclustered.out - list of all protein singletons that MCL did not group into a cluster (see Materials and Methods); PGL1_*Yersinia*_unique_locus_tags.txt - names of the 11 locus tag prefixes used for each genome; PGL1_unique_*Yersinia*.gff - mapping each *Yersinia *protein to a cluster in tab delimited GFF; PGL1_unique_*Yersinia*.sigfile - list of the longest protein in each cluster; PGL1_unique_*Yersinia*.summary - summary table of features of each of the clusters; PGL1_unique_*Yersinia*.table - summary table of each protein in the clusters. Within each cluster directory are the following files, where 'x' is the cluster name: PGL1_unique_*Yersinia*-x.faa - multifasta file of the proteins in the cluster; PGL1_unique_*Yersinia*-x.summary - summary of the properties of the proteins; PGL1_unique_*Yersinia*-x.matches - blast matches between the proteins of the cluster; PGL1_unique_*Yersinia*-x.muscle.fasta - muscle alignment of the proteins; PGL1_unique_*Yersinia*-x.muscle.fasta.gblo - gblocks output of muscle alignment (that is, auto-trimmed alignment); PGL1_unique_*Yersinia*-x.muscle.fasta.gblo.htm - as above in html format; PGL1_unique_*Yersinia*-x.muscle.tree - treefile from muscle alignment; PGL1_unique_*Yersinia*-x.sif - matches between proteins in simple interaction format for display on graphing software. Click here for file

Additional file 16 The top level directory consists of a directory called Additional_cluster_files and 5010 directories, one for each multi-protein cluster family. (This top level directory has been split into three data files for uploading purposes (Additional files 15, 16, 17.) Within the directory are the following files: PGL1_unique_*Yersinia*_unclustered.out - list of all protein singletons that MCL did not group into a cluster (see Materials and Methods); PGL1_*Yersinia*_unique_locus_tags.txt - names of the 11 locus tag prefixes used for each genome; PGL1_unique_*Yersinia*.gff - mapping each *Yersinia *protein to a cluster in tab delimited GFF; PGL1_unique_*Yersinia*.sigfile - list of the longest protein in each cluster; PGL1_unique_*Yersinia*.summary - summary table of features of each of the clusters; PGL1_unique_*Yersinia*.table - summary table of each protein in the clusters. Within each cluster directory are the following files, where 'x' is the cluster name: PGL1_unique_*Yersinia*-x.faa - multifasta file of the proteins in the cluster; PGL1_unique_*Yersinia*-x.summary - summary of the properties of the proteins; PGL1_unique_*Yersinia*-x.matches - blast matches between the proteins of the cluster; PGL1_unique_*Yersinia*-x.muscle.fasta - muscle alignment of the proteins; PGL1_unique_*Yersinia*-x.muscle.fasta.gblo - gblocks output of muscle alignment (that is, auto-trimmed alignment); PGL1_unique_*Yersinia*-x.muscle.fasta.gblo.htm - as above in html format; PGL1_unique_*Yersinia*-x.muscle.tree - treefile from muscle alignment; PGL1_unique_*Yersinia*-x.sif - matches between proteins in simple interaction format for display on graphing software. Click here for file

Additional file 17 The top level directory consists of a directory called Additional_cluster_files and 5010 directories, one for each multi-protein cluster family. (This top level directory has been split into three data files for uploading purposes (Additional files 15, 16, 17.) Within the directory are the following files: PGL1_unique_*Yersinia*_unclustered.out - list of all protein singletons that MCL did not group into a cluster (see Materials and Methods); PGL1_*Yersinia*_unique_locus_tags.txt - names of the 11 locus tag prefixes used for each genome; PGL1_unique_*Yersinia*.gff - mapping each *Yersinia *protein to a cluster in tab delimited GFF; PGL1_unique_*Yersinia*.sigfile - list of the longest protein in each cluster; PGL1_unique_*Yersinia*.summary - summary table of features of each of the clusters; PGL1_unique_*Yersinia*.table - summary table of each protein in the clusters. Within each cluster directory are the following files, where 'x' is the cluster name: PGL1_unique_*Yersinia*-x.faa - multifasta file of the proteins in the cluster; PGL1_unique_*Yersinia*-x.summary - summary of the properties of the proteins; PGL1_unique_*Yersinia*-x.matches - blast matches between the proteins of the cluster; PGL1_unique_*Yersinia*-x.muscle.fasta - muscle alignment of the proteins; PGL1_unique_*Yersinia*-x.muscle.fasta.gblo - gblocks output of muscle alignment (that is, auto-trimmed alignment); PGL1_unique_*Yersinia*-x.muscle.fasta.gblo.htm - as above in html format; PGL1_unique_*Yersinia*-x.muscle.tree - treefile from muscle alignment; PGL1_unique_*Yersinia*-x.sif - matches between proteins in simple interaction format for display on graphing software. Click here for file

Additional file 18 Complete protein sets for the 11 species of *Yersinia*. Click here for file

Additional file 19 To evaluate node support, a majority rule-consensus tree of 1,000 bootstrap replicates was computed. *E. coli *was used as an outgroup species. Click here for file

Additional file 20 To evaluate node support, a majority rule-consensus tree of 1,000 bootstrap replicates was computed. *E. coli *was used as an outgroup species. Click here for file

Additional file 21 A curve showing the rate of decline in number of this set as more non-pathogen genomes are added is also included. Click here for file

Additional file 22 Phylogeny of TTSS component YscN in *Yersinia *and other enterobacteria species. Click here for file

Additional file 23 Putative antibiotic resistance genes in the *Yersinia *genus determined using the Antibiotic Resistance Genes Database [45]. Click here for file

Additional file 24 Calculations for the estimation of Π from aligned *Yersinia *core genomes. Click here for file
