# Supplementary material for: Genomic characterization of the Yersinia genus
Source: Genome Biol. 2010 Jan 4;11(1):R1. doi: 10.1186/gb-2010-11-1-r1 (PMC2847712; doi:10.1186/gb-2010-11-1-r1)
Supplement: Additional file 2 — Results of amosvalidate [37] analysis on the eight genomes of this study. [file gb-2010-11-1-r1-S2.doc]

Number of regions identified within the 8 Yersinia genomes by Amosvalidate. High polymorphism rate indicates regions in the assembly where multiple high-quality polymorphisms were found within 500bp from each other. A polymorphism was considered high-quality if two or more reads disagree with the consensus sequence and the sum of their quality values exceeds 40 (likelihood of error lower than 1 in 10,000). Manual inspection of these polymorphisms indicated that the majority are due to homopolymer tracts. High coverage - indicates regions in the assembly that are deeper than 3 times the average coverage for the genome. Break - indicates a region in the assembly where two or more singleton reads disagree with the contig structure, i.e. these reads only partially align to the contig indicating an alternative assembly of this genomic region is possible. Breaks near contig end indicates features that occur within 20bp from the end of a contig. Such breaks are often an end-of-contig artifact rather than an indication of mis-assembly.
