# Supplementary material for: Genomic characterization of the Yersinia genus
Source: Genome Biol. 2010 Jan 4;11(1):R1. doi: 10.1186/gb-2010-11-1-r1 (PMC2847712; doi:10.1186/gb-2010-11-1-r1)
Supplement: Additional file 4 — Estimates for genome sizes (in Mbp) based on optical map data. [file gb-2010-11-1-r1-S4.doc]

**Additional file 3 – Estimates for Genome Sizes (in Mbp).**

| Species | Sequence in contigs (> 200 bp) | Sequence in contigs (≤ 200 bp) | Genome size (sequence estimate) | Optical map size | Genome size (map estimate) | Scaffold size |
| --- | --- | --- | --- | --- | --- | --- |
| *Y. aldovae* | 4.29 | 0.03 | 4.33 | AflII: 4.30 | AflII: 4.22 | AflII: 4.22 |
| *Y. bercovieri* | 4.32 | 0.11 | 4.37 | AflII: 4.54  NheI: 4.50 | AflII: 4.19  NheI: 4.24 | AflII: 4.51  NheI: 4.52 |
| *Y. frederiksenii* | 4.87 | 0.12 | 4.90 | AflII: 5.34  NheI: 5.40 | AflII: 4.96  NheI: 4.88 | AflII: 5.31  NheI: 5.30 |
| *Y. intermedia* | 4.69 | 0.13 | 4.71 | AflII: 4.95  NheI: 5.07 | AflII: 4.74  NheI: 4.56 | AflII: 5.03  NheI: 5.00 |
| *Y. kristensenii* | 4.65 | 0.04 | 4.77 | AflII: 4.63 | AflII: 4.46 | AflII: 4.65 |
| *Y. mollaretii* | 4.54 | 0.16 | 4.57 | AflII: 4.93  NheI: 4.92 | AflII: 4.75  NheI: 4.57 | AflII: 4.88  NheI: 4.86 |
| *Y. rohdei* | 4.31 | 0.02 | 4.34 | AflII: 4.65  NheI: 4.65 | AflII: 4.30  NheI: 4.20 | AflII: 4.56  NheI: 4.55 |
| *Y. ruckeri* | 3.73 | 0.03 | 3.79 | AflII: 3.90  NheI: 3.96 | AflII: 3.76  NheI: 3.58 | AflII: 3.89  NheI: 3.85 |

Repeat contigs were identified based on high coverage, and the estimated “copy number” was used to compute the sequence estimate. Contigs mapped onto the optical map were used to estimate the expansion in optical map size and this was used to compute the map estimate of genome size.
