# Supplementary material for: Genomic characterization of the Yersinia genus
Source: Genome Biol. 2010 Jan 4;11(1):R1. doi: 10.1186/gb-2010-11-1-r1 (PMC2847712; doi:10.1186/gb-2010-11-1-r1)
Supplement: Additional file 5 — An E. coli strain with known plasmids was a positive control. [file gb-2010-11-1-r1-S5.doc]

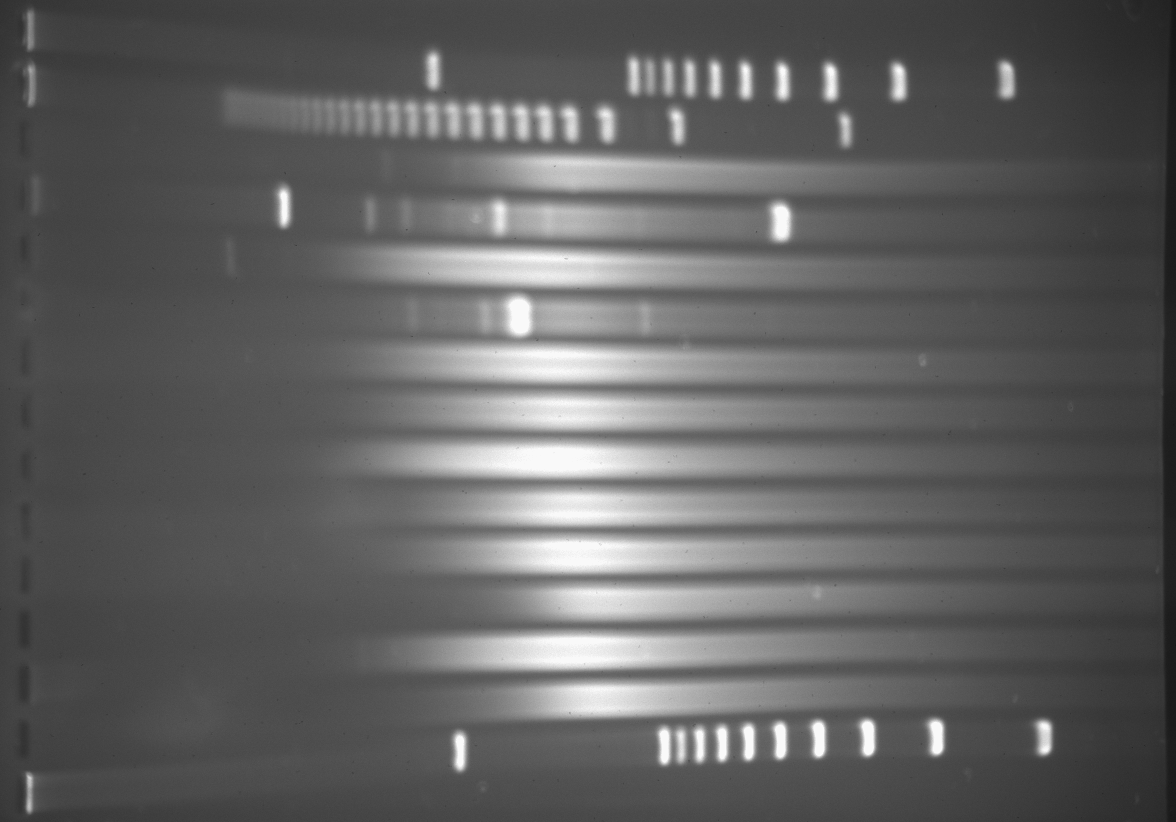


***Yersiniae***

**1. *Y. aldovae***

**2. *Y. bercoverii***

**3. *Y. frederiksenii***

**4. *Y. intermedia***

**5. *Y. kristensenii***

**6. *Y. mollaretii***

**7. *Y. rohdei***

**8. *Y. ruckeri***

***E. coli***

**1. Y1088**

**2. C600/P1**

**3. GM119/pRK2/pINT**

**4. INV110**

48 kb

20 kb

12 kb

5 kb

4 kb

3 kb

5. 8 kb

60 kb

93 kb

*

***Yersiniae Yersiniae***

***E.coli***

**1 2 3 4**

**1 2 3 4 5 6 7 8**

5 kb ladder

1 kb ladder

1 kb ladder

48 kb

Legend: Pulse field gel electrophoresis of plasmid preparations from Yersiniae strains and known *E. coli* strains carrying different sized plasmids. PFGE gel (1% agarose) was run under the following conditions in 0.5X TBE buffer at 14°C, switch time 1-6 seconds, for 18 hrs at a voltage gradient of 6V/cm
