# Supplementary figures and images for: Genomic characterization of the Yersinia genus
Source: Genome Biol. 2010 Jan 4;11(1):R1. doi: 10.1186/gb-2010-11-1-r1 (PMC2847712; doi:10.1186/gb-2010-11-1-r1)

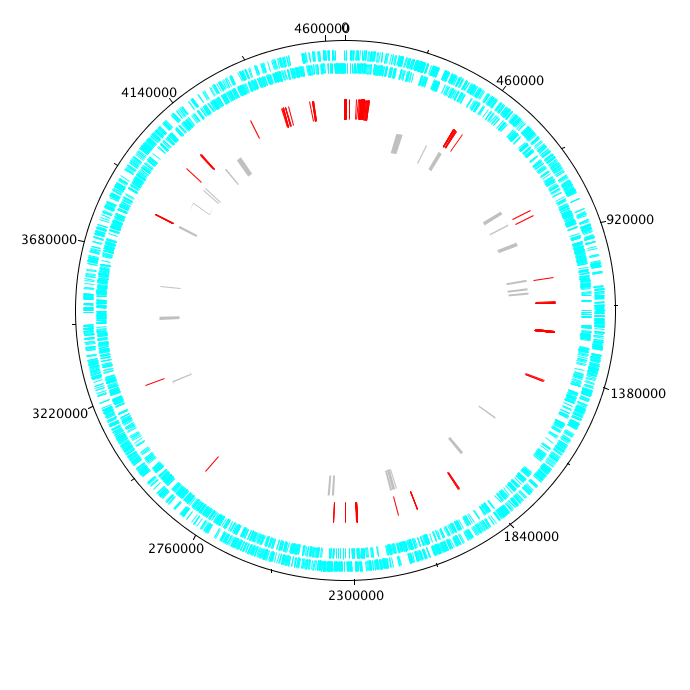

Supplement: Additional file 11 — Y. pestis genome with the Insiginia-indentified repeats and genome islands identified using IslandViewer [45] plotted. The figure was created using DNAPlotter [106]. [file gb-2010-11-1-r1-S11.png]

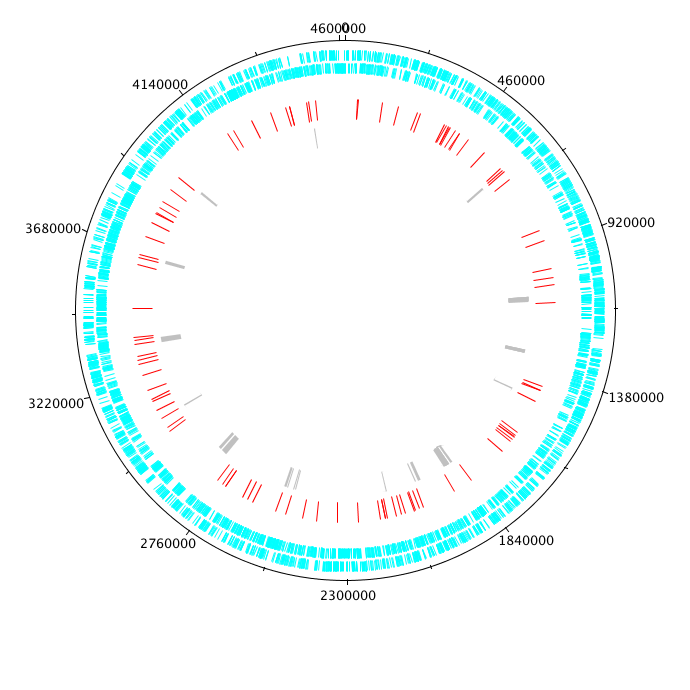

Supplement: Additional file 12 — Y. enterocolitica genome with the Insiginia-indentified repeats and genome islands identified using IslandViewer [45] plotted. The figure was created using DNAPlotter [106]. [file gb-2010-11-1-r1-S12.png]

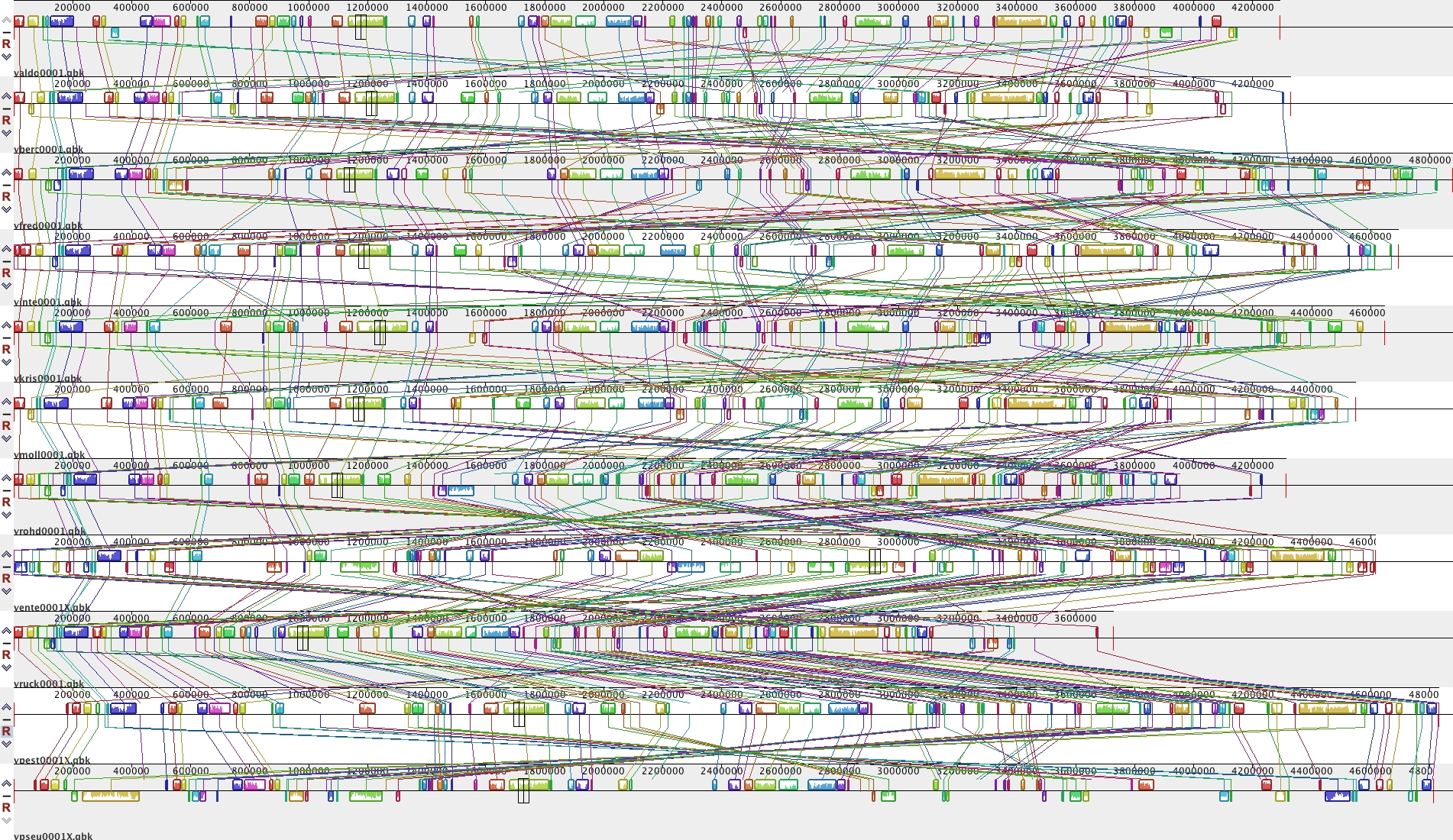

Supplement: Additional file 13 — The eight genomes sequenced in this study are represented as pseudocontigs, ordered by a combination of optical mapping and alignment to the closest completed reference genome. [file gb-2010-11-1-r1-S13.jpeg]

# Parsimony Tree (with percent bootstrap support at nodes)

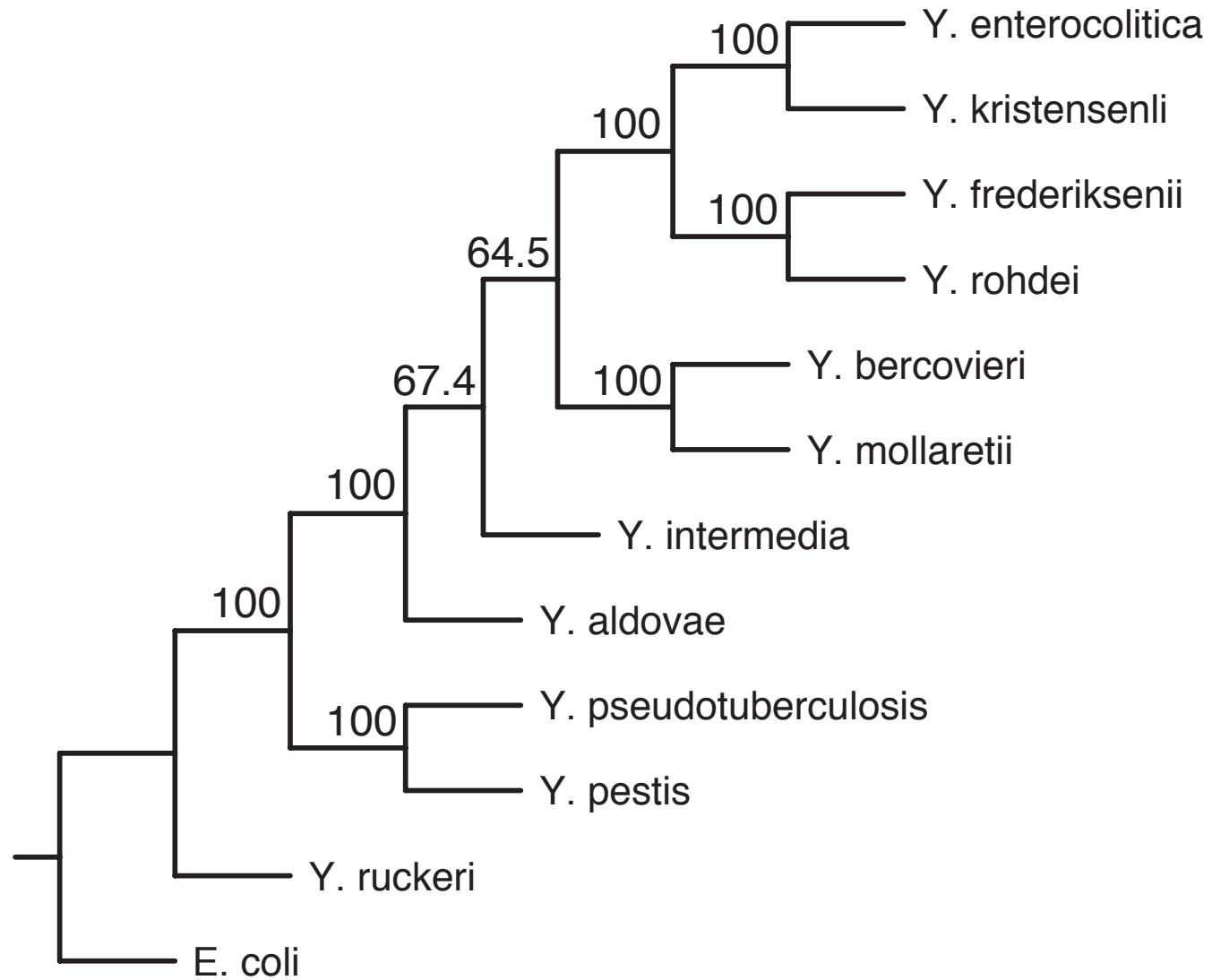

Supplement: Additional file 19 — To evaluate node support, a majority rule-consensus tree of 1,000 bootstrap replicates was computed. E. coli was used as an outgroup species. [file gb-2010-11-1-r1-S19.pdf]

Maximum Likelihood Tree (with percent bootstrap support at nodes)

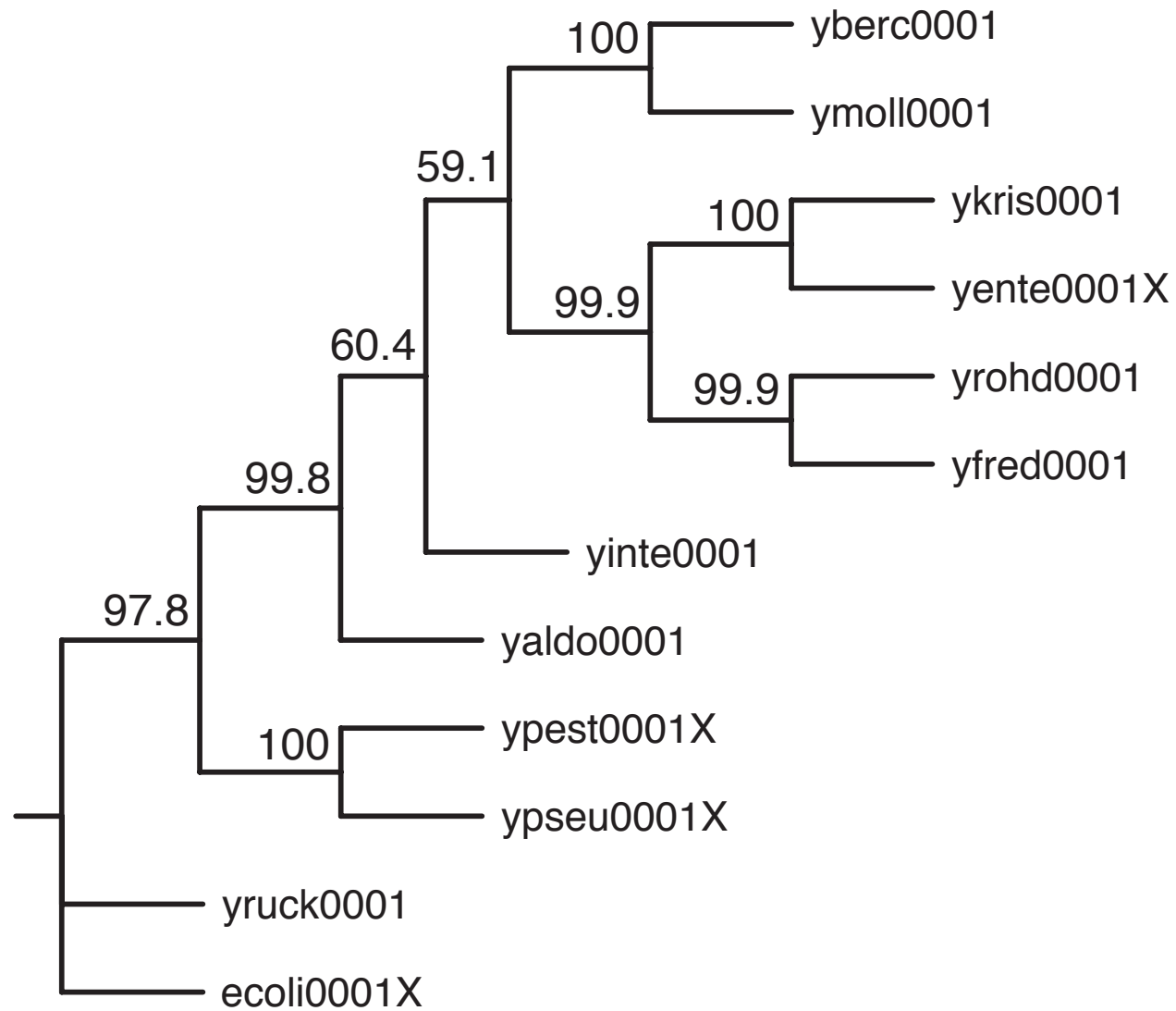

Supplement: Additional file 20 — To evaluate node support, a majority rule-consensus tree of 1,000 bootstrap replicates was computed. E. coli was used as an outgroup species. [file gb-2010-11-1-r1-S20.pdf]
