# Supplementary material for: Genomic characterization of the Yersinia genus
Source: Genome Biol. 2010 Jan 4;11(1):R1. doi: 10.1186/gb-2010-11-1-r1 (PMC2847712; doi:10.1186/gb-2010-11-1-r1)
Supplement: Additional file 15 — The top level directory consists of a directory called Additional_cluster_files and 5010 directories, one for each multi-protein cluster family. (This top level directory has been split into three data files for uploading purposes (Additional files 15, 16, 17).) Within the directory are the following files: PGL1_unique_Yersinia_unclustered.out - list of all protein singletons that MCL did not group into a cluster (see Materials and Methods); PGL1_Yersinia_unique_locus_tags.txt - names of the 11 locus tag prefixes used for each genome; PGL1_unique_Yersinia.gff - mapping each Yersinia protein to a cluster in tab delimited GFF; PGL1_unique_Yersinia.sigfile - list of the longest protein in each cluster; PGL1_unique_Yersinia.summary - summary table of features of each of the clusters; PGL1_unique_Yersinia.table - summary table of each protein in the clusters. Within each cluster directory are the following files, where 'x' is the cluster name: PGL1_unique_Yersinia-x.faa - multifasta file of the proteins in the cluster; PGL1_unique_Yersinia-x.summary - summary of the properties of the proteins; PGL1_unique_Yersinia-x.matches - blast matches between the proteins of the cluster; PGL1_unique_Yersinia-x.muscle.fasta - muscle alignment of the proteins; PGL1_unique_Yersinia-x.muscle.fasta.gblo - gblocks output of muscle alignment (that is, auto-trimmed alignment); PGL1_unique_Yersinia-x.muscle.fasta.gblo.htm - as above in html format; PGL1_unique_Yersinia-x.muscle.tree - treefile from muscle alignment; PGL1_unique_Yersinia-x.sif - matches between proteins in simple interaction format for display on graphing software. [file gb-2010-11-1-r1-S15.zip › clusters/PGL1_unique_yersinia-CL0/PGL1_unique_yersinia-CL0.muscle.fasta.gblo.htm]

PGL1\_unique\_yersinia-CL0.muscle.fasta


## Gblocks 0.91b Results

Processed file: **PGL1\_unique\_yersinia-CL0.muscle.fasta**  
Number of sequences: **144**  
Alignment assumed to be: **Protein**  
New number of positions: **50** (selected positions are underlined in blue)

```
                         10        20        30        40        50        60
                 =========+=========+=========+=========+=========+=========+
ykris0001_41300  ------------------------------------------------------------
yrohd0001_20110  ------------------------------------------------------------
yaldo0001_2440   ------------------------------MAIWLISMAVTAFFSYENTRQRLINELTNM
ypseu0001X_4220  -----------MGKASSLVTRLTLLLGVTLTVIWLILIATTAFFSYENTRQILINELTHM
ypest0001X_4540  -------------------------MGVTLTVIWLILIATTAFFSYENTRQILINELTHM
yinte0001_2660   ------------------------------------------------------------
yberc0001_2350   -----------------------------------------------------VNELTHM
ymoll0001_1840   LWRTEKNSGDFMDTSSSLVTRLTLILGMTLMAIGLISISATALFSYEDTRQRRVNELTHK
ykris0001_2490   ---------------------LTFLLGITLTAIWLISIATTAFFSYEDIRQRLVNELTHM
yinte0001_2650   ------------------------------------------------------------
yrohd0001_2860   -----------MENSSSLVTRLTLLLGITLTAIWLISIATTAFFSYEDTRQRLINELTLM
yfred0001_38320  -----------------------------------------------------------M
yinte0001_26450  ------------------------------------------------------------
yberc0001_7120   ------------------------------------------------------------
ypseu0001X_3409  ------------------------------------------------------------
ypest0001X_1004  ------------------------------------------------------------
yente0001X_9160  ------------------------------------------------------------
ykris0001_6100   ------------------------------------------------------------
yfred0001_43450  ------------------------------------------------------------
ymoll0001_7580   ------------------------------------------------------------
yinte0001_8280   ------------------------------------------------------------
ykris0001_6110   ------------------------------------------------------------
yrohd0001_35950  ------------------------------------------------------------
ypseu0001X_2873  ------------------------------------------------------------
ypest0001X_2787  ------------------------------------------------------------
ymoll0001_11800  ------------------------------------------------------------
yfred0001_8890   ------------------------------------------------------------
ykris0001_12740  ------------------------------------------------------------
yente0001X_1434  ------------------------------------------------------------
yrohd0001_31880  ------------------------------------------------------------
yrohd0001_15520  ------------------------------------------------------------
yfred0001_15260  ------------------------------------------------------------
yinte0001_16590  ------------------------------------------------------------
ymoll0001_41560  ------------------------------------------------------------
yberc0001_16020  ------------------------------------------------------------
yberc0001_5770   ------------------------------------------------------------
ymoll0001_5080   ------------------------------------------------------------
yaldo0001_37020  ------------------------------------------------------------
ypseu0001X_2733  ------------------------------------------------------------
ypest0001X_1885  ------------------------------------------------------------
yrohd0001_12010  ------------------------------------------------------------
yfred0001_12940  ------------------------------------------------------------
yinte0001_14220  ------------------------------------------------------------
ymoll0001_14640  ------------------------------------------------------------
yberc0001_15000  ------------------------------------------------------------
ykris0001_42780  ------------------------------------------------------------
yente0001X_2568  ------------------------------------------------------------
yberc0001_5850   ------------------------------------------------------------
ymoll0001_5160   ------------------------------------------------------------
yberc0001_6170   ------------------------------------------------------------
yaldo0001_6750   ------------------------------------------------------------
yfred0001_39550  ------------------------------------------------------------
yrohd0001_27170  ------------------------------------------------------------
ykris0001_32930  ------------------------------------------------------------
yente0001X_3909  ------------------------------------------------------------
ymoll0001_40600  ------------------------------------------------------------
yberc0001_29090  ------------------------------------------------------------
ypseu0001X_1449  ------------------------------------------------------------
ypest0001X_1447  ------------------------------------------------------------
yfred0001_36240  ------------------------------------------------------------
yinte0001_10140  ------------------------------------------------------------
ykris0001_10260  ------------------------------------------------------------
yente0001X_2906  ------------------------------------------------------------
yfred0001_36230  ------------------------------------------------------------
ymoll0001_9480   ------------------------------------------------------------
yberc0001_9790   ------------------------------------------------------------
yruck0001_28810  ------------------------------------------------------------
yrohd0001_31660  ------------------------------------------------------------
yaldo0001_35590  ------------------------------------------------------------
ykris0001_37060  ------------------------------------------------------------
yinte0001_37560  ------------------------------------------------------------
yfred0001_32450  ------------------------------------------------------------
yente0001X_300   ------------------------------------------------------------
ymoll0001_35290  ------------------------------------------------------------
yberc0001_34000  ------------------------------------------------------------
ykris0001_22290  ------------------------------------------------------------
yinte0001_3170   ------------------------------------------------------------
yruck0001_31650  ------------------------------------------------------------
ymoll0001_7110   ------------------------------------------------------------
yruck0001_28370  ------------------------------------------------------------
ypseu0001X_4346  ------------------------------------------------------------
ypest0001X_4370  ------------------------------------------------------------
yrohd0001_31240  ------------------------------------------------------------
yaldo0001_35130  ------------------------------------------------------------
yinte0001_36970  ------------------------------------------------------------
ykris0001_39870  ------------------------------------------------------------
ymoll0001_34760  ------------------------------------------------------------
yfred0001_31920  ------------------------------------------------------------
yberc0001_33520  ------------------------------------------------------------
yente0001X_760   ------------------------------------------------------------
yruck0001_18270  ------------------------------------------------------------
yrohd0001_33070  ------------------------------------------------------------
yfred0001_34740  ------------------------------------------------------------
yaldo0001_22600  ------------------------------------------------------------
yinte0001_40830  ------------------------------------------------------------
ymoll0001_38830  ------------------------------------------------------------
yberc0001_21820  ------------------------------------------------------------
ykris0001_22260  ------------------------------------------------------------
yente0001X_1297  ------------------------------------------------------------
yruck0001_11920  ------------------------------------------------------------
ypseu0001X_2707  ------------------------------------------------------------
ypest0001X_1916  ------------------------------------------------------------
yrohd0001_33620  ------------------------------------------------------------
yaldo0001_15230  ------------------------------------------------------------
ykris0001_37850  ------------------------------------------------------------
yente0001X_1721  ------------------------------------------------------------
ymoll0001_37090  ------------------------------------------------------------
yberc0001_15260  ------------------------------------------------------------
yinte0001_40500  ------------------------------------------------------------
yfred0001_14080  ------------------------------------------------------------
yruck0001_11930  ------------------------------------------------------------
yaldo0001_6760   ------------------------------------------------------------
ypest0001X_1918  ------------------------------------------------------------
ypest0001X_1917  ------------------------------------------------------------
ypseu0001X_2706  ------------------------------------------------------------
yrohd0001_33630  ------------------------------------------------------------
yaldo0001_15240  ------------------------------------------------------------
ymoll0001_14920  ------------------------------------------------------------
yberc0001_15270  ------------------------------------------------------------
yfred0001_14090  ------------------------------------------------------------
yinte0001_15900  ------------------------------------------------------------
ykris0001_37840  ------------------------------------------------------------
yente0001X_1722  ------------------------------------------------------------
yruck0001_10900  ------------------------------------------------------------
yaldo0001_6770   ------------------------------------------------------------
yaldo0001_15110  ------------------------------------------------------------
yinte0001_40380  ------------------------------------------------------------
ymoll0001_13500  ------------------------------------------------------------
yberc0001_14860  ------------------------------------------------------------
yrohd0001_33510  ------------------------------------------------------------
yfred0001_13960  ------------------------------------------------------------
yente0001X_1708  ------------------------------------------------------------
ykris0001_38040  ------------------------------------------------------------
yrohd0001_6540   ------------------------------------------------------------
yente0001X_6250  ------------------------------------------------------------
yruck0001_10760  ------------------------------------------------------------
ypest0001X_1900  ------------------------------------------------------------
ypseu0001X_2718  ------------------------------------------------------------
yaldo0001_37010  ------------------------------------------------------------
ypest0001X_1899  ------------------------------------------------------------
yfred0001_6650   ------------------------------------------------------------
yinte0001_6950   ------------------------------------------------------------
ymoll0001_37360  ------------------------------------------------------------
yberc0001_8160   ------------------------------------------------------------
                                                                             


                         70        80        90       100       110       120
                 =========+=========+=========+=========+=========+=========+
ykris0001_41300  ------------------------------------------------------------
yrohd0001_20110  ------------------------------------------------------------
yaldo0001_2440   AELRADLSNHQFEGAERDAASLINRWKNNQATWPLFAVNTTSDDT--IPFSLERCRRSQH
ypseu0001X_4220  ASMRADLSNHQFEGAERDAASLISRRESLQSTSPLPEISIKHYDSCYIPFNLDSCNINQH
ypest0001X_4540  ASMRADLSNHQFEGAERDAASLISRRESLQSTSPLPEISIKHYDSYYIPFNLDSCNINQH
yinte0001_2660   ------------------------------------------------------------
yberc0001_2350   ASLRADLSNYQFEGAERDAASLINHQISYQSIWRLPIPHLESDTDNSVPFSSGNCSVIQS
ymoll0001_1840   ASLRANLSNDQFEGAERDAASFINHQT---------------------------------
ykris0001_2490   ASLRADLSNYQFEGAERDAISLINRQTRHQMNWGFP-IPLKGENMDNTLVNSIICNTPQF
yinte0001_2650   ------------------------------------------------------------
yrohd0001_2860   ASLRADLSNYQFEGAERDASSLISRQSHYQTSWRFAMPLQENNADINIPFNSVSCHTLQD
yfred0001_38320  ALLRTDLSNYQFEGAERDATSLINSQVNYQAGRPLPVSFKKNDINNNNLINSVNCNSPQS
yinte0001_26450  ------------------------------------------------------------
yberc0001_7120   ------------------------------------------------------------
ypseu0001X_3409  ------------------------------------------------------------
ypest0001X_1004  ------------------------------------------------------------
yente0001X_9160  ------------------------------------------------------------
ykris0001_6100   ------------------------------------------------------------
yfred0001_43450  ------------------------------------------------------------
ymoll0001_7580   ------------------------------------------------------------
yinte0001_8280   ------------------------------------------------------------
ykris0001_6110   ------------------------------------------------------------
yrohd0001_35950  ------------------------------------------------------------
ypseu0001X_2873  ------------------------------------------------------------
ypest0001X_2787  ------------------------------------------------------------
ymoll0001_11800  ------------------------------------------------------------
yfred0001_8890   ------------------------------------------------------------
ykris0001_12740  ------------------------------------------------------------
yente0001X_1434  ------------------------------------------------------------
yrohd0001_31880  ------------------------------------------------------------
yrohd0001_15520  ------------------------------------------------------------
yfred0001_15260  ------------------------------------------------------------
yinte0001_16590  ------------------------------------------------------------
ymoll0001_41560  ------------------------------------------------------------
yberc0001_16020  ------------------------------------------------------------
yberc0001_5770   ------------------------------------------------------------
ymoll0001_5080   ------------------------------------------------------------
yaldo0001_37020  ------------------------------------------------------------
ypseu0001X_2733  ------------------------------------------------------------
ypest0001X_1885  ------------------------------------------------------------
yrohd0001_12010  ------------------------------------------------------------
yfred0001_12940  ------------------------------------------------------------
yinte0001_14220  ------------------------------------------------------------
ymoll0001_14640  ------------------------------------------------------------
yberc0001_15000  ------------------------------------------------------------
ykris0001_42780  ------------------------------------------------------------
yente0001X_2568  ------------------------------------------------------------
yberc0001_5850   ------------------------------------------------------------
ymoll0001_5160   ------------------------------------------------------------
yberc0001_6170   ------------------------------------------------------------
yaldo0001_6750   ------------------------------------------------------------
yfred0001_39550  ------------------------------------------------------------
yrohd0001_27170  ------------------------------------------------------------
ykris0001_32930  ------------------------------------------------------------
yente0001X_3909  ------------------------------------------------------------
ymoll0001_40600  ------------------------------------------------------------
yberc0001_29090  ------------------------------------------------------------
ypseu0001X_1449  ------------------------------------------------------------
ypest0001X_1447  ------------------------------------------------------------
yfred0001_36240  ------------------------------------------------------------
yinte0001_10140  ------------------------------------------------------------
ykris0001_10260  ------------------------------------------------------------
yente0001X_2906  ------------------------------------------------------------
yfred0001_36230  ------------------------------------------------------------
ymoll0001_9480   ------------------------------------------------------------
yberc0001_9790   ------------------------------------------------------------
yruck0001_28810  ------------------------------------------------------------
yrohd0001_31660  ------------------------------------------------------------
yaldo0001_35590  ------------------------------------------------------------
ykris0001_37060  ------------------------------------------------------------
yinte0001_37560  ------------------------------------------------------------
yfred0001_32450  ------------------------------------------------------------
yente0001X_300   ------------------------------------------------------------
ymoll0001_35290  ------------------------------------------------------------
yberc0001_34000  ------------------------------------------------------------
ykris0001_22290  ------------------------------------------------------------
yinte0001_3170   ------------------------------------------------------------
yruck0001_31650  ------------------------------------------------------------
ymoll0001_7110   ------------------------------------------------------------
yruck0001_28370  ------------------------------------------------------------
ypseu0001X_4346  ------------------------------------------------------------
ypest0001X_4370  ------------------------------------------------------------
yrohd0001_31240  ------------------------------------------------------------
yaldo0001_35130  ------------------------------------------------------------
yinte0001_36970  ------------------------------------------------------------
ykris0001_39870  ------------------------------------------------------------
ymoll0001_34760  ------------------------------------------------------------
yfred0001_31920  ------------------------------------------------------------
yberc0001_33520  ------------------------------------------------------------
yente0001X_760   ------------------------------------------------------------
yruck0001_18270  ------------------------------------------------------------
yrohd0001_33070  ------------------------------------------------------------
yfred0001_34740  ------------------------------------------------------------
yaldo0001_22600  ------------------------------------------------------------
yinte0001_40830  ------------------------------------------------------------
ymoll0001_38830  ------------------------------------------------------------
yberc0001_21820  ------------------------------------------------------------
ykris0001_22260  ------------------------------------------------------------
yente0001X_1297  ------------------------------------------------------------
yruck0001_11920  ------------------------------------------------------------
ypseu0001X_2707  ------------------------------------------------------------
ypest0001X_1916  ------------------------------------------------------------
yrohd0001_33620  ------------------------------------------------------------
yaldo0001_15230  ------------------------------------------------------------
ykris0001_37850  ------------------------------------------------------------
yente0001X_1721  ------------------------------------------------------------
ymoll0001_37090  ------------------------------------------------------------
yberc0001_15260  ------------------------------------------------------------
yinte0001_40500  ------------------------------------------------------------
yfred0001_14080  ------------------------------------------------------------
yruck0001_11930  ------------------------------------------------------------
yaldo0001_6760   ------------------------------------------------------------
ypest0001X_1918  ------------------------------------------------------------
ypest0001X_1917  ------------------------------------------------------------
ypseu0001X_2706  ------------------------------------------------------------
yrohd0001_33630  ------------------------------------------------------------
yaldo0001_15240  ------------------------------------------------------------
ymoll0001_14920  ------------------------------------------------------------
yberc0001_15270  ------------------------------------------------------------
yfred0001_14090  ------------------------------------------------------------
yinte0001_15900  ------------------------------------------------------------
ykris0001_37840  ------------------------------------------------------------
yente0001X_1722  ------------------------------------------------------------
yruck0001_10900  ------------------------------------------------------------
yaldo0001_6770   ------------------------------------------------------------
yaldo0001_15110  ------------------------------------------------------------
yinte0001_40380  ------------------------------------------------------------
ymoll0001_13500  ------------------------------------------------------------
yberc0001_14860  ------------------------------------------------------------
yrohd0001_33510  ------------------------------------------------------------
yfred0001_13960  ------------------------------------------------------------
yente0001X_1708  ------------------------------------------------------------
ykris0001_38040  ------------------------------------------------------------
yrohd0001_6540   -------------------------------------------------LLLIGGCDLLY
yente0001X_6250  ------------------------------------------------------------
yruck0001_10760  ------------------------------------------------------------
ypest0001X_1900  ------------------------------------------------------------
ypseu0001X_2718  ------------------------------------------------------------
yaldo0001_37010  ------------------------------------------------------------
ypest0001X_1899  ------------------------------------------------------------
yfred0001_6650   ------------------------------------------------------------
yinte0001_6950   ------------------------------------------------------------
ymoll0001_37360  ------------------------------------------------------------
yberc0001_8160   ------------------------------------------------------------
                                                                             


                        130       140       150       160       170       180
                 =========+=========+=========+=========+=========+=========+
ykris0001_41300  ------------------------------------------------------------
yrohd0001_20110  ------------------------------------------------------------
yaldo0001_2440   KNDLQIIQAYGTVGQTYYLDSFIMRKNSDITLFRPQKVSSQYLNQRRKELLLLPIFPTHN
ypseu0001X_4220  KNDLWIIQAYGTAGQTYYLDSFIIKQKEGIVLFPPQKSSSDYLNQRRKDLLLLPKFPTHN
ypest0001X_4540  KNDLWIIQAYGTAGQTYYLDSFIIKQKEGIVLFPPQKSSSDYLNQRRKDLLLLPKFPTHN
yinte0001_2660   -------------------------------------MENSSSLVTRLTLLLGMTLMAIW
yberc0001_2350   RHNYPITQAYGNSGQTYYLDSFTINSKNGITLFRPQSVSINYLSQRRKELLLLPIFPSHD
ymoll0001_1840   ---------------TYHLDSFTINSKDGITLFRSQSVSNDYLSQRHNELLLLPLFPTHG
ykris0001_2490   KHDLKVTQAYGTSGQTYYLDSFTIKRDQGITIFKPQDVSNDYLQQRRKELILLPVFPTHD
yinte0001_2650   ------------------------------------------LHQLPFALAILLLLTLVL
yrohd0001_2860   KHDLQVTQAYATSGQTYYLDSFTINQKEGITIFKPQQVSREYLNQRRKELLLLPIFPTHN
yfred0001_38320  KHSLPITQAYGTAGQTYYLDSFTINRKDGITIFKPQEVSNDYLKQRRKELQLLPIFPTHD
yinte0001_26450  ------------------------------------------------------------
yberc0001_7120   ------------------------------------------------------------
ypseu0001X_3409  ---------------------MFSRMNYVVKKYFGYIMSKLSLSESRLGILFGFTLVISL
ypest0001X_1004  ---------------------MFSRMNYVVKKYFGYIMSKLSLSESRLGILFGFTLVISL
yente0001X_9160  ------------------------------------------------------------
ykris0001_6100   ------------------------------------------------------------
yfred0001_43450  ---------------------------------MFNVKSIYSFTNGKFGILFGLILVISL
ymoll0001_7580   ---------------------MFSRIRYFVIKVASNVKSLGLFTDGRLGILSGLIVVITL
yinte0001_8280   ------------------------------VKLTSYVRRLGSISDGKFGILFGLILVISL
ykris0001_6110   ------------------------------------------------------------
yrohd0001_35950  -----------------------------------------------MKLNLWPSQALWR
ypseu0001X_2873  -------------------------------------------MLFNFRSSHAPWRAELT
ypest0001X_2787  ---------------------------------------LGVDHYCTVRYTHAPWRAELT
ymoll0001_11800  -----------------------------------------------MLFNFSSSHASSR
yfred0001_8890   -----------------------------------------------MLFNFSSSHASPR
ykris0001_12740  -----------------------------------------------MLFNFSSSHASPR
yente0001X_1434  ------------------------------------------------------------
yrohd0001_31880  -------------------------------------------MKKTLISSATVYFLLFT
yrohd0001_15520  ------------------------------------------------------------
yfred0001_15260  ------------------------------------------------------------
yinte0001_16590  -----------------------------------------MFMKKFSDLSILTKLLSGF
ymoll0001_41560  -------------------------------------MAMKKFSDLSILTKLLSGFSVVI
yberc0001_16020  ---------------------------------------MKKFAELSILTKLLSGFSVVI
yberc0001_5770   ---------------------------------------------------------MFA
ymoll0001_5080   -----------------------------------------MFKKMKISTGLSIVIATFM
yaldo0001_37020  ------------------------------------------------------------
ypseu0001X_2733  ---------------------------------------------------MRVNKPVSR
ypest0001X_1885  ---------------------------------------------------MRVNKPVSR
yrohd0001_12010  ----------------------------VPMTLSNYCIYLLSGNSPFTEVIMRVNKPVSR
yfred0001_12940  ---------------------------------------------------MRVNKPVSR
yinte0001_14220  -----------------------------------------------------VNKPVSR
ymoll0001_14640  -----------------------------------------------------VNKPVSR
yberc0001_15000  -----------------------------------------------------VNKPVSR
ykris0001_42780  -----------------------------------------------------VNKPVSR
yente0001X_2568  ---------------------------------------------------MRVNKPVSR
yberc0001_5850   ----------------------------------------MFVLLTAIIIKVIRVVILLY
ymoll0001_5160   -----------------------------------------LLSAISINKMVKFHLYIIV
yberc0001_6170   ---------------------------------------MNNIPLLKVVKIIMSLLLIMM
yaldo0001_6750   ---------------------------------------------------MALLFLVIF
yfred0001_39550  ------------------------------------------------------------
yrohd0001_27170  -----------------------------------------MLKKITIKNGLIAQLSLMS
ykris0001_32930  -----------------------------------------MLKKITIKNGLIAQLSLMS
yente0001X_3909  -----------------------------------------MLKKITIKNGLIAQLSLMS
ymoll0001_40600  -----------------------------------------MLKKITIKNGLIAQLCLMS
yberc0001_29090  -----------------------------------------MLKKITIKSGLIAQLCLMS
ypseu0001X_1449  -----------------------------------------MLDSIRSRILAACIIIVAG
ypest0001X_1447  -----------------------------------------MLDSIRSRILAACIIIVAG
yfred0001_36240  -----------------------------------------MLDSIRSRILAACIIIVAS
yinte0001_10140  -----------------------------------------MLDSIRSRILAACIIIVAG
ykris0001_10260  -----------------------------------------MLDSIRSRILAACIIIVAG
yente0001X_2906  -----------------------------------------MLDSIRSRILAACVIIVAG
yfred0001_36230  ----------------------------------------------------------LQ
ymoll0001_9480   -----------------------------------------MLDSIRSRILAACIIIVAG
yberc0001_9790   -----------------------------------------MLDSIRSRILAACIIIVAG
yruck0001_28810  ----------------------------------VSDMTMIKTTQARFTLLVSIFFILLL
yrohd0001_31660  ---------------------------------------MLKTTQARFTLLVSVFFIFLL
yaldo0001_35590  ---------------------------------------MLKTTQARFTLLVSAFFILLL
ykris0001_37060  ------------------------------------------------------------
yinte0001_37560  ---------------------------------------MLKTTQARFTLLVSAFFILLL
yfred0001_32450  ---------------------------------------MLKTTQARFTLLVSAFFILLL
yente0001X_300   ---------------------------------------MLKTTQARFTLLVSAFFIFLL
ymoll0001_35290  ------------------------------------------------------------
yberc0001_34000  ---------------------------------------MLKTTQARFTLLVSVFFILLL
ykris0001_22290  ----------------------------------------MSLSNWRIGYRLGGGFAFLV
yinte0001_3170   ----------------------------------------MRFIRNIKIRTALILILIAF
yruck0001_31650  -----------------------------------------MFERLKISQGLMGVLALFC
ymoll0001_7110   ----------------------------------------MNLADIKLSHRFMALLAIMV
yruck0001_28370  --------------------------------MAAISNIFGSFENVNVGKKLGLSFFIML
ypseu0001X_4346  --------------------------------MAVFSKIIGSFDNVKVGKKLGLSFFLML
ypest0001X_4370  --------------------------------MAVFSKIIGSFDNVKVGKKLGLSFFLML
yrohd0001_31240  --------------------------------MAAFSKVFGNFENVKVGKKLGLSFFLML
yaldo0001_35130  --------------------------------MAAFSKVFSHFENAKVGKKLGLSFFLML
yinte0001_36970  --------------------------------MAIFSNVFGRFENVRVGKKLGLSFFLML
ykris0001_39870  --------------------------------MAGFSKIFGNFENVKVGKKLGLSFFLML
ymoll0001_34760  --------------------------------MAGFSKILGHFDNVKVGKKLGLSFFLML
yfred0001_31920  --------------------------------MAVFSKVFGNFENVKVGKKLGLSFFMML
yberc0001_33520  --------------------------------MAALSKVFGNFENVKVGKKLGLSFFLML
yente0001X_760   --------------------------------MAAFSKVFGHFENVKVGKKLGLSFFLML
yruck0001_18270  ----------------------------------------MQFLKNITIRAALLWVFGAF
yrohd0001_33070  ----------------------------------------MRFFKNITIRAALLWVLGAF
yfred0001_34740  -------------------------------------------------------VLGAF
yaldo0001_22600  ----------------------------------------MQFLKNISIRVALLWVLGIF
yinte0001_40830  ----------------------------------------MQFLKNITIRVALLWVLGAF
ymoll0001_38830  ----------------------------------------MQFLKNITIRAALLWVLGAF
yberc0001_21820  ----------------------------------------MQFLKNITIRAALLWVLGAF
ykris0001_22260  ----------------------------------------MQFLRDITIRAALLWVLGAF
yente0001X_1297  ----------------------------------------MQFLRNISIRAALLWVLGAF
yruck0001_11920  ---------------------------------------------MKVVTSLLLVLVLFG
ypseu0001X_2707  ---------------------------------------------MKVVTSLLLVLVLFG
ypest0001X_1916  ---------------------------------------------MKVVTSLLLVLVLFG
yrohd0001_33620  -----------------------------------------MFKRMKVVTSLLLVLVLFG
yaldo0001_15230  ---------------------------------------------MKVVTSLLLVLVLFG
ykris0001_37850  ---------------------------------------------MKVVTSLLLVLVLFG
yente0001X_1721  -----------------------------------------MFKRMKVVTSLLLVLVLFG
ymoll0001_37090  ---------------------------------------------MKVVTSLLLVLVLFG
yberc0001_15260  ---------------------------------------------MKVVTSLLLVLVLFG
yinte0001_40500  ---------------------------------------------MRVVTSLLLVLVLFG
yfred0001_14080  ---------------------------------------------MKVVTSLLLVLVLFG
yruck0001_11930  -----------------------------------------MFGRIRISTSFFLLLIMIC
yaldo0001_6760   ------------------------------------------------------------
ypest0001X_1918  ------------------------------------------------------------
ypest0001X_1917  -----------------------------------------MFGQIRISTSFFLLLMFLC
ypseu0001X_2706  -----------------------------------------MFGQIRISTSFFLLLMFLC
yrohd0001_33630  -----------------------------------------MFGRIRISTSFFLLLILIC
yaldo0001_15240  -----------------------------------------MFGRIRISTSFFLLLMFIC
ymoll0001_14920  ------------------------------------------------------------
yberc0001_15270  ------------------------------------------------------------
yfred0001_14090  ------------------------------------------------------------
yinte0001_15900  -----------------------------------------MFGRIRISTSFFLLLILIC
ykris0001_37840  -----------------------------------------MFGRIRISTSFFLLLMLIC
yente0001X_1722  -----------------------------------------MFGRIRISTSFFLLLMLIC
yruck0001_10900  ----------------------------------------MFINNIRLVTLFVAILTGIL
yaldo0001_6770   ------------------------------------------------------------
yaldo0001_15110  -----------------MKSGSTHQPLDIKIAPEPNNTKVLFVNNLRLVTLFIAILAGIL
yinte0001_40380  ----------------MKRGSTHQPLDIKIAPEQNKDTKVLFINNMRLVTLFIAILAGIL
ymoll0001_13500  ---------------------------------------------MRLVTLFIAILAGIL
yberc0001_14860  ----------------MKRGSTHQPLDRKIMPEQSTNTKVLFVNNMRLVTLFIAILAGIL
yrohd0001_33510  -------------------------LDIKITPEHNKDAKVLFVNNVRLVTLFIAILAGIL
yfred0001_13960  ----------------MKRGSTHKSSDIKITPEHNKDTKVLFVNNMRLVTLFIAILAGIL
yente0001X_1708  -------------------------LDIKITPEHHKDTKVFFVNNMRLVTLFIAILAGIL
ykris0001_38040  -------------------------LDIKITPEHNKETKVLFVNNMRLVTLFIAILAGIL
yrohd0001_6540   WFCAILIINPDNNILMWKLSAINTLKPQGNKLKIKYKGIDVNFLKNITIRMALLSILVIF
yente0001X_6250  ------------------------------------------------------------
yruck0001_10760  ------------------------------------------------------------
ypest0001X_1900  ---------------------------MKIAPEPNKDTKVSFINNMRLVTLFIVILAGIL
ypseu0001X_2718  ----------------MKRGSAHKPLDMKIAPEPNKDTKVSFINNMRLVTLFIVILAGIL
yaldo0001_37010  ------------------------------------------------------------
ypest0001X_1899  ------------------------------------------------------------
yfred0001_6650   --------------------------------------------------MALLSILVIF
yinte0001_6950   ----------------------------------------VNFLKHITIRMALLSILVIF
ymoll0001_37360  ----------------------------------------VNFLKHITIRMALLSILVIF
yberc0001_8160   --------------------------------------------------MALLSILVIF
                                                                             


                        190       200       210       220       230       240
                 =========+=========+=========+=========+=========+=========+
ykris0001_41300  ------------------------------------------------------------
yrohd0001_20110  ------------------------------------------------------------
yaldo0001_2440   NVYWGAPAYTSKEGWHVSVAVCDKEGGLAGFALKINGLVSYNKPVEQRDINLWLDKNGDL
ypseu0001X_4220  NIYWGAPTYTPQGGWHVSVAVCDKVGTLAGFALKLNDLIAYNHPVEQRDINLLLDKNGEL
ypest0001X_4540  NIYWGAPTYTPQGGWHVSVAVCDKVGTLAGFALKLNDLIAYNHPVEQRDINLLLDKNGEL
yinte0001_2660   LISIAAMAFFSY------------------------------------------------
yberc0001_2350   NIFWGMPTYSAQNGWHVSVAACDQKGSLAGFSLKLNELFTDNQPIGQRDINIWLDKNGEL
ymoll0001_1840   NIFWGRPTYSAQNGWHVSVAAGDQKGSLAGFSLKLNKLLTDNQPIGQRDINLWLDKNGEL
ykris0001_2490   NIFWGTPTYTKKSGWHVSVAACDQEGSLAGFSLKLNELVNNNQSIEQRDINLLLDKNGEL
yinte0001_2650   SLL---------------------------------------------------------
yrohd0001_2860   NIFWGMPTYAPQSGWHVSVATCDQAGSLTGFSLKLNELVDNNQPIEQRDINLWLDKQGDL
yfred0001_38320  NIFWGMPTYTPQSGWHISVAACDQTGALAGFSLKLNELVTYNQPIEQRDINLWLDQYGEL
yinte0001_26450  ------------------------------------------------------------
yberc0001_7120   ------------------------------------------------------------
ypseu0001X_3409  FSLLQIFSIGYL--------------------------------------SHILKSTKAN
ypest0001X_1004  FSLLQIFSIGYL--------------------------------------SHILKSTKAN
yente0001X_9160  -----------------------------------------------------LDNTKDN
ykris0001_6100   ------------------------------------------------------------
yfred0001_43450  FSLLQLFSISFL--------------------------------------SHILDSTKVN
ymoll0001_7580   FSTLQLFSIGYL---------------------------------------SYISGNTKV
yinte0001_8280   FSLLQLFSIGYL---------------------------------------SHILDSTKV
ykris0001_6110   ------------------------------------------------------------
yrohd0001_35950  SEMAAIDNAVPM---------------------------------------IMFKPDGTV
ypseu0001X_2873  SIDNAVPM-------------------------------------------IIFKPDGTV
ypest0001X_2787  SIDNAVPM-------------------------------------------IIFKPDGTV
ymoll0001_11800  AELTSIDNAVPM---------------------------------------IIFKPDGTV
yfred0001_8890   AELTSIDNAVPM---------------------------------------IIFKPDGTV
ykris0001_12740  AELTSIDSAVPM---------------------------------------IIFKPDGTV
yente0001X_1434  --------------------------------------------------MIIFKPDGTV
yrohd0001_31880  SYALQAEQLSYS------------------------------------------------
yrohd0001_15520  --------------------------------------------------MMLLIGAVAL
yfred0001_15260  --------------------------------------------------MMLLLGAVAL
yinte0001_16590  SVVIVM---------------------------------------------MLLLGAVAL
ymoll0001_41560  VM-------------------------------------------------MLLLGAVAL
yberc0001_16020  VM-------------------------------------------------MLLLGAVAL
yberc0001_5770   LSLSVIAVFSLF---------------------------------------NSIESKDNF
ymoll0001_5080   LFLAVITTFSVL---------------------------------------HAISNKENF
yaldo0001_37020  ------------------------------------------------------------
ypseu0001X_2733  QEYPIERDITLQ---------------------------------------STTDIHGNI
ypest0001X_1885  QEYPIERDITLQ---------------------------------------STTDIHGNI
yrohd0001_12010  QEYPIGSDITLQ---------------------------------------STTDIHGNI
yfred0001_12940  QEYPIGSDTTLQ---------------------------------------STTDIHGNI
yinte0001_14220  QEYPIDRDTTLQ---------------------------------------STTDIHGNI
ymoll0001_14640  QEYPIDRDTTLQ---------------------------------------STTDTHGNI
yberc0001_15000  QEYPIDRDTTLQ---------------------------------------STTDTHGNI
ykris0001_42780  QEYPIDRDTTLQ---------------------------------------STTDIQGNI
yente0001X_2568  QEYPIGRDTTLQ---------------------------------------STTDIQGNI
yberc0001_5850   IVSVKKMMRFYLGMIVL----------------------------------FFIFLAGLS
ymoll0001_5160   ALFIVLAGLSLK---------------------------------------TSSTSQQNF
yberc0001_6170   VLFAVHDIYSSQ------------------------------------------QGYQQT
yaldo0001_6750   LLLWGGVSIFTL---------------------------------------FSLNQLTHS
yfred0001_39550  ------------------------------------------------------------
yrohd0001_27170  LILLIVSVIGFN---------------------------------------SIQESSRSL
ykris0001_32930  LILLIVSIIGVN---------------------------------------SIRESSRAL
yente0001X_3909  LILLIVSVIGIN---------------------------------------SIQESSRAL
ymoll0001_40600  LILLIVSVIGIN---------------------------------------SIQESSRSL
yberc0001_29090  LILLVVSVIGIN---------------------------------------SIQESSRSL
ypseu0001X_1449  SLAINTFLNYSV-------------ANKYNNSAIDNTLTALTVSHSVSIAEWVASKTQMI
ypest0001X_1447  SLAINTFLNYSV-------------ANKYNNSAIDNTLTALTVSHSVSIAEWVASKTQMI
yfred0001_36240  SLAINTYFNYSV------------------------------------------------
yinte0001_10140  SLAINTYFNYSV-------------ANKYNNSAIDNTLTAVTASHGVGIADWVASKTQMI
ykris0001_10260  SLAINTYFNYSV-------------ANKYNNSAIDNTLKAVTASHGVGIADWVAMKTQMI
yente0001X_2906  SLAINTYFNYSV-------------ANKYNSSAIDNTLKAVTASHGVGIADWVAMKTQMI
yfred0001_36230  AVKAGKPVVTPP---------------------------------------YVDAGTNQL
ymoll0001_9480   SLAINTYFNYSV-------------ANKYNSSAIDNTLTAVTASHGVGIADWVATKTQMI
yberc0001_9790   SLAINTYFNYSV-------------ANKYNSSAIDNTLTAVTASHGVGIAEWVATKTQMI
yruck0001_28810  IITFMVIQSFITPQLKRSESTIIGNSVDQIATAISTQMNKVEAQSRSMTQAVSLMDSSAI
yrohd0001_31660  IITLVVIQFFVTPQLKQSESTIIGNNVDQIATAITAQMNKVEAQARSITQAVAIMDSNTI
yaldo0001_35590  IITVVVIQLFVTPQLKQSESTIVGNSVDQIATAITAQMNKVEAQARSITQAVAVMDSNTI
ykris0001_37060  ---VVVIQLFVTPQLKQSESTIVGNSVDQIATAITAQMNKVEAQARSITQAVAIMDSNTI
yinte0001_37560  IITVVVIQLFVTPQLKQSESTIVGNSVDQIATAITAQMNKVEAQARSITQAVAIMDSNTI
yfred0001_32450  IITVVVIQLFVTPQLKQSESTIIGNSVDQIATAITAQMNKVEAQARSITQAVAIMDSNAI
yente0001X_300   IITVVVIQLFVTPQLKQSESTIVSNSVDQIATAITAQMNKVEAQARSITQAVAIMDSNTI
ymoll0001_35290  ---VVVIQLFVTPQLKQSESTIVGNSVDQIATAITAQMNKVEAQARSITQAVAIMDSNTI
yberc0001_34000  IITLVVIQLFITPQLKQSESTIVGNSVDQIATAITAQMNKVEAQARSITQAVAIMDSSTI
ykris0001_22290  LMLFIVSLLAIS-----------------------------------KLSGFQQSASDIV
yinte0001_3170   SLLWAGASGFAL---------------------------------------YSLKQLNQE
yruck0001_31650  IIQIFSGSWSIL---------------------------------------DASGTNDRL
ymoll0001_7110   LGFAAYGAWSFK----------------------------------------VLNNLKVN
yruck0001_28370  LLVSIIAGTGAYHFSAIEDRAYKVDLSYQINEEVNQAKYNRALYERSYNPQFMTTNAKHI
ypseu0001X_4346  LLVGVIAGTTAYHFSVIEEHAYKVELSYKINNEANQAKYNRALYERTYDLKYITENTQHI
ypest0001X_4370  LLVGVIAGTTAYHFSVIEEHAYKVELSYKINDEANQAKYNRALYERTYDLKYITENTQHI
yrohd0001_31240  LLVGIIAGTTAYHFSAIEKHAYKVDLSYKINDEANQAKYNRALYERTYDLKYIKENSEHI
yaldo0001_35130  LLVGIIAGTTAYHFSVIEEHAYKVNLSYKINDEANQAKYNRALYERTYDLKYIKENSGYI
yinte0001_36970  LLVGIIAGTAAYHFSVIEEHAYKVDLSYKINDEANQAKYNRALYERTYDLKYIKENSQHI
ykris0001_39870  LLVGIIAGTTAYHFSVIEEHAYKVDLSYKINDEANQAKYNRALYERTYDLKYIEENSEHI
ymoll0001_34760  LLVGIIAGTTAYHFSVIEEHAYKVDLSYKINDEANQAKYNRALYERTYDLQYIKENSQHI
yfred0001_31920  LLVGIIAGTTAYHFSVIEEHAYKVDLSYKINDEANQAKYNRALYERTYDLKYIKENSQHI
yberc0001_33520  LLVGIIAGTTAYHFSVIEEHAYKVDLSYKINDEANQAKYNRALYERTYDLKYIQENSQHI
yente0001X_760   LLVGIIAGTTAYHFSVIEEHAYKVDLSYKINDEANQAKYNRALYERTYDLQYIKENSQHI
yruck0001_18270  SLLWGGVSAYTL---------------------------------------FSLHELTDA
yrohd0001_33070  CVLWGGVSANTL---------------------------------------LSLNQLTES
yfred0001_34740  CLLWGSVSTYTL---------------------------------------LSLNQLTQS
yaldo0001_22600  CLLWGGVSAYTL---------------------------------------LSLNQLTQS
yinte0001_40830  CLLWGGVSAYTL---------------------------------------LSLNQLTQS
ymoll0001_38830  CLLWGGVSAYTL---------------------------------------LSLNQLTQS
yberc0001_21820  CLLWGGVSAYTL---------------------------------------LSLNQLTQS
ykris0001_22260  CLLWGGVSGYTL---------------------------------------LSLNQLTQS
yente0001X_1297  CLLWGGVSGYTL---------------------------------------LSLNQLTQS
yruck0001_11920  ALQLVSGGLFFN---------------------------------------SLKNDKENF
ypseu0001X_2707  ALQLVSGGLFFK---------------------------------------SLQNDQENF
ypest0001X_1916  ALQLVSGGLFFK---------------------------------------SLQNDQENF
yrohd0001_33620  ALQLVSGGLFFN---------------------------------------SLKSDKENF
yaldo0001_15230  ALQLVSGGLFFN---------------------------------------SLKNDKENF
ykris0001_37850  ALQLVSGGLFFN---------------------------------------SLKNDKENF
yente0001X_1721  ALQLVSGGLFFN---------------------------------------SLKNDKENF
ymoll0001_37090  ALQLVSSGLFFN---------------------------------------SLKNDKENF
yberc0001_15260  ALQLVSSGLFFN---------------------------------------SLKNDKENF
yinte0001_40500  ALQLVSGGLFFN---------------------------------------SLKNDKENF
yfred0001_14080  ALQLVSGGLFFN---------------------------------------SLKNDKENF
yruck0001_11930  SIQLISSGLSFT---------------------------------------AFRADYQNL
yaldo0001_6760   ------------------------------------------------------------
ypest0001X_1918  ------------------------------------------------------------
ypest0001X_1917  AIQLISSGLSFT---------------------------------------AFRSDYQNL
ypseu0001X_2706  AIQLISSGLSFT---------------------------------------AFRSDYQNL
yrohd0001_33630  TIQLISSGLSFT---------------------------------------AFRSDYQNL
yaldo0001_15240  SIQLISSGLSFT---------------------------------------AFRSDYQNL
ymoll0001_14920  ------------------------------------------------------------
yberc0001_15270  ------------------------------------------------------------
yfred0001_14090  ------------------------------------------------------------
yinte0001_15900  SIQLISSGLSFT---------------------------------------AFRSDYQNL
ykris0001_37840  SIQLISSGLSFT---------------------------------------AFRSDYQNL
yente0001X_1722  SIQLISSGLSFT---------------------------------------AFRSDYQNL
yruck0001_10900  ILFAVAIGTSSY---------------------------------------FLKQSNQSL
yaldo0001_6770   ------------------------------------------------------------
yaldo0001_15110  LLFAAAIGTSGY---------------------------------------FLKQSNQSL
yinte0001_40380  LLFAAAIGTSGY---------------------------------------FLKQSNQSL
ymoll0001_13500  LLFAAAIGTSGY---------------------------------------FLKQSNQSL
yberc0001_14860  LLFAAAIGTSGY---------------------------------------FLKQSNQSL
yrohd0001_33510  LLFAAAIGTSGY---------------------------------------FLKQSNQSL
yfred0001_13960  LLFAAAIGTSGY---------------------------------------FLKQSNQSL
yente0001X_1708  LLFAAAIGTSGY---------------------------------------FLKQSNQSL
ykris0001_38040  LLFAAAIGTSGY---------------------------------------FLKQSNQSL
yrohd0001_6540   LLLWGGVSAFTL---------------------------------------FSLNQLTDS
yente0001X_6250  ------------------------------------------------------------
yruck0001_10760  ------------------------------------------------------------
ypest0001X_1900  LLFAAAIGTSGY---------------------------------------FLKQSNQSL
ypseu0001X_2718  LLFAAAIGTSGY---------------------------------------FLKQSNQSL
yaldo0001_37010  -------------------------------------------------LQSTTDTHGNI
ypest0001X_1899  ------------------------------------------------------------
yfred0001_6650   LLLWGGVSAFTL---------------------------------------FSLNQLTHS
yinte0001_6950   LLLWGGVSTFTL---------------------------------------FSLNQLTSS
ymoll0001_37360  LLLWGGVSTFTL---------------------------------------FSLNQLTHS
yberc0001_8160   LLLWGGVSTFTL---------------------------------------FSLNQLMHS
                                                                             


                        250       260       270       280       290       300
                 =========+=========+=========+=========+=========+=========+
ykris0001_41300  ---------------------------------------MLFSDSI--------------
yrohd0001_20110  ------------------------------------------------------------
yaldo0001_2440   LPMAQQSISSHQLHEILTQLKNTVLHDGWQQTSDYLVLRKQLKGPGWQQLVIYPRIGFAW
ypseu0001X_4220  LPISQQATSSNQLHEILNQLKNSKLHDGWQQTPDYLVLRTQLKGPGWQQLVIYPRIGFAW
ypest0001X_4540  LPISQQATSSNQLHEILNQLKNSKLHDGWQQTPDYLVLRTQLKGPGWQQLVIYPRMGFAW
yinte0001_2660   ------------------------------------------------------------
yberc0001_2350   LPISQQNIPLSQLHDIVRQLKNIQLHDGWQQTSDYLVLRTQLKGPGWQQLVIYPRVGFAW
ymoll0001_1840   LPISQQNIPLSQLHEILRQLKEIHLHDGWQQTSDYWVLRTQLKGPGWQQLVIYPSVGFAW
ykris0001_2490   LPLYQQNIPSNQLYEILRQLKDSPLHDGWQQTPDYLVLRTQLKGPGWQQLVIYPRIGFAW
yinte0001_2650   ------------------------------------------------------------
yrohd0001_2860   LPFSKNNIPSNQLHEILSQLKQIQLHDGWQQTPDYLVLRTQLTGPGWQQLVIYPRIGFAW
yfred0001_38320  LSMSTQNIPSSQLYEILKQLKNSKLHDGWQQTSDYLVFRTQLKGPGWQQLVIYPRIGFAW
yinte0001_26450  ------------------------------------------------------------
yberc0001_7120   ------------------------------------------------------------
ypseu0001X_3409  VEITHRNHQQEAL------------MDRARME--LLIASDKLNRAG-IYYMEDKETGSEG
ypest0001X_1004  VEITHRNHQQEAL------------MDRARME--LLIASDKLNRAG-IYYMEDKETGSEG
yente0001X_9160  VEKTHHSHQQKVL------------MDRSRME--LLIASDKLNRAG-IYYMEDKETGSEG
ykris0001_6100   ------------------------------------------------------------
yfred0001_43450  VEKTHQSHQQEVL------------MDRARME--LLIASDKLNRAG-IYYMEDKETGSEG
ymoll0001_7580   NVERANYNHQQEL-----------LMDRARME--LLIASDKLNRAG-IYYMVDKETGSEG
yinte0001_8280   NVEKTHYSHQQEV-----------LMDRARME--LLIASDKLNRAG-IYYMEDKETGSEG
ykris0001_6110   --------------------------------MELLIASDKLNRAG--------------
yrohd0001_35950  VQANKLFLQAMGY----------------ESEEVTGKHHSLFCSPQYVATEAYRKHWQRL
ypseu0001X_2873  IQVNKLFLAAMGY----------------QEGEVIGKHHKIFCDPQYAASDAYRRHWQLL
ypest0001X_2787  VQVNKLFLAAMGY----------------QKDEVIGKHHKIFCDPQYAASDAYRRHWQLL
ymoll0001_11800  KHANKLFLQAMGY----------------QQDEVIGKHHKLFCDPQYVLSDAYRRHWKLL
yfred0001_8890   KHANSLFLQAMGY----------------QQDEVVGKHHKLFCEPHYVLSDAYRRHWQLL
ykris0001_12740  KHANVLFLQAMGY--------------QWEEV--LGKHHKLFCDPHYVQSDAYRRHWQLL
yente0001X_1434  KYANTLFLQAMGY----------------QREEVVGQHHKLFCDPKYVQSDAYRRHWQL-
yrohd0001_31880  ------------------------------------------------------------
yrohd0001_15520  SQLSNNNSRLEAY------------RDSWLPGVRYTLEMRGFLAELRLQQVQYIASTTEK
yfred0001_15260  SQLSSNNSRLEAY------------RDSWLPGVRYALEMRGVLAELRLQQVQYIASSTEK
yinte0001_16590  SQLSNNNSRLEAY------------RDSWLPGVRYALEMRGVLAELRLQQVQYIASSTEK
ymoll0001_41560  SQLSSNNARLEAY------------RDSWLPGVRYALEMRGVLAELRLQQVQYIASSTEK
yberc0001_16020  SQLSSNNARLEAY------------RDSWLPGVRYALEMRGVLAELRLQQVQYIASSTEK
yberc0001_5770   NRTIVSTSNMEGM----------------QSS--IFNLYSGLAQVN-GLLLQSSLNRTID
ymoll0001_5080   NRALNEARNTSVMGDAIFN------LNSGLAHVNALMLQASLNRPV------------KP
yaldo0001_37020  ------------------------------------------------------------
ypseu0001X_2733  AYANAAFVRASGF--------------EYQEL--QGQPHNMVRHPD-MPPAAFADMWQTL
ypest0001X_1885  AYANAAFVRASGF--------------EYQEL--QGQPHNMVRHPD-MPPAAFADMWQTL
yrohd0001_12010  AYANAAFVRASGF--------------EYQDL--QGQPHNMVRHPD-MPAAAFADMWQTL
yfred0001_12940  AYANAAFVRASGF--------------EYQDL--LGQPHNVVRHPE-MPPAAFADMWQTL
yinte0001_14220  AYANAAFVRASGF--------------EYQDL--LSQPHNVVRHPD-MPPAAFADMWQTL
ymoll0001_14640  AYANAAFVRASGF--------------EYQDL--LGQPHNIVRHPD-MPPAAFADMWQTL
yberc0001_15000  AYANAAFVRASGF--------------EYQDL--LGQPHNIVRHPD-MPPAAFADMWQTL
ykris0001_42780  TYANAAFVRASGF--------------EYQNL--LGQPHNVVRHPD-MPPAAFADMWRTL
yente0001X_2568  TYANAAFVRASGF--------------EYQNL--LGQPHNMVRHPD-MPPAAFADMWQTL
yberc0001_5850   LKMSSDARDNFSDTVSLHTRVNLVQQAQYKLRTVRGELASLTMAAMSNQPIDPARVNAIR
ymoll0001_5160   EDTIHLHGRVELV---------------------DQAMYKLMVVRGNILLIKIAALENQP
yberc0001_6170   LFAEETKSQRDDI-------------DRLHYN--LASLRAIFNNQVIGSLTNVPLDKEEI
yaldo0001_6750   LQVSSNQQKSVSI------------INKGNDQ--YFRVATRLIRAA-----IYRQNGAKA
yfred0001_39550  ------------------------------------------------------------
yrohd0001_27170  HIINQIQGEELGS-----------LSNSFNAT-----LSARTEAALAIHQLEIGLIDESL
ykris0001_32930  QVINQIQGEELGS-----------LSNSFNAT-----LSARTEAALAIHQLEIGLIDESL
yente0001X_3909  QAINQIQGEELGS-----------LSNSFTAT-----LSARTEAALAIHQLEIGLIDESL
ymoll0001_40600  QIINQIQGEELGS-----------LSNSFTAT-----LSARTEAALAIHQLEIGLIDESL
yberc0001_29090  QIINQIQGEELGS-----------LSNSFTAT-----LSARTEAALAIHQLEIGLIDESL
ypseu0001X_1449  MSLKDSALTADPLAALKQVAAAGGFINVYIG---YADKTAIFSNPD-GIPADYDPTGRPW
ypest0001X_1447  MSLKDSALTADPLAALKQVAAAGGFINVYIG---YADKTAIFSNPD-GIPADYDPTGRPW
yfred0001_36240  ------------------------------------------------------------
yinte0001_10140  VSLKDSALAADPIAALRQVAAAGNFINVYIG---YANKTAIFSNPD-GIPAGYDPTGRPW
ykris0001_10260  VSLKESSLTADPIAALRQVAAAGNFINVYIG---YANKTAIFSNPD-GIPAGYDPTGRPW
yente0001X_2906  VSLKESALAADPIAALRQVAAAGNFINVYIG---YANKTAVFSNPD-GIPAGYDPTGRPW
yfred0001_36230  VVTFALPIIEDGT-----------------------------------------------
ymoll0001_9480   VSLKESALTADPTAALRQVAAAGNFINVYIG---YANKTATFSNPD-GIPAGYDPTGRPW
yberc0001_9790   VSLKDSALTADPTAALRQVAAAGNFINVYIG---YANKTATFSNPD-GIPAGYDPTGRPW
yruck0001_28810  DSLLPGLVDQYGD-SNVFGGGIWPLPNKRQEG--TIKFSTFYARDA-SNKLAVNTHWNSP
yrohd0001_31660  DTLLPGLVDQYGD-SNVFGGGIWPLPNKREQG--VIKFSTFFARNG-ENKLTVNTHWNSP
yaldo0001_35590  DQLLPGLVDQYGD-SNVFGGGIWPLPNKREQG--VMKFSTFFARNG-ENKLTVNTHWNSP
ykris0001_37060  DQLLPGLVDQYGD-SNVFGGGIWPLPHKREQG--VIKFSTFFARNG-ENKLTVNTHWNSP
yinte0001_37560  DQLLPGLVDQYGD-SNVFGGGIWPLPNKREQG--VIKFSTFFARNG-ENKLTVNTHWNSP
yfred0001_32450  DQLLPGLVDQYGD-SNVFGGGIWPLPNKREQG--VIKFSTFFARNG-ENKLTVNTHWNSP
yente0001X_300   DSLLPGLVDQYGD-SNVFGGGIWPLPNKREQG--VIKFSTFFARNG-ENKLTVNTHWNSP
ymoll0001_35290  DSLLPGLVDQYGD-SNVFGGGIWPLPNKREQG--VAKFSTFFARNG-ENKLTVNTHWNSP
yberc0001_34000  DSLLPGLVDQYGD-SNVFGGGIWPLPNKREQG--VAKFSTFFARNG-ENKLTVNTHWNSP
ykris0001_22290  EEVYPQTVDANAL-----------------------------IDAVNSALVAYQRLLLVT
yinte0001_3170   LGVTHTQQQNGDI------------INGANAQ--YYRAITAMERAMGGLGKNDTGVFDLE
yruck0001_31650  TQISLGFNQIMAM------------DNAYASV--TEIREEVLKDAL-MLATQ----PQDA
ymoll0001_7110   GPVYQNIIQDKNL------------IADILPPPQYIIE---------SYLISLQLASASA
yruck0001_28370  NNILTLLAQNESMNWDAENQKNINRIIEVIKA--YQLDQKDFLATV-EKKDNVRKSWNIS
ypseu0001X_4346  NNIKNLLTQSANLRWSVDNRKNIANINHVVEE--YQQQQTNFVNAV-TNKDKVRQSWSIS
ypest0001X_4370  NNIKNLLTQSANLRWSVDNRKNIANINHVVEE--YQQQQTNFVNAV-TNKDKVRQSWSIS
yrohd0001_31240  NEIKSLLTESEKLSWSAENHKIINSISDIAEE--YLQQQSNFVNAV-AHKDDVRKSWNIS
yaldo0001_35130  NKIKTLLIQSEELDWSENNRKTINSIADVVEE--YLQQQSNFVNAV-AHKDDVRKSWNIS
yinte0001_36970  NNIKNLLTQSESLSWSENNRKTISSISDVVEE--YQQQRSNFVNAV-AHKDDVRKSWNIS
ykris0001_39870  NKIKSLLDQSETLGWSADNRKTISSISDVVEE--YLQQQSNFVNAV-AHKDDVRKSWNIS
ymoll0001_34760  NNIKNLLTQSETLSWSESNRKTIGAIADVVEE--YLQQQSNFVNAV-AHKDDVRKSWNIS
yfred0001_31920  NNIKNLLTQSEGLSWSAENRKIIGGITDVVEE--YLQQQSNFVNAV-AHKDDVRKSWNIS
yberc0001_33520  NNIKNLLTQSEGLSWSEDNRKTIGSIADVVDE--YLQQQSNFVNAV-AHKDDVRKSWNIS
yente0001X_760   NNIKNLLTQSESLSWSENNRKTISSIADVVDE--YLQQQSNFVNAV-AHKDDVRKSWNIS
yruck0001_18270  SNANSVLVENINL------------VNQGSDQ--YFRTVTRLGRSV-----DHRLAGNIA
yrohd0001_33070  SNANAVLVENMNL------------VNQGTDQ--YFRMVTRLARAV-----DYHQSGNMA
yfred0001_34740  SNANAVLVENMNL------------VNQGTDQ--YFRMVTRLARSV-----DYRQNGNIA
yaldo0001_22600  SNANSVLVENMNL------------VNQGTDQ--YFRMVTRLARSV-----DYRQSGNIA
yinte0001_40830  SNANSVLVENMNL------------VNQGTDQ--YFRMVTRLSRSV-----DYRQNGNIA
ymoll0001_38830  SNANSVLVDNMTL------------VNQGTDQ--YFRMVTRLARAV-----DYRQSGNIA
yberc0001_21820  SNANTVLVENMNL------------VNQGTDQ--YFRMVTRLARAV-----DYHQSGNLA
ykris0001_22260  SNANSVLVENMNL------------VNQGTDQ--YFRMVTRLARSV-----DYRQSGNIV
yente0001X_1297  SNANSVLVENMNL------------VNQGTDQ--YFRMVTRLARSV-----DYRQSGNIA
yruck0001_11920  AVLQVIRQQEASL------------GDSWVN---LVQTRNTLNRAGIRYMADANGTGSGT
ypseu0001X_2707  NVLQVIRQQQSVL------------NESWVY---LLQTRNTLNRAGVRYMMDINHTGSGP
ypest0001X_1916  NVLQVIRQQQSVL------------NESWVY---LLQTRNTLNRAGVRYMMDINHTGSGP
yrohd0001_33620  AVLQVIRQQQSVL------------NESWVN---LLQTRNTLNRAGIRYMMDVSHTGSGP
yaldo0001_15230  AVLQVIRQQQSVL------------NESWVS---LLQTRNTLNRAGIRYMMDVNHTGSGP
ykris0001_37850  AVLQVIRQQQSVL------------NESWVN---LLQTRNTLNRAGIRYMMDVNHTGSGP
yente0001X_1721  AVLQVIRQQQSVL------------NESWVN---LLQTRNTLNRAGIRYMMDVNHTGSGP
ymoll0001_37090  AVLQVIRQQQSVL------------NESWVN---LLQTRNTLNRAGIRYMMDVNHTGSGP
yberc0001_15260  AVLQVIRQQQSVL------------NESWVN---LLQTRNTLNRAGIRYMMDVNHTGSGP
yinte0001_40500  AVLQVIRQQQSVL------------NESWVN---LLQTRNTLNRAGIRYMMDVNHTGSGP
yfred0001_14080  AVLQVIRQQQSVL------------NESWVN---LLQTRNTLNRAGIRYMMDVNHTGSGP
yruck0001_11930  NRVELSSQQRDAL------------SLSWVS---LLQARNTLNRAGTRSALNVPQ----E
yaldo0001_6760   ------------------------------------------------------------
ypest0001X_1918  ------------------------------------------------------------
ypest0001X_1917  NRVELSSQQRDAL------------SLSWVS---LLQARNTLNRAATRSALNVPQ----E
ypseu0001X_2706  NRVELSSQQRDAL------------SLSWVS---LLQARNTLNRAATRSALNVPQ----E
yrohd0001_33630  NRVDLSSQQRDAL------------SLSWVS---LLQARNTLNRAGTRAALKVPQ----E
yaldo0001_15240  NRVDLSSQQRDAL------------SLSWVS---LLQARNTLNRAGTRSALKVPQ----E
ymoll0001_14920  --VDLSSQQRDAL------------SLSWVS---LLQARNTLNRAGTRSALKVPQ----E
yberc0001_15270  --VDLSSQQRDAL------------SLSWVS---LLQARNTLNRAGTRSALKVPQ----E
yfred0001_14090  --VDLSSQQRDAL------------SLSWVS---LLQARNTLNRAGTRSALKVPQ----E
yinte0001_15900  NRVDLSSQQRDAL------------SLSWVS---LLQARNTLNRAGTRSALKVPQ----E
ykris0001_37840  NRVDLSSQQRDAL------------SLSWVS---LLQARNTLNRAATRSALKVPQ----E
yente0001X_1722  NRVDLSSQQRDAL------------SLSWVS---LLQARNTLNRAATRSALKVPQ----E
yruck0001_10900  ESATQELDIRLGL----------------SNSSDYLRA----ARLTLIQAASAARIGDTE
yaldo0001_6770   ------------------------------------------------------------
yaldo0001_15110  AQATQELDIRLGL------------SNSSNHL--------RTARLILIQAASSARIGDTT
yinte0001_40380  EEATQELDIRLGL------------SNSSNHL--------RTARLILIQAASAARIGDTE
ymoll0001_13500  EEATQELDIRLGL----------------SNSSNYLRTARLIL----IQAASSARIGDTE
yberc0001_14860  EEATQELDIRLGL----------------SNSSNYLRTARLIL----IQAASSARIGDTE
yrohd0001_33510  EEATQELDIRLGL------------SNSSDHL--------RTARLILIQAASSARIGDTQ
yfred0001_13960  EEATQELDIRLGL------------SNSSNHL--------RTARLILIQAASSARIGDTQ
yente0001X_1708  EEATQELDIRLGL------------SNSSNHL--------RTARLILIQAASSARIGDAT
ykris0001_38040  EEATQELDIRLGL------------SNSSNHL--RTARLILIQAAS------SARIGDAT
yrohd0001_6540   LQQSSHQQKNIHI------------LNQGNDQ--YFRAVTRLSRAA-----AYRQAGAIT
yente0001X_6250  -------------------------------------VVTRLTRAA-----AYRQNGAIA
yruck0001_10760  ------------------------------------------------------------
ypest0001X_1900  AQATQEIDIRQGL------------SNSSNHL--------RTARLILIQAASSARIGDAT
ypseu0001X_2718  AQATQEIDIRQGL------------SNSSNHL--------RTARLILIQAASSARIGDAT
yaldo0001_37010  TYANAAFVRASGF--------------DYQDL----------------------------
ypest0001X_1899  ------------------------------------------------------------
yfred0001_6650   LQQSSSQQKNINI------------INRGNDQ--YFRVVTRLSRAE-----AYRKNGAVA
yinte0001_6950   LHLSSNQQKNINI------------INRGNDQ--YFRVVTRLSRAA-----AYRQSGATA
ymoll0001_37360  LQLSSNQQKNINI------------INRGNDQ--YFRVVTRLSRAA-----AYRQSGATA
yberc0001_8160   LQLSSNQQKNINI------------INRGNDQ--YFRVVTRLSRAA-----AYRQSGATA
                                                                             


                        310       320       330       340       350       360
                 =========+=========+=========+=========+=========+=========+
ykris0001_41300  --------------VEDAQAL---------------------------------SLEDEL
yrohd0001_20110  --------------MSDRFCR---------------------------------------
yaldo0001_2440   EAAKPALYQ-----LPLALAILLLLTLVMSLLLRYYLAIPLWNFVNIIGATGPQTMEPRL
ypseu0001X_4220  EALKPALYQ-----LPFALVILLLLTSVLSLLLRYYLAIPLWNFINIIGATGPQAMEPRL
ypest0001X_4540  EALKPALYQ-----LPFALAILLLLTSVLSLLLRYYLAIPLWNFINIIGATGPQAMEPRL
yinte0001_2660   -----------------------------------------------------EDTRQRL
yberc0001_2350   EAAKPALHQ-----LPFAFAILLLLTLVLSLLLRYYLAIPLWNFVNIMAATGPKTMEPRL
ymoll0001_1840   EAAKPALHQ-----LPFAFAILLLLTLVLSLLLRYYLAIPLWNFVNIIGATGPQAMEPRL
ykris0001_2490   EAAKPSLHQ-----LPFALAILLLLTLALSLLLRYYLAIPLWNFVNIIGATGPHAMEPRL
yinte0001_2650   --------------LRYYLAI------------------PLWNFINIIGATGPQAMEPRL
yrohd0001_2860   EAAKPALHQ-----IPFAFAILLLLTLILFLLLRYYLAIPLWNFVNIIGATGPQAMEPRL
yfred0001_38320  EAAKPALHQ-----LPFALAILLLLTLILFLLLRYYLAIPLWNFVNIIGATSPQAMEPRL
yinte0001_26450  ------------------------------------------------------------
yberc0001_7120   ------------------------------------------------------------
ypseu0001X_3409  SWQSL---------LNEALQS-------------------------------LQQSQDSY
ypest0001X_1004  SWQSL---------LNEALQS-------------------------------LQQSQDSY
yente0001X_9160  SWQSL---------LSEATKS-------------------------------IQQSQDDF
ykris0001_6100   ------------------------------------------------------------
yfred0001_43450  SWHSL---------LSEAL----------------------------------QSLLRSQ
ymoll0001_7580   SWNSL---------LSEALQS----------------------------------MQQSQ
yinte0001_8280   SWHSL---------LNEALQS----------------------------------MQQSQ
ykris0001_6110   ------------------------------------------------------------
yrohd0001_35950  NAGRA---------ITDNIKR------------------------------VRKDGSIIW
ypseu0001X_2873  NEGRP---------ISDNIKR------------------------------IRKDGSVIW
ypest0001X_2787  NEGRP---------ISDNIKR---------------------------------------
ymoll0001_11800  NEGRP---------ITDNIKR------------------------------IRKDGSTIW
yfred0001_8890   NEGRP---------ITDNIKR------------------------------IRKDGSIIW
ykris0001_12740  NEGRP---------ITDNIKR------------------------------IRKDGSIIW
yente0001X_1434  --------------LNEGRAI----------------------------------TDNIK
yrohd0001_31880  ------------------------------------------------------------
yrohd0001_15520  DRESHRLEI-----LQAVDNF--------------------------------RNAQDLF
yfred0001_15260  EREGHRLEI-----LQAVDNF--------------------------------RAAQNLF
yinte0001_16590  EREGHRLEI-----LQAVDNF--------------------------------RAAQDRF
ymoll0001_41560  EREGHRLEI-----LQAVDNF--------------------------------RAAQNRF
yberc0001_16020  EREGHRLEI-----LQAVDNF--------------------------------RAAQNRF
yberc0001_5770   PQS-----------IANAKDI----------------L---------------ANSKATM
ymoll0001_5080   ES------------IDNAKNI-------------------------------LAQAKSDM
yaldo0001_37020  ------------------------------------------------------------
ypseu0001X_2733  NAGLS---------WSSLVKN------------------------------RRKNGDYYW
ypest0001X_1885  NAGLS---------WSSLVKN------------------------------RRKNGDYYW
yrohd0001_12010  NDGLS---------WSSLVKN------------------------------RRKNGDYYW
yfred0001_12940  HDGLS---------WSSLVKN------------------------------RRKNGDYYW
yinte0001_14220  NAGLS---------WSSLVKN------------------------------RRKNGDYYW
ymoll0001_14640  NAGLS---------WSSLVKN------------------------------RRQNGDYYW
yberc0001_15000  NAGLS---------WSSLVKN------------------------------RRQNGDYYW
ykris0001_42780  NAGLS---------WSSLVKN------------------------------RRLNGDYYW
yente0001X_2568  NAGLS---------WSSLVKN------------------------------RRQNGDYYW
yberc0001_5850   NHNKE---------VRSIVEQ--------------------------------WIREPKT
ymoll0001_5160   TDPE----------LVSSINI-----------------------------------KNKV
yberc0001_6170   KHFIE-----------------------------------------------LTTSSREL
yaldo0001_6750   DADRE---------LTSAGAA-------------------------------LKNTQEDL
yfred0001_39550  ------------------------------------------------------------
yrohd0001_27170  QTAKQ---------AEAMLAL----------------------------------SQTEM
ykris0001_32930  ETAKQ---------AEESLAL----------------------------------SQKEM
yente0001X_3909  QTAKQ---------AEESLAL----------------------------------SQREM
ymoll0001_40600  QTAKQ---------AEGSLAL----------------------------------SQKEM
yberc0001_29090  QTAKQ---------AEGSLAL----------------------------------SQKEM
ypseu0001X_1449  YLQA----------VNAGKPV--------------------------VTPPYIDAGTQQL
ypest0001X_1447  YLQA----------VNAGKPV--------------------------VTPPYIDAGTQQL
yfred0001_36240  ------------------------------------------------------------
yinte0001_10140  YLQA----------VKAGKPM--------------------------VTPPYIDAGTNQL
ykris0001_10260  YLQA----------VKAGSPV--------------------------VTPPYIDAGTNQL
yente0001X_2906  YLQA----------VKAGSPV--------------------------VTPPYIDAGTNQL
yfred0001_36230  --------------VKGVLAA---------------------------------------
ymoll0001_9480   YLQA----------VKAGKPV--------------------------VTPPYVDAGTNQL
yberc0001_9790   YLQA----------VKADKPV--------------------------VTPPYVDAGTNQL
yruck0001_28810  ESQNYFEQPWYKEGLKSAKGF------------------CAWAKAYQDDASPQPRTNCAM
yrohd0001_31660  ESQNYFEQPWYKDGLQAPKGF------------------CAWAKAYQDDASPQPRTNCAM
yaldo0001_35590  ESQNYFEQPWYKEGLNAPKGF------------------CAWAKAYQDDASPQPRTNCAM
ykris0001_37060  ESQNYFEQPWYKEGLNAPKGF------------------CAWAKAYQDDASPQPRTNCAM
yinte0001_37560  ESQNYFEQPWYKDGLNAPAGF------------------CAWAKAYQDDASPQPRTNCAM
yfred0001_32450  EAQNYFEQPWYKDGLNAPKGF------------------CAWAKAYQDDASPQPRTNCAM
yente0001X_300   ESQNYFEQPWYKDGLNAPKGF------------------CAWAKAYQDDASPQPRTNCAM
ymoll0001_35290  ESQNYFEQSWYKDGLNAPKGF------------------CAWAKAYQDDASPQPRTNCAM
yberc0001_34000  ESQNYFEQSWYKDGLNAPKGF------------------CAWAKAYQDDASPQPRTNCAM
ykris0001_22290  DEAQE---------KKTIDMI------------------------------ADLRKDIST
yinte0001_3170   --------------MKATLAE-------------------------------LDSLKNGL
yruck0001_31650  KARQS---------LGELRQN----------------L---------------TEVDQQM
ymoll0001_7110   DERNE---------LISNLKY-------------------------------KKKEFENH
yruck0001_28370  ETQKY---------LNQLAQN----------------FLNEGADTNSRILLAEMNQKLMA
ypseu0001X_4346  ESLNN---------INALQQS----------------LLNEGADTNTQILLAEMSQKLMT
ypest0001X_4370  ESLNN---------INALQQS----------------LLNEGADTNTQILLAEMSQKLMT
yrohd0001_31240  ETQKN---------LNELQQN----------------FLNEGADTNTQILLAEMNQKLMT
yaldo0001_35130  ETQKN---------LNELQQN----------------LLNEGADTHTQILLAEMNQKLMT
yinte0001_36970  ETQKN---------LNELQQS----------------LLNEGADTNTQILLAEMNQKLMT
ykris0001_39870  ETQKN---------LNELQQS----------------LLNEGADTNTQILLAEMNQKLMT
ymoll0001_34760  ETQKN---------LNELQQS----------------LLNEGADTNTQILLAEMNQKLMT
yfred0001_31920  ETQKN---------LNELQQS----------------LLNEGADTNTQILLAEMNQKLMT
yberc0001_33520  ETQKN---------LNELQQS----------------LLNEGADTNTQILLAEMNQKLMT
yente0001X_760   ETQKN---------LNELQQS----------------LLNEGADTNTQILLAEMNQKLMT
yruck0001_18270  EADRE---------LKSSNKA-------------------------------LENLKNSL
yrohd0001_33070  DADKE---------LKSSNVA-------------------------------LENLKEKL
yfred0001_34740  DAEKE---------LNSSNVA-------------------------------LDNLKKKL
yaldo0001_22600  DADKE---------LKSSNVA-------------------------------LENLKKKL
yinte0001_40830  DADKE---------LKSSDVA-------------------------------LENLKQKL
ymoll0001_38830  DADKE---------LKSSNIA-------------------------------LENLKKRL
yberc0001_21820  DADKE---------LKSSSAA-------------------------------LENLKKRL
ykris0001_22260  DADKE---------LKSSNTA-------------------------------LENLKKEL
yente0001X_1297  DADKE---------LKSSNAA-------------------------------LENLKQKL
yruck0001_11920  TLPEL---------VTLAKET-------------------------------LVKAQKSY
ypseu0001X_2707  TVKDL---------ISSAKGT-------------------------------LSVAEDRF
ypest0001X_1916  TVKDL---------ISSAKGT-------------------------------LSVAEDRF
yrohd0001_33620  TVNDL---------LASAKGT-------------------------------LGVAAERF
yaldo0001_15230  TVNDL---------LASAKGT-------------------------------LGVAAERF
ykris0001_37850  TVNDL---------LASAKGT-------------------------------LGVAAERF
yente0001X_1721  TVNDL---------LASAKGT-------------------------------LGVAAERF
ymoll0001_37090  TVNDL---------LASAKGT-------------------------------LGVAADRF
yberc0001_15260  TVNDL---------LASAKGT-------------------------------LGVAADRF
yinte0001_40500  TVNDL---------LASAKGT-------------------------------LGVAAERF
yfred0001_14080  TVNDL---------LASAKGT-------------------------------LGVAEERF
yruck0001_11930  KVNEL---------MGNARSS-------------------------------LQKADLYF
yaldo0001_6760   ------------------------------------------------------------
ypest0001X_1918  ------------------------------------------------------------
ypest0001X_1917  QVNAL---------MGSARSA-------------------------------LQKAELYF
ypseu0001X_2706  QVNAL---------MGSARSA-------------------------------LQKAELYF
yrohd0001_33630  QVNAL---------MGNARSS-------------------------------LQKADLYF
yaldo0001_15240  QVNAL---------MGNARSS-------------------------------LQKADLYF
ymoll0001_14920  QVNAL---------MGNARSS-------------------------------LQKADLYF
yberc0001_15270  QVNAL---------MGNARSS-------------------------------LQKADLYF
yfred0001_14090  QVNAL---------MGNARSS-------------------------------LQKADLYF
yinte0001_15900  QVNAL---------MGNARSS-------------------------------LQKADLYF
ykris0001_37840  QVNAL---------MGNARSS-------------------------------LQKADLYF
yente0001X_1722  KVNEL---------MGNARSS-------------------------------LQKADLYF
yruck0001_10900  SYQTS---------LKEAENR-------------------------------LSESQKMF
yaldo0001_6770   ------------------------------------------------------------
yaldo0001_15110  GYQQG---------LKNAAER-------------------------------IAQSQQMF
yinte0001_40380  GYQQG---------LKNAESR-------------------------------ITQSQQMF
ymoll0001_13500  GYQQG---------LKSAEGW-------------------------------IAQSQQMF
yberc0001_14860  GYQQG---------LKSAEGW-------------------------------IAQSQQMF
yrohd0001_33510  GYQQG---------LKNAESR-------------------------------IAQSQQMF
yfred0001_13960  GYQQG---------LKNAESL-------------------------------LSQSQQMF
yente0001X_1708  GYQQG---------LKNAENR-------------------------------ISQSQQMF
ykris0001_38040  GYQQG---------LKNAENR---------------------------------ISQSQQ
yrohd0001_6540   DADRE---------LKSAGAA-------------------------------LKITKDAL
yente0001X_6250  DADRE---------LASASVA-------------------------------LKNTQEAL
yruck0001_10760  ------------------------------------------------------------
ypest0001X_1900  GYQQG---------LKNAEGR-------------------------------IAQSQQMF
ypseu0001X_2718  GYQQG---------LKNAEGR-------------------------------IAQSQQMF
yaldo0001_37010  --------------LSKPHNM---------------------------------------
ypest0001X_1899  ------------------------------------------------------------
yfred0001_6650   DADRE---------LASAGVA-------------------------------LKNTQEAL
yinte0001_6950   NADRE---------LASAGVA-------------------------------LKNTQDNL
ymoll0001_37360  DADRE---------LASAGVA-------------------------------LKNTQEAL
yberc0001_8160   EADRE---------LASAGVA-------------------------------LKNTQDAL
                                                                             


                        370       380       390       400       410       420
                 =========+=========+=========+=========+=========+=========+
ykris0001_41300  FLLRARNLYTAENI---------------------------------------------G
yrohd0001_20110  --------------------------------------------------LSS------F
yaldo0001_2440   PIKRMDELGHIACAYNNL--LDTLNEQYDTLEMKVKERTLALAKSKQVAEQAN------Q
ypseu0001X_4220  PINRIDELGHIARAYNNL--LDTLNEQYDTLEMKVKERTLALAKAKRAAEQAN------R
ypest0001X_4540  PINRIDELGHIARAYNNL--LDTLNEQYDTLEMKVKERTLALAEAKRAAEQAN------R
yinte0001_2660   VN----ELTHMASLRADL-----SNYQFEGAERDAASLI--------------------S
yberc0001_2350   PVKRMDELGQIACAYNHL--LDALNEQYDTLEMKVKKRTLALAKSKQAAEQAN------R
ymoll0001_1840   PVKRMDELGMIAMAYNKL--LDALNEQYDTLEMKVEKRTLALAKSKQAAEQAH------R
ykris0001_2490   PIKRMDELGHIARAYNNL--LDKLNEQYDTLEMKVKERTLALAKSKLAAEQAN------H
yinte0001_2650   PVKRMDELGQIARAYNNL--LDTLNEQYDTLEMKVKERTLALAKSKQVAEQAN------R
yrohd0001_2860   PIKRMDELGQIARAYNNL--LDALNEQYDTLEMKVKERTLALAKSKQAAEQAN------H
yfred0001_38320  PIKRMDELGNIACAYNKL--LDKLNEQYDTLEIKVKERTLALAKSKQAAEQAN------H
yinte0001_26450  ------------------------------------------------------------
yberc0001_7120   ------------------------------------------------------------
ypseu0001X_3409  LQLQAVSAQDSRQE------LNELKESYHQLYQGLAEIAQGLAQK--------------H
ypest0001X_1004  LQLQAVSAQDSRQE------LNELKESYHQLYQGLAEIAQGLAQK--------------H
yente0001X_9160  RQLLLVSAHDNRPE------FIALKESYLQLFQGLSEISQGLVKN--------------S
ykris0001_6100   ------------------------------------------------------------
yfred0001_43450  DDFHQLERLSAQDKRPEL---VALKESYQQLYQGLTEVGQGLEKN--------------N
ymoll0001_7580   ENYSQLSRLSAHDDRPE---FIALKESYQQLYQGLAEIAQGLSKN--------------N
yinte0001_8280   QNYRELLLLSAHDNRPE---FVALKESYQQLYQGLTELGQGLSKN--------------N
ykris0001_6110   ------------------------------------------------------------
yrohd0001_35950  LQGTYTPVFDKYGKVIEI----------IKIASDVTERILQS-----------------Q
ypseu0001X_2873  LQGTYTPVVDRQGNVIEI----------IKIASDVTERILQS-----------------Q
ypest0001X_2787  -IRKDGNVIWLQGTYTPVVDRQGNVIEIIKIASDVTERILQS-----------------Q
ymoll0001_11800  LQGTYTPVLDNQGRVMEI----------IKIAHDVTERILQS-----------------Q
yfred0001_8890   LQGTYTPVLDSQGRVVEI----------IKLASDVTERILQS-----------------Q
ykris0001_12740  LQGTYTPVLDNQGRVVEI----------IKIASDVTERIVQS-----------------Q
yente0001X_1434  RIRKDGSIIWLQGTYTPVLDSQGRVVEIIKLASDVTERMLQS-----------------Q
yrohd0001_31880  --YKDGPVVAHDNLSGNQ------------------------------------------
yrohd0001_15520  LK------LPEGYNNSEL--FKRIMSDFDHFSQ-ANSIVINAVNN--------------N
yfred0001_15260  LK------LPEGYNNSEL--FKKIISDFDHFAQ-ANNIVIDAVNN--------------N
yinte0001_16590  IK------LPEGYNHSML--FKRITSDFDHFSQ-ANSLVIDAVNN--------------N
ymoll0001_41560  LK------LPEGYNDSVL--FKRIMSDFDNFSQ-ANSIVIDAVNS--------------N
yberc0001_16020  LK------LPEGYNDSEL--FKRIMSDFDNFSQ-ANSFVIDAVNN--------------N
yberc0001_5770   DSFM--STGFNSTEVEQA--AIVLNKQFLRVLNTTLDKADYISNP---------------
ymoll0001_5080   KIFMSTPFNSPKEKQVAV--------ELNTHFERVLSLSLGKVNF--------------I
yaldo0001_37020  ------------------------------------------------------------
ypseu0001X_2733  VRANATPLRHNGRLTGYI--SVRIAPTRDEVKQ-AEALYTDFNTG--------------K
ypest0001X_1885  VRANATPLRHNGRLTGYI--SVRIAPTRDEVKQ-AEALYTDFNTG--------------K
yrohd0001_12010  VRANATPLRHQGRLTGYI--SVRIAPTREEVKQ-AETLYADFNSG--------------K
yfred0001_12940  VRANATPLRHDGRLTGYI--SVRIAPTREEIKQ-AEALYADFNSG--------------K
yinte0001_14220  VRANATPLRHDGRLTGYI--SVRIAPTREEVKQ-AEALYADFNSG--------------K
ymoll0001_14640  VRANATPLRHNGRLTGYI--SVRIAPTREEIKL-AETLYADFNSG--------------K
yberc0001_15000  VRANATPLRHNGRLTGYI--SVRIAPTREEVKQ-AEALYADFNSG--------------K
ykris0001_42780  VRANATPLLHKNRLTGYI--SVRIAPTREEVKQ-AEALYADFNSG--------------K
yente0001X_2568  VRANATPLRHDGRLTGYI--SVRIAPTREEVKQ-AEALYGDFNSG--------------K
yberc0001_5850   STYNQELSERAGAVFVKL--LDNYMRSLDNL-----------------------------
ymoll0001_5160   IRETITKWVKEPKSTEQI---QELSERTGAMFIKILDSYMNNLDA--------------R
yberc0001_6170   LASYIEAMENDRRYDATTAVLHKNITAILDHMEDAANGFIT------------------G
yaldo0001_6750   ARF---KLQSHVAIDPLL--ADKAIQSWSELLSKGVEPMFKAVKE--------------D
yfred0001_39550  ------------------------------------------------------------
yrohd0001_27170  AHFV--SAVSEQGEGQEL--ADNIQKSYKNYVDKGVIPMLAAIKA--------------G
ykris0001_32930  AHFI--SAVSERGDGQTL--ATNIQKSYKDYVDKGVTPMLVAIKA--------------G
yente0001X_3909  ARFV--SAVSERGEGQEL--ATNIQKSYKNYVDKGVIPMLTAIKA--------------G
ymoll0001_40600  ARFV--SAVSDRGDGQEL--AINIQKSYKNYVDKGVLPMLAAIKA--------------G
yberc0001_29090  ARFV--SAVSERGDGQEL--ATNIQKSYKNYVDKGVLPMLAAIKA--------------G
ypseu0001X_1449  VVTFAWSIVQDGVLKGVI--AADVTMGSVIANVNAIHPTDNSFGMLIGADGTII-----A
ypest0001X_1447  VVTFAWSIVQDGVLKGVI--AADVTMGSVIANVNAIHPTDNSFGMLIGADGTII-----A
yfred0001_36240  ------------------------------------------------------------
yinte0001_10140  VVTFALPIIQDGSVKGVL--AADVTMDSVIANVKSIHPTEDSFGM-----LIDADGTIIA
ykris0001_10260  VVTFALPIIQDGSVKGVL--AADVTMDSVITNVKSIHPTEGSFGM-----LIDTDGTIIA
yente0001X_2906  VVTFALPIIQDGSVKGVL--AADVTMDSVIANVKSIHPTDDSFGMLIDAD-----GTIIA
yfred0001_36230  ----------DVTMDSVIANVKSIHPADDSFGM-----LIDADGTII------------A
ymoll0001_9480   VVTFALPIIQDGSVKGVL--AADVTMDSVIANVKSIHPTDDSFGM-----LIDADGTIIA
yberc0001_9790   VVTFALPIIQDGSVKGVL--AADVTMDSVIANVKSIHPTDDSFGMLIDAD-----GTIIA
yruck0001_28810  AIYKGNEVYGVSTIDVTLGFFNQLVSQMEQ-KIHGSILIVESDGKIVGSSLSSEGSSGLQ
yrohd0001_31660  AIYKGDDIYGVSTIDVTLGFFNRLVKEMEQ-KVNGTILIVEADGKIVGSSALADGKAELK
yaldo0001_35590  AIYKGGEVYGVSTIDVTLGFFNRLVKEMEQ-KVNGTILIVEADGKIVGSSALADGKAELK
ykris0001_37060  AIYKGDEIYGVSTIDVTLGFFNRLVKDMEQ-KVNGTILIVEADGKIVGSSALADGKAELK
yinte0001_37560  AIYKGDEAYGVSTIDVTLGFFNRLVKEMEQ-KVNGTILIVETDGKIVGSSALADGKAELK
yfred0001_32450  AIYKGDDVYGVSTIDVTLGFFNRLVKEMEQ-KVNGTILIVEADGKIVGSSALADGKAELK
yente0001X_300   AIYKGDEAYGVSTIDVTLGFFNRLVKEMEQ-KVNGTILIVEADGKIVGSSALADGKAELK
ymoll0001_35290  AIYKGNEAYGVSTIDVTLGFFNRLVKEMEQ-KVNGTILIVEADGKIVGSSALADGKAELK
yberc0001_34000  AIYKGNEAYGVSTIDVTLGFFNRLVKEMEQ-KVNGTILIVEADGKIVGSSALADGKAELK
ykris0001_22290  KLADLEAGVQDSKSKDVLREIQNSRTDFLTSGDKILSLVLA------------------G
yinte0001_3170   SQF---KAIDHGNLDSAT--IDAIYNSSFNLYNSAVLPMFESAKA--------------K
yruck0001_31650  EKFY--QLSQSNQNEIER--TKNVKSLYEKARGDLLQLIISLEN---------------N
ymoll0001_7110   REFWTHSNLSNELKEQLLAATGKPAQEFYQIAFDQFIPALESG----------------D
yruck0001_28370  VRYNARGLLLDRNKEAEA--SMITAIDVAQNAANAFLPVLSTEQQKLMLPVIDSLKAYKG
ypseu0001X_4346  VRYNARGLLLDRNKDAET--ALITSINIANSAANAFMPVLSAEQQKLLAPVAANLALYKD
ypest0001X_4370  VRYNARGLLLDRNKDAET--ALITSINIANSAANAFMPVLSAEQQKLLAPVAANLALYKD
yrohd0001_31240  VRYNARGLLLDRNQEAES--SLITSINVANSAANAFMPVLSASQQQLMAPVISSLATYKD
yaldo0001_35130  VRYNARGLLLDRNQDAES--SLITSINIANSAANAFMPVLSADQQKLLAPVISSLSAYKD
yinte0001_36970  VRYNARGLLLDRNQDAES--SLITSINIANSAANAFMPVLSAEQQKLLAPVISSLSTYKD
ykris0001_39870  VRYNARGLLLDRNQDAES--SLITSINIANSAANAFMPVLSADQQKLLAPVISSLSAYKD
ymoll0001_34760  VRYNARGLLLDRNQEAES--SLITSINIANSAANAFMPVLSPDQQRLLAPVITSLGTYKD
yfred0001_31920  VRYNARGLLLDRSQEAES--SLITSINIANSAANAFMPVLSADQQKLLAPVISSLSAYKD
yberc0001_33520  VRYNARGLLLDRNQEAES--SLITSINIANSAANAFMPVLSPDQQKLLAPVITSLGSYKE
yente0001X_760   VRYNARGLLLDRNQDAES--SLITSINIANSAANAFMPVLSADQQKLLAPVISSLSIYKD
yruck0001_18270  AQF---KAIDHAQLNPVL--VNEVISSWNGLVIQGVDPLYQAAVV--------------N
yrohd0001_33070  AEF---KAIDHAQIDPVL--VTGVIDGWSGLIEQGVMPLYQAAMD--------------N
yfred0001_34740  AEF---KTIDHAQIDPAL--VTGVIDGWSGLIDQGVTPLYQAAMD--------------N
yaldo0001_22600  AEF---KAVDHAQIDPVL--VTGVIDGWSGLIEQGVTPLYQAAMD--------------N
yinte0001_40830  AEF---KAIDHAQLDPAL--VSGVIDGWSGLIEQGVTPLYQAAMD--------------N
ymoll0001_38830  AEF---KAIDHAQIDPAL--VSRVIDGWSGLIEQGVTPLYQAAMD--------------N
yberc0001_21820  AEF---KAIDHAQIDPAL--VAGVIDGWSGLIEQGVMPLYQAAMD--------------N
ykris0001_22260  AQF---KAIDHAQIDPAL--VTGVVNGWSGLIDQGVTPLFQAAMN--------------N
yente0001X_1297  AQF---KAIDHAQIDPTL--VNGVIDGWSGLIDQGVTPLYQAAMA--------------N
yruck0001_11920  ANFE--KISLDSGQSPES--TQRMKQTYDAYVG-ALNELIQLMEA--------------G
ypseu0001X_2707  KRYE--KIPQNSSQDPEG--AKKLKQSYEEYFS-ALTELINLMET--------------N
ypest0001X_1916  KRYE--KIPQNSSQDPEG--AKKLKQSYEEYFS-ALTELINLMET--------------N
yrohd0001_33620  KAYE--QIALDGQQDPES--AKKLKQTYDQYFG-ALAELIQLMEA--------------G
yaldo0001_15230  KSYE--QIPLDSQQNPDS--AQKLKQTYDQYFG-ALTELIQLMEA--------------G
ykris0001_37850  KSYE--QIPLDSKQDPES--AKKLKLTYDQYFA-ALTELIQLMQA--------------G
yente0001X_1721  KSYE--QIPLDSQQDPES--AKKLKQTYEQYFG-ALTELIQLMEA--------------A
ymoll0001_37090  KQYE--QIPLDSQQDAES--ATKLKQTYDQYFG-ALTELIQLMEA--------------G
yberc0001_15260  KNYE--QIPLDSQQDAES--AKKLKQTYDQYFG-ALTELIQLMEA--------------G
yinte0001_40500  KSYE--QIPLDSQQDAES--AKKLKQTYDQYFG-ALTELIQLMEA--------------G
yfred0001_14080  KSYE--QIPLDSRQDPES--AKKLKQTYDQYFG-ALTELIQLMEA--------------G
yruck0001_11930  NQFLAIPRVNDNADEL----IAATQASYQNLRT-SLRELIGFLEA--------------G
yaldo0001_6760   ------------------------------------------------------------
ypest0001X_1918  ------------------------------------------------------------
ypest0001X_1917  NQFQAVPRLDESESSGQL--LDATKNSYQNLRS-ALRELIDFLEA--------------G
ypseu0001X_2706  NQFQAVPRLDESESSGQL--LDATKNSYQNLRS-ALRELIDFLEA--------------G
yrohd0001_33630  NQFLAVPPLEDSTTGSEL--SDATQKSYQNLRT-SLRELIGFLEA--------------G
yaldo0001_15240  NQFLAVPRLDASDTGAEM--LEATQNSYQNLRT-SLRQLIDFLEA--------------G
ymoll0001_14920  NQFLAVPRLDESDTGGEL--LDATKNSYQNLRV-SLRQLIDFLEA--------------G
yberc0001_15270  NQFLAVPRLDESETGGEL--LNATKNSYQNLRL-SLRQLIDFLEA--------------G
yfred0001_14090  NQFLAVPRLDESDTGGEL--LDATKNSYQNLRT-SLRELIDFLEA--------------G
yinte0001_15900  NQFIAVPRLDESDNGSEL--LDATKNSYQNLRT-SLRQLIDFLEA--------------G
ykris0001_37840  NQFLAVPRLDESDTGGEL--LDATKNSYQNLRV-SLRELIDFLEA--------------G
yente0001X_1722  NQFLAVPRLDESDTGGEL--LDATKNSYQNLRS-SLRELIDFLEA--------------G
yruck0001_10900  DVYSSRTAKDATDIALDV----PLKKAYEQYRNEGMRAMLDATQQ--------------G
yaldo0001_6770   ------------------------------------------------------------
yaldo0001_15110  DLYYNRPIKSETDTALDV----PLKKAYEQYRDDGIKPMLASTKE--------------G
yinte0001_40380  DLYYNRPVKSQTDTALDV----PLKKAYEQYRDEGMKIMLDATKE--------------G
ymoll0001_13500  DLYYNRPIKSETDTALDG----PLKKAYEQYRDDGMKLMLDATKE--------------G
yberc0001_14860  NLYYNRPVKSETDTALDG----PLKKAYEQYRDDGMKLMLDATKE--------------G
yrohd0001_33510  DLYYNRPIKSETDTALDA----PLKKAYAEYRNDGMKPMLDATKE--------------G
yfred0001_13960  NLYYNRPIKSETDTALDA----PLKKAYEEYRDDGMKLMLAATKE--------------G
yente0001X_1708  NLYYNRPIKSETDTALDG----PLKKAYEQYRDDGMKLMLEATKE--------------G
ykris0001_38040  MFDLYYNRPTKSETDIAL--DSPLKKAYEQYRDDGMKLMLEATKE--------------G
yrohd0001_6540   EKF---TQQSPNGMDKQL--TDDTIQRWSTLLNKGIDPMFKAVTD--------------N
yente0001X_6250  QKF---KLQSHEEMDTAL--TEKTIQSWSTLLDKGIEPMFKAVSE--------------N
yruck0001_10760  ------------------------------------------------------------
ypest0001X_1900  DLYYNRPTKSETDMALDV----PLKKAYEQYRDDGMKPMLAATKE--------------G
ypseu0001X_2718  DLYYNRPTKSETDMALDV----PLKKAYEQYRDDGMKPMLAATKE--------------G
yaldo0001_37010  ------------------------------------------------------------
ypest0001X_1899  ------------------------------------------------------------
yfred0001_6650   EKF---KRQSHEGMDPSL--TDKTIEVWSTLLSKGIEPMFKAVSD--------------N
yinte0001_6950   AKF---KLQSHESMDPIL--TDKTIQSWSTLLSKGIEPMVKAVTE--------------N
ymoll0001_37360  AKF---KLQTHEPMDPLL--TDKTIQSWSTLLSDGIEPMFKAVTD--------------S
yberc0001_8160   AAF---KLQIHEPMDPAL--TDKTIQSYSTLLSQGIEPMFKAVAE--------------N
                                                                             


                        430       440       450       460       470       480
                 =========+=========+=========+=========+=========+=========+
ykris0001_41300  RWRTQLDDYGGENKDLQDKISAAENAMMRNTTRIESIEGT--------------------
yrohd0001_20110  KYSKLFNQLVRGSSMAAIRDIVKSVDQSTEASAETKETLKILMDMVEA------------
yaldo0001_2440   RKSEHLTTISHEIRTPLNGALGAVELLQNTPLSPEQMQLAETARQCSFSLLAIINNLLDF
ypseu0001X_4220  RKSDHLTTISHEIRTPLNGALGAVELLQNTPLDAGQMRLAETAHQCSLSLLAIINNLLDF
ypest0001X_4540  RKSDHLTTISHEIRTPLNGALGAVELLQNTPLDAGQMRLAETAHQCSLSLLAIINNLLDF
yinte0001_2660   RQTSYQATWRLPIRHTDDDPGNNIPFSSAICNAIQSRHDLRIAQAYGTSG----------
yberc0001_2350   RKRMHLTTISHEIRTPLNGALGAVELLQNTTLTTEQYQLAETARQCSQSLLAIINNLLDF
ymoll0001_1840   RKRVHLTTISHEIRTPLNGALGAVELLQNTALTPEQYRLAETARQCSHSLLAIINNLLDF
ykris0001_2490   RKSIHLTTISHEIRTPLNGTLGAVELLQNTYLTPEQYRLAETASQCSHSLLAIINNLLDF
yinte0001_2650   RKSVHLTTISHEIRTPLNGTIGAIELLQNTPLTSEQYRLAETARQCSHSLLTIVNNLLDF
yrohd0001_2860   RKSEHLTTISHEIRTPLNGALGAIELLQNTPLTSAQQRLAETARQCSYSLLAIINNLLDF
yfred0001_38320  RKSEHLTTISHEIRTPLNGALGAIELLQNTPLTSAQNRLAETARQCSHSLLAIINNLLDF
yinte0001_26450  ------------------------------------------------------------
yberc0001_7120   ------------------------------------------------------------
ypseu0001X_3409  NIDTFFDVPIQGFQ---SDFTEKYYRYLQESEKGSTAMDEQLLSSLSSAKQIIIGAL---
ypest0001X_1004  NIDTFFDVPIQGFQS---DFTEKYYRYLQESEKGSTAMDEQLLSSLSSAKQIIIGAL---
yente0001X_9160  NIDAFFEVPIQGFQ---SDFIEKYYRYLQESEENRTVRDSQLLSSLSSAKQAVIIAL---
ykris0001_6100   --MLFFEVPIQGFQ---SDFTEKYYRYLQESENNRMVMDSQLLSSLSLAKQAVIIAL---
yfred0001_43450  SIDAFFEVPIQGFQ---SDFTEKYYHYLQESEKNRTVMDAQLLSSLSCAKQAVIIAL---
ymoll0001_7580   NIDVFFEVPIQGFQS---DFTEKYYHYLQASENKRTLMDTQLLSSLSSAKQTVIAAL---
yinte0001_8280   NIDLFFEVPIQGFQ---SDFTEKYYHYLQQSEDNRAVMDTQLLSSLSSAKQGVITAL---
ykris0001_6110   ------------------------------------------------------------
yrohd0001_35950  EHQSLLNALNRSMGLITFTPQGIILDANDNLLNLIGYSLADIQNKSH-------------
ypseu0001X_2873  EHQSLLEALNRSMGMITFTPQGIILAANDNLLNVIGYSLADIQHKSH-------------
ypest0001X_2787  EHQSLLEALNRSMGMITFTPQGIILAANDNLLNVIGYSLADIQHKSH-------------
ymoll0001_11800  EHQSLLKALNRSMGMITFSPEGIILEANDNLLSVIGYSLADIQHKSH-------------
yfred0001_8890   EHQSLLAALNRSMGMITFTPQGTILDANDNLLKVIGYSLADIQHKPH-------------
ykris0001_12740  EHQSLLAALNRSMGMITFTPQGIILDANDNLLHVIGYSLADIRHKSH-------------
yente0001X_1434  EHQSLLAALNRSMGMITFTPQGIILDANDNLLNVIGYSLADIRHKSH-------------
yrohd0001_31880  ------------------------------------------------------------
yrohd0001_15520  QLDEASKISGDTSRKYRTQLMKDLAELVVLEVQGGEKAAAEGQQSFNTAKNLLLTLL---
yfred0001_15260  QLAEASKISGDTSRKFRTQLMKDLAELVVLEVQGGEQAASEGQQSFNTAKNILIALL---
yinte0001_16590  QLAEATKISGDTSRKYRTQLMKDLAELVVLEVQGGEQAATDGQQSFNTAKNVLIALL---
ymoll0001_41560  QLAEASKISGDTSRKYRTQLMKDLAELVVLEVQGGEKAATEGQQSFNTAKNLLIALL---
yberc0001_16020  QLAEASKISGDTSRKYRTQLMKDLAELVVLEVQGGEKAATEGQQSFNTAKSLLLALL---
yberc0001_5770   ---AGMPDTLEKEMQERTILRENINAYQNIANHLNDNFIQGADSVYRQMVTMAIVVP---
ymoll0001_5080   ANPASLPDNIDSEMQERAALREQIQNYGQVASELDSGYSQQAEADYQSMITAAIIVV---
yaldo0001_37020  ------------------------------------------------------------
ypseu0001X_2733  AKRRHIALYRGLIVRTGWLSPLSLFQTLPLRWRLRSALLSSAIIPTAAASVMEVAGQ---
ypest0001X_1885  AKRRHIALYRGLIVRTGWLSPLSLFQTLPLRWRLRSALLSSAIIPTAAASVMEVAGQ---
yrohd0001_12010  ARQRRIALYRGLIVRTGCLSLLSIFQTLPLRWRLRGASLVAALLPTVAAGVMGVTGL---
yfred0001_12940  AKQRHIALYRGLIVRTGWRSLFSIFQTLPLRWRLRGALFGAAVLPTVAAGVMGMTAM---
yinte0001_14220  AKQRHIALYRGLIVRTGWLSLLSLFQTLPLRWRLRGALLGAALLPTVAAGVIGITGL---
ymoll0001_14640  AKQRRIGLYRGLIVRTGWLSWMSLFQTLPLRWRLRGALLGASLLPTAAAGVMGITGL---
yberc0001_15000  ARQRHIRLYRGLIVRSGWLSWMSLFQTLSLRWRLRGALLTAALLPTVAAGVMGMTGS---
ykris0001_42780  AKQRRIALYRGLIVRTGVLSFLSLFQTLSLRWRLRGALLTAALLPTLAAMGMGVTGL---
yente0001X_2568  AKQRRIALHRGLIVRTGWLSFRSLFQTLPLRWRLRGALLIAALLPTLAAKGMGIFGL---
yberc0001_5850   HQSAQIINESDALMHQLEGLNQEYLKISADLISSYENKTELLHRALIVSLVIISGLFI--
ymoll0001_5160   QQGGEAINDTDELVPQLEVLVAEYLKFSADSMESFDVKNDYLHSALVVTISIIIALFI--
yberc0001_6170   NINNDL-----SLREAYQQVNDSIAEFSAVNTDIYMSTLHSANTVKKQSYVIAITLL---
yaldo0001_6750   RYEDFQRVFNHDYRSLSREFGSAVEG----DNSAVDKATVRVALLVTWCQQALLAAL---
yfred0001_39550  ------------------------------------------------------------
yrohd0001_27170  YADEYYEVLEKSITDISTAFNNDVAAFRSYALNAGQLKIAEANSAAKIKLAIIVIAG---
ykris0001_32930  HADEYYEVLEKSITAISKAFNNDVVAFRSYAQNAGHLQIDKANSDASIKLTIIVVAG---
yente0001X_3909  YADEYYEVLEKSITDISKAFNNDVATFRSYELNAGHLQIDKANSAAKIKLAIIVAAG---
ymoll0001_40600  YADEYYEVLEKSITDISKAFNNDVATFRSYALDAGQRQIDGANSAAKIKLAIIVAAG---
yberc0001_29090  YADEYYEVLEKSITDISNAFNSDVATFRRYALDAGQLQIDQANSAAKIKLAIIVAAG---
ypseu0001X_1449  HPETLLTLKPLADIAPNLNLQTLLTATVPVSTDISGSSKLLLAQAVPGTQWFTVVALDKS
ypest0001X_1447  HPETLLTLKPLADIAPNLNLQTLLTATVPVSTDISGSSKLLLAQAVPGTQWFTVVALDKS
yfred0001_36240  -----------------------ANKYNNSAIDNTLTAVTASH-----------------
yinte0001_10140  HQDAKLTLKPLSDIAPTLDLKTLLTSTEPVAANIGDNSKLLLAQPVPGTQWFTVVALDKA
ykris0001_10260  HPDAQLTLKPLSEIAPTLDLKTLLTASSPTAAEIDGSTKLLLAQAVPGTQWFTVVALDKA
yente0001X_2906  HPDSQLTLKPLSEIAPTLDLKTLLTAISPTAAEIGGSTKLLLAQAVPGTQWFTVVALDKA
yfred0001_36230  HQDAQLTLKPLSEIAPTLDLKALLSATQPITADIGGSSKLLLAQAVPGTQWFTVVALDKA
ymoll0001_9480   HLDAQLTLKPLSDIAPTLDLKTLLTATDPIVADIGDQSKLLLAQAVPGTQWFTVVALDKA
yberc0001_9790   HPDAQLTLKPLSEIAPTLELKTLLSATDPIVANIGDQSKLLLAQSVPGTQWFTVVALDKT
yruck0001_28810  NLSDISASSPMAAETQRLLANIANQPVAEQEYYRDGQSHTLFIRPIANSPWYLVTDLPTS
yrohd0001_31660  NLSDIASNSPMAAETQRLLAQMKGQQSLESEFDSDGISHTLFIRPIANSPWYLVTDLPTS
yaldo0001_35590  NLSDVAASSPMATETQRLLAQMKDQKSLESEFDVEGTSHTLFIRPIANSPWYLVTDLPTS
ykris0001_37060  NLSDFASSSPMAAETQRLLPQMKDQKLLENEFDVDGTSHTLFIRPIANSPWYLVTDLPTS
yinte0001_37560  NLSDFAANSPMAAETQRLLPQLKEQKALESEFDVDGTSHTLFIRPIANSPWYLVTDLPTS
yfred0001_32450  NLSDFAGSSPMAAETQRLLAQMKDQKSLESEFDVDGTSHTLFIRPIANSPWYLVTDLPTS
yente0001X_300   NLSDFAASSPMAAETQRLLAQMKDQKSLESEFDVDGTSHTLFIRPIANSPWYLVTDLPTS
ymoll0001_35290  NLSDFASSLPMAAETQRLLPQMKDQKSLESEFDVDGTSHTLFIRPIANSPWYLVTDLPTS
yberc0001_34000  NLSDFAASLPMAAETQRLLPQMREQKSLESEFDVDGTSHTLFIRPIANSPWYLVTDLPTS
ykris0001_22290  NREAAVTEFTDNMNITQAQYREHVRQLVAYQDQIMADSVDSMAKVYTSTRNLLLAIL---
yinte0001_3170   NTNGFETIKADKYLPLRRDFSAAIDKYNAKIISLNEEANQRISQWLVWCQYILISGL---
yruck0001_31650  DLVKFQSIMHNRSSTHFMSALDDASAYV--ANSIITPAANAAQVSYRQMLPLSLSFM---
ymoll0001_7110   NLAAAVAL--EAMKKHYGVHRHAIDKLVEQAAKLTETDEADAKTKIQTAMWSMSAIL---
yruck0001_28370  NILAYLPAYEQEV-AAGKRLENKAIELNSLISNLFAQELQGTHDEIDKAQMQMTLTA---
ypseu0001X_4346  NVLAYLPAYQQELE-VGKVMEASANALNTLATQLFTQELQGTHNEINNAQYQLAIAA---
ypest0001X_4370  NVLAYLPAYQQELE-VGKVMEASANALNTLATQLFTQELQGTHNEINNAQYQLAIAA---
yrohd0001_31240  NVLAYLPAYRQELE-VGKVMEASANELNKLATQLFTQELQGTHLEINNAQWLLAIAA---
yaldo0001_35130  NVLAYLPAYQQELA-VGKVMEYNANELNKLATHLFTQELQGTHDEINNAQLQLTITA---
yinte0001_36970  NVLAYLPAYQQELE-VGKVMESNANELSKLATHLFTQELQGTHAEINNAQLQLTIAA---
ykris0001_39870  NVLAYLPAYQQELE-VGKVMEDNANELNKLATHLFTQELQGTHDEIDNAQLQLTITV---
ymoll0001_34760  NVLAYLPAYQQELE-VGKVMETSANELNKLATHLFTQELQGTHDEINNAQLQLAIAA---
yfred0001_31920  NVLAYLPAYQQELE-VGIVMETNANELNKLATHLFTQELQGTHDEINNAQLQLTIAA---
yberc0001_33520  NVLAYLPAYQQELE-VGKVMETNANELNKLATHLFTQELQGTHDEINNAQLQLAIAA---
yente0001X_760   NVLAYLPAYQQELE-VGKVMEENANELNKLATHLFTQELQGTHDEINNAQLQLAIAA---
yruck0001_18270  DNSAYQLLSKQTVPALSRQFGASAESFDKAASEQIGVVKVQFAHLTKVSSITLISAL---
yrohd0001_33070  NSAAYQELAKKTVPALSRQYGSVAEAFNQAASHAIGVAKDQFAQLTKMSSMTLIAAL---
yfred0001_34740  NSVAYQDLAKKTVPALSRQYGAVAETFNQAASKAIGVAKDQFSHLTKVSSITLISAL---
yaldo0001_22600  NSAAYQELAKKTVPALSRQYGSVAESFNQSASKAIGVAKEQFSHLTKVSSVTLISAL---
yinte0001_40830  NSAAYQDLAKKTVPALSRQYGSVAEKFNQAASKAIGVAKDQFSHLTKVSSITLISAL---
ymoll0001_38830  NTAAYQELAKKTVPALSRQYGSVAEGFNQAASKAIGVAKEQFSQLTKVSSITLISAL---
yberc0001_21820  NTAAYQDLAKKTVPALSRQYGAVAEGFNQAASKAIGVAKVQFSQLTKVSSITLISAL---
ykris0001_22260  NDTVYEDLAKKTVPALSRQYGAVAENFNQAASKAIGVAKEQFAHLTRVSSIILISAL---
yente0001X_1297  NSAAYQDLAKKTVPALSRQYGSVAENFNQAASKAIGVAKEQFAHLTKVSSMTLISAL---
yruck0001_11920  KIKEFFDQPTTSFQ---DAFEKDYTDYRTLNDHLYTSVVEDSNQSFTFAIAVLIAIL---
ypseu0001X_2707  KINEFFNHPTTSFQ---NEFEQNYNNYLTQNDGLYDSAVEDSNQSFSFAMGVVITVL---
ypest0001X_1916  KINEFFNHPTTSFQ---NEFEQNYNNYLTQNDGLYDSAVEDSNQSFSFAMGVVITVL---
yrohd0001_33620  KINEFFDQPTSSFQ---NAFEQDYNTYLTQNDRLYSSAVENSNQSFNFALGVIVTVL---
yaldo0001_15230  KINEFFDQPTSSFQ---NAFEKDYNTYLVQNDRLYASAVEDSNQSFSFAMGVIIAVL---
ykris0001_37850  KINEFFDQPTSSFQ---NAFEQDYNTYLTQNDRLYASVVEDSNRSFTYAMSVIIFVL---
yente0001X_1721  KINEFFDQPTSSFQ---NAFEHDYNTYLTQNDRLYSGAVEDSNRSFTYAMSVIVFVL---
ymoll0001_37090  KINEFFDQPTSSFQ---NAFEKDYNTYLTQNDRLYATAVEDSNQSFSFAMGVIVAVL---
yberc0001_15260  KINEFFDQPTSSFQ---NAFEKDYNTYLTQNDRLYASAVEDSNHSFSFAMGVIVAVL---
yinte0001_40500  KINEFFDQPTSSFQ---NAFEQDYNTYLTQNDRLYAAAVEDSNQSFSFAMGVIITVL---
yfred0001_14080  KINEFFDQPTSSFQ---NAFEHDYNTYLTQNDRLYASAVEDSNQSFSFAMGVIVTVL---
yruck0001_11930  NLQAFMDQPTQKTQDL---FEADFVQYLQHTTEIITDSSQENAQAYLLSKWIFAGAV---
yaldo0001_6760   ------------------------------------------------------------
ypest0001X_1918  ------------------------------------------------------------
ypest0001X_1917  DLQAFMDQPTQKTQDL---FEADFIQYLQYANEVIADAGQQNQQAYQLSIWIFSGAI---
ypseu0001X_2706  DLQAFMDQPTQKTQDL---FEADFIQYLQYANEVIADAGQQNQQAYQLSIWIFSGAI---
yrohd0001_33630  NLQAFMDQPTQKTQDL---FEADFVQYLQYANDVIAEAGTHNQQAYHLSMWIFGGAI---
yaldo0001_15240  NLQGFMDQPTQKTQDL---FEADFVQYLQYANDVIAKAGTQNQQAYHLSMWIFGGAI---
ymoll0001_14920  NLQGFMDQPTQKTQDL---FEADFLQYLQYANEVIAEAGTQNQQAYHLSMWIFGGAI---
yberc0001_15270  NLQGFMDQPTQKTQDL---FEADFLQYLQYANEVIAEAGTENQQAYHLSIWIFGGAI---
yfred0001_14090  NLQGFMDQPTQKTQDL---FEADFVQYLQYANEVIAEAGTQNQQAYHLSMWIFGGAI---
yinte0001_15900  NLQGFMDQPTQKTQDL---FEADFLQYLQYANEVIAEAGTQNQQAYHLSMWIFGAAI---
ykris0001_37840  NLQSFMDQPTQKTQDL---FEADFMQYLQYANEVVADAGSQNQQAYHLSMWIFGGAI---
yente0001X_1722  NLQSFMDQPTQKTQDL---FEADFLQYLQYANEVIAEAGTQNQQAYHLAMWIFAGAI---
yruck0001_10900  HFEEVISLEAEKLKRLDTAYNEPLLTALKYRTERANQINQAAQKETRLGYILMGGAF---
yaldo0001_6770   ------------------------------------------------------------
yaldo0001_15110  HFEEVISLEAEKLNPLDEAYDEPLLKAVKYRTEQANLINQTAQQEARLGYILMGSAF---
yinte0001_40380  HFEEVISLETEKLNQLDDAYNEPLLKALKYRTEQANQINQAAQQEARLGYMLMGGAF---
ymoll0001_13500  HFEEVISLEAEKLNQLDSAYNEPLLKAVKYRTERANQINQTAQQEARLGYLLMGGAF---
yberc0001_14860  HFEEVISLEAEKLNQLDSAYNEPLLKAVKYRTERATQINQTAQQEARLGYILMGGAF---
yrohd0001_33510  HFEEVISLEAEKLNQLDGAYNEPLLKALKYRTELANQINLSAKEEARLGYLLMGGAF---
yfred0001_13960  HFEEVISLEAEKLNQLDDAYNEPLLKAVKYRTERANQINLTAQQEARLGYILMGGAF---
yente0001X_1708  HFEEVISLEAEKLNQLDDAYNEPLLKAVKYRTERANEINQSAQQEARLGYMLMGGAF---
ykris0001_38040  HFEEVISLEAEKLNQLDDAYNEPLLKAVKYRTELANQINQSAQQEAHLGYILMGGAF---
yrohd0001_6540   RFEDYSHLFNNVYPSFSREFGAAIDKYNSKANEETEAANIQVHLLVKWCEWALLAAL---
yente0001X_6250  RFEDYNRMFNNEYPQLSREFGATIEKYNSAVDKSTEAASLRVESLVTWCERALLAAL---
yruck0001_10760  ------------------------------------------------------------
ypest0001X_1900  HFEEVISLDAEKISLLDDGYNEPLLKAVKYRTEQANQINQSAHQEARLGYILMAGAF---
ypseu0001X_2718  HFEEVISLDAEKISLLDDGYNEPLLKAVKYRTEQANQINQSAHQEARLGYILMAGAF---
yaldo0001_37010  ------------------------------------------------------------
ypest0001X_1899  ---------------------------------------------VKYATW---------
yfred0001_6650   RIEDYNKLFNNVYPPFSREFGAAVEKYNSTINEATEAASVRVQLLVEWCQRALLAAL---
yinte0001_6950   RFEDYNKLFNNDYPPLSREFGATVEKYNSAVDIATEAASLRVETLVDWCQRALFAAL---
ymoll0001_37360  RFDEYNTLFNKSYPPLSREFGAAVEKYNSTVDEATEAASLRVELLVTWCERALMAAL---
yberc0001_8160   RFDDYNKLFNQGYPPLSREFGAAVEKYNSTVDQATEAASVRVELLVTWCVRALIAAL---
                                                                             


                        490       500       510       520       530       540
                 =========+=========+=========+=========+=========+=========+
ykris0001_41300  -----------------LASI-------------------------RKIHADADYREVAT
yrohd0001_20110  --------------KAELFENQIDNNLITGKTIDNLNVPITKVISRKKEYRAVTSNRSD-
yaldo0001_2440   SRIESGQMTLSQEKTALLPLLDQAMLTIHSLVLNKPIALSTYVSSDVPLELELDSQRLK-
ypseu0001X_4220  SRIESGQMTLSLEKTALLPLLDQAMLTIHSQALSKSLALSTFISADIPLELELDTLRLR-
ypest0001X_4540  SRIESGQMTLSLEKTALLPLLDQAMLTIHSQALSKSLALSTFISANIPLELELDTLRLR-
yinte0001_2660   ----------------------------------QTYYLDSF------------------
yberc0001_2350   SRIESGQMTLSQEKTALLPLLDQAMLAVHSQVLSQSVILSTYVSSDVPLSLELDSQRLK-
ymoll0001_1840   SRIESGQMTLSQEKTALLPLLDQAMLTIHSQALSQSLFLSTYVSSEVPLSVELDSQHLK-
ykris0001_2490   SRIESGQLTLSQDKTALLPLLDQAMLTIHSQVLSKSIVLSTYVSSDVPLELELDSQRIK-
yinte0001_2650   SRIESGQMALSQEKSALLPLLDQAMLTIHSQALSKAITLSTYVSSDVPLALELDKQRLQ-
yrohd0001_2860   SRIESGQLALAQERTALLPLLDQAMLTIHSQVSNKPISLSTYVSAEVPLELELDSQRLK-
yfred0001_38320  SRIESGQLELAQERTAILPLLDQAMLTIHSQVLSKAITLSTYVSAEVPLQLVLDGQRLK-
yinte0001_26450  ------------------------------------------------LRKSLKIIKSQ-
yberc0001_7120   -------------------------------LAVTRWIIRLL----NYLISHINVIAAG-
ypseu0001X_3409  -----------------AILL---VLAFSAWLGVTRWMIAPL----NHLISRINKIAAG-
ypest0001X_1004  -----------------AILL---VLAFSAWLGVTRWMIAPL----NHLISRINKIAAG-
yente0001X_9160  -----------------VILL---CLAFSVWLGVTRWVIRPL----NYLIAQIHVIAAG-
ykris0001_6100   -----------------VILL---CLAFSVWLGVTRWVIRPL----NYIISQIHVIAAG-
yfred0001_43450  -----------------VILL---GLAFSVWLAVTRWVIRPL----NYLISQIHVIAAG-
ymoll0001_7580   -----------------VLLL---CLAFSVWLGVTRWIIKPL----NNLILHINVIAAG-
yinte0001_8280   -----------------VILL---CLAFSVWLGVTRLIIRPL----NDLISHINVIAAG-
ykris0001_6110   ------------------------------------------------------------
yrohd0001_35950  -----------------------------------QILCTPEFSQSDEYRQHWQRLARG-
ypseu0001X_2873  -----------------------------------QILCLPEFAHSEEYHQHWQRLASG-
ypest0001X_2787  -----------------------------------QILCLPEFAHSEEYHQHWQRLARG-
ymoll0001_11800  -----------------------------------QILCTPEFAHSDQYRQHWQRLARG-
yfred0001_8890   -----------------------------------QMLCTAEFAHSDEYRKHWQRLARG-
ykris0001_12740  -----------------------------------QILCPPEFAHSDEYRQHWQRLAQG-
yente0001X_1434  -----------------------------------QILCTPEFAHSDEYRQHWQRLARG-
yrohd0001_31880  ---------------------------------------------------FYGDMTLG-
yrohd0001_15520  -----------------LFSV---FISALLASMIARHLSRSLGGEPAYAVAIMGHIAAG-
yfred0001_15260  -----------------LFAV---FISAVLAIVIARNLSRLLGGEPAYAVTIMGHIAAG-
yinte0001_16590  -----------------LFAV---FISALLAVMIARNLSRLLGGEPAYAVAIMGHIAAG-
ymoll0001_41560  -----------------LFAI---LISALLAIIIARNLSRLLGGEPAYAVAIMGHIAAG-
yberc0001_16020  -----------------LFAV---LISALLASMIARNLSRLLGGEPAYAVAIMGHIAAG-
yberc0001_5770   -----------------IISI---VLLVLVRIWLKRTLTTRM----SQTSASIKKIASG-
ymoll0001_5080   -----------------CCSI---VMMFFARLWLKRTLVTRM----AQTSLSIKKIASG-
yaldo0001_37020  ------------------------------------------------------------
ypseu0001X_2733  -----------PLLILGSVAL---TWSVLASLWLERQIARPI----AAILQQAQDVSSG-
ypest0001X_1885  -----------PLLILGSVAL---TWSVLASLWLERQIARPI----AAILQQAQDVSSG-
yrohd0001_12010  -----------PLLTMGAVTL---ASSIITSFWLERQVARPI----AAILQQAQDVSSG-
yfred0001_12940  -----------PLLVMGGVTL---ASSIITSFWLERQVARPI----AAILQQAQDVSSG-
yinte0001_14220  -----------PLLVLAAVTV---ASSVVASWWLERQVARPI----AAILQQAQDVSSG-
ymoll0001_14640  -----------PLLTLGAVTL---ASSIITSFWLERQVARPI----AAILQQAQDVSSG-
yberc0001_15000  -----------PLVILGAVTL---ASSIITALWLERQVARPI----AAILQQAQDVSSG-
ykris0001_42780  -----------PLLGLGIATL---FSSLITSFWLERQVARPI----AAILQQAQDVSSG-
yente0001X_2568  -----------PLLGLGATTL---FSSLITSLWLERQVSRPI----AAILQQAQDVSSG-
yberc0001_5850   ------------------------LLYLSLFRYITRNVLDRL----AEASTIFSEISKG-
ymoll0001_5160   ------------------------LLYFSLLKYITRFILNRL----AEASQIFSEISMG-
yberc0001_6170   -----------------LSYI---LITVVATTWFNKNIILKI----KLACDIFDKISKG-
yaldo0001_6750   -----------------VAGI---IILFLTDRYWVNFLVRPL----DLIKTHFKRLAEG-
yfred0001_39550  -----------------------------------------L----DQSIHQLEYIADG-
yrohd0001_27170  -----------------VITL---LSAVLAWFALKYIILQPL----EESIHQLEYIADG-
ykris0001_32930  -----------------IITL---TSAVLAWFALKYIILRPL----EQSIHQLEYIAAG-
yente0001X_3909  -----------------IITL---ISAVLAWFALKYIILQPL----EQSIHQLEYIADG-
ymoll0001_40600  -----------------VITL---ISALLAWFALKYIILQPL----EQSIHQLEFIASG-
yberc0001_29090  -----------------VITL---ISAVLAWFALKYIILQPL----EQSIHQLEFIASG-
ypseu0001X_1449  QATAGIHSLLTTSLVTLVIII---FISAGVIALITQRALTPL----THIQQAMDAISSGS
ypest0001X_1447  QATAGIHSLLTTSLVTLVIII---FISAGVIALITQRALTPL----THIQQAMDAISSGS
yfred0001_36240  ------------------------GVGIADWVTSKTQMIVSL------------------
yinte0001_10140  QATTGMRSLLTTSLVTLIIII---LIAVVIISLITKRALAPL----THVHKAMDAISSGS
ykris0001_10260  HATAGMRSLLTTSLVTLIVII---LIAVVIISLITQRALTPL----THVHKAMDAISSGT
yente0001X_2906  HATAGMRSLLTTSLVTLIVII---LIAVVIISLITQRALTPL----THVHKAMDAISSGT
yfred0001_36230  QSTAGMRSLLITSLVTLIVII---LIAAIIIGLITQRALTPL----THVHKAMDAISSGT
ymoll0001_9480   HATAGMRSLLTTSLVTLIVII---FIAVVIIGLITQRALLPL----NHVHKAMDAISSGT
yberc0001_9790   HATAGMRSLLTTSLVTLVVII---LIAVVIISLITQRALLPL----NHVHKAMDAISSGA
yruck0001_28810  LLVKQSNSILFHLGLVQIPIM---LLLFLFLIFSIRIFMRRL----AVLKQNITALSAGG
yrohd0001_31660  LLVKQSHSILLHLGLVQIPIM---LLLLLFLVFSIRVFMKRL----AILKENITALSAGG
yaldo0001_35590  LLVKQSHGILLHLGLVQIPIM---LLLLLFLVFSIRVFMKRL----AALKENITALSAGG
ykris0001_37060  LLVKQSHSILLHLGLVQIPIM---LLLLLFLVFSIRVFMKRL----AALKQNIMALSAGG
yinte0001_37560  LLVKQSHAILLHLGLVQIPIM---LLLLLFLVFSIRVFMKRL----AVLKENITALSAGG
yfred0001_32450  LLVKQSHSILMHLGLVQIPIM---LLLLLFLVFSIRVFMKRL----AVLKENITALSAGG
yente0001X_300   LLVKQSHSILLHLGLVQIPIM---LILLLFLVFSIRVFMKRL----AALKENITALSAGG
ymoll0001_35290  LLVKQSHSILMHLGLVQIPIM---LLLLLFLVFSIRVFMKRL----ANLKENITALSAGG
yberc0001_34000  LLVKQSHSILMHLGLVQIPIM---LLLLIFLVFSIRVFMKRL----ANLKENITALSAGG
ykris0001_22290  -----------------LTSA---VAGLLIAWAITRSVTQPI----QQALDIANKVAQG-
yinte0001_3170   -----------------LISV---LIMLTTDRYLVNFLVKPL----NRVKAHLESLAQG-
yruck0001_31650  -----------------FVFI---VLTGAVLVWIRKYVLNKI----NQVIDYQAAIAHG-
ymoll0001_7110   -----------------IAAT---VLVGLFLLAISRSLIKQLGGEPGYAVDIAGKISAG-
yruck0001_28370  -----------------IIAL---LLGLLIAWRITRQITVPL----RTTLAMAERIATG-
ypseu0001X_4346  -----------------FVAL---LLGLLIAWRMTRQITVPL----RATLDMAERIATG-
ypest0001X_4370  -----------------LVAL---LLGLLIAWRMTRQITVPL----RATLDMAERIATG-
yrohd0001_31240  -----------------LAAL---LCGLLISWRMTRQITLPL----RTTLAMAERIATG-
yaldo0001_35130  -----------------IVAL---LLGLLISWRMTRQITVPL----RTTLAMAERIATG-
yinte0001_36970  -----------------IAAL---LFGLLISWRMTRQITVPL----RTTLAMAERIATG-
ykris0001_39870  -----------------IAAL---LFGLLISWRMTRQITVPL----RTTLAMAERIANG-
ymoll0001_34760  -----------------VAAL---LLGLLISWRMTRQITVPL----RATLAMAERIATG-
yfred0001_31920  -----------------IAAL---LFGLLISWRMTRQITVPL----RTTLAMAERIATG-
yberc0001_33520  -----------------IAAL---LFGLLISWRMTRQITVPL----RTTLAMAERIATG-
yente0001X_760   -----------------IAAL---ILGLLISWRMTRQITVPL----RTTLAMAERIATG-
yruck0001_18270  -----------------IAGL---LILLATDRFLVANLVRPL----HQMRDHFKVIASG-
yrohd0001_33070  -----------------LTGL---AILLATDRYLVVNLVRPL----DDIRAHFRVIASG-
yfred0001_34740  -----------------VAGL---VILLATDRYLVVNLVRPL----DDIRAHFRVIASG-
yaldo0001_22600  -----------------VAGL---LILLATDRYLVANLVRPL----DEIREHFRVIASG-
yinte0001_40830  -----------------VIGL---LILLATDRYLVANLVRPL----DDIRKHFLVIASG-
ymoll0001_38830  -----------------VAGL---VILLATDRYLLANLVRPL----DDIRAHFRVIASG-
yberc0001_21820  -----------------VAGL---VILLATDRYLLANLVRPL----DEIRAHFRVIASG-
ykris0001_22260  -----------------VAGL---VILVVTDRYLIVNMVRPL----DDIRAHFRAIASG-
yente0001X_1297  -----------------VAGL---VILLATDRYLLANLVRPL----DDIRAHFRVIASG-
yruck0001_11920  -----------------VAVV---AVMLIVWFGIQHILINPL----NRLIEHIKHIASG-
ypseu0001X_2707  -----------------VVVL---VMMLLVWLGMQQILIHPL----NHLIAHIKHIANG-
ypest0001X_1916  -----------------VVVL---VMMLLVWLGMQQILIHPL----NHLIAHIKHIANG-
yrohd0001_33620  -----------------IVVL---AVIIFVWLGMQHILINPL----KHLIEHIKHIANG-
yaldo0001_15230  -----------------IAVT---TVIVIVWLGMQRILINPL----KHLIGQIKHIANG-
ykris0001_37850  -----------------IAVF---VVIVIVWLGMQHILINPL----KHLIEHIKHIANG-
yente0001X_1721  -----------------IAVF---VVIVGVWLGMQHILINPL----KHLIEHIKHIANG-
ymoll0001_37090  -----------------IAVL---AVIIVVWLGMQHILINPL----KHLIEHIKHIANG-
yberc0001_15260  -----------------IAVS---AVIILVWLGMQHILINPL----KHLIEHIKHIANG-
yinte0001_40500  -----------------IVVC---AVIIVVWLGMQHILINPL----KHLIEHIKHIANG-
yfred0001_14080  -----------------IVVL---AVVIVVWLGMQHILINPL----KHLIEHIKYIANG-
yruck0001_11930  -----------------VLVI---SMAISCVIWLKTMFVSPL----AIMRDHFDRIAQG-
yaldo0001_6760   ------------------------------------------------------------
ypest0001X_1918  ------------------------------------------------------------
ypest0001X_1917  -----------------LMVI---TMAISSLIWLRNMFVRPL----ATMREHFGRIAQG-
ypseu0001X_2706  -----------------LMVI---TMAISSLIWLRNMFVRPL----ATMREHFGRIAQG-
yrohd0001_33630  -----------------LMVI---VMAISSLIWLRTMFVKPL----KIMRSHFDRIAEG-
yaldo0001_15240  -----------------LMVI---VMAISSLIWLRNMFMKPL----TTMGQHFDRIAQG-
ymoll0001_14920  -----------------LMVI---LMAFSSLIWLRTMFVTPL----KIMRAHFDRIAQG-
yberc0001_15270  -----------------LMVI---LMALSSLVWLRTMFVNPL----KIMSAHFDRIAEG-
yfred0001_14090  -----------------LMVI---AMAISSLVWLRTMFVTPL----KIMRAHFDRIAQG-
yinte0001_15900  -----------------VMVI---AMAISSLVWLRTMFVTPL----KIMRAHFDRIARG-
ykris0001_37840  -----------------LMVI---VMAISALIWLRTMFVTPL----KIMRAHFDRIAQG-
yente0001X_1722  -----------------LMVI---AMAISSLIWLRTMFVSPL----KTMRSHFDRIAKG-
yruck0001_10900  -----------------IFTV---LLTLIAFIVISRVIIKPM----NVLVTRIQRIAEG-
yaldo0001_6770   ------------------------------------------------------------
yaldo0001_15110  -----------------ILAI---VLTAIAFLVISKVIINPI----NWLVERIQRIAQG-
yinte0001_40380  -----------------ILAI---LLTIVAFLVISKVIIKPI----NWLVERIQRIAQG-
ymoll0001_13500  -----------------ILAI---LLTLIAFLVISKVIIKPI----NWLVERIQRIAQG-
yberc0001_14860  -----------------ILAI---LLTLIAFLVISRVIIKPI----NWLVERIQRIAQG-
yrohd0001_33510  -----------------ILAI---LLTLIAFLVISKVIIKPI----NWLVERIKRIAQG-
yfred0001_13960  -----------------ILAL---LLTVIAFLVISKVIIKPI----NWLVERIQRIAQG-
yente0001X_1708  -----------------ILAI---LLTLIAFLVISKVIIKPI----NRLVARIQRIAQG-
ykris0001_38040  -----------------ILAI---LLTVIAFLVISKVIIKPI----NWLVVRIQRIAQG-
yrohd0001_6540   -----------------IAGG---IILLFTDRYIVNYLVQPL----NAIKSHFKLLAEG-
yente0001X_6250  -----------------IAGI---VILILTDRYIVSYLVRPL----DSIKVHFRRLAQG-
yruck0001_10760  ------------------------------------------------------------
ypest0001X_1900  -----------------VLVI---LLTMIAFLVISKVIINPI----NWLVTRIQWIAQG-
ypseu0001X_2718  -----------------VLVI---LLTMIAFLVISKVIINPI----NWLVTRIQRIAQG-
yaldo0001_37010  ------------------------------------------------------------
ypest0001X_1899  ------------------------------------------------------------
yfred0001_6650   -----------------IAGV---IILFLTDRYIVAYLVHPL----NSIKLHFKRLAEG-
yinte0001_6950   -----------------IAGV---IILFLTDRYIVSYLVRPL----ESIKVHFKRLAEG-
ymoll0001_37360  -----------------MAGL---IILLLTDRYIVHYLVRPL----DAIKQHFKRMAEG-
yberc0001_8160   -----------------IAGF---IILLLTDRYIVHYLVRPL----DSIKQHFKRMAEG-
                                                                             


                        550       560       570       580       590       600
                 =========+=========+=========+=========+=========+=========+
ykris0001_41300  EKLLRE---SDRERNELELEGLRLR--NEKLRTENEHLKKGAG-----------------
yrohd0001_20110  -II-----------EEIGKSFSELFAGDESVIKSISSIVG--------------------
yaldo0001_2440   -QILINLLGNAVKFTQQGSISLHVCRKGQKLCFSVEDTGCGIDPQNQLRIFQPFVQNSDY
ypseu0001X_4220  -QILVNLLGNAVKFTPQGRIQLRVRRQNQTLCFTVEDTGCGIDVQHQQTIFQPFMQTSDH
ypest0001X_4540  -QILVNLLGNAVKFTPQGRIQLRVRRQNQTLCFTVEDTGCGIDVQHQQTIFQPFMQTSDH
yinte0001_2660   -TIKRKE--GITIFRPQAVSNNYLSQRRKELLLL--------------------------
yberc0001_2350   -QILINLLGNAIKFTQQGHISLNIERQDKHLCFTVEDTGCGIDIQHQQAIFSPFVQTCDH
ymoll0001_1840   -QILINLLGNAIKFTQQGHISLNVECKDNQLCFTVEDTGSGIDSQHQQAIFSPFVQACDH
ykris0001_2490   -QILINLLGNSVKFTQQGRISLTVERKGQQLCFTVEDTGCGIDLQDQQKIFRPFIQTYDH
yinte0001_2650   -QILVNLLGNAVKFTQCGHIKLTVVRKGQQLCFIVEDTGCGIDLQLQQEIFHPFIQTHEH
yrohd0001_2860   -QILINLLGNAVKFTQQGNISLNVEHKDQQLCFMVEDTGCGIDLQHQQTIFHPFIQTSDH
yfred0001_38320  -QILINLLGNAVKFTQQGHIRLNVEREDSQLCFTVEDTGCGIDLQHQQQIFRPFAQTSDY
yinte0001_26450  ---------------------AVFNNYLEYFRY---------------------------
yberc0001_7120   ------------------------------------------------------------
ypseu0001X_3409  -DLSHRIEDTSFACREIRQLTYSVRHMQEGLVALVSQVRGGAE-----------------
ypest0001X_1004  -DLSHRIEDTSFACREIRQLTYSVRHMQEGLVALVSQVRGGAE-----------------
yente0001X_9160  -DLSHQIEHNAFTSREVRLLAVSIYQMQQGLVVLVSQVRSGVD-----------------
ykris0001_6100   -DLSRQIEHNAFTSREVRQLTDSIYQMQYGLVALVNQVRGGVD-----------------
yfred0001_43450  -DLSRQIGHNAFTSREVHQLADSISQMQQGLVTLVSQVRGGVD-----------------
ymoll0001_7580   -DLSRPIEHQSFTSREVRQLAASICQMQQGLVALVSQVRGGAE-----------------
yinte0001_8280   -DLSRQMAYSSFTSREARQLADSICRMQQGLIALVGQVRVGAE-----------------
ykris0001_6110   -------------------------------IYYMEDKETGSE-----------------
yrohd0001_35950  -EFIIGRFERVNRRGERVWLEASYNPIIDDDGKVLKVVKIAQD-----------------
ypseu0001X_2873  -EFIIGRFERLNRRGERVWLEASYNPIMDNEGNVLKVVKIAQD-----------------
ypest0001X_2787  -EFIIGRFERLNRRGERVWLEASYNPIMDNEGNVLKVVKIAQD-----------------
ymoll0001_11800  -EFIIGRFERVNSRGQPVWLEASYNPIMDNEGQVIKVVKIAQD-----------------
yfred0001_8890   -EFIIGRFERVNSRGERVWLEASYNPIMDNEGQIFKVVKIAQD-----------------
ykris0001_12740  -EFIVGRFERVNSRGERVWLEASYNPIMDKEGQVLKVVKIAQD-----------------
yente0001X_1434  -EFIVGRFERMNSRGQRVWLEASYNPIMDKEGQVLKVVKIAQD-----------------
yrohd0001_31880  -QLKKNN-----------QLLLELEKDVETLSQQ--------------------------
yrohd0001_15520  -NLSTEI---KLRSGDTHSLLASLNTMNQQLKKTINEIMHGSE-----------------
yfred0001_15260  -NLSTEI---KLRPGDNHSLLASLNTMNQQLKKTINEIMHGSE-----------------
yinte0001_16590  -NLSTEI---KLRPGDNHSLLASLNIMNQQLKKTINEIMHGSE-----------------
ymoll0001_41560  -NLSTEI---KLRPGDQNSLLASLNTMNQQLKSTINEIMHGSE-----------------
yberc0001_16020  -NLSTEI---KLRPGDKNSLLASLNTMNQQLKSTINEIMHGSE-----------------
yberc0001_5770   -DLSEEI--QVGDENELGLMLVELEKMRVSLTGTVSNINDGVS-----------------
ymoll0001_5080   -DLSEEI--HAGDQNELGLMLIELEKMRLSLTKIISGIRDGVS-----------------
yaldo0001_37020  ------------------------------------------------------------
ypseu0001X_2733  -EAGDYV--QLNRVDEIGYLMRSVNQLGLNLRSLTDDVSGQVD-----------------
ypest0001X_1885  -EAGDYV--QLNRVDEIGYLMRSVNQLGLNLRSLTDDVSGQVD-----------------
yrohd0001_12010  -EAGDYV--QLNRVDEIGYLMRSVNQLGLNLRSLTDDVSSQVD-----------------
yfred0001_12940  -QAGNYV--QLNRIDEIGYLMRSVNQLGLNLRSLTDDVGGQVD-----------------
yinte0001_14220  -QAGDYV--QLNRVDEIGYLMRSVNQLGLNLRSLTDDVSGQVD-----------------
ymoll0001_14640  -QAGDYV--QLNRVDEIGYLMRSVNQLGLNLRSLTDDVSGQVD-----------------
yberc0001_15000  -QAGDYV--QLNRVDEIGYLMRSVNQLGLNLRSLTDDVSGQVD-----------------
ykris0001_42780  -QAGDYV--QLNRVDEIGYLMRSVNQLGLNLRSLTDDVSGQVD-----------------
yente0001X_2568  -QAGDYV--QLNRVDEIGYLMRSVNQLGLNLRSLTDDVSGQVD-----------------
yberc0001_5850   -QLAKKV--PQYGSNEIGKLFVNIELMRKSLAEIISGVKEAAT-----------------
ymoll0001_5160   -LLSKRV--PQYGTNEVGKLFANIEEMRSGLVEIIAGVKEAAS-----------------
yberc0001_6170   -DLNSDI--SYKSKSEIGFLFSCLEDMQNSLKKIISSVKGSTI-----------------
yaldo0001_6750   -QLGRPM--AEFGRNH--------------------------------------------
yfred0001_39550  -DLTHSI--NS-------------------------------------------------
yrohd0001_27170  -DLTHNI--NSEGNTELARLAKALQTMQQSLVVSVSNVRDVGG-----------------
ykris0001_32930  -DLTHSI--NSEGNTELARLARALETMQQSLVVSVSNVRDVGE-----------------
yente0001X_3909  -DLTHNI--NSEGNTELARLAKALQTMQQSLVASVSNVRDVGG-----------------
ymoll0001_40600  -DLTHSI--NSEGNTELARLAKALQVMQQSLVVSVSNVRDVGG-----------------
yberc0001_29090  -DLTHNI--NSEGNTELARLAKALQVMQQSLVESVSNVRDVGG-----------------
ypseu0001X_1449  ADLTQRL--PIEGRDEVAQIARSFNQFADKLSLVMAQIRGTSE-----------------
ypest0001X_1447  ADLTQRL--PIEGRDEVAQIARSFNQFADKLSLVMAQIRGTSE-----------------
yfred0001_36240  ------------------------------------------------------------
yinte0001_10140  EDLTQRL--PVEGNDEVAKIALSFNNFADKLSVVMAQIRDTSE-----------------
ykris0001_10260  EDLTQRL--PVEGRDEVAKIAISFNNFADKLSSVMAQIRNTSE-----------------
yente0001X_2906  EDLTQRL--PVEGHDEVAKIALSFNNFADKLSGVMAQIRNTSE-----------------
yfred0001_36230  EDLTQRL--PVEGRDEVAKIALSFNNFADKLSAVMAQIRDTSE-----------------
ymoll0001_9480   EDLTQRL--PVEGRDEVAKIAESFNRFADKLSAVMAQIRDTSE-----------------
yberc0001_9790   EDLTRRL--PVEGRDEVAKIAQSFNQFADKLSAVMAQIRDTSE-----------------
yruck0001_28810  ADLTQRL--PASSSPEFNAINQSFNQFIDYLQQMMRQVGESSL-----------------
yrohd0001_31660  ADLTQRL--PQSNSPEFNAVNQSFNDFIDYLQQMMRQVGESSL-----------------
yaldo0001_35590  ADLTQRL--PQSSSPEFNAINQSFNDFIDYLQQMMRKVGESSL-----------------
ykris0001_37060  ADLTQRL--PQSSSPEFNAINQSFNDFIDYLQQMMRQVGESSL-----------------
yinte0001_37560  ADLTQRL--PESSSPEFNAINQSFNDFIDYLQQMMRQVGESSL-----------------
yfred0001_32450  ADLTQRL--PQSTSPEFNAINQSFNDFIDYLQQMMRQVGESSL-----------------
yente0001X_300   ADLTQRL--PQSSSPEFNAINQSFNDFIDYLQQMMRQVGESSL-----------------
ymoll0001_35290  ADLTQRL--PESSSPEFNAINQSFNAFIDYLQQMMRQVGESSL-----------------
yberc0001_34000  ADLTQRL--PESSSPEFNAINQSFNAFIDYLQQMMRQVGESSL-----------------
ykris0001_22290  -DLTSSI--VTNRKDEAGLLLQSLDHMNTSLRQIVSKVRDGAE-----------------
yinte0001_3170   -VLDHNI--VDQGKNCIGQLVPYINKMQDNWAKTVFEIRNSAD-----------------
yruck0001_31650  -NLSIEI--DANGNNEIGKLMQGLKHMRDELANTVSAVRAGTQ-----------------
ymoll0001_7110   -DLSVSV---DTKPNDQTSLLVAMKIMRDSLAKIVNEVRTGTE-----------------
yruck0001_28370  -DLTSAT--TSSRTDELGLLMNAVARMNENLRGMIDEIRIGVS-----------------
ypseu0001X_4346  -DLTAAT--TSSRTDELGMLMNAVARMNENLRAMIDEIRIGVS-----------------
ypest0001X_4370  -DLTAAT--TSSRTDELGMLMNAVARMNENLRAMIDEIRIGVS-----------------
yrohd0001_31240  -DLTTAT--KSNRTDELGLLMNAVARMNENLRAMIDDIRVGVS-----------------
yaldo0001_35130  -DLTAAA--TSTRTDELGLLMNAVARMNENLRAMIDEIRVGVS-----------------
yinte0001_36970  -DLTATT--TSHRTDELGLLMNAVARMNENLRAMIDEIRIGVS-----------------
ykris0001_39870  -DLTAET--TSTRTDELGLLMNTVARMNENLRAMIDEIRIGVS-----------------
ymoll0001_34760  -DLTAAA--TSDRTDELGLLMNAVARMNENLRAMIDEIRIGVS-----------------
yfred0001_31920  -DLTAAT--TSNRTDELGLLMNAVARMNENLRAMIDEIRIGVS-----------------
yberc0001_33520  -DLTAAT--TSNRTDELGLLMNAVARMNENLRAMIDEIRIGVS-----------------
yente0001X_760   -DLTAAT--TSNRTDELGLLMNAVARMNENLRAMIDEIRIGVS-----------------
yruck0001_18270  -QLGHPI--VDFGRNCIGQLFPLLREVQTSLANTVKTIRDSTD-----------------
yrohd0001_33070  -QLGQPI--VDFGHNCVGQLFPLLREVQTSLVNTVKAIRSSTD-----------------
yfred0001_34740  -QLGRPI--VDFGRNCVGQLFPLLRDVQTSLVNTVQAIRSSTD-----------------
yaldo0001_22600  -QLGQPI--ADFGRNCVGRLFPLLRDVQTSLVNTVQTIRSSTD-----------------
yinte0001_40830  -QLGHPI--TDFGRNCVGRLFPLLRDVQTSLVNTVQAIRSSTD-----------------
ymoll0001_38830  -QLGQPI--VDFGRNCVGQLFPLLRDVQTSLVNTVEAIRSSTD-----------------
yberc0001_21820  -QLGQPI--VDFGRNCVGQLFPLLRDVQTSLVNTVEAIRSSTD-----------------
ykris0001_22260  -QLGQPI--TDFGRNCVGQLFPLLRDVQASLANTVKAIRSSTD-----------------
yente0001X_1297  -QLGQPI--TDFGRNCVGKLFPLLRDVQASLANTVKAIRSSTD-----------------
yruck0001_11920  -DLTQPI--EVLSRNEMGTLAASLSHMQSELIGTVTGVRMGAD-----------------
ypseu0001X_2707  -DLTQNI--EVHGRNEVGTLAASLKHMQSELVTIVGEVRVGAD-----------------
ypest0001X_1916  -DLTQNI--EVHGRNEVGTLAASLKHMQSELVTIVGEVRVGAD-----------------
yrohd0001_33620  -DLTQTI--EVNSRNEMGVLAASLKNMQSELITTVSDVRLGAD-----------------
yaldo0001_15230  -DLTQNI--EVHSHNEMGTLAASLKHMQAELITTVSHVRLGAD-----------------
ykris0001_37850  -DLTQNI--EVHSRNEMGTLAASLKHMQSELITTVSDVRLGAD-----------------
yente0001X_1721  -DLTQNI--EVHSRNEMGTLAASLKHMQSELVTTVSDVRLGAD-----------------
ymoll0001_37090  -DLTQNI--DVHSRNEMGTLAASLKHMQSELATTVNDVRLGAD-----------------
yberc0001_15260  -DLTQTI--EVHSRNEMGTLAASLKHMQSELATTVNDVRLGAD-----------------
yinte0001_40500  -DLTQTI--EVHSRNEMGTLAASLKHMQTELITTVSDVRLGAD-----------------
yfred0001_14080  -DLTQTI--EVHSRNEMGTLAASLKHMQSELVTTVSDVRLGAD-----------------
yruck0001_11930  -DLAGKI--SVSGRNEISQLFASLRIMQQSLISTVSKVRDGTE-----------------
yaldo0001_6760   ------------------------------------------------------------
ypest0001X_1918  ------------------------------------------------------------
ypest0001X_1917  -DLSGQI--SVTGRNEISQMFASLRTMQQSLISTVSNVREGTE-----------------
ypseu0001X_2706  -DLSGQI--SVTGRNEISQMFASLCTMQQSLISTVSNVREGTE-----------------
yrohd0001_33630  -DLSGQI--SVSGRNEISEMFGSLHTMQQSLITTVSHVRDGTE-----------------
yaldo0001_15240  -DLSGRI--SVSGRNEISQLFASLHIMQQSLITTVSHVRDGTE-----------------
ymoll0001_14920  -DLSAQI--TVSGRNEISQMFASLRTMQHSLISTVSHVRDGTE-----------------
yberc0001_15270  -DLSAHI--DVSGRNEISQLFASLRTMQRSLITTVSHVRDGTE-----------------
yfred0001_14090  -DLSGQI--YVSGRNEISQMFASLRTMQRSLITTVSHVRDGTE-----------------
yinte0001_15900  -DLSAQI--YVSGRNEISEMFASLRIMQQSLITTVSHVRDGTE-----------------
ykris0001_37840  -DLSGQI--TVSGRNEISQMFASLRTMQQSLITTVSHVRDGTE-----------------
yente0001X_1722  -DLSAQI--SVTGRNEISQMFASLRTMQQSLITTVSHVRDGTE-----------------
yruck0001_10900  -DLTQPA--IPFGSNEIGVLGSNIQQMQKSLSVTVASVRSSAE-----------------
yaldo0001_6770   ------------------------------------------------------------
yaldo0001_15110  -DLTQAC--GSFGRNEIGVLGKNIQQMQDSLSTTVEAVRSSAE-----------------
yinte0001_40380  -DLTQTS--VSFGRNEIGVLGNNIQQMQDSLSITVGAVRSSAE-----------------
ymoll0001_13500  -DLTQMP--VAFGRNEIGVLGNNIQQMQDSLSTTVEAVRSSAE-----------------
yberc0001_14860  -DLTQTP--VAFGRNEIGVLGNNIQQMQDSLSTTVEAVRSSAE-----------------
yrohd0001_33510  -DLTQSA--VLFGRNEIGVLGSNIQQMQDSLSSTVEAVRSSAE-----------------
yfred0001_13960  -DLTQSS--VSFGRNEIGVLGNNIQQMQDSLSLTVEAVRSSAE-----------------
yente0001X_1708  -DLTQHP--APFGRNEIGVLGNNIQQMQDSLSSTVEAVRSSAE-----------------
ykris0001_38040  -DLTQSP--VAFGRNEIGVLGHNIQQMQDSLASTVEAVRSSAE-----------------
yrohd0001_6540   -QLGHPM--TEFGRNCVGQLIPYLREMQNSLVNTVSTIRNSTD-----------------
yente0001X_6250  -QLGRPM--AEFGRNCVGQLIPYLQEMQNSLVNTVSTIRSSTD-----------------
yruck0001_10760  ------------------------------------------------------------
ypest0001X_1900  -DLTQSP--VSFGRNEIGVLGSNIQQMQDALAITVEAVRSSAE-----------------
ypseu0001X_2718  -DLTQSP--VSFGRNEIGVLGSNIQQMQDALAITVEAVRSSAE-----------------
yaldo0001_37010  ------------------------------------------------------------
ypest0001X_1899  ------------------------------------------------------------
yfred0001_6650   -QLGRPM--AEFGRNCVGQLIPYLREMQNSLVNTVSTIRNSTD-----------------
yinte0001_6950   -QLGRPM--PEFGRNCVGQLIPYLREMQSSLVNTVSTIRNSTD-----------------
ymoll0001_37360  -QLGRPM--AEFGRNCVGQLIPYLREMQTSLVSTVSTIRSSTD-----------------
yberc0001_8160   -QLGRPM--AEFGRNCVGQLIPYLREMQNSLVNTVSTIRSSTD-----------------
                                                                             


                        610       620       630       640       650       660
                 =========+=========+=========+=========+=========+=========+
ykris0001_41300  ------------------------------------------------------------
yrohd0001_20110  --------KAFQAMMGVGKGQEGVSEMYEVIAEYP----------------------AIV
yaldo0001_2440   AQGTGLGLTIADNLAGMMGGHLVLTSQTDRGSRFSLILPLSETAQGRLFDGELCVP-HSL
ypseu0001X_4220  EQGTGLGLAIADNLAKMMGGHLTVFSEPGQGSCFSLCLPFNAITPPMPFHGELFAP-QRL
ypest0001X_4540  EQGTGLGLAIADNLAKMMGGHLTVFSEPGQGSCFSLCLPFNAITPPMPFHGELFAP-QRL
yinte0001_2660   --------PIFPTHDNIFWG----------------------------------------
yberc0001_2350   RHGTGLGLTIADNLARMMGGEITLFSQLGQGSRFTLSLPYQGITPAKPFKGELCAP-SSL
ymoll0001_1840   RHGTGLGLTIADNLARMMGGKITLSSQLGQGSRFTLSLPYHGVIPVKPFNSKLGAP-PSL
ykris0001_2490   GQGTGLGLTIADNLAKMMGGHITLYSKPNSGSRFSLTLPFNGITPAIPFKGELYAPLSPL
yinte0001_2650   GQGTGLGLTIADNLARMMGGNITLFSLPDHGSRFSLNLPFSGNTAVQLFKGEIYAP-QPL
yrohd0001_2860   GQGTGLGLTIADNLARMMGGNITLHSQPNKGSRFTLILPLCGTVPAKLFKGEFAAP-QSL
yfred0001_38320  GQGTGLGLTIADNLARMMGGQITLYSQPNCGSRFVLILPFSGIAGATPFQGECYAP-LPL
yinte0001_26450  ------------------------------------------------------------
yberc0001_7120   ------------------------------------------------------------
ypseu0001X_3409  --------VILTGVNQITADNHRLSEQTYSQTQSL----------------------AVT
ypest0001X_1004  --------VILTGVNQITADNHRLSEQTHSQTQSL----------------------AVT
yente0001X_9160  --------VILTGVNQIAADSHRISEQTQSQTQSL----------------------AAT
ykris0001_6100   --------LILTGVNQIAADSHRISEQTQSQTLSL----------------------ATT
yfred0001_43450  --------VILAGVNQIAADNHRISEQTQSQTQSL----------------------AAT
ymoll0001_7580   --------VILTGINQIAVDSHRLSEQTQSQTQSL----------------------AAT
yinte0001_8280   --------VILTGVNQIAADSHRLAEQTQSQAESL----------------------AAT
ykris0001_6110   ---------------------------------------------------------GSW
yrohd0001_35950  --------------------ITQLMLQQEHE--------------------------ENL
ypseu0001X_2873  --------------------ITALMLQQEQEENLI----------------------RDV
ypest0001X_2787  --------------------ITALMLQQEQE--------------------------ENL
ymoll0001_11800  --------------------ITQLMLQQEHEENLI----------------------RDV
yfred0001_8890   --------------------ITPLMLQQEYEENLI----------------------RDV
ykris0001_12740  --------------------ITQLMLQQEHEENLI----------------------RDV
yente0001X_1434  --------------------ITPLMLQQEQE--------------------------ENL
yrohd0001_31880  ------------------------NARYKDKIIEL----------------------ERN
yrohd0001_15520  --------SISVASNQIAQGNTDLSQRTEEQASSL----------------------IQT
yfred0001_15260  --------SISVASHQIAQGNTDLSQRTEEQASSL----------------------IQT
yinte0001_16590  --------SISVASNQIAQGNADLSQRTEEQASSL----------------------IQT
ymoll0001_41560  --------SISVASNQIAQGNTDLSQRTEEQASSL----------------------IQT
yberc0001_16020  --------SISVASNQIAQGNTDLSQRTEEQASSL----------------------IQT
yberc0001_5770   --------RIYSNAQEIAQGNNDLSARTEEQASAL----------------------QQT
ymoll0001_5080   --------HIYGNAQEISLGNNDLSARTEEQASAL----------------------QQT
yaldo0001_37020  ------------------------------------------------------------
ypseu0001X_2733  --------GINTASNEIAAGNRELKVRTEQATDNL----------------------QHI
ypest0001X_1885  --------GINTASNEIAAGNRELKVRTEQATDNL----------------------QHI
yrohd0001_12010  --------GISTASREIAAGNRELKVRTEHATANL----------------------QHI
yfred0001_12940  --------GINTASSEIAAGNRELKARTEQATANL----------------------QHI
yinte0001_14220  --------GINTASSEIAAGNRELKVRTEQATANL----------------------QHI
ymoll0001_14640  --------GINTASSEIAAGNRELKVRTEQATANL----------------------QHI
yberc0001_15000  --------GINTASSEIAAGNRELKVRTEQATANL----------------------QHI
ykris0001_42780  --------GINTASSEIAAGNRELKVRTEQATANL----------------------QHI
yente0001X_2568  --------GINTASSEIAAGNRELKVRTEQATANL----------------------QHI
yberc0001_5850   --------NIKNNSVEIAEGNNDLSSRTEEQASAL----------------------QET
ymoll0001_5160   --------NIKHNAMEIAEGNDDLSSRTEEQASAL----------------------QQT
yberc0001_6170   --------IITRGSTEIADANFNLSSRIEQQASAL----------------------QET
yaldo0001_6750   ---------------------------------------------------------EQH
yfred0001_39550  ------------------------------------------------------------
yrohd0001_27170  --------QIGVGSRELAAGNHHLAARTEESASSL----------------------EQT
ykris0001_32930  --------QIGVGSRELAAGNNHLAARTEESASSL----------------------EQT
yente0001X_3909  --------QIGIGSRELAAGNNHLAERTEESASSL----------------------EQT
ymoll0001_40600  --------QIGVGSRELAAGNTHLAERTEESASSL----------------------EQT
yberc0001_29090  --------QIGIGSRELAAGNTHLAERTEESASSL----------------------EQT
ypseu0001X_1449  --------SVRVAANEIAAGNQDLSGRTESAAASL----------------------QQT
ypest0001X_1447  --------SVRVAANEIAAGNQDLSGRTESAAASL----------------------QQT
yfred0001_36240  ---------------------------------------------------------KDT
yinte0001_10140  --------SVRIAANEIAAGNQDLSGRTESAAASL----------------------QQT
ykris0001_10260  --------SVSIAANEIAAGNQDLSGRTESAAASL----------------------QQT
yente0001X_2906  --------SVSIAANEIAAGNQDLSGRTESAAASL----------------------QQT
yfred0001_36230  --------SVRIAANEIAAGNQDLSGRTESAAASL----------------------QQT
ymoll0001_9480   --------SVRIAANEIAAGNQDLSGRTESAAASL----------------------QQT
yberc0001_9790   --------SVRIAANEIAAGNQDLSGRTESAAASL----------------------QQT
yruck0001_28810  --------AIASASRQIASGNLDLSARTEDQASAI----------------------EQT
yrohd0001_31660  --------AIASASRQIASGNMDLSARTEDQASSI----------------------EQT
yaldo0001_35590  --------AIASASRQIASGNLDLSARTEDQASSI----------------------EET
ykris0001_37060  --------AIASASRQIASGNLDLSARTEDQASSI----------------------EET
yinte0001_37560  --------AIASASRQIASGNLDLSARTEDQASSI----------------------EQT
yfred0001_32450  --------AIASASRQIASGNLDLSARTEDQASSI----------------------EQT
yente0001X_300   --------AIASASRQIASGNLDLSARTEDQASSI----------------------EET
ymoll0001_35290  --------AIASASREIASGNLDLSARTEDQASSI----------------------EET
yberc0001_34000  --------AIASASREIASGNLDLSARTEDQASSI----------------------EET
ykris0001_22290  --------TISTAASQIAAGNQDLSARTEEQASSL----------------------EQT
yinte0001_3170   --------SIYRGSSEISVGNTDLSSRTEEQASAL----------------------EET
yruck0001_31650  --------NIYTGVQEIAAGNSDLSSRTEEQASSL----------------------EET
ymoll0001_7110   --------TMVTSSCQIASGNQDLSSRTEQQASSL----------------------EET
yruck0001_28370  --------QVSHASGEIAAGNTDLSSRTEQQAAAV----------------------EET
ypseu0001X_4346  --------QVSHASGEIAAGNTDLSSRTEQQAAAV----------------------EET
ypest0001X_4370  --------QVSHASGEIAAGNTDLSSRTEQQAAAV----------------------EET
yrohd0001_31240  --------QVSHASGEIAAGNTDLSSRTEQQAAAV----------------------EET
yaldo0001_35130  --------QVSHASGEIAAGNTDLSSRTEQQAAAV----------------------EET
yinte0001_36970  --------QVSHASGEIAAGNTDLSSRTEQQAAAV----------------------EET
ykris0001_39870  --------QVSHASGEIAAGNTDLSSRTEQQAAAV----------------------EET
ymoll0001_34760  --------QVSHASGEIAAGNTDLSSRTEEQAAAV----------------------EET
yfred0001_31920  --------QVSHASGEIAAGNTDLSSRTEQQAAAV----------------------EET
yberc0001_33520  --------QVSHASGEIAAGNTDLSSRTEEQAAAV----------------------EET
yente0001X_760   --------QVSHASGEIAAGNTDLSSRTEQQAAAV----------------------EET
yruck0001_18270  --------SIYHGAAEIAAGNTDLSSRTEQQAAAL----------------------EET
yrohd0001_33070  --------GIYQGAAEIAAGNTDLSSRTEQQAAAL----------------------EET
yfred0001_34740  --------GIYHGAAEISAGNTDLSSRTEQQAAAL----------------------EET
yaldo0001_22600  --------GIYHGAAEISAGNTDLSSRTEQQAAAL----------------------EET
yinte0001_40830  --------GIYHGAAEISAGNTDLSSRTEQQAAAL----------------------EET
ymoll0001_38830  --------GIYHGAAEISAGNTDLSSRTEQQAAAL----------------------EET
yberc0001_21820  --------GIYHGAAEISAGNTDLSSRTEQQAAAL----------------------EET
ykris0001_22260  --------GIYHGAAEISAGNTDLSSRTEQQAAAL----------------------EET
yente0001X_1297  --------GIYHGAAEISAGNTDLSSRTEQQAAAL----------------------EET
yruck0001_11920  --------AIYSGASEIAAGNNDLSARTEQQAASL----------------------EET
ypseu0001X_2707  --------AIYSGASEIAAGNNDLSARTEQQAASL----------------------EET
ypest0001X_1916  --------AIYSGASEIAVGNNDLSARTEQQAASL----------------------EET
yrohd0001_33620  --------AIYSGASEIAAGNNDLSARTEQQAASL----------------------EET
yaldo0001_15230  --------AIYSGASEIAAGNNDLSARTEQQAASL----------------------EET
ykris0001_37850  --------AIYSGASEIAAGNNDLSARTEQQAASL----------------------EET
yente0001X_1721  --------AIYSGASEIAAGNNDLSARTEQQAASL----------------------EET
ymoll0001_37090  --------AIYSGASEIAAGNNDLSARTEQQAASL----------------------EET
yberc0001_15260  --------AIYSGASEIAAGNNDLSARTEQQAASL----------------------EET
yinte0001_40500  --------AIYSGASEISAGNNDLSARTEQQAASL----------------------EET
yfred0001_14080  --------AIYSGASEIAAGNNDLSARTEQQAASL----------------------EET
yruck0001_11930  --------SMLTGIQEISAGNNDLSARTEQQAASL----------------------EET
yaldo0001_6760   ------------------------------------------------------------
ypest0001X_1918  ------------------------------------------------------------
ypest0001X_1917  --------SMLTGIQEISAGNNDLSARTEQQAASL----------------------EQT
ypseu0001X_2706  --------SMLTGIQEISAGNNDLSARTEQQAASL----------------------EQT
yrohd0001_33630  --------SMLTGIQEISAGNNDLSARTEQQAASL----------------------EQT
yaldo0001_15240  --------SMLTGIQEISAGNNDLSARTEQQAASL----------------------EQT
ymoll0001_14920  --------SMLTGIQEISAGNNDLSARTEQQAASL----------------------EQT
yberc0001_15270  --------SMLTGIQEISAGNNDLSARTEQQAASL----------------------EQT
yfred0001_14090  --------AMLTGIQEISAGNNDLSARTEQQAASL----------------------EQT
yinte0001_15900  --------AMLTGIQEISAGNNDLSARTEQQAASL----------------------EQT
ykris0001_37840  --------SMLTGIQEISAGNNDLSARTEQQAASL----------------------EQT
yente0001X_1722  --------SMLTGIQEISAGNNDLSARTEQQAASL----------------------EQT
yruck0001_10900  --------SIYQGSSEIASGNIDLSARTEQQAASL----------------------EQT
yaldo0001_6770   ------------------------------------------------------------
yaldo0001_15110  --------SIYKGSSEIALGNTDLSARTEQQAAAL----------------------EQT
yinte0001_40380  --------SIYQGSSEIALGNTDLSARTEQQAASL----------------------EQT
ymoll0001_13500  --------SIYQGSSEIALGNTDLSARTEQQAASL----------------------EQT
yberc0001_14860  --------SIYQGSSEIALGNTDLSARTEQQAASL----------------------EQT
yrohd0001_33510  --------SIYQGSSEIALGNTDLSARTEQQAASL----------------------EQT
yfred0001_13960  --------SIYQGSSEIALGNTDLSARTEQQAASL----------------------EQT
yente0001X_1708  --------SIYQGSSEIALGNTDLSARTEQQAASL----------------------EQT
ykris0001_38040  --------SIYQGSSEIALGNTDLSARTEQQAASL----------------------EQT
yrohd0001_6540   --------AIYTGAGEIAAGNADLSSRTEQQAAAL----------------------EET
yente0001X_6250  --------AIYTGAGEIAAGNADLSSRTEQQAAAL----------------------EET
yruck0001_10760  ------------------------------------------------------------
ypest0001X_1900  --------SIYQGSSEIALGNTDLSARTEQQAASL----------------------EQT
ypseu0001X_2718  --------SIYQGSSEIALGNTDLSARTEQQAASL----------------------EQT
yaldo0001_37010  ------------------------------------------------------------
ypest0001X_1899  ------------------------------------------------------------
yfred0001_6650   --------AIYTGAGEIAAGNADLSSRTEQQAAAL----------------------EET
yinte0001_6950   --------AIYTGAGEIAAGNADLSSRTEQQAAAL----------------------EET
ymoll0001_37360  --------AIYTGAGEIAAGNADLSSRTEQQAAAL----------------------EET
yberc0001_8160   --------AIYTGAGEIAAGNADLSSRTEQQAAAL----------------------EET
                                                                             


                        670       680       690       700       710       720
                 =========+=========+=========+=========+=========+=========+
ykris0001_41300  ----------------------------------GG------------------------
yrohd0001_20110  RFDFKFWYRNIESESIFKYMENVFAVTA--------------------------------
yaldo0001_2440   HAQLSAWGITCQTPDDESSQRLPALFTDKELNYLPGRLYANVKRHLAGNHQSGEYSQENA
ypseu0001X_4220  HAQLSAWGMTCQPELANQPSRY---FVDNALCYLPGRLYANLKQYLQGAET---------
ypest0001X_4540  HAQLSAWGMTCQPELANQPSRH---FVDNALCYLPGRLYAKLKQYLQGAETEA-------
yinte0001_2660   ------------------------------------------------------------
yberc0001_2350   HPQLSAWGITCLPENNESLLSPSGPFTDKELNYLPGRLYTKVEKYLSDQNSPE----LSV
ymoll0001_1840   HPQLSAWGIICPPANNESSLSPLGPFTDKELNYLPGRLYTKVEKYLSRHHAPV----PST
ykris0001_2490   QAQLSAWGITCQSKNNQSDFLPLTLFAEKELCYLPGRLYTKVKQYLDNKRHAT----PYQ
yinte0001_2650   HHQLSAWGITCQSVSSGSVHKQQETFSDKELCYLPGRLYSKVKKHLNGEDQHA----SSQ
yrohd0001_2860   HIQLSTWGINCLPSNNKSLNLPPAFLSDKELCYLPGRLYTKVKNYLDDKSPPN----LHQ
yfred0001_38320  HAQLSAWGVTCLTESNQSLSMQQTLLTDNELCYLPGRLYTKVKKYLDIQNQLI----PYQ
yinte0001_26450  --------ETIFKNHDNIYK----------------------------------------
yberc0001_7120   ------------------------------------------------------------
ypseu0001X_3409  AQSMQQLTVRVKQNSMSAEQANHLVNEARDTASQGGEMMSNVVSSMADISTGS----REI
ypest0001X_1004  AQSMQQLTVRVKQNSMSAEQANHLVNEARDTASQGGEMMSNVVSSMADISTGS----REI
yente0001X_9160  TQNMYQLTERVKQNSKSADQANQLANEAKNIASQGGDMMSGVVNSMADISAGS----QEI
ykris0001_6100   TLNMHQLTERVKQNSLSADQANHLAIEAKNIASQGGDMMSSVVSSMADISAGS----QEI
yfred0001_43450  TLNMSQLTVRVKQNSLSADQANHLANEAKDIASHGGDMMSRVVNSMADISTGS----QEI
ymoll0001_7580   TQSMHQLTLRVKENSISADQANYLANKTKSIASQGGEMMSSVVSSMADISSGS----REI
yinte0001_8280   TQSMHQLTSRVKQNSMSADQANLLANETSAIASEGGEMMSSVVVSMADISAGS----QEI
ykris0001_6110   QSLLSEAMLSMQQSQDNYRQLLQLFAHDER------------------------------
yrohd0001_35950  FRNVHDLSLNTDRSASQGAAIVQQAVRGMQEVELIARETSDVVSNLGR---CS----QEI
ypseu0001X_2873  HHLSLTTDRSASQGAEIVQQAVRGMQEVESIARETSDVVSDL-------GRCS----QEI
ypest0001X_2787  IRDVHHLSLTTDRSASQGAEIVQQAVRGMQEVESIARETSDVVSDLGRCS-------QEI
ymoll0001_11800  HHLSLTTDRSASQGAEIVQQAVRGMQEVESIARETSDVVSDL-------GRCS----QEI
yfred0001_8890   HHLSLTTDRSASQGAEIVQQAVRGMQEVESIARETSDVVSDL-------GRCS----KEI
ykris0001_12740  HHLSLTTDRSASQGAEIVQQAVRGMQEVESVARETSGVISEL-------GRCS----KEI
yente0001X_1434  IRDVHHLSLTTDRSASQGAEIVQQAVRGMQEVESVARETSDVISELGRCS-------NEI
yrohd0001_31880  QSSLEKTVSTLEQNVKN-------------------------------------------
yrohd0001_15520  SANMQELTQTVRQNADNARLASELAINTAATATEGGIIVDEMLLRMQEISNSS----KKI
yfred0001_15260  SANMQELTQTVRQNADNARQASELAINTAATATEGGIIVDEMLLRMQEISNSS----KKI
yinte0001_16590  SANMQELTQTVRQNADNARQASELAVNTAATATEGGAIVDEMLLRMQEITKSS----KKI
ymoll0001_41560  SANMQELTQTVRQNADNARQASELAINTSATATEGGIIVDEMRLHMQEISNSS----KKI
yberc0001_16020  SANMQELTQTVRQNADNARQASELAVNTAATATEGGVIVDEMLLRMQEISNSS----KKI
yberc0001_5770   AASMEQLKTTVRQNADNAHTARQLAESASLNARNGGNVMTNLDDIMQQITQSS----RQI
ymoll0001_5080   AASMEELKTTVRQNADNAHTARQLAENASLNARSGGDVMTNLEGIMQQITQSS----RQI
yaldo0001_37020  ------------------------------------------------------------
ypseu0001X_2733  VSATEQLAATVQNSANSANETTSLAEMSSHAAEKGSELMHQVIETMGTINDSS----HRI
ypest0001X_1885  VSATEQLAATVQNSANSANETTSLAEMSSHAAEKGSELMHQVIETMGTINDSS----HRI
yrohd0001_12010  VSATDQLVATVQNSANTANETTSLAEMSSHAAEKGSELMHQVIETMGTINASS----HRI
yfred0001_12940  VSATEQLVATVQNSANTANETTSLAEMSSHAAEQGSELMHQVIETMGSINDSS----HRI
yinte0001_14220  VSATEQLVATVQHSANSANETTSLAAMSSHAAEQGSELMHQVIETMGTINASS----HRI
ymoll0001_14640  VSATEQLVATVQNSANSANETTSLAAMSSHAAEQGSELMHQVIETMGTINDSS----HRI
yberc0001_15000  VSATEQLVATVQNSANSANETTSLAAMSSHAAEQGSELMHQVIETMGTINDSS----HRI
ykris0001_42780  VSATEQLVATVQNSANSANETTSLATMSSHAAERGSELMHQVINTMGTINDSS----HRI
yente0001X_2568  VSATEQLVATVQNSANSANETTSLAAMSSHAAEQGSELMHEVIKTMGTINDSS----HRI
yberc0001_5850   AASMEQIKTTVENNTTHAHEANKLAMDTKDMADSGSLIMVDVVHSMDTIANHA----SQI
ymoll0001_5160   AASMEQIKTTVENNTAHAHEANKLATDTKELADSGSLIMVDVVRSMDTIGSHA----NQI
yberc0001_6170   AASMEEIKTTVLSNAQNAQQTNQLSHTASLAAKNGADIMHKVVSTMADIETLT----KKI
yaldo0001_6750   YRKLTPYCRYYRRNKQYC-----------------------LPDKYSGVECRS-------
yfred0001_39550  ------------------------------------------------------------
yrohd0001_27170  AASMEQLTATVKMNAENSDQANRLAMSVSDIANKGSKAVSHVVDKMQAITTSS----RRI
ykris0001_32930  AASMEQLTSTVKMNAENSDQANRLAMSVSDIANKGSKVVSHVVDKMQAITTSS----RRI
yente0001X_3909  AASMEQLTSTVKMNAENSDQANRLAMSVSDIASKGSKVVSHVVDKMQAITTSS----RRI
ymoll0001_40600  AASMEQLTSTVKMNAENSDQANRLAMSVSDIANKGSEAVSHVVDKMQAITSSS----RRI
yberc0001_29090  AASMEQLTSTVKMNAENSDQANRLAMSVSDIANKGSEAVSHVVDKMQAITSSS----RRI
ypseu0001X_1449  SAALEQISATVAQSASAARQANNAVFSASEDASRGGDVITKVITTMESIEKAS----GKI
ypest0001X_1447  SAALEQISATVAQSASAARQANNAVFSASEDASRGGDVITKVITTMESIEKAS----GKI
yfred0001_36240  ALAAD-------------------------------------------------------
yinte0001_10140  SAALEQISATVAHSASAARQANTAVLSAASDASRGGDVIAKVITTMESIEAAS----GKI
ykris0001_10260  SAALEQISATVAQSASAARQANTAVLSAANDASRGGEVIAKVITTMESIEAAS----GKI
yente0001X_2906  SAALEQISATVAQSASAARQANTAVLSAANDASRGGEVIAKVITTMESIEAAS----GKI
yfred0001_36230  SAALEQISATVAQSASAARQANTAVLSAANDASRGGDVIAKVITTMESIEAAS----GKI
ymoll0001_9480   SAALEQISATVAQSASAARQANTAVLSAANDASRGGEVIAKVITTMESIEAAS----GKI
yberc0001_9790   SAALEQISSTVAQSASAARQANTAVLSAANDASRGGEVIAKVITTMESIEAAS----GKI
yruck0001_28810  AASMDQLTSTVKQNADNASHANQLAIDSSAVAVKGSTVVGQVISTMGSINHSS----KKI
yrohd0001_31660  AASMDELTSTVKQNADNASHANQLAIDASTVAVKGSTVVKQVVNTMGSINSSS----KKI
yaldo0001_35590  AASMDELTSTVKQNADNASHANQLAIDASTVAVKGSTVVKQVVDTMGSINHSS----KKI
ykris0001_37060  AASMDELTSTVKQNADNASHANKLAMDASSVAVKGSTVVKKVVDTMGSINHSS----KKI
yinte0001_37560  AASMDELTSTVKQNADNASHANQLARDASTVAVKGGKVVKQVVDTMGSINHSS----KKI
yfred0001_32450  AASMDELTSTVKQNADNASHANQLAMDASTVAVKGSTVVKQVVDTMGSINHSS----KKI
yente0001X_300   AASMDELTSTVKQNADNASHANQLAMDASTVAVKGSTVVKKVVDTMGSINHSS----KKI
ymoll0001_35290  AASMDELTSTVKQNADNASHANQLAMDASAVAVKGSTVVKQVVDTMGSINHSS----KKI
yberc0001_34000  AASMDELTSTVKQNADNASHANQLAMDASAVAVKGSTVVKKVVDTMGSINHSS----KKI
ykris0001_22290  ASSMEQLTSTIKNTADNTHQATSIANKASETAKHSGEVMGSVTQKMRGIRDSS----QRM
yinte0001_3170   ASSMEQLGSTVQQNADNAGQASTLANQATLEAQQGGVIVSGVIATMTKITSSS----HKI
yruck0001_31650  AASMEQLSATVKNNSDSANAATSLVKRASNSAVNGGEITRKMVITMSDIADSS----RKI
ymoll0001_7110   ASSMEEFTSTVKQNADNARQANILATAASEVAIKGGSMVSQVVDTMGSIHSSS----KKI
yruck0001_28370  AASMEQLNATVKQNADNAHHANQLATEASDTAEQGGKLVNDVVRTMGDISSSS----KRI
ypseu0001X_4346  AASMEQLNATVKQNTDNAHHANQLATEASQTAQQGGKLVNDVVRTMNDISGSS----KRI
ypest0001X_4370  AASMEQLNATVKQNTDNAHHANQLATEASQTAQQGGKLVNDVVRTMNDISGSS----KRI
yrohd0001_31240  AASMEQLNATVKQNADNAHHANQLATEASTTAQQGGKLVSDVVRTMNDISGSS----KRI
yaldo0001_35130  AASMEQLNATVKQNADNAHHANQLATEASDTAQQGGKLVNDVVRTMNDISGSS----KRI
yinte0001_36970  AASMEQLNATVKQNADNAHHANQLATEASDTAQQGGKLVSDVVRTMNDISGSS----KRI
ykris0001_39870  AASMEQLNATVKQNADNAHHANQLATEASQTAQQGGKLVNDVVRTMNDISGSS----KRI
ymoll0001_34760  AASMEQLNATVKQNADNAHHANQLATEASQTAQQGGKLVSDVVRTMNDISGSS----KRI
yfred0001_31920  AASMEQLNATVKQNADNAHHANQLATEASQTAQQGGKLVNDVVRTMNDISGSS----KRI
yberc0001_33520  AASMEQLNATVKQNADNAHHANQLATEASQTAQQGGKLVSDVVRTMNDISGSS----KRI
yente0001X_760   AASMEQLNATVKQNADNAHHANQLATEASQTAQQGGKLVNDVVRTMNDISGSS----KRI
yruck0001_18270  AASMEQLTATVKHNADNAHHASQLAATASSTAKKGGELVADVVHTMADISASS----KKI
yrohd0001_33070  AASMEQLTATVKHNASNAHHASQLADNARATAKKGGVLVADVVNTMNEISASS----RKI
yfred0001_34740  AASMEQLTATVKHNADNAHHASQLAANASATAKKGGVLVADVVNTMNEISASS----RKI
yaldo0001_22600  AASMEQLTATVKHNADNAHHASQLAASASATAKKGGLLVADVVNTMNEISASS----RKI
yinte0001_40830  AASMEQLTATVKHNADNAHHASQLAANASATAKKGGVLVADVVNTMNEISASS----RKI
ymoll0001_38830  AASMEQLTATVKHNADNAHHASQLAANASITAKKGGALVADVVHTMDEISASS----RKI
yberc0001_21820  AASMEQLTATVKHNADNAHHASQLAASASITAKKGGALVADVVHTMDEISASS----RKI
ykris0001_22260  AASMEQLTATVKHNADNAHHASQLAANASITAKKGGALVADVVHTMDEISASS----RKI
yente0001X_1297  AASMEQLTATVKHNADNAHHASQLAANASITAKKGGALVADVVHTMDEISASS----RKI
yruck0001_11920  AASMEQLTATVKQNAENARQASQLALSASETAQKGGKVVANVVQTMHDIAGSS----QKI
ypseu0001X_2707  AASMEQLTATVKQNAENARQASQLALSASETAQKGGKVVADVVQTMHEIAGSS----QKI
ypest0001X_1916  AASMEQLTATVKQNAENARQASQLALSASETAQKGGKVVADVVQTMHEIAGSS----QKI
yrohd0001_33620  AASMEQLTATVKQNAENARQASQLALSASETAQKGGKVVANVVQTMHDIAGSS----QKI
yaldo0001_15230  AASMEQLTATVKQNAENARQASQLALSASETAQKGGKVVANVVQTMHDIAGSS----QKI
ykris0001_37850  AASMEQLTATVKQNAENARQASQLALSASETAQKGGKVVANVVQTMHEIAGSS----QKI
yente0001X_1721  AASMEQLTATVKQNAENARQASQLALSASETAQKGGKVVANVVQTMHDIAGSS----QKI
ymoll0001_37090  AASMEQLTATVKQNAENARQASQLALSASETAQKGGKVVANVVQTMHEIAGSS----QKI
yberc0001_15260  AASMEQLTATVKQNAENARQASQLALSASETAQKGGKVVANVVQTMHEIAGSS----QKI
yinte0001_40500  AASMEQLTATVKQNAENARQASQLALSASETAQKGGKVVANVVQTMHEIAGSS----QKI
yfred0001_14080  AASMEQLTATVKQNAENARQASQLALSASETAQKGGKVVANVVQTMHEIAGSS----QKI
yruck0001_11930  AASMEQLTATVKQNADNARQATLLAQEASATAAKGGMLAGDVVNTMHEIAASS----HKI
yaldo0001_6760   ------------------------------------------------------------
ypest0001X_1918  ------------------------------------------------------------
ypest0001X_1917  AASMEQLTATVKQNADNAHQATVLAQEASGTAAKGGELTASVVTTMHAIATSS----QKI
ypseu0001X_2706  AASMEQLTATVKQNADNAHQATVLAQEASGTAAKGGELTASVVTTMHAIATSS----QKI
yrohd0001_33630  AASMEQLTATVKQNADNARQATLLAKEASGTAAKGGELTSSVVTTMHDIATSS----QKI
yaldo0001_15240  AASMEQLTATVKQNADNARQATLLAKDASGTAARGGELTSSVVTTMHDIATSS----QKI
ymoll0001_14920  AASMEQLTATVKQNADNARQATQLAQDASGTAAKGGELTSSVVTTMHDIATSS----KKI
yberc0001_15270  AASMEQLTATVKQNADNARQATQLAQDASGTAAKGGELTRGVVTTMHDIATSS----KKI
yfred0001_14090  AASMEQLTATVKQNADNARQATLLAQDASGTAAKGGALAGSVVTTMHDIATSS----QKI
yinte0001_15900  AASMEQLTATVKQNADNARQATLLAKDASGTAAKGGELAGSVVTTMHDIATSS----QKI
ykris0001_37840  AASMEQLTATVKQNADNARQATQLAQEASGTAAKGGELTGSVVKTMHDIATSS----QKI
yente0001X_1722  AASMEQLTATVKQNADNARQATQLAQDASGTAAKGGELAGSVVTTMHDIATSS----QKI
yruck0001_10900  AASMEQLTATVKQNSENAHHASQLAANASGKASQGGDIVSDVVSTMDKISMSS----MKI
yaldo0001_6770   ------------------------------------------------------------
yaldo0001_15110  AASMEQLTATVKQNAENAHHASQLAANASGKAVQGGDIVNDVISTMDKISLSS----MKI
yinte0001_40380  AASMEQLTATVKQNAENAHHASQLAADASGKAAQGGDIVNDVVSTMDKISLSS----MKI
ymoll0001_13500  AASMEQLTATVKQNAENAHHASQLAANASAKAAQGGDIVDDVVNTMDKISLSS----MKI
yberc0001_14860  AASMEQLTATVKQNAENAHHASQLAANASAKAAQGGDIVDDVVNTMDKISLSS----MKI
yrohd0001_33510  AASMEQLTATVKQNAENAHHASQLAANASGKAAQGGDIVNDVVSTMDKISLSS----MKI
yfred0001_13960  AASMEQLTATVKQNAENAHHASQLAANASGKAAQGGDIVNDVVSTMDKISLSS----MKI
yente0001X_1708  AASMEQLTATVKQNAENAHHASQLAANASGKAAQGGDIVNDVVSTMDKISLSS----MKI
ykris0001_38040  AASMEQLTATVKQNAENAHHASQLAANASGKAAQGGDIVNDVVDTMDKISLSS----MKI
yrohd0001_6540   AASMEQLNATVKQNAENAHQASKLAENASITAQNGGKIVNNVVETMSSITESS----RRI
yente0001X_6250  AASMEQLNATVKQNAENAHQASKLAENASTTAQNGGRIVNDVVATMSSITESS----RRI
yruck0001_10760  ---------------------------MSVLARKNGTNIADITNNIVAINQSS----EKI
ypest0001X_1900  AASMEQLTATVKQNAENAHHASQLAANASGKAAQGGDIVSDVVSTMDKISLSS----MKI
ypseu0001X_2718  AASMEQLTATVKQNAENAHHASQLAANASGKAAQGGDIVSDVVSTMDKISLSS----MKI
yaldo0001_37010  ------------------------------------------------------------
ypest0001X_1899  ------------------------------------------------------------
yfred0001_6650   AASMEQLNATVKQNAENAHQASKLAENASTTAQNGGRIVNDVVATMSSITDSS----RRI
yinte0001_6950   AASMEQLNATVKQNAENAHQASKLAENASATAKNGGRIVNDVVATMSSITESS----RRI
ymoll0001_37360  AASMEQLNATVKQNAENAHQASKLADNASVTAQNGGRIVNDVVATMSSITDSS----RRI
yberc0001_8160   AASMEQLNATVKQNAENAHQASKLADNASVTAQNGGRIVNDVVATMSSITESS----RRI
                                                                             


                        730       740       750       760       770       780
                 =========+=========+=========+=========+=========+=========+
ykris0001_41300  --------------TNEVIIHNS-----------------LSMPSGGG------------
yrohd0001_20110  --VKSVVDTSKLSFHDFIALYGP-------------------------------------
yaldo0001_2440   SNVLANIPLQPWQ-MAVLLIDDMQTNRDITGMMLQQLGHQVTLAESGESALSLGRTQRFD
ypseu0001X_4220  -EVLKSLPLQPWQ-MHILLVDDSETNRDITGMMLQQLGHQVTLADSGTTALAIGRQHRFD
ypest0001X_4540  ---LKSLPLQPWQ-MHILLVDDSETNRDITGMMLQQLGHQVTRADSGTTALAIGRQHRFD
yinte0001_2660   --MPTYNPQKGWH-VSVAACDHA------------------------------GSLAGFS
yberc0001_2350   HEIQQKLPLQPWH-MSILLVDDAETNRDITGMMLRQLGHQVTLAESGEVALHLGQTQRFD
ymoll0001_1840   QEISQNLPLQPWH-MSILLVDDAETNRDITGMMLQQLGHQVTLAESGEVALHLGQSQRFD
ykris0001_2490   PEIRHTLPLQPWC-MSILLVDDAETNRDITGMMLRQLGHNVTLAESGEVALRIGQNQHFD
yinte0001_2650   PQIPHTLPLQPWH-MSILLVDDAETNRDITGMMLRQLGHQVTLAESGEIALRIGQTQHFD
yrohd0001_2860   QVIKQHLPVQPWQ-MNILLVDDAETNRDITGMMLRQLGHQVTLAESGETALHIGLAQRFD
yfred0001_38320  QETKQNLPLQPWQ-MAILLVDDAETNRDIVGMMLQELGHQVTLAESGEVALSIGQTQRFD
yinte0001_26450  -----------WK-YN--------------------------------------FTSGLS
yberc0001_7120   ------------------------------------------------------------
ypseu0001X_3409  SEIITLIESVAFQ-TNILALNAA-----------------IEAAHAGE------HGRGFS
ypest0001X_1004  SEIITLIESVAFQ-TNILALNAA-----------------IEAAHAGE------HGRGFS
yente0001X_9160  AEIITLIESVAFQ-TNILALNAA-----------------IEAAHAGQ------HGRGFS
ykris0001_6100   AEIITLIESVAFQ-TNILALNAA-----------------IEAAHAGE------HGRGFS
yfred0001_43450  AEIITLIESVAFQ-TNILALNAA-----------------IEAAHAGE------HGRGFS
ymoll0001_7580   TEIITLIESVAFQ-THILALNAA-----------------IEAAHAGE------HGRGFS
yinte0001_8280   TAIITLIESVAFQ-TNILALNAA-----------------IEAAHVGE------HGRGFS
ykris0001_6110   --------------PEFIALKES-------------------------------------
yrohd0001_35950  GTIVEAIRKISSQ-TNLLAINAS-----------------IEAAHAGE------HGRGFS
ypseu0001X_2873  GSIVEAIRKISSQ-TNLLAINAS-----------------IEAAHAGE------HGRGFS
ypest0001X_2787  GSIVEAIRKISSQ-TNLLAINAS-----------------IEAAHAGE------HGRGFS
ymoll0001_11800  GTMVEAIRKISSQ-TNLLAINAS-----------------IEAAHAGE------HGKGFS
yfred0001_8890   GTIVEAIRKISSQ-TNLLAINAS-----------------IEAAHAGE------HGKGFS
ykris0001_12740  GTIVEAIRKISSQ-TNLLAINAS-----------------IEAAHAGE------HGKGFS
yente0001X_1434  GTIVEAIRKISSQ-TNLLAINAS-----------------IEAAHAGE------HGKGFS
yrohd0001_31880  ------------------------------------------------------------
yrohd0001_15520  VDIIAVIEGIAFQ-TNILALNAA-----------------VEAARAGT------EGKGFA
yfred0001_15260  VDIIAVIEGIAFQ-TNILALNAA-----------------VEAARAGN------EGKGFA
yinte0001_16590  VDIIAVIEGIAFQ-TNILALNAA-----------------VEAARAGT------EGKGFA
ymoll0001_41560  VDIIAVIEGIAFQ-TNILALNAA-----------------VEAARAGN------EGKGFA
yberc0001_16020  VDIIAVIEGIAFQ-TNILALNAA-----------------VEAARAGT------EGKGFA
yberc0001_5770   ADINGVIDSIANQ-TNILALNAA-----------------VEAARAGE------QGRGFA
ymoll0001_5080   ADINGVIDSIANQ-TNILALNAA-----------------VEAARAGE------QGRGFA
yaldo0001_37020  ------------------------------------------------------------
ypseu0001X_2733  VDIISVIEGIAFQ-TNILALNAA-----------------VEAARAGE------QGRGFA
ypest0001X_1885  VDIISVIEGIAFQ-TNILALNAA-----------------VEAARAGE------QGRGFA
yrohd0001_12010  VDIISVIEGIAFQ-TNILALNAA-----------------VEAARAGE------QGRGFA
yfred0001_12940  VDIISVIEGIAFQ-TNILALNAA-----------------VEAARAGE------QGRGFA
yinte0001_14220  VDIISVIEGIAFQ-TNILALNAA-----------------VEAARAGE------QGRGFA
ymoll0001_14640  VDIISVIEGIAFQ-TNILALNAA-----------------VEAARAGE------QGRGFA
yberc0001_15000  VDIISVIEGIAFQ-TNILALNAA-----------------VEAARAGE------QGRGFA
ykris0001_42780  VDIISVIEGIAFQ-TNILALNAA-----------------VEAARAGE------QGRGFA
yente0001X_2568  VDIISVIEGIAFQ-TNILALNAA-----------------VEAARAGE------QGRGFA
yberc0001_5850   SNIIKVIDGIAGQ-TNILALNAA-----------------VEAARAGE------QGRGFA
ymoll0001_5160   SNIIKVIDGIASQ-TNILALNAA-----------------VEAARAGE------QGRGFA
yberc0001_6170   AGITTVIDGIASQ-TNILALNAA-----------------VEAARAGE------QGRGFA
yaldo0001_6750   ------------------------------------------------------------
yfred0001_39550  ------------------------------------------------------------
yrohd0001_27170  SDIIAVIDGIAFQ-TNILALNAA-----------------VEAARAGE------QGRGFA
ykris0001_32930  SDIIAVIDGIAFQ-TNILALNAA-----------------VEAARAGE------QGRGFA
yente0001X_3909  SDIIAVIDGIAFQ-TNILALNAA-----------------VEAARAGE------QGRGFA
ymoll0001_40600  SDIIAVIDGIAFQ-TNILALNAA-----------------VEAARAGE------QGRGFA
yberc0001_29090  SDIIAVIDGIAFQ-TNILALNAA-----------------VEAARAGE------QGRGFA
ypseu0001X_1449  GDITSVIDGIAFQ-TNILALNAA-----------------VEAARAGE------QGRGFA
ypest0001X_1447  GDITSVIDGIAFQ-TNILALNAA-----------------VEAARAGE------QGRGFA
yfred0001_36240  -------PTVALR-----------------------------------------------
yinte0001_10140  GDITSVIDGIAFQ-TNILALNAA-----------------VEAARAGE------QGRGFA
ykris0001_10260  GDITSVIDGIAFQ-TNILALNAA-----------------VEAARAGE------QGRGFA
yente0001X_2906  GDITSVIDGIAFQ-TNILALNAA-----------------VEAARAGE------QGRGFA
yfred0001_36230  GDITSVIDGIAFQ-TNILALNAA-----------------VEAARAGE------QGRGFA
ymoll0001_9480   GDITSVIDGIAFQ-TNILALNAA-----------------VEAARAGK------QGRGFA
yberc0001_9790   GDITSVIDGIAFQ-TNILALNAA-----------------VEAARAGE------QGRGFA
yruck0001_28810  VDIISVIDSIAFQ-TNILALNAA-----------------VEAARAGE------QGRGFA
yrohd0001_31660  VDIISVIDSIAFQ-TNILALNAA-----------------VEAARAGE------QGRGFA
yaldo0001_35590  VDIISVIDSIAFQ-TNILALNAA-----------------VEAARAGE------QGRGFA
ykris0001_37060  VDIISVIDSIAFQ-TNILALNAA-----------------VEAARAGE------QGRGFA
yinte0001_37560  VDIISVIDSIAFQ-TNILALNAA-----------------VEAARAGE------QGRGFA
yfred0001_32450  VDIISVIDSIAFQ-TNILALNAA-----------------VEAARAGE------QGRGFA
yente0001X_300   VDIISVIDSIAFQ-TNILALNAA-----------------VEAARAGE------QGRGFA
ymoll0001_35290  VDIISVIDSIAFQ-TNILALNAA-----------------VEAARAGE------QGRGFA
yberc0001_34000  VDIISVIDSIAFQ-TNILALNAA-----------------VEAARAGE------QGRGFA
ykris0001_22290  SEIIGVIDGIAFQ-TNILALNAA-----------------VEAARAGE------QGRGFA
yinte0001_3170   VDIIGVINSIAFQ-TNILALNAA-----------------VEAARAGE------QGRGFA
yruck0001_31650  GDITSVIDGIAFQ-TNILALNAA-----------------VEAARAGE------QGRGFA
ymoll0001_7110   VDIIGVIDSIAFQ-TNILALNAA-----------------VEAARAGE------QGRGFA
yruck0001_28370  SEITSVINSIAFQ-TNILALNAA-----------------VEAARAGE------QGRGFA
ypseu0001X_4346  SEITSVINSIAFQ-TNILALNAA-----------------VEAARAGE------QGRGFA
ypest0001X_4370  SEITSVINSIAFQ-TNILALNAA-----------------VEAARAGE------QGRGFA
yrohd0001_31240  SEITSVINSIAFQ-TNILALNAA-----------------VEAARAGE------QGRGFA
yaldo0001_35130  AEITSVINSIAFQ-TNILALNAA-----------------VEAARAGE------QGRGFA
yinte0001_36970  SEITSVINSIAFQ-TNILALNAA-----------------VEAARAGE------QGRGFA
ykris0001_39870  SEITSVINSIAFQ-TNILALNAA-----------------VEAARAGE------QGRGFA
ymoll0001_34760  SEITSVINSIAFQ-TNILALNAA-----------------VEAARAGE------QGRGFA
yfred0001_31920  SEITSVINSIAFQ-TNILALNAA-----------------VEAARAGE------QGRGFA
yberc0001_33520  SEITSVINSIAFQ-TNILALNAA-----------------VEAARAGE------QGRGFA
yente0001X_760   SEITSVINSIAFQ-TNILALNAA-----------------VEAARAGE------QGRGFA
yruck0001_18270  AEITTVINSIAFQ-TNILALNAA-----------------VEAARAGE------QGRGFA
yrohd0001_33070  ADITTVINSIAFQ-TNILALNAA-----------------VEAARAGE------QGRGFA
yfred0001_34740  ADITTVINSIAFQ-TNILALNAA-----------------VEAARAGE------QGRGFA
yaldo0001_22600  ADITTVINSIAFQ-TNILALNAA-----------------VEAARAGE------QGRGFA
yinte0001_40830  ADITTVINSIAFQ-TNILALNAA-----------------VEAARAGE------QGRGFA
ymoll0001_38830  ADITTVINSIAFQ-TNILALNAA-----------------VEAARAGE------QGRGFA
yberc0001_21820  ADITTVINSIAFQ-TNILALNAA-----------------VEAARAGE------QGRGFA
ykris0001_22260  AEITTVINSIAFQ-TNILALNAA-----------------VEAARAGE------QGRGFA
yente0001X_1297  AEITTVINSIAFQ-TNILALNAA-----------------VEAARAGE------QGRGFA
yruck0001_11920  ADITSVIDGIAFQ-TNILALNAA-----------------VEAARAGE------QGRGFA
ypseu0001X_2707  ADITSVIDGIAFQ-TNILALNAA-----------------VEAARAGE------QGRGFA
ypest0001X_1916  ADITSVIDGIAFQ-TNILALNAA-----------------VEAARAGE------QGRGFA
yrohd0001_33620  ADITSVIDGIAFQ-TNILALNAA-----------------VEAARAGE------QGRGFA
yaldo0001_15230  ADITSVIDGIAFQ-TNILALNAA-----------------VEAARAGE------QGRGFA
ykris0001_37850  ADITSVIDGIAFQ-TNILALNAA-----------------VEAARAGE------QGRGFA
yente0001X_1721  ADITSVIDGIAFQ-TNILALNAA-----------------VEAARAGE------QGRGFA
ymoll0001_37090  ADITSVIDGIAFQ-TNILALNAA-----------------VEAARAGE------QGRGFA
yberc0001_15260  ADITSVIDGIAFQ-TNILALNAA-----------------VEAARAGE------QGRGFA
yinte0001_40500  ADITSVIDGIAFQ-TNILALNAA-----------------VEAARAGE------QGRGFA
yfred0001_14080  ADITSVIDGIAFQ-TNILALNAA-----------------VEAARAGE------QGRGFA
yruck0001_11930  GAITSVIDGIAFQ-TNILALNAA-----------------VEAARAGE------QGRGFA
yaldo0001_6760   -------------------LNAA-----------------VEAARAGE------QGRGFA
ypest0001X_1918  ------------------------------------------------------------
ypest0001X_1917  GAITSVIDGIAFQ-TNILALNAA-----------------VEAARAGE------QGRGFA
ypseu0001X_2706  GAITSVIDGIAFQ-TNILALNAA-----------------VEAARAGE------QGRGFA
yrohd0001_33630  GAITSVIDGIAFQ-TNILALNAA-----------------VEAARAGE------QGRGFA
yaldo0001_15240  GAITSVIDGIAFQ-TNILALNAA-----------------VEAARAGE------QGRGFA
ymoll0001_14920  GAITSVIDGIAFQ-TNILALNAA-----------------VEAARAGE------QGRGFA
yberc0001_15270  GAITSVIDGIAFQ-TNILALNAA-----------------VEAARAGE------QGRGFA
yfred0001_14090  GAITSVIDGIAFQ-TNILALNAA-----------------VEAARAGE------QGRGFA
yinte0001_15900  GAITSVIDGIAFQ-TNILALNAA-----------------VEAARAGE------QGRGFA
ykris0001_37840  GAITSVIDGIAFQ-TNILALNAA-----------------VEAARAGE------QGRGFA
yente0001X_1722  GAITSVIDGIAFQ-TNILALNAA-----------------VEAARAGE------QGRGFA
yruck0001_10900  ADITNVINSIAFQ-TNILALNAA-----------------VEAARAGE------QGRGFA
yaldo0001_6770   ------------------------------------------------------------
yaldo0001_15110  AEITNVINSIAFQ-TNILALNAA-----------------VEAARAGE------QGRGFA
yinte0001_40380  AEITNVINSIAFQ-TNILALNAA-----------------VEAARAGE------QGRGFA
ymoll0001_13500  AEITNVINSIAFQ-TNILALNAA-----------------VEAARAGE------QGRGFA
yberc0001_14860  AEITNVINSIAFQ-TNILALNAA-----------------VEAARAGE------QGRGFA
yrohd0001_33510  AEITNVINSIAFQ-TNILALNAA-----------------VEAARAGE------QGRGFA
yfred0001_13960  AEITNVINSIAFQ-TNILALNAA-----------------VEAARAGE------QGRGFA
yente0001X_1708  AEITNVINSIAFQ-TNILALNAA-----------------VEAARAGE------QGRGFA
ykris0001_38040  AEITNVINSIAFQ-TNILALNAA-----------------VEAARAGE------QGRGFA
yrohd0001_6540   ADIIGVINSIAFQ-TNILALNAA-----------------VEAARAGE------QGRGFA
yente0001X_6250  ADIIGVINSIAFQ-TNILALNAA-----------------VEAARAGE------QGRGFA
yruck0001_10760  SNIIGVIDSISFQ-SNILALNAA-----------------VEAARAGE------AGKGFA
ypest0001X_1900  AEITNVINSIAFQ-TNILALNAA-----------------VEAARAGE------QGRGFA
ypseu0001X_2718  AEITNVINSIAFQ-TNILALNAA-----------------VEAARAGE------QGRGFA
yaldo0001_37010  ----------------------------------------------------------FA
ypest0001X_1899  ------------------------------------------------------------
yfred0001_6650   ADIIGVINSIAFQ-TNILALNAA-----------------VEAARAGE------QGRGFA
yinte0001_6950   ADIIGVINSIAFQ-TNILALNAA-----------------VEAARAGE------QGRGFA
ymoll0001_37360  ADIIGVINSIAFQ-TNILALNAA-----------------VEAARAGE------QGRGFA
yberc0001_8160   ADIIGVINSIAFQ-TNILALNAA-----------------VEAARAGE------QGRGFA
                   ###########                                         ######


                        790       800       810       820       830       840
                 =========+=========+=========+=========+=========+=========+
ykris0001_41300  -------------------------NVD--------------------------------
yrohd0001_20110  ----------------------------------------VLKEAFGDDKTKIKAFLGDA
yaldo0001_2440   LVLMDIRMPGMDGLLATQCWRNDTKNVDKHCMIMALSANASPDEKTRAYESGMGHYLSKP
ypseu0001X_4220  LVLMDIRMPVLDGLATTARWRHDPANIDSHCMITALSANASPDEQIKTSQAGMNHYLSKP
ypest0001X_4540  LVLMDIRMPVLDGLATTARWRHDPANIDSHCMITALSANASPDEQIKTSQAGMNHYLSKP
yinte0001_2660   LKLNE--------------------LVTDNQPVEQRDINLWLDKSGELL----------P
yberc0001_2350   LVLMDIRMPGMDGLTTTRCWREDIINRDNRCMITALSANTNPDEKIKAYQAGMNHYLSKP
ymoll0001_1840   LVLMDIRMPGMDGLTTTRCWREDMINCDNRCMITALSANTNPDEKIKAYQAGMNHYLSKP
ykris0001_2490   LVLMDIRMPDMDGLMTTQRWRNDAINLDRRCMITALSANTNPDEKIRAYHAGMNHYISKP
yinte0001_2650   LVLMDIRMPGMDGLATTRYWRDDVNNQDNRCMITALSANTNPEEKIRASQAGMNHYLSKP
yrohd0001_2860   LVLMDIRMPNMDGLTTTRHWRNDAMNRDNRCMITALSANASSDEKIKAYQAGINNYLPKP
yfred0001_38320  LVLMDIRMPGMDGLTTTRYWRNDVANRDNCGMITALSANANPDEKIKAYQAGMNHYLSKP
yinte0001_26450  LTNNKIK-----------------------------------------------------
yberc0001_7120   -------------------------ALSHQHFDKEVAIN---------------------
ypseu0001X_3409  VVAREVG------------------TLAHQSGHSAQNIKRLIQHSANSVSTGASLVNRSG
ypest0001X_1004  VVAREVG------------------TLSHQSGHSAQNIKRLIQHSANSVSTGASLVNRSG
yente0001X_9160  VVAREVG------------------ILAHQSGHSALNIKRLIGNSSKSISAGANLVGRSG
ykris0001_6100   VVAREVG------------------MLAHQSGHSALNIKRLIENSSTYISAGAGLVGRSG
yfred0001_43450  VVAREVG------------------ILAHQSGNSALNIKRLIDNSAKYISAGAGLVGRSG
ymoll0001_7580   VVAREVG------------------MLAHKSGNSALNIKQLIHNSSSSISAGSSLVARSG
yinte0001_8280   VVAREVG------------------LLAHQSGNSALNIKRLIHNSSSSISTGRGLVARSG
ykris0001_6110   ------------------------------------------------------------
yrohd0001_35950  VVASEVR------------------LLAEQSRKAAIEIEQM----TKTIQSG---VTAAI
ypseu0001X_2873  VVASEVR------------------LLADQSRKAASEIEQM----TKTIQNG---VTAAI
ypest0001X_2787  VVASEVR------------------LLADQSRKAASEIEQM----TKTIQNG---VTAAI
ymoll0001_11800  VVASEVR------------------LLAEHSRKAATEIEQM----TKTIQRG---VMAAM
yfred0001_8890   VVASEVR------------------MLAERSRKAATEIEHM----TKTIQNG---VMAAI
ykris0001_12740  VVASEVR------------------ILAESSRKAATEIEHM----TKTIQNG---VMAAI
yente0001X_1434  VVASEVR------------------ILAESSRKAATEIEHM----TKTIQNG---VMAAI
yrohd0001_31880  -LQDKIR-----------------------------------------------------
yrohd0001_15520  VVASEVR------------------TLAQKSANAAKEIKQLIVGTVEKITAGSERADHAS
yfred0001_15260  VVASEVR------------------TLAQKSANAAKEIKQLIIGTVEKITAGSERADHAS
yinte0001_16590  VVASEVR------------------TLAQKSANAAKEIKQLIVGTVEKINAGSERADHAS
ymoll0001_41560  VVASEVR------------------TLAQKSANAAKEIKLLIVGTVEKINAGSERADHAS
yberc0001_16020  VVASEVR------------------TLAQKSANAAKEIKLLIVGTVEKINAGSERADHAS
yberc0001_5770   VVAGEVR------------------SLAKRSADAAKEISQLITTCVANMNTGSQQVDQAG
ymoll0001_5080   VVAGEVR------------------NLAKRSADAAKEINQLITVCVANMSTGSQQVDVAG
yaldo0001_37020  ------------------------------------------------MRDGSTQVQHAG
ypseu0001X_2733  VVASEVR------------------HLAQRSSTAAKEIKHLIETSVERVRDGSTLVQNAG
ypest0001X_1885  VVASEVR------------------HLAQRSSTAAKEIKHLIETSVERVRDGSTLVQSAG
yrohd0001_12010  VVASEVR------------------HLAQRSSTAAKEIKQLIETSVDRVRDGSALVQNAG
yfred0001_12940  VVASEVR------------------HLAQRSSTAAKEIKHLIETSVERVRDGSTLVQNAG
yinte0001_14220  VVASEVR------------------HLAQRSSTAAKEIKQLIETSVERVRDGSALVQNAG
ymoll0001_14640  VVASEVR------------------HLAQRSSTAAKEIKHLIETSVERVRDGSALVQNAG
yberc0001_15000  VVASEVR------------------HLAQRSSTAAKEIKHLIETSVERVRDGSALVQNAG
ykris0001_42780  VVASEVR------------------HLAQRSSTAAKEIKHLIETSVERVRDGSKLVQNAG
yente0001X_2568  VVASEVR------------------HLAQRSSTAAKEIKHLIETSVERIRDGSALVQNAG
yberc0001_5850   VVATEVR------------------NLAQRSADAAKEIRGLIEHSVHDTQTGSKRVDSAK
ymoll0001_5160   VVATEVR------------------NLAQRSADAAKEIRGLIEHSVHDTQAGSKRVDSAK
yberc0001_6170   VVASEVR------------------NLASRSSTSAREISVLINDAIDNISRGSQLVTNAG
yaldo0001_6750   ------------------------------------------------------------
yfred0001_39550  ------------------------------------------------------------
yrohd0001_27170  VVAGEVR------------------NLAQRSAQSAKEIKDLIVESQLRVREGADMAESAG
ykris0001_32930  VVAGEVR------------------NLAQRSAQSAKEIKDLIIESQTRVREGADMAEFAG
yente0001X_3909  VVAGEVR------------------NLAQRSAQSAKEIKDLIVESQNRVREGADMAESAG
ymoll0001_40600  VVAGEVR------------------NLAQRSAQSAKEIKDLIVESQNRVREGADMAESAG
yberc0001_29090  VVAGEVR------------------NLAQRSAQSAKEIKDLIVESQNRVREGADMAESAG
ypseu0001X_1449  VVAGEVR------------------ILAQRSAQAAKEIKALIESTVSSVASGSSQVRQAS
ypest0001X_1447  VVAGEVR------------------ILAQRSAQAAKEIKALIESTVSSVASGSSQVRQAS
yfred0001_36240  ------------------------------------------------------QVAAAG
yinte0001_10140  VVAGEVR------------------TLAQRSAQAAKEIKTLIESTVSSVASGSGQVRQAS
ykris0001_10260  VVAGEVR------------------TLAQRSAQAAKEIKTLIDSTVSSVASGSGQVRQAS
yente0001X_2906  VVAGEVR------------------TLAQRSAQAAKEIKTLIDSTVSSVASGSGQVRQAS
yfred0001_36230  VVAGEVR------------------TLAQRSAQAAKEIKTLIESTVSSVASGSGQVRQAS
ymoll0001_9480   VVAGEVR------------------TLAQRSAQAAKEIKTLIESTVSSVSSGSGQVRQAS
yberc0001_9790   VVAGEVR------------------TLAQRSAQAAKEIKTLIESTVSSVSSGSGQVRLAS
yruck0001_28810  VVASEVR------------------SLAQRSATSAREIKKLIEDSVADIATGTDLVADAG
yrohd0001_31660  VVASEVR------------------NLAQRSATSAREIKKLIEDSVADIAIGTGLVAEAG
yaldo0001_35590  VVASEVR------------------NLAQRSATSAREIKKLIEDSVADIATGTRLVADAG
ykris0001_37060  VVASEVR------------------NLAQRSATSAREIKKLIEDSVSDITIGTRLVADAG
yinte0001_37560  VVASEVR------------------NLAQRSATSAREIKKLIEDSVADIAIGTDLVADAG
yfred0001_32450  VVASEVR------------------NLAQRSATSAREIKKLIEDSVADIAIGTGLVADAG
yente0001X_300   VVASEVR------------------NLAQRSATSAREIKKLIEDSVADIAIGTSLVADAG
ymoll0001_35290  VVASEVR------------------NLAQRSATSAREIKKLIEDSVSDIAIGTGLVADAG
yberc0001_34000  VVASEVR------------------NLAQRSATSAREIKKLIEDSVSDIAIGTGLVADAG
ykris0001_22290  VVASEVR------------------SLAQRSATAAKEIKTLIDNSVDKIQDGMRLVDSAE
yinte0001_3170   VVASEVR------------------SLAQRSAQAAKEIDVLINESVKNIKSGSEQVTRAG
yruck0001_31650  VVAGEVR------------------NLAQRSAQAAKEIKTLIDASVLRVDQGNDLVENVS
ymoll0001_7110   VVASEVR------------------NLAQRSASAAKEIKTLIGDSVDQVNIGTGLVDQTG
yruck0001_28370  VVASEVR------------------NLAQRSAQAAKEIEGLIGESVSQVNKGTSLVQNAG
ypseu0001X_4346  VVASEVR------------------NLAQRSAQAAKEIEGLIGESVSQVNAGTSLVKNAG
ypest0001X_4370  VVASEVR------------------NLAQRSAQAAKEIEGLIGESVSQVNAGTSLVKNAG
yrohd0001_31240  VVASEVR------------------NLAQRSAQAAKEIEGLIGESVSQVSAGTLLVQNAG
yaldo0001_35130  VVASEVR------------------NLAQRSAQAAKEIEGLIGESVSQVNSGTTLVQNAG
yinte0001_36970  VVASEVR------------------NLAQRSAQAAKEIEGLIGESVTQVNAGTTLVQNAG
ykris0001_39870  VVASEVR------------------NLAQRSAQAAKEIEGLIGESVSQVNAGTSLVQNAG
ymoll0001_34760  VVASEVR------------------NLAQRSAQAAKEIEGLIGESVSQVNAGTSLVQNAG
yfred0001_31920  VVASEVR------------------NLAQRSAQAAKEIEGLIGESVSQVNAGTSLVQNAG
yberc0001_33520  VVASEVR------------------NLAQRSAQAAKEIEGLIGESVSQVNAGTSLVQNAG
yente0001X_760   VVASEVR------------------NLAQRSAQAAKEIEGLIGESVSQVNAGTSLVQNAG
yruck0001_18270  VVASEVR------------------SLAQRSAQAAKEIDGLINESVSRVISGSAQVESAG
yrohd0001_33070  VVASEVR------------------NLAQRSAQAAKEIEGLISESVGRVSSGSALVESTG
yfred0001_34740  VVASEVR------------------NLAQRSAQAAKEIDSLINESVSRVSSGSALVESAG
yaldo0001_22600  VVASEVR------------------NLAQRSAQAAKEIDGLINESVNRVSSGSALVESAG
yinte0001_40830  VVASEVR------------------NLAQRSAQAAKEIDGLINESVSRVSSGSALVESAG
ymoll0001_38830  VVASEVR------------------NLAQRSAQAAKEIDGLITESVNRVKSGSALVESAG
yberc0001_21820  VVASEVR------------------NLAQRSAQAAKEIDGLITESVNRVKSGSALVESAG
ykris0001_22260  VVASEVR------------------NLAQRSAQAAKEIDNLITESVSRVSSGSALVESAG
yente0001X_1297  VVASEVR------------------NLAQRSAQAAKEIDSLITESVNRVSSGSALVESAG
yruck0001_11920  VVAGEVR------------------NLAQRSAQAAKEIKGLIEDSVNRVDMGSVLVESAG
ypseu0001X_2707  VVAGEVR------------------NLAQRSAQAAKEIKGLIDDSVNRVDLGSVLVESAG
ypest0001X_1916  VVAGEVR------------------NLAQRSAQAAKEIKGLIDDSVNRVDLGSVLVESAG
yrohd0001_33620  VVAGEVR------------------NLAQRSAQAAKEIKGLIEDSVNRVDMGSVLVESAG
yaldo0001_15230  VVAGEVR------------------NLAQRSAQAAKEIKGLIEDSVNRVDMGSVLVESAG
ykris0001_37850  VVAGEVR------------------NLAQRSAQAAKEIKGLIEDSVNRVDMGSVLVESAG
yente0001X_1721  VVAGEVR------------------NLAQRSAQAAKEIKGLIEDSVNRVDMGSVLVESAG
ymoll0001_37090  VVAGEVR------------------NLAQRSAQAAKEIKGLIEDSVNRVDMGSVLVESAG
yberc0001_15260  VVAGEVR------------------NLAQRSAQAAKEIKGLIEDSVNRVDMGSVLVESAG
yinte0001_40500  VVAGEVR------------------NLAQRSAQAAKEIKGLIEDSVSRVDMGSVLVESAG
yfred0001_14080  VVAGEVR------------------NLAQRSAQAAKEIKGLIEDSVSRVDMGSVLVESAG
yruck0001_11930  VVAGEVR------------------NLAQRSAQAAKEIKGLIDESVGRVRQGSTLVESSG
yaldo0001_6760   VVASEVR------------------NLAQRSAQAAKEIEGLISDRC----CGLMLAYDKL
ypest0001X_1918  ------------------------------------------------------------
ypest0001X_1917  VVAGEVR------------------NLA--------------------------------
ypseu0001X_2706  VVAGEVR------------------NLAQRSAQAAKEIKGLIDESVSRVRQGSTLVESAG
yrohd0001_33630  VVAGEVR------------------NLAQRSAQAAKEIKGLIDESVSRVRQGSTLVESAG
yaldo0001_15240  VVAGEVR------------------NLAQRSAQAAKEIKGLIDESVSRVRQGSTLVENAG
ymoll0001_14920  VVAGEVR------------------NLAQRSAQAAKEIKVLIDESVSRVSQGSTLVENAG
yberc0001_15270  VVAGEVR------------------NLAQRSAQAAKEIKVLIDESVSRVSQGSTLVENAG
yfred0001_14090  VVAGEVR------------------NLAQRSAQAAKEIKGLIEESVSRVRQGSTLVENAG
yinte0001_15900  VVAGEVR------------------NLAQRSAQAAKEIKGLIDESVSRVRQGSTLVENAG
ykris0001_37840  VVAGEVR------------------NLAQRSAQAAKEIKGLIDESVSRVRQGSTLVENAG
yente0001X_1722  VVAGEVR------------------NLAQRSAQAAKEIKGLIDESVSRVRQGSTLVENAG
yruck0001_10900  VVASEVR------------------HLAQRSADAAKEIESLIEASVDLVGDGSILVSDAG
yaldo0001_6770   ------------------------------------------------------------
yaldo0001_15110  VVASEVR------------------NLAQRSADAAKEIESLIEASVDLIGDGSILVSDAG
yinte0001_40380  VVASEVR------------------NLAQRSADAAKEIESLIEASVDLIGDGSILVSDAG
ymoll0001_13500  VVASEVR------------------NLAQRSADAAKEIELLIEASVDLIGDGSILVSDAG
yberc0001_14860  VVASEVR------------------NLAQRSADAAKEIELLIEASVDLIGDGSILVSDAG
yrohd0001_33510  VVASEVR------------------NLAQRSADAAKEIELLIEASVELIGDGSILVSDAG
yfred0001_13960  VVASEVR------------------NLAQRSADAAKEIESLIEASVDLIGDGSILVSDAG
yente0001X_1708  VVASEVR------------------NLAQRSADAAKEIESLIEVSVDLIGDGSILVSDAG
ykris0001_38040  VVASEVR------------------NLAQRSADAAKEIESLIEVSVDLIGDGSILVSDAG
yrohd0001_6540   VVASEVR------------------NLAQRSAQAAKEIEGLISESVSRVNIGSQQVSEAG
yente0001X_6250  VVASEVR------------------NLAQRSAQAAKEIEGLISESVSRVNMGSQQVSRAG
yruck0001_10760  VVASEVR------------------NLAQRSAVSAKEIKELIEDSVHKIKQGSDMAAHSS
ypest0001X_1900  VVASEVR------------------HLAQRRCRKRN------------------------
ypseu0001X_2718  VVASEVR------------------HLAQRSADAAKEIESLIEASVDLIGDGSILVSNAG
yaldo0001_37010  VVAGEVR------------------NLAQR------------------------------
ypest0001X_1899  -----------------------------RSADAAKEIESLIEASVDLIGDGSILVSNAG
yfred0001_6650   VVASEVR------------------NLAQRSAQAAKEIEGLISESVSRVNMGSKQVSEAG
yinte0001_6950   VVASEVR------------------NLAQRSAQAAKEIEGLISESVSRVNIGSQQVSEAG
ymoll0001_37360  VVASEVR------------------NLAQRSAQAAKEIEGLISESVSRVNIGSKQVSEAG
yberc0001_8160   VVASEVR------------------NLAQRSAQAAKEIEGLISESVSRVNIGSKQVSEAG
                 #######                   ##########################        


                        850       860       870       880       890       900
                 =========+=========+=========+=========+=========+=========+
ykris0001_41300  ------------------------------------------------------------
yrohd0001_20110  KEIYNLFQGGDLHNAGDLIQDYIDNSSVFYSVKSK-----------------------PF
yaldo0001_2440   VTLWQL-AGILDLTAQFQLERGLPLTPQISSPKPLLDLTNPQLNIELYRSLQLLMQKIKE
ypseu0001X_4220  VTLGQL-AEMLDLTAQFQLERGVDLSPQLSEPQPLLDLADSALSLKLYQSLQVLIQQAKD
ypest0001X_4540  VTLGQL-AEMLDLTAQFQLERGVDLSPQLSEPQPLLDLADSALSLKLYQSLQVLIQQAKD
yinte0001_2660   ISQQNI------------------PSHQLHEILRQLK--DRNLHNGWQQTPDYLVLRTQ-
yberc0001_2350   ITICQL-AEVLDLAAQFQLERDITLSPHISIPRPLLNVDDNLLRLKLSQSLKVLVQQASE
ymoll0001_1840   ITFCQL-AEVLDLAAQFQLERDISLSPHISIPRPLLNLDDDLLRLKLSQSLKVLVQQANE
ykris0001_2490   VTFDQL-AKVLDLTAQFQLERGIHLIPQITTPQPLLNLADGSLRLKVLKSIQDLLQQSRN
yinte0001_2650   VTFSQL-AEVLDLAAQFQLERGVTLSPQLSTPRPLLNLADTSLRLKLSQSLQLLVQQGRE
yrohd0001_2860   VSFCQL-AEVLDLTAQFQLERGITLSPQISIPKPLLNLEDEELRLKLSQSLQELLQQIRN
yfred0001_38320  VTFCQL-AEILDLAAQFQLERGINLTPQISAPKPLLDLADPSLHLKLAQSLQLLLQQARN
yinte0001_26450  ------------------------------------------------------------
yberc0001_7120   ------------------------------------------------------------
ypseu0001X_3409  ENLQAI----------------IDVVKKVTDLMAEITTASHYQSKGIEEMAAQVEMINGA
ypest0001X_1004  ENLQAI----------------IDVVKKVTDLMAEITTASHYQSKGIEEMAAQVEMINGA
yente0001X_9160  DNLRAI----------------IGSVIKVTDLMTEISAASQEQSKGIEDITAQVGMINEV
ykris0001_6100   DNLHAI----------------IDAVIKVTDLMAEISAASHEQSKGIEDITARVGMINEV
yfred0001_43450  DNLRAI----------------IDAVVKVTDLMAEISAASHEQSQGIEDISVRVGIINEV
ymoll0001_7580   DNLMEI----------------IDAVKKVTDLMAEISAASHDQSEGIENITQQVGVINEV
yinte0001_8280   DNLRAI----------------IDAVKKVTDLMAEISAASHDQSQGIEGITTQVGMINDV
ykris0001_6110   ---------------------------------------YQQLYQGLTELGQGLLKNNNI
yrohd0001_35950  KGMGTC----------------VQQAGGGVTLTQDAGDVINQVNIGMQDVVKLMAEFAQA
ypseu0001X_2873  KGMGTC----------------VEQAGGGVILTQDAGEVINLVNVGMQDVVKLMTTFSQV
ypest0001X_2787  KGMGTC----------------VEQAGGGVILTQDAGEVINLVNVGMQDVVKLMTTFSQV
ymoll0001_11800  KGMGTC----------------VEQAGGGVTLTQDAGEVINQVNIGMQDVVKLMTAFAQV
yfred0001_8890   KGMDVC----------------LEHAGGGVTLTQDAGEVINQVNIGMQDVVKLMAAFAQV
ykris0001_12740  KGMGVC----------------VEQAGGGVTLTQDAGEVIHQVNIGMQDVVKLMTAFSQV
yente0001X_1434  KGMGVC----------------VEQAGGGVTLTQDAGEVIHQVNIGMQDVVKLMTAFSQV
yrohd0001_31880  ------------------------------------------------------------
yrohd0001_15520  QAMGEI----------------VNSVGKVAHIIGEISTASQEQHIGIQEIGIAVEQMDHV
yfred0001_15260  QAMSEI----------------VDSVGKVAHIVGEISTASQEQHIGIQEIGIAVEQMDQV
yinte0001_16590  QAMGEI----------------VDSVGKVAHIVGEISTASHEQHIGIQEIGIAVEQMDQV
ymoll0001_41560  QAMGEI----------------VDSVGKVAHIVGEISTASQEQHIGIQEIGIAVEQMDQV
yberc0001_16020  QAMGEI----------------VDSVSKVAHIVGEISIASQEQHIGIQEIGIAVEQMDQV
yberc0001_5770   TVMKDI----------------VSSVTQVTDIMGEITSASDEQSAGINQIAQAVNEMDQV
ymoll0001_5080   TVMKDI----------------VNSVTQVTDIMGEITSASDEQSAGINQIAQAVNEMDQV
yaldo0001_37020  ATMDNI----------------VKQAGQVSTLISEISTSTHEQTQALGQINQSIGQLNQM
ypseu0001X_2733  ATMDNI----------------VKQASQVSTLISEISTSTHEQTQALGQIRQSISRLDQM
ypest0001X_1885  ATMDNI----------------VKQASQVSTLISEISTSTHEQTQALGQIRQSISRLDQM
yrohd0001_12010  ATMDNI----------------VKQAGQVSTLINEISTSTHEQTQALGQISQSIGQLDQM
yfred0001_12940  ATMDNI----------------VKQAGQVSTLISEISTSTHEQTQALGQISQSISQLDQM
yinte0001_14220  ATMDNI----------------VKQAGQVSTLISEISTSTQEQTQALGQINQSIGQLDQM
ymoll0001_14640  ATMDNI----------------VKQAGQVSTLISEISTSTHEQTQALGQISQSIGQLDQM
yberc0001_15000  ATMDNI----------------VKQAGQVSTLISEISTSTHEQTQALGQISQSIGQLDQM
ykris0001_42780  ATMDNI----------------VQQAGQVSTLIREISTSTYEQTQALGQISQSIGQLDQM
yente0001X_2568  ATMDNI----------------VKQAGQVSALISEISTSTHEQTQALGQISQSIGQLDQM
yberc0001_5850   NTMTEI----------------VAMVSKVSGIMQDITQASEEQSMGIRQVAVAINEMDVV
ymoll0001_5160   NTMSEI----------------VAMVNKVSDIMQDITQASEEQSMGIRQVAVAINEMDIV
yberc0001_6170   ETMSDI----------------VSSITNVSNIMQEISIASEEQSAGVNLIAVAINQMDTM
yaldo0001_6750   ------------------------------------------------------------
yfred0001_39550  ------------------------EVSRMTVLMREISAAIYEQSSGIEQVNVVIAQTDQV
yrohd0001_27170  KTMNEI----------------ASEVSHVTALMREISAATYEQSSGIEQVNVAIAQMDQV
ykris0001_32930  KTMHEI----------------ADEVSRVTALMREISAASYEQSSGIEQVNVAIAQMDQV
yente0001X_3909  KTMHEI----------------ADEVNRVTALMREISAASYEQSSGIEQVNVAIAQMDQV
ymoll0001_40600  KTMHEI----------------ASEVSRVTALMREISAASYEQSSGIEQVNVAIAQMDQV
yberc0001_29090  KTMHEI----------------ASEVSRVTALMREISAASYEQSSGIEQVNVAIAQMDQV
ypseu0001X_1449  NAMTDI----------------VSSVSDVTTIMSEITNAADEQMRGIHEINSAVAQLDTM
ypest0001X_1447  NAMTDI----------------VSSVSDVTTIMSEITNAADEQMRGIHEINSAVAQLDTM
yfred0001_36240  NS----------------------------------------------------------
yinte0001_10140  NTMTEI----------------VSSVSDVTTIMSEITNAADEQMRGIHEINSAVAQLDTM
ykris0001_10260  NTMTEI----------------VSSVSDVTTIMSEITNAADEQMRGIHEINSAVAQLDTM
yente0001X_2906  NTMTEI----------------VSSVSDVTTIMSEITNAADEQMRGIHEINSAVTQLDTM
yfred0001_36230  NTMTEI----------------VSSVSDVTTIMSEITNAADEQMRGIHEINSAVAQLDTM
ymoll0001_9480   NTMTEI----------------VSSVSDVTTIMSEITNAADEQMRGIHEINSAVAQLDTM
yberc0001_9790   NTMTEI----------------VSSVSDVTTIMSEITNAADEQMRGIHEINSAVAQLDTM
yruck0001_28810  HTMNDL----------------MKGVSNVAELMNEIMSSSQEQSLGIEQVNIAINQLDNS
yrohd0001_31660  TTMDDL----------------MSGVSNVAELMNEIMSSSQEQSLGIEQVNLAINQLDNS
yaldo0001_35590  TTMDNL----------------MGGVSNVATLMNEIMSSSREQSLGIDQVNLAINQLDNS
ykris0001_37060  TTMDDL----------------MSGVSNVASLMNEIMSSSQEQSLGIEQVNLAINQLDNS
yinte0001_37560  TTMDDL----------------MSGVSNVAALMNEIMSSSQEQSLGIEQVNLAINQLDNS
yfred0001_32450  TTMDDL----------------MSGVSNVAALMNEIMASSQEQSLGIEQVNLAINQLDNS
yente0001X_300   TTMDDL----------------MSGVSNVAALMNEIMSSSQEQSLGIEQVNLAINQLDNS
ymoll0001_35290  TTMDDL----------------MSGVSNVAALMNEIMSSSQEQSLGIEQVNLAINQLDNS
yberc0001_34000  TTMDDL----------------MSGVSNVAALMNEIMSSSQEQSLGIEQVNLAINQLDNA
ykris0001_22290  ETLTEL----------------VGNVQDVNSIIGEISQASREQSDGINQINLAVGQIDTT
yinte0001_3170   DAMDKI----------------VSSVSNVNDIMSEIAAASTEQSKGISQIGTAVVQMDSV
yruck0001_31650  GAMTEI----------------VTAIGQVTETMQEISNASEEQSRGITQIAQAVNEMDKV
ymoll0001_7110   ITMNEI----------------VESIKQVSDIMEEINEANQEQTSGIQQINQAIIQMDNV
yruck0001_28370  QTMEDI----------------VRSVTHVRDIMAEIASASDEQSRGITQVSQAISEMDST
ypseu0001X_4346  NTMEEI----------------VRSVSHVRDIMAEIASASDEQSRGITQVSLAISEMDST
ypest0001X_4370  NTMEEI----------------VRSVSHVRDIMAEIASASDEQSRGITQVSLAISEMDST
yrohd0001_31240  KTMDDI----------------VRSVTHVHDIMAEIASASDEQSRGITQVSQAIAEMDST
yaldo0001_35130  QTMENI----------------VRSVTHVRDIMAEIASASDEQSRGITQVSLAISEMDST
yinte0001_36970  QTMENI----------------VRSVTHVRDIMAEIASASDEQSRGITQVSQAISEMDST
ykris0001_39870  QTMEDI----------------VRSVTHVRDIMAEIASASDEQSRGITQVSQAISEMDST
ymoll0001_34760  QTMEEI----------------VRSVTHVRDIMAEIASASDEQSRGITQVSQAIAEMDST
yfred0001_31920  QTMEDI----------------VRSVTHVRDIMAEIASASDEQSRGITQVSQAISEMDST
yberc0001_33520  QTMEEI----------------VRSVTHVRDIMAEIASASDEQSRGITQVSQAISEMDST
yente0001X_760   QTMEDI----------------VRSVTHVRDIMAEIASASDEQSRGITQVSQAISEMDST
yruck0001_18270  STMNEI----------------VRSITNVTDLIGEIASASDEQSKGISQVGQAVAEMDSV
yrohd0001_33070  ATMDEI----------------VRSITNVTDLMSEIASASDEQSKGISQVGLAVAEMDTV
yfred0001_34740  VTMDEI----------------VRSITNVTDLMGEIASASDEQSKGITQVGQAVAEMDSV
yaldo0001_22600  ITMDEI----------------VRSITNVTDLMGEIASASDEQSKGISQVGQAVAEMDSV
yinte0001_40830  ITMDEI----------------VRSITNVTDLMGEIASASDEQSKGISQVGQAVAEMDSV
ymoll0001_38830  ITMDEI----------------VRSITNVTDLMGEIASASDEQSKGISQVGQAVAEMDSV
yberc0001_21820  VTMDEI----------------VRSITNVTDLMGEIASASDEQSKGISQVGQAVAEMDSV
ykris0001_22260  ITMDEI----------------VRSITNVTDLMGEIASASDEQSKGITQVGQAVAEMDSV
yente0001X_1297  ITMDEI----------------VRSITNVTDLMGEIASASDEQSKGITQVGQAVAEMDSV
yruck0001_11920  ETMGDI----------------VNAVTRVTDIMGEIASASDEQSRGIDQVGQAVTEMDRV
ypseu0001X_2707  ETMGDI----------------VNAVTRVTDIMGEIASASDEQSRGIDQIGQAVAEMDRV
ypest0001X_1916  ETMGDI----------------VNAVTRVTDIMGEIASASDEQSRGIDQIGQAVAEMDRV
yrohd0001_33620  ETMGDI----------------VNAVTRVTDIMGEIASASDEQSRGIDQVGQAVTEMDRV
yaldo0001_15230  ETMGDI----------------VSAVTRVTDIMGEIASASDEQSRGIDQVGQAVTEMDRV
ykris0001_37850  ETMGDI----------------VNAVTRVTDIMGEIASASDEQSRGIDQVGQAVTEMDRV
yente0001X_1721  ETMGDI----------------VNAVTRVTDIMGEIASASDEQSRGIDQVGQAVTEMDRV
ymoll0001_37090  ETMGDI----------------VNAVTRVTDIMGEIASASDEQSRGIDQVGQAVTEMDRV
yberc0001_15260  ETMGDI----------------VNAVTRVTDIMGEIASASDEQSRGIDQVGQAVTEMDRV
yinte0001_40500  ETMGDI----------------VNAVTRVTDIMGEIASASDEQSRGIDQVGQAVTEMDRV
yfred0001_14080  ETMGDI----------------VNAVTRVTDIMGEIASASDEQSRGIDQVGQAVTEMDRV
yruck0001_11930  TTMEEI----------------VRSVTRVTDIMGEIASASDEQSRGIEQVSLAVTQMDQV
yaldo0001_6760   LKQEKR------------------------------------------------------
ypest0001X_1918  --MEEI----------------VRSVARVTDIMGEIESASDEQSRGIEQISLAVTQMDQV
ypest0001X_1917  ------------------------------------------------------------
ypseu0001X_2706  TTMEEI----------------VRSVARVTDIMGEIESASDEQSRGIEQISLAVTQMDQV
yrohd0001_33630  TTMEEI----------------VRSVTRVTDIMGEIASASDEQSRGIEQVSLAVTQMDQV
yaldo0001_15240  TTMEEI----------------VRSVTRVTDIMGEIASASDEQSRGIEQVSLAVTQMDQV
ymoll0001_14920  TTMEEI----------------VRSVTRVTDIMGEIASASDEQSRGIEQVSLAVTQMDQV
yberc0001_15270  TTMEEI----------------VRSVTRVTDIMGEIASASDEQSRGIEQVSLAVTQMDQV
yfred0001_14090  TTMEEI----------------VRSVTRVTDIMGEIASASDEQSRGIEQVSLAVTQMDQV
yinte0001_15900  TTMEEI----------------VRSVTRVTDIMGEIASASDEQSRGIEQVSLAVTQMDQV
ykris0001_37840  TTMEEI----------------VRSVTRVTDIMGEIASASDEQSRGIEQVSLAVTQMDQV
yente0001X_1722  TTMEEI----------------VRSVTRVTDIMGEIASASDEQSRGIEQVSLAVTQMDQV
yruck0001_10900  KAMSEI----------------VTAVTQVTDIMAEIASASDEQSRGISQVAQAVSEMDNV
yaldo0001_6770   --MNSI----------------VQAVTNVTDIMGEIASASDEQSRGISQIGQAVAVLPSK
yaldo0001_15110  KTMNEI----------------VTAVIHVTDIMGEIASASDEQSRGISQVAQAISEMDDV
yinte0001_40380  KAMNQI----------------VTAVTHVTDIMGEIASASDEQSRGISQVAQAVSEMDNV
ymoll0001_13500  KTMSEI----------------VTAVTHVTDIMGEIASASDEQSRGISQVAQAVSEMDNV
yberc0001_14860  KTMSEI----------------VTAVTHVTDIMGEIASASDEQSRGISQVAQAVSEMDNV
yrohd0001_33510  KTMKEI----------------VTAVTHVTDIMGEIASASDEQSRGISQVAQAVSEMDNV
yfred0001_13960  KTMKEI----------------VTAVTHVTDIMGEIASASDEQSRGISQVAQAVSEMDNV
yente0001X_1708  KAMNEI----------------VTAVTHVTDIMGEIASASDEQSRGISQVAQAVSEMDNV
ykris0001_38040  KTMKEI----------------VTAVTHVTDIMGEIASASDEQSRGITQVAQAVSEMDNV
yrohd0001_6540   KTMSTI----------------VQAVTNVTDIMGEIASASDEQSRGIGQIGQAVAEMDGV
yente0001X_6250  ETMNSI----------------VQAVTNVTDIMGEIASASDEQSRGISQIGQAVAEMDGV
yruck0001_10760  EDMKIL----------------LDEVDQVKDFVGSIAMASDEQSRGIEQVNIAIAQLEQI
ypest0001X_1900  ------------------------------------------------------------
ypseu0001X_2718  KTMNEI----------------VTAVTHVTDIMGEIASASDEQSRGISQVAQAVSEMDNV
yaldo0001_37010  ------------------------------------------------------------
ypest0001X_1899  KTMNEI----------------VTAVTHVTDIMGEIASASDEQSRGISQVAQAVSEMDNV
yfred0001_6650   ETMNSI----------------VQAVTNVTDIMGEIASASDEQSRGISQIGQAVAEMDGV
yinte0001_6950   ETMNSI----------------VQAVTNVTDIMGEIASASDEQSRGIGQIGQAVAEMDGV
ymoll0001_37360  ETMNSI----------------VQAVTNVTDIMGEIASASDEQSRGISQIGQAVAEMDGV
yberc0001_8160   ETMNSI----------------VQAVTNVTDIMGEIASASDEQSRGISQIGQAVAEMDGV
                                                                             


                        910       920       930       940       950       960
                 =========+=========+=========+=========+=========+=========+
ykris0001_41300  ------------------------------------------------------------
yrohd0001_20110  TLTQSRVIESYS------------------------------------------------
yaldo0001_2440   ARQNLPLLSHLIHTLKGCAGQAGLIELYEAAL-QLE-----------TVLETHNIMTQQD
ypseu0001X_4220  AIENLPVLSHTLHTIKGCAGQAGLIELQDAVI-QLE-----------HALDTHETLTQQD
ypest0001X_4540  AIENLPVLSHTLHTIKGCAGQAGLIELQDAVI-QLE-----------HALDTHETLTQQE
yinte0001_2660   --------------LKGPGWQQLVIYPRVGFA----------------------------
yberc0001_2350   SLTHIPSLSHIIHTLKGCAGQAGLIELQNAAI-QLE-----------IAIEAKETITQQD
ymoll0001_1840   SLTHLPSLSHIIHTLKGCAGQAGLIELQNAAI-QLE-----------IAIEDQDTITSQD
ykris0001_2490   SLTHTPTLSDHLHTLKGCAGQAGLIELQDAVI-QLE-----------IAIEAKEVITQED
yinte0001_2650   SLTHTPTLSHQLHTLKGCAGQAGLIELQDAVI-QLE-----------IAIENNELITQQD
yrohd0001_2860   SLTHVPTLSDHLHTLKGCAGQAGLDALQDAVI-ELE-----------IAIETQEIITQQD
yfred0001_38320  SLVHIPTLSEHLHTLKGCAGQAGLIVLHDAVI-QLE-----------IAIEAQETITQQD
yinte0001_26450  -----------------------------TIF----------------------------
yberc0001_7120   -------------------------------F----------------------------
ypseu0001X_3409  TKQNAALVEQSAHASEVLQQQTLQLNQSVARF-HLP-----------VEKYSPLRLAKES
ypest0001X_1004  TKQNAALVEQSAHASEVLQQQTLQLNQSVARF-HLP-----------VEKYSPLRLAKES
yente0001X_9160  TRLNADLVDQSTQASEVLQKQIFQLNQSVARF-CLP-----------VTVRPLNILMRRS
ykris0001_6100   TKLNAELVGQSTRASEVLQQQIFQLNQSVARF-CLP-----------TTIQPPQRINEEV
yfred0001_43450  TKLNAELVEQSTRASEVLQQQIFQLNQSVARF-CLP-----------GAVRPPQDIHEEV
ymoll0001_7580   TRMNAELVEQSTRASEALQKQTFQLNQSVARF-CLP-----------VMTRPAQQGNEQV
yinte0001_8280   TKLNAELAQQSTCASEVLQKQASQLNESVARF-CLP-----------TTGRPAQGCYEAA
ykris0001_6110   ----------------------------DAFF----------------------------
yrohd0001_35950  KNQQVLM-----------------------------------------------------
ypseu0001X_2873  KNQRAPV-----------------------------------------------------
ypest0001X_2787  KNQ---------------------------------------------------------
ymoll0001_11800  KNQQTHV-----------------------------------------------------
yfred0001_8890   KNQQSLI-----------------------------------------------------
ykris0001_12740  KNQQTHV-----------------------------------------------------
yente0001X_1434  KNQQPHI-----------------------------------------------------
yrohd0001_31880  ------------------------------------------------------------
yrohd0001_15520  TQQNAALVEEAAAAAQSLTEQGAELRQVVRFF-QIN------------------------
yfred0001_15260  TQQNAALVEQAAAAAQSLTEQGAELRQVIRFF-HIN------------------------
yinte0001_16590  TQQNAALVEQAAAAAQSLTEQGAELRQIIRFF-QIN------------------------
ymoll0001_41560  TQQNAALVEEAAAAAQSLTEQGAELRQVIRFF-QIS----------------GSTIS---
yberc0001_16020  TQQNAALVEEAAAAAQSLTEQGAELRQVIRFF-QIN------------------------
yberc0001_5770   TQQNAAMVEEAALAANNLEMQSEELDSIISKF-TINKNIGNRKTHRERDNGIERNRDKKT
ymoll0001_5080   TQQNAVMVEEAALAASNLETESEALDNIISEF-VIN-----------KPIEDNGAERKRD
yaldo0001_37020  THQNAAMTEQYAGAAEELAYRTLRLTAAMRIS----------------------------
ypseu0001X_2733  TYQNAAMVEQYAGAAEELAHRTLRLTAAVRIY---R-----------PLNNVGKLRD---
ypest0001X_1885  TYQNAAMVEQYAGAAEELAHRTLRLTAAVRIY---R-----------PLNNVGKLRD---
yrohd0001_12010  THQNAAMVEQYAGAAEELAHRTLRLTRAIRIY---R-----------PIK----------
yfred0001_12940  THQNAAMVEQYAGAAEELAHRTLRLTAAVRIY---R-----------PVK----------
yinte0001_14220  THQNAAMVEQYAGAAEELAHRTLRLTAAVRIY---R-----------PVK----------
ymoll0001_14640  THQNAAMVEQYAGAAEELAHRTLRLTAAVRIY---R-----------PVK----------
yberc0001_15000  THQNAAMVEQYAGAAEELAHRTLRLTAAVRIY---R-----------PVK----------
ykris0001_42780  THQNSAMVEQYAGAAEELAHRTLRLTAAVRIY---R-----------PVK----------
yente0001X_2568  THQNAAMVEQYAGAAEELAHRTLRLTAAVRIY---R-----------PVN----------
yberc0001_5850   TQQNSALVEESATITALMNEQTMVMEEMVSVF-QLN----------EAELSR--------
ymoll0001_5160   TQRNSTLVEESAAITVLMNEQTTIMEEMVSVF-QIE-------------VDRHHA-----
yberc0001_6170   TQQNASLVEETARNTRTLSNQTKELSATVSVF-QID-----------NEDYQQKTVSSQE
yaldo0001_6750   ------------------------------------------------------------
yfred0001_39550  AQHGGI------------------------------------------------------
yrohd0001_27170  AQQNAALVEQAAAATHSLEEQADALSASVAVF-KLQ-------------GDKLAGLA---
ykris0001_32930  AQQNATLVEQAAAATRSLEEQADALSASMAVF-KLQ-------------GDELAVIA---
yente0001X_3909  AQQNATLVEQAAAATRSLEEQADALSASMAVF-KLQ-------------GDELAVVA---
ymoll0001_40600  AQQNATLVEQAAAATRSLEEQADALSASMAVF-KLQ-------------GDKLALEV---
yberc0001_29090  AQQNATLVEQAAAATRSLEEQADALSASMAVF-KLH-------------GDKLALEA---
ypseu0001X_1449  VQQNAALVQESTAASAALQAQAADLTDTVNQF-KI-------------------------
ypest0001X_1447  VQQNAALVQESTAASAALQAQAADLTDTVNQF-KI-------------------------
yfred0001_36240  ------------------------------------------------------------
yinte0001_10140  VQQNAALVQESTAASAALQAQATDLTSAVNQF-RI-------------------------
ykris0001_10260  VQQNAALVQESTAASAALQAQAADLTGAVNQF-RI-------------------------
yente0001X_2906  VQQNAALVQESTAASAALQAQAADLTSAVNQF-RI-------------------------
yfred0001_36230  VQQNAALVQESTAASAALQAQAADLTGAVNQF-RI-------------------------
ymoll0001_9480   VQQNAALVQESTAASAALQAQAADLTGAVNQF-RI-------------------------
yberc0001_9790   VQQNAALVQESTAASAALQSQAGDLTEAVNQF-RI-------------------------
yruck0001_28810  TQQNAALVEQVAAAAQAMQEQTVQLEGVISGF-KI-------------------------
yrohd0001_31660  TQQNAALVEQVAAAAQAMQDQTLQLEQVISGF-KV-------------------------
yaldo0001_35590  TQQNAALVEQVAAAAQAMQDQTLQLESVISGF-KV-------------------------
ykris0001_37060  TQQNAALVEQVAAAAQAMQDQTVQLESVISGF-KV-------------------------
yinte0001_37560  TQQNAALVEQVAAAAQAMQDQTLQLESVISGF-KV-------------------------
yfred0001_32450  TQQNAALVEQVAAAAQAMQDQTVQLETVISGF-KV-------------------------
yente0001X_300   TQQNAALVEQVAAAAQAMQDQTLQLESVISGF-KV-------------------------
ymoll0001_35290  TQQNAALVEQVAAAAQAMQDQTLQLESVISGF-KV-------------------------
yberc0001_34000  TQQNAALVEQVAAAAQAMQDQTLQLESVISGF-KV-------------------------
ykris0001_22290  TQQNAALVEESAAAALSLQAQASTLTETVSAF-KLH----------STGSDDSRELANAA
yinte0001_3170   TQQNAALVQQSAAAAASLEEQARQLTEIVSVF-KIK-------------GKAPDSSTGP-
yruck0001_31650  TQQNAALVEQSASAASALEDQADRLNQTVAIF---K-----------LAASGSAPEKIRV
ymoll0001_7110   TQQNAALVEESAAAAQSLQDQAENLSQVINIF-NLN-----------AAYTATATPTP--
yruck0001_28370  TQQNAALVEESAAAADSLAEQAILLAQAVAVF-RLS-----------ESTESTKTTPTT-
ypseu0001X_4346  TQQNAALVEESAAAADSLAEQAILLAQAVAVF-RLS-------------EDEGATDEGRV
ypest0001X_4370  TQQNAALVEESAAAADSLAEQAILLAQAVAVF-RLS-------------EDEGATDEGRV
yrohd0001_31240  TQQNAALVEESAAAADSLAEQAILLAQAVAVF-RLS-------------ESEAETPQHQH
yaldo0001_35130  TQQNAALVEESAAAANSLAEQAILLAQAVAVF-RLT------------DTETETVAAQHK
yinte0001_36970  TQQNAALVEESAAAADSLAEQAILLAQAVAVF-RLS----------DAEVESAQKNTSAS
ykris0001_39870  TQQNAALVEESAAAADSLAEQAILLEQAVAVF-RLS----------EAEVETVQSNTSAS
ymoll0001_34760  TQQNAALVEESAAAADSLAEQAILLAQAVAVF-RLS------------EAETQPVQHQPA
yfred0001_31920  TQQNAALVEESAAAADSLAEQAILLAQAVAVF-RLS----------ESEAETSQSHSVSS
yberc0001_33520  TQQNAALVEESAAAADSLAEQAILLAQAVAVF-RLS----------DAETHTAQGNIPAP
yente0001X_760   TQQNAALVEESAAAADSLAEQAILLAQAVAVF-RLS----------EAETESVQSETTTP
yruck0001_18270  TQQNAALVQEASRAAASLEDQAAKLNHAVAVF-KLK-----------DDKVTPAAKTKV-
yrohd0001_33070  TQQNAALVQEASRAAASLEEQATQLNLAVAVF-KLR-----------SDDQRSKPVA---
yfred0001_34740  TQQNAALVQEASRAAASLEEQATQLNLAVAVF-KLQ-----------SDEERVKPAAK--
yaldo0001_22600  TQQNAALVQEASRAAASLEEQATQLNRAVAVF-KLH-----------LDDERVKPAVK--
yinte0001_40830  TQQNAALVQQASRAAASLEEQATQLNQAVAVF-KLK-----------ADDERAKPVVKM-
ymoll0001_38830  TQQNAALVQEASRAAASLEEQATQLNRAVAVF-KLQ-----------SDDARAKPAMKP-
yberc0001_21820  TQQNAALVQEASRAAASLEEQATQLNRAVAVF-KLQ-----------SDDARAKPAVKP-
ykris0001_22260  TQQNAALVQQASRAAASLEEQAAQLNQAVAVF-KLR-----------SDDERAKPAAK--
yente0001X_1297  TQQNAALVQQASRAAAALEEQATQLNQAVAVF-KLQ-----------SDDVRTKSVAK--
yruck0001_11920  TQQNASLVEESASAAAALEEQASLLTQSMSVF-ILR-----------MDNGSNSKDVRRV
ypseu0001X_2707  TQQNASLVEESASAAASLEEQASMLTQAMSVF-VLS-----------VDNSNSTSDVRKV
ypest0001X_1916  TQQNASLVEESASAAASLEEQASMLTQAMSVF-VLS-----------VDNSNSTSDVRKV
yrohd0001_33620  TQQNASLVEESASAAAALEEQASMLTQSMSVF-VLR-----------MDNSSTSRNIKKT
yaldo0001_15230  TQQNASLVEESASAAAALEEQASMLTQSMSVF-VLR-----------MDNGNSTKDVRKT
ykris0001_37850  TQQNASLVEESASAAAALEEQASILTQSMSVF-VLH-----------MDNDSTKKDVKKT
yente0001X_1721  TQQNASLVEESASAAAALEEQASMLTQSMSVF-ILR-----------MDNSSSKKDVRKT
ymoll0001_37090  TQQNASLVEESASAAAALEEQASMLTQSMSVF-VLR-----------MDNGSSTRNVRKT
yberc0001_15260  TQQNASLVEESASAAAALEEQASMLTQSMSVF-VLR-----------MDNGSSTKSVRKI
yinte0001_40500  TQQNASLVEESASAAAALEEQASMLTQSMSVF-VLR-----------MDNGSTTRDARKI
yfred0001_14080  TQQNASLVEESASAAAALEEQASMLTQSMSVF-ILR-----------MDNGSTTRDVRKP
yruck0001_11930  TQQNAALVEEAASAANALEEQAGYLSHAVSAF-HLA-----------QDGYDEDWQGTGK
yaldo0001_6760   ------------------------------------------------------------
ypest0001X_1918  TQQNASLVEEAAAAANALEEQASMLSDAVSVF-RLG---------QGHDEQSVAGNSQQS
ypest0001X_1917  ------------------------------------------------------------
ypseu0001X_2706  TQQNASLVEEAAAAANALEEQASLLSDAVSVF-RLG---------QGHDEQSVAGNSQQS
yrohd0001_33630  TQQNAALVEEAAAAANALEEQASMLSNAVSVF-RLE-----------QGSDNGDWQAAEG
yaldo0001_15240  TQQNAALVEEAAAAANALEEQASMLSDAVSVF-RLE-----------QDSDSGEGQAADG
ymoll0001_14920  TQQNAALVEEAAAAANALEEQASRLSNAVSVF-RLE-----------QDSDSGEGQGSSK
yberc0001_15270  TQQNAALVEEAAAAANALEEQASMLSNAVSVF-RLE-----------QDSDSGEGQSSSK
yfred0001_14090  TQQNAALVEEAAAAANALEEQASMLSNAVSVF-RLE-----------QGSDNEEGQAADG
yinte0001_15900  TQQNAALVEEAAAAANALEEQASRLSDAVSVF-RLE-----------QDSDSGEGQAADG
ykris0001_37840  TQQNAALVEEAAAAANALEEQAGMLSDAVSVF-RLE-----------QYSDGGEGQAAAG
yente0001X_1722  TQQNAALVEEAAAAANALEEQAGMLSDAVSVF-RLE-----------QDSDNGEGQSADR
yruck0001_10900  TQQNASLVQEASAAAASLEQQAAMLTQAVAVF-QLN-----------GHSKTMTRSITAG
yaldo0001_6770   MLH---------------------------------------------------------
yaldo0001_15110  TQQNASLVQEASAAAASLEQQGEILTQAVAVF-RLN-------------GHRPAPTVKQ-
yinte0001_40380  TQQNASLVQEASAAAASLEQQAEILTQAVAVF-HLK-----------GHNPAPSLKTPAP
ymoll0001_13500  TQQNASLVQEASAAAASLEQQAEVLTQAVAVF-HLN-----------GHNPASKVKTSVP
yberc0001_14860  TQQNASLVQEASAAAASLEQQAEVLTQAVAVF-HLN-------------GHNPAAKVKRS
yrohd0001_33510  TQQNASLVQEASAAAASLEQQAEMLTQAVAVF-HLK-----------GRNSTAALKTPTL
yfred0001_13960  TQQNASLVQEASAAAASLEQQAEVLTQAVAVF-HLK-----------GRNPSTTLKTPTP
yente0001X_1708  TQQNASLVQEASAAAASLEQQAEILTQAVAVF-HLN-----------GRNPAPALKTPAP
ykris0001_38040  TQQNASLVQEASAAAASLEQQAEILTQAVAVFH-LN-----------GRNPAPSLKTPTP
yrohd0001_6540   TQQNASLVQESAAAAASLEEQARQLTQAVAVF-NLS-----------NNI----------
yente0001X_6250  TQQNASLVQESAAAAASLEEQARQLTEAVSAF-NLS-----------DNTQAA-------
yruck0001_10760  AQQNAALVEEASAATDSLADQADTLDEVMQIF-TLS-----------KTMQ---------
ypest0001X_1900  ------------------------------------------------------------
ypseu0001X_2718  TQQNASLVQEASAAAASLEQQAEILTQAVAVF-QLA-------------GHSSIKEFKSP
yaldo0001_37010  ------------------------------------------------------------
ypest0001X_1899  TQQNASLVQEASAAAASLEQQAEILTQAVAVF-QLA-------------GHSSIKEFKSP
yfred0001_6650   TQQNASLVQESAAAAASLEEQARQLTEAVSVF-NLS--------------DSAQVS----
yinte0001_6950   TQQNASLVQESAAAAASLEEQARQLTEAVSVF-NLS-----------DNMQATHS-----
ymoll0001_37360  TQQNASLVQESAAAAASLEEQARQLTDAVSAF-KLS-----------DNMQTA-------
yberc0001_8160   TQQNASLVQESAAAAASLEEQARQLTDAVSAF-KLS-----------DNMQEYNHN----
                                                                             


                        970       980       990
                 =========+=========+=========+=====
ykris0001_41300  -----------------------------------
yrohd0001_20110  -----------------------------------
yaldo0001_2440   IAHLDKIIDLLFNPGAPATPPQSSC----------
ypseu0001X_4220  IIQLDEIIHVLLQPPTTCESTFIISQGQSLMVK--
ypest0001X_4540  IIQLDEIIHVLLQPPTTCESTFIISQGQSLMVK--
yinte0001_2660   -----------------------------------
yberc0001_2350   LTRLDEIIHSLLA----------------------
ymoll0001_1840   ISRLDEIIHSLF-----------------------
ykris0001_2490   INRLDEITHWLFQP---------------------
yinte0001_2650   IDHLGEIIHLLFQSRDPRDTISIIR----------
yrohd0001_2860   ITRLEDLIHGLF-----------------------
yfred0001_38320  ITRLDEIIHGLFQPRSLPDSI--------------
yinte0001_26450  -----------------------------------
yberc0001_7120   -----------------------------------
ypseu0001X_3409  TIAAIDSRVS-------------------------
ypest0001X_1004  TIAAIDSRVS-------------------------
yente0001X_9160  L----------------------------------
ykris0001_6100   AVSF-------------------------------
yfred0001_43450  FISI-------------------------------
ymoll0001_7580   AVNF-------------------------------
yinte0001_8280   SANF-------------------------------
ykris0001_6110   -----------------------------------
yrohd0001_35950  -----------------------------------
ypseu0001X_2873  -----------------------------------
ypest0001X_2787  -----------------------------------
ymoll0001_11800  -----------------------------------
yfred0001_8890   -----------------------------------
ykris0001_12740  -----------------------------------
yente0001X_1434  -----------------------------------
yrohd0001_31880  -----------------------------------
yrohd0001_15520  ------------EPSVN------------------
yfred0001_15260  ------------EPSVS------------------
yinte0001_16590  ------------QPSLN------------------
ymoll0001_41560  -----------------------------------
yberc0001_16020  ------------GPAVS------------------
yberc0001_5770   LRP---------GPVSA-KQSEMNKSIAEEQWETF
ymoll0001_5080   KKS------ARFSSTSVKKEPEIIKNTAEAEWETF
yaldo0001_37020  ------------ARQPINAVRSIASVDKPLTLKRR
ypseu0001X_2733  -----------------------------------
ypest0001X_1885  -----------------------------------
yrohd0001_12010  -----------------------------------
yfred0001_12940  -----------------------------------
yinte0001_14220  -----------------------------------
ymoll0001_14640  -----------------------------------
yberc0001_15000  -----------------------------------
ykris0001_42780  -----------------------------------
yente0001X_2568  -----------------------------------
yberc0001_5850   -----------------------------------
ymoll0001_5160   -----------------------------------
yberc0001_6170   VEA--------------------------------
yaldo0001_6750   -----------------------------------
yfred0001_39550  -----------------------------------
yrohd0001_27170  -----------------------------------
ykris0001_32930  -----------------------------------
yente0001X_3909  -----------------------------------
ymoll0001_40600  -----------------------------------
yberc0001_29090  -----------------------------------
ypseu0001X_1449  -----------------------------------
ypest0001X_1447  -----------------------------------
yfred0001_36240  -----------------------------------
yinte0001_10140  -----------------------------------
ykris0001_10260  -----------------------------------
yente0001X_2906  -----------------------------------
yfred0001_36230  -----------------------------------
ymoll0001_9480   -----------------------------------
yberc0001_9790   -----------------------------------
yruck0001_28810  -----------------------------------
yrohd0001_31660  -----------------------------------
yaldo0001_35590  -----------------------------------
ykris0001_37060  -----------------------------------
yinte0001_37560  -----------------------------------
yfred0001_32450  -----------------------------------
yente0001X_300   -----------------------------------
ymoll0001_35290  -----------------------------------
yberc0001_34000  -----------------------------------
ykris0001_22290  FLVTER-----------------------------
yinte0001_3170   ------------LLRPKTRVKSAVLAAEQGGWTKF
yruck0001_31650  QPD---------QGRLMAAN---------------
ymoll0001_7110   -----------------------------------
yruck0001_28370  ------------KTTPPRMTAENVKLNQQDNWETF
ypseu0001X_4346  VTP---------KASNGRHTPEDHRINQQKSWETF
ypest0001X_4370  VTP---------KASNGRHTPEDHRINQQKSWETF
yrohd0001_31240  AEAVMARHVSASNSTAARQTSETRQNNQQDNWETF
yaldo0001_35130  ------------GTPSPRTGSQSSRVTQQDNWETF
yinte0001_36970  VVHK--------STPTSRTGTESSRTAQQDNWETF
ykris0001_39870  ------------RVATPRPAPDASRNDQQDNWETF
ymoll0001_34760  AT----------SASTPRSTPKANQATQQDNWETF
yfred0001_31920  APK---------NTPTPRPTPETRNSAPQDNWETF
yberc0001_33520  ATKN--------SASTPRPGPNISRANQQDNWETF
yente0001X_760   ------------RATTPRPAPEANRSQQQDNWETF
yruck0001_18270  ---------------TPVKAPMAPRTDNNANWETF
yrohd0001_33070  ------------KAKVLTKVTAPVRVDSNDNWETF
yfred0001_34740  ------------PRAKVSAATAVSKPDSNANWETF
yaldo0001_22600  ------------PRAHPTAMAPVTKVDNNANWETF
yinte0001_40830  ------------RANQRTTAPATAKADSNANWETF
ymoll0001_38830  ------------RANPLTLTPVATKADSNANWETF
yberc0001_21820  ------------RANPLKLAPVAAKADSNANWETF
ykris0001_22260  ------------PRARVLAATPTSKVDNNTNWETF
yente0001X_1297  ------------PRATVLTTAPIAKVDNNANWETF
yruck0001_11920  K-----------QPTVEAVAKKALGSDLQDNWETF
ypseu0001X_2707  KQP---------TQEMNSTAKKALGNNLQDNWETF
ypest0001X_1916  KQP---------TQEMNSTAKKALGNNLQDNWETF
yrohd0001_33620  KQP---------AQDLGVTAKKTLGSDLQENWETF
yaldo0001_15230  KQP---------AQDLNITAKKILGSDLQDNWETF
ykris0001_37850  KQP---------TQDKSGTAKKTLGSDLQDNWETF
yente0001X_1721  KQP---------TQDKSGTAKKALGSDLQENWETF
ymoll0001_37090  KQP---------TQDTSGTAKKTLGSDLQDNWETF
yberc0001_15260  KQP---------TQDLSGTAKKTLGSDLQDNWETF
yinte0001_40500  KQP---------AQDLSITAKKTLGSDLQDNWETF
yfred0001_14080  KQP---------TQDQSGAAKKTLGSDLQENWETF
yruck0001_11930  QSALKEISDC-------------------------
yaldo0001_6760   -----------------------------------
ypest0001X_1918  LATHY------------------------------
ypest0001X_1917  -----------------------------------
ypseu0001X_2706  LATHY------------------------------
yrohd0001_33630  RG----------QQPAVKEFPDCQRV---------
yaldo0001_15240  RDKQRVVKATASKENPDCQTA--------------
ymoll0001_14920  QSVV--------KATVAKEIPDCQMT---------
yberc0001_15270  QSVV--------KATVAKEIPDCQMT---------
yfred0001_14090  SGKQSVVKATVAKENPDCQTA--------------
yinte0001_15900  SG----------KQPAVKEIPDCQTA---------
ykris0001_37840  SGQQFVVKTAAAKETPDCQTV--------------
yente0001X_1722  SS----------KQPAVKEIPDCQTA---------
yruck0001_10900  ------------DKKIANNLDAKKAELNNENWETF
yaldo0001_6770   -----------------------------------
yaldo0001_15110  ------------STKLKSTSVNKKTDDDSFNWEKF
yinte0001_40380  M-----------SAMQNNAAKNKKSGDDALNWETF
ymoll0001_13500  T-----------SAPQNSKGVNKKSGDDSLNWETF
yberc0001_14860  VLA---------PTGQNSKGANKKSGDDSLNWETF
yrohd0001_33510  T-----------AAGQNKANINKKPSDDSLNWETF
yfred0001_13960  M-----------STGQNQTNLNKKSGDDSLNWETF
yente0001X_1708  T-----------SIGQNKTGGNKRPTDDSLNWETF
ykris0001_38040  S-----------SGGQNKTGGNKKSADDSLNWETF
yrohd0001_6540   -----------------------------------
yente0001X_6250  -----------------------------------
yruck0001_10760  -----------------------------------
ypest0001X_1900  -----------------------------------
ypseu0001X_2718  VSP---------PTGQNGSGINKKPTDGSLNWETF
yaldo0001_37010  -----------------------------------
ypest0001X_1899  VSP---------PTGQNGSGINKKPTDGSLNWETF
yfred0001_6650   -----------------------------------
yinte0001_6950   -----------------------------------
ymoll0001_37360  -----------------------------------
yberc0001_8160   -----------------------------------
```

```
Parameters used
Minimum Number Of Sequences For A Conserved Position: 73
Minimum Number Of Sequences For A Flanking Position: 122
Maximum Number Of Contiguous Nonconserved Positions: 8
Minimum Length Of A Block: 10
Allowed Gap Positions: With Half
Use Similarity Matrices: Yes
```

```
Flank positions of the 3 selected block(s)
Flanks: [723  733]  [775  787]  [807  832]  

New number of positions in PGL1_unique_yersinia-CLUSTERS.dir/PGL1_unique_yersinia-CL0/PGL1_unique_yersinia-CL0.muscle.fasta.gblo:  50  (5% of the original 995 positions)
```
