# Supplementary material for: Genomic characterization of the Yersinia genus
Source: Genome Biol. 2010 Jan 4;11(1):R1. doi: 10.1186/gb-2010-11-1-r1 (PMC2847712; doi:10.1186/gb-2010-11-1-r1)
Supplement: Additional file 15 — The top level directory consists of a directory called Additional_cluster_files and 5010 directories, one for each multi-protein cluster family. (This top level directory has been split into three data files for uploading purposes (Additional files 15, 16, 17).) Within the directory are the following files: PGL1_unique_Yersinia_unclustered.out - list of all protein singletons that MCL did not group into a cluster (see Materials and Methods); PGL1_Yersinia_unique_locus_tags.txt - names of the 11 locus tag prefixes used for each genome; PGL1_unique_Yersinia.gff - mapping each Yersinia protein to a cluster in tab delimited GFF; PGL1_unique_Yersinia.sigfile - list of the longest protein in each cluster; PGL1_unique_Yersinia.summary - summary table of features of each of the clusters; PGL1_unique_Yersinia.table - summary table of each protein in the clusters. Within each cluster directory are the following files, where 'x' is the cluster name: PGL1_unique_Yersinia-x.faa - multifasta file of the proteins in the cluster; PGL1_unique_Yersinia-x.summary - summary of the properties of the proteins; PGL1_unique_Yersinia-x.matches - blast matches between the proteins of the cluster; PGL1_unique_Yersinia-x.muscle.fasta - muscle alignment of the proteins; PGL1_unique_Yersinia-x.muscle.fasta.gblo - gblocks output of muscle alignment (that is, auto-trimmed alignment); PGL1_unique_Yersinia-x.muscle.fasta.gblo.htm - as above in html format; PGL1_unique_Yersinia-x.muscle.tree - treefile from muscle alignment; PGL1_unique_Yersinia-x.sif - matches between proteins in simple interaction format for display on graphing software. [file gb-2010-11-1-r1-S15.zip › clusters/PGL1_unique_yersinia-CL1/PGL1_unique_yersinia-CL1.muscle.fasta.gblo.htm]

PGL1\_unique\_yersinia-CL1.muscle.fasta


## Gblocks 0.91b Results

Processed file: **PGL1\_unique\_yersinia-CL1.muscle.fasta**  
Number of sequences: **91**  
Alignment assumed to be: **Protein**  
New number of positions: **0** (selected positions are underlined in blue)

```
                         10        20        30        40        50        60
                 =========+=========+=========+=========+=========+=========+
yberc0001_8630   -------XNKNLYRIVFNKARGLLMVVADIAASGRAASSPSSGVGHTQHRCVTALSRLSF
ykris0001_26670  -----XTMNKNLYR----------------------------------------------
ykris0001_41280  ------------------------------------------------------------
yruck0001_910    ------------------------------------------------------------
yaldo0001_37900  ------------------------------------------------------------
yberc0001_34750  XFNDRQYSSLMAAMVVFGIVSGLMYRAAKGSIELLETGKGNMGLGWLAYTLIGTM--IWF
yaldo0001_38900  ------------------------------------------------------------
yaldo0001_41000  ------------------------------------------------------------
ypseu0001X_3846  ------------------------------------------------------------
ymoll0001_35980  ------------------------------------------------------------
ypest0001X_8100  ------------------------------------------------------------
yfred0001_38200  ------------------------------------------------------------
ypest0001X_2754  ------------------------------------------------------------
yfred0001_34120  ------------------------------------------------------------
ypseu0001X_2842  ------------------------------------------------------------
ykris0001_7890   ------------------------------------------------------------
yruck0001_4640   ------------------------------------------------------------
ymoll0001_2720   ------------------------------------------------------------
yrohd0001_40310  ------------------------------------------------------------
yruck0001_34980  ------------------------------------------------------------
ypest0001X_2756  ------------------------------------------------------------
yfred0001_45620  ------------------------------------------------------------
yrohd0001_18860  ------------------------------------------------------------
yfred0001_33010  ------------------------------------------------------------
yfred0001_33210  ------------------------------------------------------------
yrohd0001_40300  ------------------------------------------------------------
yruck0001_4620   ------------------------------------------------------------
ypest0001X_8110  ------------------------------------------------------------
yberc0001_40130  ------------------------------------------------------------
yberc0001_40750  ------------------------------------------------------------
yruck0001_35050  ------------------------------------------------------------
yruck0001_13030  --------------------------------------------XKERKFKLSASGKLAL
yinte0001_17980  ------------------------------------------------------------
yaldo0001_6040   ------------------------------------------------------------
yfred0001_33270  ------------------------------------------------------------
yrohd0001_18870  ------------------------------------------------------------
yruck0001_4610   -------XNKNLYRIVFNKARGMLMVVADIAASGRVTSSASSGVGHTQRRCISALSSLSF
ypest0001X_2758  ------------------------------------------------------------
ypseu0001X_2843  ------------------------------------------------------------
ypseu0001X_2846  -------XNKNLYRIIFNKVRGMMIVVADIAASGRASSSPSSGLGHTQHRRISALSTLSF
ypest0001X_2761  -------XNKNLYRIIFNKVRGMMIVVADIAASGRASSSPSSGLGHTQHRRISALSTLSF
yrohd0001_32190  ------------------------------------------------------------
ymoll0001_21160  ------------------------------------------------------------
yrohd0001_32220  ------------------------------------------------------------
yfred0001_34080  ------------------------------------------------------------
yruck0001_4650   ------------------------------------------------------------
yaldo0001_38920  ------------------------------------------------------------
yberc0001_20820  ------------------------------------------------------------
yaldo0001_3710   -------XNKNLYRIVFNKARGLLMVVADIAASGRAASSPSSGIGHTQHRRISALSPLSF
ypest0001X_8090  -----VNFGK--------------------------------------------------
yfred0001_34100  ------------------------------------------------------------
yruck0001_25350  --------------------------------------------XKNKIFKLSPAGKLAA
yfred0001_38190  ------------------------------------------------------------
yfred0001_34090  ------------------------------------------------------------
yaldo0001_30990  ------------------------------------------------------------
yberc0001_36600  ------------------------------------------------------------
yrohd0001_40100  ------------------------------------------------------------
yrohd0001_40280  ------------------------------------------------------------
yruck0001_4660   ------------------------------------------------------------
ypseu0001X_3844  ------------------------------------------------------------
ykris0001_41250  ------------------------------------------------------------
ykris0001_21250  ------------------------------------------------------------
yruck0001_4630   ------------------------------------------------------------
yrohd0001_40320  ------------------------------------------------------------
yrohd0001_40080  ------------------------------------------------------------
yrohd0001_32210  ----------------------XLMVVADIAASGRAASSASSGVGHTQSRRISALSPLSF
yfred0001_45640  -------XNKNLYRIVFNKARGMLMVVADIAASGQAASSASSGVGHTQSRRISALSPLSF
yfred0001_38220  ------------------------------------------------------------
yrohd0001_38410  -------XNKNLYRIVFNKARGMLMVVADIAASGRAASSPSTGLGHTQNHRISALSSLSF
yfred0001_33220  -------XNKNLYRIVFNKARGMLMVVADIAASGRATSSPSSGIGHAQNRRISALSSLSF
ymoll0001_36970  -----XTMNKNLYRIVFNKARGLLMVVADIAASGRAASSPSSGLGPTLSRRISALSPLSF
yrohd0001_39710  ------------------------------------------------------------
yrohd0001_18880  ------------------------------------------------------------
yinte0001_5480   -------XNKNLYRIVFNKARGLLMVVADIAASGRAASSPSSGVGHTQRRRLSTLSPLSF
yfred0001_33200  ------------------------------------------------------------
yrohd0001_18890  ------------------------------------------------------------
yfred0001_32960  ------------------------------------------------------------
yberc0001_40160  ------------------------------------------------------------
ypseu0001X_3843  ------------------------------------------------------------
ypseu0001X_3837  ------------------------------------------------------------
ypest0001X_8140  ------------------------------------------------------------
ypseu0001X_3848  -------XNKNLYRIVFNQARGMLMVVADIAASGRAASSPSSGVGHTQRRRVSALSPLSF
ypest0001X_8130  ------------------------------------------------------------
ypest0001X_8080  -------XNKNLYRIVFNQARGMLMVVADIAASGRAASSPSSGVGHTQRRRVSALSPLSF
yinte0001_41760  ----------------------------------------XKKNNEPTIKTHHQLLSYTL
ykris0001_32060  ----------------------------------------XNNNNESTIKTHHQLLSYTL
ypseu0001X_3841  ------------------------------------------------------------
yrohd0001_38400  ------------------------------------------------------------
yfred0001_40710  ------------------------------------------------------------
yente0001X_8000  ------------------------------------------------------------
yruck0001_35040  ------------------------------------------------------------
                                                                             


                         70        80        90       100       110       120
                 =========+=========+=========+=========+=========+=========+
yberc0001_8630   SLLLALGCVSLSAQANIVADGSAPGNQQPTIISSANGTPQVNIQAPSSGGVSRNVYSQFD
ykris0001_26670  ------------------------------------------------------------
ykris0001_41280  ------------------------------------------------------------
yruck0001_910    -------------------XNNATDKKDAIKLGFSDRLKNIFTHKENKLGKLYDHLNSNG
yaldo0001_37900  ------------------------------------------------------------
yberc0001_34750  GLMVPKGTIHIYDQ-------------------------------------TRNQYQPVS
yaldo0001_38900  ------------------------------------------------------------
yaldo0001_41000  ------------------------------------------------------------
ypseu0001X_3846  ------------------------------------------------------------
ymoll0001_35980  ------------------------------------------------------------
ypest0001X_8100  ------------------------------------------------------------
yfred0001_38200  ------------------------------------------------------------
ypest0001X_2754  ------------------------------------------------------------
yfred0001_34120  ------------------------------------------------------------
ypseu0001X_2842  ------------------------------------------------------------
ykris0001_7890   ------------------------------------------------------------
yruck0001_4640   ------------------------------------------------------------
ymoll0001_2720   --------------------------XLGGLVNQVQGADAPHIDTPDADLLPENA-----
yrohd0001_40310  ------------------------------------------------------------
yruck0001_34980  ------------------------------------------------------------
ypest0001X_2756  ------------------------------------------------------------
yfred0001_45620  ------------------------------------------------------------
yrohd0001_18860  ------------------------------------------------------------
yfred0001_33010  ------------------------------------------------------------
yfred0001_33210  ------------------------------------------------------------
yrohd0001_40300  ------------------------------------------------------------
yruck0001_4620   ------------------------------------------------------------
ypest0001X_8110  ------------------------------------------------------------
yberc0001_40130  ------------------------------------------------------------
yberc0001_40750  ------------------------------------------------------------
yruck0001_35050  ------------------------------------------------------------
yruck0001_13030  IITMTLSPLSLSYGSHIVPVENTANS--PTLSKHVNNSTVVNIVAPSASGLSHNQYKEFN
yinte0001_17980  ----------------------------------------VNPQKIEDSKVNYTGFTQWD
yaldo0001_6040   ------------------------------------------------------------
yfred0001_33270  ------------------------------------------------------------
yrohd0001_18870  ------------------------------------------------------------
yruck0001_4610   SLLLALGCVSLSAQANIVADARAPGNQQPTIISSANGTPQVNIQTPSSGGVSRNVYSQFD
ypest0001X_2758  ------------------------------------------------------------
ypseu0001X_2843  ------------------------------------------------------------
ypseu0001X_2846  SLLLALGCVSLSVQAAIVADASAPGNQQPTIINSANGTPQVNIQAPSSGGVSRNVYSQFD
ypest0001X_2761  SLLLALGCVSLSVQAAIVADASAPGNQQPTIINSANGTPQVNIQAPSSGGVSRNVYSQFD
yrohd0001_32190  ------------------------------------------------------------
ymoll0001_21160  ------------------------------------------------------------
yrohd0001_32220  ------------------------------------------------------------
yfred0001_34080  ------------------------------------------------------------
yruck0001_4650   ------------------------------------------------------------
yaldo0001_38920  ------------------------------------------------------------
yberc0001_20820  ------------------------------------------------------------
yaldo0001_3710   SLLLALGCVSLSAQATIVADGSAPGNQQPTIINSANGTPQVNIQTPGSGGVSRNVYSQFD
ypest0001X_8090  ------------------------------------------------------------
yfred0001_34100  ------------------------------------------------------------
yruck0001_25350  SLAIILASQGSAYTADIVGAGDSA--HQPGISNAANGAAVVNIVTPSASGLSHNQYDRFN
yfred0001_38190  ------------------------------------------------------------
yfred0001_34090  ------------------------------------------------------------
yaldo0001_30990  ------------------------------------------------------------
yberc0001_36600  ------------------------------------------------------------
yrohd0001_40100  ------------------------------------------------------------
yrohd0001_40280  ------------------------------------------------------------
yruck0001_4660   ------------------------------------------------------------
ypseu0001X_3844  ------------------------------------------------------------
ykris0001_41250  ------------------------------------------------------------
ykris0001_21250  ------------------------------------------------------------
yruck0001_4630   ------------------------------------------------------------
yrohd0001_40320  ------------------------------------------------------------
yrohd0001_40080  ------------------------------------------------------------
yrohd0001_32210  SLLLALGCVSLSAQANIVADGSAPGNQQPTIINSANGTPQVNIQTPSSDGVSRNVYSQFD
yfred0001_45640  SLLLAFGCVSLSAQANIVADGSAPTNQQPTIINSANGTPQVNIQTPSSGGVSRNVYNQFD
yfred0001_38220  ------------------------------------------------------------
yrohd0001_38410  SLLLALGCVSLSAQADIVADGSAPGNQQPTIINSANGTPQVNIQTPSSDGVSRNVYSQLD
yfred0001_33220  SLLLALGCVSLSAQANIVADASAPGNQQPTIISSANGTPQVNIQAPSSGGVSRNVYSQFD
ymoll0001_36970  SLLLALGCVSLAAQANIVADGSAPGNQQPTIISSANGTPQVNIQAPSSGGVSRNVYSQFD
yrohd0001_39710  ------------------------------------------------------------
yrohd0001_18880  ------------------------------------------------------------
yinte0001_5480   SLLLAFGCVSLSAQATIVADASAPGNQQPTIINSANGTPQVNIQTPSSGGVSRNVYSQFD
yfred0001_33200  ------------------------------------------------------------
yrohd0001_18890  ------------------------------------------------------------
yfred0001_32960  ------------------------------------------------------------
yberc0001_40160  ------------------------------------------------------------
ypseu0001X_3843  ------------------------------------------------------------
ypseu0001X_3837  ------------------------------------------------------------
ypest0001X_8140  ------------------------------------------------------------
ypseu0001X_3848  RLLIALGCISLSVQAAIVADGSAPGNQQPTIISSANGTPQVNIQTPSSGGVSRNAYRQFD
ypest0001X_8130  ------------------------------------------------------------
ypest0001X_8080  RLLIALGCISLSAQAAIVADGSAPGNQQPTIISSANGTPQVNIQTPSSGGVSRNAYRQFD
yinte0001_41760  CALLVLQPVMPALAAEVSIAGG-----NTQLDKAGNGVPVVNIATPNQSGISHNQYNDFN
ykris0001_32060  CALLVLQPVMPALAAEVSVAGG-----NTQLDKAGNGVPVVNIATPNQSGISHNQYNDFN
ypseu0001X_3841  ------------------------------------------------------------
yrohd0001_38400  ------------------------------------------------------------
yfred0001_40710  ------------------------------------------------------------
yente0001X_8000  ---------VLRSYYFILIAGNIIASIDYSVIQCWCGISPCSTEYPPTG-----------
yruck0001_35040  ------------------------------------------------------------
                                                                             


                        130       140       150       160       170       180
                 =========+=========+=========+=========+=========+=========+
yberc0001_8630   VDNRGVILNNGQG-VNQTQLGGFINGNPSLARG-EASIILNEVNSRDPSKLNGYIEVAGR
ykris0001_26670  ------------------------------------------------------------
ykris0001_41280  ------------------------------------------------------------
yruck0001_910    CEKRLVIKTNGD------------------------------------------------
yaldo0001_37900  ------------------------------------------------------------
yberc0001_34750  GIPDFITFVAGGTNLIYQAFTNMANTNTATTTRFSGEGMPIKVLMALMTRNGASFDPYIS
yaldo0001_38900  ------------------------------------------------------------
yaldo0001_41000  ------------------------------------------------------------
ypseu0001X_3846  ----------------------------------------------------------XS
ymoll0001_35980  ------------------------------------------------------------
ypest0001X_8100  ------------------------------------------------------------
yfred0001_38200  ------------------------------------------------------------
ypest0001X_2754  ------------------------------------------------------------
yfred0001_34120  ------------------------------------------------------------
ypseu0001X_2842  ------------------------------------------------------------
ykris0001_7890   ------------------------------------------------------------
yruck0001_4640   ------------------------------------------------------------
ymoll0001_2720   --------------------------------------------------------LIAD
yrohd0001_40310  ------------------------------------------------------------
yruck0001_34980  ------------------------------------------------------------
ypest0001X_2756  ------------------------------------------------------------
yfred0001_45620  ------------------------------------------------------------
yrohd0001_18860  ------------------------------------------------------------
yfred0001_33010  ------------------------------------------------------------
yfred0001_33210  ------------------------------------------------------------
yrohd0001_40300  ------------------------------------------------------------
yruck0001_4620   ------------------------------------------------------------
ypest0001X_8110  ------------------------------------------------------------
yberc0001_40130  ------------------------------------------------------------
yberc0001_40750  ------------------------------------------------------------
yruck0001_35050  ------------------------------------------------------------
yruck0001_13030  VNQQGITFNN-------LTYEAELNSNPNLINGRPATLILNEVVGVNISELQGRQQLAGS
yinte0001_17980  A-----------------------------------------------------------
yaldo0001_6040   ------------------------------------------------------------
yfred0001_33270  ------------------------------------------------------------
yrohd0001_18870  ------------------------------------------------------------
yruck0001_4610   VDHRGAILNNGHG-INQTQLGGFVNGNPLLARG-EASVILNEVNSRDPSQLNGYIEVAGR
ypest0001X_2758  ------------------------------------------------------------
ypseu0001X_2843  ------------------------------------------------------------
ypseu0001X_2846  VDGRGVILNNGHG-VNQTELGGFIDGNPWLARG-EASIILNEVNSRDPSKLNGYIEVAGR
ypest0001X_2761  VDGRGVILNNGHG-VNQTELGGFIDGNPWLARG-EASIILNEVNSRDPSKLNGYIEVAGR
yrohd0001_32190  ------------------------------------------------------------
ymoll0001_21160  ------------------------------------------------------------
yrohd0001_32220  ------------------------------------------------------------
yfred0001_34080  ------------------------------------------------------------
yruck0001_4650   ------------------------------------------------------------
yaldo0001_38920  ------------------------------------------------------------
yberc0001_20820  ------------------------------------------------------------
yaldo0001_3710   VDNRGVILNNGQG-INQTQLGGFVNGNPSLARG-EASIILNEVNSRDPSQLNGYIEVAGR
ypest0001X_8090  ------------------------------------------------------------
yfred0001_34100  ------------------------------------------------------------
yruck0001_25350  VDTPGAVLNNSLI-NGTSQLAGQLGANPNLGGA-AAKVILNEVISLNASRLLGQQEIFGM
yfred0001_38190  ------------------------------------------------------------
yfred0001_34090  ------------------------------------------------------------
yaldo0001_30990  ------------------------------------------------------------
yberc0001_36600  ------------------------------------------------------------
yrohd0001_40100  ------------------------------------------------------------
yrohd0001_40280  ------------------------------------------------------------
yruck0001_4660   ------------------------------------------------------------
ypseu0001X_3844  ------------------------------------------------------------
ykris0001_41250  ------------------------------------------------------------
ykris0001_21250  ------------------------------------------------------------
yruck0001_4630   ------------------------------------------------------------
yrohd0001_40320  ------------------------------------------------------------
yrohd0001_40080  ------------------------------------------------------------
yrohd0001_32210  VDNRGVILNNGHG-INQTQIAGVVDGNPWLARG-EANVILNEVNSRDPSKLNGYIEVAGR
yfred0001_45640  VDNRGVILNNGHG-PNQTQIAGVVDGNPWLARG-EASIILNEVNSRDPSKLNGYIEVAGR
yfred0001_38220  ------------------------------------------------------------
yrohd0001_38410  VDNRGVILNNGHG-VNQTQLGGFVNGNPALAHG-EASIILNEVNSRDPSKLNGYIEVAGR
yfred0001_33220  VDNRGVLLNNGQG-VNQTQLGGFISGNPSLARG-EASIILNEVNSRDPSRLNGYIEVAGR
ymoll0001_36970  VDSRGVILNNGHG-VNQTQLGGFINGNPSLARG-EASIILNEVNSRDPSKLNGYIEVAGR
yrohd0001_39710  ------------------------------------------------------------
yrohd0001_18880  ------------------------------------------------------------
yinte0001_5480   VDNRGVILNNGQG-VNQTQLGGFVSGNPSLARG-EASIILNEVNSRDPSRLNGYIEVAGR
yfred0001_33200  ------------------------------------------------------------
yrohd0001_18890  ------------------------------------------------------------
yfred0001_32960  ------------------------------------------------------------
yberc0001_40160  ------------------------------------------------------------
ypseu0001X_3843  ------------------------------------------------------------
ypseu0001X_3837  ------------------------------------------------------------
ypest0001X_8140  ------------------------------------------------------------
ypseu0001X_3848  VDNRGVILNNGRG-VNQTQIAGLVDGNPWLARG-EASVILNEVNSRDPSQLNGYIEVAGR
ypest0001X_8130  ------------------------------------------------------------
ypest0001X_8080  VDNRGVILNNGRG-VNQTQIAGLVDGNPWLARG-EASVILNEVNSRDPSQLNGYIEVAGR
yinte0001_41760  VGKEGLILNNATGQLTQSQLGGLIQNNPNLQAGHEAKAIINEVVGANRSQLQGYLEVAGK
ykris0001_32060  VGKEGLILNNATGQLTQSQLGGLIQNNPNLQTGHEAKAIINEVVGANRSQLQGYLEVAGK
ypseu0001X_3841  ------------------------------------------------------------
yrohd0001_38400  ------------------------------------------------------------
yfred0001_40710  ------------------------------------------------------------
yente0001X_8000  ------------------------------------------------------------
yruck0001_35040  ------------------------------------------------------------
                                                                             


                        190       200       210       220       230       240
                 =========+=========+=========+=========+=========+=========+
yberc0001_8630   KAQVVIANPAGITCEGCGF-INANRATLTTGQVLLN-NGQLTGYDVD-RGEIIIQGKGLD
ykris0001_26670  ------------------------------------------------------------
ykris0001_41280  ------------------------------------------------------------
yruck0001_910    ------------ICEKYYL-FRQDNYIFSKQQQMQKIQAFSEKIAAK-KIQLAFREHLIK
yaldo0001_37900  ------------------------------------------------------------
yberc0001_34750  KSITEMWRQCSPVAETRGF-----------------------------------DANNLK
yaldo0001_38900  ------------------------------------------------------------
yaldo0001_41000  ------------------------------------------------------------
ypseu0001X_3846  DEVVVENNALSLVLKGCGI-AAPCRSLIAKQVLEI-------------------------
ymoll0001_35980  ------------------------------------------------------------
ypest0001X_8100  ------------------------------------------------------------
yfred0001_38200  ------------------------------------------------------------
ypest0001X_2754  ------------------------------------------------------------
yfred0001_34120  ---------------------------------------------------------XLD
ypseu0001X_2842  ------------------------------------------------------------
ykris0001_7890   ------------------------------------------------------------
yruck0001_4640   ------------------------------------------------------------
ymoll0001_2720   QPLKILAKLANESTNKGEFIAVLTAEECLVQLTHNTAPTICYAYNQQVPGPLIELEAGMK
yrohd0001_40310  ------------------------------------------------------------
yruck0001_34980  ------------------------------------------------------------
ypest0001X_2756  ------------------------------------------------------------
yfred0001_45620  ------------------------------------------------------------
yrohd0001_18860  ------------------------------------------------------------
yfred0001_33010  ------------------------------------------------------------
yfred0001_33210  ------------------------------------------------------------
yrohd0001_40300  ------------------------------------------------------------
yruck0001_4620   ------------------------------------------------------------
ypest0001X_8110  ------------------------------------------------------------
yberc0001_40130  ------------------------------------------------------------
yberc0001_40750  ------------------------------------------------------------
yruck0001_35050  ------------------------------------------------------------
yruck0001_13030  PADYILANRNGINCDGCSFDPQFNQVSLAVGETIVNQGKFEKINTVDGKGSLNINASGAY
yinte0001_17980  --------------------LGGSEKDLDKKHTYIHKPSELSGGNVYINAKENVSIVASK
yaldo0001_6040   ------------------------------------------------------------
yfred0001_33270  ------------------------------------------------------------
yrohd0001_18870  ------------------------------------------------------------
yruck0001_4610   KAQVVIANPAGITCEGCGF-INANRATLTTGQAQLN-NGQITGYDVD-RGEIIVRGAGMD
ypest0001X_2758  ------------------------------------------------------------
ypseu0001X_2843  ------------------------------------------------------------
ypseu0001X_2846  KAQVVIANSAGITCEGCGF-INANRVTLTTGQAQLN-NGQLTGYDVE-RGDIVIQGTGMD
ypest0001X_2761  KAQVVIANSAGITCEGCGF-INANRVTLTTGQAQLN-NGQLTGYDVE-RGDIVIQGTGMD
yrohd0001_32190  ------------------------------------------------------------
ymoll0001_21160  ------------------------------------------------------------
yrohd0001_32220  ------------------------------------------------------------
yfred0001_34080  ------------------------------------------------------------
yruck0001_4650   ------------------------------------------------------------
yaldo0001_38920  ------------------------------------------------------------
yberc0001_20820  ------------------------------------------------------------
yaldo0001_3710   QAQVVIANPAGITCEGCGF-INANRATLTTGQAQLN-NGQLTGFDVD-RGEIVIQGKGLD
ypest0001X_8090  ------------------------------------------------------------
yfred0001_34100  ------------------------------------------------------------
yruck0001_25350  AADYVLANPNGITCDGCGF-INTNRSSLVVGNPLVE-QGNLNGFQTF-NNTNALTVGSRG
yfred0001_38190  ------------------------------------------------------------
yfred0001_34090  ------------------------------------------------------------
yaldo0001_30990  ------------------------------------------------------------
yberc0001_36600  ------------------------------------------------------------
yrohd0001_40100  ------------------------------------------------------------
yrohd0001_40280  ------------------------------------------------------------
yruck0001_4660   ------------------------------------------------------------
ypseu0001X_3844  ------------------------------------------------------------
ykris0001_41250  ------------------------------------------------------------
ykris0001_21250  ------------------------------------------------------------
yruck0001_4630   ------------------------------------------------------------
yrohd0001_40320  ------------------------------------------------------------
yrohd0001_40080  ------------------------------------------------------------
yrohd0001_32210  KAQVVIANPSGITCDGCGF-INANRATLTTGQVQLN-NGKITGYNVE-RGEIIVQGNGLD
yfred0001_45640  KAQVVIANPSGITCEGCGF-INANRATLTTGQAQLT-NGQLTGYDVE-RGEIVIQGNGLD
yfred0001_38220  ------------------------------------------------------------
yrohd0001_38410  KAQVVIANPSGITCDGCGF-INANRATLTTGQAQLN-NGQLTGYDVD-RGEIVIQGKGLD
yfred0001_33220  KAQVVIANPSGITCEGCGF-INANRATLTTGQAQLN-NGQLTGYDVD-RGEIVIQGKGLD
ymoll0001_36970  KAQVVIANPSGITCDGCGF-INANRATLTTGQAQLN-NGQLTGYDVD-RGEIIVQGAGMD
yrohd0001_39710  ------------------------------------------------------------
yrohd0001_18880  ------------------------------------------------------------
yinte0001_5480   KAQVVIANPSGITCEGCGF-INANRATLTTGQAQLN-NGQLTGYDVD-RGEIVIQGKGLD
yfred0001_33200  ------------------------------------------------------------
yrohd0001_18890  ------------------------------------------------------------
yfred0001_32960  ------------------------------------------------------------
yberc0001_40160  ------------------------------------------------------------
ypseu0001X_3843  ------------------------------------------------------------
ypseu0001X_3837  ------------------------------------------------------------
ypest0001X_8140  ------------------------------------------------------------
ypseu0001X_3848  KAQVVIANPAGITCEGCGF-INANRATLTTGQAQLN-NGQLTGYDVE-RGEIVIQGKGLD
ypest0001X_8130  ------------------------------------------------------------
ypest0001X_8080  KAQVVIANPAGITCEGCGF-INANRATLTTGQAQLN-NGQLTGYDVE-RGEIVIQGKGLD
yinte0001_41760  QASVMVANPYGITCDGCGF-INTPNVTLTTGKPIMDANGKLQALEVT-QGAISIQGKGLD
ykris0001_32060  QASVMVANPYGITCDGCGF-INTPNVTLTTGKPMMDANGKLQALEVT-QGAISIQGKGLD
ypseu0001X_3841  ------------------------------------------------------------
yrohd0001_38400  ------------------------------------------------------------
yfred0001_40710  ------------------------------------------------------------
yente0001X_8000  ------------------------------------------------------------
yruck0001_35040  ------------------------------------------------------------
                                                                             


                        250       260       270       280       290       300
                 =========+=========+=========+=========+=========+=========+
yberc0001_8630   SSRQDHTDLIARSVKVNAGIWAS-DLKVTAGRNQVDAAHQNISAKAADGSPRPTVAVDVA
ykris0001_26670  ------------------------------------------------------------
ykris0001_41280  ------------------------------------------------------------
yruck0001_910    NNRIKSSEFDENTDFIATTYNGN-KKDLGSFKHVIRKDKRFVALASNNKNKKFSMENKID
yaldo0001_37900  ------------------------------------------------------------
yberc0001_34750  TGNTTFNIVTTLSALRNPAVFTTWYSAADPGGSTVSCTTAYSNLQSAMGSPT--------
yaldo0001_38900  ------------------------------------------------------------
yaldo0001_41000  ------------------------------------------------------------
ypseu0001X_3846  ------------------------------------------------------------
ymoll0001_35980  ------------------------------------------------------------
ypest0001X_8100  ------------------------------------------------------------
yfred0001_38200  ------------------------------------------------------------
ypest0001X_2754  ------------------------------------------------------------
yfred0001_34120  KFKDAYLELLKQR-----------------------------------------------
ypseu0001X_2842  ------------------------------------------------------------
ykris0001_7890   ------------------------------------------------------------
yruck0001_4640   ------------------------------------------------------------
ymoll0001_2720   VRITFVNNLKVPSTIHWHGL----------------------------------------
yrohd0001_40310  ------------------------------------------------------------
yruck0001_34980  ------------------------------------------------------------
ypest0001X_2756  ------------------------------------------------------------
yfred0001_45620  ------------------------------------------------------------
yrohd0001_18860  ------------------------------------------------------------
yfred0001_33010  ------------------------------------------------------------
yfred0001_33210  ------------------------------------------------------------
yrohd0001_40300  ------------------------------------------------------------
yruck0001_4620   ------------------------------------------------------------
ypest0001X_8110  ------------------------------------------------------------
yberc0001_40130  ------------------------------------------------------------
yberc0001_40750  ------------------------------------------------------------
yruck0001_35050  ------------------------------------------------------------
yruck0001_13030  WGLGKNVNLIAPNINSDRDISIDGNLNIVLGHNHVNSDGNVIYSVEPKNNPY---IRDAI
yinte0001_17980  VNALNDTSIKAAGDLILAGVLNK----ITEYHNKKTGGAFNIITDSSNKENSNERFVDTE
yaldo0001_6040   ------------------------------------------------------------
yfred0001_33270  ------------------------------------------------------------
yrohd0001_18870  ------------------------------------------------------------
yruck0001_4610   SSRQDHTDLIARSVKVNAGIWAN-DLKVSAGRNQVDAAHQNINAKASDGSVRPTVAVDVA
ypest0001X_2758  ------------------------------------------------------------
ypseu0001X_2843  ------------------------------------------------------------
ypseu0001X_2846  SSRQDHTDLIARSVKVNAGIWAN-ELSVTTGRNQVDAAHQNINAKAADGSPRPTVAVDVA
ypest0001X_2761  SSRQDHTDLIARSVKVNAGIWAN-ELSVTTGRNQVDAAHQNINAKAADGSPRPTVAVDVA
yrohd0001_32190  ------------------------------------------------------------
ymoll0001_21160  ------------------------------------------------------------
yrohd0001_32220  ------------------------------------------------------------
yfred0001_34080  ------------------------------------------------------------
yruck0001_4650   ------------------------------------------------------------
yaldo0001_38920  ------------------------------------------------------------
yberc0001_20820  ------------------------------------------------------------
yaldo0001_3710   SSRQDHTDLIARSVKVNAGIWAN-DLKVTAGRNQVDAAHQNINAKAADGSVHPGLAVDVA
ypest0001X_8090  ------------------------------------------------------------
yfred0001_34100  ------------------------------------------------------------
yruck0001_25350  LTANNALDLIAPRINSKGHISSPEQIYALSGENKLSVKGAILETRNTQKAG----ALDSY
yfred0001_38190  ------------------------------------------------------------
yfred0001_34090  ------------------------------------------------------------
yaldo0001_30990  ------------------------------------------------------------
yberc0001_36600  ------------------------------------------------------------
yrohd0001_40100  ------------------------------------------------------------
yrohd0001_40280  ------------------------------------------------------------
yruck0001_4660   ------------------------------------------------------------
ypseu0001X_3844  ------------------------------------------------------------
ykris0001_41250  ------------------------------------------------------------
ykris0001_21250  ------------------------------------------------------------
yruck0001_4630   ------------------------------------------------------------
yrohd0001_40320  ------------------------------------------------------------
yrohd0001_40080  ------------------------------------------------------------
yrohd0001_32210  SSRQDSTDLIARSIKVNAGIWAN-ELNVTAGRNQVDAAHHTINANVTDGSPRPTVAVDVA
yfred0001_45640  SSRQDHTDLIARSVKVNAGIWAK-ELNVTTGRNQVDAAHQSINAKASDGSPRPTVAVDVA
yfred0001_38220  ------------------------------------------------------------
yrohd0001_38410  SSNQDHTDLIARSVKVNAGIWAN-DLKVTAGRNQIDAAHQNINAKAADGSPHPTVAVDVA
yfred0001_33220  SSRQDHTDLIARSVKVNAGIWAN-DLKVTAGRNQIDAAHQNINAKAADGSPHPTVAVDVA
ymoll0001_36970  SSRQDHTDLIARSVKVNAGIWAN-ELNVTAGRNQVDAAHQNISAKAADGSARPTVAVDVA
yrohd0001_39710  ------------------------------------------------------------
yrohd0001_18880  ------------------------------------------------------------
yinte0001_5480   SSRQDHTDLIARSVKVNAGIWAN-DLKVTAGRNQVDAAHQNITAKADDGSARPTVAVDVA
yfred0001_33200  ------------------------------------------------------------
yrohd0001_18890  ------------------------------------------------------------
yfred0001_32960  ------------------------------------------------------------
yberc0001_40160  ------------------------------------------------------------
ypseu0001X_3843  ------------------------------------------------------------
ypseu0001X_3837  ------------------------------------------------------------
ypest0001X_8140  ------------------------------------------------------------
ypseu0001X_3848  SRGQDHTDLIARSVKVNAGIWAN-ELNITTGRNQVDAAHQNINTNAADGRHRPAVAVDVA
ypest0001X_8130  ------------------------------------------------------------
ypest0001X_8080  SRGQDHTDLIARSVKVNAGIWAN-ELNITTGRNQVDAAHQNINTNAADGRHRPAVAVDVA
yinte0001_41760  ASKSGALSIISRATEINAQLYAQ-DLTLIAGSNRVDAAGNVSALQGKGDVPK--VAVDTG
ykris0001_32060  ASKSGALSIISRATEINAQLYAQ-DLTLIAGSNRVDAAGHVSALQGKGNVPK--VAVDTG
ypseu0001X_3841  ------------------------------------------------------------
yrohd0001_38400  ------------------------------------------------------------
yfred0001_40710  ------------------------------------------------------------
yente0001X_8000  ------------------------------------------------------------
yruck0001_35040  ------------------------------------------------------------
                                                                             


                        310       320       330       340       350       360
                 =========+=========+=========+=========+=========+=========+
yberc0001_8630   NLGGMYADKIRLIGTESGVGVRNAGEIGASAGDITITADGMLVNSGQINSAQHLAVNTAV
ykris0001_26670  ------------------------------------------------------------
ykris0001_41280  ------------------------------------------------------------
yruck0001_910    DLKGIKSVKFGLAVNHNLMIARNAGESLL-------------------------------
yaldo0001_37900  ------------------------------------------------------------
yberc0001_34750  ----TYDSRLKDICSKLGYNSANSASYVDCKGRMEDTLQTVY------------------
yaldo0001_38900  ------------------------------------------------------------
yaldo0001_41000  ------------------------------------------------------------
ypseu0001X_3846  ------------------------------------------------------------
ymoll0001_35980  ------------------------------------------------------------
ypest0001X_8100  ------------------------------------------------------------
yfred0001_38200  ------------------------------------------------------------
ypest0001X_2754  ------------------------------------------------------------
yfred0001_34120  ------------------------------------------------------------
ypseu0001X_2842  ------------------------------------------------------------
ykris0001_7890   ------------------------------------------------------------
yruck0001_4640   ------------------------------------------------------------
ymoll0001_2720   ------------------------------------------------------------
yrohd0001_40310  ------------------------------------------------------------
yruck0001_34980  -------------------------------------VDGQII-----------------
ypest0001X_2756  ------------------------------------------------------------
yfred0001_45620  ------------------------------------------------------------
yrohd0001_18860  ------------------------------------------------------------
yfred0001_33010  ------------------------------------------------------------
yfred0001_33210  ------------------------------------------------------------
yrohd0001_40300  ------------------------------------------------------------
yruck0001_4620   ------------------------------------------------------------
ypest0001X_8110  ------------------------------------------------------------
yberc0001_40130  ------------------------------------------------------------
yberc0001_40750  ------------------------------------------------------------
yruck0001_35050  ------------------------------------------------------------
yruck0001_13030  LAGSMNANRIRVFDNHHDRKIKLKGDNIISSNDIDIDVNSKLM-----------------
yinte0001_17980  IISG---------------------------GSVVLDSNNVFIDGALVNTGGALGVDAKG
yaldo0001_6040   ------------------------------------------------------------
yfred0001_33270  ------------------------------------------------------------
yrohd0001_18870  ------------------------------------------------------------
yruck0001_4610   SLGGMYAGKIRLIGTESGVGVRNAGEIGASAGDITITADGMLINSGQINSAQQLAVKTST
ypest0001X_2758  ------------------------------------------------------------
ypseu0001X_2843  ------------------------------------------------------------
ypseu0001X_2846  HLGGMYAGKIRLIGTESGVGVHNAGEIGASAGDITITADGMLMNSGQINSSQQLVVNTAA
ypest0001X_2761  HLGGMYAGKIRLIGTESGVGVHNAGEIGASAGDITITADGMLMNSGQINSSQQLVVNTAA
yrohd0001_32190  ------------------------------------------------------------
ymoll0001_21160  ------------------------------------------------------------
yrohd0001_32220  ------------------------------------------------------------
yfred0001_34080  ------------------------------------------------------------
yruck0001_4650   ------------------------------------------------------------
yaldo0001_38920  ------------------------------------------------------------
yberc0001_20820  ------------------------------------------------------------
yaldo0001_3710   SLGGMYAGKIRLIGTESGVGVRNAGEIGASAGEITLTASGMLINSGQINSATHLAVNTAS
ypest0001X_8090  ------------------------------------------------------------
yfred0001_34100  ------------------------------------------------------------
yruck0001_25350  YLGSMQAGRIRLISTAEGSGVNITGQLDGSK-EVTVNAKGNLGLQAARLTGDDISLS---
yfred0001_38190  ------------------------------------------------------------
yfred0001_34090  ------------------------------------------------------------
yaldo0001_30990  ------------------------------------------------------------
yberc0001_36600  ------------------------------------------------------------
yrohd0001_40100  ------------------------------------------------------------
yrohd0001_40280  ------------------------------------------------------------
yruck0001_4660   ------------------------------------------------------------
ypseu0001X_3844  ------------------------------------------------------------
ykris0001_41250  ------------------------------------------------------------
ykris0001_21250  ------------------------------------------------------------
yruck0001_4630   ------------------------------------------------------------
yrohd0001_40320  ------------------------------------------------------------
yrohd0001_40080  ------------------------------------------------------------
yrohd0001_32210  NLGGMYAGKIRLIGTESGVGVRNAGEIGASAGDITITADGMLVNSGQVNSAQQLLVNTTA
yfred0001_45640  NLGGMYAGKIRLIGTETGVGVRNAGEIGASAGDITITADGMLMNSGQINSAQHLVVKTGA
yfred0001_38220  ------------------------------------------------------------
yrohd0001_38410  SLGGMYAGKIRLIGTESGVGVRNAGEIGASAGDITITADGMLLNSGQINSARQLAVTSSA
yfred0001_33220  NLGGMYAGKIRLIGTESGVGVRNAGEIGASAGDITITADGMLVNSGQINSARQLAVKTSA
ymoll0001_36970  NLGGMYAGKIRLIGTESGVGVRNAGEIGASAGDITITADGMLMNSGQINSTQQLAVNTTA
yrohd0001_39710  ------------------------------------------------------------
yrohd0001_18880  ------------------------------------------------------------
yinte0001_5480   SLGGMYAGKIRLIGTESGVGVRNAGEIGASAGDITITADGMLVNSGQINSAQHLAVKTTT
yfred0001_33200  ------------------------------------------------------------
yrohd0001_18890  ------------------------------------------------------------
yfred0001_32960  ------------------------------------------------------------
yberc0001_40160  ------------------------------------------------------------
ypseu0001X_3843  ------------------------------------------------------------
ypseu0001X_3837  ------------------------------------------------------------
ypest0001X_8140  ------------------------------------------------------------
ypseu0001X_3848  NLGGMYAGKIRLIGTETGVGVHNAGEIGASAGDIVITADGMLVNRGQISSAQQLAVNTPS
ypest0001X_8130  ------------------------------------------------------------
ypest0001X_8080  NLGGMYAGKIRLIGTETGVGVHNAGEIGASAGDIVITADGMLVNRGQISSAQQLAVNTPS
yinte0001_41760  ALGGMYANRIRLVSSEKGVGV-NLGNLNARQGNIQLDSSGKLTLNNSLAQGSLNVSATEM
ykris0001_32060  ALGGMYANRIRLVSSEKGVGV-NLGNLNARQGDIQLDSSGKLTLNNSLAQGNLNVSATEM
ypseu0001X_3841  ------------------------------------------------------------
yrohd0001_38400  ------------------------------------------------------------
yfred0001_40710  ------------------------------------------------------------
yente0001X_8000  ------------------------------------------------------------
yruck0001_35040  ------------------------------------------------------------
                                                                             


                        370       380       390       400       410       420
                 =========+=========+=========+=========+=========+=========+
yberc0001_8630   GIENAGV----------------------------------------LYAQGNTQLTTAG
ykris0001_26670  ------------------------------------------------------------
ykris0001_41280  ------------------------------------------------------------
yruck0001_910    ------------------------------------------------------------
yaldo0001_37900  ------------------------------------------------------------
yberc0001_34750  ---------------------------------------------------GNTTLTLNA
yaldo0001_38900  ------------------------------------------------------------
yaldo0001_41000  ------------------------------------------------------------
ypseu0001X_3846  ------------------------------------------------------------
ymoll0001_35980  ------------------------------------------------------------
ypest0001X_8100  ------------------------------------------------------------
yfred0001_38200  ------------------------------------------------------------
ypest0001X_2754  ------------------------------------------------------------
yfred0001_34120  ------------------------------------------------------------
ypseu0001X_2842  ------------------------------------------------------------
ykris0001_7890   ------------------------------------------------------------
yruck0001_4640   ------------------------------------------------------------
ymoll0001_2720   ------------------------------------------------------------
yrohd0001_40310  ------------------------------------------------------------
yruck0001_34980  ------------------------------------------------------------
ypest0001X_2756  ------------------------------------------------------------
yfred0001_45620  ------------------------------------------------------------
yrohd0001_18860  ------------------------------------------------------------
yfred0001_33010  ------------------------------------------------------------
yfred0001_33210  ------------------------------------------------------------
yrohd0001_40300  ------------------------------------------------------------
yruck0001_4620   ------------------------------------------------------------
ypest0001X_8110  ------------------------------------------------------------
yberc0001_40130  ------------------------------------------------------------
yberc0001_40750  ----------------------------------------------------------LS
yruck0001_35050  ------------------------------------------------------------
yruck0001_13030  -----------------------------------------------VHSDSLHAGSTLK
yinte0001_17980  NFVVTAA------------------------------------------RQQQQSDEENS
yaldo0001_6040   ------------------------------------------------------------
yfred0001_33270  ------------------------------------------------------------
yrohd0001_18870  ------------------------------------------------------------
yruck0001_4610   GIENAGA----------------------------------------LYAQGNNQLTTAG
ypest0001X_2758  ------------------------------------------------------------
ypseu0001X_2843  ------------------------------------------------------------
ypseu0001X_2846  DIENTGV----------------------------------------LYAQGNTQLTTAG
ypest0001X_2761  DIENTGV----------------------------------------LYAQGNTQLTTAG
yrohd0001_32190  ------------------------------------------------------------
ymoll0001_21160  ------------------------------------------------------------
yrohd0001_32220  ------------------------------------------------------------
yfred0001_34080  ------------------------------------------------------------
yruck0001_4650   ------------------------------------------------------------
yaldo0001_38920  ------------------------------------------------------------
yberc0001_20820  ------------------------------------------------------------
yaldo0001_3710   DIDNSGV----------------------------------------LYAKDNTQLSTAG
ypest0001X_8090  ------------------------------------------------------------
yfred0001_34100  ------------------------------------------------------------
yruck0001_25350  ----------------------------------------------------GKNIRAEG
yfred0001_38190  ------------------------------------------------------------
yfred0001_34090  ------------------------------------------------------------
yaldo0001_30990  ------------------------------------------------------------
yberc0001_36600  ------------------------------------------------------------
yrohd0001_40100  ------------------------------------------------------------
yrohd0001_40280  ------------------------------------------------------------
yruck0001_4660   ------------------------------------------------------------
ypseu0001X_3844  ------------------------------------------------------------
ykris0001_41250  ------------------------------------------------------------
ykris0001_21250  ------------------------------------------------------------
yruck0001_4630   ------------------------------------------------------------
yrohd0001_40320  ------------------------------------------------------------
yrohd0001_40080  ------------------------------------------------------------
yrohd0001_32210  GIDNSGVLYANANTQLTTTGKLTNSGHINSAQQLLVNTTADIDNSGILYASANTQLTTTG
yfred0001_45640  EIENTGV----------------------------------------LYASANTQLTTAG
yfred0001_38220  ------------------------------------------------------------
yrohd0001_38410  NIDNSGV----------------------------------------LYASGDTQLTTAG
yfred0001_33220  DIDNSGV----------------------------------------LYASGDTQLTSAG
ymoll0001_36970  GIDNSGA----------------------------------------LYASGNTQLTTAG
yrohd0001_39710  ------------------------------------------------------------
yrohd0001_18880  ------------------------------------------------------------
yinte0001_5480   GIANAGV----------------------------------------LYAQGNTQLTTAG
yfred0001_33200  ------------------------------------------------------------
yrohd0001_18890  ------------------------------------------------------------
yfred0001_32960  ------------------------------------------------------------
yberc0001_40160  ------------------------------------------------------------
ypseu0001X_3843  ------------------------------------------------------------
ypseu0001X_3837  ------------------------------------------------------------
ypest0001X_8140  ------------------------------------------------------------
ypseu0001X_3848  GIENSGV----------------------------------------LYGKGNTQLTTAG
ypest0001X_8130  ------------------------------------------------------------
ypest0001X_8080  GIENSGV----------------------------------------LYGKGNTQLTTAG
yinte0001_41760  TLGGSHK---------------------AGQDMVLNSQGKLAVNNASLNSDQQVVLTGGD
ykris0001_32060  ALGGSHK---------------------SGQDMVLNSQGKLTVNNASLNSDQQLALKGSD
ypseu0001X_3841  ------------------------------------------------------------
yrohd0001_38400  ------------------------------------------------------------
yfred0001_40710  ------------------------------------------------------------
yente0001X_8000  ------------------------------------------------------------
yruck0001_35040  ------------------------------------------------------------
                                                                             


                        430       440       450       460       470       480
                 =========+=========+=========+=========+=========+=========+
yberc0001_8630   KLSNSGTIAAAGDTSLRAA-EVNSSRDSVLGAGVKSDNSTITSGTLRIDASGKLTAQGKN
ykris0001_26670  ------------------------------------------------------------
ykris0001_41280  ------------------------------------------------------------
yruck0001_910    ------------------------------------------------------------
yaldo0001_37900  ------------------------------------------------------------
yberc0001_34750  AIGGVMVSQAISDALVQQNPEV---------AGTMLANRSMVNSALSDASTNPEWLTTIM
yaldo0001_38900  ------------------------------------------------------------
yaldo0001_41000  ------------------------------------------------------------
ypseu0001X_3846  ------------------------------------------------------------
ymoll0001_35980  ------------------------------------------------------------
ypest0001X_8100  ------------------------------------------------------------
yfred0001_38200  ------------------------------------------------------------
ypest0001X_2754  ------------------------------------------------------------
yfred0001_34120  ------------------------------------------------------------
ypseu0001X_2842  ------------------------------------------------------------
ykris0001_7890   -------------------------------------------XRKYLIGKGVLSGHEKT
yruck0001_4640   ---------------------------------LKSDGNRASSGDLTVSTEQGLVAEGQN
ymoll0001_2720   --------------------PVPPNQDGGPDDPVAPGTTKVYEFQLPDSLSGTYWYHPHP
yrohd0001_40310  ------------------------------------------------------------
yruck0001_34980  ------------------------------------------------------------
ypest0001X_2756  ------------------------------------------------------------
yfred0001_45620  ------------------------------------------------------------
yrohd0001_18860  ------------------------------------------------------------
yfred0001_33010  ------------------------------------------------------------
yfred0001_33210  ------------------------------------------------------------
yrohd0001_40300  ------------------------------------------------------------
yruck0001_4620   ------------------------------------------------------------
ypest0001X_8110  ------------------------------------------------------------
yberc0001_40130  -VGAKWDAISLGQDTAYSA-GMLVGVPQGLYDSVESLSKSISDPAATYDAIKQLIASDDI
yberc0001_40750  RIGDSGFGQVLGNASM--------------------------------------------
yruck0001_35050  ------------------------------------------------------------
yruck0001_13030  IKANNINVANNVESPFRNN-QVELTDISAGENIVFSANGNINLTSVNIKAKKDVSLNGGE
yinte0001_17980  RLSWEWFANKQKDKQFRAGFEIKHVNNKEKNSKTKRHFATLTANKININADKDIRFYGTA
yaldo0001_6040   ------------------------------------------------------------
yfred0001_33270  ------------------------------------------------------------
yrohd0001_18870  ------------------------------------------------------------
yruck0001_4610   TLSNSGTIAAAGDTSLRAA-EVNSSRNSVLGAGVKSDNSAITSGTLRVDASGMLTAQGKN
ypest0001X_2758  ------------------------------------------------------------
ypseu0001X_2843  ------------------------------------------------------------
ypseu0001X_2846  TLSNSGTLAAGGDTSVRAA-EVNSTRNSVLGAGVKSDNSAITSGTLSVEASGKITAQGKN
ypest0001X_2761  TLSNSGTLAAGGDTSVRAA-EVNSTRNSVLGAGVKSDNSAITSGTLSVEASGKITAQGKN
yrohd0001_32190  ------------------------------------------------------------
ymoll0001_21160  ------------------------------------------------------------
yrohd0001_32220  ------------------------------------------------------------
yfred0001_34080  ------------------------------------------------------------
yruck0001_4650   ------------------------------------------------------------
yaldo0001_38920  ------------------------------------------------------------
yberc0001_20820  ------------------------------------------------------------
yaldo0001_3710   KLSNSGTIAAAGDTTIRAA-EVNSSRNSVLGAGVKTDNSAISSGTLRIDASGQLIAHGKN
ypest0001X_8090  ------------------------------------------------------------
yfred0001_34100  ------------------------------------------------------------
yruck0001_25350  QVTNSSTNQSENDNYDGWW-SGKYVNNR--STSQKLDRTTLKGKNISLEASDSNHLTATD
yfred0001_38190  ------------------------------------------------------------
yfred0001_34090  ------------------------------------------------------------
yaldo0001_30990  ------------------------------------------------------------
yberc0001_36600  ------------------------------------------------------------
yrohd0001_40100  ------------------------------------------------------------
yrohd0001_40280  ------------------------------------------------------------
yruck0001_4660   ------------------------------------------------------------
ypseu0001X_3844  ------------------------------------------------------------
ykris0001_41250  ------------------------------------------------------------
ykris0001_21250  ------------------------------------------------------------
yruck0001_4630   ------------------------------------------------------------
yrohd0001_40320  ------------------------------------------------------------
yrohd0001_40080  ------------------------------------------------------------
yrohd0001_32210  KLTNSGTVAAAGDTSLRAT-EINSSRSSVLGAGVKSDNSAMTSGTLNVTASGQLTAQGKN
yfred0001_45640  KLTNSGTIAAAGDTSLRAA-EVNSSRNAVLGAGVKSDNSSITSGTLKVEASGKLIAQGKN
yfred0001_38220  ------------------------------------------------------------
yrohd0001_38410  KLSNSGTIAAAGDTTLRAA-EVNSSRNSMLGAGIKSDNSDITSGTLSIEANGQLIAQGKN
yfred0001_33220  KLSNSGTIAAAGDTALRAA-EVNSSRNSLLGAGVKSDNSSVTSGTLSIEANGQLIAQGKN
ymoll0001_36970  KLSNSGTIAAAGDTSIRAA-EVNSSRNSVLGAGVKSDNSSITSGTLRVEASGKLLAQGKN
yrohd0001_39710  ------------------------------------------------------------
yrohd0001_18880  ------------------------------------------------------------
yinte0001_5480   TLNNTGTVAAAGDTTLRAA-EVNSSRNSVLGAGVKSDNSSVTRGTLSIDASGQLIAQGKN
yfred0001_33200  ------------------------------------------------------------
yrohd0001_18890  ------------------------------------------------------------
yfred0001_32960  ------------------------------------------------------------
yberc0001_40160  ------------------------------------------------------------
ypseu0001X_3843  ------------------------------------------------------------
ypseu0001X_3837  ------------------------------------------------------------
ypest0001X_8140  ------------------------------------------------------------
ypseu0001X_3848  KLSNSGTVAAAGDTLIRAA-EVNSSRNSVLGAGIKSDNSVITRGTLDIKARGQLTAQGKN
ypest0001X_8130  ------------------------------------------------------------
ypest0001X_8080  KLSNSGTVAAAGDTLIRAA-EVNSSRNSVLGAGIKSDNSAITRGTLDIKARGQLTAQGKN
yinte0001_41760  LALEQSTLSAVKAMTLDSA-GKLRAANSTLLAGRDDQGKLVSGQTLTLIGTEQQWLNSQL
ykris0001_32060  LVLEQSTISAAKAVTLDSA-GKLRTANSTILAGSDVQGKLVGGQTLTLKGTEQQWLNSQL
ypseu0001X_3841  ------------------------------------------------------------
yrohd0001_38400  ------------------------------------------------------------
yfred0001_40710  ------------------------------------------------------------
yente0001X_8000  ------------------------------------------------------------
yruck0001_35040  ------------------------------------------------------------
                                                                             


                        490       500       510       520       530       540
                 =========+=========+=========+=========+=========+=========+
yberc0001_8630   VSGTAQLLNAHSIDLSGSQTESRDLTLSAQGGPIDLSGAKLSASQRLSAATASLLRTDNA
ykris0001_26670  ------------------------------------------------------------
ykris0001_41280  ------------------------------------------------------------
yruck0001_910    ------------------------------------------------------------
yaldo0001_37900  ------------XSITSHNTTAKDRDFTKEISKLSKSLDKYNEQAKIIEKNKTEIQ----
yberc0001_34750  AGVMAIILSMTPMLILLVVTPLMGKALTLLLGMWIFLTAWQVADTLLLQASTDEILTVMS
yaldo0001_38900  ------XYDERREALKGTTSF---------------------------------------
yaldo0001_41000  ----------------------XLVPPPPVAGQTPQSEANAAIAQKLDT-----------
ypseu0001X_3846  ------------------------------------------------------------
ymoll0001_35980  ------------------------------------------------------------
ypest0001X_8100  ------------------------------------------------------------
yfred0001_38200  ------------------------------------------------------------
ypest0001X_2754  ------------------------------------------------------------
yfred0001_34120  ------------------------------------------------------------
ypseu0001X_2842  --------------------------------XIDMTPIDYFKNVFLKM-----------
ykris0001_7890   LLNAAKMYGVNAIYLAVHASLETGNGTSPLGTGITVDGVMVY------------------
yruck0001_4640   IAAGQVALSGRDIDLTGSQTQGHAISLVAQSGDITLTDAVVNAATTLSARTAARXLIN--
ymoll0001_2720   LKGTAEQFAKGL------------------------------------------------
yrohd0001_40310  ------------------------------------------------------------
yruck0001_34980  ------------------------------------------------------------
ypest0001X_2756  ------------------------------------------------------------
yfred0001_45620  ------------------------------------------------------------
yrohd0001_18860  ------------------------------------------------------------
yfred0001_33010  ------------------------------------------------------------
yfred0001_33210  ------------------------------------------------------------
yrohd0001_40300  ------------------------------------------------------------
yruck0001_4620   ------------------------------------------------------------
ypest0001X_8110  ------------------------------------------------------------
yberc0001_40130  FSTMSDAVRQSYIDRIN-------------------------------------------
yberc0001_40750  ------------------------------------------------------------
yruck0001_35050  ------------------------------------------------------------
yruck0001_13030  INITAGIKSSSTDDYDREYTIGFTNIYSTSSTSIDKVVKNSINTD---------------
yinte0001_17980  I-----------------KTNQGDLALSAKEGIQFLAAIDSHLTDK--------------
yaldo0001_6040   ------------------------------------------------------------
yfred0001_33270  ------------------------------------------------------------
yrohd0001_18870  ------------------------------------------------------------
yruck0001_4610   ISGTALALNARSLDLGGSQTQSRDLTLTAQSGDIDLTGANLSASHHLSVSTTDXLIN---
ypest0001X_2758  ------------------------------------------------------------
ypseu0001X_2843  ------------------------------------------------------------
ypseu0001X_2846  ISGTAQRFTAHRLDLSGSQTQSRDITLTAQGGEIDLTGAELLASDRLSAATTALLRTDNA
ypest0001X_2761  ISGTAQRFTAHRLDLSGSQTQSRDITLIAQGGEIDLTGAELLASDRLSAATTALLRTDNA
yrohd0001_32190  ------------------------------------------------------------
ymoll0001_21160  ------------------------------------------------------------
yrohd0001_32220  ------------------------------------------------------------
yfred0001_34080  ------------------------------------------------------------
yruck0001_4650   ------------------------------------------------------------
yaldo0001_38920  ------------------------------------------------------------
yberc0001_20820  ------------------------------------------------------------
yaldo0001_3710   VSGTAQNLNAHSIDLGGSQTQSRDLTLTAQGGAIDLSGATLSASERLSASTASALRTDNA
ypest0001X_8090  ------------------------------------------------------------
yfred0001_34100  ---------------------VQDITLR--------------------------------
yruck0001_25350  INANNVRLNGGSIVLDGQQLDQTQAHTDNRNSGAWIRNVTTEKSQRQQVATTINVKDNIA
yfred0001_38190  ------------------------------------------------------------
yfred0001_34090  ------------------------------------------------------------
yaldo0001_30990  ------------------------------------------------------------
yberc0001_36600  ------------------------------------------------------------
yrohd0001_40100  ------------------------------------------------------------
yrohd0001_40280  ------------------------------------------------------------
yruck0001_4660   ------------------------------------------------------------
ypseu0001X_3844  ------------------------------------------------------------
ykris0001_41250  ------------------------------------------------------------
ykris0001_21250  ------------------------------------------------------------
yruck0001_4630   ------------------------------------------------------------
yrohd0001_40320  ------------------------------------------------------------
yrohd0001_40080  ------------------------------------------------------------
yrohd0001_32210  LSGTAQNFSARAIDLSGSQTQSRDLTLTAQDGAISLTAANLSASQYLSLSTASLLRTDNA
yfred0001_45640  ISGTAQSFNAHSIDLSGSQTQSRDVALTAQSGDINLTGAEVLASQSLSASTTSVLYTNNA
yfred0001_38220  ------------------------------------------------------------
yrohd0001_38410  VSGTAQNLNAHSIDLSGSQTQSRDITLTSQGGPIDLSGARLSASQNLSASTASQLRTDKA
yfred0001_33220  VSGTAQNLNAHSIDLSGSQTQSRDITLTSQGGPIDLSGARLSASQNLSASTASQLRTDNA
ymoll0001_36970  ISGTAQNFKAQSLDLSGSQTQSRDITLSTQGGEIDLTGANLSASQRLSASTASLLRTDNA
yrohd0001_39710  ------------------------------------------------------------
yrohd0001_18880  ------------------------------------------------------------
yinte0001_5480   VSGTAQNLNAHSIDLSGSQTQSRDITLTTQGGAIDLTGANLSASQHLSASTAAQLRTDSA
yfred0001_33200  ------------------------------------------------------------
yrohd0001_18890  ------------------------------------------------------------
yfred0001_32960  ------------------------------------------------------------
yberc0001_40160  ------------------------------------------------------------
ypseu0001X_3843  ------------------------------------------------------------
ypseu0001X_3837  ------------------------------------------------------------
ypest0001X_8140  ------------------------------------------------------------
ypseu0001X_3848  ISGTAQTFNANRIDLSGSQTQSGDLTFTTEGGDIDLTGANLFANRRLSVSTPSLLRTDKA
ypest0001X_8130  ------------------------------------------------------------
ypest0001X_8080  ISGTAQTFNANRIDLSGSQTQSGDLTFTTEGGDIDLTGANLFANRRLSVSTPSLLRTDKA
yinte0001_41760  GAGTVLAAAQNNLILDGSSTLTGLDGVTLQGGTLNLAGKTSSGGDVTLQGTDLQSTRNSQ
ykris0001_32060  GAGTVLATAQNNLILDEGSTLTGLDGVTLQGGTLNLAGKTSSGGDATLQGTDLQSTRNSL
ypseu0001X_3841  ------------------------------------------------------------
yrohd0001_38400  ------------------------------------------------------------
yfred0001_40710  ------------------------------------------------------------
yente0001X_8000  ------------------------------------------------------------
yruck0001_35040  ------------------------------------------------------------
                                                                             


                        550       560       570       580       590       600
                 =========+=========+=========+=========+=========+=========+
yberc0001_8630   RLIGEKITLDAQSLSNVGGVIA----------------------QTGTTDLNLNLPGYID
ykris0001_26670  ------------------------------------------------------------
ykris0001_41280  ------------------------------------------------------------
yruck0001_910    ------------------------------------------------------------
yaldo0001_37900  ------------------------------------------------------------
yberc0001_34750  EVKSMGFGLDAMQLAPTSAQKA--------------------------------------
yaldo0001_38900  ------------------------------------------------------------
yaldo0001_41000  ------------------------------------------------------------
ypseu0001X_3846  ------------------------------------------------------------
ymoll0001_35980  ------------------------------------------------------------
ypest0001X_8100  ------------------------------------------------------------
yfred0001_38200  ------------------------------------------------------------
ypest0001X_2754  ------------------------------------------------------------
yfred0001_34120  ------------------------------------------------------------
ypseu0001X_2842  ------------------------------------------------------------
ykris0001_7890   ------------------------------------------------------------
yruck0001_4640   ------------------------------------------------------------
ymoll0001_2720   ------------------------------------------------------------
yrohd0001_40310  ------------------------------------------------------------
yruck0001_34980  --------------------------------------------QTGRGDFHLNLPGEL-
ypest0001X_2756  ------------------------------------------------------------
yfred0001_45620  ------------------------------------------------------------
yrohd0001_18860  ------------------------------------------------------------
yfred0001_33010  ------------------------------------------------------------
yfred0001_33210  ------------------------------------------------------------
yrohd0001_40300  ------------------------------------------------------------
yruck0001_4620   ------------------------------------------------------------
ypest0001X_8110  ------------------------------------------------------------
yberc0001_40130  ------------------------------------------------------------
yberc0001_40750  ------------------------------------------------------------
yruck0001_35050  ------------------------------------------------------------
yruck0001_13030  ------------------------------------------------------------
yinte0001_17980  ------------------------------------------------------------
yaldo0001_6040   ------------------------------------------------------------
yfred0001_33270  ------------------------------------------------------------
yrohd0001_18870  ------------------------------------------------------------
yruck0001_4610   ------------------------------------------------------------
ypest0001X_2758  ------------------------------------------------------------
ypseu0001X_2843  ------------------------------------------------------------
ypseu0001X_2846  SLIAEQITLDAQALSNVGGLIA----------------------HTGTTDFNLNLP----
ypest0001X_2761  SLIAEQITLDAQALSNVGGLIA----------------------HTGTTDFNLNLP----
yrohd0001_32190  ------------------------------------------------------------
ymoll0001_21160  ------------------------------------------------------------
yrohd0001_32220  ------------------------------------------------------------
yfred0001_34080  ------------------------------------------------------------
yruck0001_4650   ------------------------------------------------------------
yaldo0001_38920  ------------------------------------------------------------
yberc0001_20820  ------------------------------------------------------------
yaldo0001_3710   NLVADQVTLSAQSLSNVGGVIA----------------------QTGTTDFNLNLPGYLD
ypest0001X_8090  ------------------------------------------------------------
yfred0001_34100  ------------------------------------------------------------
yruck0001_25350  LNASKGDISLQNASLNAGKELS--------------------------------------
yfred0001_38190  ------------------------------------------------------------
yfred0001_34090  ------------------------------------------------------------
yaldo0001_30990  ------------------------------------------------------------
yberc0001_36600  ------------------------------------------------------------
yrohd0001_40100  ------------------------------------------------------------
yrohd0001_40280  ------------------------------------------------------------
yruck0001_4660   ------------------------------------------------------------
ypseu0001X_3844  ------------------------------------------------------------
ykris0001_41250  ------------------------------------------------------------
ykris0001_21250  ------------------------------------------------------------
yruck0001_4630   ------------------------------------------------------------
yrohd0001_40320  ------------------------------------------------------------
yrohd0001_40080  ------------------------------------------------------------
yrohd0001_32210  SVIAEQITLEAQALSNVGGVIA----------------------QVGAADFKLNLT----
yfred0001_45640  RLIADKITFNAQTLSNVGGVIA----------------------QTGTTDFNLNLP----
yfred0001_38220  ------------------------------------------------------------
yrohd0001_38410  NLVAGQVTLDAQTLSNVGGVIA----------------------QTGTTDFNLNLPGNID
yfred0001_33220  KLVAGQVTLDAQALSNVRGVIA----------------------QTGTTDFNLNLPGNID
ymoll0001_36970  SLIAEQITLEAQALSNRGGVIA----------------------QTGTTDFNLHLT----
yrohd0001_39710  ------------------------------------------------------------
yrohd0001_18880  ------------------------------------------------------------
yinte0001_5480   RLIADQVTLNAQSLSNVGGVIA----------------------QTGTTDFNLNLPGDID
yfred0001_33200  ------------------------------------------------------------
yrohd0001_18890  ------------------------------------------------------------
yfred0001_32960  ------------------------------------------------------------
yberc0001_40160  ------------------------------------------------------------
ypseu0001X_3843  ------------------------------------------------------------
ypseu0001X_3837  ------------------------------------------------------------
ypest0001X_8140  ------------------------------------------------------------
ypseu0001X_3848  NLFAEQIALDAQALANVGGVIT----------------------QTGLTDFNLNLPGYID
ypest0001X_8130  ------------------------------------------------------------
ypest0001X_8080  NLFAEQIALDAQALANVGGVIT----------------------QTGLTDFNLNLPGDID
yinte0001_41760  FNAQGDIHLTFSGNADWQGQLTAGRDLTLQTATLDNSGQLAANRHSQITTQSLNNSGLMQ
ykris0001_32060  FSAQGDIHLTFSGNANWQGQLTAGRDLTLQADTLDNSGQLAANRHNQITAQSLNNSGLMQ
ypseu0001X_3841  ------------------------------------------------------------
yrohd0001_38400  ------------------------------------------------------------
yfred0001_40710  ------------------------------------------------------------
yente0001X_8000  ------------------------------------------------------------
yruck0001_35040  ------------------------------------------------------------
                                                                             


                        610       620       630       640       650       660
                 =========+=========+=========+=========+=========+=========+
yberc0001_8630   NRDGKILSSGTLALQAESLNSSGNSLLGAGVQSDGKLAQSGELNVATRQALIA-------
ykris0001_26670  ------------------------------------------------------------
ykris0001_41280  ------------------------------------------------------------
yruck0001_910    ------------------------------------------------------------
yaldo0001_37900  ------------------------------------------------------------
yberc0001_34750  ------------------------------------------------------------
yaldo0001_38900  ------------------------------------------------------------
yaldo0001_41000  ------------------------------------------------------------
ypseu0001X_3846  ------------------------------------------------------------
ymoll0001_35980  ------------------------------------------------------------
ypest0001X_8100  ------------------------------------------------------------
yfred0001_38200  ------------------------------------------------------------
ypest0001X_2754  ------------------------------------------------------------
yfred0001_34120  ------------------------------------------------------------
ypseu0001X_2842  ------------------------------------------------------------
ykris0001_7890   ------------------------------------------------------------
yruck0001_4640   ------------------------------------------------------------
ymoll0001_2720   ------------------------------------------------------------
yrohd0001_40310  ------------------------------------------------------------
yruck0001_34980  ------------------------------------------------------------
ypest0001X_2756  ------------------------------------------------------------
yfred0001_45620  ------------------------------------------------------------
yrohd0001_18860  ------------------------------------------------------------
yfred0001_33010  ------------------------------------------------------------
yfred0001_33210  ------------------------------------------------------------
yrohd0001_40300  ------------------------------------------------------------
yruck0001_4620   ------------------------------------------------------------
ypest0001X_8110  ------------------------------------------------------------
yberc0001_40130  ------------------------------------------------------------
yberc0001_40750  ------------------------------------------------------------
yruck0001_35050  ------------------------------------------------------------
yruck0001_13030  ------------------------------------------------------------
yinte0001_17980  ------------------------------------------------------------
yaldo0001_6040   ------------------------------------------------------------
yfred0001_33270  ------------------------------------------------------------
yrohd0001_18870  ------------------------------------------------------------
yruck0001_4610   ------------------------------------------------------------
ypest0001X_2758  ------------------------------------------------------------
ypseu0001X_2843  ------------------------------------------------------------
ypseu0001X_2846  ------------------------------------------------------------
ypest0001X_2761  ------------------------------------------------------------
yrohd0001_32190  ------------------------------------------------------------
ymoll0001_21160  ------------------------------------------------------------
yrohd0001_32220  ------------------------------------------------------------
yfred0001_34080  ------------------------------------------------------------
yruck0001_4650   ------------------------------------------------------------
yaldo0001_38920  ------------------------------------------------------------
yberc0001_20820  ------------------------------------------------------------
yaldo0001_3710   NRGGTLLSKGNVAVQAERLDSDSGSLLGAGVQRDGKLAPSGELNVATRQALIA-------
ypest0001X_8090  ------------------------------------------------------------
yfred0001_34100  ------------------------------------------------------------
yruck0001_25350  ------------------------------------------------------------
yfred0001_38190  ------------------------------------------------------------
yfred0001_34090  ------------------------------------------------------------
yaldo0001_30990  ------------------------------------------------------------
yberc0001_36600  ------------------------------------------------------------
yrohd0001_40100  ------------------------------------------------------------
yrohd0001_40280  ------------------------------------------------------------
yruck0001_4660   ------------------------------------------------------------
ypseu0001X_3844  ------------------------------------------------------------
ykris0001_41250  ------------------------------------------------------------
ykris0001_21250  ------------------------------------------------------------
yruck0001_4630   ------------------------------------------------------------
yrohd0001_40320  ------------------------------------------------------------
yrohd0001_40080  ------------------------------------------------------------
yrohd0001_32210  ------------------------------------------------------------
yfred0001_45640  ------------------------------------------------------------
yfred0001_38220  ------------------------------------------------------------
yrohd0001_38410  NRDGKILSGGKLSLQAETLNSNGNSLLGAGVQSDGKLAQSGELNIATRQALIA-------
yfred0001_33220  NRDGKILSGGKLSLQAETLNSNGNSLLGAGVQNDGKLAQSGELNVATRQALIA-------
ymoll0001_36970  ------------------------------------------------------------
yrohd0001_39710  ------------------------------------------------------------
yrohd0001_18880  ------------------------------------------------------------
yinte0001_5480   NRGGTLLSGGKLSLQAENLNSNGNSLLGAGVQSDGKLAQSGSLNVATRQALIA-------
yfred0001_33200  ------------------------------------------------------------
yrohd0001_18890  ------------------------------------------------------------
yfred0001_32960  ------------------------------------------------------------
yberc0001_40160  ------------------------------------------------------------
ypseu0001X_3843  ------------------------------------------------------------
ypseu0001X_3837  ------------------------------------------------------------
ypest0001X_8140  ------------------------------------------------------------
ypseu0001X_3848  NRGGSLLTRGNFLLQAERLTSNSQSLLGAGIQSDGKLAPRGDLNVTTRHALIAQGKTLAA
ypest0001X_8130  ------------------------------------------------------------
ypest0001X_8080  NRDGTLLTRGNFLLQAEHLTSNSQSLLGAGIQSDGKLAPRGDLNVTTRHALIAQGKTLTA
yinte0001_41760  AQGSQNLDVGQLDNNGQLQSAGTLTLNANTVNNRGLIGSQQQLALTVRDTLNV-------
ykris0001_32060  AQGSQNLDVRQLDNRGQLQSAGALTLNADTVNNRGLIGSEQQLTLTVRDTLNV-------
ypseu0001X_3841  ------------------------------------------------------------
yrohd0001_38400  ------------------------------------------------------------
yfred0001_40710  ------------------------------------------------------------
yente0001X_8000  ------------------------------------------------------------
yruck0001_35040  ------------------------------------------------------------
                                                                             


                        670       680       690       700       710       720
                 =========+=========+=========+=========+=========+=========+
yberc0001_8630   ------------------------------------------------------------
ykris0001_26670  ------------------------------------------------------------
ykris0001_41280  ------------------------------------------------------------
yruck0001_910    ------------------------------------------------------------
yaldo0001_37900  ------------------------------------------------------------
yberc0001_34750  ------------------------------------------------------------
yaldo0001_38900  ------------------------------------------------------------
yaldo0001_41000  ------------------------------------------------------------
ypseu0001X_3846  ------------------------------------------------------------
ymoll0001_35980  ------------------------------------------------------------
ypest0001X_8100  ------------------------------------------------------------
yfred0001_38200  ------------------------------------------------------------
ypest0001X_2754  ------------------------------------------------------------
yfred0001_34120  ------------------------------------------------------------
ypseu0001X_2842  ------------------------------------------------------------
ykris0001_7890   ------------------------------------------------------------
yruck0001_4640   ------------------------------------------------------------
ymoll0001_2720   ------------------------------------------------------------
yrohd0001_40310  ------------------------------------------------------------
yruck0001_34980  ------------------------------------------------------------
ypest0001X_2756  ------------------------------------------------------------
yfred0001_45620  ------------------------------------------------------------
yrohd0001_18860  ------------------------------------------------------------
yfred0001_33010  ------------------------------------------------------------
yfred0001_33210  ------------------------------------------------------------
yrohd0001_40300  ------------------------------------------------------------
yruck0001_4620   ------------------------------------------------------------
ypest0001X_8110  ------------------------------------------------------------
yberc0001_40130  ------------------------------------------------------------
yberc0001_40750  ------------------------------------------------------------
yruck0001_35050  ------------------------------------------------------------
yruck0001_13030  ------------------------------------------------------------
yinte0001_17980  ------------------------------------------------------------
yaldo0001_6040   ------------------------------------------------------------
yfred0001_33270  ------------------------------------------------------------
yrohd0001_18870  ------------------------------------------------------------
yruck0001_4610   ------------------------------------------------------------
ypest0001X_2758  ------------------------------------------------------------
ypseu0001X_2843  ------------------------------------------------------------
ypseu0001X_2846  ------------------------------------------------------------
ypest0001X_2761  ------------------------------------------------------------
yrohd0001_32190  ------------------------------------------------------------
ymoll0001_21160  ------------------------------------------------------------
yrohd0001_32220  ------------------------------------------------------------
yfred0001_34080  ------------------------------------------------------------
yruck0001_4650   ------------------------------------------------------------
yaldo0001_38920  ------------------------------------------------------------
yberc0001_20820  ------------------------------------------------------------
yaldo0001_3710   ------------------------------------------------------------
ypest0001X_8090  ------------------------------------------------------------
yfred0001_34100  ------------------------------------------------------------
yruck0001_25350  ------------------------------------------------------------
yfred0001_38190  ------------------------------------------------------------
yfred0001_34090  ------------------------------------------------------------
yaldo0001_30990  ------------------------------------------------------------
yberc0001_36600  ------------------------------------------------------------
yrohd0001_40100  ------------------------------------------------------------
yrohd0001_40280  ------------------------------------------------------------
yruck0001_4660   ------------------------------------------------------------
ypseu0001X_3844  ------------------------------------------------------------
ykris0001_41250  ------------------------------------------------------------
ykris0001_21250  ------------------------------------------------------------
yruck0001_4630   ------------------------------------------------------------
yrohd0001_40320  ------------------------------------------------------------
yrohd0001_40080  ------------------------------------------------------------
yrohd0001_32210  ------------------------------------------------------------
yfred0001_45640  ------------------------------------------------------------
yfred0001_38220  ------------------------------------------------------------
yrohd0001_38410  ------------------------------------------------------------
yfred0001_33220  ------------------------------------------------------------
ymoll0001_36970  ------------------------------------------------------------
yrohd0001_39710  ------------------------------------------------------------
yrohd0001_18880  ------------------------------------------------------------
yinte0001_5480   ------------------------------------------------------------
yfred0001_33200  ------------------------------------------------------------
yrohd0001_18890  ------------------------------------------------------------
yfred0001_32960  ------------------------------------------------------------
yberc0001_40160  ------------------------------------------------------------
ypseu0001X_3843  ------------------------------------------------------------
ypseu0001X_3837  ------------------------------------------------------------
ypest0001X_8140  ------------------------------------------------------------
ypseu0001X_3848  GTLALSGSRLDLTDSLTQAKDMRLTATEGDIALTGATVMAANTLFADTRQILRSDKAYLT
ypest0001X_8130  ------------------------------------------------------------
ypest0001X_8080  GTLALSGSRLDLTDSLTQAKYMRLTATEGDIALTGATVMAANTLFADTRQILRSDKAYLT
yinte0001_41760  ------------------------------------------------------------
ykris0001_32060  ------------------------------------------------------------
ypseu0001X_3841  ------------------------------------------------------------
yrohd0001_38400  ------------------------------------------------------------
yfred0001_40710  ------------------------------------------------------------
yente0001X_8000  ------------------------------------------------------------
yruck0001_35040  ------------------------------------------------------------
                                                                             


                        730       740       750       760       770       780
                 =========+=========+=========+=========+=========+=========+
yberc0001_8630   ------------------------------------------------------------
ykris0001_26670  ------------------------------------------------------------
ykris0001_41280  ------------------------------------------------------------
yruck0001_910    ------------------------------------------------------------
yaldo0001_37900  ------------------------------------------------------------
yberc0001_34750  ------------------------------------------------------------
yaldo0001_38900  ------------------------------------------------------------
yaldo0001_41000  ------------------------------------------------------------
ypseu0001X_3846  ------------------------------------------------------------
ymoll0001_35980  ------------------------------------------------------------
ypest0001X_8100  ------------------------------------------------------------
yfred0001_38200  ------------------------------------------------------------
ypest0001X_2754  ------------------------------------------------------------
yfred0001_34120  ------------------------------------------------------------
ypseu0001X_2842  ------------------------------------------------------------
ykris0001_7890   ------------------------------------------------------------
yruck0001_4640   ------------------------------------------------------------
ymoll0001_2720   ------------------------------------------------------------
yrohd0001_40310  ------------------------------------------------------------
yruck0001_34980  ------------------------------------------------------------
ypest0001X_2756  ------------------------------------------------------------
yfred0001_45620  ------------------------------------------------------------
yrohd0001_18860  ------------------------------------------------------------
yfred0001_33010  ------------------------------------------------------------
yfred0001_33210  ------------------------------------------------------------
yrohd0001_40300  ------------------------------------------------------------
yruck0001_4620   ------------------------------------------------------------
ypest0001X_8110  ------------------------------------------------------------
yberc0001_40130  ------------------------------------------------------------
yberc0001_40750  ------------------------------------------------------------
yruck0001_35050  ------------------------------------------------------------
yruck0001_13030  ------------------------------------------------------------
yinte0001_17980  ------------------------------------------------------------
yaldo0001_6040   ------------------------------------------------------------
yfred0001_33270  ------------------------------------------------------------
yrohd0001_18870  ------------------------------------------------------------
yruck0001_4610   ------------------------------------------------------------
ypest0001X_2758  ------------------------------------------------------------
ypseu0001X_2843  ------------------------------------------------------------
ypseu0001X_2846  ------------------------------------------------------------
ypest0001X_2761  ------------------------------------------------------------
yrohd0001_32190  ------------------------------------------------------------
ymoll0001_21160  ------------------------------------------------------------
yrohd0001_32220  ------------------------------------------------------------
yfred0001_34080  ------------------------------------------------------------
yruck0001_4650   ------------------------------------------------------------
yaldo0001_38920  ------------------------------------------------------------
yberc0001_20820  ------------------------------------------------------------
yaldo0001_3710   ------------------------------------------------------------
ypest0001X_8090  ------------------------------------------------------------
yfred0001_34100  ------------------------------------------------------------
yruck0001_25350  ------------------------------------------------------------
yfred0001_38190  ------------------------------------------------------------
yfred0001_34090  ------------------------------------------------------------
yaldo0001_30990  ------------------------------------------------------------
yberc0001_36600  ------------------------------------------------------------
yrohd0001_40100  ------------------------------------------------------------
yrohd0001_40280  ------------------------------------------------------------
yruck0001_4660   ------------------------------------------------------------
ypseu0001X_3844  ------------------------------------------------------------
ykris0001_41250  ------------------------------------------------------------
ykris0001_21250  ------------------------------------------------------------
yruck0001_4630   ------------------------------------------------------------
yrohd0001_40320  ------------------------------------------------------------
yrohd0001_40080  ------------------------------------------------------------
yrohd0001_32210  ------------------------------------------------------------
yfred0001_45640  ------------------------------------------------------------
yfred0001_38220  ------------------------------------------------------------
yrohd0001_38410  ------------------------------------------------------------
yfred0001_33220  ------------------------------------------------------------
ymoll0001_36970  ------------------------------------------------------------
yrohd0001_39710  ------------------------------------------------------------
yrohd0001_18880  ------------------------------------------------------------
yinte0001_5480   ------------------------------------------------------------
yfred0001_33200  ------------------------------------------------------------
yrohd0001_18890  ------------------------------------------------------------
yfred0001_32960  ------------------------------------------------------------
yberc0001_40160  ------------------------------------------------------------
ypseu0001X_3843  ------------------------------------------------------------
ypseu0001X_3837  ------------------------------------------------------------
ypest0001X_8140  ------------------------------------------------------------
ypseu0001X_3848  ADQINLTADSLSNVEGRVVQKGSGDFRLDLPGYLDNRGGVLLTKGNLALQAERLTSNSQS
ypest0001X_8130  ------------------------------------------------------------
ypest0001X_8080  ADQINLTAYSLSNVEGRVVQKGSGDFRLDLPGYLDNRGGVLLTKGNLALQAERLTSNSQS
yinte0001_41760  ------------------------------------------------------------
ykris0001_32060  ------------------------------------------------------------
ypseu0001X_3841  ------------------------------------------------------------
yrohd0001_38400  ------------------------------------------------------------
yfred0001_40710  ------------------------------------------------------------
yente0001X_8000  ------------------------------------------------------------
yruck0001_35040  ------------------------------------------------------------
                                                                             


                        790       800       810       820       830       840
                 =========+=========+=========+=========+=========+=========+
yberc0001_8630   ----------------------------QGQNVAAGAMALTGSRVDLTGSQTQASNITIT
ykris0001_26670  ------------------------------------------------------------
ykris0001_41280  ------------------------------------------------------------
yruck0001_910    ------------------------------------------------------------
yaldo0001_37900  ------------------------------------------------------------
yberc0001_34750  ------------------------------------------------------------
yaldo0001_38900  ------------------------------------------------------------
yaldo0001_41000  ------------------------------------------------------------
ypseu0001X_3846  ------------------------------------------------------------
ymoll0001_35980  ------------------------------------------------------------
ypest0001X_8100  ------------------------------------------------------------
yfred0001_38200  ------------------------------------------------------------
ypest0001X_2754  ------------------------------------------------------------
yfred0001_34120  ------------------------------------------------------------
ypseu0001X_2842  ------------------------------------------------------------
ykris0001_7890   ------------------------------------------------------------
yruck0001_4640   ------------------------------------------------------------
ymoll0001_2720   ------------------------------------------------------------
yrohd0001_40310  ------------------------------------------------------------
yruck0001_34980  ------------------------------------------------------------
ypest0001X_2756  ------------------------------------------------------------
yfred0001_45620  ------------------------------------------------------------
yrohd0001_18860  ------------------------------------------------------------
yfred0001_33010  ------------------------------------------------------------
yfred0001_33210  ------------------------------------------------------------
yrohd0001_40300  ------------------------------------------------------------
yruck0001_4620   ------------------------------------------------------------
ypest0001X_8110  ------------------------------------------------------------
yberc0001_40130  ------------------------------------------------------------
yberc0001_40750  ------------------------------------------------------------
yruck0001_35050  ------------------------------------------------------------
yruck0001_13030  ------------------------------------------------------------
yinte0001_17980  ------------------------------------------------------------
yaldo0001_6040   ------------------------------------------------------------
yfred0001_33270  ------------------------------------------------------------
yrohd0001_18870  ------------------------------------------------------------
yruck0001_4610   ------------------------------------------------------------
ypest0001X_2758  ------------------------------------------------------------
ypseu0001X_2843  ------------------------------------------------------------
ypseu0001X_2846  ------------------------------------------------------------
ypest0001X_2761  ------------------------------------------------------------
yrohd0001_32190  ------------------------------------------------------------
ymoll0001_21160  ------------------------------------------------------------
yrohd0001_32220  ------------------------------------------------------------
yfred0001_34080  ------------------------------------------------------------
yruck0001_4650   ------------------------------------------------------------
yaldo0001_38920  ------------------------------------------------------------
yberc0001_20820  ------------------------------------------------------------
yaldo0001_3710   ----------------------------QGQNVAVGAMTLTGSRVDLTGSQTRASTITLT
ypest0001X_8090  ------------------------------------------------------------
yfred0001_34100  ------------------------------------------------------------
yruck0001_25350  ------------------------------------------------------------
yfred0001_38190  ------------------------------------------------------------
yfred0001_34090  ------------------------------------------------------------
yaldo0001_30990  ------------------------------------------------------------
yberc0001_36600  ------------------------------------------------------------
yrohd0001_40100  ------------------------------------------------------------
yrohd0001_40280  ------------------------------------------------------------
yruck0001_4660   ------------------------------------------------------------
ypseu0001X_3844  ------------------------------------------------------------
ykris0001_41250  ------------------------------------------------------------
ykris0001_21250  ------------------------------------------------------------
yruck0001_4630   ------------------------------------------------------------
yrohd0001_40320  ------------------------------------------------------------
yrohd0001_40080  ------------------------------------------------------------
yrohd0001_32210  ------------------------------------------------------------
yfred0001_45640  ------------------------------------------------------------
yfred0001_38220  ------------------------------------------------------------
yrohd0001_38410  ----------------------------QGQNVAAGAMTLSGSRVDLTGSQTQAGNITVT
yfred0001_33220  ----------------------------QGQNVAAGAMTLSGSRVDLTGSQTQAGNITIT
ymoll0001_36970  ------------------------------------------------------------
yrohd0001_39710  ------------------------------------------------------------
yrohd0001_18880  ------------------------------------------------------------
yinte0001_5480   ----------------------------QGQNVAAQDMTLTGSRVDLTGSQTQAGNITIT
yfred0001_33200  ------------------------------------------------------------
yrohd0001_18890  ------------------------------------------------------------
yfred0001_32960  ------------------------------------------------------------
yberc0001_40160  ------------------------------------------------------------
ypseu0001X_3843  ------------------------------------------------------------
ypseu0001X_3837  ------------------------------------------------------------
ypest0001X_8140  ------------------------------------------------------------
ypseu0001X_3848  LLGAGIQADGSKASKGDLQVNTTQALIAQGQNVAAGAMTLSGSRIDLTGSQTHASNITIT
ypest0001X_8130  ------------------------------------------------------------
ypest0001X_8080  LLGAGIQADGSKASKGDLQANTTQALIAQGQNVAAGTMTLSGSRVDLTGSQTHASNITIT
yinte0001_41760  ----------------------------DGSLYAEGPLNVRAGEFLLTGRATGKQGIAIN
ykris0001_32060  ----------------------------DGALYAEGPLNVRAGEFLLAGRATGKQGLAIN
ypseu0001X_3841  ------------------------------------------------------------
yrohd0001_38400  ------------------------------------------------------------
yfred0001_40710  ------------------------------------------------------------
yente0001X_8000  ------------------------------------------------------------
yruck0001_35040  ------------------------------------------------------------
                                                                             


                        850       860       870       880       890       900
                 =========+=========+=========+=========+=========+=========+
yberc0001_8630   ARD---------------------------------------------------------
ykris0001_26670  ------------------------------------------------------------
ykris0001_41280  ------------------------------------------------------------
yruck0001_910    ------------------------------------------------------------
yaldo0001_37900  ------------------------------------------------------------
yberc0001_34750  ------------------------------------------------------------
yaldo0001_38900  ------------------------------------------------------------
yaldo0001_41000  ------------------------------------------------------------
ypseu0001X_3846  ------------------------------------------------------------
ymoll0001_35980  ------------------------------------------------------------
ypest0001X_8100  ------------------------------------------------------------
yfred0001_38200  ------------------------------------------------------------
ypest0001X_2754  ------------------------------------------------------------
yfred0001_34120  ------------------------------------------------------------
ypseu0001X_2842  ------------------------------------------------------------
ykris0001_7890   ------------------------------------------------------------
yruck0001_4640   ------------------------------------------------------------
ymoll0001_2720   ------------------------------------------------------------
yrohd0001_40310  ------------------------------------------------------------
yruck0001_34980  ------------------------------------------------------------
ypest0001X_2756  ------------------------------------------------------------
yfred0001_45620  ------------------------------------------------------------
yrohd0001_18860  ------------------------------------------------------------
yfred0001_33010  ------------------------------------------------------------
yfred0001_33210  ------------------------------------------------------------
yrohd0001_40300  ------------------------------------------------------------
yruck0001_4620   ------------------------------------------------------------
ypest0001X_8110  ------------------------------------------------------------
yberc0001_40130  ------------------------------------------------------------
yberc0001_40750  ------------------------------------------------------------
yruck0001_35050  ------------------------------------------------------------
yruck0001_13030  ------------------------------------------------------------
yinte0001_17980  ------------------------------------------------------------
yaldo0001_6040   ------------------------------------------------------------
yfred0001_33270  ------------------------------------------------------------
yrohd0001_18870  ------------------------------------------------------------
yruck0001_4610   ------------------------------------------------------------
ypest0001X_2758  ------------------------------------------------------------
ypseu0001X_2843  ------------------------------------------------------------
ypseu0001X_2846  ------------------------------------------------------------
ypest0001X_2761  ------------------------------------------------------------
yrohd0001_32190  ------------------------------------------------------------
ymoll0001_21160  ------------------------------------------------------------
yrohd0001_32220  ------------------------------------------------------------
yfred0001_34080  ------------------------------------------------------------
yruck0001_4650   ------------------------------------------------------------
yaldo0001_38920  ------------------------------------------------------------
yberc0001_20820  ------------------------------------------------------------
yaldo0001_3710   ARD---------------------------------------------------------
ypest0001X_8090  ------------------------------------------------------------
yfred0001_34100  ------------------------------------------------------------
yruck0001_25350  ------------------------------------------------------------
yfred0001_38190  ------------------------------------------------------------
yfred0001_34090  ------------------------------------------------------------
yaldo0001_30990  ------------------------------------------------------------
yberc0001_36600  ------------------------------------------------------------
yrohd0001_40100  ------------------------------------------------------------
yrohd0001_40280  ------------------------------------------------------------
yruck0001_4660   ------------------------------------------------------------
ypseu0001X_3844  ------------------------------------------------------------
ykris0001_41250  ------------------------------------------------------------
ykris0001_21250  ------------------------------------------------------------
yruck0001_4630   ------------------------------------------------------------
yrohd0001_40320  ------------------------------------------------------------
yrohd0001_40080  ------------------------------------------------------------
yrohd0001_32210  ------------------------------------------------------------
yfred0001_45640  ------------------------------------------------------------
yfred0001_38220  ------------------------------------------------------------
yrohd0001_38410  AHD---------------------------------------------------------
yfred0001_33220  ARD---------------------------------------------------------
ymoll0001_36970  ------------------------------------------------------------
yrohd0001_39710  ------------------------------------------------------------
yrohd0001_18880  ------------------------------------------------------------
yinte0001_5480   ARD---------------------------------------------------------
yfred0001_33200  ------------------------------------------------------------
yrohd0001_18890  ------------------------------------------------------------
yfred0001_32960  ------------------------------------------------------------
yberc0001_40160  ------------------------------------------------------------
ypseu0001X_3843  ------------------------------------------------------------
ypseu0001X_3837  ------------------------------------------------------------
ypest0001X_8140  ------------------------------------------------------------
ypseu0001X_3848  ARD---------------------------------------------------------
ypest0001X_8130  ------------------------------------------------------------
ypest0001X_8080  ARDGDVTTREATLITPGTLSMTAVANPEQTLNNRGGKLHADNIQLNLAKLENSNGEIAAA
yinte0001_41760  SNL---------------------------------------------------------
ykris0001_32060  SNL---------------------------------------------------------
ypseu0001X_3841  ------------------------------------------------------------
yrohd0001_38400  ------------------------------------------------------------
yfred0001_40710  ------------------------------------------------------------
yente0001X_8000  ------------------------------------------------------------
yruck0001_35040  ------------------------------------------------------------
                                                                             


                        910       920       930       940       950       960
                 =========+=========+=========+=========+=========+=========+
yberc0001_8630   ------------------------------------------------------------
ykris0001_26670  ------------------------------------------------------------
ykris0001_41280  ------------------------------------------------------------
yruck0001_910    ------------------------------------------------------------
yaldo0001_37900  ------------------------------------------------------------
yberc0001_34750  ------------------------------------------------------------
yaldo0001_38900  ------------------------------------------------------------
yaldo0001_41000  ------------------------------------------------------------
ypseu0001X_3846  ------------------------------------------------------------
ymoll0001_35980  ------------------------------------------------------------
ypest0001X_8100  ------------------------------------------------------------
yfred0001_38200  ------------------------------------------------------------
ypest0001X_2754  ------------------------------------------------------------
yfred0001_34120  ------------------------------------------------------------
ypseu0001X_2842  ------------------------------------------------------------
ykris0001_7890   ------------------------------------------------------------
yruck0001_4640   ------------------------------------------------------------
ymoll0001_2720   ------------------------------------------------------------
yrohd0001_40310  ------------------------------------------------------------
yruck0001_34980  ------------------------------------------------------------
ypest0001X_2756  ------------------------------------------------------------
yfred0001_45620  ------------------------------------------------------------
yrohd0001_18860  ------------------------------------------------------------
yfred0001_33010  ------------------------------------------------------------
yfred0001_33210  ------------------------------------------------------------
yrohd0001_40300  ------------------------------------------------------------
yruck0001_4620   ------------------------------------------------------------
ypest0001X_8110  ------------------------------------------------------------
yberc0001_40130  ------------------------------------------------------------
yberc0001_40750  ------------------------------------------------------------
yruck0001_35050  ------------------------------------------------------------
yruck0001_13030  ------------------------------------------------------------
yinte0001_17980  ------------------------------------------------------------
yaldo0001_6040   ------------------------------------------------------------
yfred0001_33270  ------------------------------------------------------------
yrohd0001_18870  ------------------------------------------------------------
yruck0001_4610   ------------------------------------------------------------
ypest0001X_2758  ------------------------------------------------------------
ypseu0001X_2843  ------------------------------------------------------------
ypseu0001X_2846  ------------------------------------------------------------
ypest0001X_2761  ------------------------------------------------------------
yrohd0001_32190  ------------------------------------------------------------
ymoll0001_21160  ------------------------------------------------------------
yrohd0001_32220  ------------------------------------------------------------
yfred0001_34080  ------------------------------------------------------------
yruck0001_4650   ------------------------------------------------------------
yaldo0001_38920  ------------------------------------------------------------
yberc0001_20820  ------------------------------------------------------------
yaldo0001_3710   ------------------------------------------------------------
ypest0001X_8090  ------------------------------------------------------------
yfred0001_34100  ------------------------------------------------------------
yruck0001_25350  ------------------------------------------------------------
yfred0001_38190  ------------------------------------------------------------
yfred0001_34090  ------------------------------------------------------------
yaldo0001_30990  ------------------------------------------------------------
yberc0001_36600  ------------------------------------------------------------
yrohd0001_40100  ------------------------------------------------------------
yrohd0001_40280  ------------------------------------------------------------
yruck0001_4660   ------------------------------------------------------------
ypseu0001X_3844  ------------------------------------------------------------
ykris0001_41250  ------------------------------------------------------------
ykris0001_21250  ------------------------------------------------------------
yruck0001_4630   ------------------------------------------------------------
yrohd0001_40320  ------------------------------------------------------------
yrohd0001_40080  ------------------------------------------------------------
yrohd0001_32210  ------------------------------------------------------------
yfred0001_45640  ------------------------------------------------------------
yfred0001_38220  ------------------------------------------------------------
yrohd0001_38410  ------------------------------------------------------------
yfred0001_33220  ------------------------------------------------------------
ymoll0001_36970  ------------------------------------------------------------
yrohd0001_39710  ------------------------------------------------------------
yrohd0001_18880  ------------------------------------------------------------
yinte0001_5480   ------------------------------------------------------------
yfred0001_33200  ------------------------------------------------------------
yrohd0001_18890  ------------------------------------------------------------
yfred0001_32960  ------------------------------------------------------------
yberc0001_40160  ------------------------------------------------------------
ypseu0001X_3843  ------------------------------------------------------------
ypseu0001X_3837  ------------------------------------------------------------
ypest0001X_8140  ------------------------------------------------------------
ypseu0001X_3848  ------------------------------------------------------------
ypest0001X_8130  ------------------------------------------------------------
ypest0001X_8080  TDLWLRLQSDFIHQAGARLTAGRDLLFNSRGALINQYKLEAGRDMQLTALSIRNTSADRN
yinte0001_41760  ------------------------------------------------------------
ykris0001_32060  ------------------------------------------------------------
ypseu0001X_3841  ------------------------------------------------------------
yrohd0001_38400  ------------------------------------------------------------
yfred0001_40710  ------------------------------------------------------------
yente0001X_8000  ------------------------------------------------------------
yruck0001_35040  ------------------------------------------------------------
                                                                             


                        970       980       990      1000      1010      1020
                 =========+=========+=========+=========+=========+=========+
yberc0001_8630   -------------------------------------------GDVSTQDATVMTPGTLA
ykris0001_26670  ------------------------------------------------------------
ykris0001_41280  ------------------------------------------------------------
yruck0001_910    ------------------------------------------------------------
yaldo0001_37900  ------------------------------------------------------------
yberc0001_34750  ------------------------------------------------------------
yaldo0001_38900  ------------------------------------------------------------
yaldo0001_41000  ------------------------------------------------------------
ypseu0001X_3846  ------------------------------------------------------------
ymoll0001_35980  ------------------------------------------------------------
ypest0001X_8100  ------------------------------------------------------------
yfred0001_38200  ------------------------------------------------------------
ypest0001X_2754  ------------------------------------------------------------
yfred0001_34120  ------------------------------------------------------------
ypseu0001X_2842  ------------------------------------------------------------
ykris0001_7890   ------------------------------------------------------------
yruck0001_4640   ------------------------------------------------------------
ymoll0001_2720   ------------------------------------------------------------
yrohd0001_40310  ------------------------------------------------------------
yruck0001_34980  ------------------------------------------------------------
ypest0001X_2756  ------------------------------------------------------------
yfred0001_45620  ------------------------------------------------------------
yrohd0001_18860  ------------------------------------------------------------
yfred0001_33010  ------------------------------------------------------------
yfred0001_33210  ------------------------------------------------------------
yrohd0001_40300  ------------------------------------------------------------
yruck0001_4620   ------------------------------------------------------------
ypest0001X_8110  ------------------------------------------------------------
yberc0001_40130  ------------------------------------------------------------
yberc0001_40750  ------------------------------------------------------------
yruck0001_35050  ------------------------------------------------------------
yruck0001_13030  ------------------------------------------------------------
yinte0001_17980  ------------------------------------------------------------
yaldo0001_6040   ------------------------------------------------------------
yfred0001_33270  ------------------------------------------------------------
yrohd0001_18870  ------------------------------------------------------------
yruck0001_4610   ------------------------------------------------------------
ypest0001X_2758  ------------------------------------------------------------
ypseu0001X_2843  ------------------------------------------------------------
ypseu0001X_2846  ------------------------------------------------------------
ypest0001X_2761  ------------------------------------------------------------
yrohd0001_32190  ------------------------------------------------------------
ymoll0001_21160  ------------------------------------------------------------
yrohd0001_32220  ------------------------------------------------------------
yfred0001_34080  ------------------------------------------------------------
yruck0001_4650   ------------------------------------------------------------
yaldo0001_38920  ------------------------------------------------------------
yberc0001_20820  ------------------------------------------------------------
yaldo0001_3710   -------------------------------------------GDVSTRGATVLTPGKLS
ypest0001X_8090  ------------------------------------------------------------
yfred0001_34100  ------------------------------------------------------------
yruck0001_25350  ------------------------------------------------------------
yfred0001_38190  ------------------------------------------------------------
yfred0001_34090  ------------------------------------------------------------
yaldo0001_30990  ------------------------------------------------------------
yberc0001_36600  ------------------------------------------------------------
yrohd0001_40100  ------------------------------------------------------------
yrohd0001_40280  ------------------------------------------------------------
yruck0001_4660   ------------------------------------------------------------
ypseu0001X_3844  ------------------------------------------------------------
ykris0001_41250  ------------------------------------------------------------
ykris0001_21250  ------------------------------------------------------------
yruck0001_4630   ------------------------------------------------------------
yrohd0001_40320  ------------------------------------------------------------
yrohd0001_40080  ------------------------------------------------------------
yrohd0001_32210  ------------------------------------------------------------
yfred0001_45640  ------------------------------------------------------------
yfred0001_38220  ------------------------------------------------------------
yrohd0001_38410  -------------------------------------------GDISTQDATVLTPGILA
yfred0001_33220  -------------------------------------------GDISTQDATVLTPGTLA
ymoll0001_36970  ------------------------------------------------------------
yrohd0001_39710  ------------------------------------------------------------
yrohd0001_18880  ------------------------------------------------------------
yinte0001_5480   -------------------------------------------GDVSTQDATVLTPGTLE
yfred0001_33200  ------------------------------------------------------------
yrohd0001_18890  ------------------------------------------------------------
yfred0001_32960  ------------------------------------------------------------
yberc0001_40160  ------------------------------------------------------------
ypseu0001X_3843  ------------------------------------------------------------
ypseu0001X_3837  ------------------------------------------------------------
ypest0001X_8140  ------------------------------------------------------------
ypseu0001X_3848  -------------------------------------------GDVTTREATLITPGTLS
ypest0001X_8130  ------------------------------------------------------------
ypest0001X_8080  TNADNSSLLAGRGLSLSTDSLFNRGAIYTTGVGQFTVNGNTENIGEIYTEQQLTFTATGN
yinte0001_41760  ------------------------------------------------------------
ykris0001_32060  ------------------------------------------------------------
ypseu0001X_3841  ------------------------------------------------------------
yrohd0001_38400  ------------------------------------------------------------
yfred0001_40710  ------------------------------------------------------------
yente0001X_8000  ------------------------------------------------------------
yruck0001_35040  ------------------------------------------------------------
                                                                             


                       1030      1040      1050      1060      1070      1080
                 =========+=========+=========+=========+=========+=========+
yberc0001_8630   ITAAADPKQTLNNSGGKLHADNIQLNLARLDSSKGEIAAATDMWIRLQSDFTHQAGARLT
ykris0001_26670  ------------------------------------------------------------
ykris0001_41280  ------------------------------------------------------------
yruck0001_910    ------------------------------------------------------------
yaldo0001_37900  ------------------------------------------------------------
yberc0001_34750  ------------------------------------------------------------
yaldo0001_38900  ------------------------------------------------------------
yaldo0001_41000  ------------------------------------------------------------
ypseu0001X_3846  ------------------------------------------------------------
ymoll0001_35980  ------------------------------------------------------------
ypest0001X_8100  ------------------------------------------------------------
yfred0001_38200  ------------------------------------------------------------
ypest0001X_2754  ------------------------------------------------------------
yfred0001_34120  ------------------------------------------------------------
ypseu0001X_2842  ------------------------------------------------------------
ykris0001_7890   ------------------------------------------------------------
yruck0001_4640   ------------------------------------------------------------
ymoll0001_2720   ------------------------------------------------------------
yrohd0001_40310  ------------------------------------------------------------
yruck0001_34980  ------------------------------------------------------------
ypest0001X_2756  ------------------------------------------------------------
yfred0001_45620  ------------------------------------------------------------
yrohd0001_18860  ------------------------------------------------------------
yfred0001_33010  ------------------------------------------------------------
yfred0001_33210  ------------------------------------------------------------
yrohd0001_40300  ------------------------------------------------------------
yruck0001_4620   ------------------------------------------------------------
ypest0001X_8110  ------------------------------------------------------------
yberc0001_40130  ------------------------------------------------------------
yberc0001_40750  ------------------------------------------------------------
yruck0001_35050  ------------------------------------------------------------
yruck0001_13030  ------------------------------------------------------------
yinte0001_17980  ------------------------------------------------------------
yaldo0001_6040   ------------------------------------------------------------
yfred0001_33270  ------------------------------------------------------------
yrohd0001_18870  ------------------------------------------------------------
yruck0001_4610   ------------------------------------------------------------
ypest0001X_2758  ------------------------------------------------------------
ypseu0001X_2843  ------------------------------------------------------------
ypseu0001X_2846  ------------------------------------------------------------
ypest0001X_2761  ------------------------------------------------------------
yrohd0001_32190  ------------------------------------------------------------
ymoll0001_21160  ------------------------------------------------------------
yrohd0001_32220  ------------------------------------------------------------
yfred0001_34080  ------------------------------------------------------------
yruck0001_4650   ------------------------------------------------------------
yaldo0001_38920  ------------------------------------------------------------
yberc0001_20820  ------------------------------------------------------------
yaldo0001_3710   ITAAANPEQTLNNRGGKLHADNIQLNLAKLDNSQGEIAAATDLRIRLQSDVTHQAGARLT
ypest0001X_8090  ------------------------------------------------------------
yfred0001_34100  ------------------------------------------------------------
yruck0001_25350  ------------------------------------------------------------
yfred0001_38190  ------------------------------------------------------------
yfred0001_34090  ------------------------------------------------------------
yaldo0001_30990  ------------------------------------------------------------
yberc0001_36600  ------------------------------------------------------------
yrohd0001_40100  ------------------------------------------------------------
yrohd0001_40280  ------------------------------------------------------------
yruck0001_4660   ------------------------------------------------------------
ypseu0001X_3844  ------------------------------------------------------------
ykris0001_41250  ------------------------------------------------------------
ykris0001_21250  ------------------------------------------------------------
yruck0001_4630   ------------------------------------------------------------
yrohd0001_40320  ------------------------------------------------------------
yrohd0001_40080  ------------------------------------------------------------
yrohd0001_32210  ------------------------------------------------------------
yfred0001_45640  ------------------------------------------------------------
yfred0001_38220  ------------------------------------------------------------
yrohd0001_38410  ITAAANSKQTLNNRGGKLHADNIQLNLAKLDSSKGEIAAATDMWIRLQSDFTHQAGARLT
yfred0001_33220  ITAAANTKQTLNNSGGKLHADNIQLNLAKLDSSKGEIAAATDMWVRLQSDFTHQAGARLT
ymoll0001_36970  ------------------------------------------------------------
yrohd0001_39710  ------------------------------------------------------------
yrohd0001_18880  ------------------------------------------------------------
yinte0001_5480   ITAAANPKQTLNNSGGKLHADNIQLNLAKLDNSKGEIAAATDLWIRLQSDFTHQAGARLT
yfred0001_33200  ------------------------------------------------------------
yrohd0001_18890  ------------------------------------------------------------
yfred0001_32960  ------------------------------------------------------------
yberc0001_40160  ------------------------------------------------------------
ypseu0001X_3843  ------------------------------------------------------------
ypseu0001X_3837  ------------------------------------------------------------
ypest0001X_8140  ------------------------------------------------------------
ypseu0001X_3848  MTAVANPEQTLNNRGGKLHADNIQLNLAKLENSNGEIAAATDLWLRLQSDFIHQAGARLT
ypest0001X_8130  ------------------------------------------------------------
ypest0001X_8080  LANRGVMQTRGEMQLSAQGDVNNSGMLYSAGDQMRLSIAGNLTNEGKLHVANGEMRLLTE
yinte0001_41760  ---TTAEGSALLSTGDIHLQGGALLLSGLLSGDHALTVAGEQFTTGSSAQIQAKDSITLN
ykris0001_32060  ---TATEGSALLSSGDIHLQGETLLLSGLLSGDRALTVAGEQFTTGNRAQIQAKDSITLN
ypseu0001X_3841  ------------------------------------------------------------
yrohd0001_38400  ------------------------------------------------------------
yfred0001_40710  ------------------------------------------------------------
yente0001X_8000  ------------------------------------------------------------
yruck0001_35040  ------------------------------------------------------------
                                                                             


                       1090      1100      1110      1120      1130      1140
                 =========+=========+=========+=========+=========+=========+
yberc0001_8630   AGRDLHFNTSGALINQYKLEAGRDMQLTALSISNSNAD-----NSSALLAGRDLSLNTDS
ykris0001_26670  ------------------------------------------------------------
ykris0001_41280  ------------------------------------------------------------
yruck0001_910    ------------------------------------------------------------
yaldo0001_37900  ------------------------------------------------------------
yberc0001_34750  ------------------------------------------------------------
yaldo0001_38900  ------------------------------------------------------------
yaldo0001_41000  ------------------------------------------------------------
ypseu0001X_3846  ------------------------------------------------------------
ymoll0001_35980  ------------------------------------------------------------
ypest0001X_8100  ------------------------------------------------------------
yfred0001_38200  ------------------------------------------------------------
ypest0001X_2754  ------------------------------------------------------------
yfred0001_34120  ------------------------------------------------------------
ypseu0001X_2842  ------------------------------------------------------------
ykris0001_7890   ------------------------------------------------------------
yruck0001_4640   ------------------------------------------------------------
ymoll0001_2720   ------------------------------------------------------------
yrohd0001_40310  ------------------------------------------------------------
yruck0001_34980  ------------------------------------------------------------
ypest0001X_2756  ------------------------------------------------------------
yfred0001_45620  ------------------------------------------------------------
yrohd0001_18860  ------------------------------------------------------------
yfred0001_33010  ------------------------------------------------------------
yfred0001_33210  ------------------------------------------------------------
yrohd0001_40300  ------------------------------------------------------------
yruck0001_4620   ------------------------------------------------------------
ypest0001X_8110  ------------------------------------------------------------
yberc0001_40130  ------------------------------------------------------------
yberc0001_40750  ------------------------------------------------------------
yruck0001_35050  ------------------------------------------------------------
yruck0001_13030  ------------------------------------------------------------
yinte0001_17980  ------------------------------------------------------------
yaldo0001_6040   ------------------------------------------------------------
yfred0001_33270  ------------------------------------------------------------
yrohd0001_18870  ------------------------------------------------------------
yruck0001_4610   ------------------------------------------------------------
ypest0001X_2758  ------------------------------------------------------------
ypseu0001X_2843  ------------------------------------------------------------
ypseu0001X_2846  ------------------------------------------------------------
ypest0001X_2761  ------------------------------------------------------------
yrohd0001_32190  ------------------------------------------------------------
ymoll0001_21160  ------------------------------------------------------------
yrohd0001_32220  ------------------------------------------------------------
yfred0001_34080  ------------------------------------------------------------
yruck0001_4650   ------------------------------------------------------------
yaldo0001_38920  ------------------------------------------------------------
yberc0001_20820  ------------------------------------------------------------
yaldo0001_3710   AGRDLVFNSTGALINQYKLEAGRDMQLTALSISNSG-------SSSALLAGRDVSLSTDN
ypest0001X_8090  ------------------------------------------------------------
yfred0001_34100  ------------------------------------------------------------
yruck0001_25350  ------------------------------------------------------------
yfred0001_38190  ------------------------------------------------------------
yfred0001_34090  ------------------------------------------------------------
yaldo0001_30990  ------------------------------------------------------------
yberc0001_36600  ------------------------------------------------------------
yrohd0001_40100  ------------------------------------------------------------
yrohd0001_40280  ------------------------------------------------------------
yruck0001_4660   ------------------------------------------------------------
ypseu0001X_3844  ------------------------------------------------------------
ykris0001_41250  ------------------------------------------------------------
ykris0001_21250  ------------------------------------------------------------
yruck0001_4630   ------------------------------------------------------------
yrohd0001_40320  ------------------------------------------------------------
yrohd0001_40080  ------------------------------------------------------------
yrohd0001_32210  ------------------------------------------------------------
yfred0001_45640  ------------------------------------------------------------
yfred0001_38220  ------------------------------------------------------------
yrohd0001_38410  AGSDLAFTTSGTLTNQYKLEAGRDMQLTALSISNTNAD-----NNSALLAGRDLSLNTDS
yfred0001_33220  AGRDLAFATSGALTNQYKLEAGRDMQLTALSISNANTD-----NSSALLAGRDLSLNTDS
ymoll0001_36970  ------------------------------------------------------------
yrohd0001_39710  ------------------------------------------------------------
yrohd0001_18880  ------------------------------------------------------------
yinte0001_5480   AGRDLVFSSTGSLINQYKLEAGRDMQLTALSISNTNAD-----DSSALLAGRDMALSTDS
yfred0001_33200  ------------------------------------------------------------
yrohd0001_18890  ------------------------------------------------------------
yfred0001_32960  ------------------------------------------------------------
yberc0001_40160  ------------------------------------------------------------
ypseu0001X_3843  ------------------------------------------------------------
ypseu0001X_3837  ------------------------------------------------------------
ypest0001X_8140  ------------------------------------------------------------
ypseu0001X_3848  AGRDLLFNSRGALINQYKLEAGRNMQLTALSIRNTSADRNTNADNSSLLAGRGLSLSTDS
ypest0001X_8130  ------------------------------------------------------------
ypest0001X_8080  GNLDNRGSLYGAGNSDITTQGNAVNTGSVYTQGALQWLTKGSVRNSASIAALGDLQLRAN
yinte0001_41760  AEKNAQLAGIFTTFGDLNVVSGATENRADIVARNIGWHSDSLTQQGRMQADHDLTLTVNQ
ykris0001_32060  TEKNAQLAGIFTTLGDLNLVSGTTENRADIVARNIDWRTDSLIQQGRMQADHDLMLTVNQ
ypseu0001X_3841  ------------------------------------------------------------
yrohd0001_38400  ------------------------------------------------------------
yfred0001_40710  ------------------------------------------------------------
yente0001X_8000  ------------------------------------------------------------
yruck0001_35040  ------------------------------------------------------------
                                                                             


                       1150      1160      1170      1180      1190      1200
                 =========+=========+=========+=========+=========+=========+
yberc0001_8630   LFNSGTLYATGVGQFTVNGNAENLGEIYTEQQLTLVSAGNLDNRGVVQTRGNMQLSTQGY
ykris0001_26670  ------------------------------------------------------------
ykris0001_41280  ------------------------------------------------------------
yruck0001_910    ------------------------------------------------------------
yaldo0001_37900  ------------------------------------------------------------
yberc0001_34750  ------------------------------------------------------------
yaldo0001_38900  ------------------------------------------------------------
yaldo0001_41000  ------------------------------------------------------------
ypseu0001X_3846  ------------------------------------------------------------
ymoll0001_35980  ------------------------------------------------------------
ypest0001X_8100  ------------------------------------------------------------
yfred0001_38200  ------------------------------------------------------------
ypest0001X_2754  ------------------------------------------------------------
yfred0001_34120  ------------------------------------------------------------
ypseu0001X_2842  ------------------------------------------------------------
ykris0001_7890   ------------------------------------------------------------
yruck0001_4640   ------------------------------------------------------------
ymoll0001_2720   ------------------------------------------------------------
yrohd0001_40310  ------------------------------------------------------------
yruck0001_34980  ------------------------------------------------------------
ypest0001X_2756  ------------------------------------------------------------
yfred0001_45620  ------------------------------------------------------------
yrohd0001_18860  ------------------------------------------------------------
yfred0001_33010  ------------------------------------------------------------
yfred0001_33210  ------------------------------------------------------------
yrohd0001_40300  ------------------------------------------------------------
yruck0001_4620   ------------------------------------------------------------
ypest0001X_8110  ------------------------------------------------------------
yberc0001_40130  ------------------------------------------------------------
yberc0001_40750  ------------------------------------------------------------
yruck0001_35050  ------------------------------------------------------------
yruck0001_13030  ------------------------------------------------------------
yinte0001_17980  ------------------------------------------------------------
yaldo0001_6040   ------------------------------------------------------------
yfred0001_33270  ------------------------------------------------------------
yrohd0001_18870  ------------------------------------------------------------
yruck0001_4610   ------------------------------------------------------------
ypest0001X_2758  ------------------------------------------------------------
ypseu0001X_2843  ------------------------------------------------------------
ypseu0001X_2846  ------------------------------------------------------------
ypest0001X_2761  ------------------------------------------------------------
yrohd0001_32190  ------------------------------------------------------------
ymoll0001_21160  ------------------------------------------------------------
yrohd0001_32220  ------------------------------------------------------------
yfred0001_34080  ------------------------------------------------------------
yruck0001_4650   ------------------------------------------------------------
yaldo0001_38920  ------------------------------------------------------------
yberc0001_20820  ------------------------------------------------------------
yaldo0001_3710   LFNSGTLYATGTGQFTVNGNAENHGEIYTEQQLTFTTTGNLANHGVMQTRGDMLLSTQGE
ypest0001X_8090  ------------------------------------------------------------
yfred0001_34100  ------------------------------------------------------------
yruck0001_25350  ------------------------------------------------------------
yfred0001_38190  ------------------------------------------------------------
yfred0001_34090  ------------------------------------------------------------
yaldo0001_30990  ------------------------------------------------------------
yberc0001_36600  ------------------------------------------------------------
yrohd0001_40100  ------------------------------------------------------------
yrohd0001_40280  ------------------------------------------------------------
yruck0001_4660   ------------------------------------------------------------
ypseu0001X_3844  ------------------------------------------------------------
ykris0001_41250  ------------------------------------------------------------
ykris0001_21250  ------------------------------------------------------------
yruck0001_4630   ------------------------------------------------------------
yrohd0001_40320  ------------------------------------------------------------
yrohd0001_40080  ------------------------------------------------------------
yrohd0001_32210  ------------------------------------------------------------
yfred0001_45640  ------------------------------------------------------------
yfred0001_38220  ------------------------------------------------------------
yrohd0001_38410  LFNSGTLYAAGVGQFTINGNAENRGEIYTEQQLTLASAGNLANSGVIQTRGDMQLSTEGY
yfred0001_33220  LFNSGTLYAAGVGQFTINGNVENRGEIYTEQQLTLASAGNLVNSGVIQTRGDMQLSTAGY
ymoll0001_36970  ------------------------------------------------------------
yrohd0001_39710  ------------------------------------------------------------
yrohd0001_18880  ------------------------------------------------------------
yinte0001_5480   LFNRGAIYATGVGQFTVNGNVENRGEIYTEQQLTFTTTGHLANRGVMQTRGDMRLSTQGD
yfred0001_33200  ------------------------------------------------------------
yrohd0001_18890  ------------------------------------------------------------
yfred0001_32960  ------------------------------------------------------------
yberc0001_40160  ------------------------------------------------------------
ypseu0001X_3843  ------------------------------------------------------------
ypseu0001X_3837  ------------------------------------------------------------
ypest0001X_8140  ------------------------------------------------------------
ypseu0001X_3848  LFNRGAIYTTGVGQFTVNGNTENIGEIYTEQQLTFTATGNLANRGVMQTRGEMQLSSQGE
ypest0001X_8130  ------------------------------------------------------------
ypest0001X_8080  DLLSDNQSLMAAGLKADGSRSDSGNLAVSTEQALIAQGQNIAAGSLALAGSQIDLTGSQT
yinte0001_41760  LNQQGTLQAGQQLTLRGEQLVNSGLVSAPQLELAFTRSLDNSGSLIASQGLTLDVPSLSN
ykris0001_32060  LNQHGTLQAGQKLALRGEQLVNSGRIGAPQLELAFTRSLDNSGSLMASQGLTLEVPSLSN
ypseu0001X_3841  ------------------------------------------------------------
yrohd0001_38400  ------------------------------------------------------------
yfred0001_40710  ------------------------------------------------------------
yente0001X_8000  ------------------------------------------------------------
yruck0001_35040  ------------------------------------------------------------
                                                                             


                       1210      1220      1230      1240      1250      1260
                 =========+=========+=========+=========+=========+=========+
yberc0001_8630   LNNSGTLYGAGDHMGLSVTGNLTNKGSLYAAKGYLHLLTEGNLDNSGSLYGAG-------
ykris0001_26670  ------------------------------------------------------------
ykris0001_41280  ------------------------------------------------------------
yruck0001_910    ------------------------------------------------------------
yaldo0001_37900  ------------------------------------------------------------
yberc0001_34750  ------------------------------------------------------------
yaldo0001_38900  ------------------------------------------------------------
yaldo0001_41000  ------------------------------------------------------------
ypseu0001X_3846  ------------------------------------------------------------
ymoll0001_35980  ------------------------------------------------------------
ypest0001X_8100  ------------------------------------------------------------
yfred0001_38200  ------------------------------------------------------------
ypest0001X_2754  ------------------------------------------------------------
yfred0001_34120  ------------------------------------------------------------
ypseu0001X_2842  ------------------------------------------------------------
ykris0001_7890   ------------------------------------------------------------
yruck0001_4640   ------------------------------------------------------------
ymoll0001_2720   ------------------------------------------------------------
yrohd0001_40310  ------------------------------------------------------------
yruck0001_34980  ------------------------------------------------------------
ypest0001X_2756  ------------------------------------------------------------
yfred0001_45620  ------------------------------------------------------------
yrohd0001_18860  ------------------------------------------------------------
yfred0001_33010  ------------------------------------------------------------
yfred0001_33210  ------------------------------------------------------------
yrohd0001_40300  ------------------------------------------------------------
yruck0001_4620   ------------------------------------------------------------
ypest0001X_8110  ------------------------------------------------------------
yberc0001_40130  ------------------------------------------------------------
yberc0001_40750  ------------------------------------------------------------
yruck0001_35050  ------------------------------------------------------------
yruck0001_13030  ------------------------------------------------------------
yinte0001_17980  ------------------------------------------------------------
yaldo0001_6040   ------------------------------------------------------------
yfred0001_33270  ------------------------------------------------------------
yrohd0001_18870  ------------------------------------------------------------
yruck0001_4610   ------------------------------------------------------------
ypest0001X_2758  ------------------------------------------------------------
ypseu0001X_2843  ------------------------------------------------------------
ypseu0001X_2846  ------------------------------------------------------------
ypest0001X_2761  ------------------------------------------------------------
yrohd0001_32190  ------------------------------------------------------------
ymoll0001_21160  ------------------------------------------------------------
yrohd0001_32220  ------------------------------------------------------------
yfred0001_34080  ------------------------------------------------------------
yruck0001_4650   ------------------------------------------------------------
yaldo0001_38920  ------------------------------------------------------------
yberc0001_20820  ------------------------------------------------------------
yaldo0001_3710   LNNSGTLYAAGDQMQLSINGNLTNQGKLYAANGNVHLRAEGYLDNSGSLYSAG-------
ypest0001X_8090  ------------------------------------------------------------
yfred0001_34100  ------------------------------------------------------------
yruck0001_25350  ------------------------------------------------------------
yfred0001_38190  ------------------------------------------------------------
yfred0001_34090  ------------------------------------------------------------
yaldo0001_30990  ------------------------------------------------------------
yberc0001_36600  ------------------------------------------------------------
yrohd0001_40100  ------------------------------------------------------------
yrohd0001_40280  ------------------------------------------------------------
yruck0001_4660   ------------------------------------------------------------
ypseu0001X_3844  ------------------------------------------------------------
ykris0001_41250  ------------------------------------------------------------
ykris0001_21250  ------------------------------------------------------------
yruck0001_4630   ------------------------------------------------------------
yrohd0001_40320  ------------------------------------------------------------
yrohd0001_40080  ------------------------------------------------------------
yrohd0001_32210  ------------------------------------------------------------
yfred0001_45640  ------------------------------------------------------------
yfred0001_38220  ------------------------------------------------------------
yrohd0001_38410  LNNSGTLYSAGDQMALSVTGDLTNKGSLYAAKGNLHLLTKGNLDNSGSLFGAG-------
yfred0001_33220  LNNSGTLYSAGDQMALSTSGDLTNKGSLYAARGNLHLLTKANLDNSGSLYGTG-------
ymoll0001_36970  ------------------------------------------------------------
yrohd0001_39710  ------------------------------------------------------------
yrohd0001_18880  ------------------------------------------------------------
yinte0001_5480   LNNSGTLYSAGDQMQLSINGNLTNQGKLYAANGNVHLRAEGYLDNSGSLYSAG-------
yfred0001_33200  ------------------------------------------------------------
yrohd0001_18890  ------------------------------------------------------------
yfred0001_32960  ------------------------------------------------------------
yberc0001_40160  ------------------------------------------------------------
ypseu0001X_3843  ------------------------------------------------------------
ypseu0001X_3837  ------------------------------------------------------------
ypest0001X_8140  ------------------------------------------------------------
ypseu0001X_3848  LNNSGMLYSAGDQMRLSIAGNLTNEGKLHVANGEMRLLTEGNLDNRGSLYGAG-------
ypest0001X_8130  ------------------------------------------------------------
ypest0001X_8080  QANAISLTAKSGDITLTSAVIKAATQLLVTQLAATR------------------------
yinte0001_41760  SGTLAASTLALKAHNLDNRGLMQADVNAIIDAQTFNNRAEGRLLAGGALTLQGVQLNNAG
ykris0001_32060  SGTLAANALALKTHNLDNSGLVQADVNAIIDAQTLTNRDTGRLLAGNALTLQGVQLNNAG
ypseu0001X_3841  ------------------------------------------------------------
yrohd0001_38400  ------------------------------------------------------------
yfred0001_40710  ------------------------------------------------------------
yente0001X_8000  ------------------------------------------------------------
yruck0001_35040  ------------------------------------------------------------
                                                                             


                       1270      1280      1290      1300      1310      1320
                 =========+=========+=========+=========+=========+=========+
yberc0001_8630   ----NSELTVQGNAVNSGSVYTAGALQWQSGASVHNSGSIAALGNLQLSASDLLSSTPSL
ykris0001_26670  ------------------------------------------------------------
ykris0001_41280  ------------------------------------------------------------
yruck0001_910    ------------------------------------------------------------
yaldo0001_37900  ------------------------------------------------------------
yberc0001_34750  ------------------------------------------------------------
yaldo0001_38900  ------------------------------------------------------------
yaldo0001_41000  ------------------------------------------------------------
ypseu0001X_3846  ------------------------------------------------------------
ymoll0001_35980  ------------------------------------------------------------
ypest0001X_8100  ------------------------------------------------------------
yfred0001_38200  ------------------------------------------------------------
ypest0001X_2754  ------------------------------------------------------------
yfred0001_34120  ------------------------------------------------------------
ypseu0001X_2842  ------------------------------------------------------------
ykris0001_7890   ------------------------------------------------------------
yruck0001_4640   ------------------------------------------------------------
ymoll0001_2720   ------------------------------------------------------------
yrohd0001_40310  ------------------------------------------------------------
yruck0001_34980  ------------------------------------------------------------
ypest0001X_2756  ------------------------------------------------------------
yfred0001_45620  ------------------------------------------------------------
yrohd0001_18860  ------------------------------------------------------------
yfred0001_33010  ------------------------------------------------------------
yfred0001_33210  ------------------------------------------------------------
yrohd0001_40300  ------------------------------------------------------------
yruck0001_4620   ------------------------------------------------------------
ypest0001X_8110  ------------------------------------------------------------
yberc0001_40130  ------------------------------------------------------------
yberc0001_40750  ------------------------------------------------------------
yruck0001_35050  ------------------------------------------------------------
yruck0001_13030  ------------------------------------------------------------
yinte0001_17980  ------------------------------------------------------------
yaldo0001_6040   ------------------------------------------------------------
yfred0001_33270  ------------------------------------------------------------
yrohd0001_18870  ------------------------------------------------------------
yruck0001_4610   ------------------------------------------------------------
ypest0001X_2758  ------------------------------------------------------------
ypseu0001X_2843  ------------------------------------------------------------
ypseu0001X_2846  ------------------------------------------------------------
ypest0001X_2761  ------------------------------------------------------------
yrohd0001_32190  ------------------------------------------------------------
ymoll0001_21160  ------------------------------------------------------------
yrohd0001_32220  ------------------------------------------------------------
yfred0001_34080  ------------------------------------------------------------
yruck0001_4650   ------------------------------------------------------------
yaldo0001_38920  ------------------------------------------------------------
yberc0001_20820  ------------------------------------------------------------
yaldo0001_3710   ----NSELITHGNAVNSGSVYSQGALQWQSGGSVVNSGSIAALGNLQLSANDLLSSTPSL
ypest0001X_8090  ------------------------------------------------------------
yfred0001_34100  ------------------------------------------------------------
yruck0001_25350  ------------------------------------------------------------
yfred0001_38190  ------------------------------------------------------------
yfred0001_34090  ------------------------------------------------------------
yaldo0001_30990  ------------------------------------------------------------
yberc0001_36600  ------------------------------------------------------------
yrohd0001_40100  ------------------------------------------------------------
yrohd0001_40280  ------------------------------------------------------------
yruck0001_4660   ------------------------------------------------------------
ypseu0001X_3844  ------------------------------------------------------------
ykris0001_41250  ------------------------------------------------------------
ykris0001_21250  ------------------------------------------------------------
yruck0001_4630   ------------------------------------------------------------
yrohd0001_40320  ------------------------------------------------------------
yrohd0001_40080  ------------------------------------------------------------
yrohd0001_32210  ------------------------------------------------------------
yfred0001_45640  ------------------------------------------------------------
yfred0001_38220  ------------------------------------------------------------
yrohd0001_38410  ----NSELTVHGNAANSGSVYTQGALQWQSGGSVANSGSIAALGDFQLKASDLLSSNPSM
yfred0001_33220  ----NSELTVHGNAANSGSVYTQGTLQWQSDGSVANSGSIAALGDLQLKASDLLSSNPSM
ymoll0001_36970  ------------------------------------------------------------
yrohd0001_39710  ------------------------------------------------------------
yrohd0001_18880  ------------------------------------------------------------
yinte0001_5480   ----NSELITHGDAVNSGSIYTQGALQWQADGRVGNSGSIAALRDLQLRANDLFSTHQSL
yfred0001_33200  ------------------------------------------------------------
yrohd0001_18890  ------------------------------------------------------------
yfred0001_32960  ------------------------------------------------------------
yberc0001_40160  ------------------------------------------------------------
ypseu0001X_3843  ------------------------------------------------------------
ypseu0001X_3837  ------------------------------------------------------------
ypest0001X_8140  ------------------------------------------------------------
ypseu0001X_3848  ----NSDITTQGNAVNTGSVYTQGALQWLTKGSVRNSGSIAALGDLQLRANDLLSDNQSL
ypest0001X_8130  ------------------------------------------------------------
ypest0001X_8080  ------------------------------------------------------------
yinte0001_41760  KLQANRLNIETQTWDNTGSALGISQLTANATQSLTNSGQLLSQGPITLSTESLTNNGKIL
ykris0001_32060  KLQANRLNIETQTWDNTGSALGISQLTANATQKLTNRGQLLSQGPLTLNTKSLTNNGKIL
ypseu0001X_3841  ------------------------------------------------------------
yrohd0001_38400  ------------------------------------------------------------
yfred0001_40710  ------------------------------------------------------------
yente0001X_8000  ------------------------------------------------------------
yruck0001_35040  ------------------------------------------------------------
                                                                             


                       1330      1340      1350      1360      1370      1380
                 =========+=========+=========+=========+=========+=========+
yberc0001_8630   IAAGLTADGSHANSGDLTITTEHDLIAQGQNIAAGTLALSGSQLDLTGSQTQANAITLAA
ykris0001_26670  ------------------------------------------------------------
ykris0001_41280  ------------------------------------------------------------
yruck0001_910    ------------------------------------------------------------
yaldo0001_37900  ------------------------------------------------------------
yberc0001_34750  ------------------------------------------------------------
yaldo0001_38900  ------------------------------------------------------------
yaldo0001_41000  ------------------------------------------------------------
ypseu0001X_3846  ------------------------------------------------------------
ymoll0001_35980  ------------------------------------------------------------
ypest0001X_8100  ------------------------------------------------------------
yfred0001_38200  ------------------------------------------------------------
ypest0001X_2754  ------------------------------------------------------------
yfred0001_34120  ------------------------------------------------------------
ypseu0001X_2842  ------------------------------------------------------------
ykris0001_7890   ------------------------------------------------------------
yruck0001_4640   ------------------------------------------------------------
ymoll0001_2720   ------------------------------------------------------------
yrohd0001_40310  ------------------------------------------------------------
yruck0001_34980  ------------------------------------------------------------
ypest0001X_2756  ------------------------------------------------------------
yfred0001_45620  ------------------------------------------------------------
yrohd0001_18860  ------------------------------------------------------------
yfred0001_33010  ------------------------------------------------------------
yfred0001_33210  ------------------------------------------------------------
yrohd0001_40300  ------------------------------------------------------------
yruck0001_4620   ------------------------------------------------------------
ypest0001X_8110  ------------------------------------------------------------
yberc0001_40130  ------------------------------------------------------------
yberc0001_40750  ------------------------------------------------------------
yruck0001_35050  ------------------------------------------------------------
yruck0001_13030  ------------------------------------------------------------
yinte0001_17980  ------------------------------------------------------------
yaldo0001_6040   ------------------------------------------------------------
yfred0001_33270  ------------------------------------------------------------
yrohd0001_18870  ------------------------------------------------------------
yruck0001_4610   ------------------------------------------------------------
ypest0001X_2758  ------------------------------------------------------------
ypseu0001X_2843  ------------------------------------------------------------
ypseu0001X_2846  ------------------------------------------------------------
ypest0001X_2761  ------------------------------------------------------------
yrohd0001_32190  ------------------------------------------------------------
ymoll0001_21160  ------------------------------------------------------------
yrohd0001_32220  ------------------------------------------------------------
yfred0001_34080  ------------------------------------------------------------
yruck0001_4650   ------------------------------------------------------------
yaldo0001_38920  ------------------------------------------------------------
yberc0001_20820  ------------------------------------------------------------
yaldo0001_3710   IAAGLKADGSRSSHGDLTVSTQHALIAQGQNIAAGALALSGSLLDLTASQTQANAITLVA
ypest0001X_8090  ------------------------------------------------------------
yfred0001_34100  ------------------------------------------------------------
yruck0001_25350  ------------------------------------------------------------
yfred0001_38190  ------------------------------------------------------------
yfred0001_34090  ------------------------------------------------------------
yaldo0001_30990  ------------------------------------------------------------
yberc0001_36600  ------------------------------------------------------------
yrohd0001_40100  ------------------------------------------------------------
yrohd0001_40280  ------------------------------------------------------------
yruck0001_4660   ------------------------------------------------------------
ypseu0001X_3844  ------------------------------------------------------------
ykris0001_41250  ------------------------------------------------------------
ykris0001_21250  ------------------------------------------------------------
yruck0001_4630   ------------------------------------------------------------
yrohd0001_40320  ------------------------------------------------------------
yrohd0001_40080  ------------------------------------------------------------
yrohd0001_32210  ------------------------------------------------------------
yfred0001_45640  ------------------------------------------------------------
yfred0001_38220  ------------------------------------------------------------
yrohd0001_38410  IAAGLTADGSRANSSDLTITTEHTLIAQGQNIATGTLALSGSQLDLTGSQTQANAITLAA
yfred0001_33220  IAAGLTANGSRANNSDLTITTEHTLIAQGQNIATGTLALSGSQLDLTASQTQANAITLAA
ymoll0001_36970  ------------------------------------------------------------
yrohd0001_39710  ------------------------------------------------------------
yrohd0001_18880  ------------------------------------------------------------
yinte0001_5480   IGAGLQSDGNRASSGDLTVSTEHALIAQGQNMAAGALALSGSQLDLTGSQTQANTINFVA
yfred0001_33200  ------------------------------------------------------------
yrohd0001_18890  ------------------------------------------------------------
yfred0001_32960  ------------------------------------------------------------
yberc0001_40160  ------------------------------------------------------------
ypseu0001X_3843  ------------------------------------------------------------
ypseu0001X_3837  ------------------------------------------------------------
ypest0001X_8140  ------------------------------------------------------------
ypseu0001X_3848  MAAGLKADGSRSDSGNLAVSTEQALIAQGQNIAAGSLALAGSQIDLTGSQTQANAISLTA
ypest0001X_8130  ------------------------------------------------------------
ypest0001X_8080  ------------------------------------------------------------
yinte0001_41760  SESQLSLSAQEFINQGEAQGTTTQLNAEQLTNRGHLIGVERLMLQLQQDLNNATGGKLLS
ykris0001_32060  SENQLNLNAQQFINQGEAQGTTTQLNAEQLTNSGHLIGVERLVLQLQQDLNNATGGKLLS
ypseu0001X_3841  ------------------------------------------------------------
yrohd0001_38400  ------------------------------------------------------------
yfred0001_40710  ------------------------------------------------------------
yente0001X_8000  ------------------------------------------------------------
yruck0001_35040  ------------------------------------------------------------
                                                                             


                       1390      1400      1410      1420      1430      1440
                 =========+=========+=========+=========+=========+=========+
yberc0001_8630   KSADITLTEAVV------------------------------------KAATQLSANTST
ykris0001_26670  ------------------------------------------------------------
ykris0001_41280  ------------------------------------------------------------
yruck0001_910    ------------------------------------------------------------
yaldo0001_37900  ------------------------------------------------------------
yberc0001_34750  ------------------------------------------------------------
yaldo0001_38900  ------------------------------------------------------------
yaldo0001_41000  ------------------------------------------------------------
ypseu0001X_3846  ------------------------------------------------------------
ymoll0001_35980  ------------------------------------------------------------
ypest0001X_8100  ------------------------------------------------------------
yfred0001_38200  ------------------------------------------------------------
ypest0001X_2754  ------------------------------------------------------------
yfred0001_34120  ------------------------------------------------------------
ypseu0001X_2842  ------------------------------------------------------------
ykris0001_7890   ------------------------------------------------------------
yruck0001_4640   ------------------------------------------------------------
ymoll0001_2720   ------------------------------------------------------------
yrohd0001_40310  ------------------------------------------------------------
yruck0001_34980  ------------------------------------------------------------
ypest0001X_2756  ------------------------------------------------------------
yfred0001_45620  ------------------------------------------------------------
yrohd0001_18860  ------------------------------------------------------------
yfred0001_33010  ------------------------------------------------------------
yfred0001_33210  ------------------------------------------------------------
yrohd0001_40300  ------------------------------------------------------------
yruck0001_4620   ------------------------------------------------------------
ypest0001X_8110  ------------------------------------------------------------
yberc0001_40130  ------------------------------------------------------------
yberc0001_40750  ------------------------------------------------------------
yruck0001_35050  ------------------------------------------------------------
yruck0001_13030  ------------------------------------------------------------
yinte0001_17980  ------------------------------------------------------------
yaldo0001_6040   ------------------------------------------------------------
yfred0001_33270  ------------------------------------------------------------
yrohd0001_18870  ------------------------------------------------------------
yruck0001_4610   ------------------------------------------------------------
ypest0001X_2758  ------------------------------------------------------------
ypseu0001X_2843  ------------------------------------------------------------
ypseu0001X_2846  ------------------------------------------------------------
ypest0001X_2761  ------------------------------------------------------------
yrohd0001_32190  ------------------------------------------------------------
ymoll0001_21160  ------------------------------------------------------------
yrohd0001_32220  ------------------------------------------------------------
yfred0001_34080  ------------------------------------------------------------
yruck0001_4650   ------------------------------------------------------------
yaldo0001_38920  ------------------------------------------------------------
yberc0001_20820  ------------------------------------------------------------
yaldo0001_3710   KSGDITLTQAVV------------------------------------KAVTQLSASTTA
ypest0001X_8090  ------------------------------------------------------------
yfred0001_34100  ------------------------------------------------------------
yruck0001_25350  ------------------------------------------------------------
yfred0001_38190  ------------------------------------------------------------
yfred0001_34090  ------------------------------------------------------------
yaldo0001_30990  ------------------------------------------------------------
yberc0001_36600  ------------------------------------------------------------
yrohd0001_40100  ------------------------------------------------------------
yrohd0001_40280  ------------------------------------------------------------
yruck0001_4660   ------------------------------------------------------------
ypseu0001X_3844  ------------------------------------------------------------
ykris0001_41250  ------------------------------------------------------------
ykris0001_21250  ------------------------------------------------------------
yruck0001_4630   ------------------------------------------------------------
yrohd0001_40320  ------------------------------------------------------------
yrohd0001_40080  ------------------------------------------------------------
yrohd0001_32210  ------------------------------------------------------------
yfred0001_45640  ------------------------------------------------------------
yfred0001_38220  ------------------------------------------------------------
yrohd0001_38410  KSGDITLTEAVV------------------------------------KAATQLSAGTTT
yfred0001_33220  KSGDITLTEAVV------------------------------------KAATQLSAGTTT
ymoll0001_36970  ------------------------------------------------------------
yrohd0001_39710  ------------------------------------------------------------
yrohd0001_18880  ------------------------------------------------------------
yinte0001_5480   KSGDITLTEAVV------------------------------------KAATQLSASTTA
yfred0001_33200  ------------------------------------------------------------
yrohd0001_18890  ------------------------------------------------------------
yfred0001_32960  ------------------------------------------------------------
yberc0001_40160  ------------------------------------------------------------
ypseu0001X_3843  ------------------------------------------------------------
ypseu0001X_3837  ------------------------------------------------------------
ypest0001X_8140  ------------------------------------------------------------
ypseu0001X_3848  KSGDITLTSAVIKAATQLLVTQLAATRSLFIPPSSIPPSSTRSSSTQSSSSTQASASPSA
ypest0001X_8130  ------------------------------------------------------------
ypest0001X_8080  ---------------------------------SSFIPPSSIPPSSTQSSSTQASASPSA
yinte0001_41760  GGDLKVNAGTVSNAGTWQGEQIILAAHQLDNTGTLQANNVIQLDLTGDINSGAGSQIITL
ykris0001_32060  GGDLKVNAAAVSNAGTWQGEQIILAARQLDNTGILQANNRIQLDLTGNVNSGVGSQIVTL
ypseu0001X_3841  ------------------------------------------------------------
yrohd0001_38400  ------------------------------------------------------------
yfred0001_40710  ------------------------------------------------------------
yente0001X_8000  ------------------------------------------------------------
yruck0001_35040  ------------------------------------------------------------
                                                                             


                       1450      1460      1470      1480      1490      1500
                 =========+=========+=========+=========+=========+=========+
yberc0001_8630   LLRTDKAHLIAEQITLAAQSLSNLGGVIAQTGATDFNLNLPGYL-----DNRGGTLLSKG
ykris0001_26670  ------------------------------------------------------------
ykris0001_41280  ------------------------------------------------------------
yruck0001_910    ------------------------------------------------------------
yaldo0001_37900  ------------------------------------------------------------
yberc0001_34750  -------------------------------------------------------MTVLA
yaldo0001_38900  ------------------------------------------------------------
yaldo0001_41000  ------------------------------------------------------------
ypseu0001X_3846  ------------------------------------------------------------
ymoll0001_35980  ------------------------------------------------------------
ypest0001X_8100  ------------------------------------------------------------
yfred0001_38200  ------------------------------------------------------------
ypest0001X_2754  ------------------------------------------------------------
yfred0001_34120  ------------------------------------------------------------
ypseu0001X_2842  ------------------------------------------------------------
ykris0001_7890   ------------------------------------------------------------
yruck0001_4640   ------------------------------------------------------------
ymoll0001_2720   ------------------------------------------------------------
yrohd0001_40310  ------------------------------------------------------------
yruck0001_34980  -------------------------------------------------DNRGGVLLAAG
ypest0001X_2756  ------------------------------------------------------------
yfred0001_45620  ------------------------------------------------------------
yrohd0001_18860  ------------------------------------------------------------
yfred0001_33010  ------------------------------------------------------------
yfred0001_33210  ------------------------------------------------------------
yrohd0001_40300  ------------------------------------------------------------
yruck0001_4620   ------------------------------------------------------------
ypest0001X_8110  ------------------------------------------------------------
yberc0001_40130  ------------------------------------------------------------
yberc0001_40750  ------------------------------------------------------------
yruck0001_35050  ------------------------------------------------------------
yruck0001_13030  -----------------------------------------------------------G
yinte0001_17980  ------------------------------------------------------------
yaldo0001_6040   ------------------------------------------------------------
yfred0001_33270  ------------------------------------------------------------
yrohd0001_18870  ------------------------------------------------------------
yruck0001_4610   ------------------------------------------------------------
ypest0001X_2758  ------------------------------------------------------------
ypseu0001X_2843  ------------------------------------------------------------
ypseu0001X_2846  -----------------------------------------GDV-----DNRGGTLLSSG
ypest0001X_2761  -----------------------------------------GDV-----DNRGGTLLSSG
yrohd0001_32190  ------------------------------------------------------------
ymoll0001_21160  ------------------------------------------------------------
yrohd0001_32220  ------------------------------------------------------------
yfred0001_34080  ------------------------------------------------------------
yruck0001_4650   ------------------------------------------------------------
yaldo0001_38920  ------------------------------------------------------------
yberc0001_20820  ------------------------------------------------------------
yaldo0001_3710   LLRSDKASLIAEQITLTAQALSNLGGVIAQTGITDFTLNLAGYL-----DNRAGTLLSKG
ypest0001X_8090  ------------------------------------------------------------
yfred0001_34100  ------------------------------------------------------------
yruck0001_25350  -------------------------------------LDAKGNIHLSGSTTQNHSAETGY
yfred0001_38190  ------------------------------------------------------------
yfred0001_34090  ------------------------------------------------------------
yaldo0001_30990  ------------------------------------------------------------
yberc0001_36600  ------------------------------------------------------------
yrohd0001_40100  ------------------------------------------------------------
yrohd0001_40280  ------------------------------------------------------------
yruck0001_4660   ------------------------------------------------------------
ypseu0001X_3844  ------------------------------------------------------------
ykris0001_41250  ------------------------------------------------------------
ykris0001_21250  ------------------------------------------------------------
yruck0001_4630   ------------------------------------------------------------
yrohd0001_40320  ------------------------------------------------------------
yrohd0001_40080  ------------------------------------------------------------
yrohd0001_32210  -----------------------------------------DYL-----DNRGGTLLSKG
yfred0001_45640  -----------------------------------------GYI-----DNRDGTLLSTG
yfred0001_38220  ------------------------------------------------------------
yrohd0001_38410  LLRADKASLIADQITLTAQSLSNLGGVIAQTGITDFNLNLAGYL-----DNRAGTLLSKG
yfred0001_33220  LLRTDKASLIADQITLTVQSLSNFGGVIAQTGITDFNLNLAGYL-----DNRAGTLLSKG
ymoll0001_36970  -----------------------------------------DYL-----DNRSGTLLSKG
yrohd0001_39710  ------------------------------------------------------------
yrohd0001_18880  ------------------------------------------------------------
yinte0001_5480   LLRTDKANLMAERVTLSAQSLSNLGGVIAQTGIMDFTLNLAGYL-----DNRAGTLLSKG
yfred0001_33200  ------------------------------------------------------------
yrohd0001_18890  ------------------------------------------------------------
yfred0001_32960  ------------------------------------------------------------
yberc0001_40160  ------------------------------------------------------------
ypseu0001X_3843  ------------------------------------------------------------
ypseu0001X_3837  ------------------------------------------------------------
ypest0001X_8140  ------------------------------------------------------------
ypseu0001X_3848  LLRTDKASLIADQLTFDVQALSNLGGVIAQTGATDFNLNLPGYL-----DNRGGTILSKG
ypest0001X_8130  ------------------------------------------------------------
ypest0001X_8080  WLRTDKASLIADQLTFDVQALSNLGGVIAQTGATDFNLNLPGYL-----DNRGGTILSKG
yinte0001_41760  GEAAINALTLVNHGNWQATSLFLKGDSLLNSGVIVGVNQLKSEISDGITQQKNGEMLSKG
ykris0001_32060  GEATINALALVNHGNWQAASLFLKGDSLLNNGVIAGVNQLKSEISRDITQQKSGEMLSNG
ypseu0001X_3841  ------------------------------------------------------------
yrohd0001_38400  ------------------------------------------------------------
yfred0001_40710  ------------------------------------------------------------
yente0001X_8000  ------------------------------------------------------------
yruck0001_35040  ------------------------------------------------------------
                                                                             


                       1510      1520      1530      1540      1550      1560
                 =========+=========+=========+=========+=========+=========+
yberc0001_8630   NVAVNAQRLDSNSTSLLAAGVQSDGRLTDAGDLAVTTGQDLIAQGQTLAAGAMTLTGSRV
ykris0001_26670  ------------------------------------------------------------
ykris0001_41280  ------------------------------------------------------------
yruck0001_910    ------------------------------------------------------------
yaldo0001_37900  ------------------------------------------------------------
yberc0001_34750  SARTNAIYIATLFAGIFGVSAYGLTSFAGKSMQQLDRASNESERSLTNEGRGGLIEGAKS
yaldo0001_38900  ------------------------------------------------------------
yaldo0001_41000  ------------------------------------------------------------
ypseu0001X_3846  ------------------------------------------------------------
ymoll0001_35980  ------------------------------------------------------------
ypest0001X_8100  ------------------------------------------------------------
yfred0001_38200  ------------------------------------------------------------
ypest0001X_2754  ------------------------------------------------------------
yfred0001_34120  ------------------------------------------------------------
ypseu0001X_2842  ------------------------------------------------------------
ykris0001_7890   ------------------------------------------------------------
yruck0001_4640   ------------------------------------------------------------
ymoll0001_2720   ------------------------------------------------------------
yrohd0001_40310  ------------------------------------------------------------
yruck0001_34980  NMRLQADKLTSNDHSLLGAGIHADGRQXXN------------------------------
ypest0001X_2756  ------------------------------------------------------------
yfred0001_45620  ------------------------------------------------------------
yrohd0001_18860  ------------------------------------------------------------
yfred0001_33010  ------------------------------------------------------------
yfred0001_33210  ------------------------------------------------------------
yrohd0001_40300  ------------------------------------------------------------
yruck0001_4620   --------------------------------LQVNTAQALMAQGQNVAVDALTLSGSQI
ypest0001X_8110  ------------------------------------------------------------
yberc0001_40130  ------------------------------------------------------------
yberc0001_40750  ------------------------------------------------------------
yruck0001_35050  ------------------------------------------------------------
yruck0001_13030  NVRISATDGDVTAGALLVNAGNELNVLASGSINTHGISGLNVNRKHIRKVDSLFYAEKDK
yinte0001_17980  --------------------------------------ERTIYSGGAHYDGGIDMFGSGV
yaldo0001_6040   ------------------------------------------------------------
yfred0001_33270  ------------------------------------------------------------
yrohd0001_18870  ------------------------------------------------------------
yruck0001_4610   ------------------------------------------------------------
ypest0001X_2758  ------------------------------------------------------------
ypseu0001X_2843  ------------------------------------------------------------
ypseu0001X_2846  TLSLQAESLNSNGNSLLGAGVQSDGRLTEIGDLRVTTRQDLIAHGQTLAAGAMALTGSRI
ypest0001X_2761  TLSLQAESLNSNGNSLLGAGVQSDGRLTEIGDLRVTTRQDLIAHGQTLAAGAMALTGSRI
yrohd0001_32190  ------------------------------------------------------------
ymoll0001_21160  ------------------------------------------------------------
yrohd0001_32220  ------------------------------------------------------------
yfred0001_34080  ------------------------------------------------------------
yruck0001_4650   ------------------------------------------------------------
yaldo0001_38920  ------------------------------------------------------------
yberc0001_20820  ------------------------------------------------------------
yaldo0001_3710   NVAIQAQRLDSDSGSLLAAGVQSNGRLTDTGDLRVVTGQDLNAQGQTLAAGAMTLTGSRV
ypest0001X_8090  ------------------------------------------------------------
yfred0001_34100  ------------------------------------------------------------
yruck0001_25350  HKNDTAKLLTGNWHNSTQEENLARTQLTAGDNLKLSAGHDVTARGAQVHAGK--------
yfred0001_38190  ------------------------------------------------------------
yfred0001_34090  ------------------------------------------------------------
yaldo0001_30990  ------------------------------------------------------------
yberc0001_36600  ------------------------------------------------------------
yrohd0001_40100  ------------------------------------------------------------
yrohd0001_40280  ------------------------------------------------------------
yruck0001_4660   ------------------------------------------------------------
ypseu0001X_3844  ------------------------------------------------------------
ykris0001_41250  ------------------------------------------------------------
ykris0001_21250  ------------------------------------------------------------
yruck0001_4630   ------------------------------------------------------------
yrohd0001_40320  ------------------------------------------------------------
yrohd0001_40080  ------------------------------------------------------------
yrohd0001_32210  HVAINAQRLESNSDSLLAAGVQSDGRLMEAGDLVVTTRQELIAQGQTLAAGAMTLSGSRV
yfred0001_45640  TLSLQAEGVNSNSNSLLGAGIQSDGRLTESGNLLVNTRQELIAQGQTLAADTMILTGSRI
yfred0001_38220  ------------------------------------------------------------
yrohd0001_38410  DVAVQAQRLDSNSTSLLGAGIQSDGRLTDTGNLAVSTHQDLIAQGQTLAAGAMTLTGSRV
yfred0001_33220  NVAVQAQRLDSNSTSLLGAGIQSDGRLTDAGNLAVTTHQDLIAQGQTLAAGAMTLTGSRV
ymoll0001_36970  NVAVNALRLDSDSGSLLAAGVQSDGRLMDAGDLAVTTTQELIAQGQTLAAGGMTLTGSRI
yrohd0001_39710  ------------------------------------------------------------
yrohd0001_18880  ------------------------------------------------------------
yinte0001_5480   NVTVQTERLDSDGDSLLGAGVQSNGRLTDTGDLVVATGQDLNAQGQNIAAGAITLTGSRV
yfred0001_33200  ------------------------------------------------------------
yrohd0001_18890  ------------------------------------------------------------
yfred0001_32960  ------------------------------------------------------------
yberc0001_40160  ------------------------------------------------------------
ypseu0001X_3843  ------------------------------------------------------------
ypseu0001X_3837  ------------------------------------------------------------
ypest0001X_8140  ------------------------------------------------------------
ypseu0001X_3848  NVAIQAQGLDSDSGSLLGAGVQSDGKLTNAGDLAVTVRQDLIAHGQSLAAGAMTLTGSRV
ypest0001X_8130  ------------------------------------------------------------
ypest0001X_8080  NVAIQAQGLDSDSGSLLGAGVQSDGKLTNAGDLAVTVRQDLIAHGQSLAAGAMTLTGSGV
yinte0001_41760  LLTLNATQVDNQGRIQASSLTLHTADITNTGVMQGQDTFSAQLSGVFHNLASGDLRSQNG
ykris0001_32060  LLTLNATQVDNQGRIQANSLALHAVDMTNNGVMQGQDVFNAQLSGVFHNLASGDLRSQNG
ypseu0001X_3841  ------------------------------------------------------------
yrohd0001_38400  ------------------------------------------------------------
yfred0001_40710  ------------------------------------------------------------
yente0001X_8000  ------------------------------------------------------------
yruck0001_35040  ------------------------------------------------------------
                                                                             


                       1570      1580      1590      1600      1610      1620
                 =========+=========+=========+=========+=========+=========+
yberc0001_8630   SLADSHTQAREMNIIANSGDVSTQRANIISLGSLTISAGANAGQTLNNQGGALAANNISL
ykris0001_26670  ------------------------------------------------------------
ykris0001_41280  ------------------------------------------------------------
yruck0001_910    ------------------------------------------------------------
yaldo0001_37900  ------------------------------------------------------------
yberc0001_34750  GLASQKAYADIGDIDMMARGSATGELSSIKSNNAQVDA----------LGGDLSSATTRM
yaldo0001_38900  ------------------------------------------------------------
yaldo0001_41000  ------------------------------------------------------------
ypseu0001X_3846  ------------------------------------------------------------
ymoll0001_35980  ------------------------------------------------------------
ypest0001X_8100  ------------------------------------------------------------
yfred0001_38200  ------------------------------------------------------------
ypest0001X_2754  ------------------------------------------------------------
yfred0001_34120  ------------------------------------------------------------
ypseu0001X_2842  ------------------------------------------------------------
ykris0001_7890   ------------------------------------------------------------
yruck0001_4640   ------------------------------------------------------------
ymoll0001_2720   ------------------------------------------------------------
yrohd0001_40310  ------------------------------------------------------------
yruck0001_34980  ------------------------------------------------------------
ypest0001X_2756  ------------------------------------------------------------
yfred0001_45620  ------------------------------------------------------------
yrohd0001_18860  ------------------------------------------------------------
yfred0001_33010  ------------------------------------------------------------
yfred0001_33210  ------------------------------------------------------------
yrohd0001_40300  ------------------------------------------------------------
yruck0001_4620   DLTGSQTQASNIALTARDGDVVTREATVLTPGTLAITAAANREQNLDNRGGKLHANNLRL
ypest0001X_8110  ------------------------------------------------------------
yberc0001_40130  ------------------------------------------------------------
yberc0001_40750  ------------------------------------------------------------
yruck0001_35050  ------------------------------------------------------------
yruck0001_13030  NNKLIKRQFLEKNHFFANKNMNLNARDNINLTALTTHAG---GDLFIHSDGTVNINVQKT
yinte0001_17980  EGKYSYYEHHKMTNTSIASKTDINGNMLITAGEDITHQGAQHQVNGKYQESAKNIYHMAS
yaldo0001_6040   ------------------------------------------------------------
yfred0001_33270  ------------------------------------------------------------
yrohd0001_18870  ------------------------------------------------------------
yruck0001_4610   ------------------------------------------------------------
ypest0001X_2758  ------------------------------------------------------------
ypseu0001X_2843  ------------------------------------------------------------
ypseu0001X_2846  DLADSYTQAREMTLTANRGDISTQRATVLALDTLSI----NTAQTLNNQGGTLAGNTLAL
ypest0001X_2761  DLADSYTQAREMTLTANRGDISTQRATVLALDTLSI----NTAQTLNNQGGTLAGNTLAL
yrohd0001_32190  ------------------------------------------------------------
ymoll0001_21160  ------------------------------------------------------------
yrohd0001_32220  ------------------------------------------------------------
yfred0001_34080  ------------------------------------------------------------
yruck0001_4650   ------XQAREIKISANKGDVTTQRANMVSLGSLTINAGANLGQTLNNQGGTLQANNIYL
yaldo0001_38920  ------------------------------------------------------------
yberc0001_20820  ------------------------------------------------------------
yaldo0001_3710   DLTGSHTQAREMNITAHSGDVSTQRANIISLGTLTINAGANAGQTLNNQGGALTANNIAL
ypest0001X_8090  ------------------------------------------------------------
yfred0001_34100  ------------------------------------------------------------
yruck0001_25350  -------------------NLDVTAGNKISVDVQKTANTHTIKNDKTSWGGIGGGENQDN
yfred0001_38190  ------------------------------------------------------------
yfred0001_34090  ------------------------------------------------------------
yaldo0001_30990  ------------------------------------------------------------
yberc0001_36600  ------------------------------------------------------------
yrohd0001_40100  ------------------------------------------------------------
yrohd0001_40280  ------------------------------------------------------------
yruck0001_4660   ------------------------------------------------------------
ypseu0001X_3844  ------------------------------------------------------------
ykris0001_41250  ------------------------------------------------------------
ykris0001_21250  ------------------------------------------------------------
yruck0001_4630   ------------------------------------------------------------
yrohd0001_40320  ------------------------------------------------------------
yrohd0001_40080  ------------------------------------------------------------
yrohd0001_32210  DLTDSYTQAREMNITANSGDISTQRANILALGSLTINAGA---QTLNNQNGTLAANNIAL
yfred0001_45640  DLSDSYTQAREMNITATQGDISTQRANILALGTLTINASANAGQTLNNQGGVLAANSIHL
yfred0001_38220  ------------------------------------------------------------
yrohd0001_38410  SLADSHTQAREVNIIANSGDVSTQRANIISLGSLTITAGANAAQKLNNQGGALAANNISL
yfred0001_33220  SLADSHTQAREMNIIANSGDVSTQRAKIISLGSLTITAGANAAQKLNNQGGALAANNISL
ymoll0001_36970  DLTDSHTQAREMNITANSGDISTQRAHILSLGSLTLNAGASAGQTLNNQSGTLAANDIAL
yrohd0001_39710  ------------------------------------------------------------
yrohd0001_18880  ------------------------------------------------------------
yinte0001_5480   DLTGSQTQAREINITANSGDISTQRANILSLGTLTINAGANAGQTLHNQGGALAANNISL
yfred0001_33200  ------------------------------------------------------------
yrohd0001_18890  ------------------------------------------------------------
yfred0001_32960  ------------------------------------------------------------
yberc0001_40160  ------------------------------------------------------------
ypseu0001X_3843  ------------------------------------------------------------
ypseu0001X_3837  ------------------------------------------------------------
ypest0001X_8140  ------------------------------------------------------------
ypseu0001X_3848  DLTGSQTQARGITITANKGDVSTQRANILSLGSLAINAGANAGQTLNNQGGALQASNIAL
ypest0001X_8130  ------------------------------------------------------------
ypest0001X_8080  DLTGSQTQARGITITANKGDVSTQRANILSLGSLAINAGANAGQTLNNQGGSLQANNIAL
yinte0001_41760  LNLNAAGLDNAGNIQGAGASTFVLTTPMLNTGKIVVGGDLDIGSLALNNSGWLQANNITF
ykris0001_32060  LKLNAAGLDNAGNIQSTEASTFVLTTPMLNTGKIVVGGDLDIGALALNNSGWLQANNITF
ypseu0001X_3841  ------------------------------------------------------------
yrohd0001_38400  ------------------------------------------------------------
yfred0001_40710  ------------------------------------------------------------
yente0001X_8000  ------------------------------------------------------------
yruck0001_35040  ------------------------------------------------------------
                                                                             


                       1630      1640      1650      1660      1670      1680
                 =========+=========+=========+=========+=========+=========+
yberc0001_8630   NLGQFDGGAGKVTASQD-LTIGLLSDFNNLAGSTLKAGRDLTFTTHGALTNDGQLLAGRK
ykris0001_26670  ------------------------------------------------------------
ykris0001_41280  ------------------------------------------------------------
yruck0001_910    ------------------------------------------------------------
yaldo0001_37900  ------------------------------------------------------------
yberc0001_34750  SQVSAGAATGNVRGTEQ-------------------------------------------
yaldo0001_38900  ------------------------------------------------------------
yaldo0001_41000  ------------------------------------------------------------
ypseu0001X_3846  ------------------------------------------------------------
ymoll0001_35980  ------------------------------------------------------------
ypest0001X_8100  ------------------------------------------------------------
yfred0001_38200  ------------------------------------------------------------
ypest0001X_2754  ------------------------------------------------------------
yfred0001_34120  ------------------------------------------------------------
ypseu0001X_2842  ------------------------------------------------------------
ykris0001_7890   ------------------------------------------------------------
yruck0001_4640   ------------------------------------------------------------
ymoll0001_2720   ------------------------------------------------------------
yrohd0001_40310  ------------------------------------------------------------
yruck0001_34980  ------------------------------------------------------------
ypest0001X_2756  ------------------------------------------------------------
yfred0001_45620  ------------------------------------------------------------
yrohd0001_18860  ------------------------------------------------------------
yfred0001_33010  ------------------------------------------------------------
yfred0001_33210  ------------------------------------------------------------
yrohd0001_40300  ------------------------------------------------------------
yruck0001_4620   DLARLDNGTEKXX-----------------------------------------------
ypest0001X_8110  ------------------------------------------------------------
yberc0001_40130  ------------------------------------------------------------
yberc0001_40750  ------------------------------------------------------------
yruck0001_35050  ------------------------------------------------------------
yruck0001_13030  KNTEIDNYDKDKFFEIGGEDKDNHSDSYEISHRTELTGRDIHITSGNNMQIFGAKIDARR
yinte0001_17980  NDKTISNSTEKEIKSGLGFNIN-YSKYTRQIEKIIKDPANVLHHLGGTGSIKGISDPNAG
yaldo0001_6040   ------------------------------------------------------------
yfred0001_33270  ------------------------------------------------------------
yrohd0001_18870  ------------------------------------------------------------
yruck0001_4610   ------------------------------------------------------------
ypest0001X_2758  ------------------------------------------------------------
ypseu0001X_2843  ------------------------------------------------------------
ypseu0001X_2846  DLGQFDNQGGQVTASQD-LTIDLQRDFSHQAGSTLQAGRDLTLTSLGAVTNDGHLVAGGT
ypest0001X_2761  DLGQFDNQGGQVTASQD-LTIDLQRDFSHQAGSTLQAGRDLTLTSLGAVTNDGQLVAGGT
yrohd0001_32190  ------------------------------------------------------------
ymoll0001_21160  ------------------------------------------------------------
yrohd0001_32220  ------------------------------------------------------------
yfred0001_34080  ------------------------------------------------------------
yruck0001_4650   NLGRFDGSEGNIRASQD-LTIGLQSDFSHLADSKLQAGRDLTFTTTGALTNDGQLVAGRK
yaldo0001_38920  ------------------------------------------------------------
yberc0001_20820  ------------------------------------------------------------
yaldo0001_3710   HLGRFDSSAGKITASQD-LAIGLQSDFSNQAGSTLQAGRDLTLSTDGALTNDGQLVAGGK
ypest0001X_8090  ------------------------------------------------------------
yfred0001_34100  ------------------------------------------------------------
yruck0001_25350  GRLNEVSHSSELTAGNELFLSGQQGVSITGSKVKAVNGGYVDTKMGGLLIDNAVSTSTDK
yfred0001_38190  ------------------------------------------------------------
yfred0001_34090  ------------------------------------------------------------
yaldo0001_30990  ------------------------------------------------------------
yberc0001_36600  ------------------------------------------------------------
yrohd0001_40100  ------------------------------------------------------------
yrohd0001_40280  ------------------------------------------------------------
yruck0001_4660   ------------------------------------------------------------
ypseu0001X_3844  ------------------------------------------------------------
ykris0001_41250  ------------------------------------------------------------
ykris0001_21250  ------------------------------------------------------------
yruck0001_4630   ---------------------------------VLQTGEEMQLSTQGDLNNSGTLYA---
yrohd0001_40320  ------------------------------------------------------------
yrohd0001_40080  ------------------------------------------------------------
yrohd0001_32210  NLGQLENSAGNITASQD-LTLNLQSNFTHLASSTLQAGRDFTFTTRGSLTNDGQLLAGGK
yfred0001_45640  KLSQLNNNAGKVTASED-LTINLQSDFNHLAGSTLQAGRDFTLTTAGDVTNSGQMLAGGK
yfred0001_38220  ------------------------------------------------------------
yrohd0001_38410  NLGQFDGSAGKVSASQD-LTIGLLSDFNNLAGSTLQAGRDLTFTTQGALTNGGQLLAGRK
yfred0001_33220  NLGQFDGSAGKVSASQD-LTIGLLSDFNNLAGSTLQAGRDLTFTTQGALTNGGQLLAGRK
ymoll0001_36970  NLGQLNSSAGKITASQD-LTIDLQSDFNNLADSTLQAGRDFSLTTAGALTNDGQLLAGGK
yrohd0001_39710  ------------------------------------------------------------
yrohd0001_18880  ------------------------------------------------------------
yinte0001_5480   NLGQFDGSAGKVSASQD-LTIGLLSDFNNLAGSTLQAGRDLTLSTDGTLTNDGQLLAGGK
yfred0001_33200  ------------------------------------------------------------
yrohd0001_18890  ------------------------------------------------------------
yfred0001_32960  ------------------------------------------------------------
yberc0001_40160  ------------------------------------------------------------
ypseu0001X_3843  ------------------------------------------------------------
ypseu0001X_3837  ------------------------------------------------------------
ypest0001X_8140  ------------------------------------------------------------
ypseu0001X_3848  NLGQLDNRTGKIAASQD-LTLGLQRDFNILADSTLQAGRDFSFTTHGALTNDGQLLAGRK
ypest0001X_8130  ------------------------------------------------------------
ypest0001X_8080  NLGQLDNRTGKIAASQD-LVLGLQSDFNILADSTLQAGRDFSFTTHGALTNDGQLLAGRK
yinte0001_41760  NGTRLDNTGTLIAAGDNLLTLDIFNNRGTVQGDNLQ-------MTIGSLNNAGTLLATRQ
ykris0001_32060  NGTRLDNSGTLIAAGDNRLTLDIFNNRGTVQGDNLQ-------LKIGSLNNAGTLLATRQ
ypseu0001X_3841  ------------------------------------------------------------
yrohd0001_38400  ------------------------------------------------------------
yfred0001_40710  ------------------------------------------------------------
yente0001X_8000  ------------------------------------------------------------
yruck0001_35040  ------------------------------------------------------------
                                                                             


                       1690      1700      1710      1720      1730      1740
                 =========+=========+=========+=========+=========+=========+
yberc0001_8630   LSTDST----------------------------------------TLFNRGSIIGAEAT
ykris0001_26670  ------------------------------------------------------------
ykris0001_41280  ------------------------------------------------------------
yruck0001_910    ------------------------------------------------------------
yaldo0001_37900  -------------------------------------------------KKESITSLKSE
yberc0001_34750  ---------------------------------------------------ATQPGQSAF
yaldo0001_38900  ------------------------------------------------------------
yaldo0001_41000  ------------------------------------------------------------
ypseu0001X_3846  ------------------------------------------------------------
ymoll0001_35980  ------------------------------------------------------------
ypest0001X_8100  ------------------------------------------------------------
yfred0001_38200  ------------------------------------------------------------
ypest0001X_2754  ------------------------------------------------------------
yfred0001_34120  ------------------------------------------------------------
ypseu0001X_2842  ------------------------------------------------------------
ykris0001_7890   ------------------------------------------------------------
yruck0001_4640   ------------------------------------------------------------
ymoll0001_2720   ------------------------------------------------------------
yrohd0001_40310  ------------------------------------------------------------
yruck0001_34980  ------------------------------------------------------------
ypest0001X_2756  ------------------------------------------------------------
yfred0001_45620  ------------------------------------------------------------
yrohd0001_18860  ------------------------------------------------------------
yfred0001_33010  ------------------------------------------------------------
yfred0001_33210  ------------------------------------------------------------
yrohd0001_40300  ------------------------------------------------------------
yruck0001_4620   ------------------------------------------------------------
ypest0001X_8110  ------------------------------------------------------------
yberc0001_40130  ------------------------------------------------------------
yberc0001_40750  ------------------------------------------------------------
yruck0001_35050  ------------------------------------------------------------
yruck0001_13030  ------------------------------------------------------------
yinte0001_17980  VDIYASGGKTTKSG------------------------------LSSLASVTEIAAADIT
yaldo0001_6040   ------------------------------------------------------------
yfred0001_33270  ------------------------------------------------------------
yrohd0001_18870  ------------------------------------------------------------
yruck0001_4610   ------------------------------------------------------------
ypest0001X_2758  ------------------------------------------------------------
ypseu0001X_2843  ------------------------------------------------------------
ypseu0001X_2846  LSTRSDSLLN--SGNLIATQAELNATGALINHGEILTLGGLDTDSNTLFNTGSIISAEAT
ypest0001X_2761  LSTHSDSLLN--SGNLIATQAELNATGALINHGEILTLGGLDTDSNTLFNTGSIISAEAT
yrohd0001_32190  ------------------------------------------------------------
ymoll0001_21160  ------------------------------------------------------------
yrohd0001_32220  ------------------------------------------------------------
yfred0001_34080  ------------------------------------------------------------
yruck0001_4650   LSTKTNSLLN--NGKILAVEANLRTVGAMINQGEILTSGWLASDTNTLFNSGSIIGAEVE
yaldo0001_38920  ------------------------------------------------------------
yberc0001_20820  ------------------------------------------------------------
yaldo0001_3710   LSTHSASLLN--NGNISATQARLTAAGALLNRGEILTRGRLDTDSHTLFNTGTLIGADAT
ypest0001X_8090  ------------------------------------------------------------
yfred0001_34100  ------------------------------------------------------------
yruck0001_25350  VNSRTGTVFNITSGSQKHDNVKQSSTQSELKSDTDLTL--------RSAKDVNVVGSQVT
yfred0001_38190  ------------------------------------------------------------
yfred0001_34090  ------------------------------------------------------------
yaldo0001_30990  ------------------------------------------------------------
yberc0001_36600  ------------------------------------------------------------
yrohd0001_40100  ------------------------------------------------------------
yrohd0001_40280  ------------------------------------------------------------
yruck0001_4660   ------------------------------------------------------------
ypseu0001X_3844  ------------------------------------------------------------
ykris0001_41250  ------------------------------------------------------------
ykris0001_21250  ------------------------------------------------------------
yruck0001_4630   ------------------------------------------------------------
yrohd0001_40320  ------------------------------------------------------------
yrohd0001_40080  ------------------------------------------------------------
yrohd0001_32210  LSTYSSSLLN--NSNIVATRASLTTAGALINSGEILTLGSLNTDSNTLFNTGTIISSQAT
yfred0001_45640  LSTDSNSLLN--SGTIIATQADLKAVGALINSGDILTSGLLNTDVNTLFNTGTIISAQAT
yfred0001_38220  ------------------------------------------------------------
yrohd0001_38410  LSTDST----------------------------------------TLFNSGSIIGAEAT
yfred0001_33220  LSTDST----------------------------------------TLFNSGSIIGAEAT
ymoll0001_36970  LTTDSSSLLN--RGQMIAAQASLTSAGALTNRGEILTQGRLDTDSNTLFNTGTIISAEAT
yrohd0001_39710  ------------------------------------------------------------
yrohd0001_18880  ------------------------------------------------------------
yinte0001_5480   LSTHSVSLLN--NGNISATQASLTATGALTNRGEILTRGRLDTDSNTLFNTGSLISAEAT
yfred0001_33200  ------------------------------------------------------------
yrohd0001_18890  ------------------------------------------------------------
yfred0001_32960  ------------------------------------------------------------
yberc0001_40160  ------------------------------------------------------------
ypseu0001X_3843  ------------------------------------------------------------
ypseu0001X_3837  ------------------------------------------------------------
ypest0001X_8140  ------------------------------------------------------------
ypseu0001X_3848  LSTRSNSLLN--NGNIRAVQADLRASGALTNRGEILTRGGLSTDANTLFNSGTLIGATAT
ypest0001X_8130  ------------------------------------------------------------
ypest0001X_8080  LSTRSNSLLN--NGNIRAVQADLRASGALTNRGEILTRGGLSTDANTLFNSGTLIGATAT
yinte0001_41760  MGVQAQQIENQQDAKLFSAGDLTVVGGTFSLFGQLVALGNLSVTLNDALTQHGTLAAGKV
ykris0001_32060  MSVQAQQIDNQQDAKLFSAGDLTVVSGGFSLFGQLVALGNLSLTLNDALTQHGTLAAGKM
ypseu0001X_3841  ------------------------------------------------------------
yrohd0001_38400  ------------------------------------------------------------
yfred0001_40710  ------------------------------------------------------------
yente0001X_8000  ------------------------------------------------------------
yruck0001_35040  ------------------------------------------------------------
                                                                             


                       1750      1760      1770      1780      1790      1800
                 =========+=========+=========+=========+=========+=========+
yberc0001_8630   LKARERITNSGPNALIGATDENGTLALLAPVIENSDTATNTDSAPSTTILGMGKVILAGG
ykris0001_26670  ------------------------------------------------------------
ykris0001_41280  ------------------------------------------------------------
yruck0001_910    ------------------------------------------------------------
yaldo0001_37900  IKLREEIKNSGNELLIKAQENSDKLALSKISMTAIGEFKPSQ------------------
yberc0001_34750  TAASESSSVSTQGDIGRNRGVEDSASAMNMSVTDQATYNSAIGKSTETGEAKGTEQAFGS
yaldo0001_38900  ------------------------------------------------------------
yaldo0001_41000  ------------------------------------------------------------
ypseu0001X_3846  ------------------------------------------------------------
ymoll0001_35980  ------------------------------------------------------------
ypest0001X_8100  ------------------------------------------------------------
yfred0001_38200  ------------------------------------------------------------
ypest0001X_2754  ------------------------------------------------------------
yfred0001_34120  ------------------------------------------------------------
ypseu0001X_2842  ------------------------------------------------------------
ykris0001_7890   ------------------------------------------------------------
yruck0001_4640   ------------------------------------------------------------
ymoll0001_2720   ---------AGPMVIRHPQESLGDLPETHWVVND--------------------------
yrohd0001_40310  ------------------------------------------------------------
yruck0001_34980  ------------------------------------------------------------
ypest0001X_2756  ------------------------------------------------------------
yfred0001_45620  ------------------------------------------------------------
yrohd0001_18860  ------------------------------------------------------------
yfred0001_33010  ------------------------------------------------------------
yfred0001_33210  ------------------------------------------------------------
yrohd0001_40300  ------------------------------------------------------------
yruck0001_4620   ------------------------------------------------------------
ypest0001X_8110  ------------------------------------------------------------
yberc0001_40130  ------------------------------------------------------------
yberc0001_40750  ------------------------------------------------------------
yruck0001_35050  ------------------------------------------------------------
yruck0001_13030  ---------------------NSNIEAGKALFFGGAINERREKGKKVTTGIFDIPFSSNQ
yinte0001_17980  LLARENIIDDGTQYHAKGLNKEGGLFSLDAGHHFSHTAINSSQSEVDGEKGEGAIRVS--
yaldo0001_6040   ------------------------------------------------------------
yfred0001_33270  ---------------------------------------------------XGKVILAGA
yrohd0001_18870  ------------------------------------------------------------
yruck0001_4610   ------------------------------------------------------------
ypest0001X_2758  ------------------------------------------------------------
ypseu0001X_2843  ------------------------------------------------------------
ypseu0001X_2846  LNARERITNSGPDALIGATDENGTLALLAPVIENSDTVTHTDTAPTTTILGMGTVILAGG
ypest0001X_2761  LNARERITNSGPDALIGATDENGTLALLAPVIENSDTVTHTDTAPTTTILGMGTVILAGG
yrohd0001_32190  ------------------------------------------------------------
ymoll0001_21160  ------------------------------------------------------------
yrohd0001_32220  ------------------------------------------------------------
yfred0001_34080  ------------------------------------------------------------
yruck0001_4650   LKARERMTNSGTKALIGATDEKGTLALLAPVIENSDTVTNTDTSPSTTILGMGKVVLAGG
yaldo0001_38920  ------------------------------------------------------------
yberc0001_20820  ------------------------------------------------------------
yaldo0001_3710   LKARERITNSGPKALIGATDENGTLTLLAPVIENSDTVTRTDTAPSTTLLGMGKIILAGA
ypest0001X_8090  ------------------------------------------------------------
yfred0001_34100  ------------------------------------------------------------
yruck0001_25350  SQGKLTIQTAGNLNVTASKEQQKIDEQNSALNINAYAKEESDKQYRA---GLRIEHTSDS
yfred0001_38190  ------------------------------------------------------------
yfred0001_34090  ------------------------------------------------------------
yaldo0001_30990  ------------------------------------------------------------
yberc0001_36600  ------------------------------------------------------------
yrohd0001_40100  ------------------------------------------------------------
yrohd0001_40280  ------------------------------------------------------------
yruck0001_4660   ------------------------------------------------------------
ypseu0001X_3844  ------------------------------------------------------------
ykris0001_41250  ------------------------------------------------------------
ykris0001_21250  ------------------------------------------------------------
yruck0001_4630   ------------------------------------------------------------
yrohd0001_40320  ------------------------------------------------------------
yrohd0001_40080  ------------------------------------------------------------
yrohd0001_32210  LKAQERITNSGPSALIGATDENGTLALLAPVIENSDTVTDTDTAPTTTILGVGNIILAGA
yfred0001_45640  LKARERITNSGPNALIGATDENGTLALLAPVXLIN-------------------------
yfred0001_38220  ------------------------------------------------------------
yrohd0001_38410  LKARDRITNSGPKALIGATDENGTLTLLAPVIENSDTVTHTDSAPTTTLLGMGKVILAGG
yfred0001_33220  LKARDRITNSGPKALIGATDENGTLTLLAPVLENSDTVTNTDSAPTTTILGMGKVILAGR
ymoll0001_36970  LKARERITNSGPNALIGATDENGTLALLAPVIENSDTVTNTDTAPTTTILGMGKMILAGA
yrohd0001_39710  ------------------------------------------------------------
yrohd0001_18880  ------------------------------------------------------------
yinte0001_5480   LKARERITNSGPNALMGATDENGTLALLAPVIENSDLVTSTDTAPTTTLLGMGKVILAGG
yfred0001_33200  ---------------------------------------------------XGKVILAGG
yrohd0001_18890  ------------------------------------------------------------
yfred0001_32960  ------------------------------------------------------------
yberc0001_40160  ------------------------------------------------------------
ypseu0001X_3843  ------------------------------------------------------------
ypseu0001X_3837  ------------------------------------------------------------
ypest0001X_8140  ------------------------------------------------------------
ypseu0001X_3848  LNARERITNSGPNALIGATDKNGTLALLAPVIENSDTVTRTDTAPTTTLLGMGKVILAGG
ypest0001X_8130  ------------------------------------------------------------
ypest0001X_8080  LNARERITNSGPNALIGATDKNGTLALLAPVIENSDTVTRTDTAPTTTLLGMGKVILAGG
yinte0001_41760  LN----LSSNGDITLAGTTQGQSLAIHSLGQFTNSGTLRGGNGDVRINAAGITQNDTASL
ykris0001_32060  LN----LSSHGDITLTGTTQGQSLSIHSLGQFTHSGTLRGGNGDVRIEAMGITQNDTASL
ypseu0001X_3841  ------------------------------------------------------------
yrohd0001_38400  ------------------------------------------------------------
yfred0001_40710  ------------------------------------------------------------
yente0001X_8000  ------------------------------------------------------------
yruck0001_35040  ------------------------------------------------------------
                                                                             


                       1810      1820      1830      1840      1850      1860
                 =========+=========+=========+=========+=========+=========+
yberc0001_8630   QDNGGNYQTAAQVLNISGLIESGKDLLVYATTLTNRRHILTANSNFVAAGTESGTAYWTA
ykris0001_26670  ------------------------------------------------------------
ykris0001_41280  ------------------------------------------------------------
yruck0001_910    ------------------------------------------------------------
yaldo0001_37900  ------------------------------------------------------------
yberc0001_34750  LDNIGNKTAFNTAVSKATQAGESKGVM---------------------------------
yaldo0001_38900  ------------------------------------------------------------
yaldo0001_41000  ------------------------------------------------------------
ypseu0001X_3846  ------------------------------------------------------------
ymoll0001_35980  ------------------------------------------------------------
ypest0001X_8100  ------------------------------------------------------------
yfred0001_38200  ------------------------------------------------------------
ypest0001X_2754  ------------------------------------------------------------
yfred0001_34120  ------------------------------------------------------------
ypseu0001X_2842  ------------------------------------------------------------
ykris0001_7890   ------------------------------------------------------------
yruck0001_4640   ------------------------------------------------------------
ymoll0001_2720   ------------------------------------------------------------
yrohd0001_40310  ------------------------------------------------------------
yruck0001_34980  ------------------------------------------------------------
ypest0001X_2756  ------------------------------------------------------------
yfred0001_45620  ------------------------------------------------------------
yrohd0001_18860  ------------------------------------------------------------
yfred0001_33010  ------------------------------------------------------------
yfred0001_33210  ------------------------------------------------------------
yrohd0001_40300  ------------------------------------------------------------
yruck0001_4620   ------------------------------------------------------------
ypest0001X_8110  ------------------------------------------------------------
yberc0001_40130  ------------------------------------------------------------
yberc0001_40750  ------------------------------------------------------------
yruck0001_35050  ------------------------------------------------------------
yruck0001_13030  KDNSYEAFVDSQITTGGDLVARGDTVQIEGSIFDINNHLSIHSDNDITVIAAREQQKKDE
yinte0001_17980  ------------------------------------------------------------
yaldo0001_6040   ------------------------------------------------------------
yfred0001_33270  LDNSGNYLTAAQVLNISGLIESGKDMLIYANTLTNSRHILTANSDFVVADTVTGSAYWTA
yrohd0001_18870  ------------------------------------------------------------
yruck0001_4610   ------------------------------------------------------------
ypest0001X_2758  ------------------------------------------------------------
ypseu0001X_2843  ------------------------------------------------------------
ypseu0001X_2846  QASDGHYASAAQVLNLSGLIESGKDMLIYATTLTNSRHILTANTDFIVADTVTGTAVWTA
ypest0001X_2761  HARDGHYASAAQVLNLSGLIESGKDMLIYATTLTNSRHILTANTDFIVADTVTGTAVWTA
yrohd0001_32190  ------------------------------------------------------------
ymoll0001_21160  ------------------------------------------------------------
yrohd0001_32220  ------------------------------------------------------------
yfred0001_34080  ------------------------------------------------------------
yruck0001_4650   QDNSGNYQTAAQILNLSGLIESGKDLLIYANTLTNRRHVLTANTQFVAGDTVNGTAYWTP
yaldo0001_38920  ------------------------------------------------------------
yberc0001_20820  ------------------------------------------------------------
yaldo0001_3710   LDNSGNYLAAAQVLNISGLIESGKDMLIYATTLTNRRHILTANSDFVVANTVNGTAYWTA
ypest0001X_8090  ------------------------------------------------------------
yfred0001_34100  ------------------------------------------------------------
yruck0001_25350  EKTSRNENTGAKLSGGSVDINAAKDVTFTGSKLETTNGDAVVKGDNVAFLAAEDKTVSEK
yfred0001_38190  ------------------------------------------------------------
yfred0001_34090  ------------------------------------------------------------
yaldo0001_30990  ------------------------------------------------------------
yberc0001_36600  ------------------------------------------------------------
yrohd0001_40100  ------------------------------------------------------------
yrohd0001_40280  ------------------------------------------------------------
yruck0001_4660   ------------------------------------------------------------
ypseu0001X_3844  ------------------------------------------------------------
ykris0001_41250  ------------------------------------------------------------
ykris0001_21250  ------------------------------------------------------------
yruck0001_4630   ------------------------------------------------------------
yrohd0001_40320  ------------------------------------------------------------
yrohd0001_40080  ------------------------------------------------------------
yrohd0001_32210  LDNSGNYLAAAQVFNISGLIESGKDMLIYANTLTNSRHILSANSDFVIADTVTGSAVWTE
yfred0001_45640  ------------------------------------------------------------
yfred0001_38220  ------------------------------------------------------------
yrohd0001_38410  QDNGGNYQPAAQILNISGLIESGKDLLVYASKLTNRRHILTANSNFVAAGTVSGTGYWTA
yfred0001_33220  QDNGGNYQTAAQILNISGLIESGKDLLVYASKLTNSRHILTANSNFVAAGTVSGTGYWTA
ymoll0001_36970  LDSSGNYLAAAQVLNISGLIESGKDMVIYATTLTNRRHILTANSDFVVADTVNGSAYWTA
yrohd0001_39710  ------------------------------------------------------------
yrohd0001_18880  ------------------------------------------------------------
yinte0001_5480   QDKGGNYLTAAQILNISGLIESGKDMLIYATTLTNRRHILTANTQFVTGNTVSGSAYWSP
yfred0001_33200  QDSGGNYQQAAQVLNISGLIESGQDMLIYATTLTNSRHILTANSDFVMADTVNGSAYWTV
yrohd0001_18890  ------------------------------------------------------------
yfred0001_32960  ------------------------------------------------------------
yberc0001_40160  ------------------------------------------------------------
ypseu0001X_3843  ------------------------------------------------------------
ypseu0001X_3837  ------------------------------------------------------------
ypest0001X_8140  ------------------------------------------------------------
ypseu0001X_3848  QDNSGNYSSAAQVLNLSGLIESGNDLLVYAKTLTNRRQILTATTDFIVGDTVTGAAYWTA
ypest0001X_8130  ------------------------------------------------------------
ypest0001X_8080  QDNSGNYSSAAQVLNLSGLIESGNDLLVYAKTLTNRRQILTATTDFIVGDTETGAAYWTA
yinte0001_41760  QAGGRIQLLSSSTISNNGFIGTAGDLLLNAASQLFNSGMLYSGGNMQLLADRITNHYGDI
ykris0001_32060  QAGGRIQLLSSSTISNNGFIGTAGDLLLNAASQLFNSGMLYSGGNMQLLADQITNHYGDI
ypseu0001X_3841  ------------------------------------------------------------
yrohd0001_38400  ------------------------------------------------------------
yfred0001_40710  ------------------------------------------------------------
yente0001X_8000  ------------------------------------------------------------
yruck0001_35040  ------------------------------------------------------------
                                                                             


                       1870      1880      1890      1900      1910      1920
                 =========+=========+=========+=========+=========+=========+
yberc0001_8630   ANPDIPGGRYAEPPHGGSMNSDYIGT----------------------------------
ykris0001_26670  ------------------------------------------------------------
ykris0001_41280  ------------------------------------------------------------
yruck0001_910    ------------------------------------------------------------
yaldo0001_37900  ------------------------------------------------------------
yberc0001_34750  ------------------------------------------------------------
yaldo0001_38900  ------------------------------------------------------------
yaldo0001_41000  ------------------------------------------------------------
ypseu0001X_3846  ------------------------------------------------------------
ymoll0001_35980  ------------------------------------------------------------
ypest0001X_8100  ------------------------------------------------------------
yfred0001_38200  ------------------------------------------------------------
ypest0001X_2754  ------------------------------------------------------------
yfred0001_34120  ------------------------------------------------------------
ypseu0001X_2842  ------------------------------------------------------------
ykris0001_7890   ------------------------------------------------------------
yruck0001_4640   ------------------------------------------------------------
ymoll0001_2720   ------------------------------------------------------------
yrohd0001_40310  ------------------------------------------------------------
yruck0001_34980  ------------------------------------------------------------
ypest0001X_2756  ------------------------------------------------------------
yfred0001_45620  ------------------------------------------------------------
yrohd0001_18860  ------------------------------------------------------------
yfred0001_33010  ------------------------------------------------------------
yfred0001_33210  ------------------------------------------------------------
yrohd0001_40300  ------------------------------------------------------------
yruck0001_4620   ------------------------------------------------------------
ypest0001X_8110  ------------------------------------------------------------
yberc0001_40130  ------------------------------------------------------------
yberc0001_40750  ------------------------------------------------------------
yruck0001_35050  ------------------------------------------------------------
yruck0001_13030  QSTQLSMGFYSEESDKNQYNAGF-------------------------------------
yinte0001_17980  ------------------------------------------------------------
yaldo0001_6040   ------------------------------------------------------------
yfred0001_33270  ENPDIPGGRYKEPEHGGANNSDYIGT----------------------------------
yrohd0001_18870  ------------------------------------------------------------
yruck0001_4610   ------------------------------------------------------------
ypest0001X_2758  ------------------------------------------------------------
ypseu0001X_2843  ------------------------------------------------------------
ypseu0001X_2846  ENPDIPGGRYAEPPNGGADNSDYIGT----------------------------------
ypest0001X_2761  ENPDIPGGRYAEPPDGGADNSDYIGT----------------------------------
yrohd0001_32190  ------------------------------------------------------------
ymoll0001_21160  ------------------------------------------------------------
yrohd0001_32220  ------------------------------------------------------------
yfred0001_34080  ------------------------------------------------------------
yruck0001_4650   QNPDIPGGRYIEPPHGGKNNSSYINT----------------------------------
yaldo0001_38920  ------------------------------------------------------------
yberc0001_20820  ------------------------------------------------------------
yaldo0001_3710   DKPDIPGGRYHEPDHGGANNSDYIGT----------------------------------
ypest0001X_8090  ------------------------------------------------------------
yfred0001_34100  ------------------------------------------------------------
yruck0001_25350  NSTKTGGGFYYTGGIDKAGNGYEV------------------------------------
yfred0001_38190  ------------------------------------------------------------
yfred0001_34090  ------------------------------------------------------------
yaldo0001_30990  ------------------------------------------------------------
yberc0001_36600  ------------------------------------------------------------
yrohd0001_40100  ------------------------------------------------------------
yrohd0001_40280  ------------------------------------------------------------
yruck0001_4660   ------------------------------------------------------------
ypseu0001X_3844  ------------------------------------------------------------
ykris0001_41250  ------------------------------------------------------------
ykris0001_21250  ------------------------------------------------------------
yruck0001_4630   ------------------------------------------------------------
yrohd0001_40320  ------------------------------------------------------------
yrohd0001_40080  ------------------------------------------------------------
yrohd0001_32210  ENPDIPGGRYDEPPHGGSMNSDYVNT----------------------------------
yfred0001_45640  ------------------------------------------------------------
yfred0001_38220  ------------------------------------------------------------
yrohd0001_38410  ENPDIPGGRYAEPPHGGSMNSDYTGT----------------------------------
yfred0001_33220  ENPDIPGGRYAEPPHGGSMNSDYIGT----------------------------------
ymoll0001_36970  ENPDIPGGRYSEPPHGGSMNSDYIGT----------------------------------
yrohd0001_39710  ------------------------------------------------------------
yrohd0001_18880  ------------------------------------------------------------
yinte0001_5480   TNPDVPGGRYAEPPHGGSMNSDYIGT----------------------------------
yfred0001_33200  ENPDIPGGRYSEPPHEGADNSDYIGT----------------------------------
yrohd0001_18890  ------------------------------------------------------------
yfred0001_32960  ------------------------------------------------------------
yberc0001_40160  ------------------------------------------------------------
ypseu0001X_3843  ------------------------------------------------------------
ypseu0001X_3837  ------------------------------------------------------------
ypest0001X_8140  ------------------------------------------------------------
ypseu0001X_3848  ENPDIPGGRYTQPPAGGPMNSDYIGT----------------------------------
ypest0001X_8130  ------------------------------------------------------------
ypest0001X_8080  ENPDIPGGRYTQPPAGGPMNSDYIGT----------------------------------
yinte0001_41760  LADNSLWMQKDTTGNANSEVINTSGTIETGHGDITINTAHLLNQRDGLSVTRTDKDLTNE
ykris0001_32060  LADNSLWMQKDTTGSANSEVINTSGTIETGHGDITINTNHLLNQRDGLSVTRTDKDLTNE
ypseu0001X_3841  ------------------------------------------------------------
yrohd0001_38400  ------------------------------------------------------------
yfred0001_40710  ------------------------------------------------------------
yente0001X_8000  ------------------------------------------------------------
yruck0001_35040  ------------------------------------------------------------
                                                                             


                       1930      1940      1950      1960      1970      1980
                 =========+=========+=========+=========+=========+=========+
yberc0001_8630   ------------------------------------------------------NYTSTI
ykris0001_26670  ------------------------------------------------------------
ykris0001_41280  ------------------------------------------------------------
yruck0001_910    ------------------------------------------------------------
yaldo0001_37900  ------------------------------------------------------------
yberc0001_34750  ------------------------------------------------------DAAGNN
yaldo0001_38900  ------------------------------------------------------------
yaldo0001_41000  ------------------------------------------------------------
ypseu0001X_3846  ------------------------------------------------------------
ymoll0001_35980  ------------------------------------------------------------
ypest0001X_8100  ------------------------------------------------------------
yfred0001_38200  ------------------------------------------------------------
ypest0001X_2754  ------------------------------------------------------------
yfred0001_34120  ------------------------------------------------------------
ypseu0001X_2842  ------------------------------------------------------------
ykris0001_7890   ------------------------------------------------------------
yruck0001_4640   ------------------------------------------------------------
ymoll0001_2720   ------------------------------------------------------------
yrohd0001_40310  ------------------------------------------------------------
yruck0001_34980  ------------------------------------------------------------
ypest0001X_2756  ------------------------------------------------------------
yfred0001_45620  ------------------------------------------------------------
yrohd0001_18860  ------------------------------------------------------------
yfred0001_33010  ------------------------------------------------------------
yfred0001_33210  ------------------------------------------------------------
yrohd0001_40300  ------------------------------------------------------------
yruck0001_4620   ------------------------------------------------------------
ypest0001X_8110  ------------------------------------------------------------
yberc0001_40130  ------------------------------------------------------------
yberc0001_40750  ------------------------------------------------------------
yruck0001_35050  ------------------------------------------------------------
yruck0001_13030  ------------------------------------------------------MIKYIN
yinte0001_17980  ------------------------------------------------------------
yaldo0001_6040   ------------------------------------------------------------
yfred0001_33270  ------------------------------------------------------DYTSTV
yrohd0001_18870  ------------------------------------------------------------
yruck0001_4610   ------------------------------------------------------------
ypest0001X_2758  ------------------------------------------------------------
ypseu0001X_2843  ------------------------------------------------------------
ypseu0001X_2846  ------------------------------------------------------EYTSVI
ypest0001X_2761  ------------------------------------------------------EYTSVI
yrohd0001_32190  ------------------------------------------------------------
ymoll0001_21160  ------------------------------------------------------------
yrohd0001_32220  ------------------------------------------------------------
yfred0001_34080  ------------------------------------------------------------
yruck0001_4650   ------------------------------------------------------SYTSTT
yaldo0001_38920  ------------------------------------------------------------
yberc0001_20820  ------------------------------------------------------------
yaldo0001_3710   ------------------------------------------------------SYTSTL
ypest0001X_8090  ------------------------------------------------------------
yfred0001_34100  ------------------------------------------------------------
yruck0001_25350  ------------------------------------------------------SHESST
yfred0001_38190  ------------------------------------------------------------
yfred0001_34090  ------------------------------------------------------------
yaldo0001_30990  ------------------------------------------------------------
yberc0001_36600  ------------------------------------------------------------
yrohd0001_40100  ------------------------------------------------------------
yrohd0001_40280  ------------------------------------------------------------
yruck0001_4660   ------------------------------------------------------------
ypseu0001X_3844  ------------------------------------------------------------
ykris0001_41250  ------------------------------------------------------------
ykris0001_21250  ------------------------------------------------------------
yruck0001_4630   ------------------------------------------------------------
yrohd0001_40320  ------------------------------------------------------------
yrohd0001_40080  ------------------------------------------------------------
yrohd0001_32210  ------------------------------------------------------DYTLTF
yfred0001_45640  ------------------------------------------------------------
yfred0001_38220  ------------------------------------------------------------
yrohd0001_38410  ------------------------------------------------------DYTSTY
yfred0001_33220  ------------------------------------------------------NYTSTT
ymoll0001_36970  ------------------------------------------------------DYTSTI
yrohd0001_39710  ------------------------------------------------------------
yrohd0001_18880  ------------------------------------------------------------
yinte0001_5480   ------------------------------------------------------NYTSTY
yfred0001_33200  ------------------------------------------------------DYTSTI
yrohd0001_18890  ------------------------------------------------------------
yfred0001_32960  ------------------------------------------------------------
yberc0001_40160  ------------------------------------------------------------
ypseu0001X_3843  ------------------------------------------------------------
ypseu0001X_3837  ------------------------------------------------------------
ypest0001X_8140  ------------------------------------------------------------
ypseu0001X_3848  ------------------------------------------------------NYTSTV
ypest0001X_8130  ------------------------------------------------------------
ypest0001X_8080  ------------------------------------------------------NYTSTV
yinte0001_41760  YPWLNGALVKVPLSFFEAGEIGYYTIMITRQEAGDDARQVSHTDTYAAPFEKTRELALSV
ykris0001_32060  YPWLNGAIVKVPLSFFEEGEVGYYTIKITQQEAGDDANQVSHTETYAAPFEKTRELALSV
ypseu0001X_3841  ------------------------------------------------------------
yrohd0001_38400  ------------------------------------------------------------
yfred0001_40710  ------------------------------------------------------------
yente0001X_8000  ------------------------------------------------------------
yruck0001_35040  ------------------------------------------------------------
                                                                             


                       1990      2000      2010      2020      2030      2040
                 =========+=========+=========+=========+=========+=========+
yberc0001_8630   AKNSIDKISPEAQLLAGGSLTPHVGTLENYWSKISAQGEIDLSSVTLNQDGWGSAQRLIE
ykris0001_26670  ------------------------------------------------------------
ykris0001_41280  ------------------------------------------------------------
yruck0001_910    ------------------------------------------------------------
yaldo0001_37900  ------------------------------------------------------------
yberc0001_34750  LSNVESRQSEISSVRSAENIGEARGTRDAFGSLSGIESSTQFNTGESKAQGLVDNQRQRD
yaldo0001_38900  ----------------GADFVPVVGDIKSF------------------------------
yaldo0001_41000  ------------------------------------------------------------
ypseu0001X_3846  ------------------------------------------------------------
ymoll0001_35980  ------------------------------------------------------------
ypest0001X_8100  ------------------------------------------------------------
yfred0001_38200  ------------------------------------------------------------
ypest0001X_2754  ------------------------------------------------------------
yfred0001_34120  ------------------------------------------------------------
ypseu0001X_2842  ------------------------------------------------------------
ykris0001_7890   ------------------------------------------------------------
yruck0001_4640   ------------------------------------------------------------
ymoll0001_2720   ------------------------------------------------------------
yrohd0001_40310  ------------------------------------------------------------
yruck0001_34980  ------------------------------------------------------------
ypest0001X_2756  ------------------------------------------------------------
yfred0001_45620  ------------------------------------------------------------
yrohd0001_18860  ------------------------------------------------------------
yfred0001_33010  ------------------------------------------------------------
yfred0001_33210  ------------------------------------------------------------
yrohd0001_40300  ------------------------------------------------------------
yruck0001_4620   ------------------------------------------------------------
ypest0001X_8110  ------------------------------------------------------------
yberc0001_40130  ------------------------------------------------------------
yberc0001_40750  ------------------------------------------------------------
yruck0001_35050  ------------------------------------------------------------
yruck0001_13030  ESEKSTHNNSKTSTLKAGDIDISAGGDLLYYGTAIETTAGNLTINADKNVGFFAARNNMT
yinte0001_17980  ------------------------------------------------------------
yaldo0001_6040   ------------------------------------------------------------
yfred0001_33270  AYNSIDNISPEAQLLAGGNLTPQVGTLENFWSKISAQGEINLTGVTLQQDGWGSAQRLIE
yrohd0001_18870  ------------------------------------------------------------
yruck0001_4610   ------------------------------------------------------------
ypest0001X_2758  ------------------------------------------------------------
ypseu0001X_2843  ------------------------------------------------------------
ypseu0001X_2846  AYNGIDQISPEAQLLAGGNLTPQVGTLENFWSKVSAQGEIDLTGVTLQQDGWGDQQRLME
ypest0001X_2761  AYNGIDQISPEAQLLAGGNLTPQVGTLENFWSKVSAQGEIDLTGVTLQQDGWGDQQRLME
yrohd0001_32190  ------------------------------------------------------------
ymoll0001_21160  ------------------------------------------------------------
yrohd0001_32220  ------------------------------------------------------------
yfred0001_34080  ------------------------------------------------------------
yruck0001_4650   SYNSIDKISPEAQLLAGGNLTPHVGTLENYWSKVSAQGEINLTGVALQQDGWGSQQRLIE
yaldo0001_38920  ------------------------------------------------------------
yberc0001_20820  ------------------------------------------------------------
yaldo0001_3710   AYNSIDQISPEAQLLAGGNLTPQVGTLENFWSKVSAQGEIDLTGVTLNQDGWGSAQRLIE
ypest0001X_8090  ------------------------------------------------------------
yfred0001_34100  ------------------------------------------------------------
yruck0001_25350  THSDSSKAVVSSSQVAGNLSIEAKGSLTNQGTQHQVDGKLKQDAANVNNLTAKNSENSQT
yfred0001_38190  ------------------------------------------------------------
yfred0001_34090  ------------------------------------------------------------
yaldo0001_30990  ------------------------------------------------------------
yberc0001_36600  ------------------------------------------------------------
yrohd0001_40100  ------------------------------------------------------------
yrohd0001_40280  ------------------------------------------------------------
yruck0001_4660   ------------------------------------------------------------
ypseu0001X_3844  ------------------------------------------------------------
ykris0001_41250  ------------------------------------------------------------
ykris0001_21250  ------------------------------------------------------------
yruck0001_4630   ------------------------------------------------------------
yrohd0001_40320  ------------------------------------------------------------
yrohd0001_40080  ------------------------------------------------------------
yrohd0001_32210  AYNGIDEISPEAQLLAGGNLTPQVGTLENFWSKISAQGEIDLSGVTLQQDGWGSAQRLIE
yfred0001_45640  ------------------------------------------------------------
yfred0001_38220  ------------------------------------------------------------
yrohd0001_38410  AYNGIDQISPEAQLLAGGNLTPQVGALENFWSKVSAQGEINLSGVTLNQDGWGNAQRLIE
yfred0001_33220  AKNSIDKISPEAQLLAGGNLMPHVSTLENFWSKVSAQGEINLSGVTLNQDGWGNAQRLIE
ymoll0001_36970  AYNGIDQISPEAQLLAGGDLTPQVGTLENFWSKVSAQGEIDLSGVTLNQDGWGSAQRLIE
yrohd0001_39710  ------------------------------------------------------------
yrohd0001_18880  ------------------------------------------------------------
yinte0001_5480   AYNGIDHISPEAHLLAGGNLTPQVGTLENFWSKVSAQGEIDLSGVTLNQDGWGNAQRLIE
yfred0001_33200  AYNSIDNISPEAQLLAGRNLTPQVGTLENFWSKISAQGEINLTGVTLQQDGWGSAQRLME
yrohd0001_18890  ------------------------------------------------------------
yfred0001_32960  ------------------------------------------------------------
yberc0001_40160  ------------------------------------------------------------
ypseu0001X_3843  ------------------------------------------------------------
ypseu0001X_3837  ------------------------------------------------------------
ypest0001X_8140  ------------------------------------------------------------
ypseu0001X_3848  AYNRIDQISSEAQLLAGGNLTLQVGTLENNWSKVSAQGVIDLTGVTLQQDDWGSQQRLVE
ypest0001X_8130  ------------------------------------------------------------
ypest0001X_8080  AYNRIDQISPEAQLLAGGNLTLQVGTLENNWSKVSAQGVIDLTGVTLQQDDWGSQQRLVE
yinte0001_41760  STLSVTSKGAAGRISSGKDLIVTAQRFDNLASDILSNGDISLTGNTLNNQSWLAGTETRY
ykris0001_32060  STLSVTSKGAAGRISSGKDLVVTAQTLDNLASDILSNGDISLTGNTLNNQSWLAGTETRY
ypseu0001X_3841  ------------------------------------------------------------
yrohd0001_38400  ------------------------------------------------------------
yfred0001_40710  ------------------------------------------------------------
yente0001X_8000  ------------------------------------------------------------
yruck0001_35040  ------------------------------------------------------------
                                                                             


                       2050      2060      2070      2080      2090      2100
                 =========+=========+=========+=========+=========+=========+
yberc0001_8630   RTTSTGEWRYRTYKGNLWGTGW--------------------------------------
ykris0001_26670  ------------------------------------------------------------
ykris0001_41280  ------------------------------------------------------------
yruck0001_910    ------------------------------------------------------------
yaldo0001_37900  ------------------------------------------------------------
yberc0001_34750  QVETI-------------------------------------------------------
yaldo0001_38900  ------------------------------------------------------------
yaldo0001_41000  ------------------------------------------------------------
ypseu0001X_3846  ------------------------------------------------------------
ymoll0001_35980  ------------------------------------------------------------
ypest0001X_8100  ------------------------------------------------------------
yfred0001_38200  ------------------------------------------------------------
ypest0001X_2754  ------------------------------------------------------------
yfred0001_34120  ------------------------------------------------------------
ypseu0001X_2842  ------------------------------------------------------------
ykris0001_7890   ------------------------------------------------------------
yruck0001_4640   ------------------------------------------------------------
ymoll0001_2720   ------------------------------------------------------------
yrohd0001_40310  ------------------------------------------------------------
yruck0001_34980  ------------------------------------------------------------
ypest0001X_2756  ------------------------------------------------------------
yfred0001_45620  ------------------------------------------------------------
yrohd0001_18860  ------------------------------------------------------------
yfred0001_33010  ------------------------------------------------------------
yfred0001_33210  ------------------------------------------------------------
yrohd0001_40300  ------------------------------------------------------------
yruck0001_4620   ------------------------------------------------------------
ypest0001X_8110  ------------------------------------------------------------
yberc0001_40130  ------------------------------------------------------------
yberc0001_40750  ------------------------------------------------------------
yruck0001_35050  ------------------------------------------------------------
yruck0001_13030  ------------------------------------------------------------
yinte0001_17980  ------------------------------------------------------------
yaldo0001_6040   ------------------------------------------------------------
yfred0001_33270  RTTSSGTWNYRNYKGGLWSDNW--------------------------------------
yrohd0001_18870  ------------------------------------------------------------
yruck0001_4610   ------------------------------------------------------------
ypest0001X_2758  ------------------------------------------------------------
ypseu0001X_2843  ------------------------------------------------------------
ypseu0001X_2846  QTTSSGVWRYRTYKGGLWAWAW--------------------------------------
ypest0001X_2761  QTTSSGVWRYRTYKGGLWTREW--------------------------------------
yrohd0001_32190  ------------------------------------------------------------
ymoll0001_21160  ------------------------------------------------------------
yrohd0001_32220  ------------------------------------------------------------
yfred0001_34080  ------------------------------------------------------------
yruck0001_4650   KVISKGYYHYRTYKGRLWTNGW--------------------------------------
yaldo0001_38920  ------------------------------------------------------------
yberc0001_20820  ------------------------------------------------------------
yaldo0001_3710   RTTSTGLWNYRNYKGGLWNKGW--------------------------------------
ypest0001X_8090  ------------------------------------------------------------
yfred0001_34100  ------------------------------------------------------------
yruck0001_25350  ETLKVG------------------------------------------------------
yfred0001_38190  ------------------------------------------------------------
yfred0001_34090  ------------------------------------------------------------
yaldo0001_30990  ------------------------------------------------------------
yberc0001_36600  ------------------------------------------------------------
yrohd0001_40100  ------------------------------------------------------------
yrohd0001_40280  ------------------------------------------------------------
yruck0001_4660   ------------------------------------------------------------
ypseu0001X_3844  ------------------------------------------------------------
ykris0001_41250  ------------------------------------------------------------
ykris0001_21250  ------------------------------------------------------------
yruck0001_4630   ------------------------------------------------------------
yrohd0001_40320  ------------------------------------------------------------
yrohd0001_40080  ------------------------------------------------------------
yrohd0001_32210  RTTSSGQWNYRNYKGDLWFWNW--------------------------------------
yfred0001_45640  ------------------------------------------------------------
yfred0001_38220  ------------------------------------------------------------
yrohd0001_38410  RTTSSGEWRYRTYKGNLWGTGW--------------------------------------
yfred0001_33220  RTTSSGEWRYRTYKGNLWGTGW--------------------------------------
ymoll0001_36970  KTTSTGEWRYRTYKGDLWSIGW--------------------------------------
yrohd0001_39710  ------------------------------------------------------------
yrohd0001_18880  ------------------------------------------------------------
yinte0001_5480   RTTSSGEWRYRTYKGNLWGTAW--------------------------------------
yfred0001_33200  QTTSSGQWNYRNYKGGLWHRDW--------------------------------------
yrohd0001_18890  ------------------------------------------------------------
yfred0001_32960  ------------------------------------------------------------
yberc0001_40160  ------------------------------------------------------------
ypseu0001X_3843  ------------------------------------------------------------
ypseu0001X_3837  ------------------------------------------------------------
ypest0001X_8140  ------------------------------------------------------------
ypseu0001X_3848  QTTSSGEYRYRTYKGKLWGIAW--------------------------------------
ypest0001X_8130  --------VRRTYKGKLWGIAW--------------------------------------
ypest0001X_8080  QTTSSGEYRYRTYKGKLWGIAW--------------------------------------
yinte0001_41760  QTYRTGELPQSRYWYEVSKLRPLNIYALGQIKDQSVTYTADGDIRTE----------RSE
ykris0001_32060  QTYRTGELPESRYWYEVSKLQPLNIYALGQIEDQSVTYAADGDIRTERSEDSQLYRSVIQ
ypseu0001X_3841  ------------------------------------------------------------
yrohd0001_38400  ------------------------------------------------------------
yfred0001_40710  ------------------------------------------------------------
yente0001X_8000  ------------------------------------------------------------
yruck0001_35040  ------------------------------------------------------------
                                                                             


                       2110      2120      2130      2140      2150      2160
                 =========+=========+=========+=========+=========+=========+
yberc0001_8630   -----------GPEVREHATNQYASTLTAKTISGSG----------ATIHNGANSGATTP
ykris0001_26670  ------------------------------------------------------------
ykris0001_41280  ------------------------------------------------------------
yruck0001_910    ------------------------------------------------------------
yaldo0001_37900  ------------------------------------------------------------
yberc0001_34750  ----------------------------------------------SAETGSTIPEARRH
yaldo0001_38900  ------------------------------------------------------------
yaldo0001_41000  ------------------------------------------------------------
ypseu0001X_3846  ------------------------------------------------------------
ymoll0001_35980  ------------------------------------------------------------
ypest0001X_8100  ------------------------------------------------------------
yfred0001_38200  ------------------------------------------------------------
ypest0001X_2754  ------------------------------------------------------------
yfred0001_34120  ------------------------------------------------------------
ypseu0001X_2842  ------------------------------------------------------------
ykris0001_7890   ------------------------------------------------------------
yruck0001_4640   ------------------------------------------------------------
ymoll0001_2720   ------------------------------------------------------------
yrohd0001_40310  ------------------------------------------------------------
yruck0001_34980  ------------------------------------------------------------
ypest0001X_2756  ------------------------------------------------------------
yfred0001_45620  ------------------------------------------------------------
yrohd0001_18860  ------------------------------------------------------------
yfred0001_33010  ------------------------------------------------------------
yfred0001_33210  ------------------------------------------------------------
yrohd0001_40300  ------------------------------------------------------------
yruck0001_4620   ------------------------------------------------------------
ypest0001X_8110  ------------------------------------------------------------
yberc0001_40130  ------------------------------------------------------------
yberc0001_40750  ------------------------------------------------------------
yruck0001_35050  ------------------------------------------------------------
yruck0001_13030  ----------------------------------------------SDKNTKITSGGFYY
yinte0001_17980  ------------------------------------------------------------
yaldo0001_6040   ------------------------------------------------------------
yfred0001_33270  -----------GPEVSERATNQYASSITAQTLSGSG----------TTINNGANPGAIAP
yrohd0001_18870  ------------------------------------------------------------
yruck0001_4610   ------------------------------------------------------------
ypest0001X_2758  ------------------------------------------------------------
ypseu0001X_2843  ------------------------------------------------------------
ypseu0001X_2846  -----------GPEVSERATSEYASSFTAKTLSGSG----------TTINNGANPGAIAP
ypest0001X_2761  -----------GPEVSERATSEYASSFTAKTLSGSG----------TTINNGANPGAIAP
yrohd0001_32190  ------------------------------------------------------------
ymoll0001_21160  ------------------------------------------------------------
yrohd0001_32220  ------------------------------------------------------------
yfred0001_34080  ------------------------------------------------------------
yruck0001_4650   -----------GPEVKERPSTQYASSLTAKTLTGSG----------TVINNGANPGSIAS
yaldo0001_38920  ------------------------------------------------------------
yberc0001_20820  ------------------------------------------------------------
yaldo0001_3710   -----------GPEVKERTTNQYASSLNAKTVSGSG----------TIIHNGANPGAIAP
ypest0001X_8090  ------------------------------------------------------------
yfred0001_34100  ------------------------------------------------------------
yruck0001_25350  ------------------------------------------------------------
yfred0001_38190  ------------------------------------------------------------
yfred0001_34090  ------------------------------------------------------------
yaldo0001_30990  ------------------------------------------------------------
yberc0001_36600  ------------------------------------------------------------
yrohd0001_40100  ------------------------------------------------------------
yrohd0001_40280  ------------------------------------------------------------
yruck0001_4660   ------------------------------------------------------------
ypseu0001X_3844  ------------------------------------------------------------
ykris0001_41250  ------------------------------------------------------------
ykris0001_21250  ------------------------------------------------------------
yruck0001_4630   ------------------------------------------------------------
yrohd0001_40320  ------------------------------------------------------------
yrohd0001_40080  ------------------------------------------------------------
yrohd0001_32210  -----------GPEVQERATNQYASSLTAKTISGSG----------TTINNGANPGAVAP
yfred0001_45640  ------------------------------------------------------------
yfred0001_38220  ------------------------------------------------------------
yrohd0001_38410  -----------GPEVKEHTTNQYASSLTAKTISGSG----------ATINNGANPGVIPP
yfred0001_33220  -----------GPEVKENTTNQYASSLTAKTISGSG----------TTINNGANSGAISP
ymoll0001_36970  -----------GPEISDLTTTQYASSLTAQTISGSG----------ATINNGANPGTIAP
yrohd0001_39710  ------------------------------------------------------------
yrohd0001_18880  ------------------------------------------------------------
yinte0001_5480   -----------GPEVRERTTNQYASSLTAKTLSGSG----------TTINNGANPGAIAP
yfred0001_33200  -----------GPEVSARATNQYASSLTAQTVSGSG----------TTINNGANPGAIAP
yrohd0001_18890  ------------------------------------------------------------
yfred0001_32960  ------------------------------------------------------------
yberc0001_40160  ------------------------------------------------------------
ypseu0001X_3843  ------------------------------------------------------------
ypseu0001X_3837  ------------------------------------------------------------
ypest0001X_8140  ------------------------------------------------------------
ypseu0001X_3848  -----------GPEMKLRPNNQYASSITAKTLTGSG-----TVINNTVINNGAAPGAIVA
ypest0001X_8130  -----------GPEMKLRPNNQYASSITAKTLTGSG-----TVINNTVINNGTAPGAIVA
ypest0001X_8080  -----------GPEMKLRLNNQYASSITAKTLTGSGTVINNTVINNGAAPGAIVAPRDRD
yinte0001_41760  DSQLYRSVIQASGAVNAHFTGDISNTTATPNAGGVSHTLAAPKLDLLSQPDNIDTAQAQD
ykris0001_32060  A----------GGAVNAHFTGDISNTTATPNAGGVSHTLAAPKLDLQSQPDNIGTAQAQD
ypseu0001X_3841  ------------------------------------------------------------
yrohd0001_38400  ------------------------------------------------------------
yfred0001_40710  ------------------------------------------------------------
yente0001X_8000  ------------------------------------------------------------
yruck0001_35040  ------------------------------------------------------------
                                                                             


                       2170      2180      2190      2200      2210      2220
                 =========+=========+=========+=========+=========+=========+
yberc0001_8630   PGDRDNTGKEIAVEFDGISLTLPSGGLY---------QLNTDKGHYAPSPEGDLSLGSIN
ykris0001_26670  ------------------------------------------------------------
ykris0001_41280  ------------------------------------------------------------
yruck0001_910    ------------------------------------------------------------
yaldo0001_37900  ------------------------------------------------------------
yberc0001_34750  LSDANSAPVQASLAANGYD-----------------------------------------
yaldo0001_38900  ------------------------------------------------------------
yaldo0001_41000  ------------------------------------------------------------
ypseu0001X_3846  ------------------------------------------------------------
ymoll0001_35980  ------------------------------------------------------------
ypest0001X_8100  ------------------------------------------------------------
yfred0001_38200  ------------------------------------------------------------
ypest0001X_2754  ------------------------------------------------------------
yfred0001_34120  ------------------------------------------------------------
ypseu0001X_2842  ------------------------------------------------------------
ykris0001_7890   ------------------------------------------------------------
yruck0001_4640   ------------------------------------------------------------
ymoll0001_2720   ------------------------------------------------------------
yrohd0001_40310  ------------------------------------------------------------
yruck0001_34980  ------------------------------------------------------------
ypest0001X_2756  ------------------------------------------------------------
yfred0001_45620  ------------------------------------------------------------
yrohd0001_18860  ------------------------------------------------------------
yfred0001_33010  ------------------------------------------------------------
yfred0001_33210  ------------------------------------------------------------
yrohd0001_40300  ------------------------------------------------------------
yruck0001_4620   ------------------------------------------------------------
ypest0001X_8110  ------------------------------------------------------------
yberc0001_40130  ------------------------------------------------------------
yberc0001_40750  ------------------------------------------------------------
yruck0001_35050  ------------------------------------------------------------
yruck0001_13030  TGGIDRAGSGISVSHQD-------------------------------------------
yinte0001_17980  ----TMTGKDIKVVLSGAAET---------------------------------------
yaldo0001_6040   ------------------------------------------------------------
yfred0001_33270  PLGSNNSGKDLAIEFNGISLTPPNGGLY---------QFSTD------------------
yrohd0001_18870  ------------------------------------------------------------
yruck0001_4610   ------------------------------------------------------------
ypest0001X_2758  ------------------------------------------------------------
ypseu0001X_2843  ------------------------------------------------------------
ypseu0001X_2846  PADRDNSGKDLAIEFNGISLTPPNGGLY---------QFTTD------------------
ypest0001X_2761  PADRDNSGKDLAVEFNGISL----------------------TQP---------------
yrohd0001_32190  ------------------------------------------------------------
ymoll0001_21160  ------------------------------------------------------------
yrohd0001_32220  ------------------------------------------------------------
yfred0001_34080  ------------------------------------------------------------
yruck0001_4650   PASRDSTGKNITVEFNGVSLTLPSGGLY---------RLNTDKGDYAPEPDGGLSFANVN
yaldo0001_38920  ------------------------------------------------------------
yberc0001_20820  ------------------------------------------------------------
yaldo0001_3710   PSGNDSTGKDIAVEFNGISLTLPSGGLY---------QLNTDQGHYAANPAGELSLDSIK
ypest0001X_8090  ------------------------------------------------------------
yfred0001_34100  ------------------------------------------------------------
yruck0001_25350  ------------------------------------------------------------
yfred0001_38190  ------------------------------------------------------------
yfred0001_34090  ------------------------------------------------------------
yaldo0001_30990  ------------------------------------------------------------
yberc0001_36600  ------------------------------------------------------------
yrohd0001_40100  ------------------------------------------------------------
yrohd0001_40280  ------------------------------------------------------------
yruck0001_4660   ------------------------------------------------------------
ypseu0001X_3844  ------------------------------------------------------------
ykris0001_41250  ------------------------------------------------------------
ykris0001_21250  ------------------------------------------------------------
yruck0001_4630   ------------------------------------------------------------
yrohd0001_40320  ------------------------------------------------------------
yrohd0001_40080  ------------------------------------------------------------
yrohd0001_32210  PAGSATSGKNISVEFNGL------------------------------------------
yfred0001_45640  ------------------------------------------------------------
yfred0001_38220  ------------------------------------------------------------
yrohd0001_38410  PSGRDHTGKDLAVEFNGISLTLPSGGLY---------QLSTDKGHYAPDPEGKLSFDSI-
yfred0001_33220  PNNRDNIGKDIAVEFNGISLTLPSGGLY---------QLSTDKGHYAPTPEGDLSLGSIN
ymoll0001_36970  PNDRDNSGKDLAVEFNGISLTQPNGGLY---------QFNTD------------------
yrohd0001_39710  ------------------------------------------------------------
yrohd0001_18880  ------------------------------------------------------------
yinte0001_5480   PSGRDHTGKDLAVEFNGISLTLPNGGLY---------QLNTDKGHYAPDPEGKLSFDSIN
yfred0001_33200  PQGSNNSGKDLAIEFNGISLTPPNGGLY---------QFSTD------------------
yrohd0001_18890  ------------------------------------------------------------
yfred0001_32960  ------------------------------------------------------------
yberc0001_40160  ------------------------------------------------------------
ypseu0001X_3843  ------------------------------------------------------------
ypseu0001X_3837  ------------------------------------------------------------
ypest0001X_8140  ------------------------------------------------------------
ypseu0001X_3848  PRDRDSTGKNIAVEFNGISLTLPRSGLY---------QLKTDKGDYAPGPEAALSLANIS
ypest0001X_8130  PRDRDSTGKNIAVEFNGIALTLPRSGLY---------QLKTDKGDYAPGPEAALSLANIS
ypest0001X_8080  STGKNIAVEFNGIALTLPRSGLYQLK--------------TDKGDYAPGPEAALSLANIS
yinte0001_41760  LKDDQHITVGTPVWKDNLQNALGSLGN-NATELADYPLPNSNNGHFVLAPDPSSPYLITT
ykris0001_32060  LTDDQYITVGTPVWKDNLQNALGSLGNN-ATELADYPLPNSNNGHFVLAPDPSSPYLITT
ypseu0001X_3841  ------------------------------------------------------------
yrohd0001_38400  ------------------------------------------------------------
yfred0001_40710  ------------------------------------------------------------
yente0001X_8000  ------------------------------------------------------------
yruck0001_35040  ------------------------------------------------------------
                                                                             


                       2230      2240      2250      2260      2270      2280
                 =========+=========+=========+=========+=========+=========+
yberc0001_8630   NPSPLDP-----------------------------------------------------
ykris0001_26670  ------------------------------------------------------------
ykris0001_41280  ------------------------------------------------------------
yruck0001_910    ------------------------------------------------------------
yaldo0001_37900  ------------------------------------------------------------
yberc0001_34750  ------------------------------------------------------------
yaldo0001_38900  ------------------------------------------------------------
yaldo0001_41000  ------------------------------------------------------------
ypseu0001X_3846  ------------------------------------------------------------
ymoll0001_35980  ------------------------------------------------------------
ypest0001X_8100  ------------------------------------------------------------
yfred0001_38200  ------------------------------------------------------------
ypest0001X_2754  ------------------------------------------------------------
yfred0001_34120  ------------------------------------------------------------
ypseu0001X_2842  ------------------------------------------------------------
ykris0001_7890   ------------------------------------------------------------
yruck0001_4640   ------------------------------------------------------------
ymoll0001_2720   ------------------------------------------------------------
yrohd0001_40310  ------------------------------------------------------------
yruck0001_34980  ------------------------------------------------------------
ypest0001X_2756  ------------------------------------------------------------
yfred0001_45620  ------------------------------------------------------------
yrohd0001_18860  ------------------------------------------------------------
yfred0001_33010  ------------------------------------------------------------
yfred0001_33210  ------------------------------------------------------------
yrohd0001_40300  ------------------------------------------------------------
yruck0001_4620   ------------------------------------------------------------
ypest0001X_8110  ------------------------------------------------------------
yberc0001_40130  ------------------------------------------------------------
yberc0001_40750  ------------------------------------------------------------
yruck0001_35050  ------------------------------------------------------------
yruck0001_13030  ------------------------------------------------------------
yinte0001_17980  ------------------------------------------------------------
yaldo0001_6040   ------------------------------------------------------------
yfred0001_33270  ------------------------------------------------------------
yrohd0001_18870  ------------------------------------------------------------
yruck0001_4610   ------------------------------------------------------------
ypest0001X_2758  ------------------------------------------------------------
ypseu0001X_2843  ------------------------------------------------------------
ypseu0001X_2846  ------------------------------------------------------------
ypest0001X_2761  ------------------------------------------------------------
yrohd0001_32190  ------------------------------------------------------------
ymoll0001_21160  ------------------------------------------------------------
yrohd0001_32220  ------------------------------------------------------------
yfred0001_34080  ------------------------------------------------------------
yruck0001_4650   TPSTLDSTGKRFDSQQTTAVSLTPSTGELQQVNAVGGKYEPDPEGALSFASLSSPSFVDS
yaldo0001_38920  ------------------------------------------------------------
yberc0001_20820  ------------------------------------------------------------
yaldo0001_3710   TPS---------------------------------------------------------
ypest0001X_8090  ------------------------------------------------------------
yfred0001_34100  ------------------------------------------------------------
yruck0001_25350  ------------------------------------------------------------
yfred0001_38190  ------------------------------------------------------------
yfred0001_34090  ------------------------------------------------------------
yaldo0001_30990  ------------------------------------------------------------
yberc0001_36600  ------------------------------------------------------------
yrohd0001_40100  ------------------------------------------------------------
yrohd0001_40280  ------------------------------------------------------------
yruck0001_4660   ------------------------------------------------------------
ypseu0001X_3844  ------------------------------------------------------------
ykris0001_41250  ------------------------------------------------------------
ykris0001_21250  ------------------------------------------------------------
yruck0001_4630   ------------------------------------------------------------
yrohd0001_40320  ------------------------------------------------------------
yrohd0001_40080  ------------------------------------------------------------
yrohd0001_32210  ------------------------------------------------------------
yfred0001_45640  ------------------------------------------------------------
yfred0001_38220  ------------------------------------------------------------
yrohd0001_38410  ------------------------------------------------------------
yfred0001_33220  NPSA--------------------------------------------------------
ymoll0001_36970  ------------------------------------------------------------
yrohd0001_39710  ------------------------------------------------------------
yrohd0001_18880  ------------------------------------------------------------
yinte0001_5480   TPSA--------------------------------------------------------
yfred0001_33200  ------------------------------------------------------------
yrohd0001_18890  ------------------------------------------------------------
yfred0001_32960  ------------------------------------------------------------
yberc0001_40160  ------------------------------------------------------------
ypseu0001X_3843  ------------------------------------------------------------
ypseu0001X_3837  ------------------------------------------------------------
ypest0001X_8140  ------------------------------------------------------------
ypseu0001X_3848  PPSSLDATGQR-------------------------------------------------
ypest0001X_8130  PPSSLDATGPR-------------------------------------------------
ypest0001X_8080  PPSSLDATGPR-------------------------------------------------
yinte0001_41760  NPKLNELGQLD-------------------------------------------------
ykris0001_32060  NPKLNELGQLD-------------------------------------------------
ypseu0001X_3841  ------------------------------------------------------------
yrohd0001_38400  ------------------------------------------------------------
yfred0001_40710  ------------------------------------------------------------
yente0001X_8000  ------------------------------------------------------------
yruck0001_35040  ------------------------------------------------------------
                                                                             


                       2290      2300      2310      2320      2330      2340
                 =========+=========+=========+=========+=========+=========+
yberc0001_8630   -----------NLGGTNLATPGRAVSGGYLIETNPAFANLNNWRGSDYVLQQLNNDPSVI
ykris0001_26670  ------------------------------------------------------------
ykris0001_41280  ------------------------------------------------------------
yruck0001_910    ------------------------------------------------------------
yaldo0001_37900  ------------------------------------------------------------
yberc0001_34750  ------------------------------------------------------------
yaldo0001_38900  ------------------------------------------------------------
yaldo0001_41000  ------------------------------------------------------------
ypseu0001X_3846  ------------------------------------------------------------
ymoll0001_35980  ------------------------------------------------------------
ypest0001X_8100  ------------------------------------------------------------
yfred0001_38200  ------------------------------------------------------------
ypest0001X_2754  ------------------------------------------------------------
yfred0001_34120  ------------------------------------------------------------
ypseu0001X_2842  ------------------------------------------------------------
ykris0001_7890   ------------------------------------------------------------
yruck0001_4640   ------------------------------------------------------------
ymoll0001_2720   ------------------------------------------------------------
yrohd0001_40310  ------------------------------------------------------------
yruck0001_34980  ------------------------------------------------------------
ypest0001X_2756  ------------------------------------------------------------
yfred0001_45620  ------------------------------------------------------------
yrohd0001_18860  ------------------------------------------------------------
yfred0001_33010  ------------------------------------------------------------
yfred0001_33210  ------------------------------------------------------------
yrohd0001_40300  ------------------------------------------------------------
yruck0001_4620   ------------------------------------------------------------
ypest0001X_8110  ------------------------------------------------------------
yberc0001_40130  ------------------------------------------------------------
yberc0001_40750  ------------------------------------------------------------
yruck0001_35050  ------------------------------------------------------------
yruck0001_13030  ------------------------------------------------------------
yinte0001_17980  ------------------------------------------------------------
yaldo0001_6040   ------------------------------------------------------------
yfred0001_33270  ----------------------YTVNSQYLIETNPAFANLNNWRGSDYVLQQLNNDPNVI
yrohd0001_18870  ------------------------------------------------------------
yruck0001_4610   ------------------------------------------------------------
ypest0001X_2758  ------------------------------------------------------------
ypseu0001X_2843  ------------------------------------------------------------
ypseu0001X_2846  ---------------------HTVGGGGYLIETHPAFANLNNWRGSDYVLQQLNNDPDVI
ypest0001X_2761  -----------NGGLYQFTTDHTVGGGGYLIETHPAFANLNNWRGSDYVLQQLNNDPDVI
yrohd0001_32190  ------------------------------------------------------------
ymoll0001_21160  ------------------------------------------------------------
yrohd0001_32220  ------------------------------------------------------------
yfred0001_34080  ------------------------------------------------------------
yruck0001_4650   TGQQLNSNQSQGLNRSNLSMPNRAVGGGYLIETNPAFANLNHWKGSDAYLQTLNNDPSLI
yaldo0001_38920  ------------------------------------------------------------
yberc0001_20820  ------------------------------------------------------------
yaldo0001_3710   ------T----TVDRTGLSAPERAVSGGYLVETHPAFANLNNWRGSDYVLQQLNNDPDVI
ypest0001X_8090  ------------------------------------------------------------
yfred0001_34100  ------------------------------------------------------------
yruck0001_25350  ------------------------------------------------------------
yfred0001_38190  ------------------------------------------------------------
yfred0001_34090  ------------------------------------------------------------
yaldo0001_30990  ------------------------------------------------------------
yberc0001_36600  ------------------------------------------------------------
yrohd0001_40100  ------------------------------------------------------------
yrohd0001_40280  ------------------------------------------------------------
yruck0001_4660   ------------------------------------------------------------
ypseu0001X_3844  ------------------------------------------------------------
ykris0001_41250  ------------------------------------------------------------
ykris0001_21250  ------------------------------------------------------------
yruck0001_4630   ------------------------------------------------------------
yrohd0001_40320  ------------------------------------------------------------
yrohd0001_40080  ------------------------------------------------------------
yrohd0001_32210  ------------------------------------------------------------
yfred0001_45640  ------------------------------------------------------------
yfred0001_38220  ------------------------------------------------------------
yrohd0001_38410  ------------------------------------------------------------
yfred0001_33220  -----------NLDRTGLTAPDRAVSGGYLIETNPAFANLNNWKGSDYYLQQLNSDPSFI
ymoll0001_36970  ----------------------YTVSGGYLIETHPAFANLNNWRGSDYVLQQLNSDPDVI
yrohd0001_39710  ------------------------------------------------------------
yrohd0001_18880  ------------------------------------------------------------
yinte0001_5480   -----------NVDRTGLAAPDRAVSGGYLVETNPAFANLNNWRGSDYVLQQLNNDPDVI
yfred0001_33200  ----------------------YTVNSQYLIETNPAFANLNNWRGSDYVLQQLNNDPNVI
yrohd0001_18890  ------------------------------------------------VLQQLNNDPNVI
yfred0001_32960  ------------------------------------------------------------
yberc0001_40160  ------------------------------------------------------------
ypseu0001X_3843  ------------------------------------------------------------
ypseu0001X_3837  ------------------------------------------------------------
ypest0001X_8140  ------------------------------------------------------------
ypseu0001X_3848  ----GVPPPSDDLNRTGLVTPDRAVSGGYLVETHPAFASLNNWKGSDLYLQQLSSDPSVI
ypest0001X_8130  ----GVPPPSDDLNRTGLVTPDRAVSGGYLVETHPAFASLNNWKGSDLYLQQLSSDPSVI
ypest0001X_8080  ----GVPPPSDDLNRTGLVTPDRAVSGGYLVETHPAFASLNNWKGSDLYLQQLSSDPSVI
yinte0001_41760  -----------NSLFDGLYAMLGQQPEAAPQENNSQFTDQKQFLGSAYFLDRLNLKPDYD
ykris0001_32060  -----------NSLFDGLYAMLGQQPGAAPQENNSQFTDQKQFLGSAYFLDRLNLKPDYD
ypseu0001X_3841  ------------------------------------------------------------
yrohd0001_38400  ------------------------------------------------------------
yfred0001_40710  ------------------------------------------------------------
yente0001X_8000  ------------------------------------------------------------
yruck0001_35040  ------------------------------------------------------------
                                                                             


                       2350      2360      2370      2380      2390      2400
                 =========+=========+=========+=========+=========+=========+
yberc0001_8630   HKRLGDNIYEQRLVRDQVLALTGQTVATGYSDAQAQFEQLFAAGLEYSKAFNLALGTHLS
ykris0001_26670  ------------------------------------------------------------
ykris0001_41280  ------------------------------------------------------------
yruck0001_910    ------------------------------------------------------------
yaldo0001_37900  ------------------------------------------------------------
yberc0001_34750  ------------------------------------------------------------
yaldo0001_38900  ------------------------------------------------------------
yaldo0001_41000  ------------------------------------------------------------
ypseu0001X_3846  ------------------------------------------------------------
ymoll0001_35980  ------------------------------------------------------------
ypest0001X_8100  ------------------------------------------------------------
yfred0001_38200  ------------------------------------------------------------
ypest0001X_2754  ------------------------------------------------------------
yfred0001_34120  ------------------------------------------------------------
ypseu0001X_2842  ------------------------------------------------------------
ykris0001_7890   ------------------------------------------------------------
yruck0001_4640   ------------------------------------------------------------
ymoll0001_2720   ------------------------------------------------------------
yrohd0001_40310  ------------------------------------------------------------
yruck0001_34980  ------------------------------------------------------------
ypest0001X_2756  ------------------------------------------------------------
yfred0001_45620  ------------------------------------------------------------
yrohd0001_18860  ------------------------------------------------------------
yfred0001_33010  ------------------------------------------------------------
yfred0001_33210  ------------------------------------------------------------
yrohd0001_40300  ------------------------------------------------------------
yruck0001_4620   ------------------------------------------------------------
ypest0001X_8110  ------------------------------------------------------------
yberc0001_40130  ------------------------------------------------------------
yberc0001_40750  ------------------------------------------------------------
yruck0001_35050  ------------------------------------------------------------
yruck0001_13030  ------------------------------------------------------------
yinte0001_17980  ------------------------------------------------------------
yaldo0001_6040   ------------------------------------------------------------
yfred0001_33270  FKRLGDNAYEQRLVRDQVLALTGQAVASDYRSAQEQFEALFAAGLEYSKAFNIALGTHLS
yrohd0001_18870  ------------------------------------------------------------
yruck0001_4610   ------------------------------------------------------------
ypest0001X_2758  ------------------------------------------------------------
ypseu0001X_2843  ------------------------------------------------------------
ypseu0001X_2846  FKRLGDNAYEQRLVRDQVLALTGQAVASDYRSAQEQFEALFAAGLEYSKAFNIALGTHLS
ypest0001X_2761  FKRLGDNAYEQRLVRDQVLALTGQAVASDYRSAQEQFEALFAAGLEYSKAFNIALGTHLS
yrohd0001_32190  ------------------------------------------------------------
ymoll0001_21160  ------------------------------------------------------------
yrohd0001_32220  ------------------------------------------------------------
yfred0001_34080  ------------------------------------------------------------
yruck0001_4650   HKRLGDNMYEQRLVRDQVLALTGKTVASDYRSAQEQFEQLFAAGAQYSKRFNLSPGTHLS
yaldo0001_38920  ------------------------------------------------------------
yberc0001_20820  ------------------------------------------------------------
yaldo0001_3710   FKRLGDNAYEQRLVRDQVLALTGKTVSSDYRSAQEQFEQLFAAGLEYSKAFNLALGTHLS
ypest0001X_8090  ------------------------------------------------------------
yfred0001_34100  ------------------------------------------------------------
yruck0001_25350  ------------------------------------------------------------
yfred0001_38190  ------------------------------------------------------------
yfred0001_34090  ------------------------------------------------------------
yaldo0001_30990  ------------------------------------------------------------
yberc0001_36600  ------------------------------------------------------------
yrohd0001_40100  ------------------------------------------------------------
yrohd0001_40280  ------------------------------------------------------------
yruck0001_4660   ------------------------------------------------------------
ypseu0001X_3844  ------------------------------------------------------------
ykris0001_41250  ------------------------------------------------------------
ykris0001_21250  ------------------------------------------------------------
yruck0001_4630   ------------------------------------------------------------
yrohd0001_40320  ------------------------------------------------------------
yrohd0001_40080  ------------------------------------------------------------
yrohd0001_32210  ------------------------------------------------------------
yfred0001_45640  ------------------------------------------------------------
yfred0001_38220  ------------------------------------------------------------
yrohd0001_38410  ------------------------------------------------------------
yfred0001_33220  HKRLGDNAYEQRLVRDQVLALTGQTVASDYRSAQEQFEQLFAAGIEYSKAFNIALGSHLS
ymoll0001_36970  FKRLGDNAYEQRLVRDQVLALTGQVVASDYRSAQEQFEALFAAGLEYSQAFNLALGTHLS
yrohd0001_39710  ------------------------------------------------------------
yrohd0001_18880  ------------------------------------------------------------
yinte0001_5480   FKRLGDNAYEQRLVRDQVLALTGQAVASDYRSAQEQFEQLFAAGLEYSKTFNLALGTHLS
yfred0001_33200  FKRLGDNAYEQRLVRDQVLALTGQTVASDYRSAQEQFEELFAAGLEYSEAFNIALGTHLS
yrohd0001_18890  FKRLGDNAYEQRLVRDQVLALTGQAVASDYRSAQEQFEELFAAGLEYSKAFNIALGTHLS
yfred0001_32960  ------------------------------------------------------------
yberc0001_40160  ------------------------------------------------------------
ypseu0001X_3843  ------------------------------------------------------------
ypseu0001X_3837  ------------------------------------------------------------
ypest0001X_8140  ------------------------------------------------------------
ypseu0001X_3848  HKRLGDNAYEQRLLRDQVLALTGRTVASDYRSEQAQFEQLFAAGVQYSKAFNLAPGTRLS
ypest0001X_8130  HKRLGDNAYEQRLLRDQVLALTGRTVASDYRSEQAQFEQLFAAGVQYRKAFNLAPGTRLS
ypest0001X_8080  HKRLGDNAYEQRLLRDQVLALTGRTVASDYRSEQAQFEQLFAAGVQYSKAFNLAPGTRLS
yinte0001_41760  YRFLGDAAFDTRYISNAVLSQTGQRYLTGLGSDLAQMQYLIDNAAQAQSGLGLTLGVSLT
ykris0001_32060  YRFLGDAAFDTRYISNAVLSQTGQRYLNGLGSDLAQMQYLIDNAAQAQSGLGLTLGVSLT
ypseu0001X_3841  ------------------------------------------------------------
yrohd0001_38400  ------------------------------------------------------------
yfred0001_40710  ------------------------------------------------------------
yente0001X_8000  ------------------------------------------------------------
yruck0001_35040  ------------------------------------------------------------
                                                                             


                       2410      2420      2430      2440      2450      2460
                 =========+=========+=========+=========+=========+=========+
yberc0001_8630   AEQMAALTGNIVLMETREVAGQTVLVPVVYLAGVKPGDLQANGALIAANNIELTDMQGLT
ykris0001_26670  ------------------------------------------------------------
ykris0001_41280  ------------------------------------------------------------
yruck0001_910    ------------------------------------------------------------
yaldo0001_37900  ------------------------------------------------------------
yberc0001_34750  --------------------GQKVIDTATYDAQQHVASQHGERAAFAEQGVVPQEVAGYT
yaldo0001_38900  ------------------------------------------------------------
yaldo0001_41000  ------------------------------------------------------------
ypseu0001X_3846  ------------------------------------------------------------
ymoll0001_35980  ------------------------------------------------------------
ypest0001X_8100  ------------------------------------------------------------
yfred0001_38200  ------------------------------------------------------------
ypest0001X_2754  ------------------------------------------------------------
yfred0001_34120  ------------------------------------------------------------
ypseu0001X_2842  ------------------------------------------------------------
ykris0001_7890   ------------------------------------------------------------
yruck0001_4640   ------------------------------------------------------------
ymoll0001_2720   ------------------------------------------------------------
yrohd0001_40310  ------------------------------------------------------------
yruck0001_34980  ------------------------------------------------------------
ypest0001X_2756  ------------------------------------------------------------
yfred0001_45620  ------------------------------------------------------------
yrohd0001_18860  ------------------------------------------------------------
yfred0001_33010  ------------------------------------------------------------
yfred0001_33210  ------------------------------------------------------------
yrohd0001_40300  ------------------------------------------------------------
yruck0001_4620   ------------------------------------------------------------
ypest0001X_8110  ------------------------------------------------------------
yberc0001_40130  ------------------------------------------------------------
yberc0001_40750  ------------------------------------------------------------
yruck0001_35050  ------------------------------------------------------------
yruck0001_13030  ------------------------------------------------------------
yinte0001_17980  ---------------SKKNNASSMLLPSSIKAGSHILIKTIGNAYYQATEFSSEQGGGHI
yaldo0001_6040   ------------------------------------------------------------
yfred0001_33270  AEQMAALTTNIVLMESREVAGQTVLVPVVYLAGVKPGDLQANGALIAAENITLTEVQGFT
yrohd0001_18870  ------------------------------------------------------------
yruck0001_4610   ------------------------------------------------------------
ypest0001X_2758  ------------------------------------------------------------
ypseu0001X_2843  ------------------------------------------------------------
ypseu0001X_2846  AEQMAALTHNIVLMETRDVAGQTVLVPVVYLAGVKPGDLQANGALIAAENISLTEVQGFT
ypest0001X_2761  AEQMAALTHNIVLMETRDVAGQTVLVPVVYLAGVKPGDLQANGALIAAENISLTEVQGFT
yrohd0001_32190  ------------------------------------------------------------
ymoll0001_21160  ------------------------------------------------------------
yrohd0001_32220  ------------------------------------------------------------
yfred0001_34080  ------------------------------------------------------------
yruck0001_4650   AEQMATLTDNIVLMETREVAGQTVLVPVVYLARVKPGELHANGALIAADNIELTDVQGLT
yaldo0001_38920  ------------------------------------------------------------
yberc0001_20820  ------------------------------------------------------------
yaldo0001_3710   AGQMAALTSNIVLMESRVVAGQTVLVPVVYLAGVKPGDLRANGALIAANNIELTNVQGFT
ypest0001X_8090  ------------------------------------------------------------
yfred0001_34100  ------------------------------------------------------------
yruck0001_25350  ------------------------------------------------------------
yfred0001_38190  ------------------------------------------------------------
yfred0001_34090  ------------------------------------------------------------
yaldo0001_30990  ------------------------------------------------------------
yberc0001_36600  ------------------------------------------------------------
yrohd0001_40100  ------------------------------------------------------------
yrohd0001_40280  ------------------------------------------------------------
yruck0001_4660   ------------------------------------------------------------
ypseu0001X_3844  ------------------------------------------------------------
ykris0001_41250  ------------------------------------------------------------
ykris0001_21250  ------------------------------------------------------------
yruck0001_4630   ------------------------------------------------------------
yrohd0001_40320  ---------------------------VVYLAGVKSGDLQANGALISAENIALTEMQGFT
yrohd0001_40080  ---------------------------VVYLAGVKPGDLQANGALIAAENITLTDVQGFN
yrohd0001_32210  ------------------------------------------------------------
yfred0001_45640  ------------------------------------------------------------
yfred0001_38220  ------------------------------------------------------------
yrohd0001_38410  ------------------------------------------------------------
yfred0001_33220  AEQMAALTSNIVLMETREIAGETVLVPVVYLVGVKPGDLHANGALIAANNIELTDVQGLS
ymoll0001_36970  AEQMAALTRDIVLMESREVAGQTVLVPVVYLAGVKPGDLQANGALIAANNIELTDMQGFS
yrohd0001_39710  ------------------------------------------------------------
yrohd0001_18880  ------------------------------------------------------------
yinte0001_5480   AEQMAALTHNIVLMESRDVAGQTVLVPVVYLAGVKPGDLQANGALIAAENIELTEVKGFT
yfred0001_33200  AEQMATLTTNIVLMESREVAGQTVLVPVVYLAGVKPGDLQANGALISAENIALTEVQGFT
yrohd0001_18890  AEQMAALTTNIVLMETREVAXXN-------------------------------------
yfred0001_32960  ------------------------------------------------------------
yberc0001_40160  ------------------------------------------------------------
ypseu0001X_3843  ------------------------------------------------------------
ypseu0001X_3837  ------------------------------------------------------------
ypest0001X_8140  ------------------------------------------------------------
ypseu0001X_3848  AEQMATLTGNIVLMENRDVAGQTVLVPVVYLAGVKPGDLRANGALIAAENISLTEVQGFA
ypest0001X_8130  AEQMATLTGNIVLME---------------------------------------------
ypest0001X_8080  AEQMATLTGNIVLMENRDVAGQTVLVPVVYLAGVKPGDLRANGALIAAENISLTEVQGFA
yinte0001_41760  AEQVAALNKSIVWWEEINVNGQTVLAPKLYLAKADSASL--TGSIIAGNQVNLDAGKVIN
ykris0001_32060  AEQVAALNKSIVWWEEINVNGQTVLAPKLYLAKADSASL--NGSVISGNQVNLAAGKVIN
ypseu0001X_3841  ------------------------------------------------------------
yrohd0001_38400  ------------------------------------------------------------
yfred0001_40710  ------------------------------------------------------------
yente0001X_8000  ------------------------------------------------------------
yruck0001_35040  ------------------------------------------------------------
                                                                             


                       2470      2480      2490      2500      2510      2520
                 =========+=========+=========+=========+=========+=========+
yberc0001_8630   NQGAIKATNNLQISMAKDITLTSNGGLLQAGGNMQLSTLNSDIDLTGARLNATNLQLDSG
ykris0001_26670  ------------------------------------------------------------
ykris0001_41280  ------------------------------------------------------------
yruck0001_910    ------------------------------------------------------------
yaldo0001_37900  ------------------------------------------------------------
yberc0001_34750  G-----------------------------------------------------------
yaldo0001_38900  ------------------------------------------------------------
yaldo0001_41000  ------------------------------------------------------------
ypseu0001X_3846  ------------------------------------------------------------
ymoll0001_35980  ------------------------------------------------------------
ypest0001X_8100  ------------------------------------------------------------
yfred0001_38200  ------------------------------------------------------------
ypest0001X_2754  ------------------------------------------------------------
yfred0001_34120  ------------------------------------------------------------
ypseu0001X_2842  ------------------------------------------------------------
ykris0001_7890   ------------------------------------------------------------
yruck0001_4640   ------------------------------------------------------------
ymoll0001_2720   ------------------------------------------------------------
yrohd0001_40310  ------------------------------------------------------------
yruck0001_34980  ------------------------------------------------------------
ypest0001X_2756  ------------------------------------------------------------
yfred0001_45620  ------------------------------------------------------------
yrohd0001_18860  ------------------------------------------------------------
yfred0001_33010  ------------------------------------------------------------
yfred0001_33210  ------------------------------------------------------------
yrohd0001_40300  ------------------------------------------------------------
yruck0001_4620   ------------------------------------------------------------
ypest0001X_8110  ------------------------------------------------------------
yberc0001_40130  ------------------------------------------------------------
yberc0001_40750  ------------------------------------------------------------
yruck0001_35050  ------------------------------------------------------------
yruck0001_13030  ------------------------------------------------------------
yinte0001_17980  KSGGDIYFDQVIDSRSNNATGTHGKGKLTLGGGMGSKELRLD------------------
yaldo0001_6040   ------------------------------------------------------------
yfred0001_33270  NQGAMKAGNDLSISMAQDINLNNRGGLLEAGNNMLLSTLNSDIDLTSARLNATNLQLDSG
yrohd0001_18870  ------------------------------------------------------------
yruck0001_4610   ------------------------------------------------------------
ypest0001X_2758  ------------------------------------------------------------
ypseu0001X_2843  ------------------------------------------------------------
ypseu0001X_2846  NAGAITATNDLKISMAQDITLNNRGGLLQAGGDMQLSTLNSDIDLTSARINATNLQLDSG
ypest0001X_2761  NAGAITATNDLKISMAQDITLNNRGGLLQAGGDMQLSTLNSDIDLTSARINATNLQLDSG
yrohd0001_32190  ------------------------------------------------------------
ymoll0001_21160  ------------------------------------------------------------
yrohd0001_32220  ------------------------------------------------------------
yfred0001_34080  ------------------------------------------------------------
yruck0001_4650   NQGAIKATDNLRIGMAKDIILNSRGGLLQAGNNLQLSTLNSDIDLTSARINATNLQLDSG
yaldo0001_38920  ------------------------------------------------------------
yberc0001_20820  ------------------------------------------------------------
yaldo0001_3710   NQGAITATNNLHISMAQDITLNNRGGLLDAGNNLQLSTLNSDIDLTGARLNATNLQLDSG
ypest0001X_8090  ------------------------------------------------------------
yfred0001_34100  ------------------------------------------------------------
yruck0001_25350  ---------------------------VDIGANVDYSGITRPIEKAVNKIGNLDVL----
yfred0001_38190  ------------------------------------------------------------
yfred0001_34090  ------------------------------------------------------------
yaldo0001_30990  ------------------------------------------------------------
yberc0001_36600  ------------------------------------------------------------
yrohd0001_40100  ------------------------------------------------------------
yrohd0001_40280  ------------------------------------------------------------
yruck0001_4660   ------------------------------------------------------------
ypseu0001X_3844  ------------------------------------------------------------
ykris0001_41250  ------------------------------------------------------------
ykris0001_21250  ------------------------------------------------------------
yruck0001_4630   ------------------------------------------------------------
yrohd0001_40320  NSGALKATHNLNLSMAQDMALNNRGGLLEAGNNLQLSTLNSDIDLTGSRLTATNLQLDSG
yrohd0001_40080  NSGAIKASNDLQISMAQDMALNNRGGLLQAGNNMQLSTLNSDIDLTGSRLTATNLQLDSG
yrohd0001_32210  ------------------------------------------------------------
yfred0001_45640  ------------------------------------------------------------
yfred0001_38220  ------------------------------------------------------------
yrohd0001_38410  ------------------------------------------------------------
yfred0001_33220  HQGAIKATHDLRISMAKDITLTSNGGLLQAGNNLQLSTLNSDIDLTGARLNATNLQLDSG
ymoll0001_36970  NSGAIKATHDLSISMAQDITLNNHGGLLQAGNNMLLSTLNSDIDLTSARLNATNLQLDSG
yrohd0001_39710  ------------------------------------------------------------
yrohd0001_18880  ------------------------------------------------------------
yinte0001_5480   NQGAIKASNDLRINMAKDITLTSNGGLLQAGGNMQLSTLNSDIDLTGTRLNATNLQLDSG
yfred0001_33200  NQGAINATNDLQISMAQDINLNNRGGLLQAGGNMLLSTLNSDIDLTGSRLTATNLQLDSG
yrohd0001_18890  ------------------------------------------------------------
yfred0001_32960  ------------------------------------------------------------
yberc0001_40160  ------------------------------------------------------------
ypseu0001X_3843  ------------------------------------------------------------
ypseu0001X_3837  ------------------------------------------------------------
ypest0001X_8140  ------------------------------------------------------------
ypseu0001X_3848  NAGAISATNNLQISMAKDITLNNRGGLLQAGNHLQLSTLNSDIDLTGARLNATNLQLDSG
ypest0001X_8130  ------------------------------------------------------------
ypest0001X_8080  NAGAISASNNLQISMAKDITLNNRCGLLQAGNHLQLSTLNSDIDLTSARLNATNLQLDSG
yinte0001_41760  VESTLKADQLLAVNSLTTLS-NLQGGKITSGGDLQLSAISDISNIGSSIAGQRVELESLD
ykris0001_32060  AGSTLKADQLLAVNSQITLS-NLQGGKITSGGDLQLSAIGDISNIGSSIAGQRVALESLD
ypseu0001X_3841  ------------------------------------------------------------
yrohd0001_38400  ------------------------------------------------------------
yfred0001_40710  ------------------------------------------------------------
yente0001X_8000  ------------------------------------------------------------
yruck0001_35040  ------------------------------------------------------------
                                                                             


                       2530      2540      2550      2560      2570      2580
                 =========+=========+=========+=========+=========+=========+
yberc0001_8630   RDLILRTDSEQLSSNG---------AVLRNQTILGPLASINVTNNAVINSDRDFIMQGAG
ykris0001_26670  ------------------------------------------------------------
ykris0001_41280  ------------------------------------------------------------
yruck0001_910    ------------------------------------------------------------
yaldo0001_37900  ------------------------------------------------------------
yberc0001_34750  ------------------------------------------------------------
yaldo0001_38900  ------------------------------------------------------------
yaldo0001_41000  ------------------------------------------------------------
ypseu0001X_3846  ------------------------------------------------------------
ymoll0001_35980  ------------------------------------------------------------
ypest0001X_8100  ------------------------------------------------------------
yfred0001_38200  ------------------------------------------------------------
ypest0001X_2754  ------------------------------------------------------------
yfred0001_34120  ------------------------------------------------------------
ypseu0001X_2842  ------------------------------------------------------------
ykris0001_7890   ------------------------------------------------------------
yruck0001_4640   ------------------------------------------------------------
ymoll0001_2720   ------------------------------------------------------------
yrohd0001_40310  ------------------------------------------------------------
yruck0001_34980  ------------------------------------------------------------
ypest0001X_2756  ------------------------------------------------------------
yfred0001_45620  ------------------------------------------------------------
yrohd0001_18860  ------------------------------------------------------------
yfred0001_33010  ------------------------------------------------------------
yfred0001_33210  ------------------------------------------------------------
yrohd0001_40300  ------------------------------------------------------------
yruck0001_4620   ------------------------------------------------------------
ypest0001X_8110  ------------------------------------------------------------
yberc0001_40130  ------------------------------------------------------------
yberc0001_40750  ------------------------------------------------------------
yruck0001_35050  ------------------------------------------------------------
yruck0001_13030  -------------------------HEENSSSDSGIVSRTDIKGSLLINAKGDITHQGAQ
yinte0001_17980  ------------------------------------------------------------
yaldo0001_6040   ------------------------------------------------------------
yfred0001_33270  RDVILRTGSEQLSSDNG--------AVQRNQTVLGPLASINVSNNAVINTERDFIMQGAG
yrohd0001_18870  ------------------------------------------------------------
yruck0001_4610   ------------------------------------------------------------
ypest0001X_2758  ------------------------------------------------------------
ypseu0001X_2843  ------------------------------------------------------------
ypseu0001X_2846  RDVILRTDSAQLSSDNG--------AVSRDQTILGPLASINVSNNATINTGRDFIMQGAS
ypest0001X_2761  RDVILRTDSAQLSSDNG--------AVSRDQTILGPLASINVSNNATINTGRDFIMQGAS
yrohd0001_32190  ------------------------------------------------------------
ymoll0001_21160  ------------------------------------------------------------
yrohd0001_32220  ------------------------------------------------------------
yfred0001_34080  ------------------------------------------------------------
yruck0001_4650   RDVILRTAGDQLSSNNG--------VVQRTQTVLGPLASINISNNAVINTERDFIQQGAG
yaldo0001_38920  ------------------------------------------------------------
yberc0001_20820  ------------------------------------------------------------
yaldo0001_3710   RDVILRTDTEQLSSSNG--------SVLRNQTLLGPLASINVSNNAVINTARDFIQQGAG
ypest0001X_8090  ------------------------------------------------------------
yfred0001_34100  ------------------------------------------------------------
yruck0001_25350  ------------------------------------------------------------
yfred0001_38190  ------------------------------------------------------------
yfred0001_34090  ------------------------------------------------------------
yaldo0001_30990  ------------------------------------------------------------
yberc0001_36600  ------------------------------------------------------------
yrohd0001_40100  ------------------------------------------------------------
yrohd0001_40280  ------------------------------------------------------------
yruck0001_4660   ------------------------------------------------------------
ypseu0001X_3844  ------------------------------------------------------------
ykris0001_41250  ------------------------------------------------------------
ykris0001_21250  ------------------------------------------------------------
yruck0001_4630   ------------------------------------------------------------
yrohd0001_40320  RDIILRTDSAQLSSDNG--------AVQRTQTLLGPLASINVSNNAVINTERDFILQGAS
yrohd0001_40080  RDLILRTGTEQLSSSNG--------SVLRNQTLLGPLASINISNNAVINTERDFILQGAS
yrohd0001_32210  ------------------------------------------------------------
yfred0001_45640  ------------------------------------------------------------
yfred0001_38220  ------------------------------------------------------------
yrohd0001_38410  ------------------------------------------------------------
yfred0001_33220  RDLILRTDTEQLSSSNG--------AVLRNQTLLGPLASINVSNNAVINTGRDFILQGAS
ymoll0001_36970  RDLILRTSSEQLSSDNG--------AVLRNQTILGPLASINVSNNATINTERDFIQQGAG
yrohd0001_39710  ------------------------------------------------------------
yrohd0001_18880  ------------------------------------------------------------
yinte0001_5480   RDVVLRTSSEQLSSSNG--------AVLRNQTLLGPLASINVSNNAAINTGRDFILQGAG
yfred0001_33200  RDIILRTDSAQLSSDNG--------AVQRSQTVLGPLASINVSNNAVINTERDFIMQGAG
yrohd0001_18890  ------------------------------------------------------------
yfred0001_32960  ------------------------------------------------------------
yberc0001_40160  ------------------------------------------------------------
ypseu0001X_3843  ------------------------------------------------------------
ypseu0001X_3837  ------------------------------------------------------------
ypest0001X_8140  ------------------------------------------------------------
ypseu0001X_3848  RDVILRTASDQYSSGNG--------AVQRTQTILGPLASLNISNNAVITAQRDFIQQGAG
ypest0001X_8130  ------------------------------------------------------------
ypest0001X_8080  RDVILRTASDQYSSGNG--------AVQRTQTILGPLASLNISNNAVITAQRDFIQQGAG
yinte0001_41760  GNIINQTLSQQWTATAAGDGRWDRESLSLTRTEIGDTATISAGDSLSLNAGKDILVTGAK
ykris0001_32060  GNIINQTLSQQWTATTAGNGRWNSESLSLTRTEIGDTSTISAGDSLSLNAGKDILVTGAK
ypseu0001X_3841  ------------------------------------------------------------
yrohd0001_38400  ------------------------------------------------------------
yfred0001_40710  ------------------------------------------------------------
yente0001X_8000  ------------------------------------------------------------
yruck0001_35040  ------------------------------------------------------------
                                                                             


                       2590      2600      2610      2620      2630      2640
                 =========+=========+=========+=========+=========+=========+
yberc0001_8630   LNVGKDLQVNTGGDWLLNT----VQTRDQISANYGYGHASSEHIRHLGSEVNVGGALTAK
ykris0001_26670  ------------------------------------------------------------
ykris0001_41280  ------------------------------------------------------------
yruck0001_910    ------------------------------------------------------------
yaldo0001_37900  ------------------------------------------------------------
yberc0001_34750  ------------------------------------------------------------
yaldo0001_38900  ------------------------------------------------------------
yaldo0001_41000  ------------------------------------------------------------
ypseu0001X_3846  ------------------------------------------------------------
ymoll0001_35980  ------------------------------------------------------------
ypest0001X_8100  ------------------------------------------------------------
yfred0001_38200  ------------------------------------------------------------
ypest0001X_2754  ------------------------------------------------------------
yfred0001_34120  ------------------------------------------------------------
ypseu0001X_2842  ------------------------------------------------------------
ykris0001_7890   ------------------------------------------------------------
yruck0001_4640   ------------------------------------------------------------
ymoll0001_2720   ------------------------------------------------------------
yrohd0001_40310  ------------------------------------------------------------
yruck0001_34980  ------------------------------------------------------------
ypest0001X_2756  ------------------------------------------------------------
yfred0001_45620  ------------------------------------------------------------
yrohd0001_18860  ------------------------------------------------------------
yfred0001_33010  ------------------------------------------------------------
yfred0001_33210  ------------------------------------------------------------
yrohd0001_40300  ------------------------------------------------------------
yruck0001_4620   ------------------------------------------------------------
ypest0001X_8110  ------------------------------------------------------------
yberc0001_40130  ------------------------------------------------------------
yberc0001_40750  ------------------------------------------------------------
yruck0001_35050  ------------------------------------------------------------
yruck0001_13030  HKVTEEYHAQGGTINNLASNNIYIDRSNHEKWNGGLGFNIDYSGITRPIRKSLEKKLETE
yinte0001_17980  ------------------------------------------------------------
yaldo0001_6040   ------------------------------------------------------------
yfred0001_33270  LNVGQDLQVNTGGDWLLNT----VETRDQITANSGRSSSTSEHIRHLGSEVNVGGALTAN
yrohd0001_18870  ------------------------------------------------------------
yruck0001_4610   ------------------------------------------------------------
ypest0001X_2758  ------------------------------------------------------------
ypseu0001X_2843  ------------------------------------------------------------
ypseu0001X_2846  LNVGQDLQVTTGGDWKLET----VQTRDQISTHDGRGSATSEHIRHLGSEVNVGGALTAN
ypest0001X_2761  LNVGQDLQVTTGGDWQLET----VQTRDQISTHDGRGSATSEHIRHLGSEVNVGGALTAN
yrohd0001_32190  ------------------------------------------------------------
ymoll0001_21160  ------------------------------------------------------------
yrohd0001_32220  ------------------------------------------------------------
yfred0001_34080  ------------------------------------------------------------
yruck0001_4650   LNVGQDLQVNTGGGWILNT----VQSSDQISANYGYGRSTSEHIRHLGSEVNVGGALTAK
yaldo0001_38920  ------------------------------------------------------------
yberc0001_20820  ------------------------------------------------------------
yaldo0001_3710   VNVGQDLQVNTGGDWLLNT----VERSDQISTSDGRSSSTSEHIRHLGSEVNVGGALTAN
ypest0001X_8090  ------------------------------------------------------------
yfred0001_34100  ------------------------------------------------------------
yruck0001_25350  ------------------------------------------------------------
yfred0001_38190  ------------------------------------------------------------
yfred0001_34090  ------------------------------------------------------------
yaldo0001_30990  ------------------------------------------------------------
yberc0001_36600  ------------------------------------------------------------
yrohd0001_40100  ------------------------------------------------------------
yrohd0001_40280  ------------------------------------------------------------
yruck0001_4660   ------------------------------------------------------------
ypseu0001X_3844  ------------------------------------------------------------
ykris0001_41250  ------------------------------------------------------------
ykris0001_21250  ------------------------------------------------------------
yruck0001_4630   ------------------------------------------------------------
yrohd0001_40320  VNVGKDLQVNTGGDWILNT----VERSDQISANYGYGSSTSEHIRHLGSEVNVGGALTAK
yrohd0001_40080  VNVGKDLQVNTGGDWILNT----VQTRDQISGQYSGGSSTSEHIRHLGSEVNVGGALTAN
yrohd0001_32210  ------------------------------------------------------------
yfred0001_45640  ------------------------------------------------------------
yfred0001_38220  ------------------------------------------------------------
yrohd0001_38410  ------------------------------------------------------------
yfred0001_33220  LTVGKDLQVNTGGDWILNT----VERSDQISADYGYGHATSEHIRHFGSEVNVSGALTAK
ymoll0001_36970  LNVGQDLQVNTGGDWLLNT----LQSSDQISTHYGSGSATSEHIRHLGSEVNVGGALTAN
yrohd0001_39710  --------------------------------------------------VNVGGALTAN
yrohd0001_18880  ------------------------------------------------------------
yinte0001_5480   LNVGKDLQVNTGGDWILNT----VQSSDQISANYGYGSSTSEHIRHLGSEVNVGGALIAK
yfred0001_33200  LNVGQDLQVNTGGDWLLNT----VQTRDQITANYGRSSSTSEHIRHLGSEVNVGGALTAN
yrohd0001_18890  ------------------------------------------------------------
yfred0001_32960  ------------------------------------------------------------
yberc0001_40160  ------------------------------------------------------------
ypseu0001X_3843  ------------------------------------------------------------
ypseu0001X_3837  -----------------------VQRSDQISAQYGGGSATSGSLRHLGSEVKVGGALSAN
ypest0001X_8140  ------------------------------------------------------------
ypseu0001X_3848  INIGKDLQVNTGGDWLLST----VQRSDQISAQYGGGSATSGSLRHLGSEVKVGGALSAN
ypest0001X_8130  ------------------------------------------------------------
ypest0001X_8080  INIGKDLQVNTGGDWLLST----VQRSDQISAQYGGGSATSGSLRHLGSEVKVGGALSAN
yinte0001_41760  VSAGGNLDVQAGGDVDIKANTTLSNNEHNQQRDRRNGYKQSEQRDSLGSEISAGGNLTLN
ykris0001_32060  VSAGGNLDMQAGGDIAITANTTLSNNEHNLQRDRRNGYQQSEQRDSLSSEISAGGDLTLN
ypseu0001X_3841  ------------------------------------------------------------
yrohd0001_38400  ------------------------------------------------------------
yfred0001_40710  ------------------------------------------------------------
yente0001X_8000  ------------------------------------------------------------
yruck0001_35040  ------------------------------------------------------------
                                                                             


                       2650      2660      2670      2680      2690      2700
                 =========+=========+=========+=========+=========+=========+
yberc0001_8630   VDNLTAV-----------------------------------------------------
ykris0001_26670  ------------------------------------------------------------
ykris0001_41280  ------------------------------------------------------------
yruck0001_910    ------------------------------------------------------------
yaldo0001_37900  ------------------------------------------------------------
yberc0001_34750  ------------------------------------------------------------
yaldo0001_38900  ------------------------------------------------------------
yaldo0001_41000  ------------------------------------------------------------
ypseu0001X_3846  ------------------------------------------------------------
ymoll0001_35980  ------------------------------------------------------------
ypest0001X_8100  ------------------------------------------------------------
yfred0001_38200  ------------------------------------------------------------
ypest0001X_2754  ------------------------------------------------------------
yfred0001_34120  ------------------------------------------------------------
ypseu0001X_2842  ------------------------------------------------------------
ykris0001_7890   ------------------------------------------------------------
yruck0001_4640   ------------------------------------------------------------
ymoll0001_2720   ------------------------------------------------------------
yrohd0001_40310  ------------------------------------------------------------
yruck0001_34980  ------------------------------------------------------------
ypest0001X_2756  ------------------------------------------------------------
yfred0001_45620  ------------------------------------------------------------
yrohd0001_18860  ------------------------------------------------------------
yfred0001_33010  ------------------------------------------------------------
yfred0001_33210  ------------------------------------------------------------
yrohd0001_40300  ------------------------------------------------------------
yruck0001_4620   ------------------------------------------------------------
ypest0001X_8110  ------------------------------------------------------------
yberc0001_40130  ------------------------------------------------------------
yberc0001_40750  ------------------------------------------------------------
yruck0001_35050  ------------------------------------------------------------
yruck0001_13030  GLVIPNA-----------------------------------------------------
yinte0001_17980  ------------------------------------------------------------
yaldo0001_6040   ------------------------------------------------------------
yfred0001_33270  VDNLTAV-----------------------------------------------------
yrohd0001_18870  ------------------------------------------------------------
yruck0001_4610   ------------------------------------------------------------
ypest0001X_2758  ------------------------------------------------------------
ypseu0001X_2843  ------------------------------------------------------------
ypseu0001X_2846  VDNLTAV-----------------------------------------------------
ypest0001X_2761  VDNLTAV-----------------------------------------------------
yrohd0001_32190  ------------------------------------------------------------
ymoll0001_21160  ------------------------------------------------------------
yrohd0001_32220  ------------------------------------------------------------
yfred0001_34080  ------------------------------------------------------------
yruck0001_4650   VDNFTAV-----------------------------------------------------
yaldo0001_38920  ------------------------------------------------------------
yberc0001_20820  ------------------------------------------------------------
yaldo0001_3710   VNNLTAV-----------------------------------------------------
ypest0001X_8090  ------------------------------------------------------------
yfred0001_34100  ------------------------------------------------------------
yruck0001_25350  ------------------------------------------------------------
yfred0001_38190  ------------------------------------------------------------
yfred0001_34090  ------------------------------------------------------------
yaldo0001_30990  ------------------------------------------------------------
yberc0001_36600  ------------------------------------------------------------
yrohd0001_40100  ------------------------------------------------------------
yrohd0001_40280  ------------------------------------------------------------
yruck0001_4660   ------------------------------------------------------------
ypseu0001X_3844  ------------------------------------------------------------
ykris0001_41250  ------------------------------------------------------------
ykris0001_21250  ------------------------------------------------------------
yruck0001_4630   ------------------------------------------------------------
yrohd0001_40320  VDNLTAV-----------------------------------------------------
yrohd0001_40080  VSDLTAV-----------------------------------------------------
yrohd0001_32210  ------------------------------------------------------------
yfred0001_45640  ------------------------------------------------------------
yfred0001_38220  ------------------------------------------------------------
yrohd0001_38410  ------------------------------------------------------------
yfred0001_33220  VDNLSAV-----------------------------------------------------
ymoll0001_36970  VNNLTAV-----------------------------------------------------
yrohd0001_39710  VNNLTAV-----------------------------------------------------
yrohd0001_18880  ------------------------------------------------------------
yinte0001_5480   VDNLTAV-----------------------------------------------------
yfred0001_33200  VNNLAAV-----------------------------------------------------
yrohd0001_18890  ------------------------------------------------------------
yfred0001_32960  ------------------------------------------------------------
yberc0001_40160  ------------------------------------------------------------
ypseu0001X_3843  ------------------------------------------------------------
ypseu0001X_3837  VDNLTAV-----------------------------------------------------
ypest0001X_8140  ------V-----------------------------------------------------
ypseu0001X_3848  VDNLTAV-----------------------------------------------------
ypest0001X_8130  ------------------------------------------------------------
ypest0001X_8080  VDNLTAV-----------------------------------------------------
yinte0001_41760  AGNDVSLTASELAAKGNVGLSAGRDISLETAEKNSQQKTNNSENRTTDATRSVITSGNNL
ykris0001_32060  AGNDVSLTASELAAKGNVGLSAGRDISLETAEKNSQQKTNNSENRTTDATRSVITSGNNL
ypseu0001X_3841  ------------------------------------------------------------
yrohd0001_38400  ------------------------------------------------------------
yfred0001_40710  ------------------------------------------------------------
yente0001X_8000  ------------------------------------------------------------
yruck0001_35040  ------------------------------------------------------------
                                                                             


                       2710      2720      2730      2740      2750      2760
                 =========+=========+=========+=========+=========+=========+
yberc0001_8630   ----GANINAGTIDVQAQNINLSAATDSLQVTGKATTKRHSDSVDLYDET-----LLGSQ
ykris0001_26670  ------------------------------------------------------------
ykris0001_41280  ------------------------------------------------------------
yruck0001_910    ------------------------------------------------------------
yaldo0001_37900  ------------------------------------------------------------
yberc0001_34750  ------------------------------------------------------------
yaldo0001_38900  ------------------------------------------------------------
yaldo0001_41000  ------------------------------------------------------------
ypseu0001X_3846  ------------------------------------------------------------
ymoll0001_35980  ------------------------------------------------------------
ypest0001X_8100  ------------------------------------------------------------
yfred0001_38200  ------------------------------------------------------------
ypest0001X_2754  ------------------------------------------------------------
yfred0001_34120  ------------------------------------------------------------
ypseu0001X_2842  ------------------------------------------------------------
ykris0001_7890   ------------------------------------------------------------
yruck0001_4640   ------------------------------------------------------------
ymoll0001_2720   ------------------------------------------------------------
yrohd0001_40310  ------------------------------------------------------------
yruck0001_34980  ------------------------------------------------------------
ypest0001X_2756  ------------------------------------------------------------
yfred0001_45620  ------------------------------------------------------------
yrohd0001_18860  ------------------------------------------------------------
yfred0001_33010  ------------------------------------------------------------
yfred0001_33210  ------------------------------------------------------------
yrohd0001_40300  ------------------------------------------------------------
yruck0001_4620   ------------------------------------------------------------
ypest0001X_8110  ------------------------------------------------------------
yberc0001_40130  ------------------------------------------------------------
yberc0001_40750  ------------------------------------------------------------
yruck0001_35050  ------------------------------------------------------------
yruck0001_13030  ------------------------------------------------------------
yinte0001_17980  ------------------------------------------------------------
yaldo0001_6040   ------------------------------------------------------------
yfred0001_33270  ----GANINANTIDIQAQNINLSAATDSLHVTGESSSNRHTSSVNLYDET-----LRGSQ
yrohd0001_18870  ------------------------------------------------------------
yruck0001_4610   ------------------------------------------------------------
ypest0001X_2758  ------------------------------------------------------------
ypseu0001X_2843  ------------------------------------------------------------
ypseu0001X_2846  ----GANINAATLEVQAQNISLSAATDSLHVTGESSSKRHTSSVNLYDET-----LLGSQ
ypest0001X_2761  ----GANINAATLEVQAQNISLSAATDSLHVTGESSSKRHTSSVNLYDET-----LLGSQ
yrohd0001_32190  ------------------------------------------------------------
ymoll0001_21160  ------------------------------------------------------------
yrohd0001_32220  ------------------------------------------------------------
yfred0001_34080  ------------------------------------------------------------
yruck0001_4650   ----GARINAGNIHLQAQNIDLSAASDRLQVTGSSSSKRHTSSVNLYDET-----LLGSE
yaldo0001_38920  ------------------------------------------------------------
yberc0001_20820  ------------------------------------------------------------
yaldo0001_3710   ----GANINANTIDVQAQNITLSAATDSLHVTGESSSKRHTSSVDLYDET-----LLGSQ
ypest0001X_8090  ------------------------------------------------------------
yfred0001_34100  ------------------------------------------------------------
yruck0001_25350  ----GAANDLGAIGTPNLGIDIGAKGSSSDTQKNTTTAVVTS------------------
yfred0001_38190  ------------------------------------------------------------
yfred0001_34090  ------------------------------------------------------------
yaldo0001_30990  ------------------------------------------------------------
yberc0001_36600  ------------------------------------------------------------
yrohd0001_40100  ------------------------------------------------------------
yrohd0001_40280  ------------------------------------------------------------
yruck0001_4660   ------------------------------------------------------------
ypseu0001X_3844  ------------------------------------------------------------
ykris0001_41250  ------------------------------------------------------------
ykris0001_21250  ------------------------------------------------------------
yruck0001_4630   ------------------------------------------------------------
yrohd0001_40320  ----GANINANTI-----------------------------------------------
yrohd0001_40080  ----GANINANTI-----------------------------------------------
yrohd0001_32210  ------------------------------------------------------------
yfred0001_45640  ------------------------------------------------------------
yfred0001_38220  -------------------------VDSLQVTGKVSTKRHSDSVDLYDET-----LLGSQ
yrohd0001_38410  ------------------------------------------------------------
yfred0001_33220  ----GANINAGTVDVQAQNINLSAATDSLHVTGESSSKRHTSSVDLYDET-----LLGSQ
ymoll0001_36970  ----GASINADTIDVQAQNINLSAATDSLSVTGESSSKRHTSSVNLYDET-----LRGSQ
yrohd0001_39710  ----GANINASTIDVQAQNINLSAATDSLQVSGESSSKRHTSSVELYDET-----LRGSQ
yrohd0001_18880  ------------------------------------------------------------
yinte0001_5480   ----GANINAGAVDVQAQNINLSAATDSLHVTGESSSTRHTSSVDLYDET-----LRGSQ
yfred0001_33200  ----GATINAGSIDVQAQNINLSAATDSLNVTGESSSKRSRSSVDLYDET-----LLGSQ
yrohd0001_18890  ------------------------------------------------------------
yfred0001_32960  ------------------------------------------------------------
yberc0001_40160  ------------------------------------------------------------
ypseu0001X_3843  ------------------------------------------------------------
ypseu0001X_3837  ----GARVNAGTIDVQAQNITLSAATDSLSVTGGSSSKRHTAALNLY-------------
ypest0001X_8140  ----GARVNAGTIDVQAQNITLSAATDSLSVTGGSSSKRHTAALNLY-------------
ypseu0001X_3848  ----GARVNAGTIDVRAQNITLSAATDSLSVTGVSSSKRHTSSVNLYDET-----LLGSQ
ypest0001X_8130  ------------------------------------------------------------
ypest0001X_8080  ----GARVNAGTIDVRAQNITLSAATDSLSVTGGSSSKRHTSSVNLYDET-----LLGSQ
yinte0001_41760  TLDAGRDINSQAAALVSDNDSTLKAGRDVNLNAQQSSTYSESHGDRKQQINESIRQQGTE
ykris0001_32060  TLDAGRDINSQAAALVSDNATTLKAGRDVNLNAQQSSTYSENHGDRKQQINESIRQQGTE
ypseu0001X_3841  ------------------------------------------------------------
yrohd0001_38400  ------------------------------------------------------------
yfred0001_40710  ------------------------------------------------------------
yente0001X_8000  ------------------------------------------------------------
yruck0001_35040  ------------------------------------------------------------
                                                                             


                       2770      2780      2790      2800      2810      2820
                 =========+=========+=========+=========+=========+=========+
yberc0001_8630   LNAKGDINLQAAKDITLSASAIQTDGALKLAAGGD-VTLTTQTEQHDELRIHTGKHKGLA
ykris0001_26670  ------------------------------------------------------------
ykris0001_41280  ------------------------------------------------------------
yruck0001_910    ------------------------------------------------------------
yaldo0001_37900  ------------------------------------------------------------
yberc0001_34750  ------------------------------------------------------------
yaldo0001_38900  ------------------------------------------------------------
yaldo0001_41000  ------------------------------------------------------------
ypseu0001X_3846  ------------------------------------------------------------
ymoll0001_35980  ------------------------------------------------------------
ypest0001X_8100  ------------------------------------------------------------
yfred0001_38200  ------------------------------------------------------------
ypest0001X_2754  ------------------------------------------------------------
yfred0001_34120  ------------------------------------------------------------
ypseu0001X_2842  ------------------------------------------------------------
ykris0001_7890   ------------------------------------------------------------
yruck0001_4640   ------------------------------------------------------------
ymoll0001_2720   ------------------------------------------------------------
yrohd0001_40310  ------------------------------------------------------------
yruck0001_34980  ------------------------------------------------------------
ypest0001X_2756  ------------------------------------------------------------
yfred0001_45620  ------------------------------------------------------------
yrohd0001_18860  ------------------------------------------------------------
yfred0001_33010  ------------------------------------------------------------
yfred0001_33210  ------------------------------------------------------------
yrohd0001_40300  ------------------------------------------------------------
yruck0001_4620   ------------------------------------------------------------
ypest0001X_8110  ------------------------------------------------------------
yberc0001_40130  ------------------------------------------------------------
yberc0001_40750  ------------------------------------------------------------
yruck0001_35050  ------------------------------------------------------------
yruck0001_13030  ------------------------------------KPPTAGMDITLNKKDTDKSDKKIV
yinte0001_17980  ------------------------------------------------------------
yaldo0001_6040   ------------------------------------------------------------
yfred0001_33270  LNATGDIHLQAAKDIKLSASAVQTDGALKLAAGGD-ITLTTQTEQHDEQRNHTGTKKGLA
yrohd0001_18870  ------------------------------------------------------------
yruck0001_4610   ------------------------------------------------------------
ypest0001X_2758  ------------------------------------------------------------
ypseu0001X_2843  ------------------------------------------------------------
ypseu0001X_2846  LNATGDINLQAAQDITLRASAVQTDGALTLAAGGD-VLLTTQTEQHDEQRNHTGLSKGIA
ypest0001X_2761  LNATGDINLQAAQDITLRASAVQTDGALTLAAGGD-VLLTTQTEQHDEQRNHTGLSKGIA
yrohd0001_32190  ------------------------------------------------------------
ymoll0001_21160  ------------------------------------------------------------
yrohd0001_32220  ------------------------------------------------------------
yfred0001_34080  ------------------------------------------------------------
yruck0001_4650   LISKGNIHLQAVRDINLSASKVETEGAMKLAAGGD-VTLTTQTEQHDAQRNHTGKSKGLA
yaldo0001_38920  ------------------------------------------------------------
yberc0001_20820  ------------------------------------------------------------
yaldo0001_3710   LNAKGDINLKAAKDITLSASTVQTDGALKLAAGGD-VTLTTQTEQHDAQRNHTGVSKGLA
ypest0001X_8090  ------------------------------------------------------------
yfred0001_34100  ------------------------------------------------------------
yruck0001_25350  --------LQAGSIDTTTKGEVKDQGTQYNATKGSVNIDAQSHHSETAVNQEEIHSRETK
yfred0001_38190  ------------------------------------------------------------
yfred0001_34090  ------------------------------------------------------------
yaldo0001_30990  ------------------------------------------------------------
yberc0001_36600  ------------------------------------------------------------
yrohd0001_40100  ------------------------------------------------------------
yrohd0001_40280  ------------------------------------------------------------
yruck0001_4660   ------------------------------------------------------------
ypseu0001X_3844  ------------------------------------------------------------
ykris0001_41250  ------------------------------------------------------------
ykris0001_21250  ------------------------------------------------------------
yruck0001_4630   ------------------------------------------------------------
yrohd0001_40320  ------------------------------------------------------------
yrohd0001_40080  ------------------------------------------------------------
yrohd0001_32210  ------------------------------------------------------------
yfred0001_45640  ------------------------------------------------------------
yfred0001_38220  LNARGDINLQTAKDMTLSASTVQTDGALKLVAGGDV------------------------
yrohd0001_38410  ------------------------------------------------------------
yfred0001_33220  LNAKGDINLQAAKDMTLSASTVQTDGALKLAAGGDV------------------------
ymoll0001_36970  LNATGDINLQAAKDINISASTVQTNGALNLAAGGD-VTLTTQTEQHDEQRNHTGTKKGLA
yrohd0001_39710  LNATGDINLQAAKDINISASAVQTDGALKLAAGGD-VNLTTQTEQHDEQRNHTGISKSLA
yrohd0001_18880  LNAKSDINLQAAKDINISASAVQTDGALTLAAGGD-ITLTTQTX----------------
yinte0001_5480   LNATGDINLQAAKDINISASAVQTDGALKLAAGGDVVTLTTQTEQHDAQRNHTGISKGLA
yfred0001_33200  LNATGDINLNTAHDINISASAVQTSGALTLAAGGD-VNLTTQTEQHDAQRTHTGTSKGLA
yrohd0001_18890  ------------------------------------------------------------
yfred0001_32960  ------------------------------------------------------------
yberc0001_40160  ------------------------------------------------------------
ypseu0001X_3843  ------------------------------------------------------------
ypseu0001X_3837  ------------------------------------------------------------
ypest0001X_8140  ------------------------------------------------------------
ypseu0001X_3848  LNATDDINLQTANDITLSASAVQTDGALKLAAGGD-VTLTSQTEQHDEQRNHTGTKKGLV
ypest0001X_8130  ------------------------------------------------------------
ypest0001X_8080  LNATGDINLQTVNDITLSASAVQTDGALKLAAGGD-VTLTSQTEQHDEQRNHTGTKKGLL
yinte0001_41760  IVSGGDTTILAGNDINLQATQAQASGDIALKAGHDINVATARESDYSFFEETTVKKKRLS
ykris0001_32060  IVSGGDTTILAGHDINLQATQAQASGDIALKAGHDINVTTATESDYSFFEETTVKKKRLS
ypseu0001X_3841  ------------------------------------------------------------
yrohd0001_38400  ------------------------------------------------------------
yfred0001_40710  ------------------------------------------------------------
yente0001X_8000  ------------------------------------------------------------
yruck0001_35040  ------------------------------------------------------------
                                                                             


                       2830      2840      2850      2860      2870      2880
                 =========+=========+=========+=========+=========+=========+
yberc0001_8630   SSSTTHTEDSRSQTLAVGSMLSAGSLDISGKNIAVSGSNVVADNDVSLRAKENLTIGTAQ
ykris0001_26670  ------------------------------------------------------------
ykris0001_41280  ------------------------------------------------------------
yruck0001_910    ------------------------------------------------------------
yaldo0001_37900  ------------------------------------------------------------
yberc0001_34750  --------------------YKEGFNTVASNETNRKLSDAMDGEDNRARAEAGANHHMAL
yaldo0001_38900  ------------------------------------------------------------
yaldo0001_41000  ------------------------------------------------------------
ypseu0001X_3846  ------------------------------------------------------------
ymoll0001_35980  ------------------------------------------------------------
ypest0001X_8100  ------------------------------------------------------------
yfred0001_38200  ------------------------------------------------------------
ypest0001X_2754  ------------------------------------------------------------
yfred0001_34120  ------------------------------------------------------------
ypseu0001X_2842  ------------------------------------------------------------
ykris0001_7890   ------------------------------------------------------------
yruck0001_4640   ------------------------------------------------------------
ymoll0001_2720   ------------------------------------------------------------
yrohd0001_40310  ------------------------------------------------------------
yruck0001_34980  ------------------------------------------------------------
ypest0001X_2756  ------------------------------------------------------------
yfred0001_45620  ------------------------------------------------------------
yrohd0001_18860  ----------------VGSMLSAGSIDVSGKNISVMGSNVVADNDINLRAKENIIVGTAQ
yfred0001_33010  ------------------------------------------------------------
yfred0001_33210  ------------------------------------------------------------
yrohd0001_40300  ------------------------------------------------------------
yruck0001_4620   ------------------------------------------------------------
ypest0001X_8110  ------------------------------------------------------------
yberc0001_40130  ------------------------------------------------------------
yberc0001_40750  ------------------------------------------------------------
yruck0001_35050  ------------------------------------------------------------
yruck0001_13030  AYVTTIHAGKGIVEHATGTMIDEGTQYNSAGAIKITANDYFNNTAEITSLGHQHDIHGQG
yinte0001_17980  ------------------------------------------------------------
yaldo0001_6040   ------------------------------------------------------------
yfred0001_33270  SSTTTRTEDSISQTLAVGSVLSAGSMDISGKNIAVIGSNVVADNDINLRAQENIIIGTAQ
yrohd0001_18870  ----------------VGSMLSAASIDVSGKNIAVIGSNVVADNDINLRAQENIIIGTAQ
yruck0001_4610   ------------------------------------------------------------
ypest0001X_2758  ------------------------------------------------------------
ypseu0001X_2843  ------------------------------------------------------------
ypseu0001X_2846  SSTLTRTEDSLSQTLAVGSMLSAGSIDVSGKNIAVMGSNVVADQDISLRAQENITVGTAQ
ypest0001X_2761  SSTLTRTEDSLSQTLAVGSMLSAGSIDVSGKNIAVMGSNVVADQDISLRAQENITVGTAQ
yrohd0001_32190  ------------------------------------------------------------
ymoll0001_21160  ------------------------------------------------------------
yrohd0001_32220  ------------------------------------------------------------
yfred0001_34080  ------------------------------------------------------------
yruck0001_4650   STTTTRTEDSLSQRLAVGSMLSAGTIDVSGKNIAVTGSHVVADKEINLRAKENITVGTAQ
yaldo0001_38920  ------------------------------------------------------------
yberc0001_20820  ------------------------------------------------------------
yaldo0001_3710   SSTTLRTEDSMSQTLAVGSMLSAGSIDVSGKNIAVIGSNVVADNDINLRAKENITVGTAQ
ypest0001X_8090  ------------------------------------------------------------
yfred0001_34100  ------------------------------------------------------------
yruck0001_25350  GSAGARVYTTTGSDVTVDAKGEGGTTRSEKTANTAVTGNMTAADGISIKVKDNASYQGTS
yfred0001_38190  ------------------------------------------------------------
yfred0001_34090  ------------------------------------------------------------
yaldo0001_30990  ------------------------------------------------------------
yberc0001_36600  ------------------------------------------------------------
yrohd0001_40100  ------------------------------------------------------------
yrohd0001_40280  ------------------------------------------------------------
yruck0001_4660   ------------------------------------------------------------
ypseu0001X_3844  ------------------------------------------------------------
ykris0001_41250  ------------------------------------------------------------
ykris0001_21250  ------------------------------------------------------------
yruck0001_4630   ------------------------------------------------------------
yrohd0001_40320  ------------------------------------------------------------
yrohd0001_40080  ------------------------------------------------------------
yrohd0001_32210  ------------------------------------------------------------
yfred0001_45640  ------------------------------------------------------------
yfred0001_38220  ------------------------------------------------------------
yrohd0001_38410  ------------------------------------------------------------
yfred0001_33220  ------------------------------------------------------------
ymoll0001_36970  SSTTTRTEDSLSQTLAVGSMLSAGSIDVSGKNIAVIGSHVVADNDINLRAKENITVGTAQ
yrohd0001_39710  SSTTTRTEDSINQTLEVGSMLSAGSIDVSGKNIAVMGSNVVADNDINLRAKENITIGTAQ
yrohd0001_18880  ------------------------------------------------------------
yinte0001_5480   STTTTRTEDSISQTLAVGSMLSAGSIDISGNTIAVTGSNVVADNDINLRAKENLTVSTAQ
yfred0001_33200  SSTTTRTEDSISQTLAVGSMLSAGSIDVSGKNIVVIGSNVVADNDINLRAQENIIIGTAQ
yrohd0001_18890  ------------------------------------------------------------
yfred0001_32960  ------------------------------------------------------------
yberc0001_40160  ------------------------------------------------------------
ypseu0001X_3843  -------------------XLSAGSIDVSSQNIAVAGSSVVADKDIRLRAQENLTVSTAQ
ypseu0001X_3837  ------------------------------------------------------------
ypest0001X_8140  ------------------------------------------------------------
ypseu0001X_3848  SSTTARSEEGRSQTLAVGSMLSAGSIDVSSQNIAVAGSSVVADKDIRLRAQENLTVSTAQ
ypest0001X_8130  ------------------------------------------------------------
ypest0001X_8080  SSTTTRSEEGRSQTLAVGSMLSAGSIDVSSQNIAVAGSSVVADKDIRLRAQENLTVSTAQ
yinte0001_41760  KTTTHVVSEDYATQEQGSLLSGKNVSVSAGNDLLVKGSAVVGDNNVALTAGNNVDIVAAT
ykris0001_32060  KTTTHVVSEDYATQEQGSLLSGKNVSLSAGNDLLVKGSAVVGDNNVALTAGNNVDIVAAT
ypseu0001X_3841  ------------------------------------------------------------
yrohd0001_38400  ------------------------------------------------------------
yfred0001_40710  ------------------------------------------------------------
yente0001X_8000  ------------------------------------------------------------
yruck0001_35040  ------------------------------------------------------------
                                                                             


                       2890      2900      2910      2920      2930      2940
                 =========+=========+=========+=========+=========+=========+
yberc0001_8630   QRESESHLYEKKKSGLMSTGGIGVTVG--SNRQKTTDQAQTLTNIGSTVGSLGGNVTLEA
ykris0001_26670  ------------------------------------------------------------
ykris0001_41280  ------------------------------------------------------------
yruck0001_910    ------------------------------------------------------------
yaldo0001_37900  ------------------------------------------------------------
yberc0001_34750  NKDNAEGLHK--------------------------------------------------
yaldo0001_38900  ------------------------------------------------------------
yaldo0001_41000  ------------------------------------------------------------
ypseu0001X_3846  ------------------------------------------------------------
ymoll0001_35980  ------------------------------------------------------------
ypest0001X_8100  ------------------------------------------------------------
yfred0001_38200  ------------------------------------------------------------
ypest0001X_2754  ------------------------------------------------------------
yfred0001_34120  ------------------------------------------------------------
ypseu0001X_2842  ------------------------------------------------------------
ykris0001_7890   ------------------------------------------------------------
yruck0001_4640   ------------------------------------------------------------
ymoll0001_2720   ------------------------------------------------------------
yrohd0001_40310  ------------------------------------------------------------
yruck0001_34980  ------------------------------------------------------------
ypest0001X_2756  ------------------------------------------------------------
yfred0001_45620  ------------------------------------------------------------
yrohd0001_18860  QSESESHLHEQKKSGLMSTGGIGVTIG--SNSQKTTDNTQTLSNVGSTIGSLGGNVTLDA
yfred0001_33010  ------------------------------------------------------------
yfred0001_33210  ------------------------------------------------------------
yrohd0001_40300  ------------------------------------------------------------
yruck0001_4620   ------------------------------------------------------------
ypest0001X_8110  ------------------------------------------------------------
yberc0001_40130  ------------------------------------------------------------
yberc0001_40750  ------------------------------------------------------------
yruck0001_35050  ------------------------------------------------------------
yruck0001_13030  KLRVATSSGKDIKISLKGQGGVNISDY-------------------YTQDALPGIIQAKD
yinte0001_17980  ---------------------------------------------------LGGGYQKND
yaldo0001_6040   ------------------------------------------------------------
yfred0001_33270  QSESESHLYEQKKSGLMSTGGIGV------------------------------------
yrohd0001_18870  QSESESHLFEQKKSGLMSTGGIGVTVG--SNSTKVX------------------------
yruck0001_4610   ------------------------------------------------------------
ypest0001X_2758  ------------------------------------------------------------
ypseu0001X_2843  ------------------------------------------------------------
ypseu0001X_2846  QSESESHLFEQKKSGLMSTGGIGVTVG--SSSTKMTDSGQSISSVGSTVGSVLGNVSMTA
ypest0001X_2761  QSESESHLFEQKKSGLMSTGGIGVTVG--SSSTKMTDSGQSISSVGSTVGSVLGNVSMTA
yrohd0001_32190  ------------------------------------------------------------
ymoll0001_21160  ------------------------------------------------------------
yrohd0001_32220  ------------------------------------------------------------
yfred0001_34080  ------------------------------------------------------------
yruck0001_4650   QSESESHLFEQKKSGLMTTGGIGITVG--SNSNKVTDNGKTFSSVGSMVGSVQGNVNMTA
yaldo0001_38920  ------------------------------------------------------------
yberc0001_20820  ------------------------------------------------------------
yaldo0001_3710   QSESESHLFEQTKSGLMGTGGIGVTVG--SNSQKTTDKTQTLSNVGSTVGSVLGNVSMTA
ypest0001X_8090  ------------------------------------------------------------
yfred0001_34100  ------------------------------------------------------------
yruck0001_25350  LDAGAGK----------------------VSVKAGGDIQFNQAADTNSENHNGFNVKLSA
yfred0001_38190  ------------------------------------------------------------
yfred0001_34090  ------------------------------------------------------------
yaldo0001_30990  ------------------------------------------------------------
yberc0001_36600  ------------------------------------------------------------
yrohd0001_40100  ------------------------------------------------------------
yrohd0001_40280  ------------------------------------------------------------
yruck0001_4660   ------------------------------------------------------------
ypseu0001X_3844  ------------------------------------------------------------
ykris0001_41250  ------------------------------------------------------------
ykris0001_21250  ------------------------------------------------------------
yruck0001_4630   ------------------------------------------------------------
yrohd0001_40320  ------------------------------------------------------------
yrohd0001_40080  ------------------------------------------------------------
yrohd0001_32210  ------------------------------------------------------------
yfred0001_45640  ------------------------------------------------------------
yfred0001_38220  ------------------------------------------------------------
yrohd0001_38410  ------------------------------------------------------------
yfred0001_33220  ------------------------------------------------------------
ymoll0001_36970  QIESESHLYEQKKSGLMSTGGIGVMVG--SSSTKMTDSGQSISNVGSTVGSVLGNVSMTA
yrohd0001_39710  QSESESHLYEQKKSGLMSTGGIGVTVG--SNSTKVTXLIN--------------------
yrohd0001_18880  ------------------------------------------------------------
yinte0001_5480   QSENESHLFEQKKSGLMSTGGIGVTVG--SNSQKTTDTGKSLSNVGSTVGSLGGNVTLDA
yfred0001_33200  QSESESHLREEKKSGLMSTGGIGV------------------------------------
yrohd0001_18890  ------------------------------------------------------------
yfred0001_32960  ------------------------------------------------------------
yberc0001_40160  ------------------------------------------------------------
ypseu0001X_3843  QSESGSQLFEQKKSGLMSTGGIGVFIG--TSRQKTTDQTQTVSHIGSTVGSLTGNVRLEA
ypseu0001X_3837  ------------------------------------------------------------
ypest0001X_8140  ------------------------------------------------------------
ypseu0001X_3848  QSESGSQLFEQKKSGLMSTGGIGVFIG--TSRQKTTDQTQTVSHIGSTVGSLTGNVRLEA
ypest0001X_8130  ------------------------------------------------------------
ypest0001X_8080  QSESGSQLFEQKKSGLMSTGGIGVFIG--TSRQKTTDQTQTVSHVGSTVGSLTGNVRLEA
yinte0001_41760  EEQSSYRLSEQKKSGMFSGGGIGVTIGSTSSRQQSRDSGTTQSQSASTIGSTGGDVAIKA
ykris0001_32060  EEQSSYRLSEQKKSGMFSGGGIGVTIGSTSSRQQSRDSGTTQSQSASTIGSTGGDVTINA
ypseu0001X_3841  ------------------------------------------------------------
yrohd0001_38400  ------------------------------------------------------------
yfred0001_40710  ------------------------------------------------------------
yente0001X_8000  ------------------------------------------------------------
yruck0001_35040  ------------------------------------------------------------
                                                                             


                       2950      2960      2970      2980      2990      3000
                 =========+=========+=========+=========+=========+=========+
yberc0001_8630   GDQLTLHGSEVIAGKDIALKGADVAIIAAENSLSQQHTTESKQSGLTVALSGPV-GSAVN
ykris0001_26670  ------------------------------------------------------------
ykris0001_41280  ------------------------------------------------------------
yruck0001_910    ------------------------------------------------------------
yaldo0001_37900  ------------------------------------------------------------
yberc0001_34750  --------------------------------------------------AGIINEEQLN
yaldo0001_38900  ------------------------------------------------------------
yaldo0001_41000  ------------------------------------------------------------
ypseu0001X_3846  ------------------------------------------------------------
ymoll0001_35980  ------------------------------------------------------------
ypest0001X_8100  ------------------------------------------------------------
yfred0001_38200  ------------------------------------------------------------
ypest0001X_2754  ------------------------------------------------------------
yfred0001_34120  ------------------------------------------------------------
ypseu0001X_2842  ------------------------------------------------------------
ykris0001_7890   ------------------------------------------------------------
yruck0001_4640   ------------------------------------------------------------
ymoll0001_2720   ------------------------------------------------------------
yrohd0001_40310  ------------------------------------------------------------
yruck0001_34980  ------------------------------------------------------------
ypest0001X_2756  ------------------------------------------------------------
yfred0001_45620  ------------------------------------------------------------
yrohd0001_18860  GHQLTIHGSEVIANKDISLKGSDVAITAAENHLSQQHTTESKQSGITVALSGTV-GSAIN
yfred0001_33010  ------------------------------------------------------------
yfred0001_33210  ------------------------------------------------------------
yrohd0001_40300  ------------------------------------------------------------
yruck0001_4620   ------------------------------------------------------------
ypest0001X_8110  ------------------------------------------------------------
yberc0001_40130  ------------------------------------------------------------
yberc0001_40750  ------------------IAGKNVTILAAENQSSQTHSVEQKSSGLTLALSGAV-GSAMN
yruck0001_35050  ------------------------------------------------------------
yruck0001_13030  GVSIDIINDGYFQATQIEGGNNDVIVNAGN------------------------------
yinte0001_17980  DHESNAKVSQINARHDVIFDAANITLIGTEIGNEKAKVGNVQMMAGQKLILGASLSDSAN
yaldo0001_6040   ------------------------------------------------------------
yfred0001_33270  ------------------------------------------------------------
yrohd0001_18870  ------------------------------------------------------------
yruck0001_4610   ------------------------------------------------------------
ypest0001X_2758  ------------------------------------------------------------
ypseu0001X_2843  ------------------------------------------------------------
ypseu0001X_2846  GEDLRVQGAEVLAGKDINLTGKNVSILAAENQLTQSHTVEQKQSGLTLALSGAV-GSAVN
ypest0001X_2761  GEDLRVQGAEVLAGKDINLTGKNVSILAAENQLTQSHTVEQKQSGLTLALSGAV-GSAVN
yrohd0001_32190  ----------VLAGKDINLSGKNVSILAAENQSSQTYSVEQKSSGLTLALSGAV-GSA--
ymoll0001_21160  ------------------------------------------------------------
yrohd0001_32220  ------------------------------------------------------------
yfred0001_34080  ------------------------------------------------------------
yruck0001_4650   GEDLRVQGSDVLAGKDIHLTGKNVAIVAAENQLSQTHSVEQKQSGLTLALSGPV-GSAIN
yaldo0001_38920  ------------------------------------------------------------
yberc0001_20820  ------------------------------------------------------------
yaldo0001_3710   GEDLTVQGSEVLAGKDISLTGKNVAILAAENQSSQTHTVEQKQSGLTLALSGTV-GSAAN
ypest0001X_8090  ------------------------------------------------------------
yfred0001_34100  ------------------------------------------------------------
yruck0001_25350  KGGITADSKSFGGGLGGSIDNGSSSTSDAKVSQLTGQQGVELDSGRDLALKGSQIGSKEQ
yfred0001_38190  ------------------------------------------------------------
yfred0001_34090  ------------------------------------------------------------
yaldo0001_30990  ------------------------------------------------------------
yberc0001_36600  ------------------------------------------------------------
yrohd0001_40100  ------------------------------------------------------------
yrohd0001_40280  ------------------------------------------------------------
yruck0001_4660   ------------------------------------------------------------
ypseu0001X_3844  ------------------------------------------------------------
ykris0001_41250  ------------------------------------------------------------
ykris0001_21250  ------------------------------------------------------------
yruck0001_4630   ------------------------------------------------------------
yrohd0001_40320  ------------------------------------------------------------
yrohd0001_40080  ------------------------------------------------------------
yrohd0001_32210  ------------------------------------------------------------
yfred0001_45640  ------------------------------------------------------------
yfred0001_38220  ------------------------------------------------------------
yrohd0001_38410  ------------------------------------------------------------
yfred0001_33220  ------------------------------------------------------------
ymoll0001_36970  GEDLTIKGSDVLAGKDINLTGKNVAILAAENQSSQTHSVEQKSSGLTLALSGAV-GSAAN
yrohd0001_39710  ------------------------------------------------------------
yrohd0001_18880  ------------------------------------------------------------
yinte0001_5480   GNQLTIHGSEVIANKDISLQGSDVAITAAENNLSQQHTTESKQSGITVALSGAV-GSAVN
yfred0001_33200  ------------------------------------------------------------
yrohd0001_18890  ------------------------------------------------------------
yfred0001_32960  ------------------------------------------------------VGSALN
yberc0001_40160  -------------------------------------------------LQPNN------
ypseu0001X_3843  GDQLALHGSDVVAGKDLALTGADVAISAAENSRSQQYTAESKQSGLTVALSGPV-GSAVN
ypseu0001X_3837  ------------------------------------------------------------
ypest0001X_8140  ------------------------------------------------------------
ypseu0001X_3848  GNQLALHGSDVVAGKDLALTGADVAISAAENSRSQQYTAESKQSGLTVALSGPV-GSAVN
ypest0001X_8130  ------------------------------------------------------------
ypest0001X_8080  GNQLTLHGSDVVAGKDLALTGADVAISAAENSRSQQYTAESKQRGLTVALSGPV-GSAVN
yinte0001_41760  GGTAHIGGADILANKNLSVTGDSVIIEPGQDKRSSDQLYEQKSSGLTLALSGAV-GSALN
ykris0001_32060  GGTAHIGGADILANKNLNVTGDSVVIEPGQDKRSSDQLYEQKSSGLTLALSGAV-GSALN
ypseu0001X_3841  ------------------------------------------------------------
yrohd0001_38400  --------------------------LAAENQSSQTHTVEQKQSGLTLALSGTV-GSAVN
yfred0001_40710  -----------------------------------------------------V-GSALN
yente0001X_8000  ------------------------------------------------------------
yruck0001_35040  ------------------------------------------------------------
                                                                             


                       3010      3020      3030      3040      3050      3060
                 =========+=========+=========+=========+=========+=========+
yberc0001_8630   SAVTTAQQVTKETDGRLAALQGTKAALSG--VQAVQAGQLVQAQGGDTAS-MFGVSASLG
ykris0001_26670  ---------------------------------------------------MVGVSASLG
ykris0001_41280  ------------------------------------------------------------
yruck0001_910    ------------------------------------------------------------
yaldo0001_37900  -------------------LRAINNII---------------------------------
yberc0001_34750  SLDSGGRADFSMISGKDGNVTG---------TSAVHSGQSTSADSSATYNSQSSINQGLD
yaldo0001_38900  -------AEADSAIGYLAAVVGLVPGLGDAAGKALKAAETALKKGD--------------
yaldo0001_41000  ------------------------------------------------------------
ypseu0001X_3846  ---------------------GVKAGITGVVAKEIADKISEDDLDHLVTLKMMGNDEVTE
ymoll0001_35980  ------------------------------------------------------------
ypest0001X_8100  ------------------------------------------------------------
yfred0001_38200  ------------------------------------------------------------
ypest0001X_2754  ------------------------------------------------------------
yfred0001_34120  ------------------------------------------------------------
ypseu0001X_2842  ------------------------------------------------------------
ykris0001_7890   ------------------------------------------------------------
yruck0001_4640   ------------------------------------------------------------
ymoll0001_2720   -------------------------------LRLDSTGKVPPHTYADWVDGREGEFVLLN
yrohd0001_40310  ------------------------------------------------------------
yruck0001_34980  ------------------------------------------------------------
ypest0001X_2756  ------------------------------------------------------------
yfred0001_45620  ------------------------------------------------------------
yrohd0001_18860  SAVTTAQDAKKETNGRLQALQTTKAVLQG--VQAAQGKVLANETGDPNAV---GVSISLG
yfred0001_33010  -------------------LQTTKAVLQG--VQAAQGGVLANETGDPNAV---GVSISLG
yfred0001_33210  -------------------LQTTKAVLQG--VQAAQGKVLANETGDPNAV---GVSISLG
yrohd0001_40300  ------------------------------------------------------------
yruck0001_4620   ------------------------------------------------------------
ypest0001X_8110  ------------------------------------------------------------
yberc0001_40130  ----------------LMESEYQKAGASGAYNAGVEAGKLVSELIGTVAGGVSVAKVGTA
yberc0001_40750  TAVTTAKEASEESNGRLSALQGVKAALSG-------------------------------
yruck0001_35050  ------------------------------------------------------------
yruck0001_13030  ---------------QLNLDQSQYSEINGLSNAFGKGGLKLDSKAGANGGLIEGGGGMHR
yinte0001_17980  NGLKAGGNIRVGGKVSASVTDSSKMGFIG-------------------------------
yaldo0001_6040   ------------------------------------------------------------
yfred0001_33270  ------------------------------------------------------------
yrohd0001_18870  ------------------------------------------------------------
yruck0001_4610   ------------------------------------------------------------
ypest0001X_2758  ------------------------------------------------------------
ypseu0001X_2843  ------------------------------------------------------------
ypseu0001X_2846  TAVTTAKAASEESSGRLGALQGVKAALNG--VQAVQAGQLVQAEGGDAAS-MFGISASLG
ypest0001X_2761  TAVTTAKAASEESSGRLGALQGVKAALNG--VQAVQAGQLVQAEGGDAAS-MFGISASLG
yrohd0001_32190  -----AKDARDESNGRLAALQGVKAALSG--AQAVQAGQLVQAQGGDAAS-MFGVSASLG
ymoll0001_21160  ------------------------------------------------------------
yrohd0001_32220  ------------------------------------------------------------
yfred0001_34080  ------------------------------------------------------------
yruck0001_4650   TAVTTAKAASEESNGRLAALQGIKAALSG--VQAVQAGQLVQAEGGDTAS-MFGISASLG
yaldo0001_38920  ------------------------------------------------------------
yberc0001_20820  ------------------------------------------------------------
yaldo0001_3710   TAVTTAKAASEESNGRLAALQGVKAALSG--VQAVQAGQLVQAQGGDTAS-MFGISASLG
ypest0001X_8090  ------------------------------------------------------------
yfred0001_34100  ------------------------------------------------------------
yruck0001_25350  PTGDVSLKAAGKVDVQAAESSRSK-------DNTKLSGNINIGTNNTDSKTNSSGGFNLG
yfred0001_38190  ------------------------------------------------------------
yfred0001_34090  ------------------------------------------------------------
yaldo0001_30990  ----------------LAALQGVKAALSG--VQAVQGGQLAAVNASDQNA--IGVSLSYG
yberc0001_36600  ------------------------------------------------------------
yrohd0001_40100  ------------------------------------------------------------
yrohd0001_40280  ------------------------------------------------------------
yruck0001_4660   ------------------------------------------------------------
ypseu0001X_3844  ------------------------------------------------------------
ykris0001_41250  ------------------------------------------------------------
ykris0001_21250  ------------------------------------------------------------
yruck0001_4630   ------------------------------------------------------------
yrohd0001_40320  ------------------------------------------------------------
yrohd0001_40080  ------------------------------------------------------------
yrohd0001_32210  ------------------------------------------------------------
yfred0001_45640  ------------------------------------------------------------
yfred0001_38220  ------------------------------------------------------------
yrohd0001_38410  ------------------------------------------------------------
yfred0001_33220  ------------------------------------------------------------
ymoll0001_36970  TAVTTAKAASEESNGRLAALQGVKAALNG--AQAVQAGQLVQAQGGDAAS-MFGVSASLG
yrohd0001_39710  ------------------------------------------------------------
yrohd0001_18880  ------------------------------------------------------------
yinte0001_5480   TAVTTAQDAKKETNGRLAALQGTKAALSG--IQAVQAGQLAQAQGGDAAS-MVGISISLG
yfred0001_33200  ------------------------------------------------------------
yrohd0001_18890  ------------------------------------------------------------
yfred0001_32960  TAVTTAKAASEESNGRLSALQGVKAALSG--AQAVQAGQLVQAQGGDTAS-MFGISASLG
yberc0001_40160  ----------TGSSGHLQ-LFGI----------------------SDTAS-MFGVSASLG
ypseu0001X_3843  TAVTTAKAAREENTGRLAGLQGVKAALSG--VQAVQAGQLVQAQGGGVAE-MVGVSVSLG
ypseu0001X_3837  ------------------------------------------------------------
ypest0001X_8140  ------------------------------------------------------------
ypseu0001X_3848  TAVTTAKAAREENTGRLAGLQGVKAALSG--VQAVQAGQLVQAQGGGVTE-MVGVSVSLG
ypest0001X_8130  ------------------------------------------------------------
ypest0001X_8080  TAVTTAKAAREENTGRLAGLQGVKAALSG--VQAVQAGQLVQAQGGGITE-MVGVSVSLG
yinte0001_41760  TAVTTVQEAKDETNGRLAALKGTKAALTG--ISAKQAADLAQAQGNTDTGSLIGISVSLG
ykris0001_32060  TAVTTVQEAKDETNGRLAALKGTKAALTG--ISAKQAVDLAQAQGNSDTGSLFGVSVSLG
ypseu0001X_3841  ------------------------------------------------------------
yrohd0001_38400  TAVTTAKEAREESNGRLSALQGVKAALSG--VQAVQGGQLATLDASDQNA--IGVSLSYG
yfred0001_40710  TAVTTAKAASEESNGRLAALQGVKAALSG--MQAVQGGQLATLDARDQNA--IGVSLSYG
yente0001X_8000  ------------------------------------------------------------
yruck0001_35040  ------------------------------------------------------------
                                                                             


                       3070      3080      3090      3100      3110      3120
                 =========+=========+=========+=========+=========+=========+
yberc0001_8630   SQKSASQQHQEQTSVSGSTVTAGNNLSVTAT-----GEGNSANSGDIVVQGSQLKAGGDT
ykris0001_26670  SQKSSSQQHQEQTSVTGSTLTAGNNLTVTAT--------GDGNSGDILVQGSQLKAGGDT
ykris0001_41280  ------------------------------------------------VQGSQLKAGGDT
yruck0001_910    ------------------------------------------------------------
yaldo0001_37900  MLSDKINSKPEWLQEKGILRVPGNNKDIESI-----------------------------
yberc0001_34750  AGSAVSQRNQLSTKEGVRNLIDQ-------------SERNSPKSGQFINDLAHFSAESLT
yaldo0001_38900  ------------------------------------------------------------
yaldo0001_41000  ------------------------------------------------------------
ypseu0001X_3846  KYLNSLQDKYVPAHTGGDQIA---------------GLGPSNTGGDQIADNSPDHTGNDQ
ymoll0001_35980  ------------------------------------------------------------
ypest0001X_8100  ------------------------------------------------------------
yfred0001_38200  ------------------------------------------------------------
ypest0001X_2754  ------------------------------------------------------------
yfred0001_34120  ------------------------------------------------------------
ypseu0001X_2842  ------------------------------------------------------------
ykris0001_7890   ------------------------------------------------------------
yruck0001_4640   ------------------------------------------------------------
ymoll0001_2720   GQYKPHMTLTSQQRIRIWNMCSARYLNLSLP-----------------------------
yrohd0001_40310  ------------------------------------------------------------
yruck0001_34980  ------------------------------------------------------------
ypest0001X_2756  ------------------------------------------------------------
yfred0001_45620  ------------------------------------------------------------
yrohd0001_18860  SQKSKSESRLEQTTASGSNIAAGNNLSITAT--------GNHGAGDIRVQGSELQAGKNL
yfred0001_33010  SQKSKSESRLEQTTASGSNIAAGNNLSITAT-------GNHXLIN---------------
yfred0001_33210  SQKSKSESRLEQTTASGSNIAAGNNLSITATGNHXLIN----------------------
yrohd0001_40300  ------------------------------------------------------------
yruck0001_4620   ------------------------------------------------------------
ypest0001X_8110  ------------------------------------------------------------
yberc0001_40130  LTEKIAAKVVGKIDSPN-------------------------------------------
yberc0001_40750  ------------------------------------------------------------
yruck0001_35050  ------------------------------------------------------------
yruck0001_13030  QNKNANNAMKTRITTEGKVKLASGNGDLTLK---------------GVEIGSADAAVADV
yinte0001_17980  GDGNADKVNESRVVRKGMKIYSAGVLSLQAG---------SDDNQAIYTQGLQVDAKKVD
yaldo0001_6040   ------------------------------------------------------------
yfred0001_33270  ------------------------------------------------------------
yrohd0001_18870  ------------------------------------------------------------
yruck0001_4610   ------------------------------------------------------------
ypest0001X_2758  ------------------------------------------------------------
ypseu0001X_2843  ------------------------------------------------------------
ypseu0001X_2846  SQKSSSEQHQEQTHVTGSTLTAGNNLTINAT-----GEGNAANSGDIVVQGSQLQAGGDT
ypest0001X_2761  SQKSSSEQHQEQTHVTGSTLTAGNNLTINAT-----GEGNAANSGDIVVQGSQLQAGGDT
yrohd0001_32190  SQKSSSQQHQQQTSVTGSTLTAGNNLTINAT-----GDGHSANSGDIVVQGSQLKAGGDT
ymoll0001_21160  ------------------------------------------------------------
yrohd0001_32220  ------------------------------------------------------------
yfred0001_34080  ------------------------------------------------------------
yruck0001_4650   SQKSSSQQHQEQTSVAGSTLTAGNNLSVMAT-----GEGNSANSGDILIAGSQLKAGGDT
yaldo0001_38920  ------------------------------------------------------------
yberc0001_20820  ------------------------------------------------------------
yaldo0001_3710   SQKSASQQHQEQTNVTGSTLTAGNNLTINAT-----GDGSSANSGDIVVQGSQLKAGGDT
ypest0001X_8090  ------------------------------------------------------------
yfred0001_34100  ------------------------------------ANAYKGTDGNIVVYGSQLQSG---
yruck0001_25350  GDANFDKVHESATTHQGGRISSNGTLKIESG---------SEDKQAIHLQGTETSSKDTI
yfred0001_38190  ------------------------------------------------------------
yfred0001_34090  ------------------------------------------------------------
yaldo0001_30990  SQSSKLEQTVNQTTHQGSTLTAGNNLNITAT-----GNGVKGVDGDIVVQGSQLQAGKDT
yberc0001_36600  ------------------------------------------------------------
yrohd0001_40100  ------------------------------------------------------------
yrohd0001_40280  ------------------------------------------------------------
yruck0001_4660   ------------------------------------------------------------
ypseu0001X_3844  ------------------------------------------------------------
ykris0001_41250  ------------------------------------------------------------
ykris0001_21250  ------------------------------------------------------------
yruck0001_4630   ------------------------------------------------------------
yrohd0001_40320  ------------------------------------------------------------
yrohd0001_40080  ------------------------------------------------------------
yrohd0001_32210  ------------------------------------------------------------
yfred0001_45640  ------------------------------------------------------------
yfred0001_38220  ------------------------------------------------------------
yrohd0001_38410  ------------------------------------------------------------
yfred0001_33220  ------------------------------------------------------------
ymoll0001_36970  SQKSSSQQHQEQTSVTGSTLTAGNNLTVTAT-----GEGNSANSGDIVVQGSQLKAGGDT
yrohd0001_39710  ------------------------------------------------------------
yrohd0001_18880  ------------------------------------------------------------
yinte0001_5480   SQKSSSQQHQEQTRVSGSTLTAGNNLTINAT-----GKGSSANSGDILIAGSQLKAGGDT
yfred0001_33200  ------------------------------------------------------------
yrohd0001_18890  ------------------------------------------------------------
yfred0001_32960  SQKSASQQHQEQTSVTGSTLTAGNNLTINAT-----GDGNPANSGDIVVQGSQLKAGGDT
yberc0001_40160  SQKSASQQHQEQTSVSGSTVTAGNNLTINAT-----GQGGAASSGDILIAGSQLKAGGDT
ypseu0001X_3843  SQKSSSQQQQEQTQVSGSALTAGNNLSIKAT-----GGGNAANSGDILIAGSQLKAGGDT
ypseu0001X_3837  ------------------------------------------------------------
ypest0001X_8140  ------------------------------------------------------------
ypseu0001X_3848  SQKSSSQQQQEQTQVSGSALTAGNNLSIKAT-----GGGNAANSGDILIAGSQLKAGGDT
ypest0001X_8130  ------------------------------------------------------------
ypest0001X_8080  SQKSSSQQQQEQTQVSGSALTAGNNLSIKAS-----G-------SDILIAGSQLKAGGDT
yinte0001_41760  SQKSTSQQHQEQQAVSGSTLTAGNDLNITAT-----GKGQLANSGDIIIGGSQLQAGHDT
ykris0001_32060  SQKSTSQQHQEQQAVSGSTLTAGNDLNITAT-----GKGQSTNSGDIIIGGSQLKAGHDT
ypseu0001X_3841  ------------------------------------------------------------
yrohd0001_38400  SQSSKSEQTVNQTTHQGSTLTAGNNLNITAT-----GNGVKGQDGDIVVQGSQLQAGKDA
yfred0001_40710  SQSSKSEQTVNQTTHQGSTLTAGNNLNITAT-----GNGVKGQDGDIVVQGSQLQAGKDA
yente0001X_8000  ------------------------------------------PAGDIVVQGSQLQAGKDT
yruck0001_35040  ------------------------------------------------------------
                                                                             


                       3130      3140      3150      3160      3170      3180
                 =========+=========+=========+=========+=========+=========+
yberc0001_8630   TLDAARDLLLLGAANTQXLIN---------------------------------------
ykris0001_26670  TLDAARDLLLLGAANTQKTDGSNSSSGGSVGVSLGLNGASSGLSIFANANKGQGNEHG--
ykris0001_41280  TLDAARDLLLLGAANTQKTDGSNSSSGGSVGGSLGLNGTSSGLSIFANANKGQGNEHG--
yruck0001_910    ------------------------------------------------------------
yaldo0001_37900  ------------------------------------------------------------
yberc0001_34750  PFTNSTKMDSVHKSASTSLTG---------------------------------------
yaldo0001_38900  ------------------------------------------------------------
yaldo0001_41000  ------------------------------------------------------------
ypseu0001X_3846  STGQ-------GATNTGNIDGKPDTGGNSTVTPIPDGPNKDDLAYLQSGNKIE-------
ymoll0001_35980  ------------------------------------------------------------
ypest0001X_8100  ------------------------------------------------------------
yfred0001_38200  ------------------------------------------------------------
ypest0001X_2754  ------------------------------------------------------------
yfred0001_34120  ------------------------------------------------------------
ypseu0001X_2842  ------------------------------------------------------------
ykris0001_7890   ------------------------------------------------------------
yruck0001_4640   ------------------------------------------------------------
ymoll0001_2720   ------------------------------------------------------------
yrohd0001_40310  ------------------------------------------------------------
yruck0001_34980  ------------------------------------------------------------
ypest0001X_2756  ------------------------------------------------------------
yfred0001_45620  ------------------------------------------------VNASKGKEQG--
yrohd0001_18860  SLDAKNDIALNSAENSESLRGSNKSSGGNIGIGIGVGK-GAGISIFAGVNASKGKEQG--
yfred0001_33010  ------------------------------------------------------------
yfred0001_33210  ------------------------------------------------------------
yrohd0001_40300  ------------------------------------------------------------
yruck0001_4620   ------------------------------------------------------------
ypest0001X_8110  ------------------------------------------------------------
yberc0001_40130  ------------------------------------------------------------
yberc0001_40750  ------------------------------------------------------------
yruck0001_35050  ------------------------------------------------------------
yruck0001_13030  ELISGGKVKLLASVSDSATQASKQGGSVLLGMTKSSSGDKNTTSGAIGVTAEENIINE--
yinte0001_17980  VSANKGGIFMESSLTILPKDNWNFDINADLVLTSKFNKDSDGLIDKSSGSKSHYTGAGIK
yaldo0001_6040   ------------------------------------------------------------
yfred0001_33270  ------------------------------------------------------------
yrohd0001_18870  ------------------------------------------------------------
yruck0001_4610   ------------------------------------------------------------
ypest0001X_2758  ------------------------------------------------------------
ypseu0001X_2843  ------------------------------------------------------------
ypseu0001X_2846  TLDAARDVLLLGAANTQKTDGSNSSSGGSVGVSLGISGASSGLSIFANANKGQGSEHG--
ypest0001X_2761  TLDAARDVLLLGAANTQKTDGSNSSSGGSVGVSLGISGASSGLSIFANANKGQGSEHG--
yrohd0001_32190  TLDAARDLLLLGAANTQKTDGSNSSSGGSVGVSLGLGGSGGGLSIFANANKGQGSEHG--
ymoll0001_21160  ------------------------------------------------------------
yrohd0001_32220  ------------------------------------------------------------
yfred0001_34080  ------------------------------------------------------------
yruck0001_4650   TLDAARDVWLLGAANTQKTDGSNRSSGGSVGVSLGMGGSGSGLSVFANANKGQGRERG--
yaldo0001_38920  ------------------------------------------------------------
yberc0001_20820  ------------------------------------------------------------
yaldo0001_3710   TLDAARDVLLLGAANTQKTDGSNSSSGGSVGVSLGFGSAGGGLSIFANANKGQGNEHG--
ypest0001X_8090  ---------------------------------------EYGLSLFVSSNKSQGNDRG--
yfred0001_34100  ----------------------------------------------------KGSESG--
yruck0001_25350  LDAKKGGVVLESVQNDEKKNNWNLGLKGNVGISQSYNKDDKGVVDSNSGEDSHAVSAGLN
yfred0001_38190  ------------------------------------------------------------
yfred0001_34090  ------------------------------------------------------------
yaldo0001_30990  SLTANRDVILQSTQESQTLDGSNKSSGGSLGIGIGAGQGGWGINIS--------------
yberc0001_36600  ------------------------------------------------------------
yrohd0001_40100  ------------------------------------------------------------
yrohd0001_40280  ------------------------------------------------------------
yruck0001_4660   ------------------------------------------------------------
ypseu0001X_3844  -------VQLLGAANRQKTDGSNSSRGGSVGVSVG----GSGLSVFANANKGQGNERG--
ykris0001_41250  ------------------------------------------------------------
ykris0001_21250  ------------------------------------------------------------
yruck0001_4630   ------------------------------------------------------------
yrohd0001_40320  ------------------------------------------------------------
yrohd0001_40080  ------------------------------------------------------------
yrohd0001_32210  ------------------------------------------------------------
yfred0001_45640  ------------------------------------------------------------
yfred0001_38220  ------------------------------------------------------------
yrohd0001_38410  ------------------------------------------------------------
yfred0001_33220  ------------------------------------------------------------
ymoll0001_36970  TLDAARDLLLLSAANTQKTDGSNSSSGAAIGVSLGLSGTGSGLXXN--------------
yrohd0001_39710  ------------------------------------------------------------
yrohd0001_18880  ------------------------------------------------------------
yinte0001_5480   TLDAARDLLLLGAANTQKTDGSNNSSGGNIGVSLGVGSSGGGLSIFANANKSQGKEHG--
yfred0001_33200  ------------------------------------------------------------
yrohd0001_18890  ------------------------------------------------------------
yfred0001_32960  TLDAARDLLLLGAA----------------------------------------------
yberc0001_40160  TLDAARDLLLLGAANTXXN-----------------------------------------
ypseu0001X_3843  RLDAARDVRLLGAANRQKTDGSNSSRGGSVGVSVG----GSGLSVFANANKGQGNERG--
ypseu0001X_3837  ------------------------------------------------------------
ypest0001X_8140  ------------------------------------------------------------
ypseu0001X_3848  RLDAARDVRLLGAANRQKTDGSNSSRGGSVGVSVG----GSGLSVFANANKGQGNERG--
ypest0001X_8130  ------------------------------------------------------------
ypest0001X_8080  RLDAARDVQLLGAANRQKTDGSNSSRGGSVGVSVG----GSGLSVFANANKGQGNERG--
yinte0001_41760  TLDADRDLLLLGAANTQKSEGSNSSSGGNIGASISFGD-KIGVSVFANANKSKGNDSG--
ykris0001_32060  TLDADRDLFLLGAANTQKTEGSNSSSGGSIGASISLGK-ESGLSIFANANKSKGNDSG--
ypseu0001X_3841  ------------------------------------------------------------
yrohd0001_38400  SLTANRDVILQSTQESQTLDGSNSSSGGSLGIGIGAGQGGWGINISASLNQGKGSESG--
yfred0001_40710  SLTANRDVILQSTQESQTLDGSNSSSGGSLGVGIGAGQGGWGINVSASLNKGKGSESG--
yente0001X_8000  RLTANRDVILQSTQESQTLDGNNSSSGGSLGVGIGAGQGGWGINISASLNKGKGSESG--
yruck0001_35040  ------------------------------------------------------------
                                                                             


                       3190      3200      3210      3220      3230      3240
                 =========+=========+=========+=========+=========+=========+
yberc0001_8630   ------------------------------------------------------------
ykris0001_26670  -----NGTSWTETTLDSGGTLSLNSGRDTSLXXN--------------------------
ykris0001_41280  -----NGTTWTETTIDSGGTLSLNSGRDTSLXXN--------------------------
yruck0001_910    ------------------------------------------------------------
yaldo0001_37900  ------------------------------------------------------------
yberc0001_34750  ------------------------------------------------------------
yaldo0001_38900  ------------------------------------------------------------
yaldo0001_41000  ------------------------------------------------------------
ypseu0001X_3846  ------------------------------------------------------------
ymoll0001_35980  ------------------------------------------------------------
ypest0001X_8100  ------------------------------------------------------------
yfred0001_38200  ------------------------------------------------------------
ypest0001X_2754  ------------------------------------------------------------
yfred0001_34120  ------------------------------------------------------------
ypseu0001X_2842  ------------------------------------------------------------
ykris0001_7890   ------------------------------------------------------------
yruck0001_4640   ------------------------------------------------------------
ymoll0001_2720   ------------------------------------------------------------
yrohd0001_40310  ------------------------------------------------------------
yruck0001_34980  ------------------------------------------------------------
ypest0001X_2756  ------------------------------------------------------------
yfred0001_45620  -----DSLTHTETQLKAGDTVSITSGRDTSLKGAQVSGETVKVDAG---R-------NLT
yrohd0001_18860  -----DSLTHTETQLKAGNTVSITSGRDTKLQGAQVSGETVKVDVG---R-------DLT
yfred0001_33010  ------------------------------------------------------------
yfred0001_33210  ------------------------------------------------------------
yrohd0001_40300  ------------------------------------------------------------
yruck0001_4620   ------------------------------------------------------------
ypest0001X_8110  ------------------------------------------------------------
yberc0001_40130  ------------------------------------------------------------
yberc0001_40750  ------------------------------------------------------------
yruck0001_35050  ------------------------------------------------------------
yruck0001_13030  -----STLFSQGSYINSRDNIIISAGSYDEKSIYTQGLEAIAPYIS--------------
yinte0001_17980  VGIDKQDIVEHQNTHIKTDTFTLKSHKDTRMVGADVSTDIALVDVG------------GD
yaldo0001_6040   ------------------------------------------------------------
yfred0001_33270  ------------------------------------------------------------
yrohd0001_18870  ------------------------------------------------------------
yruck0001_4610   ------------------------------------------------------------
ypest0001X_2758  ------------------------------------------VEVG---R-------DLL
ypseu0001X_2843  ------------------------------------------VEVG---R-------DLL
ypseu0001X_2846  -----DGISWTETTLDSGGTLSVHSGRDTSLVGAQVSGETVKVEVG---R-------DLL
ypest0001X_2761  -----DGTSWTETTLDSGGTLSLYSGRDTSLVGAQVSGETVKVEVG---R-------DLL
yrohd0001_32190  -----DGTFWTETTVDSGGTLSLNSGRDTSLIGAQASGDTVKVDVG---R-------DLL
ymoll0001_21160  -------------------------------VGAQASGETVKVDVG---R-------DLL
yrohd0001_32220  ------------------------------------------VDVG---R-------DLL
yfred0001_34080  -------------------------------VGAQASGETVKVDVG---R-------DLL
yruck0001_4650   -----DGTFWSETQVDSGGTLSLHSGRDTSLIGAQASGESIKADVG---R-------DLL
yaldo0001_38920  ------------------------------------------------------------
yberc0001_20820  ------------------------------------------VDVG---R-------DLL
yaldo0001_3710   -----DGTFWTETQIDTGGTLSLSSGRDTSLIGAQASGETVKVDVG---R-------DLL
ypest0001X_8090  -----DGTFWTETQLNSGGTLSLKSGRDLL------------------------------
yfred0001_34100  -----NGLTHTETTVNAGNQLNIISGRDTVLQGAQVSGETVKADVG---R-------NLL
yruck0001_25350  IGVEKQDATKQQNTKINTGNLTLNSGKDTTLAGAGITADKVTGDIGGDLRVESRKDVETG
yfred0001_38190  ------------------------------------------------------------
yfred0001_34090  ------------------------------------------------------------
yaldo0001_30990  ------------------------------------------------------------
yberc0001_36600  ------------------------------------------------------------
yrohd0001_40100  ------------------------------------------------------------
yrohd0001_40280  ------------------------------------------------------------
yruck0001_4660   ------------------------------------------------------------
ypseu0001X_3844  -----DGTFWTETTVDSGGMFSLRSGRDTALTGAQVSAETVKADVG---R-------NLT
ykris0001_41250  ------------------------------------------------------------
ykris0001_21250  ------------------------------------------------------------
yruck0001_4630   ------------------------------------------------------------
yrohd0001_40320  ------------------------------------------------------------
yrohd0001_40080  ------------------------------------------------------------
yrohd0001_32210  ------------------------------------------------------------
yfred0001_45640  ------------------------------------------------------------
yfred0001_38220  ------------------------------------------------------------
yrohd0001_38410  ------------------------------------------------------------
yfred0001_33220  ------------------------------------------------------------
ymoll0001_36970  ------------------------------------------------------------
yrohd0001_39710  ------------------------------------------------------------
yrohd0001_18880  ------------------------------------------------------------
yinte0001_5480   -----DGTFWSETQVDTGGTLSLHSGRDTSLIGAQASGESVKADVG---R-------DLL
yfred0001_33200  ------------------------------------------------------------
yrohd0001_18890  ------------------------------------------------------------
yfred0001_32960  ------------------------------------------------------------
yberc0001_40160  ------------------------------------------------------------
ypseu0001X_3843  -----DGTFWTETTVDSGGMFSLRSGRDTALTGAQVSAETVKADVG---R-------NLT
ypseu0001X_3837  ------------------------------------------------------------
ypest0001X_8140  ------------------------------------------------------------
ypseu0001X_3848  -----DGTFWTETTVDSGGMFSLRSGRDTALTGAQVSAETVKADVG---R-------NLT
ypest0001X_8130  ------------------------------------------------------------
ypest0001X_8080  -----DGTFWTETTVDSGGMFSLRSGRDTALTGAQVSAETVKADVG---R-------NLT
yinte0001_41760  -----DGTYWSETTLDSGNTLSLTSGRDTTLQGAQLSGDKVEADVG---R-------NLA
ykris0001_32060  -----NGTSWSETTLDSGNTLSLTSGRDTTLLGAQLSGDKVEADVG---R-------NLT
ypseu0001X_3841  ------------------------------------------------------------
yrohd0001_38400  -----NGVSHTETTVNAGNTLNITSGRDTVLQGAQVSGDTVKADVG---R-------DLL
yfred0001_40710  -----NGVSYTETTVNAGNQLNITSGRDTVLQGAQVSGETVKADVG---R-------DLL
yente0001X_8000  -----NGVTHTETTVNAGNQLNITSGRDTVLQGAQVSGETVKADVG---R-------DLL
yruck0001_35040  ------------------------------------------------------------
                                                                             


                       3250      3260      3270      3280      3290      3300
                 =========+=========+=========+=========+=========+=========+
yberc0001_8630   ------------------------------------------------------------
ykris0001_26670  ------------------------------------------------------------
ykris0001_41280  ------------------------------------------------------------
yruck0001_910    ---------------------NCIEKKQNILPNHFNNAVNDLKKLHNKHTYLRDIKPGNM
yaldo0001_37900  --------------------AKVMNEGSDQEIKNILMKNDSHHDIFGLIKNQFGLA----
yberc0001_34750  ---------------------GTPGSGILGFGASATATVEGSTTDQDHYNQRAGQFKHQL
yaldo0001_38900  ---------------------------IGEASRLINQASDELTAATRPSHRQSEIDVGEG
yaldo0001_41000  ---------------------AVKEFGSDVVTKCLSGSDCPMAAQVAILAIQAMMGQGEN
ypseu0001X_3846  ------------------------------------------------------------
ymoll0001_35980  ------------------------------------------------------------
ypest0001X_8100  ---------------------XSMVVVGSGGSANLSMSRDQLRSNFDSVQEQTEKNAVEN
yfred0001_38200  ------------------------------------------------------------
ypest0001X_2754  -------------------------------------------------------LPPLR
yfred0001_34120  ------------------------------SGKELDMDDLYLEKDYSIVDSIQGSI----
ypseu0001X_2842  ------------------------------------------------------------
ykris0001_7890   ------------------------------------------------------------
yruck0001_4640   ------------------------------------------------------------
ymoll0001_2720   -----------------GCQLIQVGSDGGLMEKALASQHDILLSPGERMEVLVTGESGEY
yrohd0001_40310  ------------------------------------------------------------
yruck0001_34980  ------------------------------------------------------------
ypest0001X_2756  ------------------------------------------------------------
yfred0001_45620  LQSEQDRNNYDSKQTSVSAS-GSFTFGTMTGSGSVSASKSKIDSDYTSVQEQTGFFXXN-
yrohd0001_18860  LQSEQDRNNYDSKQTSVSAS-GSFTFGTMTGSGSVSASKSKIDSDYTSVQEQTGFFAGKG
yfred0001_33010  ------------------------------------------------------------
yfred0001_33210  ------------------------------------------------------------
yrohd0001_40300  ------------------------------------------------------------
yruck0001_4620   ------------------------------------------------------------
ypest0001X_8110  --------------LPAIQGKIAPAFGEPGGGIQIL------------------------
yberc0001_40130  ---------------------LKTPSGNPNSSSAITDVEAGGYSYYDQFKNANGGWDWPK
yberc0001_40750  ------------------------------------------------------------
yruck0001_35050  ------------------------------------------------------------
yruck0001_13030  MTAENGGIFMESARSSSPKDNKNLYIGISANGEKTSGSNSNSINGYDVTGGSFNLISANE
yinte0001_17980  LHIESQKDKQDITKVLVNFALSHTNDKSSSVVSNISKIGTKRFEGQIKDILTRGINKTGA
yaldo0001_6040   ------------------------------------------------------------
yfred0001_33270  ------------------------------------------------------------
yrohd0001_18870  ------------------------------------------------------------
yruck0001_4610   ------------------------------------------------------------
ypest0001X_2758  LQSQQDSDNYDAKQQSSSVG-GSFSPGSMTGSISINGSQDKLNSNFDSVQEQTGIFAGSG
ypseu0001X_2843  LQSQQDSDNYDAKQQNSSVG-GSFSPGSMTGSISINGSQDKLNSNFDSVQEQTGIFAGSG
ypseu0001X_2846  LQSQQDSDNYDAKQQSSSVG-GSFSPGSMTGSISINGSQDKLNSNFDSVQEQTGIFAGSG
ypest0001X_2761  LQSQQDSDNYDAKQQNSSVG-GSFSPGSMTGSISINGSQDKLHSNFDSVQEQTGIFAGSG
yrohd0001_32190  LQSQQDSDYYDSKQTSTSGG-VSVAVIGSGSSANLSMSQDKLHSNYDSVQEQTGIFAGKG
ymoll0001_21160  LQSQQDSDNYDAKQTSISGG-ISVPIAGGX------------------------------
yrohd0001_32220  LQSQQDSDNYDSKQTSTSGG-VSVAVTGG-GSANLSMSRDKLHSNYXLIN----------
yfred0001_34080  LQSQQDSDNYDSKQTSTSGG-VSVAVVGGGGSANLSMSQDKLHSNYDSVQEQTGIFAGKG
[truncated: 91,739 more chars]
