# Supplementary material for: Genomic characterization of the Yersinia genus
Source: Genome Biol. 2010 Jan 4;11(1):R1. doi: 10.1186/gb-2010-11-1-r1 (PMC2847712; doi:10.1186/gb-2010-11-1-r1)
Supplement: Additional file 15 — The top level directory consists of a directory called Additional_cluster_files and 5010 directories, one for each multi-protein cluster family. (This top level directory has been split into three data files for uploading purposes (Additional files 15, 16, 17).) Within the directory are the following files: PGL1_unique_Yersinia_unclustered.out - list of all protein singletons that MCL did not group into a cluster (see Materials and Methods); PGL1_Yersinia_unique_locus_tags.txt - names of the 11 locus tag prefixes used for each genome; PGL1_unique_Yersinia.gff - mapping each Yersinia protein to a cluster in tab delimited GFF; PGL1_unique_Yersinia.sigfile - list of the longest protein in each cluster; PGL1_unique_Yersinia.summary - summary table of features of each of the clusters; PGL1_unique_Yersinia.table - summary table of each protein in the clusters. Within each cluster directory are the following files, where 'x' is the cluster name: PGL1_unique_Yersinia-x.faa - multifasta file of the proteins in the cluster; PGL1_unique_Yersinia-x.summary - summary of the properties of the proteins; PGL1_unique_Yersinia-x.matches - blast matches between the proteins of the cluster; PGL1_unique_Yersinia-x.muscle.fasta - muscle alignment of the proteins; PGL1_unique_Yersinia-x.muscle.fasta.gblo - gblocks output of muscle alignment (that is, auto-trimmed alignment); PGL1_unique_Yersinia-x.muscle.fasta.gblo.htm - as above in html format; PGL1_unique_Yersinia-x.muscle.tree - treefile from muscle alignment; PGL1_unique_Yersinia-x.sif - matches between proteins in simple interaction format for display on graphing software. [file gb-2010-11-1-r1-S15.zip › clusters/PGL1_unique_yersinia-CL10/PGL1_unique_yersinia-CL10.muscle.fasta.gblo.htm]

PGL1\_unique\_yersinia-CL10.muscle.fasta


## Gblocks 0.91b Results

Processed file: **PGL1\_unique\_yersinia-CL10.muscle.fasta**  
Number of sequences: **57**  
Alignment assumed to be: **Protein**  
New number of positions: **317** (selected positions are underlined in blue)

```
                         10        20        30        40        50        60
                 =========+=========+=========+=========+=========+=========+
ypseu0001X_140   MRLFGYARVSTSQQSLDIQIKTL-KDAGVKANRI------------FTDKASGSSADREG
ypest0001X_830   MRLFGYARVSTSQQSLDIQIKTL-KDAGVKANRI------------FTDKASGSSADREG
yente0001X_2473  MEKPQTIVGFKIGNAMVDELDIL--PVAKARLAWRKIIARMRYKLKGVRNGIDVTTTPEG
ykris0001_3040   ----------------------LAQETFIRALTV--------------PLPI--------
ypest0001X_3403  MVTFETVMEIKILHKQGMSSRAIARELGISRNTVKRYLQAKSEPPKYTPRPAVASLLDEY
ypest0001X_2225  MVTFETVMEIKILHKQGMSSRAIARELGISRNTVKRYLQAKSEPPKYTPRPAVASLLDEY
ypest0001X_2950  MVTFETVMEIKILHKQGMSSRAIARELGISRNTVKRYLQAKSEPPKYTPRPAVASLLDEY
ypseu0001X_4293  -------MEIKILHKQGMSSRAIARELGISRNTVKRYLQAKSEPPKYTPRPAVASLLDEY
ypseu0001X_2061  -------MEIKILHKQGMSSRAIARELGISRNTVKRYLQAKSEPPKYTPRPAVASLLDEY
ypseu0001X_1809  -------MEIKILHKQGMSSRAIARELGISRNTVKRYLQAKSEPPKYTPRPAVASLLDEY
ypseu0001X_1304  -------MEIKILHKQGMSSRAIARELGISRNTVKRYLQAKSEPPKYTPRPAVASLLDEY
ypseu0001X_4150  -------MEIKILHKQGMSSRAIARELGISRNTVKRYLQAKSEPPKYTPRPAVASLLDEY
ypest0001X_4315  MVTFETVMEIKILHKQGMSSRAIARELGISRNTVKRYLQAKSEPPKYTPRPAVASLLDEY
ypest0001X_4080  MVTFETVMEIKILHKQGMSSRAIARELGISRNTVKRYLQAKSEPPKYTPRPAVASLLDEY
ypest0001X_4060  MVTFETVMEIKILHKQGMSSRAIARELGISRNTVKRYLQAKSEPPKYTPRPAVASLLDEY
ypest0001X_4005  MVTFETVMEIKILHKQGMSSRAIARELGISRNTVKRYLQAKSEPPKYTPRPAVASLLDEY
ypest0001X_3885  MVTFETVMEIKILHKQGMSSRAIARELGISRNTVKRYLQAKSEPPKYTPRPAVASLLDEY
ypest0001X_3836  MVTFETVMEIKILHKQGMSSRAIARELGISRNTVKRYLQAKSEPPKYTPRPAVASLLDEY
ypest0001X_3727  MVTFETVMEIKILHKQGMSSRAIARELGISRNTVKRYLQAKSEPPKYTPRPAVASLLDEY
ypest0001X_3562  MVTFETVMEIKILHKQGMSSRAIARELGISRNTVKRYLQAKSEPPKYTPRPAVASLLDEY
ypest0001X_3528  MVTFETVMEIKILHKQGMSSRAIARELGISRNTVKRYLQAKSEPPKYTPRPAVASLLDEY
ypest0001X_3487  MVTFETVMEIKILHKQGMSSRAIARELGISRNTVKRYLQAKSEPPKYTPRPAVASLLDEY
ypest0001X_3212  MVTFETVMEIKILHKQGMSSRAIARELGISRNTVKRYLQAKSEPPKYTPRPAVASLLDEY
ypest0001X_3093  MVTFETVMEIKILHKQGMSSRAIARELGISRNTVKRYLQAKSEPPKYTPRPAVASLLDEY
ypest0001X_3005  MVTFETVMEIKILHKQGMSSRAIARELGISRNTVKRYLQAKSEPPKYTPRPAVASLLDEY
ypest0001X_2922  MVTFETVMEIKILHKQGMSSRAIARELGISRNTVKRYLQAKSEPPKYTPRPAVASLLDEY
ypest0001X_2788  MVTFETVMEIKILHKQGMSSRAIARELGISRNTVKRYLQAKSEPPKYTPRPAVASLLDEY
ypest0001X_2665  MVTFETVMEIKILHKQGMSSRAIARELGISRNTVKRYLQAKSEPPKYTPRPAVASLLDEY
ypest0001X_2634  MVTFETVMEIKILHKQGMSSRAIARELGISRNTVKRYLQAKSEPPKYTPRPAVASLLDEY
ypest0001X_2441  MVTFETVMEIKILHKQGMSSRAIARELGISRNTVKRYLQAKSEPPKYTPRPAVASLLDEY
ypest0001X_2350  MVTFETVMEIKILHKQGMSSRAIARELGISRNTVKRYLQAKSEPPKYTPRPAVASLLDEY
ypest0001X_2288  MVTFETVMEIKILHKQGMSSRAIARELGISRNTVKRYLQAKSEPPKYTPRPAVASLLDEY
ypest0001X_2153  MVTFETVMEIKILHKQGMSSRAIARELGISRNTVKRYLQAKSEPPKYTPRPAVASLLDEY
ypest0001X_1996  MVTFETVMEIKILHKQGMSSRAIARELGISRNTVKRYLQAKSEPPKYTPRPAVASLLDEY
ypest0001X_1975  MVTFETVMEIKILHKQGMSSRAIARELGISRNTVKRYLQAKSEPPKYTPRPAVASLLDEY
ypest0001X_1936  MVTFETVMEIKILHKQGMSSRAIARELGISRNTVKRYLQAKSEPPKYTPRPAVASLLDEY
ypest0001X_1862  MVTFETVMEIKILHKQGMSSRAIARELGISRNTVKRYLQAKSEPPKYTPRPAVASLLDEY
ypest0001X_1756  MVTFETVMEIKILHKQGMSSRAIARELGISRNTVKRYLQAKSEPPKYTPRPAVASLLDEY
ypest0001X_1659  MVTFETVMEIKILHKQGMSSRAIARELGISRNTVKRYLQAKSEPPKYTPRPAVASLLDEY
ypest0001X_1562  MVTFETVMEIKILHKQGMSSRAIARELGISRNTVKRYLQAKSEPPKYTPRPAVASLLDEY
ypest0001X_1343  MVTFETVMEIKILHKQGMSSRAIARELGISRNTVKRYLQAKSEPPKYTPRPAVASLLDEY
ypest0001X_1298  MVTFETVMEIKILHKQGMSSRAIARELGISRNTVKRYLQAKSEPPKYTPRPAVASLLDEY
ypest0001X_1222  MVTFETVMEIKILHKQGMSSRAIARELGISRNTVKRYLQAKSEPPKYTPRPAVASLLDEY
ypest0001X_1127  MVTFETVMEIKILHKQGMSSRAIARELGISRNTVKRYLQAKSEPPKYTPRPAVASLLDEY
ypest0001X_1028  MVTFETVMEIKILHKQGMSSRAIARELGISRNTVKRYLQAKSEPPKYTPRPAVASLLDEY
ypest0001X_9850  MVTFETVMEIKILHKQGMSSRAIARELGISRNTVKRYLQAKSEPPKYTPRPAVASLLDEY
ypest0001X_8440  MVTFETVMEIKILHKQGMSSRAIARELGISRNTVKRYLQAKSEPPKYTPRPAVASLLDEY
ypest0001X_7760  MVTFETVMEIKILHKQGMSSRAIARELGISRNTVKRYLQAKSEPPKYTPRPAVASLLDEY
ypest0001X_7380  MVTFETVMEIKILHKQGMSSRAIARELGISRNTVKRYLQAKSEPPKYTPRPAVASLLDEY
ypest0001X_5890  MVTFETVMEIKILHKQGMSSRAIARELGISRNTVKRYLQAKSEPPKYTPRPAVASLLDEY
ypest0001X_4460  MVTFETVMEIKILHKQGMSSRAIARELGISRNTVKRYLQAKSEPPKYTPRPAVASLLDEY
ypest0001X_3710  MVTFETVMEIKILHKQGMSSRAIARELGISRNTVKRYLQAKSEPPKYTPRPAVASLLDEY
ypest0001X_2310  MVTFETVMEIKILHKQGMSSRAIARELGISRNTVKRYLQAKSEPPKYTPRPAVASLLDEY
ypest0001X_1670  MVTFETVMEIKILHKQGMSSRAIARELGISRNTVKRYLQAKSEPPKYTPRPAVASLLDEY
ypest0001X_1000  MVTFETVMEIKILHKQGMSSRAIARELGISRNTVKRYLQAKSEPPKYTPRPAVASLLDEY
ypest0001X_890   MVTFETVMEIKILHKQGMSSRAIARELGISRNTVKRYLQAKSEPPKYTPRPAVASLLDEY
ypest0001X_10    MVTFETVMEIKILHKQGMSSRAIARELGISRNTVKRYLQAKSEPPKYTPRPAVASLLDEY
                 ############################################################


                         70        80        90       100       110       120
                 =========+=========+=========+=========+=========+=========+
ypseu0001X_140   LDLLRMKVEEGD------VILVKKLDRLG-RDTVDMIQ-LIKEF---DAQGVSIQFI-DD
ypest0001X_830   LDLLRMKVEEGD------VILVKKLDRLG-RDTVDMIQ-LIKEF---DAQGVSIQFI-DD
yente0001X_2473  FKFVYQQFVKA----------VRDKPELATLYGLVQASTFDNEANLPDDYIPSL-LESYP
ykris0001_3040   ------------------------------RSHRSLLYRIARNLLIDHYRRPDTHIHEPS
ypest0001X_3403  RDYIRQRIADAHPYKIPATVIAREIRDQGYRGGMTILRAFIRSLSVPQEQEPAVRFETEP
ypest0001X_2225  RDYIRQRIADAHPYKIPATVIAREIRDQGYRGGMTILRAFIRSLSVPQEQEPAVRFETEP
ypest0001X_2950  RDYIRQRIADAHPYKIPATVIAREIRDQGYRGGMTILRAFIRSLSVPQEQEPAVRFETEP
ypseu0001X_4293  RDYIRQRIADAHPYKIPATVIAREIRDQGYRGGMTILRAFIRSLSVPQEQEPAVRFETEP
ypseu0001X_2061  RDYIRQRIADAHPYKIPATVIAREIRDQGYRGGMTILRAFIRSLSVPQEQEPAVRFETEP
ypseu0001X_1809  RDYIRQRIADAHPYKIPATVIAREIRDQGYRGGMTILRAFIRSLSVPQEQEPAVRFETEP
ypseu0001X_1304  RDYIRQRIADAHPYKIPATVIAREIRDQGYRGGMTILRAFIRSLSVPQEQEPAVRFETEP
ypseu0001X_4150  RDYIRQRIADAHPYKIPATVIAREIRDQGYRGGMTILRAFIRSLSVPQEQEPAVRFETEP
ypest0001X_4315  RDYIRQRIADAHPYKIPATVIAREIRDQGYRGGMTILRAFIRSLSVPQEQEPAVRFETEP
ypest0001X_4080  RDYIRQRIADAHPYKIPATVIAREIRDQGYRGGMTILRAFIRSLSVPQEQEPAVRFETEP
ypest0001X_4060  RDYIRQRIADAHPYKIPATVIAREIRDQGYRGGMTILRAFIRSLSVPQEQEPAVRFETEP
ypest0001X_4005  RDYIRQRIADAHPYKIPATVIAREIRDQGYRGGMTILRAFIRSLSVPQEQEPAVRFETEP
ypest0001X_3885  RDYIRQRIADAHPYKIPATVIAREIRDQGYRGGMTILRAFIRSLSVPQEQEPAVRFETEP
ypest0001X_3836  RDYIRQRIADAHPYKIPATVIAREIRDQGYRGGMTILRAFIRSLSVPQEQEPAVRFETEP
ypest0001X_3727  RDYIRQRIADAHPYKIPATVIAREIRDQGYRGGMTILRAFIRSLSVPQEQEPAVRFETEP
ypest0001X_3562  RDYIRQRIADAHPYKIPATVIAREIRDQGYRGGMTILRAFIRSLSVPQEQEPAVRFETEP
ypest0001X_3528  RDYIRQRIADAHPYKIPATVIAREIRDQGYRGGMTILRAFIRSLSVPQEQEPAVRFETEP
ypest0001X_3487  RDYIRQRIADAHPYKIPATVIAREIRDQGYRGGMTILRAFIRSLSVPQEQEPAVRFETEP
ypest0001X_3212  RDYIRQRIADAHPYKIPATVIAREIRDQGYRGGMTILRAFIRSLSVPQEQEPAVRFETEP
ypest0001X_3093  RDYIRQRIADAHPYKIPATVIAREIRDQGYRGGMTILRAFIRSLSVPQEQEPAVRFETEP
ypest0001X_3005  RDYIRQRIADAHPYKIPATVIAREIRDQGYRGGMTILRAFIRSLSVPQEQEPAVRFETEP
ypest0001X_2922  RDYIRQRIADAHPYKIPATVIAREIRDQGYRGGMTILRAFIRSLSVPQEQEPAVRFETEP
ypest0001X_2788  RDYIRQRIADAHPYKIPATVIAREIRDQGYRGGMTILRAFIRSLSVPQEQEPAVRFETEP
ypest0001X_2665  RDYIRQRIADAHPYKIPATVIAREIRDQGYRGGMTILRAFIRSLSVPQEQEPAVRFETEP
ypest0001X_2634  RDYIRQRIADAHPYKIPATVIAREIRDQGYRGGMTILRAFIRSLSVPQEQEPAVRFETEP
ypest0001X_2441  RDYIRQRIADAHPYKIPATVIAREIRDQGYRGGMTILRAFIRSLSVPQEQEPAVRFETEP
ypest0001X_2350  RDYIRQRIADAHPYKIPATVIAREIRDQGYRGGMTILRAFIRSLSVPQEQEPAVRFETEP
ypest0001X_2288  RDYIRQRIADAHPYKIPATVIAREIRDQGYRGGMTILRAFIRSLSVPQEQEPAVRFETEP
ypest0001X_2153  RDYIRQRIADAHPYKIPATVIAREIRDQGYRGGMTILRAFIRSLSVPQEQEPAVRFETEP
ypest0001X_1996  RDYIRQRIADAHPYKIPATVIAREIRDQGYRGGMTILRAFIRSLSVPQEQEPAVRFETEP
ypest0001X_1975  RDYIRQRIADAHPYKIPATVIAREIRDQGYRGGMTILRAFIRSLSVPQEQEPAVRFETEP
ypest0001X_1936  RDYIRQRIADAHPYKIPATVIAREIRDQGYRGGMTILRAFIRSLSVPQEQEPAVRFETEP
ypest0001X_1862  RDYIRQRIADAHPYKIPATVIAREIRDQGYRGGMTILRAFIRSLSVPQEQEPAVRFETEP
ypest0001X_1756  RDYIRQRIADAHPYKIPATVIAREIRDQGYRGGMTILRAFIRSLSVPQEQEPAVRFETEP
ypest0001X_1659  RDYIRQRIADAHPYKIPATVIAREIRDQGYRGGMTILRAFIRSLSVPQEQEPAVRFETEP
ypest0001X_1562  RDYIRQRIADAHPYKIPATVIAREIRDQGYRGGMTILRAFIRSLSVPQEQEPAVRFETEP
ypest0001X_1343  RDYIRQRIADAHPYKIPATVIAREIRDQGYRGGMTILRAFIRSLSVPQEQEPAVRFETEP
ypest0001X_1298  RDYIRQRIADAHPYKIPATVIAREIRDQGYRGGMTILRAFIRSLSVPQEQEPAVRFETEP
ypest0001X_1222  RDYIRQRIADAHPYKIPATVIAREIRDQGYRGGMTILRAFIRSLSVPQEQEPAVRFETEP
ypest0001X_1127  RDYIRQRIADAHPYKIPATVIAREIRDQGYRGGMTILRAFIRSLSVPQEQEPAVRFETEP
ypest0001X_1028  RDYIRQRIADAHPYKIPATVIAREIRDQGYRGGMTILRAFIRSLSVPQEQEPAVRFETEP
ypest0001X_9850  RDYIRQRIADAHPYKIPATVIAREIRDQGYRGGMTILRAFIRSLSVPQEQEPAVRFETEP
ypest0001X_8440  RDYIRQRIADAHPYKIPATVIAREIRDQGYRGGMTILRAFIRSLSVPQEQEPAVRFETEP
ypest0001X_7760  RDYIRQRIADAHPYKIPATVIAREIRDQGYRGGMTILRAFIRSLSVPQEQEPAVRFETEP
ypest0001X_7380  RDYIRQRIADAHPYKIPATVIAREIRDQGYRGGMTILRAFIRSLSVPQEQEPAVRFETEP
ypest0001X_5890  RDYIRQRIADAHPYKIPATVIAREIRDQGYRGGMTILRAFIRSLSVPQEQEPAVRFETEP
ypest0001X_4460  RDYIRQRIADAHPYKIPATVIAREIRDQGYRGGMTILRAFIRSLSVPQEQEPAVRFETEP
ypest0001X_3710  RDYIRQRIADAHPYKIPATVIAREIRDQGYRGGMTILRAFIRSLSVPQEQEPAVRFETEP
ypest0001X_2310  RDYIRQRIADAHPYKIPATVIAREIRDQGYRGGMTILRAFIRSLSVPQEQEPAVRFETEP
ypest0001X_1670  RDYIRQRIADAHPYKIPATVIAREIRDQGYRGGMTILRAFIRSLSVPQEQEPAVRFETEP
ypest0001X_1000  RDYIRQRIADAHPYKIPATVIAREIRDQGYRGGMTILRAFIRSLSVPQEQEPAVRFETEP
ypest0001X_890   RDYIRQRIADAHPYKIPATVIAREIRDQGYRGGMTILRAFIRSLSVPQEQEPAVRFETEP
ypest0001X_10    RDYIRQRIADAHPYKIPATVIAREIRDQGYRGGMTILRAFIRSLSVPQEQEPAVRFETEP
                 ############################################################


                        130       140       150       160       170       180
                 =========+=========+=========+=========+=========+=========+
ypseu0001X_140   GISTDGEMGKM-----VVTILSAVAQAERQRI----LERTNEGREE----------AKLK
ypest0001X_830   GISTDGEMGKM-----VVTILSAVAQAERQRI----LERTNEGREE----------AKLK
yente0001X_2473  EELIKAYLRGQFTNLTSGTIYH---QFDRRLNHCDEVEQPGEPLYIGMDFNVGKMAAIVH
ykris0001_3040   EEADESDFAA------PSYLQP--------------YELIEESQYNALMT------QTIE
ypest0001X_3403  GRQMQVDWGTMRNGRSPLHVFVAVLGYSRMLY----IEFTDNMRYDTLET------CHRN
ypest0001X_2225  GRQMQVDWGTMRNGRSPLHVFVAVLGYSRMLY----IEFTDNMRYDTLET------CHRN
ypest0001X_2950  GRQMQVDWGTMRNGRSPLHVFVAVLGYSRMLY----IEFTDNMRYDTLET------CHRN
ypseu0001X_4293  GRQMQVDWGTMRNGRSPLHVFVAVLGYSRMLY----IEFTDNMRYDTLET------CHRN
ypseu0001X_2061  GRQMQVDWGTMRNGRSPLHVFVAVLGYSRMLY----IEFTDNMRYDTLET------CHRN
ypseu0001X_1809  GRQMQVDWGTMRNGRSPLHVFVAVLGYSRMLY----IEFTDNMRYDTLET------CHRN
ypseu0001X_1304  GRQMQVDWGTMRNGRSPLHVFVAVLGYSRMLY----IEFTDNMRYDTLET------CHRN
ypseu0001X_4150  GRQMQVDWGTMRNGRSPLHVFVAVLGYSRMLY----IEFTDNMRYDTLET------CHRN
ypest0001X_4315  GRQMQVDWGTMRNGRSPLHVFVAVLGYSRMLY----IEFTDNMRYDTLET------CHRN
ypest0001X_4080  GRQMQVDWGTMRNGRSPLHVFVAVLGYSRMLY----IEFTDNMRYDTLET------CHRN
ypest0001X_4060  GRQMQVDWGTMRNGRSPLHVFVAVLGYSRMLY----IEFTDNMRYDTLET------CHRN
ypest0001X_4005  GRQMQVDWGTMRNGRSPLHVFVAVLGYSRMLY----IEFTDNMRYDTLET------CHRN
ypest0001X_3885  GRQMQVDWGTMRNGRSPLHVFVAVLGYSRMLY----IEFTDNMRYDTLET------CHRN
ypest0001X_3836  GRQMQVDWGTMRNGRSPLHVFVAVLGYSRMLY----IEFTDNMRYDTLET------CHRN
ypest0001X_3727  GRQMQVDWGTMRNGRSPLHVFVAVLGYSRMLY----IEFTDNMRYDTLET------CHRN
ypest0001X_3562  GRQMQVDWGTMRNGRSPLHVFVAVLGYSRMLY----IEFTDNMRYDTLET------CHRN
ypest0001X_3528  GRQMQVDWGTMRNGRSPLHVFVAVLGYSRMLY----IEFTDNMRYDTLET------CHRN
ypest0001X_3487  GRQMQVDWGTMRNGRSPLHVFVAVLGYSRMLY----IEFTDNMRYDTLET------CHRN
ypest0001X_3212  GRQMQVDWGTMRNGRSPLHVFVAVLGYSRMLY----IEFTDNMRYDTLET------CHRN
ypest0001X_3093  GRQMQVDWGTMRNGRSPLHVFVAVLGYSRMLY----IEFTDNMRYDTLET------CHRN
ypest0001X_3005  GRQMQVDWGTMRNGRSPLHVFVAVLGYSRMLY----IEFTDNMRYDTLET------CHRN
ypest0001X_2922  GRQMQVDWGTMRNGRSPLHVFVAVLGYSRMLY----IEFTDNMRYDTLET------CHRN
ypest0001X_2788  GRQMQVDWGTMRNGRSPLHVFVAVLGYSRMLY----IEFTDNMRYDTLET------CHRN
ypest0001X_2665  GRQMQVDWGTMRNGRSPLHVFVAVLGYSRMLY----IEFTDNMRYDTLET------CHRN
ypest0001X_2634  GRQMQVDWGTMRNGRSPLHVFVAVLGYSRMLY----IEFTDNMRYDTLET------CHRN
ypest0001X_2441  GRQMQVDWGTMRNGRSPLHVFVAVLGYSRMLY----IEFTDNMRYDTLET------CHRN
ypest0001X_2350  GRQMQVDWGTMRNGRSPLHVFVAVLGYSRMLY----IEFTDNMRYDTLET------CHRN
ypest0001X_2288  GRQMQVDWGTMRNGRSPLHVFVAVLGYSRMLY----IEFTDNMRYDTLET------CHRN
ypest0001X_2153  GRQMQVDWGTMRNGRSPLHVFVAVLGYSRMLY----IEFTDNMRYDTLET------CHRN
ypest0001X_1996  GRQMQVDWGTMRNGRSPLHVFVAVLGYSRMLY----IEFTDNMRYDTLET------CHRN
ypest0001X_1975  GRQMQVDWGTMRNGRSPLHVFVAVLGYSRMLY----IEFTDNMRYDTLET------CHRN
ypest0001X_1936  GRQMQVDWGTMRNGRSPLHVFVAVLGYSRMLY----IEFTDNMRYDTLET------CHRN
ypest0001X_1862  GRQMQVDWGTMRNGRSPLHVFVAVLGYSRMLY----IEFTDNMRYDTLET------CHRN
ypest0001X_1756  GRQMQVDWGTMRNGRSPLHVFVAVLGYSRMLY----IEFTDNMRYDTLET------CHRN
ypest0001X_1659  GRQMQVDWGTMRNGRSPLHVFVAVLGYSRMLY----IEFTDNMRYDTLET------CHRN
ypest0001X_1562  GRQMQVDWGTMRNGRSPLHVFVAVLGYSRMLY----IEFTDNMRYDTLET------CHRN
ypest0001X_1343  GRQMQVDWGTMRNGRSPLHVFVAVLGYSRMLY----IEFTDNMRYDTLET------CHRN
ypest0001X_1298  GRQMQVDWGTMRNGRSPLHVFVAVLGYSRMLY----IEFTDNMRYDTLET------CHRN
ypest0001X_1222  GRQMQVDWGTMRNGRSPLHVFVAVLGYSRMLY----IEFTDNMRYDTLET------CHRN
ypest0001X_1127  GRQMQVDWGTMRNGRSPLHVFVAVLGYSRMLY----IEFTDNMRYDTLET------CHRN
ypest0001X_1028  GRQMQVDWGTMRNGRSPLHVFVAVLGYSRMLY----IEFTDNMRYDTLET------CHRN
ypest0001X_9850  GRQMQVDWGTMRNGRSPLHVFVAVLGYSRMLY----IEFTDNMRYDTLET------CHRN
ypest0001X_8440  GRQMQVDWGTMRNGRSPLHVFVAVLGYSRMLY----IEFTDNMRYDTLET------CHRN
ypest0001X_7760  GRQMQVDWGTMRNGRSPLHVFVAVLGYSRMLY----IEFTDNMRYDTLET------CHRN
ypest0001X_7380  GRQMQVDWGTMRNGRSPLHVFVAVLGYSRMLY----IEFTDNMRYDTLET------CHRN
ypest0001X_5890  GRQMQVDWGTMRNGRSPLHVFVAVLGYSRMLY----IEFTDNMRYDTLET------CHRN
ypest0001X_4460  GRQMQVDWGTMRNGRSPLHVFVAVLGYSRMLY----IEFTDNMRYDTLET------CHRN
ypest0001X_3710  GRQMQVDWGTMRNGRSPLHVFVAVLGYSRMLY----IEFTDNMRYDTLET------CHRN
ypest0001X_2310  GRQMQVDWGTMRNGRSPLHVFVAVLGYSRMLY----IEFTDNMRYDTLET------CHRN
ypest0001X_1670  GRQMQVDWGTMRNGRSPLHVFVAVLGYSRMLY----IEFTDNMRYDTLET------CHRN
ypest0001X_1000  GRQMQVDWGTMRNGRSPLHVFVAVLGYSRMLY----IEFTDNMRYDTLET------CHRN
ypest0001X_890   GRQMQVDWGTMRNGRSPLHVFVAVLGYSRMLY----IEFTDNMRYDTLET------CHRN
ypest0001X_10    GRQMQVDWGTMRNGRSPLHVFVAVLGYSRMLY----IEFTDNMRYDTLET------CHRN
                 ################################    ##############      ####


                        190       200       210       220       230       240
                 =========+=========+=========+=========+=========+=========+
ypseu0001X_140   GVKF--GRKRRIN--------------RKELLELHEQ-------------GMGATEIA--
ypest0001X_830   GVKF--GRKRRIN--------------RKELLELHEQ-------------GMGATEIA--
yente0001X_2473  VLRL--GLPCVVTEIINAYDTPDMIRIIKERFWL----YDGNNYRKVREIYIYPDASGDS
ykris0001_3040   ALP-----PR-----------------CREAFIL--HRFD----------GLSQKEVAS-
ypest0001X_3403  AFRFFGGVPREVL-----YDNMKTVVLQRDAYQTGQHRFHPSLWQFGKEMGFSPRLCRPF
ypest0001X_2225  AFRFFGGVPREVL-----YDNMKTVVLQRDAYQTGQHRFHPSLWQFGKEMGFSPRLCRPF
ypest0001X_2950  AFRFFGGVPREVL-----YDNMKTVVLQRDAYQTGQHRFHPSLWQFGKEMGFSPRLCRPF
ypseu0001X_4293  AFRFFGGVPREVL-----YDNMKTVVLQRDAYQTGQHRFHPSLWQFGKEMGFSPRLCRPF
ypseu0001X_2061  AFRFFGGVPREVL-----YDNMKTVVLQRDAYQTGQHRFHPSLWQFGKEMGFSPRLCRPF
ypseu0001X_1809  AFRFFGGVPREVL-----YDNMKTVVLQRDAYQTGQHRFHPSLWQFGKEMGFSPRLCRPF
ypseu0001X_1304  AFRFFGGVPREVL-----YDNMKTVVLQRDAYQTGQHRFHPSLWQFGKEMGFSPRLCRPF
ypseu0001X_4150  AFRFFGGVPREVL-----YDNMKTVVLQRDAYQTGQHRFHPSLWQFGKEMGFSPRLCRPF
ypest0001X_4315  AFRFFGGVPREVL-----YDNMKTVVLQRDAYQTGQHRFHPSLWQFGKEMGFSPRLCRPF
ypest0001X_4080  AFRFFGGVPREVL-----YDNMKTVVLQRDAYQTGQHRFHPSLWQFGKEMGFSPRLCRPF
ypest0001X_4060  AFRFFGGVPREVL-----YDNMKTVVLQRDAYQTGQHRFHPSLWQFGKEMGFSPRLCRPF
ypest0001X_4005  AFRFFGGVPREVL-----YDNMKTVVLQRDAYQTGQHRFHPSLWQFGKEMGFSPRLCRPF
ypest0001X_3885  AFRFFGGVPREVL-----YDNMKTVVLQRDAYQTGQHRFHPSLWQFGKEMGFSPRLCRPF
ypest0001X_3836  AFRFFGGVPREVL-----YDNMKTVVLQRDAYQTGQHRFHPSLWQFGKEMGFSPRLCRPF
ypest0001X_3727  AFRFFGGVPREVL-----YDNMKTVVLQRDAYQTGQHRFHPSLWQFGKEMGFSPRLCRPF
ypest0001X_3562  AFRFFGGVPREVL-----YDNMKTVVLQRDAYQTGQHRFHPSLWQFGKEMGFSPRLCRPF
ypest0001X_3528  AFRFFGGVPREVL-----YDNMKTVVLQRDAYQTGQHRFHPSLWQFGKEMGFSPRLCRPF
ypest0001X_3487  AFRFFGGVPREVL-----YDNMKTVVLQRDAYQTGQHRFHPSLWQFGKEMGFSPRLCRPF
ypest0001X_3212  AFRFFGGVPREVL-----YDNMKTVVLQRDAYQTGQHRFHPSLWQFGKEMGFSPRLCRPF
ypest0001X_3093  AFRFFGGVPREVL-----YDNMKTVVLQRDAYQTGQHRFHPSLWQFGKEMGFSPRLCRPF
ypest0001X_3005  AFRFFGGVPREVL-----YDNMKTVVLQRDAYQTGQHRFHPSLWQFGKEMGFSPRLCRPF
ypest0001X_2922  AFRFFGGVPREVL-----YDNMKTVVLQRDAYQTGQHRFHPSLWQFGKEMGFSPRLCRPF
ypest0001X_2788  AFRFFGGVPREVL-----YDNMKTVVLQRDAYQTGQHRFHPSLWQFGKEMGFSPRLCRPF
ypest0001X_2665  AFRFFGGVPREVL-----YDNMKTVVLQRDAYQTGQHRFHPSLWQFGKEMGFSPRLCRPF
ypest0001X_2634  AFRFFGGVPREVL-----YDNMKTVVLQRDAYQTGQHRFHPSLWQFGKEMGFSPRLCRPF
ypest0001X_2441  AFRFFGGVPREVL-----YDNMKTVVLQRDAYQTGQHRFHPSLWQFGKEMGFSPRLCRPF
ypest0001X_2350  AFRFFGGVPREVL-----YDNMKTVVLQRDAYQTGQHRFHPSLWQFGKEMGFSPRLCRPF
ypest0001X_2288  AFRFFGGVPREVL-----YDNMKTVVLQRDAYQTGQHRFHPSLWQFGKEMGFSPRLCRPF
ypest0001X_2153  AFRFFGGVPREVL-----YDNMKTVVLQRDAYQTGQHRFHPSLWQFGKEMGFSPRLCRPF
ypest0001X_1996  AFRFFGGVPREVL-----YDNMKTVVLQRDAYQTGQHRFHPSLWQFGKEMGFSPRLCRPF
ypest0001X_1975  AFRFFGGVPREVL-----YDNMKTVVLQRDAYQTGQHRFHPSLWQFGKEMGFSPRLCRPF
ypest0001X_1936  AFRFFGGVPREVL-----YDNMKTVVLQRDAYQTGQHRFHPSLWQFGKEMGFSPRLCRPF
ypest0001X_1862  AFRFFGGVPREVL-----YDNMKTVVLQRDAYQTGQHRFHPSLWQFGKEMGFSPRLCRPF
ypest0001X_1756  AFRFFGGVPREVL-----YDNMKTVVLQRDAYQTGQHRFHPSLWQFGKEMGFSPRLCRPF
ypest0001X_1659  AFRFFGGVPREVL-----YDNMKTVVLQRDAYQTGQHRFHPSLWQFGKEMGFSPRLCRPF
ypest0001X_1562  AFRFFGGVPREVL-----YDNMKTVVLQRDAYQTGQHRFHPSLWQFGKEMGFSPRLCRPF
ypest0001X_1343  AFRFFGGVPREVL-----YDNMKTVVLQRDAYQTGQHRFHPSLWQFGKEMGFSPRLCRPF
ypest0001X_1298  AFRFFGGVPREVL-----YDNMKTVVLQRDAYQTGQHRFHPSLWQFGKEMGFSPRLCRPF
ypest0001X_1222  AFRFFGGVPREVL-----YDNMKTVVLQRDAYQTGQHRFHPSLWQFGKEMGFSPRLCRPF
ypest0001X_1127  AFRFFGGVPREVL-----YDNMKTVVLQRDAYQTGQHRFHPSLWQFGKEMGFSPRLCRPF
ypest0001X_1028  AFRFFGGVPREVL-----YDNMKTVVLQRDAYQTGQHRFHPSLWQFGKEMGFSPRLCRPF
ypest0001X_9850  AFRFFGGVPREVL-----YDNMKTVVLQRDAYQTGQHRFHPSLWQFGKEMGFSPRLCRPF
ypest0001X_8440  AFRFFGGVPREVL-----YDNMKTVVLQRDAYQTGQHRFHPSLWQFGKEMGFSPRLCRPF
ypest0001X_7760  AFRFFGGVPREVL-----YDNMKTVVLQRDAYQTGQHRFHPSLWQFGKEMGFSPRLCRPF
ypest0001X_7380  AFRFFGGVPREVL-----YDNMKTVVLQRDAYQTGQHRFHPSLWQFGKEMGFSPRLCRPF
ypest0001X_5890  AFRFFGGVPREVL-----YDNMKTVVLQRDAYQTGQHRFHPSLWQFGKEMGFSPRLCRPF
ypest0001X_4460  AFRFFGGVPREVL-----YDNMKTVVLQRDAYQTGQHRFHPSLWQFGKEMGFSPRLCRPF
ypest0001X_3710  AFRFFGGVPREVL-----YDNMKTVVLQRDAYQTGQHRFHPSLWQFGKEMGFSPRLCRPF
ypest0001X_2310  AFRFFGGVPREVL-----YDNMKTVVLQRDAYQTGQHRFHPSLWQFGKEMGFSPRLCRPF
ypest0001X_1670  AFRFFGGVPREVL-----YDNMKTVVLQRDAYQTGQHRFHPSLWQFGKEMGFSPRLCRPF
ypest0001X_1000  AFRFFGGVPREVL-----YDNMKTVVLQRDAYQTGQHRFHPSLWQFGKEMGFSPRLCRPF
ypest0001X_890   AFRFFGGVPREVL-----YDNMKTVVLQRDAYQTGQHRFHPSLWQFGKEMGFSPRLCRPF
ypest0001X_10    AFRFFGGVPREVL-----YDNMKTVVLQRDAYQTGQHRFHPSLWQFGKEMGFSPRLCRPF
                 #############     ##########################################


                        250       260       270       280       290       300
                 =========+=========+=========+=========+=========+=========+
ypseu0001X_140   ---------------------------KKMNIARST----------IYKVINESQ-----
ypest0001X_830   ---------------------------KKMNIARST----------IYKVINESQ-----
yente0001X_2473  RKSSHASTTDIVQLKQAGFNVVVNDSNPPVKDRINA-------MNAMFCNGDGERRYKVN
ykris0001_3040   ----------------------------QMGISINM--VEKHIIRAM--LACRVSNESWQ
ypest0001X_3403  RAQTKGKVERMVQYTRNSFYIPLMTRLRPMGITVDVETANRHGLRWLHDVANQRKHETIQ
ypest0001X_2225  RAQTKGKVERMVQYTRNSFYIPLMTRLRPMGITVDVETANRHGLRWLHDVANQRKHETIQ
ypest0001X_2950  RAQTKGKVERMVQYTRNSFYIPLMTRLRPMGITVDVETANRHGLRWLHDVANQRKHETIQ
ypseu0001X_4293  RAQTKGKVERMVQYTRNSFYIPLMTRLRPMGITVDVETANRHGLRWLHDVANQRKHETIQ
ypseu0001X_2061  RAQTKGKVERMVQYTRNSFYIPLMTRLRPMGITVDVETANRHGLRWLHDVANQRKHETIQ
ypseu0001X_1809  RAQTKGKVERMVQYTRNSFYIPLMTRLRPMGITVDVETANRHGLRWLHDVANQRKHETIQ
ypseu0001X_1304  RAQTKGKVERMVQYTRNSFYIPLMTRLRPMGITVDVETANRHGLRWLHDVANQRKHETIQ
ypseu0001X_4150  RAQTKGKVERMVQYTRNSFYIPLMTRLRPMGITVDVETANRHGLRWLHDVANQRKHETIQ
ypest0001X_4315  RAQTKGKVERMVQYTRNSFYIPLMTRLRPMGITVDVETANRHGLRWLHDVANQRKHETIQ
ypest0001X_4080  RAQTKGKVERMVQYTRNSFYIPLMTRLRPMGITVDVETANRHGLRWLHDVANQRKHETIQ
ypest0001X_4060  RAQTKGKVERMVQYTRNSFYIPLMTRLRPMGITVDVETANRHGLRWLHDVANQRKHETIQ
ypest0001X_4005  RAQTKGKVERMVQYTRNSFYIPLMTRLRPMGITVDVETANRHGLRWLHDVANQRKHETIQ
ypest0001X_3885  RAQTKGKVERMVQYTRNSFYIPLMTRLRPMGITVDVETANRHGLRWLHDVANQRKHETIQ
ypest0001X_3836  RAQTKGKVERMVQYTRNSFYIPLMTRLRPMGITVDVETANRHGLRWLHDVANQRKHETIQ
ypest0001X_3727  RAQTKGKVERMVQYTRNSFYIPLMTRLRPMGITVDVETANRHGLRWLHDVANQRKHETIQ
ypest0001X_3562  RAQTKGKVERMVQYTRNSFYIPLMTRLRPMGITVDVETANRHGLRWLHDVANQRKHETIQ
ypest0001X_3528  RAQTKGKVERMVQYTRNSFYIPLMTRLRPMGITVDVETANRHGLRWLHDVANQRKHETIQ
ypest0001X_3487  RAQTKGKVERMVQYTRNSFYIPLMTRLRPMGITVDVETANRHGLRWLHDVANQRKHETIQ
ypest0001X_3212  RAQTKGKVERMVQYTRNSFYIPLMTRLRPMGITVDVETANRHGLRWLHDVANQRKHETIQ
ypest0001X_3093  RAQTKGKVERMVQYTRNSFYIPLMTRLRPMGITVDVETANRHGLRWLHDVANQRKHETIQ
ypest0001X_3005  RAQTKGKVERMVQYTRNSFYIPLMTRLRPMGITVDVETANRHGLRWLHDVANQRKHETIQ
ypest0001X_2922  RAQTKGKVERMVQYTRNSFYIPLMTRLRPMGITVDVETANRHGLRWLHDVANQRKHETIQ
ypest0001X_2788  RAQTKGKVERMVQYTRNSFYIPLMTRLRPMGITVDVETANRHGLRWLHDVANQRKHETIQ
ypest0001X_2665  RAQTKGKVERMVQYTRNSFYIPLMTRLRPMGITVDVETANRHGLRWLHDVANQRKHETIQ
ypest0001X_2634  RAQTKGKVERMVQYTRNSFYIPLMTRLRPMGITVDVETANRHGLRWLHDVANQRKHETIQ
ypest0001X_2441  RAQTKGKVERMVQYTRNSFYIPLMTRLRPMGITVDVETANRHGLRWLHDVANQRKHETIQ
ypest0001X_2350  RAQTKGKVERMVQYTRNSFYIPLMTRLRPMGITVDVETANRHGLRWLHDVANQRKHETIQ
ypest0001X_2288  RAQTKGKVERMVQYTRNSFYIPLMTRLRPMGITVDVETANRHGLRWLHDVANQRKHETIQ
ypest0001X_2153  RAQTKGKVERMVQYTRNSFYIPLMTRLRPMGITVDVETANRHGLRWLHDVANQRKHETIQ
ypest0001X_1996  RAQTKGKVERMVQYTRNSFYIPLMTRLRPMGITVDVETANRHGLRWLHDVANQRKHETIQ
ypest0001X_1975  RAQTKGKVERMVQYTRNSFYIPLMTRLRPMGITVDVETANRHGLRWLHDVANQRKHETIQ
ypest0001X_1936  RAQTKGKVERMVQYTRNSFYIPLMTRLRPMGITVDVETANRHGLRWLHDVANQRKHETIQ
ypest0001X_1862  RAQTKGKVERMVQYTRNSFYIPLMTRLRPMGITVDVETANRHGLRWLHDVANQRKHETIQ
ypest0001X_1756  RAQTKGKVERMVQYTRNSFYIPLMTRLRPMGITVDVETANRHGLRWLHDVANQRKHETIQ
ypest0001X_1659  RAQTKGKVERMVQYTRNSFYIPLMTRLRPMGITVDVETANRHGLRWLHDVANQRKHETIQ
ypest0001X_1562  RAQTKGKVERMVQYTRNSFYIPLMTRLRPMGITVDVETANRHGLRWLHDVANQRKHETIQ
ypest0001X_1343  RAQTKGKVERMVQYTRNSFYIPLMTRLRPMGITVDVETANRHGLRWLHDVANQRKHETIQ
ypest0001X_1298  RAQTKGKVERMVQYTRNSFYIPLMTRLRPMGITVDVETANRHGLRWLHDVANQRKHETIQ
ypest0001X_1222  RAQTKGKVERMVQYTRNSFYIPLMTRLRPMGITVDVETANRHGLRWLHDVANQRKHETIQ
ypest0001X_1127  RAQTKGKVERMVQYTRNSFYIPLMTRLRPMGITVDVETANRHGLRWLHDVANQRKHETIQ
ypest0001X_1028  RAQTKGKVERMVQYTRNSFYIPLMTRLRPMGITVDVETANRHGLRWLHDVANQRKHETIQ
ypest0001X_9850  RAQTKGKVERMVQYTRNSFYIPLMTRLRPMGITVDVETANRHGLRWLHDVANQRKHETIQ
ypest0001X_8440  RAQTKGKVERMVQYTRNSFYIPLMTRLRPMGITVDVETANRHGLRWLHDVANQRKHETIQ
ypest0001X_7760  RAQTKGKVERMVQYTRNSFYIPLMTRLRPMGITVDVETANRHGLRWLHDVANQRKHETIQ
ypest0001X_7380  RAQTKGKVERMVQYTRNSFYIPLMTRLRPMGITVDVETANRHGLRWLHDVANQRKHETIQ
ypest0001X_5890  RAQTKGKVERMVQYTRNSFYIPLMTRLRPMGITVDVETANRHGLRWLHDVANQRKHETIQ
ypest0001X_4460  RAQTKGKVERMVQYTRNSFYIPLMTRLRPMGITVDVETANRHGLRWLHDVANQRKHETIQ
ypest0001X_3710  RAQTKGKVERMVQYTRNSFYIPLMTRLRPMGITVDVETANRHGLRWLHDVANQRKHETIQ
ypest0001X_2310  RAQTKGKVERMVQYTRNSFYIPLMTRLRPMGITVDVETANRHGLRWLHDVANQRKHETIQ
ypest0001X_1670  RAQTKGKVERMVQYTRNSFYIPLMTRLRPMGITVDVETANRHGLRWLHDVANQRKHETIQ
ypest0001X_1000  RAQTKGKVERMVQYTRNSFYIPLMTRLRPMGITVDVETANRHGLRWLHDVANQRKHETIQ
ypest0001X_890   RAQTKGKVERMVQYTRNSFYIPLMTRLRPMGITVDVETANRHGLRWLHDVANQRKHETIQ
ypest0001X_10    RAQTKGKVERMVQYTRNSFYIPLMTRLRPMGITVDVETANRHGLRWLHDVANQRKHETIQ
                 ############################################################


                        310       320       330       340       350       360
                 =========+=========+=========+=========+=========+=========+
ypseu0001X_140   ------------------------------------------------------------
ypest0001X_830   ------------------------------------------------------------
yente0001X_2473  VMRCPVYTESLEQQVWADNGEPDKSADNDHPNDAG-------------------------
ykris0001_3040   QQ---EW---RDKKVG-------KKSED--------------------------------
ypest0001X_3403  ARPCDRW---LEEQQSMLALPPEKKEYDVHLDENLELRQTPPASSTLHLRLILQRSGVMM
ypest0001X_2225  ARPCDRW---LEEQQSMLALPPEKKEYDVHLDENL-------------------------
ypest0001X_2950  ACTCDRW---LEEQQSMLALPPEKKEYDVHLDENL-------------------------
ypseu0001X_4293  ARPCDRW---LEEQQSMLALPPEKKEYDVHLDENL-------------------------
ypseu0001X_2061  ARPCDRW---LEEQQSMLALPPEKKEYDVHLDENL-------------------------
ypseu0001X_1809  ARPCDRW---LEEQQSMLALPPEKKEYDVHLDENL-------------------------
ypseu0001X_1304  ARPCDRW---LEEQQSMLALPPEKKEYDVHLDENL-------------------------
ypseu0001X_4150  ARPCDRW---LEEQQSMLALPPEKKEYDVHLDENL-------------------------
ypest0001X_4315  ARPCDRW---LEEQQSMLALPPEKKEYDVHLDENL-------------------------
ypest0001X_4080  ARPCDRW---LEEQQSMLALPPEKKEYDVHLDENL-------------------------
ypest0001X_4060  ARPCDRW---LEEQQSMLALPPEKKEYDVHLDENL-------------------------
ypest0001X_4005  ARPCDRW---LEEQQSMLALPPEKKEYDVHLDENL-------------------------
ypest0001X_3885  ARPCDRW---LEEQQSMLALPPEKKEYDVHLDENL-------------------------
ypest0001X_3836  ARPCDRW---LEEQQSMLALPPEKKEYDVHLDENL-------------------------
ypest0001X_3727  ARPCDRW---LEEQQSMLALPPEKKEYDVHLDENL-------------------------
ypest0001X_3562  ARPCDRW---LEEQQSMLALPPEKKEYDVHLDENL-------------------------
ypest0001X_3528  ARPCDRW---LEEQQSMLALPPEKKEYDVHLDENL-------------------------
ypest0001X_3487  ARPCDRW---LEEQQSMLALPPEKKEYDVHLDENL-------------------------
ypest0001X_3212  ARPCDRW---LEEQQSMLALPPEKKEYDVHLDENL-------------------------
ypest0001X_3093  ARPCDRW---LEEQQSMLALPPEKKEYDVHLDENL-------------------------
ypest0001X_3005  ARPCDRW---LEEQQSMLALPPEKKEYDVHLDENL-------------------------
ypest0001X_2922  ARPCDRW---LEEQQSMLALPPEKKEYDVHLDENL-------------------------
ypest0001X_2788  ARPCDRW---LEEQQSMLALPPEKKEYDVHLDENL-------------------------
ypest0001X_2665  ARPCDRW---LEEQQSMLALPPEKKEYDVHLDENL-------------------------
ypest0001X_2634  ARPCDRW---LEEQQSMLALPPEKKEYDVHLDENL-------------------------
ypest0001X_2441  ARPCDRW---LEEQQSMLALPPEKKEYDVHLDENL-------------------------
ypest0001X_2350  ARPCDRW---LEEQQSMLALPPEKKEYDVHLDENL-------------------------
ypest0001X_2288  ARPCDRW---LEEQQSMLALPPEKKEYDVHLDENL-------------------------
ypest0001X_2153  ARPCDRW---LEEQQSMLALPPEKKEYDVHLDENL-------------------------
ypest0001X_1996  ARPCDRW---LEEQQSMLALPPEKKEYDVHLDENL-------------------------
ypest0001X_1975  ARPCDRW---LEEQQSMLALPPEKKEYDVHLDENL-------------------------
ypest0001X_1936  ARPCDRW---LEEQQSMLALPPEKKEYDVHLDENL-------------------------
ypest0001X_1862  ARPCDRW---LEEQQSMLALPPEKKEYDVHLDENL-------------------------
ypest0001X_1756  ARPCDRW---LEEQQSMLALPPEKKEYDVHLDENL-------------------------
ypest0001X_1659  ARPCDRW---LEEQQSMLALPPEKKEYDVHLDENL-------------------------
ypest0001X_1562  ARPCDRW---LEEQQSMLALPPEKKEYDVHLDENL-------------------------
ypest0001X_1343  ARPCDRW---LEEQQSMLALPPEKKEYDVHLDENL-------------------------
ypest0001X_1298  ARPCDRW---LEEQQSMLALPPEKKEYDVHLDENL-------------------------
ypest0001X_1222  ARPCDRW---LEEQQSMLALPPEKKEYDVHLDENL-------------------------
ypest0001X_1127  ARPCDRW---LEEQQSMLALPPEKKEYDVHLDENL-------------------------
ypest0001X_1028  ARPCDRW---LEEQQSMLALPPEKKEYDVHLDENL-------------------------
ypest0001X_9850  ARPCDRW---LEEQQSMLALPPEKKEYDVHLDENL-------------------------
ypest0001X_8440  ARPCDRW---LEEQQSMLALPPEKKEYDVHLDENL-------------------------
ypest0001X_7760  ARPCDRW---LEEQQSMLALPPEKKEYDVHLDENL-------------------------
ypest0001X_7380  ARPCDRW---LEEQQSMLALPPEKKEYDVHLDENL-------------------------
ypest0001X_5890  ARPCDRW---LEEQQSMLALPPEKKEYDVHLDENL-------------------------
ypest0001X_4460  ARPCDRW---LEEQQSMLALPPEKKEYDVHLDENL-------------------------
ypest0001X_3710  ARPCDRW---LEEQQSMLALPPEKKEYDVHLDENL-------------------------
ypest0001X_2310  ARPCDRW---LEEQQSMLALPPEKKEYDVHLDENL-------------------------
ypest0001X_1670  ARPCDRW---LEEQQSMLALPPEKKEYDVHLDENL-------------------------
ypest0001X_1000  ARPCDRW---LEEQQSMLALPPEKKEYDVHLDENL-------------------------
ypest0001X_890   ARPCDRW---LEEQQSMLALPPEKKEYDVHLDENL-------------------------
ypest0001X_10    ARPCDRW---LEEQQSMLALPPEKKEYDVHLDENL-------------------------
                 #######   #########################                         


                        370       380       390       400       410       420
                 =========+=========+=========+=========+=========+=========+
ypseu0001X_140   ------------------------------------------------------------
ypest0001X_830   ------------------------------------------------------------
yente0001X_2473  -----------------------------------GYFIVKQFPIVKPTGRVTS------
ykris0001_3040   ----------------------------------------DRHE----------------
ypest0001X_3403  ELQHQRLMALAGQLQLESLISAAPALSQQAVDQEWSYMDFLEHLLHEEKLARHQRKQAMY
ypest0001X_2225  -------------------------------------VNFDKHPLH------HP------
ypest0001X_2950  -------------------------------------VNFDKHPLH------HP------
ypseu0001X_4293  -------------------------------------VNFDKHPLH------HP------
ypseu0001X_2061  -------------------------------------VNFDKHPLH------HP------
ypseu0001X_1809  -------------------------------------VNFDKHPLH------HP------
ypseu0001X_1304  -------------------------------------VNFDKHPLH------HP------
ypseu0001X_4150  -------------------------------------VNFDKHPLH------HP------
ypest0001X_4315  -------------------------------------VNFDKHPLH------HP------
ypest0001X_4080  -------------------------------------VNFDKHPLH------HP------
ypest0001X_4060  -------------------------------------VNFDKHPLH------HP------
ypest0001X_4005  -------------------------------------VNFDKHPLH------HP------
ypest0001X_3885  -------------------------------------VNFDKHPLH------HP------
ypest0001X_3836  -------------------------------------VNFDKHPLH------HP------
ypest0001X_3727  -------------------------------------VNFDKHPLH------HP------
ypest0001X_3562  -------------------------------------VNFDKHPLH------HP------
ypest0001X_3528  -------------------------------------VNFDKHPLH------HP------
ypest0001X_3487  -------------------------------------VNFDKHPLH------HP------
ypest0001X_3212  -------------------------------------VNFDKHPLH------HP------
ypest0001X_3093  -------------------------------------VNFDKHPLH------HP------
ypest0001X_3005  -------------------------------------VNFDKHPLH------HP------
ypest0001X_2922  -------------------------------------VNFDKHPLH------HP------
ypest0001X_2788  -------------------------------------VNFDKHPLH------HP------
ypest0001X_2665  -------------------------------------VNFDKHPLH------HP------
ypest0001X_2634  -------------------------------------VNFDKHPLH------HP------
ypest0001X_2441  -------------------------------------VNFDKHPLH------HP------
ypest0001X_2350  -------------------------------------VNFDKHPLH------HP------
ypest0001X_2288  -------------------------------------VNFDKHPLH------HP------
ypest0001X_2153  -------------------------------------VNFDKHPLH------HP------
ypest0001X_1996  -------------------------------------VNFDKHPLH------HP------
ypest0001X_1975  -------------------------------------VNFDKHPLH------HP------
ypest0001X_1936  -------------------------------------VNFDKHPLH------HP------
ypest0001X_1862  -------------------------------------VNFDKHPLH------HP------
ypest0001X_1756  -------------------------------------VNFDKHPLH------HP------
ypest0001X_1659  -------------------------------------VNFDKHPLH------HP------
ypest0001X_1562  -------------------------------------VNFDKHPLH------HP------
ypest0001X_1343  -------------------------------------VNFDKHPLH------HP------
ypest0001X_1298  -------------------------------------VNFDKHPLH------HP------
ypest0001X_1222  -------------------------------------VNFDKHPLH------HP------
ypest0001X_1127  -------------------------------------VNFDKHPLH------HP------
ypest0001X_1028  -------------------------------------VNFDKHPLH------HP------
ypest0001X_9850  -------------------------------------VNFDKHPLH------HP------
ypest0001X_8440  -------------------------------------VNFDKHPLH------HP------
ypest0001X_7760  -------------------------------------VNFDKHPLH------HP------
ypest0001X_7380  -------------------------------------VNFDKHPLH------HP------
ypest0001X_5890  -------------------------------------VNFDKHPLH------HP------
ypest0001X_4460  -------------------------------------VNFDKHPLH------HP------
ypest0001X_3710  -------------------------------------VNFDKHPLH------HP------
ypest0001X_2310  -------------------------------------VNFDKHPLH------HP------
ypest0001X_1670  -------------------------------------VNFDKHPLH------HP------
ypest0001X_1000  -------------------------------------VNFDKHPLH------HP------
ypest0001X_890   -------------------------------------VNFDKHPLH------HP------
ypest0001X_10    -------------------------------------VNFDKHPLH------HP------
                                                                             


                        430       440       450       460       470       480
                 =========+=========+=========+=========+=========+=========+
ypseu0001X_140   ------------------------------------------------------------
ypest0001X_830   ------------------------------------------------------------
yente0001X_2473  ------------------------------------------------------------
ykris0001_3040   ------------------------------------------------------------
ypest0001X_3403  TRMAAFPAVKTFEEYDFTFATGAPQKQLQSLRSLSFIERNENIVLLGPSGVGKTHLAIAM
ypest0001X_2225  ------------------------------------------------------------
ypest0001X_2950  ------------------------------------------------------------
ypseu0001X_4293  ------------------------------------------------------------
ypseu0001X_2061  ------------------------------------------------------------
ypseu0001X_1809  ------------------------------------------------------------
ypseu0001X_1304  ------------------------------------------------------------
ypseu0001X_4150  ------------------------------------------------------------
ypest0001X_4315  ------------------------------------------------------------
ypest0001X_4080  ------------------------------------------------------------
ypest0001X_4060  ------------------------------------------------------------
ypest0001X_4005  ------------------------------------------------------------
ypest0001X_3885  ------------------------------------------------------------
ypest0001X_3836  ------------------------------------------------------------
ypest0001X_3727  ------------------------------------------------------------
ypest0001X_3562  ------------------------------------------------------------
ypest0001X_3528  ------------------------------------------------------------
ypest0001X_3487  ------------------------------------------------------------
ypest0001X_3212  ------------------------------------------------------------
ypest0001X_3093  ------------------------------------------------------------
ypest0001X_3005  ------------------------------------------------------------
ypest0001X_2922  ------------------------------------------------------------
ypest0001X_2788  ------------------------------------------------------------
ypest0001X_2665  ------------------------------------------------------------
ypest0001X_2634  ------------------------------------------------------------
ypest0001X_2441  ------------------------------------------------------------
ypest0001X_2350  ------------------------------------------------------------
ypest0001X_2288  ------------------------------------------------------------
ypest0001X_2153  ------------------------------------------------------------
ypest0001X_1996  ------------------------------------------------------------
ypest0001X_1975  ------------------------------------------------------------
ypest0001X_1936  ------------------------------------------------------------
ypest0001X_1862  ------------------------------------------------------------
ypest0001X_1756  ------------------------------------------------------------
ypest0001X_1659  ------------------------------------------------------------
ypest0001X_1562  ------------------------------------------------------------
ypest0001X_1343  ------------------------------------------------------------
ypest0001X_1298  ------------------------------------------------------------
ypest0001X_1222  ------------------------------------------------------------
ypest0001X_1127  ------------------------------------------------------------
ypest0001X_1028  ------------------------------------------------------------
ypest0001X_9850  ------------------------------------------------------------
ypest0001X_8440  ------------------------------------------------------------
ypest0001X_7760  ------------------------------------------------------------
ypest0001X_7380  ------------------------------------------------------------
ypest0001X_5890  ------------------------------------------------------------
ypest0001X_4460  ------------------------------------------------------------
ypest0001X_3710  ------------------------------------------------------------
ypest0001X_2310  ------------------------------------------------------------
ypest0001X_1670  ------------------------------------------------------------
ypest0001X_1000  ------------------------------------------------------------
ypest0001X_890   ------------------------------------------------------------
ypest0001X_10    ------------------------------------------------------------
                                                                             


                        490       500       510       520       530       540
                 =========+=========+=========+=========+=========+=========+
ypseu0001X_140   ------------------------------------------------------------
ypest0001X_830   ------------------------------------------------------------
yente0001X_2473  ----------------------------LRI-----------------------------
ykris0001_3040   ------------------------------------------------------------
ypest0001X_3403  GYEAVRAGIKVRFTTAADLLLQLSTAQRQGRYKTTLQRGVMAPRLLIIDEIGYLPFSQEE
ypest0001X_2225  ----------------------------LSIYDSFSEEGVMMNCNIND------------
ypest0001X_2950  ----------------------------LSIYDSFC-RGVA-------------------
ypseu0001X_4293  ----------------------------LSIYDSFC-RGVA-------------------
ypseu0001X_2061  ----------------------------LSIYDSFC-RGVA-------------------
ypseu0001X_1809  ----------------------------LSIYDSFC-RGVA-------------------
ypseu0001X_1304  ----------------------------LSIYDSFC-RGVA-------------------
ypseu0001X_4150  ----------------------------LSIYDSFC-RGVA-------------------
ypest0001X_4315  ----------------------------LSIYDSFC-RGVA-------------------
ypest0001X_4080  ----------------------------LSIYDSFC-RGVA-------------------
ypest0001X_4060  ----------------------------LSIYDSFC-RGVA-------------------
ypest0001X_4005  ----------------------------LSIYDSFC-RGVA-------------------
ypest0001X_3885  ----------------------------LSIYDSFC-RGVA-------------------
ypest0001X_3836  ----------------------------LSIYDSFC-RGVA-------------------
ypest0001X_3727  ----------------------------LSIYDSFC-RGVA-------------------
ypest0001X_3562  ----------------------------LSIYDSFC-RGVA-------------------
ypest0001X_3528  ----------------------------LSIYDSFC-RGVA-------------------
ypest0001X_3487  ----------------------------LSIYDSFC-RGVA-------------------
ypest0001X_3212  ----------------------------LSIYDSFC-RGVA-------------------
ypest0001X_3093  ----------------------------LSIYDSFC-RGVA-------------------
ypest0001X_3005  ----------------------------LSIYDSFC-RGVA-------------------
ypest0001X_2922  ----------------------------LSIYDSFC-RGVA-------------------
ypest0001X_2788  ----------------------------LSIYDSFC-RGVA-------------------
ypest0001X_2665  ----------------------------LSIYDSFC-RGVA-------------------
ypest0001X_2634  ----------------------------LSIYDSFC-RGVA-------------------
ypest0001X_2441  ----------------------------LSIYDSFC-RGVA-------------------
ypest0001X_2350  ----------------------------LSIYDSFC-RGVA-------------------
ypest0001X_2288  ----------------------------LSIYDSFC-RGVA-------------------
ypest0001X_2153  ----------------------------LSIYDSFC-RGVA-------------------
ypest0001X_1996  ----------------------------LSIYDSFC-RGVA-------------------
ypest0001X_1975  ----------------------------LSIYDSFC-RGVA-------------------
ypest0001X_1936  ----------------------------LSIYDSFC-RGVA-------------------
ypest0001X_1862  ----------------------------LSIYDSFC-RGVA-------------------
ypest0001X_1756  ----------------------------LSIYDSFC-RGVA-------------------
ypest0001X_1659  ----------------------------LSIYDSFC-RGVA-------------------
ypest0001X_1562  ----------------------------LSIYDSFC-RGVA-------------------
ypest0001X_1343  ----------------------------LSIYDSFC-RGVA-------------------
ypest0001X_1298  ----------------------------LSIYDSFC-RGVA-------------------
ypest0001X_1222  ----------------------------LSIYDSFC-RGVA-------------------
ypest0001X_1127  ----------------------------LSIYDSFC-RGVA-------------------
ypest0001X_1028  ----------------------------LSIYDSFC-RGVA-------------------
ypest0001X_9850  ----------------------------LSIYDSFC-RGVA-------------------
ypest0001X_8440  ----------------------------LSIYDSFC-RGVA-------------------
ypest0001X_7760  ----------------------------LSIYDSFC-RGVA-------------------
ypest0001X_7380  ----------------------------LSIYDSFC-RGVA-------------------
ypest0001X_5890  ----------------------------LSIYDSFC-RGVA-------------------
ypest0001X_4460  ----------------------------LSIYDSFC-RGVA-------------------
ypest0001X_3710  ----------------------------LSIYDSFC-RGVA-------------------
ypest0001X_2310  ----------------------------LSIYDSFC-RGVA-------------------
ypest0001X_1670  ----------------------------LSIYDSFC-RGVA-------------------
ypest0001X_1000  ----------------------------LSIYDSFC-RGVA-------------------
ypest0001X_890   ----------------------------LSIYDSFC-RGVA-------------------
ypest0001X_10    ----------------------------LSIYDSFC-RGVA-------------------
                                                                             


                        550       560       570       580       590       600
                 =========+=========+=========+=========+=========+=========+
ypseu0001X_140   ------------------------------------------------------------
ypest0001X_830   ------------------------------------------------------------
yente0001X_2473  ------------------------------------------------------------
ykris0001_3040   ------------------------------------------------------------
ypest0001X_3403  AKLFFQVIAKRYEKSAMILTSNLPFGQWDQTFAGDAALTSAMLDRILHHSHVVQIKGESY
ypest0001X_2225  ------------------------------------------------------------
ypest0001X_2950  ------------------------------------------------------------
ypseu0001X_4293  ------------------------------------------------------------
ypseu0001X_2061  ------------------------------------------------------------
ypseu0001X_1809  ------------------------------------------------------------
ypseu0001X_1304  ------------------------------------------------------------
ypseu0001X_4150  ------------------------------------------------------------
ypest0001X_4315  ------------------------------------------------------------
ypest0001X_4080  ------------------------------------------------------------
ypest0001X_4060  ------------------------------------------------------------
ypest0001X_4005  ------------------------------------------------------------
ypest0001X_3885  ------------------------------------------------------------
ypest0001X_3836  ------------------------------------------------------------
ypest0001X_3727  ------------------------------------------------------------
ypest0001X_3562  ------------------------------------------------------------
ypest0001X_3528  ------------------------------------------------------------
ypest0001X_3487  ------------------------------------------------------------
ypest0001X_3212  ------------------------------------------------------------
ypest0001X_3093  ------------------------------------------------------------
ypest0001X_3005  ------------------------------------------------------------
ypest0001X_2922  ------------------------------------------------------------
ypest0001X_2788  ------------------------------------------------------------
ypest0001X_2665  ------------------------------------------------------------
ypest0001X_2634  ------------------------------------------------------------
ypest0001X_2441  ------------------------------------------------------------
ypest0001X_2350  ------------------------------------------------------------
ypest0001X_2288  ------------------------------------------------------------
ypest0001X_2153  ------------------------------------------------------------
ypest0001X_1996  ------------------------------------------------------------
ypest0001X_1975  ------------------------------------------------------------
ypest0001X_1936  ------------------------------------------------------------
ypest0001X_1862  ------------------------------------------------------------
ypest0001X_1756  ------------------------------------------------------------
ypest0001X_1659  ------------------------------------------------------------
ypest0001X_1562  ------------------------------------------------------------
ypest0001X_1343  ------------------------------------------------------------
ypest0001X_1298  ------------------------------------------------------------
ypest0001X_1222  ------------------------------------------------------------
ypest0001X_1127  ------------------------------------------------------------
ypest0001X_1028  ------------------------------------------------------------
ypest0001X_9850  ------------------------------------------------------------
ypest0001X_8440  ------------------------------------------------------------
ypest0001X_7760  ------------------------------------------------------------
ypest0001X_7380  ------------------------------------------------------------
ypest0001X_5890  ------------------------------------------------------------
ypest0001X_4460  ------------------------------------------------------------
ypest0001X_3710  ------------------------------------------------------------
ypest0001X_2310  ------------------------------------------------------------
ypest0001X_1670  ------------------------------------------------------------
ypest0001X_1000  ------------------------------------------------------------
ypest0001X_890   ------------------------------------------------------------
ypest0001X_10    ------------------------------------------------------------
                                                                             


                        610
                 =========+=======
ypseu0001X_140   -----------------
ypest0001X_830   -----------------
yente0001X_2473  -----------------
ykris0001_3040   -----------------
ypest0001X_3403  RLRQKRKAGVIAEANPE
ypest0001X_2225  -----------------
ypest0001X_2950  -----------------
ypseu0001X_4293  -----------------
ypseu0001X_2061  -----------------
ypseu0001X_1809  -----------------
ypseu0001X_1304  -----------------
ypseu0001X_4150  -----------------
ypest0001X_4315  -----------------
ypest0001X_4080  -----------------
ypest0001X_4060  -----------------
ypest0001X_4005  -----------------
ypest0001X_3885  -----------------
ypest0001X_3836  -----------------
ypest0001X_3727  -----------------
ypest0001X_3562  -----------------
ypest0001X_3528  -----------------
ypest0001X_3487  -----------------
ypest0001X_3212  -----------------
ypest0001X_3093  -----------------
ypest0001X_3005  -----------------
ypest0001X_2922  -----------------
ypest0001X_2788  -----------------
ypest0001X_2665  -----------------
ypest0001X_2634  -----------------
ypest0001X_2441  -----------------
ypest0001X_2350  -----------------
ypest0001X_2288  -----------------
ypest0001X_2153  -----------------
ypest0001X_1996  -----------------
ypest0001X_1975  -----------------
ypest0001X_1936  -----------------
ypest0001X_1862  -----------------
ypest0001X_1756  -----------------
ypest0001X_1659  -----------------
ypest0001X_1562  -----------------
ypest0001X_1343  -----------------
ypest0001X_1298  -----------------
ypest0001X_1222  -----------------
ypest0001X_1127  -----------------
ypest0001X_1028  -----------------
ypest0001X_9850  -----------------
ypest0001X_8440  -----------------
ypest0001X_7760  -----------------
ypest0001X_7380  -----------------
ypest0001X_5890  -----------------
ypest0001X_4460  -----------------
ypest0001X_3710  -----------------
ypest0001X_2310  -----------------
ypest0001X_1670  -----------------
ypest0001X_1000  -----------------
ypest0001X_890   -----------------
ypest0001X_10    -----------------
```

```
Parameters used
Minimum Number Of Sequences For A Conserved Position: 29
Minimum Number Of Sequences For A Flanking Position: 48
Maximum Number Of Contiguous Nonconserved Positions: 8
Minimum Length Of A Block: 10
Allowed Gap Positions: With Half
Use Similarity Matrices: Yes
```

```
Flank positions of the 5 selected block(s)
Flanks: [1  152]  [157  170]  [177  193]  [199  307]  [311  335]  

New number of positions in PGL1_unique_yersinia-CLUSTERS.dir/PGL1_unique_yersinia-CL10/PGL1_unique_yersinia-CL10.muscle.fasta.gblo:  317  (51% of the original 617 positions)
```
