# Supplementary material for: Genomic characterization of the Yersinia genus
Source: Genome Biol. 2010 Jan 4;11(1):R1. doi: 10.1186/gb-2010-11-1-r1 (PMC2847712; doi:10.1186/gb-2010-11-1-r1)
Supplement: Additional file 15 — The top level directory consists of a directory called Additional_cluster_files and 5010 directories, one for each multi-protein cluster family. (This top level directory has been split into three data files for uploading purposes (Additional files 15, 16, 17).) Within the directory are the following files: PGL1_unique_Yersinia_unclustered.out - list of all protein singletons that MCL did not group into a cluster (see Materials and Methods); PGL1_Yersinia_unique_locus_tags.txt - names of the 11 locus tag prefixes used for each genome; PGL1_unique_Yersinia.gff - mapping each Yersinia protein to a cluster in tab delimited GFF; PGL1_unique_Yersinia.sigfile - list of the longest protein in each cluster; PGL1_unique_Yersinia.summary - summary table of features of each of the clusters; PGL1_unique_Yersinia.table - summary table of each protein in the clusters. Within each cluster directory are the following files, where 'x' is the cluster name: PGL1_unique_Yersinia-x.faa - multifasta file of the proteins in the cluster; PGL1_unique_Yersinia-x.summary - summary of the properties of the proteins; PGL1_unique_Yersinia-x.matches - blast matches between the proteins of the cluster; PGL1_unique_Yersinia-x.muscle.fasta - muscle alignment of the proteins; PGL1_unique_Yersinia-x.muscle.fasta.gblo - gblocks output of muscle alignment (that is, auto-trimmed alignment); PGL1_unique_Yersinia-x.muscle.fasta.gblo.htm - as above in html format; PGL1_unique_Yersinia-x.muscle.tree - treefile from muscle alignment; PGL1_unique_Yersinia-x.sif - matches between proteins in simple interaction format for display on graphing software. [file gb-2010-11-1-r1-S15.zip › clusters/PGL1_unique_yersinia-CL100/PGL1_unique_yersinia-CL100.muscle.fasta.gblo.htm]

PGL1\_unique\_yersinia-CL100.muscle.fasta


## Gblocks 0.91b Results

Processed file: **PGL1\_unique\_yersinia-CL100.muscle.fasta**  
Number of sequences: **23**  
Alignment assumed to be: **Protein**  
New number of positions: **535** (selected positions are underlined in blue)

```
                         10        20        30        40        50        60
                 =========+=========+=========+=========+=========+=========+
ypest0001X_6140  -------------MLKF------IQNNREGT------ALLAILTLFALLGIIDRN-YFSL
ypseu0001X_6750  -------------MLKF------IQNNREGT------ALLAILTLFALLGIIDRN-YFSL
yinte0001_4020   -------------MLRF------IQNNREGT------ALLAILALFALLGVIDSN-YFTL
yaldo0001_3880   -------------MLKFIQSYQFIQNNREGT------ALLAILALFALLGVIDRN-YFNL
yente0001X_3794  -------------MLKFTQSYQFIQNNREGT------ALLAILALFALLGVIDRN-YFNL
yrohd0001_3510   -------------MLKF------IQNNREAT------ALLAILALFALLGVIDSN-YFSM
yfred0001_2930   -------------MLKF------IQNNREGT------ALLAILALFALLGVIDSN-YFSL
yruck0001_23680  --------------MKAARTGKLKRQEEQASFISWRFALLCGCILLALVGLMLRTAYLQV
ypseu0001X_8150  --------------MKTARPGKLKRQEEQASFISWRFALLCGCILLALVGLIMRTAYLQV
ypest0001X_7580  --------------MKTARPGKLKRQEEQASFISWRFALLCGCILLALVGLIMRTAYLQV
yaldo0001_5620   --------------MKAARPGKLKRQEEQASFISWRFALLCGCILLALVGLMLRTAYLQV
yfred0001_4390   --------------MKAARPGKLKRQEEQASFVSWRFALLCGCILLALVGLMLRTAYLQV
yente0001X_3654  --------------MKAARPGKLKRQEEQASFISWRFALLCGCILLALVGLMLRTAYLQV
ykris0001_30760  --------------MKAARPGKLKRQEEQASFISWRFALLCGCILLALVGLMLRTAYLQV
yinte0001_31860  --------------MKAARPGKLKRQEEQASFISWRFALLCGCILLALVGLMLRTAYLQV
yrohd0001_4760   --------------MKAARPGKLKRQEEQASFISWRFALLCGCILLALVGLMLRTAYLQV
ymoll0001_4420   --------------MKAARPGKLKRQEEQASFISWRFALLCGCILLALVGLMLRTAYLQV
yberc0001_4860   --------------MKAARPGKLKRQEEQASFISWRFALLCGCILLALIGLLLRTAYLQV
yruck0001_11350  --------------------MISKVNVETNNFIRWRFSLLCGCILLSLLGLLGRVAWLQV
yaldo0001_36760  ---------------------------------------------MSLAGLLARVAWLQV
yente0001X_2532  --------------------VISKPKNDSSSFIRWRFSLLCGCILLSFIGLIARVAWLQI
ykris0001_43150  MQWIGFNGCYNSCDHPGLISVISKPKNDSSNFIRWRFSLLCGGILLSFFGLLARVAWLQI
ymoll0001_14870  --------------------MISKPKNDSNNFIRWRFSLLCCSILMSFFGLIARVAWLQI
                                                      #######################


                         70        80        90       100       110       120
                 =========+=========+=========+=========+=========+=========+
ypest0001X_6140  QTFTMIFSSAQI--LILLAIGATLVMLTRN------IDVSVGSI----------TGLCAV
ypseu0001X_6750  QTFTMIFSSAQI--LILLAIGATLVMLTRN------IDVSVGSI----------TGLCAV
yinte0001_4020   QTFTMIFSSAQI--LILLAIGATMVMLTRN------IDVSVGSI----------TGLCAV
yaldo0001_3880   QTFTMIFSSAQI--LILLAIGATMVMLTRN------IDVSVGSI----------TGLCAV
yente0001X_3794  QTFTMIFSSAQI--LILLAIGATMVMLTRN------IDVSVGSI----------TGLCAV
yrohd0001_3510   QTFTMIFSSAQI--LILLAIGATMVMLTRN------IDVSVGSI----------TGLCAV
yfred0001_2930   QTFTMIFSSAQI--LILLAIGATMVMLTRN------IDVSVGSI----------TGLCAV
yruck0001_23680  INPDRLVREGDMRSLRVQAVPTARGMISDRAGRPLAVSVPVNAIWADPKELHDRGGITLD
ypseu0001X_8150  INPDKLVREGDMRSLRVQEVPTARGMISDRSGRPLAVSVPVNAVWADPKELIEQGGISLD
ypest0001X_7580  INPDKLVREGDMRSLRVQEVPTARGMISDRSGRPLAVSVPVNAVWADPKELIEQGGISLD
yaldo0001_5620   INPDRLVREGDMRSLRVQEVPTARGMISDRSGRPLAVSVPVNAVWADPKELIERGGITLD
yfred0001_4390   INPDRLVREGDMRSLRVQEVPTARGMISDRSGRPLAVSVPVNAVWADPKELIERGGITLD
yente0001X_3654  INPDKLVREGDMRSLRVQEVPTARGMISDRSGRPLAVSVPVNAVWADPKELTERGGITLD
ykris0001_30760  INPDKLVREGDMRSLRVQEVPTARGMISDRSGRPLAVSVPVNAVWADPKELTERGGITLD
yinte0001_31860  INPDKLVREGDMRSLRVQEVPTARGMISDRSGRPLAVSVPVNAVWADPKELTERGGITLD
yrohd0001_4760   INPDKLVREGDMRSLRVQEVPTARGMISDRSGRPLAVSVPVNAVWADPKELTERGGITLD
ymoll0001_4420   INPDKLVREGDMRSLRVQAVPTARGMISDRSGRPLAVSVPVNAVWADPKELTERGGITLD
yberc0001_4860   INPDKLVREGDMRSLRVQAVPTARGMISDRSGRPLAVSVPVNAVWADPKELTERGGITLD
yruck0001_11350  VEAESLAKEENLRSVRVMTTPNMRGMITDHNGQPLAVSVPVDAIWADPQVVLDKGGVGKG
yaldo0001_36760  IEPDPLVKEEDMRSLRVVVTQNSRGMITDRNGHPLAVSVPVEAIWADPKIVLEQGGAGNS
yente0001X_2532  IEPDPLVKEEDMRSVRVVATPNTRGMITDRNGHPLAVSVPVEAIWADPATVLEKGGVGVS
ykris0001_43150  IEPDPLVKEEDMRSVRVVTTPNTRGMITDRNGHPLAVSVPVEAIWADPSTVLDKGGVSVS
ymoll0001_14870  IEPDPLVKEEDMRSVRVVETPNTRGMITDRNEHPLAVSVPVAAVWADPQTVLERGGVGVS
                 ############################################################


                        130       140       150       160       170       180
                 =========+=========+=========+=========+=========+=========+
ypest0001X_6140  TVGMALNAGFGLAASCLFALLVGMVAGFF----------NGILVTWLRIPAI--------
ypseu0001X_6750  TVGMALNAGFGLVASCLFALLVGMVAGFF----------NGILVTWLRIPAI--------
yinte0001_4020   TVGMALNAGFGLATSCLFALLVGMVTGFF----------NGILVTWLRIPAI--------
yaldo0001_3880   TVGMALNAGFGLALSCLFALLVGMGAGFF----------NGILVTWLRIPAI--------
yente0001X_3794  TVGMALNAGFGLALSCLFALLVGMGAGFF----------NGILVTWLRIPAI--------
yrohd0001_3510   TVGMALNAGFGLALSCLFALLVGMMTGFF----------NGILVTWLRIPAI--------
yfred0001_2930   TVGMALNAGFGLALSCLFALLVGMLAGFF----------NGILVTWLRIPAI--------
yruck0001_23680  TRWKALSDALGVPLDQLVSRINANPKGRFVYLARQVNPAIGDYIHKLKLPGIYLRQESRR
ypseu0001X_8150  TRWKALSDALEIPLDQLATRINANPKGRFVYLARQVNPAIGDYIRKLKLPGIHLRQESRR
ypest0001X_7580  TRWKALSDALEIPLDQLATRINANPKGRFVYLARQVNPAIGDYIRKLKLPGIHLRQESRR
yaldo0001_5620   TRWKALSDALEIPLDQLATRINANPKGRFVYLARQVNPAIGDYIHKLKLPGIYLRQESRR
yfred0001_4390   TRWKALSDALEIPLDQLATRINANPKGRFVYLARQVNPAIGDYIHKLKLPGIYLRQESRR
yente0001X_3654  TRWKALSDALEIPLDQLATRINANPKGRFVYLARQVNPAIGDYIHKLKLPGIYLRQESRR
ykris0001_30760  TRWKALSDALEIPLDQLAARINANPKGRFVYLARQVNPAIGDYIHKLKLPGIYLRQESRR
yinte0001_31860  TRWKALSDALEIPLDQLATRINANPKGRFVYLARQVNPAIGDYIHKLKLPGIYLRQESRR
yrohd0001_4760   TRWKALSDALDIPLDQLATRINANPKGRFVYLARQVNPAIGDYIHKLKLPGIYLRQESRR
ymoll0001_4420   TRWKALSDALEIPLDQLATRINANPKGRFVYLARQVNPAIGDYIHKLKLPGIYLRQESRR
yberc0001_4860   TRWKALSDALEIPLDQLATRINANPKGRFVYLARQVNPAIGDYIHKLKLPGIYLRQESRR
yruck0001_11350  PRWQALAQTLHQSLTEIASRINQSPTGRFVYLARQVEPTSADYIQRLKLPGIALRAESRR
yaldo0001_36760  ARWQALAQALNLPLDHLINRINQAPAARFIYLARQVEPDVAEYIQGLKLPGIATKAESRR
yente0001X_2532  ERWQALAQELNIPLDQLNSRIHQNPHARFIYLARQVEPDVAEYIQRLKLPGIAIKEESRR
ykris0001_43150  ARWQALAQELNMPLDQLNHRIHQNPGARFIYLARQVEPDVAEYIQRLKLPGIAIKEESRR
ymoll0001_14870  ERWQALAQALNMPLDQVSSRITQIPHARFIYLARQVEPNVAEYIQRLKLPGIATKVESRR
                 ############################################################


                        190       200       210       220       230       240
                 =========+=========+=========+=========+=========+=========+
ypest0001X_6140  ----------------VATLGTLGLYRGLMLLLTG------------GKWIEGLPAD---
ypseu0001X_6750  ----------------VATLGTLGLYRGLMLLLTG------------GKWIEGLPAD---
yinte0001_4020   ----------------VATLGTLGLYRGLMLLITG------------GKWIEGLPAD---
yaldo0001_3880   ----------------VATLGTLGLYRGLMLLLTG------------GKWIEGLPAD---
yente0001X_3794  ----------------VATLGTLGLYRGLMLLLTG------------GKWIEGLPAD---
yrohd0001_3510   ----------------VATLGTLGLYRGLMLLLTG------------GKWIEGLPAD---
yfred0001_2930   ----------------VATLGTLGLYRGLMLLLTG------------GKWIEGLPAD---
yruck0001_23680  YYPAGQVMAHIIGVTNIDSQGIEGVEKSFDRWLTGQPGERTVRKDRYGRVIEDISSVDSQ
ypseu0001X_8150  YYPAGQVMAHIIGVTNIDGQGIEGVEKSFDRWLTGQPGERTVRKDRYGRVIEDISSVDSQ
ypest0001X_7580  YYPAGQVMAHIIGVTNIDGQGIEGVEKSFDRWLTGQPGERTVRKDHYGRVIEDISSVDSQ
yaldo0001_5620   YYPAGQVMAHIIGVTNIDGQGIEGVEKSFDRWLTGQPGERTVRKDRFGRVIEDISSVDSQ
yfred0001_4390   YYPAGQVMAHIIGVTNIDSQGIEGVEKSFDRWLTGQPGERTVRKDRYGRVIEDISSVDSQ
yente0001X_3654  YYPAGQVMAHIIGVTNIDSQGIEGVEKSFDRWLTGQPGERTVRKDRYGRVIEDISSVDSQ
ykris0001_30760  YYPAGQVMAHIIGVTNIDSQGIEGVEKSFDRWLTGQPGERTVRKDRYGRVIEDISSVDSQ
yinte0001_31860  YYPAGQVMAHIIGVTNIDGQGIEGVEKSFDRWLTGQPGERTVRKDRYGRVIEDISSVDSQ
yrohd0001_4760   YYPAGQVMAHIIGVTNIDSQGIEGVEKSFDRWLTGQPGERTVRKDRYGRVIEDISSVDSQ
ymoll0001_4420   YYPAGQVMAHIIGVTNIDSQGIEGVEKSFDRWLTGQPGERTVRKDRYGRVIEDISSVDSQ
yberc0001_4860   YYPAGQVMAHIIGVTNIDSQGIEGVEKSFDRWLTGQPGERTVRKDRYGRVIEDISSVDSQ
yruck0001_11350  FYPAGATVANLVGFTNIDDQGIEGIEKSFNQLLSGTAGHRVVRKDRYGHVVEDISATDSH
yaldo0001_36760  FYPAGDIAANLVGFTNIDDQGIEGVEKSFNSLLSGTAGSRVVRKDRFGRVVEDISSTDSH
yente0001X_2532  FYPSGDIAANLVGFTNIDDQGIEGVEKSFNTLLSGTAGSRVVRKDRFGRVVEDISSTDSH
ykris0001_43150  FYPSGDIAANLVGFTNIDDQGIEGVEKSFNSLLSGTAGSRVVRKDRFGRVVEDISSTDSH
ymoll0001_14870  FYPSGDIAANLVGFTNIDDQGIEGVEKSFNSLLSGTAGSRVVRKDRFGRVIEDISSTDSH
                 ############################################################


                        250       260       270       280       290       300
                 =========+=========+=========+=========+=========+=========+
ypest0001X_6140  -----------------LKSLSTPILFSISPIGWLTMLLILAMAWLLGKTAFGRSFYATG
ypseu0001X_6750  -----------------LKSLSTPILFSISPIGWLTMLLILSMAWLLGNTAFGRSFYATG
yinte0001_4020   -----------------LKSLSTPILFSISPIGWLIMLLIVAMALLLGKTAFGRSFYATG
yaldo0001_3880   -----------------LKSLSTPILFSISPIGWLIMLLIVAMALLLGKTAFGRSFYATG
yente0001X_3794  -----------------LKSLSTPILFSISPIGWLIMLLIVAMALLLGKTAFGRSFYATG
yrohd0001_3510   -----------------LKSLSTPILFSISPIGWLIMLLIVAMALLLGKTSFGRSFYATG
yfred0001_2930   -----------------LKSLSTPILFSISPIGWLIMLLIIAMALLLGKTAFGRSIYATG
yruck0001_23680  AAHNLVLSVDERLQALVYRELNNAVAFNKAESGTAVLVDVSTGEVL----AMANSPSYNP
ypseu0001X_8150  AAHNLVLSVDERLQALVYRELNNAVAFNKAESGTAVLVDVNTGEVL----AMANSPSYNP
ypest0001X_7580  AAHNLVLSVDERLQALVYRELNNAVAFNKAESGTAVLVDVNTGEVL----AMANSPSYNP
yaldo0001_5620   AAHNLVLSVDERLQALVYRELNNAVAFNKAESGTAVLVDVTTGEVL----AMANSPSYNP
yfred0001_4390   AAHNLVLSVDERLQALVYRELNNAVAFNKAESGTAVLVDVNTGEVL----AMANSPSYNP
yente0001X_3654  AAHNLVLSVDERLQALVYRELNNAVAFNKAESGTAVLVDVNTGEVL----AMANSPSYNP
ykris0001_30760  AAHNLVLSVDERLQALVYRELNNAVAFNKAESGTAVLVDVNTGEVL----AMANSPSYNP
yinte0001_31860  AAHNLVLSVDERLQALVYRELNNAVAFNKAESGTAVLVDVSTGEVL----AMANSPSYNP
yrohd0001_4760   AAHNLVLSVDERLQALVYRELNNAVAFNKAESGTAVLVDVNTGEVL----AMANSPSYNP
ymoll0001_4420   AAHNLVLSVDERLQALVYRELNNAVAFNKAESGTAVLVDVNTGEVL----AMANSPSYNP
yberc0001_4860   AAHNLVLSVDERLQALVYRELNNAVAFNKAESGTAVLVDVNTGEVL----AMANSPSYNP
yruck0001_11350  PGQNVQLSIDERLQTEASHALTNAVLFNKAESGSAVLVDVNTGEVL----AMANYPTYNP
yaldo0001_36760  PAQNVQLSIDERLQAEASHALTNAVMFNKADSGSAIVIDVNTGEVL----AMANYPLFNP
yente0001X_2532  PGQNVQLSIDERLQTEASHALTNAVIFNKADSGSAVVIDVNTGEVL----AMANYPTFNP
ykris0001_43150  PGQNVELSIDERLQAETSHALSNAVMFNKADSGSAVVIDVNTGEIL----AMANYPTFNP
ymoll0001_14870  PGQRVELSIDERLQAETSHALTNAVMFNKADSGSAVVIDVNTGEVL----AMANYPTFNP
                 ##############################################    ##########


                        310       320       330       340       350       360
                 =========+=========+=========+=========+=========+=========+
ypest0001X_6140  DNLQGARQLGVRTDSLR-IF------------------AFSMNGVMAALAGIVFASQI--
ypseu0001X_6750  DNLQGARQLGVRTDSIR-IF------------------AFSMNGVMAALAGIVFASQI--
yinte0001_4020   DNLQGARQLGIRTDSIR-IF------------------AFSMNGVMAALAGIVFASQI--
yaldo0001_3880   DNLQGARQLGIRTDSIR-IF------------------AFSMNGVMAALAGIVFASQI--
yente0001X_3794  DNLQGARQLGIRTDSIR-IF------------------AFSMNGVMAALAGIVFASQI--
yrohd0001_3510   DNLQGARQLGIRTDSIR-IF------------------AFSMNGVMAALAGIVFASQI--
yfred0001_2930   DNLQGARQLGIRTDSIR-IF------------------AFSMNGVMAALAGIVFASQI--
yruck0001_23680  NNLTGTPKEAMRNRAITDIFEPGSTVKPMVVMTALQHGVVKENSVLNTLPYSISGHQIKD
ypseu0001X_8150  NNLTGTPKDAMRNRAITDIFEPGSTVKPMVVMTALQHGVVKENSVLNTLPYFVNGHQIKD
ypest0001X_7580  NNLTGTPKDAMRNRAITDIFEPGSTVKPMVVMTALQHGVVKENSVLNTLPYFVNGHQIKD
yaldo0001_5620   NNLTGTPKDAMRNRAITDIFEPGSTVKPMVVMTALQNGVVKENSVLNTLPYFVNGHQIKD
yfred0001_4390   NNLTGTPKDAMRNRAITDIFEPGSTVKPMVVMTALQHGVVKENSVLNTLPYFVNGHQIKD
yente0001X_3654  NNLTGTPKDAMRNRAITDIFEPGSTVKPMVVMTALQHGVVKENSVLNTLPYFVNGHQIKD
ykris0001_30760  NNLTGTPKDAMRNRAITDIFEPGSTVKPMVVMTALQHGVVKENSVLNTLPYFVNGHQIKD
yinte0001_31860  NNLTGTPKDAMRNRAITDIFEPGSTVKPMVVMTALQHGVVKENSVLNTLPYFVNGHQIKD
yrohd0001_4760   NNLTGTPKDAMRNRAITDIFEPGSTVKPMVVMTALQHGVVKENSVLNTLPYFVNGHQIKD
ymoll0001_4420   NNLTGTPKDAMRNRAITDIFEPGSTVKPMVVMTALQHGVVKENSVLNTLPYFVNGHQIKD
yberc0001_4860   NNLTGTPKDAMRNRAITDIFEPGSTVKPMVVMTALQHGVVKENSVLNTLPYFVNGHQIKD
yruck0001_11350  NNRADTPTENFRNRAISDIFEPGSTVKPMVIMTALQRKLVKPDSVLDTHPYTLSGHQIRD
yaldo0001_36760  NNRAATPEENFRNRAISDIFEPGSTVKPMVVMTALQRHLVQPDTVLDTHPYILSGHQIKD
yente0001X_2532  NNRVGTPEENFRNRAISDIFEPGSTVKPMVVMTALERHIVKPDAVLDTHPYILSGHLIKD
ykris0001_43150  NNRAGTPEENFRNRAISDIFEPGSTVKPMVVMTALQRHLVKPDAVLDTRPYILSGHLIKD
ymoll0001_14870  NNRADTPEENFRNRAISDIFEPGSTVKPMVVMTALQRHLVKPDTVLDTHPYILSGHQIKD
                 ############################################################


                        370       380       390       400       410       420
                 =========+=========+=========+=========+=========+=========+
ypest0001X_6140  ----------GFIPNQTGNGLEMKAIAACV-----------LGGISLLGGTGTIIG----
ypseu0001X_6750  ----------GFIPNQTGNGLEMKAIAACV-----------LGGISLLGGTGTIIG----
yinte0001_4020   ----------GFIPNQTGSGLEMKAIAACV-----------LGGISLLGGTGTILG----
yaldo0001_3880   ----------GFIPNQTGSGLEMKAIAACV-----------LGGISLLGGTGTIIG----
yente0001X_3794  ----------GFIPNQTGSGLEMKAIAACV-----------LGGISLLGGTGTIIG----
yrohd0001_3510   ----------GFIPNQTGSGLEMKAIAACV-----------LGGISLLGGTGTIIG----
yfred0001_2930   ----------GFIPNQTGNGLEMKAIAACV-----------LGGISLLGGTGTIIG----
yruck0001_23680  VARYAELSVTGILQKSSNVGVSKLALAMPSSALVDTYSRFGFGKATNLGLVGESSGLYPK
ypseu0001X_8150  VARYAELSVTGILQKSSNVGVSKLALAMPSSALVDTYSRFGFGKATNLGLVGESSGLYPK
ypest0001X_7580  VARYAELSVTGILQKSSNVGVSKLALAMPSSALVDTYSRFGFGKATNLGLVGESSGLYPK
yaldo0001_5620   VARYAELSVTGILQKSSNVGVSKLALAMPSSALVETYLKFGFGKATNLGLVGESSGLYPK
yfred0001_4390   VARYAELSVTGILQKSSNVGVSKLALAMPSSALVDTYSRFGFGKATNLGLVGESSGLYPK
yente0001X_3654  VARYAELSVTGILQKSSNVGVSKLALAMPSSALVETYSKFGFGKATNLGLVGESSGLYPK
ykris0001_30760  VARYAELSVTGILQKSSNVGVSKLALAMPSSALVETYSKFGFGKATNLGLVGESSGLYPK
yinte0001_31860  VARYAELSVTGILQKSSNVGVSKLALAMPSSALVDTYSRFGFGKATNLGLVGESSGLYPK
yrohd0001_4760   VARYAELSVTGILQKSSNVGVSKLALAMPSSALVDTYSRFGFGKATNLGLVGESSGLYPK
ymoll0001_4420   VARYAELSVTGILQKSSNVGVSKLALAMPSSALVDTYSRFGFGKATNLGLVGESSGLYPK
yberc0001_4860   VARYAELSVTGILQKSSNVGVSKLALAMPSSALVDTYSRFGFGKATNLGLVGESSGLYPK
yruck0001_11350  VGFYPTLSLTGVLQKSSDVGVSRLALAMPASALLQTYGLFGLGKPTELGLTGESSGLMPQ
yaldo0001_36760  VALYPALSLTGVLQKSSDVGVSRLALAMPASALIQTYSLFGLGKPTQLGLTGESRGLMPH
yente0001X_2532  VALYPALSLTGVLQKSSDVGVSRLALAMPASALMQTYSAFGLGKPTQLGLTGESSGLMPH
ykris0001_43150  VALYPALSLTGVLQKSSDVGVSRLALAMPASALMQTYTEFGLGQSTQLGLTGESSGLMPH
ymoll0001_14870  VGFYPALSLTGVLQKSSDVGVSRLALAMPASALMETYSLFGLGKPTQLGLTGESSGLMPH
                 ############################################################


                        430       440       450       460       470       480
                 =========+=========+=========+=========+=========+=========+
ypest0001X_6140  -----------------------------AILGAF------LLTQIDSVLV--------L
ypseu0001X_6750  -----------------------------AILGAF------LLTQIDSVLV--------L
yinte0001_4020   -----------------------------AILGAY------LLTQIDSVLV--------L
yaldo0001_3880   -----------------------------AILGAY------LLTQIDSVLV--------L
yente0001X_3794  -----------------------------AILGAY------LLTQIDSVLV--------L
yrohd0001_3510   -----------------------------AILGAY------LLTQIDSVLV--------L
yfred0001_2930   -----------------------------AILGAY------LLTQIDSVLV--------L
yruck0001_23680  KQRWSDIERATFSYGYGLMVTPLQLARVYATIGSLGIYRPLSITKVDPPVAGERIFPESI
ypseu0001X_8150  KQRWSDIERATFSFGYGLMVTPLQLARVYATIGSMGVYRPLSITRVDPPVAGERIFPEPL
ypest0001X_7580  KQRWSDIERATFSFGYGLMVTPLQLARVYATIGSMGVYRPLSITRVDPPVAGERIFPEPL
yaldo0001_5620   KQRWSDIERATFSFGYGLMVTPLQLARVYATIGSMGIYRPLSITKVDPPVAGERVFPEPL
yfred0001_4390   KQRWSDIERATFSFGYGLMVTPLQLARVYATIGSMGIYRPLSITKVDPPVAGERVFPEPL
yente0001X_3654  KQRWSDIERATFSFGYGLMVTPLQLARVYATIGSMGIYRPLSITKVDPPVAGERVFPEPL
ykris0001_30760  KQRWSDIERATFSFGYGLMVTPLQLARVYATIGSMGIYRPLSITKVDPPVAGERVFPEPL
yinte0001_31860  KQRWSDIERATFSFGYGLMVTPLQLARVYATIGSMGIYRPLSITKVDPPVAGERIFPEPL
yrohd0001_4760   KQRWSDIERATFSFGYGLMVTPLQLARVYATIGSMGIYRPLSITKVDPPVAGERIFPEPL
ymoll0001_4420   KQRWSDIERATFSFGYGLMVTPLQLARVYATIGSMGIYRPLSITKVDPPVAGERIFPEPL
yberc0001_4860   KQRWSDIERATFSFGYGLMVTPLQLARVYATIGSMGIYRPLSITKVDPPVAGERIFPEPL
yruck0001_11350  RQRWSDLDRATFSFGYGLMVTPLQLARVYATIGSYGIYRPLSITKVDPPVVGHRVMLAER
yaldo0001_36760  RQRWSDLDRATFSFGYGLMVTPLQLARVYATIGSFGIYRPLSITKVDPPVLGTRVFPQAL
yente0001X_2532  RQRWSDLDRATFSFGYGLMVTPLQLARVYATIGSFGIYRPLSITKVDPPVLGQRIFPEEL
ykris0001_43150  RQRWSDLDRATFSFGYGLMVTPLQLARVYATIGSFGIYRPLSITKVDPPVLGKRVFPEVL
ymoll0001_14870  RQRWSDLDRATFSFGYGLMVTPLQLARVYATIGSFGIYRPLSITKVDPPVLGQRVFPEGE
                 ############################################################


                        490       500       510       520       530       540
                 =========+=========+=========+=========+=========+=========+
ypest0001X_6140  LRLPAWWNDFIA----GLVLLGVLVFDGRLRCAVERNIRQ-----QKYARFTAQAIISDK
ypseu0001X_6750  LRLPAWWNDFIA----GLVLLGVLVFDGRLRCAVERNIRQ-----QKYARFTAQAIISDK
yinte0001_4020   LRLPAWWNDFIA----GLVLLGVLVFDGRLRCAIERNIRQ-----QKYARFTARPAVSGK
yaldo0001_3880   LRLPAWWNDFIA----GLVLLGVLVFDGRLRCAVERNIRQ-----QKYARFTTRPVASDK
yente0001X_3794  LRLPAWWNDFIA----GLVLLGVLVFDGRLRCAVERNIRQ-----QKYARFTTRPVAPDK
yrohd0001_3510   LRLPAWWNDFIA----GLVLLGVLVFDGRLRCAVERNIRQ-----QKYARFATRPVKDNK
yfred0001_2930   LRLPAWWNDFIA----GLVLLGVLVFDGRLRCAVERNLRQ-----QKYARFSTHPVRDDK
yruck0001_23680  VRTVVHMMESVALPGGGGTKAAIKGYRIAIKTGTAKKVGPDGKYVNRYLAYTAGVAPASN
ypseu0001X_8150  VRTVVHMMESVALPGGGGTKAAIKGYRIAIKTGTAKKVGPDGKYMDRYLAYTAGVAPASN
ypest0001X_7580  VRTVVHMMESVALPGGGGTKAAIKGYRIAIKTGTAKKVGPDGKYMDRYLAYTAGVAPASN
yaldo0001_5620   VRTVVHMMESVALPGGGGTKAAIKGYRIAIKTGTAKKVGPDGKYMDRYLAYTAGVAPASN
yfred0001_4390   VRTVVHMMESVALPGGGGTKAAIKGYRIAIKTGTAKKVGPDGKYIDRYLAYTAGVAPASN
yente0001X_3654  VRTVVHMMESVALPGGGGTKAAIKGYRIAIKTGTAKKVGPDGKYMDRYLAYTAGVAPASN
ykris0001_30760  VRTVVHMMESVALPGGGGTKAAIKGYRIAIKTGTAKKVGPDGKYMDRYLAYTAGVAPASN
yinte0001_31860  VRTVVHMMESVALPGGGGTKAAIKGYRIAIKTGTAKKVGPDGKYMDRYLAYTAGVAPASN
yrohd0001_4760   VRTVVHMMESVALPGGGGTKAAIKGYRIAIKTGTAKKVGPDGKYMDRYLAYTAGVAPASN
ymoll0001_4420   VRTVVHMMESVALPGGGGTKAAIKGYRIAIKTGTAKKVGPDGKYMDRYLAYTAGVAPASN
yberc0001_4860   VRTVVHMMESVALPGGGGTKAAIKGYRIAIKTGTAKKVGPDGKYMDRYLAYTAGVAPASN
yruck0001_11350  VKEVEYMMESVALPGGGGVKAAVRGYRVAVKTGTAKKIGPNGQYIDKYVAYTAGVAPASQ
yaldo0001_36760  VRQVEHMMESVALPGGGGIKAAVRGYRVAVKTGTAKKIGPNGQYVNKYIAYTAGVAPASQ
yente0001X_2532  VRQVEHMMESVALPGGGGVKAAVRGYRVAVKTGTAKKIGPNGQYIDKYVAYTAGVAPASQ
ykris0001_43150  VRQVEHMMESVALPGGGGVKAAVRGYRVAVKTGTAKKIGPNGQYIDKYVAYTAGVAPASQ
ymoll0001_14870  VREVEHMMESVALPGGGGTKAAVRGYRVAIKTGTAKKIGPNGQYIDKYVAYTAGVAPASQ
                 ############################################################


                        550       560       570       580       590       600
                 =========+=========+=========+=========+=========+=========+
ypest0001X_6140  KPTV----------------SDNNPAASNKKKAAL-------------------------
ypseu0001X_6750  KPTV----------------SDNNPAASNKKKAAL-------------------------
yinte0001_4020   P-----TTSDKKK-------NVIPNRFHDAGRRQTRESR---------------------
yaldo0001_3880   KSAAAPSKTPDSK-------APNGKSFT--KKEVAR------------------------
yente0001X_3794  K-----VKSNNNK-------APSSKSFT--KKEVVR------------------------
yrohd0001_3510   KKNISNTSSSDKP-------VNNSKPFNKIKKEVAR------------------------
yfred0001_2930   KKKTNSNKSSNDKS------VNNNKPFNKIKKEVAR------------------------
yruck0001_23680  PRFALVVVIDDPQGGKYYGGAVSAPVFGAIMGGVLRTMNIEPDALPTVDKSELVTNTKEG
ypseu0001X_8150  PRFALVVVINDPQAGKYYGGAVSAPVFGAIMGGVLRTMNIEPDALPTGDKSELVINTKEG
ypest0001X_7580  PRFALVVVINDPQAGKYYGGAVSAPVFGAIMGGVLRTMNIEPDALPTGDKSELVINTKEG
yaldo0001_5620   PRFALVVVINDPQAGKYYGGAVSAPVFGAIMGGVLRTMNIEPDGLPTGDKSELVINTKEG
yfred0001_4390   PRFALVVVINDPQAGKYYGGAVSAPVFGAIMGGVLRTMNIEPDALPTGDKSELVINTKEG
yente0001X_3654  PRFALVVVINDPQAGKYYGGAVSAPVFGAIMGGVLRTMNIEPDALPTGDKSELVTNTKEG
ykris0001_30760  PRFALVVVINDPQAGKYYGGAVSAPVFGAIMGGVLRTMNIEPDALPTGDKSELVTNTKEG
yinte0001_31860  PRFALVVVINDPQAGKYYGGAVSAPVFGAIMGGVLRTMNIEPDALPTGDKSELVINTKEG
yrohd0001_4760   PRFALVVVINDPQAGKYYGGAVSAPVFGAIMGGVLRTMNIEPDALPTGDKSELVINTKEG
ymoll0001_4420   PRFALVVVINDPQAGKYYGGAVSAPVFGAIMGGVLRTMNIEPDALPTGDKSELVINTKEG
yberc0001_4860   PRFALVVVINDPQAGKYYGGAVSAPVFGAIMGGVLRTMNIEPDALPTGDKSELVINTKEG
yruck0001_11350  PRFALVVVINNPQGGKYYGGAVAAPVFSEIMGEILRTMNVEPDALPTQALR---------
yaldo0001_36760  PRFALVVVINNPQGGKYYGGAVSAPVFSDIMGQILRTMNVAPDAIPSDGTR---------
yente0001X_2532  PRFALVVVINNPQAGKYYGGAVSAPVFSDIMGQILRTMNVEPDAIPVNVIR---------
ykris0001_43150  PRFALVVVINNPQGGKYYGGAVSAPVFSDIMGQILRTMNVEPDAIPVNMIR---------
ymoll0001_14870  PRFALVVVINNPQGGKYYGGAVSAPVFSDIMGQILRTMNVEPDAIPTGDTR---------
                 ####################################                        


                 
                 =====
ypest0001X_6140  -----
ypseu0001X_6750  -----
yinte0001_4020   -----
yaldo0001_3880   -----
yente0001X_3794  -----
yrohd0001_3510   -----
yfred0001_2930   -----
yruck0001_23680  SGGRS
ypseu0001X_8150  SGGRS
ypest0001X_7580  SGGRS
yaldo0001_5620   SGGRS
yfred0001_4390   SGGRS
yente0001X_3654  SGGRS
ykris0001_30760  SGGRS
yinte0001_31860  SGGRS
yrohd0001_4760   SGGRS
ymoll0001_4420   SGGRS
yberc0001_4860   SGGRS
yruck0001_11350  ---HS
yaldo0001_36760  ---HS
yente0001X_2532  ---HS
ykris0001_43150  ---HS
ymoll0001_14870  ---HS
```

```
Parameters used
Minimum Number Of Sequences For A Conserved Position: 12
Minimum Number Of Sequences For A Flanking Position: 19
Maximum Number Of Contiguous Nonconserved Positions: 8
Minimum Length Of A Block: 10
Allowed Gap Positions: With Half
Use Similarity Matrices: Yes
```

```
Flank positions of the 2 selected block(s)
Flanks: [38  286]  [291  576]  

New number of positions in PGL1_unique_yersinia-CLUSTERS.dir/PGL1_unique_yersinia-CL100/PGL1_unique_yersinia-CL100.muscle.fasta.gblo:  535  (88% of the original 605 positions)
```
