# Supplementary material for: Genomic characterization of the Yersinia genus
Source: Genome Biol. 2010 Jan 4;11(1):R1. doi: 10.1186/gb-2010-11-1-r1 (PMC2847712; doi:10.1186/gb-2010-11-1-r1)
Supplement: Additional file 15 — The top level directory consists of a directory called Additional_cluster_files and 5010 directories, one for each multi-protein cluster family. (This top level directory has been split into three data files for uploading purposes (Additional files 15, 16, 17).) Within the directory are the following files: PGL1_unique_Yersinia_unclustered.out - list of all protein singletons that MCL did not group into a cluster (see Materials and Methods); PGL1_Yersinia_unique_locus_tags.txt - names of the 11 locus tag prefixes used for each genome; PGL1_unique_Yersinia.gff - mapping each Yersinia protein to a cluster in tab delimited GFF; PGL1_unique_Yersinia.sigfile - list of the longest protein in each cluster; PGL1_unique_Yersinia.summary - summary table of features of each of the clusters; PGL1_unique_Yersinia.table - summary table of each protein in the clusters. Within each cluster directory are the following files, where 'x' is the cluster name: PGL1_unique_Yersinia-x.faa - multifasta file of the proteins in the cluster; PGL1_unique_Yersinia-x.summary - summary of the properties of the proteins; PGL1_unique_Yersinia-x.matches - blast matches between the proteins of the cluster; PGL1_unique_Yersinia-x.muscle.fasta - muscle alignment of the proteins; PGL1_unique_Yersinia-x.muscle.fasta.gblo - gblocks output of muscle alignment (that is, auto-trimmed alignment); PGL1_unique_Yersinia-x.muscle.fasta.gblo.htm - as above in html format; PGL1_unique_Yersinia-x.muscle.tree - treefile from muscle alignment; PGL1_unique_Yersinia-x.sif - matches between proteins in simple interaction format for display on graphing software. [file gb-2010-11-1-r1-S15.zip › clusters/PGL1_unique_yersinia-CL1000/PGL1_unique_yersinia-CL1000.muscle.fasta.gblo.htm]

PGL1\_unique\_yersinia-CL1000.muscle.fasta


## Gblocks 0.91b Results

Processed file: **PGL1\_unique\_yersinia-CL1000.muscle.fasta**  
Number of sequences: **11**  
Alignment assumed to be: **Protein**  
New number of positions: **71** (selected positions are underlined in blue)

```
                         10        20        30        40        50        60
                 =========+=========+=========+=========+=========+=========+
ypseu0001X_2000  VWCLAEQGWIATRPNIE-VSMKQGIHPKYEQVTASCSCGNVIKINSTVGHDLNLDVCGEC
ypest0001X_3090  VWCLAEQGWIATRPNIE-VSMKQGIHPKYEQVTASCSCGNVIKINSTVGHDLNLDVCGEC
yente0001X_4209  --------------------MKQGIHPKYDLVTASCSCGNVIKINSTVGHDLNLDVCGEC
ykris0001_930    --------------------MKKGIHPKYDLVTASCSCGNVIKINSTVGHDLNLDVCGEC
yrohd0001_930    VWCLAEQGWIATRPKYRGFSMKKGIHPKYDLVTASCSCGNVIKINSTVGHDLNLDVCGEC
yruck0001_860    --------------------MKKGIHPKYELVTASCSCGNVIKINSTVGHDLNLDVCGEC
yaldo0001_1140   --------------------MKQGIHPKYEQVTASCSCGNVIKINSTVGHDLNLDVCGEC
ymoll0001_33530  --------------------MKQGIHPKYDLVTASCSCGNVIKINSTVGHDLNLDVCGEC
yberc0001_950    -------------------------------------------------------VCGEC
yfred0001_980    --------------------MKQGIHPKYDQVTASCSCGNVIKINSTVGHDLNLDVCGEC
yinte0001_35800  --------------------MKQGIHPKYDLVTASCSCGNVIKINSTVGHDLNLDVCGEC
                                     ########################################


                         70        80        90
                 =========+=========+=========+==
ypseu0001X_2000  HPFYTGKQRDVASGGRVDRFNKRFSVPGAKK-
ypest0001X_3090  HPFYTGKQRDVASGGRVDRFNKRFSVPGAKK-
yente0001X_4209  HPFYTGKQRDVATGGRVDRFNKRFSVPGTTKA
ykris0001_930    HPFYTGKQRDVATGGRVDRFNKRFSVPGTTKA
yrohd0001_930    HPFYTGKQRDVATGGRVDRFNKRFSVPGAKK-
yruck0001_860    HPFYTGKQRDVATGGRVDRFNKRFSVPGAKK-
yaldo0001_1140   HPFYTGKQRDVATGGRVDRFNKRFSVPGATK-
ymoll0001_33530  HPFYTGKQRDVATGGRVDRFNKRFSVPGAAKK
yberc0001_950    HPFYTGKQRDVATGGRVDRFNKRFSVPGAAKK
yfred0001_980    HPFYTGKQRDVATGGRVDRFNKRFSVPGAKK-
yinte0001_35800  HPFYTGKQRDVATGGRVDRFNKRFSVPGAKK-
                 ###############################
```

```
Parameters used
Minimum Number Of Sequences For A Conserved Position: 6
Minimum Number Of Sequences For A Flanking Position: 9
Maximum Number Of Contiguous Nonconserved Positions: 8
Minimum Length Of A Block: 10
Allowed Gap Positions: With Half
Use Similarity Matrices: Yes
```

```
Flank positions of the 1 selected block(s)
Flanks: [21  91]  

New number of positions in PGL1_unique_yersinia-CLUSTERS.dir/PGL1_unique_yersinia-CL1000/PGL1_unique_yersinia-CL1000.muscle.fasta.gblo:  71  (77% of the original 92 positions)
```
