# Supplementary material for: Genomic characterization of the Yersinia genus
Source: Genome Biol. 2010 Jan 4;11(1):R1. doi: 10.1186/gb-2010-11-1-r1 (PMC2847712; doi:10.1186/gb-2010-11-1-r1)
Supplement: Additional file 15 — The top level directory consists of a directory called Additional_cluster_files and 5010 directories, one for each multi-protein cluster family. (This top level directory has been split into three data files for uploading purposes (Additional files 15, 16, 17).) Within the directory are the following files: PGL1_unique_Yersinia_unclustered.out - list of all protein singletons that MCL did not group into a cluster (see Materials and Methods); PGL1_Yersinia_unique_locus_tags.txt - names of the 11 locus tag prefixes used for each genome; PGL1_unique_Yersinia.gff - mapping each Yersinia protein to a cluster in tab delimited GFF; PGL1_unique_Yersinia.sigfile - list of the longest protein in each cluster; PGL1_unique_Yersinia.summary - summary table of features of each of the clusters; PGL1_unique_Yersinia.table - summary table of each protein in the clusters. Within each cluster directory are the following files, where 'x' is the cluster name: PGL1_unique_Yersinia-x.faa - multifasta file of the proteins in the cluster; PGL1_unique_Yersinia-x.summary - summary of the properties of the proteins; PGL1_unique_Yersinia-x.matches - blast matches between the proteins of the cluster; PGL1_unique_Yersinia-x.muscle.fasta - muscle alignment of the proteins; PGL1_unique_Yersinia-x.muscle.fasta.gblo - gblocks output of muscle alignment (that is, auto-trimmed alignment); PGL1_unique_Yersinia-x.muscle.fasta.gblo.htm - as above in html format; PGL1_unique_Yersinia-x.muscle.tree - treefile from muscle alignment; PGL1_unique_Yersinia-x.sif - matches between proteins in simple interaction format for display on graphing software. [file gb-2010-11-1-r1-S15.zip › clusters/PGL1_unique_yersinia-CL1003/PGL1_unique_yersinia-CL1003.muscle.fasta.gblo.htm]

PGL1\_unique\_yersinia-CL1003.muscle.fasta


## Gblocks 0.91b Results

Processed file: **PGL1\_unique\_yersinia-CL1003.muscle.fasta**  
Number of sequences: **11**  
Alignment assumed to be: **Protein**  
New number of positions: **79** (selected positions are underlined in blue)

```
                         10        20        30        40        50        60
                 =========+=========+=========+=========+=========+=========+
yruck0001_1400   MASLMQLRDAIALNGRMEASQLSQLLAAPLPLIEAMLERLIAMGKLKRIEQDNSGCLSGG
yrohd0001_1710   MASLLQLRDAIALNGSADAHQLSHQFAMPLPLVEAMLEKLTAMGKLERIEQDNRGCLTGS
ypseu0001X_4174  MASLLQLRDAIALNGSAEASQLSRQLAIPLPLVNAMLEKLTAMGKIERIELDHSGCLTGS
ypest0001X_3300  MASLLQLRDAIALNGSAEASQLSRQLAIPLPLVNAMLEKLTAMGKIERIELDHSGCLTGS
yinte0001_1460   MASLLQLRDAIALCGSTGANQLSQQLATPLPLVEAMLERLTAMGKIERIEQDNRGCLTGS
yaldo0001_1220   MASLLQLRDAIALSGSVGASQLSQQLATPLSLVEAMLEKLTVMGKIERIEQDSSGCLTGS
ykris0001_1330   MASLVQLRDAIALSGSTDAKQLSHQLALPLPLVEAMLEKLTAIGKIERIEQDNSGCMSGS
yente0001X_2260  VASLVQLRDAIALSGSADANQLSHQLAMPLPLVEAMLEKLTAMGKIERIEQDNSGCLTGS
yfred0001_1600   MASLLQLRDAIALSGNADANQLSHQLAMPLPLVEAMLERLMAMGKIERIEQDNNGCLTGS
yberc0001_1160   MASLLQLRDAIALSGSADAHQLSQQLAAPLPLVEAMLERLTAMGKIERIEQDNSGCLTGS
ymoll0001_700    MASLLQLRDAIALSGSIDANQLSQQLALPLPLVEAMLERLTAMGKIERIEQDNSGCLTGS
                 ############################################################


                         70        80
                 =========+=========+=====
yruck0001_1400   CKSCPEGP-KCNTVVYQLKTH----
yrohd0001_1710   CKQCPEGQAQCNTVIYHIAPNS---
ypseu0001X_4174  CKSCPEGHQHCNTVIYQLKEPHAHQ
ypest0001X_3300  CKSCPEGHQHCNTVIYQLKEPHAHQ
yinte0001_1460   CKRCPEGNNHCNIVIYQIKNHH---
yaldo0001_1220   CKHCPEGRNQCNTVIYQLKDHR---
ykris0001_1330   CKSCPEGKSQCNTVIYQLKKHH---
yente0001X_2260  CKSCPEGKNQCSTVIYQLKNHR---
yfred0001_1600   CKSCPEGQNHCRTVIYQLK------
yberc0001_1160   CKSCPEGQNQCHTVIYQLKDQH---
ymoll0001_700    CKSCPEGQNQCHPVSYQLKNHQ---
                 ###################
```

```
Parameters used
Minimum Number Of Sequences For A Conserved Position: 6
Minimum Number Of Sequences For A Flanking Position: 9
Maximum Number Of Contiguous Nonconserved Positions: 8
Minimum Length Of A Block: 10
Allowed Gap Positions: With Half
Use Similarity Matrices: Yes
```

```
Flank positions of the 1 selected block(s)
Flanks: [1  79]  

New number of positions in PGL1_unique_yersinia-CLUSTERS.dir/PGL1_unique_yersinia-CL1003/PGL1_unique_yersinia-CL1003.muscle.fasta.gblo:  79  (92% of the original 85 positions)
```
