# Supplementary material for: Genomic characterization of the Yersinia genus
Source: Genome Biol. 2010 Jan 4;11(1):R1. doi: 10.1186/gb-2010-11-1-r1 (PMC2847712; doi:10.1186/gb-2010-11-1-r1)
Supplement: Additional file 15 — The top level directory consists of a directory called Additional_cluster_files and 5010 directories, one for each multi-protein cluster family. (This top level directory has been split into three data files for uploading purposes (Additional files 15, 16, 17).) Within the directory are the following files: PGL1_unique_Yersinia_unclustered.out - list of all protein singletons that MCL did not group into a cluster (see Materials and Methods); PGL1_Yersinia_unique_locus_tags.txt - names of the 11 locus tag prefixes used for each genome; PGL1_unique_Yersinia.gff - mapping each Yersinia protein to a cluster in tab delimited GFF; PGL1_unique_Yersinia.sigfile - list of the longest protein in each cluster; PGL1_unique_Yersinia.summary - summary table of features of each of the clusters; PGL1_unique_Yersinia.table - summary table of each protein in the clusters. Within each cluster directory are the following files, where 'x' is the cluster name: PGL1_unique_Yersinia-x.faa - multifasta file of the proteins in the cluster; PGL1_unique_Yersinia-x.summary - summary of the properties of the proteins; PGL1_unique_Yersinia-x.matches - blast matches between the proteins of the cluster; PGL1_unique_Yersinia-x.muscle.fasta - muscle alignment of the proteins; PGL1_unique_Yersinia-x.muscle.fasta.gblo - gblocks output of muscle alignment (that is, auto-trimmed alignment); PGL1_unique_Yersinia-x.muscle.fasta.gblo.htm - as above in html format; PGL1_unique_Yersinia-x.muscle.tree - treefile from muscle alignment; PGL1_unique_Yersinia-x.sif - matches between proteins in simple interaction format for display on graphing software. [file gb-2010-11-1-r1-S15.zip › clusters/PGL1_unique_yersinia-CL1004/PGL1_unique_yersinia-CL1004.muscle.fasta.gblo.htm]

PGL1\_unique\_yersinia-CL1004.muscle.fasta


## Gblocks 0.91b Results

Processed file: **PGL1\_unique\_yersinia-CL1004.muscle.fasta**  
Number of sequences: **11**  
Alignment assumed to be: **Protein**  
New number of positions: **255** (selected positions are underlined in blue)

```
                         10        20        30        40        50        60
                 =========+=========+=========+=========+=========+=========+
yruck0001_26800  MSAQPLLVVEGLSMRFGGLLAVNNVGLTLNQGEIVSLIGPNGAGKTTIFNCLTGFYRPTG
ypseu0001X_3360  MNGQPLLTVEGLSMRFGGLLAVNNVGLTLNQGEIVSLIGPNGAGKTTIFNCLTGFYRPTG
ypest0001X_4110  MNGQPLLTVEGLSMRFGGLLAVNNVGLTLNQGEIVSLIGPNGAGKTTIFNCLTGFYRPTG
yfred0001_29830  MSTQPLLAVEGLSMRFGGLLAVNNVGLTLNQGEIVSLIGPNGAGKTTIFNCLTGFYRPTG
yrohd0001_29720  MSTQPLLAVEGLSMRFGGLLAVNNVGLTLNPGEIVSLIGPNGAGKTTIFNCLTGFYRPTG
ymoll0001_31960  MNTQPLLAVEGLSMRFGGLLAVNNVGLTLNQGEIVSLIGPNGAGKTTIFNCLTGFYRPTG
yberc0001_31570  -----LLAVEGLSMRFGGLLAVNNVGLTLNQGEIVSLIGPNGAGKTTIFNCLTGFYRPTG
yaldo0001_32640  MNTQPLLAVEGLSMRFGGLLAVNNVGLTLNQGEIVSLIGPNGAGKTTIFNCLTGFYRPTG
yinte0001_34500  MNTQPLLAVEGLSMRFGGLLAVNNVGLTLNQGEIVSLIGPNGAGKTTIFNCLTGFYRPTG
ykris0001_35530  MNTQPLLAVEGLSMRFGGLLAVNNVGLNLNQGEIVSLIGPNGAGKTTIFNCLTGFYRPTG
yente0001X_4086  MSTQPLLAVEGLSMRFGGLLAVNNVGLNLNQGEIVSLIGPNGAGKTTIFNCLTGFYRPTG
                 ############################################################


                         70        80        90       100       110       120
                 =========+=========+=========+=========+=========+=========+
yruck0001_26800  GTIKLRDRHLEGLPGQAIARMGVIRTFQHVRLFREMTVIENLLVAQHQHLKSGVFAGLLK
ypseu0001X_3360  GTIKLRERHLEGLPGQMIARMGVIRTFQHVRLFREMTVIENLLVAQHQHLKSGIFAGLLK
ypest0001X_4110  GTIKLRERHLEGLPGQMIARMGVIRTFQHVRLFREMTVIENLLVAQHQHLKSGIFAGLLK
yfred0001_29830  GTIKLRDRHLEGLPGQMIARMGVIRTFQHVRLFREMTVVENLLVAQHQHLKSGVFAGLLK
yrohd0001_29720  GTIKLRDRHLEGLPGQMIARMGVIRTFQHVRLFREMTVVENLLVAQHQHLKSGVFAGLLK
ymoll0001_31960  GTIKLRERHLEGLPGQMIARMGVIRTFQHVRLFREMTVVENLLVAQHQHLKSGIFAGLLK
yberc0001_31570  GTIKLRDRHLEGLPGQMIARMGVIRTFQHVRLFREMTVVENLLVAQHQHLKSGIFAGLLK
yaldo0001_32640  GTIKLRDRHLEGLPGQMIARMGLIRTFQHVRLFREMTVVENLLVAQHQHLKSGVFAGLLK
yinte0001_34500  GTIKLRDRHLEGLPGQMIARMGVIRTFQHVRLFREMTVVENLLVAQHQHLKSGVFAGLLK
ykris0001_35530  GTIKLRDRHIEGLPGQVIARMGVIRTFQHVRLFREMTVVENLLVAQHQHLKSGVFAGLLK
yente0001X_4086  GTIKLRDRHIEGLPGQVIARMGVIRTFQHVRLFREMTVVENLLVAQHQHLKSGVFAGLLK
                 ############################################################


                        130       140       150       160       170       180
                 =========+=========+=========+=========+=========+=========+
yruck0001_26800  TPAFRRAEADALDRAAVWLERVGLLDLANRQAGNLAYGQQRRLEIARCMVTRPALLMLDE
ypseu0001X_3360  TPGFRRAEADALARAATWLERVGLLALANRQAGNLAYGQQRRLEIARCMVTRPELLMLDE
ypest0001X_4110  TPGFRRAEADALARAATWLERVGLLALANRQAGNLAYGQQRRLEIARCMVTRPELLMLDE
yfred0001_29830  TPGFRRAEADALERAATWLDRVGLLDLANRQAGNLAYGQQRRLEIARCMVTRPELLMLDE
yrohd0001_29720  TPAFRRAEADALERAATWLERIGLLDLANRQAGNLAYGQQRRLEIARCMVTRPELLMLDE
ymoll0001_31960  TPGFRRAEADALERAATWLERVGLLELANRQAGNLAYGQQRRLEIARCMVTRPELLMLDE
yberc0001_31570  TPGFRRAEADALERAATWLERVGLLELANRQAGNLAYGQQRRLEIARCMVTRPELLMLDE
yaldo0001_32640  TPGFRRAEADALERAATWLERVGLLELANRQAGNLAYGQQRRLEIARCMVTRPELLMLDE
yinte0001_34500  TPGFRRAEADALERAATWLERVGLLELANRQAGNLAYGQQRRLEIARCMVTRPELLMLDE
ykris0001_35530  TPGFRRAEADALERAATWLERVGLLELANRQAGNLAYGQQRRLEIARCMVTRPELLMLDE
yente0001X_4086  TPGFRRAEADALERAATWLERVGLLELANRQAGNLAYGQQRRLEIARCMVTRPELLMLDE
                 ############################################################


                        190       200       210       220       230       240
                 =========+=========+=========+=========+=========+=========+
yruck0001_26800  PAAGLNPKETDELNQLIMELRNQHQVSVLLIEHDMKLVMGISDRIYVVNQGTPLAQGIPA
ypseu0001X_3360  PAAGLNPKETDELNQLIMELRDQHQVSVLLIEHDMKLVMGISDRIYVVNQGTPLAQGIPA
ypest0001X_4110  PAAGLNPKETDELNQLIMELRDQHQVSVLLIEHDMKLVMGISDRIYVVNQGTPLAQGLPA
yfred0001_29830  PAAGLNPKETDELNQLIMELRDQHQVSVLLIEHDMKLVMGISDRIYVVNQGTPLAHGTPA
yrohd0001_29720  PAAGLNPKETDELNQLIMELRDQHQVSVLLIEHDMKLVMGISDRIYVVNQGTPLAHGTPA
ymoll0001_31960  PAAGLNPKETDELNQLIMELRGQHQVSVLLIEHDMKLVMGISDRIYVVNQGTPLAQGTPA
yberc0001_31570  PAAGLNPKETDELNQLIMELRSQHQVSVLLIEHDMKLVMGISDRIYVVNQGTPLAQGTPA
yaldo0001_32640  PAAGLNPKETEELNQLIMELRDQHQVSVLLIEHDMKLVMGISDRIYVVNQGTPLAQGTPA
yinte0001_34500  PAAGLNPKETDELNQLIMELRDQHQVSVLLIEHDMKLVMGISDRIYVVNQGTPLAQGTPG
ykris0001_35530  PAAGLNPKETDELNQLIMELRDQHQVSVLLIEHDMKLVMGISDRIYVVNQGTPLAQGLPA
yente0001X_4086  PAAGLNPKETDELNQLIMELRDQHQVSVLLIEHDMKLVMGISDRIYVVNQGTPLAQGSPI
                 ############################################################


                        250
                 =========+=====
yruck0001_26800  EIRNNPDVIRAYLGE
ypseu0001X_3360  EIRNNPDVIRAYLGE
ypest0001X_4110  EIRNNPDVIRAYLGE
yfred0001_29830  EIRNNPDVIRAYLGE
yrohd0001_29720  EIRNNPDVIRAYLGE
ymoll0001_31960  EIRDNPDVIRAYLGE
yberc0001_31570  EIRDNPDVIRAYLGE
yaldo0001_32640  EIRNNPDVIRAYLGE
yinte0001_34500  --RNS----------
ykris0001_35530  EIRNNPDVIRAYLGE
yente0001X_4086  EIRNNPDVIRAYLGE
                 ###############
```

```
Parameters used
Minimum Number Of Sequences For A Conserved Position: 6
Minimum Number Of Sequences For A Flanking Position: 9
Maximum Number Of Contiguous Nonconserved Positions: 8
Minimum Length Of A Block: 10
Allowed Gap Positions: With Half
Use Similarity Matrices: Yes
```

```
Flank positions of the 1 selected block(s)
Flanks: [1  255]  

New number of positions in PGL1_unique_yersinia-CLUSTERS.dir/PGL1_unique_yersinia-CL1004/PGL1_unique_yersinia-CL1004.muscle.fasta.gblo:  255  (100% of the original 255 positions)
```
