# Supplementary material for: Genomic characterization of the Yersinia genus
Source: Genome Biol. 2010 Jan 4;11(1):R1. doi: 10.1186/gb-2010-11-1-r1 (PMC2847712; doi:10.1186/gb-2010-11-1-r1)
Supplement: Additional file 15 — The top level directory consists of a directory called Additional_cluster_files and 5010 directories, one for each multi-protein cluster family. (This top level directory has been split into three data files for uploading purposes (Additional files 15, 16, 17).) Within the directory are the following files: PGL1_unique_Yersinia_unclustered.out - list of all protein singletons that MCL did not group into a cluster (see Materials and Methods); PGL1_Yersinia_unique_locus_tags.txt - names of the 11 locus tag prefixes used for each genome; PGL1_unique_Yersinia.gff - mapping each Yersinia protein to a cluster in tab delimited GFF; PGL1_unique_Yersinia.sigfile - list of the longest protein in each cluster; PGL1_unique_Yersinia.summary - summary table of features of each of the clusters; PGL1_unique_Yersinia.table - summary table of each protein in the clusters. Within each cluster directory are the following files, where 'x' is the cluster name: PGL1_unique_Yersinia-x.faa - multifasta file of the proteins in the cluster; PGL1_unique_Yersinia-x.summary - summary of the properties of the proteins; PGL1_unique_Yersinia-x.matches - blast matches between the proteins of the cluster; PGL1_unique_Yersinia-x.muscle.fasta - muscle alignment of the proteins; PGL1_unique_Yersinia-x.muscle.fasta.gblo - gblocks output of muscle alignment (that is, auto-trimmed alignment); PGL1_unique_Yersinia-x.muscle.fasta.gblo.htm - as above in html format; PGL1_unique_Yersinia-x.muscle.tree - treefile from muscle alignment; PGL1_unique_Yersinia-x.sif - matches between proteins in simple interaction format for display on graphing software. [file gb-2010-11-1-r1-S15.zip › clusters/PGL1_unique_yersinia-CL1007/PGL1_unique_yersinia-CL1007.muscle.fasta.gblo.htm]

PGL1\_unique\_yersinia-CL1007.muscle.fasta


## Gblocks 0.91b Results

Processed file: **PGL1\_unique\_yersinia-CL1007.muscle.fasta**  
Number of sequences: **11**  
Alignment assumed to be: **Protein**  
New number of positions: **341** (selected positions are underlined in blue)

```
                         10        20        30        40        50        60
                 =========+=========+=========+=========+=========+=========+
yruck0001_31230  -MSSLSDINRARWARFKHNRRGYWSLWLFMTLFIISLFAEFIANDRPLLVSYQGKLYLPF
yaldo0001_10710  -MIKLSAINQARWARFRHNKRGYWSLWIFLALFVISLFAEFIANDKPLLVSYQGKIYMPF
yrohd0001_9360   -MIRLSAINQIRWNQFRRNRRGYWSLWIFLTLFIISLFAEFIANDKPLLVSYQGKIYMPF
ypseu0001X_1493  MMMRLSAINQARWARFRQNRRGYWSLWIFLTLFVISLFAELIANDKPLLVSYQGKFYMPF
ypest0001X_1490  MMMRLSAINQARWARFRQNRRGYWSLWIFLTLFVISLFAELIANDKPLLVSYQGKFYMPF
yente0001X_2883  MIKRLSAINQARWARFRQNRRGYWSLWIFLTLFIISLFAELIANDKPLLVSYQGKFYMPF
ykris0001_10650  MMKRLNAINQARWARFRQNRRGYWSLWIFLTLFIISLFAELIANDKPLLVSYQGKFYMPF
yberc0001_10260  -MIKLSAINQARWARFRQNRRGYWSLWIFLTLFIISLFAELIANDKPLLVNYQGKIYLPF
ymoll0001_9890   -MIRLSAINQARWARFRQNRRGYWSLWIFLTLFIISLFAELLANDKPLLVNYQGKIYMPF
yinte0001_10360  -MIRLSAINQARWARFRNNRRGYWSLWIFLTLFIISLFAELIANDKPLLVNYQGRIYMPF
yfred0001_9640   -MIRLSAINQARWGRFRQNRRGYWSLWIFLTLFIISLFAELIANDKPLLVSYQGRIYMPF
                  ###########################################################


                         70        80        90       100       110       120
                 =========+=========+=========+=========+=========+=========+
yruck0001_31230  MVNYNETTFGGVLTTAADYQDPFVIRQIENHGWAIWAPIRYANNTINFNTQIPFPSPPSR
yaldo0001_10710  MSNYSESTFGGILTTAADYQDPYVIGRINDNGWAIWAPIRFSNNTINFATDVPFPSPPNR
yrohd0001_9360   MTNYSESTFGGILTTAADYQDPYVINRIKEHGWAIWAPIRFSNNTINFATDVPFPSPPSR
ypseu0001X_1493  MVNYSESTFGGVLTTAADYQDPYVIGRINDSGWAIWAPIRFSNNTINFATDVPFPSPPSR
ypest0001X_1490  MVNYSESTFGGVLTTAADYQDPYVIGRINDSGWAIWAPIRFSNNTINFATDVPFPSPPSR
yente0001X_2883  MVNYTESTFGGILTTAADYQDPYVIGRIKDNGWAIWAPIRFSNNTINFATDVPFPSPPSH
ykris0001_10650  MVNYTESTFGGILTTAADYQDPYVIGRIKDNGWAIWAPIRFSNNTINFATNVPFPSPPSR
yberc0001_10260  MVNYSESTFGGILTTAADYQDPYVLDRIKNNGWAIWAPIRFSNNTINFATEVPFPSPPSR
ymoll0001_9890   MVNYSESTFGGILTTAADYQDPYVLDRIKNNGWAIWAPIRFSNNTINFATDVPFPSPPSR
yinte0001_10360  MTNYSESTFGGILTTAADYQDPYVIGRINHNGWAIWAPIRFSNNTINFATDVPFPSPPSR
yfred0001_9640   MTNYSESTFGGILTTAADYQDPYVIDRIKDNGWAIWAPIRFSNNTINFATDVPFPSPPSR
                 ############################################################


                        130       140       150       160       170       180
                 =========+=========+=========+=========+=========+=========+
yruck0001_31230  TNWLGTDSNGNDVLAQVIYGFRISMLFGLTLTLFSSLIGIAVGAIQGYYGGKIDLWGQRF
yaldo0001_10710  TNLLGTDSTGGDVLAKVIYGFRISLLFGLTLTLFSSVIGICAGAIQGYYGGRIDLWGQRF
yrohd0001_9360   TNWLGTDSTGGDVLAKVIYGFRISLLFGLTLTLFSSVIGICAGAIQGYYGGRVDLLGQRF
ypseu0001X_1493  TNLLGTDSTGGDVLAKVIYGFRISLLFGLTLTLFSSVIGICAGAVQGYYGGRVDLWGQRF
ypest0001X_1490  TNLLGTDSTGGDVLAKVIYGFRISLLFGLTLTLFSSVIGICAGAVQGYYGGRVDLWGQRF
yente0001X_2883  TNWLGTDSTGGDVLAKIIYGFRISLLFGLTLTLLSSVIGICAGAVQGYYGGKIDLLGQRF
ykris0001_10650  TNLLGTDSTGGDVLAKVIYGFRISLLFGLTLTLFSSVIGICAGAIQGYYGGRVDLWGQRF
yberc0001_10260  TNLLGTDSTGGDVLTKVIYGFRISLLFGLTLTLFSSVIGICAGAIQGYYGGRVDLWGQRF
ymoll0001_9890   TNLLGTDSTGGDVLTKVIYGFRISLLFGLTLTLFSSVIGICAGAIQGYYGGKIDLLGQRF
yinte0001_10360  TNLLGTDSTGGDVLAKVIYGFRISLLFGLTLTLFSSVIGICAGAVQGYYGGKIDLWGQRF
yfred0001_9640   TNLLGTDSTGGDVLAKIIYGFRISLLFGLTLTLFSSVIGICAGAVQGYYGGRVDLWGQRF
                 ############################################################


                        190       200       210       220       230       240
                 =========+=========+=========+=========+=========+=========+
yruck0001_31230  IEVWSGMPTLFLVILLSSIIQPNFWWLLVITVLFGWMALVGVVRAEFLRTRNYDYIRAAR
yaldo0001_10710  IEVWSGMPTLFLVILLSSIVQPNFWWLLAITVLFGWMGLVGVVRAEFLRTRNYDYIRAAR
yrohd0001_9360   IEVWSGMPTLFLVILLSSIVQPNFWWLLAITVIFGWMGLVGVVRAEFLRTRNYDYIRAAR
ypseu0001X_1493  IEVWSGMPTLFLVILLSSIVQPNFWWLLAITVIFGWMGLVGVVRAEFLRTRNYDYIRAAR
ypest0001X_1490  IEVWSGMPTLFLVILLSSIVQPNFWWLLAITVIFGWMGLVGVVRAEFLRTRNYDYIRAAR
yente0001X_2883  IEVWSGMPTLFLIILLSSIVQPNFWWLLAITVIFGWMGLVGVVRAEFLRTRNYDYIRAAR
ykris0001_10650  IEVWSGMPTLFLVILLSSIVQPNFWWLLAITVIFGWMGLVGVVRAEFLRTRNYDYIRAAK
yberc0001_10260  IEVWSGMPTLFLVILLSSIVQPNFWWLLAITVLFGWMGLVGVVRAEFLRTRNYDYIRAAR
ymoll0001_9890   IEVWSGMPTLFLVILLSSIVQPNFWWLLAITVLFGWMGLVGVVRAEFLRTRNYDYIRAAR
yinte0001_10360  IEVWSGMPTLFLVILLSSIVQPNFWWLLAITVLFGWMGLVGVVRAEFLRTRNYDYIRAAR
yfred0001_9640   IEVWSGMPTLFLVILLSSIVQPNFWWLLGITVIFGWMGLVGVVRAEFLRTRNYDYIRAAR
                 ############################################################


                        250       260       270       280       290       300
                 =========+=========+=========+=========+=========+=========+
yruck0001_31230  AMGVQDRVIMSRHILPNAMVATLTFMPFILCGSITTLTSLDFLGFGLPIGSPSLGGLLLE
yaldo0001_10710  AMGVGDRVIMTRHMLPNAMVATLTFLPFILCGSITTLTSLDFLGFGLPMGSPSLGGLLLE
yrohd0001_9360   AMGVRDRVIMSRHMLPNAMVATLTFLPFILCGSITTLTSLDFLGFGLPMGSPSLGGLLLE
ypseu0001X_1493  AMGVRDRTIMTRHMLPNAMVATLTFLPFILCGSITTLTSLDFLGFGLPIGSPSLGGLLLE
ypest0001X_1490  AMGVRDRTIMTRHMLPNAMVATLTFLPFILCGSITTLTSLDFLGFGLPIGSPSLGGLLLE
yente0001X_2883  AMGVRDRVIMSRHMLPNAMVATLTFLPFILCGSITTLTSLDFLGFGLPMGSPSLGGLLLE
ykris0001_10650  AMGVRDRVIMSRHMLPNAMVATLTFLPFILCGSITTLTSLDFLGFGLPMGSPSLGGLLLE
yberc0001_10260  AMGVRDRTIMSRHMLPNAMVATLTFLPFILCGSITTLTSLDFLGFGLPMGSPSLGGLLLE
ymoll0001_9890   AMGVRDRTIMSRHMLPNAMVATLTFLPFILCGSITTLTSLDFLGFGLPMGSPSLGGLLLE
yinte0001_10360  AMGVGDRVIMSRHMLPNAMVATLTFLPFILCGSITTLTSLDFLGFGLPMGSPSLGGLLLE
yfred0001_9640   AMGVRDRVIMSRHMLPNAMVATLTFLPFILCGSITTLTSLDFLGFGLPMGSPSLGGLLLE
                 ############################################################


                        310       320       330       340
                 =========+=========+=========+=========+==
yruck0001_31230  GKNNLQAPWLGLTAFITLAMLLSLLIFIGEAVRDAFDPSKAY
yaldo0001_10710  GKNNLQAPWLGITAFLVLAMLLSLLIFIGEAVRDAFDPSKAY
yrohd0001_9360   GKNNLQAPWLGITAFLVLAVLLSLLIFIGEAVRDAFDPNKVY
ypseu0001X_1493  GKNNLQAPWLGITAFLVLAVLLSLLIFIGEAVRDAFDPSKVY
ypest0001X_1490  GKNNLQAPWLGITAFLVLAVLLSLLIFIGEAVRDAFDPSKVY
yente0001X_2883  GKNNLQAPWLGITAFLVLAVLLSLLIFIGEAVRDAFDPSKVY
ykris0001_10650  GKNNLQAPWLGITAFLVLAVLLSLLIFIGEAVRDAFDPSKVY
yberc0001_10260  GKNNLQAPWLGITAFLVLAVLLSLLIFIGEAVRDAFDPSKVY
ymoll0001_9890   GKNNLQAPWLGITAFLVLAVLLSLLIFIGEAVRDAFDPSKVY
yinte0001_10360  GKNNLQAPWLGITAFLVLAVLLSLLIFIGEAVRDAFDPSKVY
yfred0001_9640   GKNNLQAPWLGITAFLVLAVLLSLLIFIGEAVRDAFDPSKVY
                 ##########################################
```

```
Parameters used
Minimum Number Of Sequences For A Conserved Position: 6
Minimum Number Of Sequences For A Flanking Position: 9
Maximum Number Of Contiguous Nonconserved Positions: 8
Minimum Length Of A Block: 10
Allowed Gap Positions: With Half
Use Similarity Matrices: Yes
```

```
Flank positions of the 1 selected block(s)
Flanks: [2  342]  

New number of positions in PGL1_unique_yersinia-CLUSTERS.dir/PGL1_unique_yersinia-CL1007/PGL1_unique_yersinia-CL1007.muscle.fasta.gblo:  341  (99% of the original 342 positions)
```
