# Supplementary material for: Genomic characterization of the Yersinia genus
Source: Genome Biol. 2010 Jan 4;11(1):R1. doi: 10.1186/gb-2010-11-1-r1 (PMC2847712; doi:10.1186/gb-2010-11-1-r1)
Supplement: Additional file 15 — The top level directory consists of a directory called Additional_cluster_files and 5010 directories, one for each multi-protein cluster family. (This top level directory has been split into three data files for uploading purposes (Additional files 15, 16, 17).) Within the directory are the following files: PGL1_unique_Yersinia_unclustered.out - list of all protein singletons that MCL did not group into a cluster (see Materials and Methods); PGL1_Yersinia_unique_locus_tags.txt - names of the 11 locus tag prefixes used for each genome; PGL1_unique_Yersinia.gff - mapping each Yersinia protein to a cluster in tab delimited GFF; PGL1_unique_Yersinia.sigfile - list of the longest protein in each cluster; PGL1_unique_Yersinia.summary - summary table of features of each of the clusters; PGL1_unique_Yersinia.table - summary table of each protein in the clusters. Within each cluster directory are the following files, where 'x' is the cluster name: PGL1_unique_Yersinia-x.faa - multifasta file of the proteins in the cluster; PGL1_unique_Yersinia-x.summary - summary of the properties of the proteins; PGL1_unique_Yersinia-x.matches - blast matches between the proteins of the cluster; PGL1_unique_Yersinia-x.muscle.fasta - muscle alignment of the proteins; PGL1_unique_Yersinia-x.muscle.fasta.gblo - gblocks output of muscle alignment (that is, auto-trimmed alignment); PGL1_unique_Yersinia-x.muscle.fasta.gblo.htm - as above in html format; PGL1_unique_Yersinia-x.muscle.tree - treefile from muscle alignment; PGL1_unique_Yersinia-x.sif - matches between proteins in simple interaction format for display on graphing software. [file gb-2010-11-1-r1-S15.zip › clusters/PGL1_unique_yersinia-CL1008/PGL1_unique_yersinia-CL1008.muscle.fasta.gblo.htm]

PGL1\_unique\_yersinia-CL1008.muscle.fasta


## Gblocks 0.91b Results

Processed file: **PGL1\_unique\_yersinia-CL1008.muscle.fasta**  
Number of sequences: **11**  
Alignment assumed to be: **Protein**  
New number of positions: **424** (selected positions are underlined in blue)

```
                         10        20        30        40        50        60
                 =========+=========+=========+=========+=========+=========+
yruck0001_1500   -------------------VTLLFVSLVTTYLVVLNFAILPSLQQFNKVLAYEVRMLMTD
ypseu0001X_4167  MKWWRFSPRSSFARTLLLIVTLLFVSLVTTYLVVLNFAILPSLQQFNKVLAYEVRMLMTD
ypest0001X_3360  MKWWRFSPRSSFARTLLLIVTLLFVSLVTTYLVVLNFAILPSLQQFNKVLAYEVRMLMTD
yrohd0001_1770   MRRWRFSPRSSFARTLLLIVTLLFVSLVTTYLVVLNFAILPSLQQFNKVLAYEVRMLMTD
ykris0001_1390   MRRWRFSPRSSFARTLLLIVTLLFVSLVTTYLVVLNFAILPSLQQFNKVLAYEVRMLMTD
yente0001X_2170  MRRWRFSPRSSFARTLLLIVTLLFVSLVTTYLVVLNFAILPSLQQFNKVLAYEVRMLMTD
yfred0001_1660   -------------------VTLLFVSLVTTYLVVLNFAILPSLQQFNKVLAYEVRMLMTD
yaldo0001_1350   MKRWRFSPRSSFARTLLLIVTLLFVSLVTTYLVVLNFAILPSLQQFNKVLAYEVRMLMTD
yinte0001_1520   -------------------VTLLFVSLVTTYLVVLNFAILPSLQQFNKVLAYEVRMLMTD
ymoll0001_760    MRWWRFSPRSSFARTLLLIVTLLFVSLVTTYLVVLNFAILPSLQQFNKVLAYEVRMLMTD
yberc0001_1230   MRRWRFSPRSSFARTLLLIVTLLFVSLVTTYLVVLNFAILPSLQQFNKVLAYEVRMLMTD
                                    #########################################


                         70        80        90       100       110       120
                 =========+=========+=========+=========+=========+=========+
yruck0001_1500   RLQLEDGTLLEVPPAFRREIYRELGISLYTNAAAEESGLRWAQHYKFLSDQMAQQLGGPT
ypseu0001X_4167  RLQLEDGTLLEVPPAFRREIYRELGISLYTNAAAEESGLRWAQHYKFLSDQMAQQLGGPT
ypest0001X_3360  RLQLEDGTLLEVPPAFRREIYRELGISLYTNAAAEESGLRWAQHYKFLSDQMAQQLGGPT
yrohd0001_1770   RLQLEDGTLLEVPPAFRREIYRELGISLYTNAAAEESGLRWAQHYKFLSDQMAQQLGGPT
ykris0001_1390   RLQLEDGTLLEVPPAFRREIYRELGISLYTNAAAEESGLRWAQHYKFLSDQMAQQLGGPT
yente0001X_2170  RLQLEDGTLLEVPPAFRREIYRELGISLYTNAAAEESGLRWAQHYKFLSDQMAQQLGGPT
yfred0001_1660   RLQLEDGTLLEVPPAFRREIYRELGISLYTNAAAEESGLRWAQHYKFLSDQMAQQLGGPT
yaldo0001_1350   RLQLEDGTLLEVPPAFRREIYRELGISLYTNAAAEESGLRWAQHYKFLSDQMAQQLGGPT
yinte0001_1520   RLQLEDGTLLEVPPAFRREIYRELGISLYTNAAAEESGLRWAQHYKFLSDQMAQQLGGPT
ymoll0001_760    RLQLEDGTLLEVPPAFRREIYRELGISLYTNAAAEESGLRWAQHYKFLSDQMAQQLGGPT
yberc0001_1230   RLQLEDGTLLEVPPAFRREIYRELGISLYTNAAAEESGLRWAQHYKFLSDQMAQQLGGPT
                 ############################################################


                        130       140       150       160       170       180
                 =========+=========+=========+=========+=========+=========+
yruck0001_1500   DVRVEVSKNSPVVWLKTWLSPDIWVRVPLTEIHQGDFSPLFRYTLAIMLLAVGGAWLFIR
ypseu0001X_4167  DVRVEVNKNSPVVWLKTWLSPDIWVRVPLTEIHQGDFSPLFRYTLAIMLLAVGGAWLFIR
ypest0001X_3360  DVRVEVNKNSPVVWLKTWLSPDIWVRVPLTEIHQGDFSPLFRYTLAIMLLAVGGAWLFIR
yrohd0001_1770   DVRVEVNKNSPVVWLKTWLSPDIWVRVPLTEIHQGDFSPLFRYTLAIMLLAVGGAWLFIR
ykris0001_1390   DVRVEVNKNSPVVWLKTWLSPDIWVRVPLTEIHQGDFSPLFRYTLAIMLLAVGGAWLFIR
yente0001X_2170  DVRVEVNKNSPVVWLKTWLSPDIWVRVPLTEIHQGDFSPLFRYTLAIMLLAVGGAWLFIR
yfred0001_1660   DVRVEVNKNSPVVWLKTWLSPDIWVRVPLTEIHQGDFSPLFRYTLAIMLLAVGGAWLFIR
yaldo0001_1350   DVRVEVNKNSPVVWLKTWLSPDIWVRVPLTEIHQGDFSPLFRYTLAIMLLAVGGAWLFIR
yinte0001_1520   DVRVEVNKNSPVVWLKTWLSPDIWVRVPLTEIHQGDFSPLFRYTLAIMLLAVGGAWLFIR
ymoll0001_760    DVRVEVNKNSPVVWLKTWLSPDIWVRVPLTEIHQGDFSPLFRYTLAIMLLAVGGAWLFIR
yberc0001_1230   DVRVEVNKNSPVVWLKTWLSPDIWVRVPLTEIHQGDFSPLFRYTLAIMLLAVGGAWLFIR
                 ############################################################


                        190       200       210       220       230       240
                 =========+=========+=========+=========+=========+=========+
yruck0001_1500   IQNRPLVELEHAALQVGKGNIPPPLREYGASEVRSVTRAFNQMASGVKLLADDRTLLMAG
ypseu0001X_4167  IQNRPLVELEHAALQVGKGNIPPPLREYGASEVRSVTRAFNQMAAGVKLLSDDRTLLMAG
ypest0001X_3360  IQNRPLVELEHAALQVGKGNIPPPLREYGASEVRSVTRAFNQMAAGVKLLSDDRTLLMAG
yrohd0001_1770   IQNRPLVELEHAAVQVGKGIIPPPLREYGASEVRSVTRAFNQMAAGVKLLADDRTLLMAG
ykris0001_1390   IQNRPLVELEHAALQVGKGIIPPPLREYGASEVRSVTRAFNQMAAGVKLLADDRTLLMAG
yente0001X_2170  IQNRPLVELEHAALQVGKGIIPPPLREYGASEVRSVTRAFNQMAAGVKLLADDRTLLMAG
yfred0001_1660   IQNRPLVELEHAALQVGKGIIPPPLREYGASEVRSVTRAFNQMAAGVKLLADDRTLLMAG
yaldo0001_1350   IQNRPLVELEHAAMQVGKGIIPPPLREYGASEVRSVTRAFNQMAAGVKLLADDRTLLMAG
yinte0001_1520   IQNRPLVELEHAALQVGKGIIPPPLREYGASEVRSVTRAFNQMAAGVKLLADDRTLLMAG
ymoll0001_760    IQNRPLVELEHAALQVGKGIIPPPLREYGASEVRSVTRAFNQMAAGVKLLADDRTLLMAG
yberc0001_1230   IQNRPLVELEHAALQVGKGIIPPPLREYGASEVRSVTRAFNQMAAGVKLLADDRTLLMAG
                 ############################################################


                        250       260       270       280       290       300
                 =========+=========+=========+=========+=========+=========+
yruck0001_1500   VSHDLRTPLTRIRLATEMMAEEDGYLSESINKDIEECNAIIEQFIDYLRTGQEMPTEMSE
ypseu0001X_4167  VSHDLRTPLTRIRLATEMMSEDDAYLSESINKDIEECNAIIEQFIDYLRTGQEMPTEPSD
ypest0001X_3360  VSHDLRTPLTRIRLATEMMSEDDAYLSESINKDIEECNAIIEQFIDYLRTGQEMPTEPSD
yrohd0001_1770   VSHDLRTPLTRIRLATEMMSEADGYLSESINKDIEECNAIIEQFIDYLRTGQEMPTESSD
ykris0001_1390   VSHDLRTPLTRIRLATEMMSEADGYLSESINKDIEECNAIIEQFIDYLRTGQEMPTEPSD
yente0001X_2170  VSHDLRTPLTRIRLATEMMSEADGYLSESINKDIEECNAIIEQFIDYLRTGQEMPTEPSD
yfred0001_1660   VSHDLRTPLTRIRLATEMMSEADGYLSESINKDIEECNAIIEQFIDYLRTGQEMPTEPSD
yaldo0001_1350   VSHDLRTPLTRIRLATEMMSEADGYLSESINKDIEECNAIIEQFIDYLRTGQEMPTESSD
yinte0001_1520   VSHDLRTPLTRIRLATEMMSEADGYLSESINKDIEECNAIIEQFIDYLRTGQEMPTESSD
ymoll0001_760    VSHDLRTPLTRIRLATEMMSEADGYLSESINKDIEECNAIIEQFIDYLRTGQEMPTEPSE
yberc0001_1230   VSHDLRTPLTRIRLATEMMSEADGYLSESINKDIEECNAIIEQFIDYLRTGQEMPTEPSD
                 ############################################################


                        310       320       330       340       350       360
                 =========+=========+=========+=========+=========+=========+
yruck0001_1500   LNAILGEVIAAESGYEREIETDLCDEEVLVEVHPLSIKRALANMVVNAARYGNGWIKVSS
ypseu0001X_4167  LNSVLGEVIAAESGYERVIETDLAEGEVLVDIHPLSIKRALTNMVVNAARYGNGWIKVSS
ypest0001X_3360  LNSVLGEVIAAESGYERVIETDLAEGEVLVDIHPLSIKRALTNMVVNAARYGNGWIKVSS
yrohd0001_1770   LNAVLGEVIAAESGYERVIETDLCDGEVMVDIHPLSIKRALANMVVNAARYGNGWIKVSS
ykris0001_1390   LNAVLGEVIAAESGYERVIETDLCEGEVIVDIHPLSIKRALANMVVNAARYGNGWIKVSS
yente0001X_2170  LNAVLGEVIAAESGYERVIETDLCEGEVMVDIHPLSIKRALANMVVNAARYGNGWIKVSS
yfred0001_1660   LNAVLGEVIAAESGYERVIETDLCEGEVMVDIHPLSIKRALANMVVNAARYGNGWIKVSS
yaldo0001_1350   LNAVLGEVIAAESGYERVIETDLCAGEVLVDIHPLSIKRALANMVVNAARYGNGWIKVSS
yinte0001_1520   LNAVLGEVIAAESGYERIIETDLCAGEVLVDIHPLSIKRALANMVVNAARYGNGWIKVSS
ymoll0001_760    LNSVLGEVIAAESGYERVIETDLCEGEVLVDIHPLSIKRALANMVVNAARYGNGWIKVSS
yberc0001_1230   LNSVLGEVIAAESGYERVIETDLCAGEVLVDIHPLSIKRALANMVVNAARYGNGWIKVSS
                 ############################################################


                        370       380       390       400       410       420
                 =========+=========+=========+=========+=========+=========+
yruck0001_1500   GTELQRAWFQVEDDGPGIKPEELKHLFQPFVRGDSARSTSGTGLGLAIVQRIIDAHSGML
ypseu0001X_4167  GKELQRAWFQVEDDGPGIKPEDLKHLLQPFVRGDSARSTSGTGLGLAIVQRIIDAHAGSL
ypest0001X_3360  GKELQRAWFQVEDDGPGIKPEDLKHLLQPFVRGDSARSTSGTGLGLAIVQRIIDAHAGSL
yrohd0001_1770   GTELQRAWFQVEDDGPGIKPEDLKHLFQPFVRGDSARSTSGTGLGLAIVQRIIDAHAGSL
ykris0001_1390   GTELQRAWFQVEDDGPGIKPEDLKHLFQPFVRGDSARSTSGTGLGLAIVQRIIDAHAGSL
yente0001X_2170  GTELQRAWFQVEDDGPGIKPEDLKHLFQPFVRGDSARSTSGTGLGLAIVQRIIDAHAGSL
yfred0001_1660   GTELQRAWFQVEDDGPGIKPEDLKHLFQPFVRGDSARSTSGTGLGLAIVQRIIDAHAGSL
yaldo0001_1350   GTELQRAWFQVEDDGPGIKPEDLKHLFQPFVRGDSARSTSGTGLGLAIVQRIIDAHAGSL
yinte0001_1520   GTELQRAWFQVEDDGPGIKPEDLKHLFQPFVRGDSARSTSGTGLGLAIVQRIIDAHAGSL
ymoll0001_760    GTELQRAWFQVEDDGPGIKPEELKHLFQPFVRGDSARSTSGTGLGLAIVQRIIDAHAGSL
yberc0001_1230   GTELQRAWFQVEDDGPGIKPEELKHLFQPFVRGDSARSTSGTGLGLAIVQRIIDAHAGSL
                 ############################################################


                        430       440       450
                 =========+=========+=========+===
yruck0001_1500   DIGTSDKGGLRIRAYLPLSLDVKSSTLGKHS--
ypseu0001X_4167  EIGTSKRGGLRIRAYIPLPLDVKPKPPAVA---
ypest0001X_3360  EIGTSKRGGLRIRAYIPLPLDVKPKPPAVA---
yrohd0001_1770   EIGASERGGLRIRAYIPLPLDVKPKLPVVVGNG
ykris0001_1390   DIGTSERGGLRIRAYIPLPLDVKPKPPAVV---
yente0001X_2170  DIGTSERGGLRIRAYIPLPLDVKPKPPAVA---
yfred0001_1660   DIGTSERGGLRIRAYIPLPLDVKPKLPVVVGNG
yaldo0001_1350   DIGTSERGGLRIRAYIPLPLDVKPKPPAVV---
yinte0001_1520   DIGTSERGGLRIRAYIPLPLDMKAKPPAVV---
ymoll0001_760    DIGTSERGGLRIRAYIPQLNNITSTHKS-----
yberc0001_1230   DIGTSERGGLRIRAYIPQLNYLTLSNKS-----
                 #######################
```

```
Parameters used
Minimum Number Of Sequences For A Conserved Position: 6
Minimum Number Of Sequences For A Flanking Position: 9
Maximum Number Of Contiguous Nonconserved Positions: 8
Minimum Length Of A Block: 10
Allowed Gap Positions: With Half
Use Similarity Matrices: Yes
```

```
Flank positions of the 1 selected block(s)
Flanks: [20  443]  

New number of positions in PGL1_unique_yersinia-CLUSTERS.dir/PGL1_unique_yersinia-CL1008/PGL1_unique_yersinia-CL1008.muscle.fasta.gblo:  424  (93% of the original 453 positions)
```
