# Supplementary material for: Genomic characterization of the Yersinia genus
Source: Genome Biol. 2010 Jan 4;11(1):R1. doi: 10.1186/gb-2010-11-1-r1 (PMC2847712; doi:10.1186/gb-2010-11-1-r1)
Supplement: Additional file 15 — The top level directory consists of a directory called Additional_cluster_files and 5010 directories, one for each multi-protein cluster family. (This top level directory has been split into three data files for uploading purposes (Additional files 15, 16, 17).) Within the directory are the following files: PGL1_unique_Yersinia_unclustered.out - list of all protein singletons that MCL did not group into a cluster (see Materials and Methods); PGL1_Yersinia_unique_locus_tags.txt - names of the 11 locus tag prefixes used for each genome; PGL1_unique_Yersinia.gff - mapping each Yersinia protein to a cluster in tab delimited GFF; PGL1_unique_Yersinia.sigfile - list of the longest protein in each cluster; PGL1_unique_Yersinia.summary - summary table of features of each of the clusters; PGL1_unique_Yersinia.table - summary table of each protein in the clusters. Within each cluster directory are the following files, where 'x' is the cluster name: PGL1_unique_Yersinia-x.faa - multifasta file of the proteins in the cluster; PGL1_unique_Yersinia-x.summary - summary of the properties of the proteins; PGL1_unique_Yersinia-x.matches - blast matches between the proteins of the cluster; PGL1_unique_Yersinia-x.muscle.fasta - muscle alignment of the proteins; PGL1_unique_Yersinia-x.muscle.fasta.gblo - gblocks output of muscle alignment (that is, auto-trimmed alignment); PGL1_unique_Yersinia-x.muscle.fasta.gblo.htm - as above in html format; PGL1_unique_Yersinia-x.muscle.tree - treefile from muscle alignment; PGL1_unique_Yersinia-x.sif - matches between proteins in simple interaction format for display on graphing software. [file gb-2010-11-1-r1-S15.zip › clusters/PGL1_unique_yersinia-CL1009/PGL1_unique_yersinia-CL1009.muscle.fasta.gblo.htm]

PGL1\_unique\_yersinia-CL1009.muscle.fasta


## Gblocks 0.91b Results

Processed file: **PGL1\_unique\_yersinia-CL1009.muscle.fasta**  
Number of sequences: **11**  
Alignment assumed to be: **Protein**  
New number of positions: **539** (selected positions are underlined in blue)

```
                         10        20        30        40        50        60
                 =========+=========+=========+=========+=========+=========+
yruck0001_1510   MSVKGITPQELASYGIHNVSEIVYNPSYELLFQEETKPGLEGYERGTVTNLGAVAVDTGI
ypseu0001X_4166  MSVKGITPQELAAYGIHNVSEIVYNPSYDLLFEEETKPTLEGYERGTLTTTGAIAVDTGI
ypest0001X_3370  MSVKGITPQELAAYGIHNVSEIVYNPSYDLLFEEETKPTLEGYERGTLTTTGAIAVDTGI
yinte0001_1530   MSVKGITPQELAAYGIHNVSEIVYNPSYDLLFQEETKPTLEGYERGILTTTGAIAVDTGI
yfred0001_1670   MSVKGITPQELAAYGIHNVSEIVYNPSYDLLFQEETKPTLEGYERGTLTTTGAIAVDTGI
ymoll0001_780    MSVKGITPQELAAYGIHNVSEIVYNPSYDLLFQEETKPTLEGYERGTLTTTGAIAVDTGI
yberc0001_1250   MSVKGITPQELAAYGIHNVSEIVYNPSYDLLFQEETKPTLEGYERGTLTTTGAIAVDTGI
yrohd0001_1780   MSVKGITPQELAAYGIHNVSEIVYNPSYDLLFQEETKPTLEGYERGTLTTTGAIAVDTGI
yaldo0001_1370   MSVKGITPQELAAYGIHNVSEIVYNPSYDLLFQEETKPTLKGYERGTLTTTGAIAVDTGI
yente0001X_2390  MSVKGITPQELAAYGIHNVSEIVYNPSYDLLFQEETKPTLEGYERGTLTNTGAIAVDTGI
ykris0001_1410   MSVKGITPQELAAYGIHNVSEIVYNPSYDLLFQEETKPTLEGYERGTLTTTGAIAVDTGI
                 ############################################################


                         70        80        90       100       110       120
                 =========+=========+=========+=========+=========+=========+
yruck0001_1510   FTGRSPKDKYIVRDAITQNTVWWADQGKGKNDNKPLSQETWSHLKELVTKQLSDKRLFVV
ypseu0001X_4166  FTGRSPKDKYIVRDAITQDTVWWADQGKGKNDNKPLSQEIWSHLKGLVTEQLSGKRLFVV
ypest0001X_3370  FTGRSPKDKYIVRDAITQDTVWWADQGKGKNDNKPLSQEIWNHLKGLVTEQLSGKRLFVV
yinte0001_1530   FTGRSPKDKYIVRDAITQDTVWWADQGKGKNDNKPLSQETWSHLKGLVTEQLSGKRLFVV
yfred0001_1670   FTGRSPKDKYIVRDDITRDTVWWADQGKGKNDNKPLSPEIWSHLKGLVTEQLSGKRLFVV
ymoll0001_780    FTGRSPKDKYIVRDAITQDTVWWADQGKGKNDNKPLSQETWSHLKELVTEQLSGKRLFVV
yberc0001_1250   FTGRSPKDKYIVRDAITQDTVWWADQGKGKNDNKPLSQETWSHLKGLVTEQLSGKRLFVV
yrohd0001_1780   FTGRSPKDKYIVRDDITRDTVWWADQGKGKNDNKPLSQETWAHLKGLVTQQLSGKRLFVV
yaldo0001_1370   FTGRSPKDKYIVRDAITQDTVWWADQGKGKNDNKPLSQETWSHLKGLVTEQLSGKRLFVV
yente0001X_2390  FTGRSPKDKYIVRDAITQDTVWWADQGKGKNDNKPLSQETWTHLKGLVTNQLSGKRLFVV
ykris0001_1410   FTGRSPKDKYIVRDAITQDTVWWADQGKGKNDNKPLSQETWAHLKGLVTNQLSGKRLFVV
                 ############################################################


                        130       140       150       160       170       180
                 =========+=========+=========+=========+=========+=========+
yruck0001_1510   DTFCGANADTRLKVRFITEVAWQAHFVKNMFIRPSDEELAHFEPDFIVMNGAKCTNPNWK
ypseu0001X_4166  DTFCGANADTRLQVRFITEVAWQAHFVKNMFIRPSDEELARFEPDFIVMNGAKCTNPQWK
ypest0001X_3370  DTFCGANADTRLQVRFITEVAWQAHFVKNMFIRPSDEELARFEPDFIVMNGAKCTNPQWK
yinte0001_1530   DTFCGANADTRLQVRFVTEVAWQAHFVKNMFIRPSDEELAHFEPDFIVMNGAKCTNPDWK
yfred0001_1670   DTFCGANADTRLQVRFVTEVAWQAHFVKNMFIRPSDEELAHFEPDFIVMNGAKCTNPNWK
ymoll0001_780    DTFCGANPDSRLRVRFVTEVAWQAHFVKNMFIRPTDEELADFEPDFIVMNGAKCTNPNWK
yberc0001_1250   DTFCGANADSRLQVRFVTEVAWQAHFVKNMFIRPTDEELADFEPDFIVMNGAKCTNPNWK
yrohd0001_1780   DTFCGANADTRLQVRFVTEVAWQAHFVKNMFIRPTDEELAHFEPDFIVMNGAKCTNPNWK
yaldo0001_1370   DTFCGANADTRLQVRFVTEVAWQAHFVKNMFIRPTDEELARFEPDFIVMNGAKCTNPDWK
yente0001X_2390  DTFCGANADTRLQVRFVTEVAWQAHFVKNMFIRPTDEELAHFEPDFIVMNGAKCINPNWK
ykris0001_1410   DTFCGANADTRLQVRFVTEVAWQAHFVKNMFIRPTDEELAHFEPDFIVMNGAKCTNPNWK
                 ############################################################


                        190       200       210       220       230       240
                 =========+=========+=========+=========+=========+=========+
yruck0001_1510   QQGLNSENFVAFNLTERMQLIGGTWYGGEMKKGMFSMMNYLLPLKGIASMHCSANVGEKG
ypseu0001X_4166  EQGLNSENFVAFNLTERMQLIGGTWYGGEMKKGMFSMMNYLLPLKGIASMHCSANVGEKG
ypest0001X_3370  EQGLNSENFVAFNLTERMQLIGGTWYGGEMKKGMFSMMNYLLPLKGIASMHCSANVGEKG
yinte0001_1530   EQGLNSENFVAFNLTERMQLIGGTWYGGEMKKGMFSMMNYLLPLKGIASMHCSANVGEKG
yfred0001_1670   EQGLNSENFVAFNLTERMQLIGGTWYGGEMKKGMFSMMNYLLPLKGIASMHCSANVGEKG
ymoll0001_780    EQGLNSENFVAFNLTERMQLIGGTWYGGEMKKGMFSMMNYLLPLKGIASMHCSANVGEKG
yberc0001_1250   EQGLNSENFVAFNLTERMQLIGGTWYGGEMKKGMFSMMNYLLPLKGIASMHCSANVGEKG
yrohd0001_1780   EQGLNSENFVAFNLTERMQLIGGTWYGGEMKKGMFSMMNYLLPLKGIASMHCSANVGEKG
yaldo0001_1370   EQGLNSENFVAFNLTERMQLIGGTWYGGEMKKGMFSMMNYLLPLKGIASMHCSANVGEKG
yente0001X_2390  EQGLNSENFVAFNLTERMQLIGGTWYGGEMKKGMFSMMNYLLPLKGIASMHCSANVGEKG
ykris0001_1410   EQGLNSENFVAFNLTERMQLIGGTWYGGEMKKGMFSMMNYLLPLKGIASMHCSANVGEKG
                 ############################################################


                        250       260       270       280       290       300
                 =========+=========+=========+=========+=========+=========+
yruck0001_1510   DVAIFFGLSGTGKTTLSTDPKRKLIGDDEHGWDDDGVFNFEGGCYAKTIKLSAEAEPDIY
ypseu0001X_4166  DVAIFFGLSGTGKTTLSTDPKRKLIGDDEHGWDDDGVFNFEGGCYAKTIKLSEEAEPDIY
ypest0001X_3370  DVAIFFGLSGTGKTTLSTDPKRKLIGDDEHGWDDDGVFNFEGGCYAKTIKLSEEAEPDIY
yinte0001_1530   DVAIFFGLSGTGKTTLSTDPKRKLIGDDEHGWDDDGVFNFEGGCYAKTIKLSEEAEPDIY
yfred0001_1670   DVAIFFGLSGTGKTTLSTDPKRKLIGDDEHGWDDDGVFNFEGGCYAKTIKLSEEAEPDIY
ymoll0001_780    DVAIFFGLSGTGKTTLSTDPKRKLIGDDEHGWDDDGVFNFEGGCYAKTIKLSEEAEPDIY
yberc0001_1250   DVAIFFGLSGTGKTTLSTDPKRKLIGDDEHGWDDDGVFNFEGGCYAKTIKLSEEAEPDIY
yrohd0001_1780   DVAIFFGLSGTGKTTLSTDPKRKLIGDDEHGWDDDGVFNFEGGCYAKTIKLSEEAEPDIY
yaldo0001_1370   DVAIFFGLSGTGKTTLSTDPKRKLIGDDEHGWDDDGVFNFEGGCYAKTIKLSEEAEPDIF
yente0001X_2390  DVAIFFGLSGTGKTTLSTDPKRKLIGDDEHGWDDDGVFNFEGGCYAKTIKLSEEAEPDIY
ykris0001_1410   DVAIFFGLSGTGKTTLSTDPKRKLIGDDEHGWDDDGVFNFEGGCYAKTIKLSEEAEPDIY
                 ############################################################


                        310       320       330       340       350       360
                 =========+=========+=========+=========+=========+=========+
yruck0001_1510   HAIKRDALLENVMVLADGTVDFNDGSKTENTRVSYPIYHIENIVKPVSKAGHANKVIFLT
ypseu0001X_4166  HAIKRDALLENVVVLADGTVDFNDGSKTENTRVSYPIYHIDNIVKPVSKAGHATKVIFLT
ypest0001X_3370  HAIKRDALLENVVVLADGTVDFNDGSKTENTRVSYPIYHIDNIVKPVSKAGHATKVIFLT
yinte0001_1530   HAIKRDALLENVVVLADGTVDFNDGSKTENTRVSYPIYHIENIVKPVSKAGHATKVIFLT
yfred0001_1670   HAIKRDALLENVVVLADGTVDFNDGSKTENTRVSYPIYHIENIVKPVSKAGHATKVIFLT
ymoll0001_780    HAIRRDALLENVVVLPDGTVDFNDSSKTENTRVSYPIYHIENIVKPVSKAGHATKVIFLT
yberc0001_1250   HAIRRDALLENVVVLPDGTVDFNDSSKTENTRVSYPIYHIENIVKPVSKAGHATKVIFLT
yrohd0001_1780   HAIKRDALLENVVVLPDGTVDFNDGSKTENTRVSYPIYHIENIVKPVSKAGHATKVIFLT
yaldo0001_1370   HAIKRDALLENVVVLADGTVDFNDGSKTENTRVSYPIYHIENIVKPVSKAGHATKVIFLT
yente0001X_2390  HAIKRDALLENVVVLPDGTVDFNDGSKTENTRVSYPIYHIENIVKPVSKAGHATKVIFLT
ykris0001_1410   HAIKRDALLENVVVLPDGTVDFNDGSKTENTRVSYPIYHIENIVKPVSKAGHATKVIFLT
                 ############################################################


                        370       380       390       400       410       420
                 =========+=========+=========+=========+=========+=========+
yruck0001_1510   ADAFGVLPPVSRLTADQTQYHFLSGFTAKLAGTERGVTEPTPTFSACFGAAFLSLHPTQY
ypseu0001X_4166  ADAFGVLPPVSRLTANQTQYHFLSGFTAKLAGTERGVTEPTPTFSACFGAAFLSLHPTQY
ypest0001X_3370  ADAFGVLPPVSRLTANQTQYHFLSGFTAKLAGTERGVTEPTPTFSACFGAAFLSLHPTQY
yinte0001_1530   ADAFGVLPPVSRLTANQTQYHFLSGFTAKLAGTERGVTEPTPTFSACFGAAFLSLHPTQY
yfred0001_1670   ADAFGVLPPVSRLTANQTQYHFLSGFTAKLAGTERGVTEPTPTFSACFGAAFLSLHPTQY
ymoll0001_780    ADAFGVLPPVSRLTANQTQYHFLSGFTAKLAGTERGVTEPTPTFSACFGAAFLSLHPTQY
yberc0001_1250   ADAFGVLPPVSRLTANQTQYHFLSGFTAKLAGTERGVTEPTPTFSACFGAAFLSLHPTQY
yrohd0001_1780   ADAFGVLPPVSRLTASQTQYHFLSGFTAKLAGTERGVTEPTPTFSACFGAAFLSLHPTQY
yaldo0001_1370   ADAFGVLPPVSRLTANQTQYHFLSGFTAKLAGTERGVTEPTPTFSACFGAAFLSLHPTQY
yente0001X_2390  ADAFGVLPPVSRLTANQTQYHFLSGFTAKLAGTERGVTEPTPTFSACFGAAFLSLHPTQY
ykris0001_1410   ADAFGVLPPVSRLTANQTQYHFLSGFTAKLAGTERGVTEPTPTFSACFGAAFLSLHPTQY
                 ############################################################


                        430       440       450       460       470       480
                 =========+=========+=========+=========+=========+=========+
yruck0001_1510   AEVLVKRMQAAGAQAYLVNTGWNGTGKRISIKDTRAIIDAILNGDIDKTETFTLPIFNLA
ypseu0001X_4166  AEVLVKRMQAVGAQAYLVNTGWNGTGKRISIKDTRAIIDAILNGEIDKAETFTLPIFDLA
ypest0001X_3370  AEVLVKRMQAVGAQAYLVNTGWNGTGKRISIKDTRAIIDAILNGEIDKAETFTLPIFDLA
yinte0001_1530   AEVLVKRMQAVGAQAYLVNTGWNGTGKRISIKDTRGIIDAILNGEIDKAETFTLPIFDLA
yfred0001_1670   AEVLVKRMQAVGAQAYLVNTGWNGTGKRISIKDTRGIIDAILNGEIDKAETFTLPIFDLA
ymoll0001_780    AEVLVKRMQAVGAQAYLVNTGWNGTGKRISIKDTRAIIDAILNGEIDKAETFTLPIFDLA
yberc0001_1250   AEVLVKRMQAVGAQAYLVNTGWNGTGKRISIKDTRAIIDAILNGEIDKAETFTLPIFDLA
yrohd0001_1780   AEVLLKRMQAVGAQAYLVNTGWNGTGKRISIKDTRGIIDAILNGEIDKAETFTLPIFDLA
yaldo0001_1370   AEVLVKRMQAVGAQAYLVNTGWNGTGKRISIKDTRAIIDAILNGEIDKAETFTLPIFDLA
yente0001X_2390  AEVLVKRMQAVGAQAYLVNTGWNGTGKRISIKDTRAIIDAILNGEIDKAETFTLPIFDLA
ykris0001_1410   AEVLVKRMQAVGAQAYLVNTGWNGTGKRISIKDTRAIIDAILNGEIDKAETFTLPIFDLA
                 ############################################################


                        490       500       510       520       530
                 =========+=========+=========+=========+=========+=========
yruck0001_1510   VPTALPGVNPDILDPRQTYGSIEQWQEKAEDLAKRFATNFDKYTDTPAGAALVSAGPKI
ypseu0001X_4166  VPMALPGVNPDILDPRDTYADKAQWQEKAEDLAKRFATNFDKYTDTPAGAALVSAGPKI
ypest0001X_3370  VPMALPGVNPDILDPRDTYADKAQWQEKAEDLAKRFATNFDKYTDTPAGAALVSAGPKI
yinte0001_1530   VPTALPGVNPDILDPRDTYADVAQWQEKAEDLAKRFTTNFDKYTDTPAGAALVSAGPKL
yfred0001_1670   VPTSLPGVNPDILDPRDTYADVAQWQEKAEDLAKRFTTNFDKYTDTPAGAALVSAGPKI
ymoll0001_780    VPMALPGVDPAILDPRDTYADVAQWQEKAENLAHRFTTNFDKYTDTPAGAALVSAGPKI
yberc0001_1250   VPMALPGVDPAILDPRDTYADIAQWQEKAEDLARRFTTNFDKYTDTPAGAALMSAGPKI
yrohd0001_1780   VPMALPGVNPDILDPRDTYADVAQWQEKAEDLAKRFTTNFDKYTDTPAGAALVSAGPKV
yaldo0001_1370   VPMALPGVDPAILDPRDTYADVAQWQEKAEDLAKRFTTNFDKYTDTPAGAALVSAGPKI
yente0001X_2390  VPMALPGVDPAILDPRDTYADVAQWQEKAEDLAKRFTTNFDKYTDTPAGAALVSAGPKI
ykris0001_1410   VPMALPGVDPAILDPRDTYADVAQWQEKAEDLAKRFTTNFDKYTDTPAGAALVSAGPKI
                 ###########################################################
```

```
Parameters used
Minimum Number Of Sequences For A Conserved Position: 6
Minimum Number Of Sequences For A Flanking Position: 9
Maximum Number Of Contiguous Nonconserved Positions: 8
Minimum Length Of A Block: 10
Allowed Gap Positions: With Half
Use Similarity Matrices: Yes
```

```
Flank positions of the 1 selected block(s)
Flanks: [1  539]  

New number of positions in PGL1_unique_yersinia-CLUSTERS.dir/PGL1_unique_yersinia-CL1009/PGL1_unique_yersinia-CL1009.muscle.fasta.gblo:  539  (100% of the original 539 positions)
```
