# Supplementary material for: Genomic characterization of the Yersinia genus
Source: Genome Biol. 2010 Jan 4;11(1):R1. doi: 10.1186/gb-2010-11-1-r1 (PMC2847712; doi:10.1186/gb-2010-11-1-r1)
Supplement: Additional file 15 — The top level directory consists of a directory called Additional_cluster_files and 5010 directories, one for each multi-protein cluster family. (This top level directory has been split into three data files for uploading purposes (Additional files 15, 16, 17).) Within the directory are the following files: PGL1_unique_Yersinia_unclustered.out - list of all protein singletons that MCL did not group into a cluster (see Materials and Methods); PGL1_Yersinia_unique_locus_tags.txt - names of the 11 locus tag prefixes used for each genome; PGL1_unique_Yersinia.gff - mapping each Yersinia protein to a cluster in tab delimited GFF; PGL1_unique_Yersinia.sigfile - list of the longest protein in each cluster; PGL1_unique_Yersinia.summary - summary table of features of each of the clusters; PGL1_unique_Yersinia.table - summary table of each protein in the clusters. Within each cluster directory are the following files, where 'x' is the cluster name: PGL1_unique_Yersinia-x.faa - multifasta file of the proteins in the cluster; PGL1_unique_Yersinia-x.summary - summary of the properties of the proteins; PGL1_unique_Yersinia-x.matches - blast matches between the proteins of the cluster; PGL1_unique_Yersinia-x.muscle.fasta - muscle alignment of the proteins; PGL1_unique_Yersinia-x.muscle.fasta.gblo - gblocks output of muscle alignment (that is, auto-trimmed alignment); PGL1_unique_Yersinia-x.muscle.fasta.gblo.htm - as above in html format; PGL1_unique_Yersinia-x.muscle.tree - treefile from muscle alignment; PGL1_unique_Yersinia-x.sif - matches between proteins in simple interaction format for display on graphing software. [file gb-2010-11-1-r1-S15.zip › clusters/PGL1_unique_yersinia-CL101/PGL1_unique_yersinia-CL101.muscle.fasta.gblo.htm]

PGL1\_unique\_yersinia-CL101.muscle.fasta


## Gblocks 0.91b Results

Processed file: **PGL1\_unique\_yersinia-CL101.muscle.fasta**  
Number of sequences: **23**  
Alignment assumed to be: **Protein**  
New number of positions: **186** (selected positions are underlined in blue)

```
                         10        20        30        40        50        60
                 =========+=========+=========+=========+=========+=========+
yruck0001_3360   VKIKTVVTLLITLLLTACDKPTPEVVESVRPVKIFTVEDSGQNGVRYFPARLQAGDET--
ypseu0001X_6900  ----------------------------VRPVKIFMVEDSGQNGTRYFPARLQAGDET--
ypest0001X_6240  ----------------------------VRPVKIFMVEDSGQNGTRYFPARLQAGDET--
yrohd0001_3590   ----------------------------VRPVKIFTVEDSGQNGTRYFPARLQAGDET--
yfred0001_3020   -------------------------VESVRPVKIFTVEDSGQNGTRYFPARLQAGDET--
ymoll0001_3130   VKIKTIVTLLVTFLLAACDRTPPEVVESVRPVKIFIVEDSGQNGTRYFPARLQAGDET--
yberc0001_3580   VKIKTVVTLLVTFLLAACDRTPPEVVESVRPVKIFIVEDSGQNGTRYFPARLQAGDET--
yaldo0001_4000   ------------------------------------------------------------
ykris0001_31540  -------------------------VESVRPVKIFIVEDSGQNGTRYFPARLQAGDET--
yente0001X_3786  -------------------------VESVRPVKIFIVEDSGQNGTRYFPARLQAGDET--
yaldo0001_3990   ----------------------------VRPVKVFTVEDSGQNGTRYFPARLQAGDET--
yinte0001_4130   VKIKTVITLLLTFLLAACDRTPPEVVESVRPVKIFTVEDSGQNGTRYFPARLQAGDET--
ymoll0001_28940  ----------------LNQEQKNALKPIKKIAVTFLVLLLVGSSLIYFGSRNDAVSVAQS
yruck0001_7910   ----------------------MNRKRIAVIVIVIALIAAAMYGWSDYRQQQDQSLTL--
ypseu0001X_1390  ----------------------MNRKKIIVAAVIVALLATLAYGWHYYRQQNDASLTL--
yaldo0001_9700   -----------------------------VAVVIVALLAAIGYGWSYYRQQHDASLTL--
yberc0001_9900   ----------------------------------------MGYGWRYYRQQHDSSLTL--
ymoll0001_8840   -----------------------------VAVVIVALLAAMGYGWSYYRQQHDSALTL--
yrohd0001_35570  -----------------------------VAVVIVALLAAIGYGWNYYRQQQDASLTL--
yinte0001_9300   -----------------------------VAVVIVALLAAVGYGWSYYRQQHDASLTL--
ykris0001_9430   -----------------------------VAVVIVAVLAAIGYGWNYYRQQQDATLTL--
yente0001X_1385  -----------------------------VAVVIVALLAAIGYGWSYYRQQQDATLTL--
yfred0001_8440   -----------------------------VAVVIVALLAAIGYGWSYYRQQHDASLTL--
                                                #######################      


                         70        80        90       100       110       120
                 =========+=========+=========+=========+=========+=========+
yruck0001_3360   -----------QLSF-KRSGQLQQLLVREGERVKKGQNIAMLNTTDLTLRVRDRQSTFNL
ypseu0001X_6900  -----------QLSF-KRGGQLQELLVREGEQVKKGQKIAMLNDTDLSLRVRDRQSTFNL
ypest0001X_6240  -----------QLSF-KRGGQLQELLVREGEQVKKGQKIAMLNDTDLSLRVRDRQSTFNL
yrohd0001_3590   -----------QLSF-KRSGQLQQLLVREGEQVKKGQTIAVLNDTDLNLRVRDRQSTFNL
yfred0001_3020   -----------QLSF-KRSGQLQQLLVREGEQVKKGQRIAMLNDTDLNLRVRDRQSTFNL
ymoll0001_3130   -----------QLSF-KRSGQLQQLLVREGEQVKKGQTIAKLNDTDLNLRVRDRQSTFNL
yberc0001_3580   -----------QLSF-KRSGQLQQLLVREGEQVKRGQVIAKLNDTDLNLRVRDRQSTFNL
yaldo0001_4000   ------------------------------------------------------------
ykris0001_31540  -----------QLSF-KRSGQLQQLLVREGEQVKKGQVIAKLNDTDLTLRVRDRQSTFNL
yente0001X_3786  -----------QLSF-KRSGQLQQLLVREGEQVKKGQVIAKLNDTDLTLRVRDRQSTFNL
yaldo0001_3990   -----------QLSF-KRGGQLQQLLVREGEQVKKGQVIARLNDTDLNLRVRDRQSTFNL
yinte0001_4130   -----------QLSF-KRSGQLQQLLVREGEQVKKGQVIARLNDTDLNLRVRDRQSTFNL
ymoll0001_28940  LKSGVLTADNINVAFENVGGKLITRHVQESQRVQKGDILMALDEVDTNISIERLKAVIRS
yruck0001_7910   --YGNVDIRTVNLGF-RVGGRLASLDVDEGDKIQPGQQLGQLDQGPFNNALKQAQANVES
ypseu0001X_1390  --YGNVDIRTVNLGF-RVAGRLASLAVDEGDDIHPGQTLGKLDDGPYLNALKQAQANVQS
yaldo0001_9700   --YGNVDIRTVNLGF-RVGGRLASLNVDEGDKVQPGQLLGKLDDGPYLNAFKQAQANVQN
yberc0001_9900   --YGNVDIRTVNLGF-RVGGRLASLAVDEGDELQPGQLLGKLDDGPYLNALKQAQANVQS
ymoll0001_8840   --YGNVDIRTVNLGF-RVGGRLASLAVDEGDDIQPGQQLGKLDDGPYLNALKQAQANVQS
yrohd0001_35570  --YGNVDVRTVNLGF-RVAGRLASLSVDEGDKIQPGQVLGKLDDGPYLNALKQAQANVQS
yinte0001_9300   --YGNVDIRTVNLGF-RVGGRLASLTVDEGDKIQPGEVLGKLDNGPYLNALKQAQANVQS
ykris0001_9430   --YGNVDIRTVNLGF-RVGGRLASLAVDEGDKVQPGEVLGKLDDGPYLNALKQAQANVQS
yente0001X_1385  --YGNVDIRTVNLGF-RVSGRLASLAVDEGDKIQPGEVLGKLDDGPYVNALKQAQANVQS
yfred0001_8440   --YGNVDIRTVNLGF-RVAGRLASLSVDEGDKIQPGQELGKLDDGPYLNALKQAQANVQS
                                    #########################################


                        130       140       150       160       170       180
                 =========+=========+=========+=========+=========+=========+
yruck0001_3360   ARDQFNRFNTLQG---------------RNAISRAELDIRRAELESAQAALDIARKELSD
ypseu0001X_6900  ARDQFNRFNTLQG---------------RSAVSRADLDIRRAEMESAQAALDIARKELSD
ypest0001X_6240  ARDQFNRFNTLQG---------------RSAVSRADLDIRRAEMESAQAALDIARKELSD
yrohd0001_3590   ARDQFNRFNTLQG---------------QRAVSRAELDVRRAEMESARAALEIAQKELSD
yfred0001_3020   ARDQFNRFNTLQG---------------QRAVSRAELDVRRAEMESARAALEIAQKELSD
ymoll0001_3130   ARDQFNRFNTLQG---------------QRAVSRAELDVRRAEMESARAGLEIAQKELSD
yberc0001_3580   ARDQFNRFNTLQG---------------QRAVSRAELDVRRAEMESARAGLEIAQKELSD
yaldo0001_4000   ----------LQG---------------QRAVSRAELDVRRAEMESARAALEIAQKELSD
ykris0001_31540  ARDQFNRFNTLQG---------------QRAVSRAELDVRRAEMESARAGLEIAQKELSD
yente0001X_3786  ARDQFNRFNTLQG---------------QRAVSRAELDVRRAEMESARAALEIAQKELSD
yaldo0001_3990   ARDQFNRF----------------------------------------------------
yinte0001_4130   ARDQFNRFNTLQG---------------QRAVSRAELDVRRAEMESARAALEIAQKELSD
ymoll0001_28940  QEASIRLEESATRIASDETKLTELSSWRKIEEIQATLSAARASEELARTDFNRAAKLSHT
yruck0001_7910   AQAQLALLQAGYR---DE----------EIAQVKSEVAQKQAAFNYADSFLKRQQGLWSS
ypseu0001X_1390  AQAQLALLKAGYR---EE----------EIAQVRSEVAQRQAAFDYADNFLKRQQGLWAS
yaldo0001_9700   AQAQLALLKAGYR---DE----------EIAQVRSEVSQREAAFSYADSFLKRQQGLWAS
yberc0001_9900   AQAQLALLKAGYR---DE----------EIAQVKSEVSQREAAFSYADSFLKRQQGLWAS
ymoll0001_8840   AQAQLALLKAGYR---DE----------EIAQVKSEVSQREAAFSYADSFLKRQQGLWAS
yrohd0001_35570  AQAQLALLKAGYR---DE----------EIAQVKSEVSQREAAFSYAESFLKRQQGLWAN
yinte0001_9300   AQAQLALLKAGYR---DE----------EIAQVKSEVAQREAAFSYADSFLKRQQGLWSS
ykris0001_9430   AQAQLDLLKAGYR---EE----------EIAQVRAEVSQREAAFSYADSFLKRQQGLWTS
yente0001X_1385  AQAQLALLKAGYR---EE----------EIAQVRSEVSQREAAFSYADSFLKRQQGLWAN
yfred0001_8440   AQAQLALLKAGYR---EE----------EIAQVKSEVSQREAAFSYADSFLKRQQGLWAN
                 ####                          ##############################


                        190       200       210       220       230       240
                 =========+=========+=========+=========+=========+=========+
yruck0001_3360   GTITA-PFDGVIANVAVRNHQVMAPGQVVATLSA--------------------------
ypseu0001X_6900  ATIIA-PFDGIIANVNVRNHQVMAAGQPVATLSA--------------------------
ypest0001X_6240  ATIIA-PFDGIIANVNVRNHQVMAAGQPVATLSA--------------------------
yrohd0001_3590   ATITA-PFDGVIANVNVRNHQVMAPGQPVATLSA--------------------------
yfred0001_3020   ATITA-PFDGIIANVNVRNHQVMAPGQPVATLSA--------------------------
ymoll0001_3130   ATITA-PFDGIIANVSARNHQVMAPGQAVATLSA--------------------------
yberc0001_3580   ATITA-PFDGIIANVSARNHQVMAPGQAVATLSA--------------------------
yaldo0001_4000   ATITA-PFDGIIANVNVRNHQVMAAGQPVATLSA--------------------------
ykris0001_31540  ATITA-PFDGIIANVNARNHQVMAPGQAVATLSA--------------------------
yente0001X_3786  ATITA-PFDGIIANVNARNHQVMAPGQPVATLSA--------------------------
yaldo0001_3990   ------------------------------------------------------------
yinte0001_4130   ATITA-PFDGIIANVNVRNHQVMAPGQAVATLSA--------------------------
ymoll0001_28940  GSVSQSMLDNARSTLTQTHSAVVQAERQLASAMIGTTPEQMKRLAEKASAQGMTLQAIAN
yruck0001_7910   KATSANDLDDARTSRNQAQAALQAAKDKLAQFLSGNRPQE--------------------
ypseu0001X_1390  KAVSANELENARTARNQARANLQAAKDKLAQFLSGNRPQE--------------------
yaldo0001_9700   KATSANELENARTARNQAQANLQASKDKLAQYLSGNRPQE--------------------
yberc0001_9900   KATSANELENARTARNQAQANLQASKDKLAQYLSGNRPQE--------------------
ymoll0001_8840   KATSANELENARTARNQAQANLQASKDKLAQYLSGNRPQE--------------------
yrohd0001_35570  KATSANDLENARTARNQAQANLQASKDKLAQYLSGNRPQE--------------------
yinte0001_9300   KATSANELENARTARNQAQANLQASKDKLAQYLSGNRPQE--------------------
ykris0001_9430   KAVSANELENARTARSQAQANLQASKDKLAQYLSGNRPQE--------------------
yente0001X_1385  KATSANELENARTARNQAQANLQASKDKLAQYLSGNRPQE--------------------
yfred0001_8440   KATSANELENARTARNQAQANLQASKDKLAQYLSGNRPQE--------------------
                 #####   ######################                              


                        250       260       270       280       290       300
                 =========+=========+=========+=========+=========+=========+
yruck0001_3360   -----------LDSLDVVFSVPERLFTALDIGNKDYHPMVRLNHLPDREFVAQYKEHTTS
ypseu0001X_6900  -----------LDTLDVVFSVPERLFTTLDISNRNYKPTVLLNHMPGREFIAEYKEHTTS
ypest0001X_6240  -----------LDTLDVVFSVPERLFTTLDISNRNYKPTVLLNHMPGREFIAEYKEHTTS
yrohd0001_3590   -----------LDSLDVIFSVPERLFTTLDISNRSYKPTVLLNHLPGREFTAEYKEHTTS
yfred0001_3020   -----------LDTLDVVFSVPERLFTTLDISNRSYRPTVLLNHLPGREFIAEYKEHTTS
ymoll0001_3130   -----------LDTLDVVFSVPERLFTTLDISNRNYKPTVLLNHLPGREFIAEYKEHTTS
yberc0001_3580   -----------LDTLDVVFSVPERLFTTLDISNRNYKPTVLLNHMPGREFIAEYKEHTTT
yaldo0001_4000   -----------LDTLDVVFSVPERLFTTLEISNRTYKPTVLLNHMPGREFIAEYKEHTTS
ykris0001_31540  -----------LDTLDVVFSVPERLFTTLDISNRNYKPTVLLNHMPGREFIAEYKEHTTS
yente0001X_3786  -----------LDTLDVVFSVPERLFTTLDISNRNYKPTVLLNHLPGREFVAEYKEHTTS
yaldo0001_3990   ------------------------------------------------------------
yinte0001_4130   -----------LDTLDVVFSVPERLFTTLDISNRTYKPTVLLNHMPGREFIAEYKEHTTS
ymoll0001_28940  SRESIKNRENVLDQLRAQLAQSQAELKQLEV---NHSRLTLTAPADGKILKLLYEPGEIV
yruck0001_7910   -----------IAQAAANVAQAEAELAQAQL---NLHDTSLISPSSGTILTRAVEPGTIL
ypseu0001X_1390  -----------IAQAEANLAQTEAELAQAQL---NLQDTILLAPSAGTVLTRAVEPGTIL
yaldo0001_9700   -----------IAQAEANLAQSEAELAQAQL---NLHDTTLLSPSAGTVLTRAVEPGTIL
yberc0001_9900   -----------IAQAEANLAQAEAELAQAQL---NLQDTILLSPSAGTVLTRAVEPGTIL
ymoll0001_8840   -----------IAQAEANLAQAEAELAQAQL---NLQDTTLLSPSAGTVLTRAVEPGTIL
yrohd0001_35570  -----------IAQAEANLAQAEAAQAQAQL---NLQDTLLLSPSTGTVLTRAVEPGTIL
yinte0001_9300   -----------IAQAEANLAQSEAELAQAQL---NLQDTTLLAPSAGTVLTRAVEPGTIL
ykris0001_9430   -----------IAQAEANLAQSEAELAQAQL---NLQDTTLLSPSGGTVLTRAVEPGTIL
yente0001X_1385  -----------IAQAEANLAQSEAELAQAQL---NLQDTTLLSPSGGTVLTRAVEPGTIL
yfred0001_8440   -----------IAQAEANLAQSEAELAQAQL---NLQDTTLLSPSSGTVLTRAVEPGTIL
                                                   ##########################


                        310       320       330       340       350       360
                 =========+=========+=========+=========+=========+=========+
yruck0001_3360   TSAGSMTFQVTLTMKRPPDLPLLSGISGSVRINTNKLTGSAHPSIIVPVEAVFNPDSSQL
ypseu0001X_6900  TTSASQTFQVTLTMKRPADMPLLSGISGRVRINSGNLSGTDHPTIIVPVEAVFNPDHAQL
ypest0001X_6240  TTSASQTFQVTLTMKRPADMPLLSGISGRVRINSGNLSGTDHPTIIVPVEAVFNPDHAQL
yrohd0001_3590   TSSASQTFQVTLTMKRPADMPLLSGISGRVRINSGSLSGTDHPTIVIPAEAVFNPDSAQL
yfred0001_3020   TTSASQTFQVTLTMKRPPDMPLLSGISGRVRINSGNLSGTAQPTIVVPVEAVFNPDSTQL
ymoll0001_3130   TSSASQTFQVTLTMKRPADMPLLSGISGRVRINSGNLSGTDHPTIVVPVEAVFNPDNTQL
yberc0001_3580   TSSASQTFQVTLTMKRPADMPLLSGISGRVRINSGTLSGTDHPTIVVPVEAVFNPDSAQL
yaldo0001_4000   TTSASQTFQVTLTMKRPADMPLLSGISGRVRINSGNLSGNDHPTIIVPVEAVFNPDSAQL
ykris0001_31540  TTSASQTFQVTLTMKRPADMPLLSGISGRVRINSGNLSGTDHPTIVVPVEAVFNPDSAQL
yente0001X_3786  TTSASQTFQVTLTMKRPPDMPLLSGISGRVRINSGNLSGTDHPTIVIPVEAVFNPDSAQL
yaldo0001_3990   ------------------------------------------------------------
yinte0001_4130   TTSASQTFQVTLTMKRPADMPLLSGISGRVRINSGNLSGTDHPTIIVPVEAVFNPDSAQL
ymoll0001_28940  PTGAPA---VLLE-TDH--------RYVDIYVNENMV-SAYQPGTAVTAQVPA-LD----
yruck0001_7910   SAGNTV---FTLSLTNP--------VWVRAYVDERHL-GQAVPGTEVDVYTDSRPG----
ypseu0001X_1390  SASNTV---FTVSLTDP--------VWVRAYVSERHL-GQAIPGSEVEVFTDGRPD----
yaldo0001_9700   SASNTV---FTLSLTHP--------VWVRAYVSEPHL-SLAIPGTQVEVFTDGRPD----
yberc0001_9900   SASNTV---FTLSLTDP--------VWVRAYVSERNL-DRAIPGTQVEVFTDGRPD----
ymoll0001_8840   SASNTV---FTLSLTDP--------VWVRAYVSECNL-DKAIPGTQVEVFTDGRPD----
yrohd0001_35570  SASNTV---FTLSLTDP--------VWVRAYISEPHL-GQAIPGTKVEVFTDGRPN----
yinte0001_9300   SASNTV---FTLSLTDP--------VWVRAYVSEPHL-NQAIPGTEVDVFTDGRPG----
ykris0001_9430   SASNTV---FTLSLTDP--------VWVRAYVSERHL-GQAIPGTEVEIFTDGRPG----
yente0001X_1385  SASNTV---FTLSLTDP--------VWVRAYVSERHL-GQAIPGTEVEVFTDGRPD----
yfred0001_8440   SASNTV---FTLSLTDP--------VWVRAYVSEPHL-NQAIPGTEVEIFTDGRPN----
                 ###                                   #################     


                        370       380       390       400       410       420
                 =========+=========+=========+=========+=========+=========+
yruck0001_3360   NDARVWVVKEDKGQLHVEERKVQVGQLTANGILITSGLA--DGEQIVAAGTSELRPQQLV
ypseu0001X_6900  NDARVWVIKNDNDQMHVEERKVQVGQLVENGIQVTSGLA--DGEQIVAAGTGELRPQQVV
ypest0001X_6240  NDARVWVIKNDNDQMHVEERKVQVGQLVENGIQVTSGLA--DGEQIVAAGTGELRPQQVV
yrohd0001_3590   NDARVWVIKNDDGQMRVEERKVQVGQLTANGIQITSGLT--DGEHIVAAGTGELRPNQVV
yfred0001_3020   NDARVWVIKNDDGQMHVEERNVQVGQLTANGIQITSGLA--DGEQIVSAGTGELRPNQIV
ymoll0001_3130   NDARVWVIKNDDGQMHVEERKVQVGQLTENGIQVTSGLV--DGEQIVAAGTGELRPNQVV
yberc0001_3580   NDARVWVIKSDDGQMHVEERKVQVGQLTENGIQVTSGLA--DGEQIVSAGTGELRPNQVV
yaldo0001_4000   NDARVWVIKNDDGQMHVEARKVQVGQLTANGIQVISGLA--DGEQIVAAGTGELRPNQVV
ykris0001_31540  NDARVWVIKNDEGQMHVEERKVQVGQLTANGIQITSGLA--DGEQIVAAGTGELRPNQVV
yente0001X_3786  NDARVWVIKNDDGQMHVEERKVQVGQLTANGIQITSGLA--DGEQIVAAGTGELRPNQVV
yaldo0001_3990   ------------------------------------------------------------
yinte0001_4130   NDARVWVIKNDDGQMHVEERKVQVGQLTANGIQITSGLA--DGEQIVSAGTGELRPNQVV
ymoll0001_28940  --------TQVKGVVRFANAAPSFSDLRMTRERGQADLTSYQVRIY-TEVKPQLITGMTL
yruck0001_7910   --------KPYRGQIGFVSPTAEFTPKTVETPDLRTDLV-YRLRIIVTDADSELRQGMPV
ypseu0001X_1390  --------KPYHGKIGFVSPTAEFTPKTVETPDLRTDLV-YRLRIIITDADESLRQGMPV
yaldo0001_9700   --------KPYHGKVGFVSPTAEFTPKTVETPDLRTDLV-YRLRIIITDADESLRQGMPV
yberc0001_9900   --------KPYHGKIGFVSPTAEFTPKSVETPDLRTDLV-YRLRIIITDADESLRQGMPV
ymoll0001_8840   --------KPYHGKIGFVSPTAEFTPKSVETPELRTDLV-YRLRIIITDADESLRQGMPV
yrohd0001_35570  --------KPYHGQIGFVSPTAEFTPKSVETPDLRTDLV-YRLRIVITDADESLRQGMPV
yinte0001_9300   --------KPYHGKIGFVSPTAEFTPKSVETPELRTDLV-YRLRIIITDADESLRQGMPV
ykris0001_9430   --------KPYHGKIGFVSPTAEFTPKSVETPDLRTDLV-YRLRIIITDADESLRQGMPV
yente0001X_1385  --------KPYHGKIGFVSPTAEFTPKSVETPDLRTDLV-YRLRIIITDADESLRQGMPV
yfred0001_8440   --------KPYHGKIGFVSPTAEFTPKSVETPDLRTDLV-YRLRIIITDADESLRQGMPV
                                                              ###############


                 
                 =========
yruck0001_3360   RAWVRERGL
ypseu0001X_6900  RAWVRERGL
ypest0001X_6240  RAWVRERGL
yrohd0001_3590   RAWVRERGL
yfred0001_3020   RAWVRERGL
ymoll0001_3130   RAWVRERGL
yberc0001_3580   RAWVRERGL
yaldo0001_4000   RAWVRERGL
ykris0001_31540  RAWVRERGL
yente0001X_3786  RAWVRERGL
yaldo0001_3990   ---------
yinte0001_4130   RAWVRERGL
ymoll0001_28940  EVDDAQHR-
yruck0001_7910   TIRFSQP--
ypseu0001X_1390  TVRFPQR--
yaldo0001_9700   TVRFVQP--
yberc0001_9900   TVRFAQP--
ymoll0001_8840   TVRFAQP--
yrohd0001_35570  TVRFVQP--
yinte0001_9300   TIRFAQP--
ykris0001_9430   TISFAQP--
yente0001X_1385  TVRFVQP--
yfred0001_8440   TVRFAQP--
```

```
Parameters used
Minimum Number Of Sequences For A Conserved Position: 12
Minimum Number Of Sequences For A Flanking Position: 19
Maximum Number Of Contiguous Nonconserved Positions: 8
Minimum Length Of A Block: 10
Allowed Gap Positions: With Half
Use Similarity Matrices: Yes
```

```
Flank positions of the 7 selected block(s)
Flanks: [32  54]  [80  124]  [151  185]  [189  210]  [275  303]  [339  355]  [406  420]  

New number of positions in PGL1_unique_yersinia-CLUSTERS.dir/PGL1_unique_yersinia-CL101/PGL1_unique_yersinia-CL101.muscle.fasta.gblo:  186  (43% of the original 429 positions)
```
