# Supplementary material for: Genomic characterization of the Yersinia genus
Source: Genome Biol. 2010 Jan 4;11(1):R1. doi: 10.1186/gb-2010-11-1-r1 (PMC2847712; doi:10.1186/gb-2010-11-1-r1)
Supplement: Additional file 15 — The top level directory consists of a directory called Additional_cluster_files and 5010 directories, one for each multi-protein cluster family. (This top level directory has been split into three data files for uploading purposes (Additional files 15, 16, 17).) Within the directory are the following files: PGL1_unique_Yersinia_unclustered.out - list of all protein singletons that MCL did not group into a cluster (see Materials and Methods); PGL1_Yersinia_unique_locus_tags.txt - names of the 11 locus tag prefixes used for each genome; PGL1_unique_Yersinia.gff - mapping each Yersinia protein to a cluster in tab delimited GFF; PGL1_unique_Yersinia.sigfile - list of the longest protein in each cluster; PGL1_unique_Yersinia.summary - summary table of features of each of the clusters; PGL1_unique_Yersinia.table - summary table of each protein in the clusters. Within each cluster directory are the following files, where 'x' is the cluster name: PGL1_unique_Yersinia-x.faa - multifasta file of the proteins in the cluster; PGL1_unique_Yersinia-x.summary - summary of the properties of the proteins; PGL1_unique_Yersinia-x.matches - blast matches between the proteins of the cluster; PGL1_unique_Yersinia-x.muscle.fasta - muscle alignment of the proteins; PGL1_unique_Yersinia-x.muscle.fasta.gblo - gblocks output of muscle alignment (that is, auto-trimmed alignment); PGL1_unique_Yersinia-x.muscle.fasta.gblo.htm - as above in html format; PGL1_unique_Yersinia-x.muscle.tree - treefile from muscle alignment; PGL1_unique_Yersinia-x.sif - matches between proteins in simple interaction format for display on graphing software. [file gb-2010-11-1-r1-S15.zip › clusters/PGL1_unique_yersinia-CL1010/PGL1_unique_yersinia-CL1010.muscle.fasta.gblo.htm]

PGL1\_unique\_yersinia-CL1010.muscle.fasta


## Gblocks 0.91b Results

Processed file: **PGL1\_unique\_yersinia-CL1010.muscle.fasta**  
Number of sequences: **11**  
Alignment assumed to be: **Protein**  
New number of positions: **293** (selected positions are underlined in blue)

```
                         10        20        30        40        50        60
                 =========+=========+=========+=========+=========+=========+
yruck0001_1520   ------------------MSNHDQLHRYLFNHHAVRGELVTVNETYQQVVANHDYPAPVK
ypseu0001X_4165  ------------------MSNHDQLHRYLFANHAVRGELVSVNETYQQVLANHDYPPAVQ
ypest0001X_3380  ------------------MSNHDQLHRYLFANHAVRGELVSVNETYQQVLANHDYPPAVQ
yrohd0001_1790   ------------------MSNHDQLHRYLFANHAVRGELVSVNETYQQVLANHDYPPAVQ
yaldo0001_1380   ------------------MSNHDQLHRYLFANHAVRGELVSVNETYQQVLANHDYPPAVQ
ymoll0001_790    ------------------MSNHDQLHRYLFANHAVRGELVSVNETYQQVLANHDYPPAVQ
yberc0001_1260   ------------------MSNHDQLHRYLFTNHAVRGELVSVNETYQQVLANHDYPPAVK
ykris0001_1420   LQPEREKTMS-----NHDQSNHDQLHRYLFANHAVRGELVSVNETYQQVLANHDYPPAVQ
yente0001X_2400  LQPEREKPMSNHDQANHDQSNHDQLQRYLFANHAVRGELVSVNETYQQVLANHDYPPAVQ
yinte0001_1540   LQPEREKP----------MSNHDQLHRYLFANHAVRGELVSVNETYQQVLANHDYPPAVQ
yfred0001_1680   ------------------MSNHDQLHRYLFANHAVRGELVSVNETYQQVLANHDYPPAVQ
                                   ##########################################


                         70        80        90       100       110       120
                 =========+=========+=========+=========+=========+=========+
yruck0001_1520   KLLGEMLVATSLLTATLKFDGNITVQLQG-DGPLTLAVINGNNQQELRGVARTNGEISAE
ypseu0001X_4165  KLLGEMLVATSLLTATLKFDGDITVQLQGGDGPLTLAVINGNNRQEMRGVARVKGEISDD
ypest0001X_3380  KLLGEMLVATSLLTATLKFDGDITVQLQGGDGPLTLAVINGNNRQEMRGVARVKGEISDD
yrohd0001_1790   KLLGEMLVATSLLTATLKFDGDITVQLQGGDGPLSLAVINGNNRQEMRGVARFKGEISDE
yaldo0001_1380   KLLGEMLVATSLLTATLKFEGDITVQLQGGDGPLTLAVINGNNRQEMRGVARFKGEISDE
ymoll0001_790    KLLGEMLVATSLLTATLKFDGDITVQLQSTDGPLTLAVINGNNQQEMRGVARFKGEISDE
yberc0001_1260   KLLGEMLVATSLLTATLKFDGDITVQLQSTDGPLTLAVINGNNQQEMRGVARFKGEISDE
ykris0001_1420   KLLGEMLVATSLLTATLKFDGDITVQLQGGEGPLSLAVINGNNQQEMRGVARFKGEISDE
yente0001X_2400  KLLGEMLVATSLLTATLKFDGDITVQLQGGEGPLSLAVINGNNQQEMRGVARFKGEISDE
yinte0001_1540   KLLGEMLVATSLLTATLKFDGDITVQLQGADGPLSLAVINGNNQQQMRGVARFKGEISDE
yfred0001_1680   KLLGEMLVATSLLTATLKFDGDITVQLQGGDGPLSLAVINGNNQQEMRGVARYKGEISDE
                 ############################################################


                        130       140       150       160       170       180
                 =========+=========+=========+=========+=========+=========+
yruck0001_1520   SSLKQMVGN-GYMVITITPTKGERYQGVVGLEGETLAECLESYFMQSEQLPTRLFIRTGE
ypseu0001X_4165  STLQEMVGN-GYLVITITPAQGERYQGVVALEGETIAACLENYFMQSEQLPTRLFIRTGH
ypest0001X_3380  STLQEMVGN-GYLVITITPAQGERYQGVVALEGETIAACLENYFMQSEQLPTRLFIRTGH
yrohd0001_1790   STLKEMVGN-AYLVITITPEQGERYQGVVALEGDTIAACLESYFMQSEQLPTRLFIRTGN
yaldo0001_1380   STLKEMVGN-GCLVITITPARGERYQGVVALEGDTIAACLESYFMQSEQLPTRLFIRTGN
ymoll0001_790    STLKEMVGN-GYLVITITPAKGERYQGVVALEGETIAACLESYFMQSEQLPTRLFIRTGE
yberc0001_1260   STLKEMVGN-GYLVITITPAKGERYQGVVALEGETIGACLESYFMQSEQLPTRLFIRTGE
ykris0001_1420   STLKEMMGNSGYLVITITPAQGERYQGVVALEGETIAACLENYFMQSEQLPTRLFIRTGS
yente0001X_2400  STLKEMMGNNGYLVITITPAQGERYQGVVALEGETIAACLENYFMQSEQLPTRLFIRTGD
yinte0001_1540   STLKEMVGN-GYLVITITPAKGERYQGVVALEGETIAACLENYFMQSEQLPTRLFIRTGN
yfred0001_1680   STLKEMVGN-GYLVITITPAKGERYQGVVALEGDTIAACLENYFMQSEQLPTRLFIRTGS
                 ######### ##################################################


                        190       200       210       220       230       240
                 =========+=========+=========+=========+=========+=========+
yruck0001_1520   VDGKVAAAGMLLQVLPAEERNEDEFDHLAQLTTTIKAEELFTLPANEVLYRLYHQEEVTL
ypseu0001X_4165  VADKAAAGGMLLQVLPAQERNEDEFDHLAQLTATIKAEELFTLPANEVLYRLYHQEEVTL
ypest0001X_3380  VADKAAAGGMLLQVLPAQERNEDEFDHLAQLTATIKAEELFTLPANEVLYRLYHQEEVTL
yrohd0001_1790   VAGKAAAGGMLLQVLPAEERNEDEFDHLAQLTATIKAEELFTLPANEVLYRLYHQEEVTL
yaldo0001_1380   TEGKAAAGGMLLQVLPAQERNEDEFDHLAQLTATIKAEELFTLPANEVLYRLYHQEEVTL
ymoll0001_790    VSGKAAAGGMLLQVLPAQERNEDEFDHLAQLTTTIKAEELFTLPANEVLYRLYHQEEVTL
yberc0001_1260   VEGKAAAGGMLLQVMPAQERNEDEFDHLAQLTTTIKAEELFTLPANEVLYRLYHQEEVTL
ykris0001_1420   VEGKAAAGGMLLQVLPAQERNEDEFDHLAQLTTTIKAEELFTLPANEVLYRLYHQEEVTL
yente0001X_2400  VEGKPAAGGMLLQVLPAQERNEDEFDHLAQLTTTIKAEELFTLPANEVLYRLYHQEEVTL
yinte0001_1540   VEGKAAAGGMLLQVLPAEERNEDEFDHLAQLTTTIKAEELFTLPANEVLYRLYHQEEVTL
yfred0001_1680   VEGKAAAGGMLLQVLPAQERNEDEFDHLAQLTTTIKAEELFTLPANEVLYRLYHQEEVTL
                 ############################################################


                        250       260       270       280       290       300
                 =========+=========+=========+=========+=========+=========+
yruck0001_1520   YEPQNVSFRCTCSRERCADALATLPAEEVQELLEQDGKIDMHCEYCGTHHLFDAVDISRL
ypseu0001X_4165  YEPQNVSFRCTCSRQRCADALVTLADDDVTEMLEQDGNIDMHCEYCGNHYLFDAVDIATL
ypest0001X_3380  YEPQNVSFRCTCSRQRCADALVTLADDDVTEMLEQDGNIDMHCEYCGNHYLFDAVDIATL
yrohd0001_1790   YEPQNVSFRCTCSRTRCADALVTLSEDDINEMLAQDGNIDMHCEYCGSHYLFDAVDIATL
yaldo0001_1380   YEPQNVSFRCTCSRERCADALVTLSEDDVNEMLEQDGNIDMHCEYCGNHYLFDAVDIASL
ymoll0001_790    YEPQNVSFRCTCSRERCADALVTLSEEDISEMLEQDGNIDMHCEYCGNHYLFDAVDIASI
yberc0001_1260   YEPQNVSFRCTCSRERCADALVTLSEDDIKEMLEQDGNIDMHCEYCGNHYLFDAVDIASI
ykris0001_1420   YEPQNVSFRCTCSRERCADALVTLSEDDVKEMLELDGNIDMNCEYCGTHYLFDAVDIAAL
yente0001X_2400  YEPQNVSFRCTCSRERCADALVTLSEDDVKEMLEQDGNIDMNCEYCGTHYLFDAVDIATL
yinte0001_1540   YEPQNVSFRCTCSRERCADALVTLSEDDITEMLEQDGNIDMHCEYCGNHYLFDAVDIASL
yfred0001_1680   YEPQNVSFRCSCSRERCADALITLSEDDIKEMLEQDGNIDMHCEYCGNHYLFDAVDIATL
                 ############################################################


                        310
                 =========+==
yruck0001_1520   LGGNSPADTQIH
ypseu0001X_4165  KNGNSASSEQIH
ypest0001X_3380  KNGNSASSEQIH
yrohd0001_1790   KSGNSPSSEQIH
yaldo0001_1380   KSGNSSSSEQIH
ymoll0001_790    KNGNSTSSDQMH
yberc0001_1260   KSGNSASSEQIH
ykris0001_1420   KSGNSPSPEQIH
yente0001X_2400  KSGNSPSSEQIH
yinte0001_1540   KSGNSSSSEQIH
yfred0001_1680   KSGNSSSSEQIH
                 ############
```

```
Parameters used
Minimum Number Of Sequences For A Conserved Position: 6
Minimum Number Of Sequences For A Flanking Position: 9
Maximum Number Of Contiguous Nonconserved Positions: 8
Minimum Length Of A Block: 10
Allowed Gap Positions: With Half
Use Similarity Matrices: Yes
```

```
Flank positions of the 2 selected block(s)
Flanks: [19  129]  [131  312]  

New number of positions in PGL1_unique_yersinia-CLUSTERS.dir/PGL1_unique_yersinia-CL1010/PGL1_unique_yersinia-CL1010.muscle.fasta.gblo:  293  (93% of the original 312 positions)
```
