# Supplementary material for: Genomic characterization of the Yersinia genus
Source: Genome Biol. 2010 Jan 4;11(1):R1. doi: 10.1186/gb-2010-11-1-r1 (PMC2847712; doi:10.1186/gb-2010-11-1-r1)
Supplement: Additional file 15 — The top level directory consists of a directory called Additional_cluster_files and 5010 directories, one for each multi-protein cluster family. (This top level directory has been split into three data files for uploading purposes (Additional files 15, 16, 17).) Within the directory are the following files: PGL1_unique_Yersinia_unclustered.out - list of all protein singletons that MCL did not group into a cluster (see Materials and Methods); PGL1_Yersinia_unique_locus_tags.txt - names of the 11 locus tag prefixes used for each genome; PGL1_unique_Yersinia.gff - mapping each Yersinia protein to a cluster in tab delimited GFF; PGL1_unique_Yersinia.sigfile - list of the longest protein in each cluster; PGL1_unique_Yersinia.summary - summary table of features of each of the clusters; PGL1_unique_Yersinia.table - summary table of each protein in the clusters. Within each cluster directory are the following files, where 'x' is the cluster name: PGL1_unique_Yersinia-x.faa - multifasta file of the proteins in the cluster; PGL1_unique_Yersinia-x.summary - summary of the properties of the proteins; PGL1_unique_Yersinia-x.matches - blast matches between the proteins of the cluster; PGL1_unique_Yersinia-x.muscle.fasta - muscle alignment of the proteins; PGL1_unique_Yersinia-x.muscle.fasta.gblo - gblocks output of muscle alignment (that is, auto-trimmed alignment); PGL1_unique_Yersinia-x.muscle.fasta.gblo.htm - as above in html format; PGL1_unique_Yersinia-x.muscle.tree - treefile from muscle alignment; PGL1_unique_Yersinia-x.sif - matches between proteins in simple interaction format for display on graphing software. [file gb-2010-11-1-r1-S15.zip › clusters/PGL1_unique_yersinia-CL1012/PGL1_unique_yersinia-CL1012.muscle.fasta.gblo.htm]

PGL1\_unique\_yersinia-CL1012.muscle.fasta


## Gblocks 0.91b Results

Processed file: **PGL1\_unique\_yersinia-CL1012.muscle.fasta**  
Number of sequences: **11**  
Alignment assumed to be: **Protein**  
New number of positions: **224** (selected positions are underlined in blue)

```
                         10        20        30        40        50        60
                 =========+=========+=========+=========+=========+=========+
yruck0001_1540   -----------VIMPWDVNWQDVDTVLLDMDGTLLDLEFDSYFWLRLVPETLSQRRNIPL
ypseu0001X_4163  -------------MPPDFNWQEIDTVLLDMDGTLLDLAFDSHFWLKLVPETLSQRRGIPL
ypest0001X_3400  -------------MPPDFNWQEIDTVLLDMDGTLLDLAFDSHFWLKLVPETLSQRRGIPL
yaldo0001_1400   -------------MPPDLNWQEIDTVLLDMDGTLLDLEFDSHFWLKQVPMALSEHRGIPL
yberc0001_1280   -------------MPPELNWQEIDTVLLDMDGTLLDLEFDSHFWLKQVPERLSQHRGIPL
ymoll0001_810    -------------MPPEFNWQEIDTVLLDMDGTLLDLEFDSHFWLKQVPETLSQRRGIPL
yrohd0001_1810   -------------MPAEFNWQEIDTVLLDMDGTLLDLEFDSHFWLKQVPETLSQHRGISL
yinte0001_1560   -------------MPPELNWQEIDTVLLDMDGTLLDLEFDSHFWLKQVPETLSQHRGIPL
yfred0001_1700   -------------MPPEFNWQEIDTVLLDMDGTLLDLEFDSHFWLKQVPETLSQHRGISL
yente0001X_2420  -------------MPPELNWQEIDTVLLDMDGTLLDLEFDSHFWLKQVPETLSQRRGISL
ykris0001_1440   MTGYHHYCDCGAAMPPEFNWQEIDTVLLDMDGTLLDLEFDSHFWLKQVPETLSQQRGISL
                              ###############################################


                         70        80        90       100       110       120
                 =========+=========+=========+=========+=========+=========+
yruck0001_1540   AQAKAIINDEYHAVQHTLNWYCFDYWSQRLDLDIYAMTTQVGPRASLREDTLPFLQGLRD
ypseu0001X_4163  EQAHKIIHDEYNAVQHTLNWYCFDYWRERLDLDIYAMTTDIGSRARLRQDTVPFLSGLRQ
ypest0001X_3400  EQAHKIIHDEYNAVQHTLNWYCFDYWRERLDLDIYAMTTDIGSRARLRQDTVPFLSGLRQ
yaldo0001_1400   EQAHKIIHDEYLAVQHTLNWYCFDYWSERLDLDIYAMTTDAGSRVRLRQDTAPFLAGLRE
yberc0001_1280   ASAHKIIHDEYLAVQHTLNWYCFDYWSERLDLDIYAMTTEAGSRVRLRQDTEPFLSSLRE
ymoll0001_810    ASAHKIIHDEYLAVQHTLNWYCFDYWSERLDLDIYAMTSEAGSRVRLRQDTEPFLSSLRE
yrohd0001_1810   DQAHKIIHDEYLAVQHTLNWYCFDYWSERLDLDIYAMTTQAGSRVQLRQDTKPFLAGLRE
yinte0001_1560   EQAHKIIHDEYLAVQHTLNWYCFDYWSERLDLDIYAMTTAAGNRVRLRQDTEPFLSGLRQ
yfred0001_1700   DQAHKIIHDEYLAVQHTLNWYCFDYWSERLDLDIYAMTTQAGSRVRLRQDTKPFLASLRE
yente0001X_2420  EQAHKIIHDEYLAVQHTLNWYCFDYWSERLDLDIYAMTTQAGNRVRLRQDTQPFLASLRE
ykris0001_1440   EQAHKIIHDEYLAVQHTLNWYCFDYWSERLDLDIYTMTTQAGSRVRLRQDTQPFLTSLRE
                 ############################################################


                        130       140       150       160       170       180
                 =========+=========+=========+=========+=========+=========+
yruck0001_1540   SGRKTILLTNAHPHSLHTKIAHTGLDQHLDLLLSTHTFGYPKEDQRLWQAVAQQTGFNPA
ypseu0001X_4163  HGLQTILLTNAHPHSLAVKIEHTALDQHLDLLLSTHTFGYPKEDQRLWQAVTQHTGLNPA
ypest0001X_3400  HGLQTILLTNAHPHSLAVKIEHTALDQHLDLLLSTHTFGYPKEDQRLWQAVTQHTGLNPA
yaldo0001_1400   RGVKTILLTNAHPHSLAVKVEHTALDQHLDLLLSTHTFGYPKEDQRLWQAVAQHTGFNPA
yberc0001_1280   RGLQTILLTNAHPHSLAVKIEHTALDQHLDLLLSTHTFGYPKEDQRLWQAVAQHTGLVPA
ymoll0001_810    RGLQTILLTNAHPHSLAVKIEHTALDQHLDLLLSTHTFGYPKEDQRLWLAVAQHTGLNPA
yrohd0001_1810   CGLQTILLTNAHPHSLAVKIEHTALDQHLDLLLSTHTFGYPKEDQRLWQAVTQHTGLNPA
yinte0001_1560   QGLQTILLTNAHPHSLAVKIEHTALDQHLDLLLSTHTFGYPKEDQRLWQAVAQHTGLNPA
yfred0001_1700   RGLQTILLTNAHPHSLAVKIEHTALDQHLDLLLSTHTFGYPKEDQRLWQAVTQHTGLNPA
yente0001X_2420  RGLQTILLTNAHPHSLAVKIEHTALDQHLDLLLSTHTFGYPKEDQRLWQAVTQHTGLNPA
ykris0001_1440   RGLQTILLTNAHPHSLAVKIEHTALDQHLDLLLSTHTFGYPKEDQRLWQAVTQHTGLNPA
                 ############################################################


                        190       200       210       220       230       240
                 =========+=========+=========+=========+=========+=========+
yruck0001_1540   RTLFVDDSEAILDAAKAFGIRYCLGVKNPDSSCAEKTFQSHPAIGDYRKLLPAIRHNGTG
ypseu0001X_4163  RTLFVDDSEAILDAAQTFGIRYCLGVENPDSSCADKTFHRHPAINDYRKLLPALQLRCE-
ypest0001X_3400  RTLFVDDSEAILDAAQTFGIRYCLGVENPDSSCADKTFHRHPAINDYRKLLPALQLRCE-
yaldo0001_1400   RTLFVDDSETILDAAHTFGIRYCLGIENPDSSCADKAFRNHPAINDYRKLLPALQLRCE-
yberc0001_1280   RTLFVDDSEAILDAAHTFGIRYCLGIENPDSSCADKIFHRHPAINDYRKLLPALKLRCE-
ymoll0001_810    RTLFVDDSETILDAARTFGIRYCLGIENPDSSCADKVFHSHPAINDYRKLLPALHLRSE-
yrohd0001_1810   RTLFVDDSETILDAAHTFGIRYCLGITNPDSSCAEKTFHNHPAINDYRKLLPALQLRSQ-
yinte0001_1560   RTLFVDDSETILDAARTFGIRYCLGIENPDSSCADKAFHSHPAINDYRKLLPALQLRGD-
yfred0001_1700   RTLFVDDSEKILDAARAFGIRYCLGIENPDSSCADKAFCGHPAINDYRKLLPALQLR---
yente0001X_2420  RTLFVDDSEKILDAARTFGIRYCLGIENPDSSCADKAFHSHPAINDYRKLLPALELRK--
ykris0001_1440   RTLFVDDSEKILDAAHTFGIRYCLGIENPDSSCADKAFHRHPAINDYRKLLPALQLRK--
                 #########################################################   


                 
                 ==
yruck0001_1540   AA
ypseu0001X_4163  --
ypest0001X_3400  --
yaldo0001_1400   --
yberc0001_1280   --
ymoll0001_810    --
yrohd0001_1810   --
yinte0001_1560   --
yfred0001_1700   --
yente0001X_2420  --
ykris0001_1440   --
```

```
Parameters used
Minimum Number Of Sequences For A Conserved Position: 6
Minimum Number Of Sequences For A Flanking Position: 9
Maximum Number Of Contiguous Nonconserved Positions: 8
Minimum Length Of A Block: 10
Allowed Gap Positions: With Half
Use Similarity Matrices: Yes
```

```
Flank positions of the 1 selected block(s)
Flanks: [14  237]  

New number of positions in PGL1_unique_yersinia-CLUSTERS.dir/PGL1_unique_yersinia-CL1012/PGL1_unique_yersinia-CL1012.muscle.fasta.gblo:  224  (92% of the original 242 positions)
```
