# Supplementary material for: Genomic characterization of the Yersinia genus
Source: Genome Biol. 2010 Jan 4;11(1):R1. doi: 10.1186/gb-2010-11-1-r1 (PMC2847712; doi:10.1186/gb-2010-11-1-r1)
Supplement: Additional file 15 — The top level directory consists of a directory called Additional_cluster_files and 5010 directories, one for each multi-protein cluster family. (This top level directory has been split into three data files for uploading purposes (Additional files 15, 16, 17).) Within the directory are the following files: PGL1_unique_Yersinia_unclustered.out - list of all protein singletons that MCL did not group into a cluster (see Materials and Methods); PGL1_Yersinia_unique_locus_tags.txt - names of the 11 locus tag prefixes used for each genome; PGL1_unique_Yersinia.gff - mapping each Yersinia protein to a cluster in tab delimited GFF; PGL1_unique_Yersinia.sigfile - list of the longest protein in each cluster; PGL1_unique_Yersinia.summary - summary table of features of each of the clusters; PGL1_unique_Yersinia.table - summary table of each protein in the clusters. Within each cluster directory are the following files, where 'x' is the cluster name: PGL1_unique_Yersinia-x.faa - multifasta file of the proteins in the cluster; PGL1_unique_Yersinia-x.summary - summary of the properties of the proteins; PGL1_unique_Yersinia-x.matches - blast matches between the proteins of the cluster; PGL1_unique_Yersinia-x.muscle.fasta - muscle alignment of the proteins; PGL1_unique_Yersinia-x.muscle.fasta.gblo - gblocks output of muscle alignment (that is, auto-trimmed alignment); PGL1_unique_Yersinia-x.muscle.fasta.gblo.htm - as above in html format; PGL1_unique_Yersinia-x.muscle.tree - treefile from muscle alignment; PGL1_unique_Yersinia-x.sif - matches between proteins in simple interaction format for display on graphing software. [file gb-2010-11-1-r1-S15.zip › clusters/PGL1_unique_yersinia-CL1013/PGL1_unique_yersinia-CL1013.muscle.fasta.gblo.htm]

PGL1\_unique\_yersinia-CL1013.muscle.fasta


## Gblocks 0.91b Results

Processed file: **PGL1\_unique\_yersinia-CL1013.muscle.fasta**  
Number of sequences: **11**  
Alignment assumed to be: **Protein**  
New number of positions: **708** (selected positions are underlined in blue)

```
                         10        20        30        40        50        60
                 =========+=========+=========+=========+=========+=========+
yruck0001_1550   -----------------LALLFTSLVAVALLWWFKCR--SASSVPLSFIKPTHRTLAPEE
ypseu0001X_4162  -----------------LALLLTSLIAVGLLWWLRFRRPHPITAALPFVKPTHRKLTPEE
ypest0001X_3410  -----------------LALLLTSLIAVGLLWWLRFRRPHPITAALPFVKLTHRKLTPEE
yaldo0001_1410   -------------MVLILALLLTSMIVVGLWWWFRFRHLSLATVTMPFVKPTCRKLTPEE
yberc0001_1290   ----------MSTIVLILALLLTSLIAVGLLWWFKFRHSPRIAATLPFVKPTHRLLTPEE
ymoll0001_820    -----------------LALLLTSLIAVGLLWWFKFRPSPRVAATLPFAKPTHRTLTPEE
yrohd0001_1820   --------------VLILALLLTGLIAAALLWWFRSRRTAPVTATLLFAKPIHRKLTQEE
yinte0001_1570   -------------MVLILALLLTSLIAVGLLWWFRFRRPAPVTATLPFAKPIYRKLTPEE
yfred0001_1710   VSQGRSDGDGMSTIVLILALLLTSLIAVG-LWWFRFRRTAPVTATLLFAKPTHRKLTPEE
ykris0001_1450   -----------------LALLLTSLIAVGLLWWFRFRRPAPVTATLPFAKPTHRKLTPEE
yente0001X_2430  ---------------------------VGLLWWFRFRRPIPVTATLPFAKPTHRKLTPEE
                                  ###########################################


                         70        80        90       100       110       120
                 =========+=========+=========+=========+=========+=========+
yruck0001_1550   RVAVENYLLNLPGSSTSNTLPSFDPKALQGHTNASTELALTPDSDNVYAVTRAITRYGVA
ypseu0001X_4162  RVSIENYLRNQQNKHGFNTQPAFDSHALAASTSSTPMLVLTPQSDNVYSVTRAITRYGVA
ypest0001X_3410  RVSIENYLRNQQNKHGFNTQPAFDSHALAASTSSTPMLVLTPQSDNVYSVTRAITRYGVA
yaldo0001_1410   RVNIENYLLNQQEKVGFKSQPTFDSRTLTNDNISSGKLVLTPQSDNVYSVTRAITRYGVA
yberc0001_1290   RVNIENYLLGQHENSGLKNLSAFDPNTLINRDLSPEKLVLTPQSEKVYSVTRAITRYGVA
ymoll0001_820    RVNIENYLLSQHENSGFKALSTFDPNTLTNRDFAPEKLVLTPQSEKVYSVTRAITRYGVA
yrohd0001_1820   RVNIENYLLNQQDKLGFKPQSTFDSRELVN----SGKLSLTSQSDDVYSVTRAITRYGVA
yinte0001_1570   RVNIENYLLGQREKLGFKAQSTFDTRTLTNNNLVPAKLALTPQSENVYSVTRAITRYGVA
yfred0001_1710   RVNIENYLLSQQDKLGFKPQSNFDTRTLTDNSATPAKLILTPQSDNVYSVTRAITRYGVA
ykris0001_1450   RVNIENYLLNQQDNIGFKPQSTFDTRSLSNSTPSPAKLILTPQSDNVYSVTRAITRYGVA
yente0001X_2430  RVNIENYLLNQQDKIGFKPQSTFDTRTLTNSALSPAKLILIPQSDNVYAVTRAITRYGVA
                 ############################################################


                        130       140       150       160       170       180
                 =========+=========+=========+=========+=========+=========+
yruck0001_1550   SDEPNKWRYYLDSVEIHLPPFWKPFIAQDNFVEIIQTNTVPLVISLNGHSLKDYTLEIPF
ypseu0001X_4162  SDEPNKWRYYLDSIEVHLPSAWEQYITQDNDVELIQTQTIPLVISLNGHTLNNHQSENTY
ypest0001X_3410  SDEPNKWRYYLDSIEVHLPSAWEQYITQDNDVELIQTQTIPLVISLNGHTLNNHQSENTY
yaldo0001_1410   SDEPNEWRYYLDSIEVHLPPSWEQYITQDNDVELIQTQTIPLVISLNGHTLKNHQPENSY
yberc0001_1290   SDEPNKWRYYLDSIEVHLPSSWEQYITQDNDVELIQTQSIPLVISLNGHTLKNHQPEISY
ymoll0001_820    SDEPNKWRYYLDSIEVHLPSSWEQYITQDNDVELIQTQSIPLVISLNGHTLKNHQPENTY
yrohd0001_1820   SDEPNKWRYYLDSIEIHLPASWEQYIAQDNDVELIQTQTIPLVISLNGHTLKNHQSEHTY
yinte0001_1570   SDEPNKWRYYLDSIEIHLPSSWEQYIAQDNDVELIQTQTIPLVISLNGHTLKNHQAENSY
yfred0001_1710   SDEPNKWRYYLDSIEVHLPASWEQYITQDNDVELIQTQTLPLVISLNGHTLKNHQSENIY
ykris0001_1450   TDEPNNWRYYLDSIEVHLPASWEQYITQNNDVELIQTQPIPLIISLNGHTLKNHQSENSY
yente0001X_2430  TDEPNKWRYYLDSIEVHLPASWEQYITQDNDVELIQTQTIPLVISLNGHTLKNHQSENTY
                 ############################################################


                        190       200       210       220       230       240
                 =========+=========+=========+=========+=========+=========+
yruck0001_1550   PQVAPSSLQNASIRKEDSEHIELVNIRKETPEEHALYGSNGLTQAATICVALLLLFLSLI
ypseu0001X_4162  QPILPSVSKNASIRKEDSEHIELLNIRKETPEEYALHGPNGLKEACAICIALLLLFFALS
ypest0001X_3410  QPILPSVSKNASIRKEDSEHIELLNIRKETPEEYALHGPNGLKEACAICIALLLLFFALS
yaldo0001_1410   QPLLPSAVQNASIRKKDSEHIELLNIRKETAEEYALHGPNGLKEALVICVALLLLFLALT
yberc0001_1290   QPISPSSAQNASIRKADSEHIELLNIRKETAEEYALHSANDLKEAVAICLALLLLFFALI
ymoll0001_820    QPILPSTAQNASIRKADSEHIELLNIRKETAEEYALHGANDLKEAIAICLALLILFFALI
yrohd0001_1820   PPILASVAQNASIRKADSEHIELLNIRKETPEEYALHGSNGLKEAGAICVALLLLFFALT
yinte0001_1570   QPILPSTAQNASIRKADSEHIELLNIRKETPEEYALHGANGLKEAAAICLALLLLFFALI
yfred0001_1710   QPILPSVTQNASIRKADSEHIELLNIRKETPEEYALHGPNGLKEAAAICLALLLLFFALT
ykris0001_1450   LPILPSVAQNASIRKADSEHIELLNIRKETAEEYALHGANGLKEAGAICLALLLLFFALT
yente0001X_2430  QPILSSVTQNASIRKADSEHIELLNIRKETPEEYALHGTNGLKEAGAICLALLLLFFALT
                 ############################################################


                        250       260       270       280       290       300
                 =========+=========+=========+=========+=========+=========+
yruck0001_1550   SPAVVLPWLILVAIALISWACWHLFRPLSANDLKEVHCLSGTPKRWGLFGESNQSQMNNI
ypseu0001X_4162  GPTVTLPWLVIVAVSLTCWACWNLFRPLSEKDLREVHCLNGTPKRWGLFGESNQGQINNI
ypest0001X_3410  GPTVTLPWLVIVAVSLTCWACWYLFRPLSEKDLREVHCLNGTPKRWGLFGESNQGQINNI
yaldo0001_1410   GPAVTMLWLVIVAATLTGWACWNMLRPLADKDLREVHCLRGTPKRWGLFGESNQAQMNNI
yberc0001_1290   GPAVTLPWLVIVAATLTGWACWNMFRPLSEKDLREVHCLSGTPKRWGLFGESNQGQMSNI
ymoll0001_820    GPAVTLPWLVIVAATLIGWACWNIFRPLSEKDLREVHCLSGTPKRWGLFGESNQGQMSNI
yrohd0001_1820   GPAVTLPWLVIVAVALTGWACWNMFRPLSEKDLREVHCLSGTPKRWGLFGESNQGQMSNI
yinte0001_1570   GPAVILPWLIIVAITLTGWACWNMFRPLSEKDLREVHCLSGTPKRWGLFGESNQGQMNNI
yfred0001_1710   GPTVTLPWLIIVAATLTGWACWNMFRPLSEKDLREVHCLSGTPKRWGLFGESNQSQMNNI
ykris0001_1450   GPTVILPWLIIVATLLTGWACWNMFRPLSEKDLREVHCLSGTPKRWGLFGESNQGQMNNI
yente0001X_2430  GPAVTLPWLIIVAATLTGWACWNMFRPLSEKDLREVHCLSGTPKRWGLFGESNQGQMNNI
                 ############################################################


                        310       320       330       340       350       360
                 =========+=========+=========+=========+=========+=========+
yruck0001_1550   SLGIIDLIYPAHWLPYFTHDLGKKTNVDIYLNRQVIRQGRYLSLHDEMKNFPLQRWGKNL
ypseu0001X_4162  SLGIVDLIYPAHWGPYFVHDLGKKTHIDIYLNRQVVRQGAFLSLHDEMKMFPLQRWGKNL
ypest0001X_3410  SLGIVDLIYPAHWGPYFVHDLGKKTHIDIYLNRQVVRQGAFLSLHDEMKMFPLQRWGKNL
yaldo0001_1410   SLGIVDLIYPAHWGPYFAQDLGKKTHIDIYLNRQVVRQGAFLSLHDEMKHFPLQRWGKNL
yberc0001_1290   SLGVVDLIYPAHWGPYFAHDLGKKTNIDIYLNRQVIRQGRFLSLHDEMKHFPLQRWGKNL
ymoll0001_820    SLGVVDLIYPAHWGPYFAHDLGKKTNIDIYLNRQVIRQGRFLSLHDEMKHFPLQRWGKNL
yrohd0001_1820   SLGIVDLIYPAHWGPYFAHDLGKKTNIDIYLNRQVVRQGTFLSLHDEMKNFPLQRWGKNL
yinte0001_1570   SLGIVDLIYPAHWGPYFAHDLGKKTNIDIYLNRQVVRQGPFLSLHDEMKHFPLQRWGKNL
yfred0001_1710   SLGIVDLIYPAHWGPYFTHDLGKKTNIDIYLNRQVVRQGPFLSLHDEMKHFPLQRWGKNL
ykris0001_1450   SLGIVDLIYPAHWGPYFAHDLGKKTNIDIYLNRQVVRQGPFLSLHDEMKHFPLQRWGKNL
yente0001X_2430  SLGIVDLIYPAHWGPYFAHDLGKKTNIDIYLNRQVVRQGPFLSLHDEMKHFPLQRWGKNL
                 ############################################################


                        370       380       390       400       410       420
                 =========+=========+=========+=========+=========+=========+
yruck0001_1550   TLMLGSLAILILLLTYIPLSLPLKLSMAWIQGAQTQQVTSVLALEKMPLRIGDMLKARGT
ypseu0001X_4162  TLIVGSLLVLVLLLIYVPLGLPLKLSVAWLQGAQSQQVTSVAALDKMPLRIGDMLKAQGN
ypest0001X_3410  TLIVGSLLVLVLLLIYVPLGLPLKLSVAWLQGAQSQQVTSVAALDKMPLRIGDMLKAQGN
yaldo0001_1410   TLVAGSLLVLVLLLIYVPLGLPLKLSVAWLQGAQSQQVTSVEALEKMPLRIGDMLKAQGN
yberc0001_1290   TLIAGSLLVMTLLLIYVPLSLPLKLSVAWLQGAQSQQVTSIEALEKMPLRIGDMLKAQGT
ymoll0001_820    TLMAGSLLVMALLLIYVPLGLPLKLSVAWLQGAQSQQVTSVEALEKMPLRIGDMLKAQGT
yrohd0001_1820   TLMLGSLLVLALLLIYVPLGLPLKLSVAWIQGAQSQQVTSVDALEKMPLRIGDMLKAQGT
yinte0001_1570   TLIAGSLLIMVLLLIYVPLGLPLKLSIAWLQGAQSQQVTSVEALEKMPLHIGDMLKAQGT
yfred0001_1710   TLIAGSLLVMALLLIYVPLGLPLKLSVAWLQGAQSQQVTSVTALEKMPLRIGDMLKAQGT
ykris0001_1450   TLMAGSLLVMGLLLIYIPLGLPLKLSVAWLQGAQSQQVTSVEALEKMPLHIGDMLKAQGM
yente0001X_2430  TLMTGSLLVMALLLIYVPLGLPLKLSVAWLQGAQSQQVTSVEALEKMPLRIGDMLKAQGM
                 ############################################################


                        430       440       450       460       470       480
                 =========+=========+=========+=========+=========+=========+
yruck0001_1550   GMCYVPPSTMSQNNLAFTPFDCSSIYWNTASPLPQPESEIIEKAAALVETVHQQLHPQES
ypseu0001X_4162  GMCYVPPNIQNTRGFVFTPFDCSGIYWNTASPLPQPESETIEKAAALVETINKQLHPQGS
ypest0001X_3410  GMCYVPPNIQNTRGFVFTPFDCSGIYWNTASPLPQPESETIEKAAALVETINKQLHPQGS
yaldo0001_1410   GMCYVPPNSQNSRNFVFTPFDCSGIYWNNAEPLPQPESDTIEKAAALVASVNHQLHPQGT
yberc0001_1290   GMCYVPPNTQNPHSFVFTPFDCSGIYWNSAAPLPLPESDTIEKAASLMATVNQQLHPQGS
ymoll0001_820    GMCYVPPNTQNPHSFVFTPFDCSGIYWNNAAPLPLPESDTIERAASLMASVTQQLHPQGS
yrohd0001_1820   GMCYVPPNTQTPHSFVFTPFDCSGIYWNNAAPLPQPESDIIEKAAALVSTINQQLHPQGA
yinte0001_1570   GMCYVPPNTQNPHSFVFTPFDCSGIYWNNAAPLPQPESDTIEKAAALVATVNQQLHPQGI
yfred0001_1710   GMCYVPPNSQNPNSFAFTPFDCSGIYWNNAAPLPQPESETIEKAAALIATVNQQLHPQGT
ykris0001_1450   GMCYVPPNTQNPHSFVFTPFDCSGIYWNNAAPLPQPESETIEKAAALVATVNQQLHPQGT
yente0001X_2430  GMCYVPPNTQNSHSFVFTPFDCSGIYWNNAAPLPQPESEIIEKAASLVATVNQQLHPQGS
                 ############################################################


                        490       500       510       520       530       540
                 =========+=========+=========+=========+=========+=========+
yruck0001_1550   NDANVNPKLATAIEKSGMTLLDDFADIVLKTNELCRNESDCGRLKNALVNLGNAKNWAAL
ypseu0001X_4162  -DASVNPKLATAIEKSGMILLDDFSDIVLKTQALCSENTDCIRLKNALVNLGNAKNWSAL
ypest0001X_3410  -DASVNPKLATAIEKSGMILLDDFSDIVLKTQALCSENTDCIRLKNALVNLGNAKNWSAL
yaldo0001_1410   -DASVNPQLATAIEKSGMILLDNFADIVLKTQTLCSNDADCIRLKNALVNLGNARNWPGL
yberc0001_1290   -DANVNPQLATAIEKSGMILLNDFADIVLKTQALCGADSDCIRLKNALVNLGNAKNWPGL
ymoll0001_820    -DANVNPQLATAIEKSGMILLDNFADIVLKTQALCGAESDCIRLKNALVNLGNAKNWSGL
yrohd0001_1820   -DASINPQLATAIEKSGMILLDNFADIVLKTQALCGGDTDCIRLKNALVNLGNAKNWQGL
yinte0001_1570   -DVNVNPQLATAIEKSGMILLDNFADIVLKTQALCGGDNDCVRLKNALVNLGNAKNWSGL
yfred0001_1710   -DTSVNPQLATAIEKSGMILLDNFADIVLKTQQLCGGDSDCVRLKNALVNLGNAKSWSGL
ykris0001_1450   -DTNVNPQLATAIEKSGMILLDNFADIVLKTQALCGGDADCIRLKNALVNLGNAKNWPGL
yente0001X_2430  -DANVNPQLATAIEKSGMILLDNFADIVLKTQALCGGDADCIRLKNALVNLGNAKNWSGL
                  ###########################################################


                        550       560       570       580       590       600
                 =========+=========+=========+=========+=========+=========+
yruck0001_1550   TKRAQSGALQGMNVLLRPVSADALERLVKTATSSFVYRETHLATEALNSPPPGGFLISSD
ypseu0001X_4162  VKRAQSGNLEGMNVLLRPISADVLENLINTAASSFVYRETHLATEALNSPPPGGFLITSD
ypest0001X_3410  VKRAQSGNLEGMNVLLRPISADVLENLINTAASSFVYRETHLATEALNSPPPGGFLITSD
yaldo0001_1410   VKRAQSGALKGMNVLLRPVSADALENLVNTATSSFVYRETHLATEALNSPPPGGFLITSD
yberc0001_1290   VKRAQSGTLKGMNVLLRPVSADTLENLVKTATSSFVYRETHLATEALNSPPPGGFLITSD
ymoll0001_820    VKRAQSGTLKGMNVLLRPVSADTLESLVKTATSSFVYRETHLATEALNSPPPGGFLITSD
yrohd0001_1820   VKRAQSGALKGMNVLLRPVSADTLESLVKNATSSFVYRETHLATEALNSPPPGGFLITSD
yinte0001_1570   VKRAQSGTLKGMNVLLRPVSADALESLVKTATSSFVYRETHLATEALNSPPPGGFLITSD
yfred0001_1710   VKRAQSGTLKGMNVLLRPVSADTLENLVKTATSSFVYRETHLATEALNSPPPGGFLITSD
ykris0001_1450   VKRAQSGTLKGMNVLLRPVSADTLENLVKTATSSFVYRETHLATEALNSPPPGGFLITSD
yente0001X_2430  VKRAQSGTLKGMNVLLRPVSADTLENLVKTATSSFVYRETHLATEALNSPPPGGFLITSD
                 ############################################################


                        610       620       630       640       650       660
                 =========+=========+=========+=========+=========+=========+
yruck0001_1550   EGKQLVSHPIPSIPLFDYTALEQWRELQRLSGLLLNTPFKAEGVITSITVDANGTRHITL
ypseu0001X_4162  EGKQLVNHPAPTLPLFDYSALEQWRELQRLSALLLDTPFKAEGIITNITTDANGTRHIAL
ypest0001X_3410  EGKQLVNHPAPTLPLFDYSALEQWRELQRLSALLLDTPFKAEGIITNITTDANGTRHIAL
yaldo0001_1410   EGKQLVNHPAPSVPLFDYSALEQWRELQRLSGLLLNTPFKAEGIITNITVDANGTRHIAL
yberc0001_1290   EGKQLVNHPAPAVPLFDYSALEQWRELQRLSGLLLNTPFKAEGIITNITVDANGTRHIAL
ymoll0001_820    EGKQLVNHPAPAVPLFDYSALEQWRELQRLSGLLLNTPFKAEGIITNITIDANGTRHIAL
yrohd0001_1820   EGKQLVSHPVPAVPMFDYSALEQWRELQRLSGLLLNTPFKAEGIITNITTDANGTRHIAL
yinte0001_1570   EGKQLVNHPAPAVPLFDYSALEQWRELQRLSGLLLNTPFKAEGIITNITIDANGTRHIAL
yfred0001_1710   EGKQLVSHPVPAVPLFDYSALEQWRELQRLSGLLLNTPFKAEGIITNITTDANGTRHIAL
ykris0001_1450   EGKQLVNHPAPSVPLFDYSALEQWRELQRLSGLLLNTPFKAEGIITNITTDANGTRHIAL
yente0001X_2430  EGKQLVNHPAPSVPLFDYSALEQWRELQRLSGLLLNTPFKAEGIITNITTDANGTRHIAL
                 ############################################################


                        670       680       690       700       710       720
                 =========+=========+=========+=========+=========+=========+
yruck0001_1550   HSEPDVITLSRYLGTTLLLFALMACLAINGYLVLVRMRKNRTRLVDIQRYYDHCFDTPLS
ypseu0001X_4162  HSEPDIVTLGRYLATSLLLLVLIFCLVVNMVLLIQRAMKNRRRMDNIQRYYDDCFNQTLT
ypest0001X_3410  HSEPDIVTLGRYLATSLLLLVLIFCLVVNMVLLIQRAMKNRRRMDNIQRYYDDCFNQTLT
yaldo0001_1410   HSEPDIVTLGRYLGTSLLLLALVVCLLVNATLLIMRVLKNRSRMDDIQHYYDDCFNHNLT
yberc0001_1290   HSEPDIMTLSRYLGTCLLLLALIICLVVNTTLLIQRILQNRSRMDNIQRYYDNCFNQTLT
ymoll0001_820    HSEPDMMTLSRYLGTSLLLLTLIICLVVNTTLLIQRVLQNRSRMDNIQRYYDNCFNQTLT
yrohd0001_1820   HSEPDIITLGRYLGTSSLLLTLIVCLVVNATLLIQRILKNRSRMDNIQRYYDNCFNQPLA
yinte0001_1570   HSEPDIVTLGRYLGTSLLLLVLIACLVVNTSLLIQRILKNRSRMDNIQRYYDNCFNQTLT
yfred0001_1710   HSEPDIVTLGRYLGTSLLLLVLIVCLVVNTTLLVQRILKNRSRMDNIQRYYDNCFNQPLP
ykris0001_1450   HSEPDIVTLGRYLGTSLLLLTLIVCFAVNATLFIQRILKNRSRMDNIQRYYDNCFNQPLT
yente0001X_2430  HSEPDIVTLGRYLGTSLLLLVLIVCLVVNTTLFIRRVLKNRSRMDNIQRYYDNCFNQPLT
                 ############################################################


                 
                 ======
yruck0001_1550   PPSYLR
ypseu0001X_4162  PPPFLR
ypest0001X_3410  PPPFLR
yaldo0001_1410   PSPRLR
yberc0001_1290   PTALLR
ymoll0001_820    PAPFLR
yrohd0001_1820   PTSFLR
yinte0001_1570   PTPFLR
yfred0001_1710   PAPFLR
ykris0001_1450   PAPFPR
yente0001X_2430  PAPFLR
                 ######
```

```
Parameters used
Minimum Number Of Sequences For A Conserved Position: 6
Minimum Number Of Sequences For A Flanking Position: 9
Maximum Number Of Contiguous Nonconserved Positions: 8
Minimum Length Of A Block: 10
Allowed Gap Positions: With Half
Use Similarity Matrices: Yes
```

```
Flank positions of the 2 selected block(s)
Flanks: [18  480]  [482  726]  

New number of positions in PGL1_unique_yersinia-CLUSTERS.dir/PGL1_unique_yersinia-CL1013/PGL1_unique_yersinia-CL1013.muscle.fasta.gblo:  708  (97% of the original 726 positions)
```
