# Supplementary material for: Genomic characterization of the Yersinia genus
Source: Genome Biol. 2010 Jan 4;11(1):R1. doi: 10.1186/gb-2010-11-1-r1 (PMC2847712; doi:10.1186/gb-2010-11-1-r1)
Supplement: Additional file 15 — The top level directory consists of a directory called Additional_cluster_files and 5010 directories, one for each multi-protein cluster family. (This top level directory has been split into three data files for uploading purposes (Additional files 15, 16, 17).) Within the directory are the following files: PGL1_unique_Yersinia_unclustered.out - list of all protein singletons that MCL did not group into a cluster (see Materials and Methods); PGL1_Yersinia_unique_locus_tags.txt - names of the 11 locus tag prefixes used for each genome; PGL1_unique_Yersinia.gff - mapping each Yersinia protein to a cluster in tab delimited GFF; PGL1_unique_Yersinia.sigfile - list of the longest protein in each cluster; PGL1_unique_Yersinia.summary - summary table of features of each of the clusters; PGL1_unique_Yersinia.table - summary table of each protein in the clusters. Within each cluster directory are the following files, where 'x' is the cluster name: PGL1_unique_Yersinia-x.faa - multifasta file of the proteins in the cluster; PGL1_unique_Yersinia-x.summary - summary of the properties of the proteins; PGL1_unique_Yersinia-x.matches - blast matches between the proteins of the cluster; PGL1_unique_Yersinia-x.muscle.fasta - muscle alignment of the proteins; PGL1_unique_Yersinia-x.muscle.fasta.gblo - gblocks output of muscle alignment (that is, auto-trimmed alignment); PGL1_unique_Yersinia-x.muscle.fasta.gblo.htm - as above in html format; PGL1_unique_Yersinia-x.muscle.tree - treefile from muscle alignment; PGL1_unique_Yersinia-x.sif - matches between proteins in simple interaction format for display on graphing software. [file gb-2010-11-1-r1-S15.zip › clusters/PGL1_unique_yersinia-CL1014/PGL1_unique_yersinia-CL1014.muscle.fasta.gblo.htm]

PGL1\_unique\_yersinia-CL1014.muscle.fasta


## Gblocks 0.91b Results

Processed file: **PGL1\_unique\_yersinia-CL1014.muscle.fasta**  
Number of sequences: **11**  
Alignment assumed to be: **Protein**  
New number of positions: **206** (selected positions are underlined in blue)

```
                         10        20        30        40        50        60
                 =========+=========+=========+=========+=========+=========+
yruck0001_19220  ----LNLEQNQANTGRQPYLSTDQDNTVINGADESPSDGKRIIFFDLDGTLHQQDMFGSF
ypseu0001X_3231  ------------------------------VVEPQSAGAKRIIFFDLDGTLHQQDMFGSF
ypest0001X_3204  ------------------------------VVEPQSAGAKRIIFFDLDGTLHQQDMFGSF
yaldo0001_24270  ----LSVEQ----------------------AKPQSERAKRIVFFDLDGTLHQQDMFGSF
yinte0001_23260  ----LSAEQATSTFKHA-------GNKCEGTAEAPSTSAKRIVFFDLDGTLHQQDMFGSF
yberc0001_23820  ----LSGKQASSTFEQT-------DLHPKTTREPLAVSGKRVVFFDLDGTLHQQDMFGSF
yrohd0001_20680  ----LGVEQAAPAFEK--------------TEKPHSASTKRVVFFDLDGTLHQQDMFGSF
ymoll0001_22660  ----LSAEQATSPFEQA-------DTDSKAAREPQVASAKRVVFFDLDGTLHQQDMFGSF
ykris0001_24110  MTNVFSAEQNDT------------------MAEPPAENVKRVVFFDLDGTLHQQDMFGSF
yente0001X_3297  MTKVSNAESVST------------------TAEPPSASVKRVVFFDLDGTLHQQDMFGSF
yfred0001_25230  -----------------------------------------VVFFDLDGTLHQEDMFGSF
                                                        #####################


                         70        80        90       100       110       120
                 =========+=========+=========+=========+=========+=========+
yruck0001_19220  LRFLIRHLPLNLLLVLPLLPIVGFGLLVNGRCARWPISLLLWGMTFGRRESDLNDLERRF
ypseu0001X_3231  LRFLLRHLPLNLLLVIPLLPLIGVGLLVGGRCARWPMSLLLWAMTFGRREAHLRDLEWRF
ypest0001X_3204  LRFLLRHLPLNLLLVIPLLPLIGVGLLVGGRCARWPMSLLLWAMTFGRREAHLRDLEWRF
yaldo0001_24270  LRFLLRHLPLNLLLVIPVLPVIGLGLLVGGRCTRWPMSLLLWAITFGRREAHLKDLELRF
yinte0001_23260  LRFLLRHLPLNLLLVVPLLPVIGLGLLAGGRCARWPMSLLLWAITFGRREAHLNDLELRF
yberc0001_23820  LRFLLRHLPLNLLLVIPLLPVIGLGLLVGGRCARWPMSLLLWATTFGRREAKLKDLELRF
yrohd0001_20680  VRFLLRHLPLNLLLVIPLLPIIGLGFVIKGRCARWPMSLLLWAITFGRSEAHLKGLELRF
ymoll0001_22660  LRFLLRHLPLNLLLVIPLLPVIGLGLLVGGRCARWPMSLLLWAATFGRREAHLKDLELRF
ykris0001_24110  LRFLLRHLPLNILLVIPLLPVIGLGLLVGGRCARWPISLLLWALTFGRPEAQLKDLELRF
yente0001X_3297  LRFLLRHLPLNLLLVIPLLPVIGLGLLVGGRCARWPMSLLLWAITFGRREAHLRDLELRF
yfred0001_25230  LRFLLRHLPLNLLLVIPLLPVIGLGLLVGGRCARWPISLLLWAITFGRSEAHLKDLELRF
                 ############################################################


                        130       140       150       160       170       180
                 =========+=========+=========+=========+=========+=========+
yruck0001_19220  VKIFRQKVVGFPVVKMRLRQYLEQENTQVWLITGSPQRLVEQVYHDSPYRHRLHLVGSQM
ypseu0001X_3231  VAAFRQKVTEFPVVAMRLRQYLESSDAEVWLITGSPQRLVEQVYHDSNFIHRLRLIGSRM
ypest0001X_3204  VAAFRQKVTEFPVVAMRLRQYLESSDAEVWLITGSPQRLVEQVYHDSNFIHRLRLIGSRM
yaldo0001_24270  VNEFRQQVTAFPVVMMRLRQYLASDEAEVWLITGSPQRLVEQVYHDSTFIHHLRLVGSRM
yinte0001_23260  VNDFRQKVTEFPVVVMRLRQYLESHDVQVWLITGSPQRLVEQVYHDSTFIHRLRLVGSRM
yberc0001_23820  VQAFRQKVTAFPVVVMRLREYLESSDAEVWLITGSPQRLVEQVYHDSTFIHRLRLVGSRM
yrohd0001_20680  VNEFRQKVTEFPVVMMRLRQYLVSNDAEVWLITGSPQRLVEQVYHDSEFIHHLRLVGSRM
ymoll0001_22660  VKAFRQKVTAFPVVVMRLREYLESSDAEVWLITGSPQRLVEQVYHDSAFIHRLRLVGSRM
ykris0001_24110  VKEFRQKVTEFPVVMMRLRQYLQSTDAQVWLITGSPQRLVEQVYHDSDFIHHLRLVGSRM
yente0001X_3297  VNEFRQKVTEFPVVMMRLRQYLKSTDAQVWLITGSPQRLVEQVYHDSDFIHHLRLVGSRM
yfred0001_25230  VNDFRQKVTEFPVVMMRLRQYLDSTDAQVWLITGSPQRLVEQVYHDSEFIHHLRLVGSRM
                 ############################################################


                        190       200       210       220       230       240
                 =========+=========+=========+=========+=========+=========+
yruck0001_19220  ARRNGGWILPLRCLGAEKVVQLEQRLGSPLKLYSGYSDSRQDNPLLFFCEHRWRVSKDGE
ypseu0001X_3231  ERRNGGWVLPLRCLGPEKVVQLEQRLGSPLKLYSGYSDSKQDNPLLAFCEHRWRVSKTGE
ypest0001X_3204  ERRNGGWVLPLRCLGPEKVVQLEQRLGSPLKLYSGYSDSKQDNPLLAFCEHRWRVSKTGE
yaldo0001_24270  ARRNGGWVLPLRCLGPEKVVQLEQRLGAPLKLYSGYSDSKQDNPLLYFCEHRWRVSKNGE
yinte0001_23260  ARRHGGWVLPLRCLGPEKVVQLEQRLGSPLKLYSGYSDSKQDNPLLYFCEHRWRVSKSGE
yberc0001_23820  ARRNGGWVLPLRCLGPEKVVQLEQRLGSPLKLYSGYSDSKQDNPLLHFCEHRWRVSKTGE
yrohd0001_20680  ARRNGGWVLPLRCLGPEKVVQLEQRLGSPLQLYSGYSDSKQDNPLLHFCEHRWRVSKTGE
ymoll0001_22660  ARRNGGWVLPLRCLGPEKVVQLEQRLGSPLKLYSGYSDSKQDNPLLHFCEHRWRVSKTGE
ykris0001_24110  ARRNGGWVLPLRCLGPEKVVQLEQRLGSPLKLYSGYSDSKQDNPLLHFCEHRWRVSKTGE
yente0001X_3297  ARRNGGWVLPLRCLGAEKVLQLEQRLGSPLKLYSGYSDSKQDNPLLHFCEHRWRVSKTGE
yfred0001_25230  ARRNGGWVLPLRCLGPEKVVQLEQRLGSPLKLYSGYSDSKQDNPLLKFCEHRWRVSKTGE
                 ############################################################


                 
                 =====
yruck0001_19220  LQQLE
ypseu0001X_3231  LQQLE
ypest0001X_3204  LQQLE
yaldo0001_24270  LQQLE
yinte0001_23260  LQQLE
yberc0001_23820  LQQLE
yrohd0001_20680  LQQLE
ymoll0001_22660  LQQLE
ykris0001_24110  LQQLE
yente0001X_3297  LQQLE
yfred0001_25230  LQQLE
                 #####
```

```
Parameters used
Minimum Number Of Sequences For A Conserved Position: 6
Minimum Number Of Sequences For A Flanking Position: 9
Maximum Number Of Contiguous Nonconserved Positions: 8
Minimum Length Of A Block: 10
Allowed Gap Positions: With Half
Use Similarity Matrices: Yes
```

```
Flank positions of the 1 selected block(s)
Flanks: [40  245]  

New number of positions in PGL1_unique_yersinia-CLUSTERS.dir/PGL1_unique_yersinia-CL1014/PGL1_unique_yersinia-CL1014.muscle.fasta.gblo:  206  (84% of the original 245 positions)
```
