# Supplementary material for: Genomic characterization of the Yersinia genus
Source: Genome Biol. 2010 Jan 4;11(1):R1. doi: 10.1186/gb-2010-11-1-r1 (PMC2847712; doi:10.1186/gb-2010-11-1-r1)
Supplement: Additional file 15 — The top level directory consists of a directory called Additional_cluster_files and 5010 directories, one for each multi-protein cluster family. (This top level directory has been split into three data files for uploading purposes (Additional files 15, 16, 17).) Within the directory are the following files: PGL1_unique_Yersinia_unclustered.out - list of all protein singletons that MCL did not group into a cluster (see Materials and Methods); PGL1_Yersinia_unique_locus_tags.txt - names of the 11 locus tag prefixes used for each genome; PGL1_unique_Yersinia.gff - mapping each Yersinia protein to a cluster in tab delimited GFF; PGL1_unique_Yersinia.sigfile - list of the longest protein in each cluster; PGL1_unique_Yersinia.summary - summary table of features of each of the clusters; PGL1_unique_Yersinia.table - summary table of each protein in the clusters. Within each cluster directory are the following files, where 'x' is the cluster name: PGL1_unique_Yersinia-x.faa - multifasta file of the proteins in the cluster; PGL1_unique_Yersinia-x.summary - summary of the properties of the proteins; PGL1_unique_Yersinia-x.matches - blast matches between the proteins of the cluster; PGL1_unique_Yersinia-x.muscle.fasta - muscle alignment of the proteins; PGL1_unique_Yersinia-x.muscle.fasta.gblo - gblocks output of muscle alignment (that is, auto-trimmed alignment); PGL1_unique_Yersinia-x.muscle.fasta.gblo.htm - as above in html format; PGL1_unique_Yersinia-x.muscle.tree - treefile from muscle alignment; PGL1_unique_Yersinia-x.sif - matches between proteins in simple interaction format for display on graphing software. [file gb-2010-11-1-r1-S15.zip › clusters/PGL1_unique_yersinia-CL1015/PGL1_unique_yersinia-CL1015.muscle.fasta.gblo.htm]

PGL1\_unique\_yersinia-CL1015.muscle.fasta


## Gblocks 0.91b Results

Processed file: **PGL1\_unique\_yersinia-CL1015.muscle.fasta**  
Number of sequences: **11**  
Alignment assumed to be: **Protein**  
New number of positions: **174** (selected positions are underlined in blue)

```
                         10        20        30        40        50        60
                 =========+=========+=========+=========+=========+=========+
yaldo0001_1450   MYQVNFIPWRAERQRARFRFWRNTGLCHLGLVVIGFVVMSMQLRGERILLQGNLVVLSQQ
ypest0001X_3450  MYQVNFLPWRIARQQARYRFWRNTMLIQGIGLALYLVVVLMQARSEHKHRQGTAAALSQQ
ypseu0001X_4158  MYQVNFLPWRLVRQQARYRFWRNTMLIQGIGLALYLVVVLMQARSEHKHRQGTAAALSQQ
yrohd0001_1870   MYQVNFSLWRTERQLARYRFWRNIGLCQCVVFVLVLWAILAQSHRMNINQQGRLAALLQQ
yfred0001_1750   MYQVNFSLWRAERQLTRYRFWRNAGLYQCALLALIVGGILMQSQTVKTRQQNSLAGLLQQ
yente0001X_2490  MYQVNFSSWRTARQLSRYRFWRNIGLCQGILFAISLVVMQMQLHSRQISQQGSLRALLQQ
ykris0001_1500   ------------------------------------------------------------
yfred0001_1760   ------------------------------------------------------------
yinte0001_1610   MYQVNFSLWRAERQSARYRFWRNIGLCQCLFLIILLVVIQRQSRAVNLSQQNRLAALSQQ
ymoll0001_860    MYQVNFSLWRTERLLARYRFWRNVGLCQCLLLVIALVVAQMQSYAVKIRQQGSLAELTQQ
yberc0001_1330   MYQVNFSLWRTEHLLARYRFWRNAGLCQCSLLVITLVIALIQSHGVQTRQQGSLLELTQQ
                 ############################################################


                         70        80        90       100       110       120
                 =========+=========+=========+=========+=========+=========+
yaldo0001_1450   QAVLSQRHQVIQQEIARLAEMKQRFQLYQQARQPARHYFRLLQHLSEHIPDSCWLEALIP
ypest0001X_3450  QAALSQRQQQVQQAMAQLQRIKQHIQRYHQAHQPARGYSILLQQLSQQIPERCWLMALRQ
ypseu0001X_4158  QAALSQRQQQVQQAMAQLQRIKQHIQRYHQAHQPARGYSILLQQLSQQIPERCWLMALRQ
yrohd0001_1870   QIALSQQQQKVQQTMAQLQHAEQRLQVYRLAHQPARRYSSLLRLLSQQIPQSCWLVSIIP
yfred0001_1750   QTALSQHHKAVQQTMVRLQHVERRLQVYRQVRQPAQRYSTLLQQLSQEIPNSCWLISVIP
yente0001X_2490  QSVLSQQHQQVQQLMARKQRAEQRIQMYRQMHQSAHRYSVLLQQLSQQIPDNCWLISLIP
ykris0001_1500   -----------------------------------------LQLLAQEVPDNCWLISLIP
yfred0001_1760   ------------------------------------------------------------
yinte0001_1610   QVALSQHHQEVQQAMAQLRHWEHRAQIYRQAHQLAHRYTTLLQQLSQQIPDSCWLVSLVP
ymoll0001_860    QVALSQHHQKVQQAIAQLQQTEQRLLIYRQAHQSARRYATLLQQLSQQIPDSCWLVSLVP
yberc0001_1330   QGALSQHYQEVQHAMAQLQQTEQRVQIYRQAHQSARRYTTLLQQLSQQIPDSCWLISLMP
                 ############################################################


                        130       140       150       160       170       180
                 =========+=========+=========+=========+=========+=========+
yaldo0001_1450   QGNILAFTVISRDYIAIDTFLRMLYRQPLLEKVRLEGITQRDDGNFRFVVRANWQPGSGV
ypest0001X_3450  QGDVLSFEVISRDYATINAFLAMLGRQPLLVNVSLQGITQQDEGGFRFVVHASWRDENHH
ypseu0001X_4158  QGDVLSFEVISRDYATINAFLAMLGRQPLLVNVSLQGITQQDEGGFRFVVHASWRDENHH
yrohd0001_1870   QEDTLVFSAFCQDYAAIDDFLIMLSRLSLLTNVILQRITQ-DEGHFRFVVHASWQDEGNN
yfred0001_1750   QGDKLVFEAISQDYANINTFLVN-------------------------------------
yente0001X_2490  QSDKLVFEAVSQDYAAIHDFLARLGRQSLLANVRLQRIAQRDDGHFRFLAHADWPQEGVS
ykris0001_1500   KDEHLIFEAVSQDYAAIHDFLVKLRRQSLLANVRLQKIAQQDDGYFRFTAQANWQEEDIY
yfred0001_1760   --------------------------LPLLANIHLQTIAQRDDGNFRFVAQADWQGGENH
yinte0001_1610   QGDKVVFEVMSQSYAAINLFLVRLGRLPLFTKVHLQKIVQQDDGNFRFAVWADWQDRDSD
ymoll0001_860    QGDRVIFEAISQEYAAINDFLVQLGRQSLLANVRLQKIAQQDDGHFRFAVWANWRGGDDN
yberc0001_1330   QGDRLLFEVISQEYAAINHFLVQLGRQSLLANVQLQKIAQQDDGNFRFAVWADWQSGEAN
                 ######################################################      


                        190       200
                 =========+=========+=
yaldo0001_1450   K--------------------
ypest0001X_3450  VEGHHAEGHDIDGHHVAGDYE
ypseu0001X_4158  VEGHHAEGHDIDGHHVAGDYE
yrohd0001_1870   NDE------------------
yfred0001_1750   ---------------------
yente0001X_2490  DE-------------------
ykris0001_1500   YDE------------------
yfred0001_1760   NDE------------------
yinte0001_1610   E--------------------
ymoll0001_860    NE-------------------
yberc0001_1330   HE-------------------
```

```
Parameters used
Minimum Number Of Sequences For A Conserved Position: 6
Minimum Number Of Sequences For A Flanking Position: 9
Maximum Number Of Contiguous Nonconserved Positions: 8
Minimum Length Of A Block: 10
Allowed Gap Positions: With Half
Use Similarity Matrices: Yes
```

```
Flank positions of the 1 selected block(s)
Flanks: [1  174]  

New number of positions in PGL1_unique_yersinia-CLUSTERS.dir/PGL1_unique_yersinia-CL1015/PGL1_unique_yersinia-CL1015.muscle.fasta.gblo:  174  (86% of the original 201 positions)
```
