# Supplementary material for: Genomic characterization of the Yersinia genus
Source: Genome Biol. 2010 Jan 4;11(1):R1. doi: 10.1186/gb-2010-11-1-r1 (PMC2847712; doi:10.1186/gb-2010-11-1-r1)
Supplement: Additional file 15 — The top level directory consists of a directory called Additional_cluster_files and 5010 directories, one for each multi-protein cluster family. (This top level directory has been split into three data files for uploading purposes (Additional files 15, 16, 17).) Within the directory are the following files: PGL1_unique_Yersinia_unclustered.out - list of all protein singletons that MCL did not group into a cluster (see Materials and Methods); PGL1_Yersinia_unique_locus_tags.txt - names of the 11 locus tag prefixes used for each genome; PGL1_unique_Yersinia.gff - mapping each Yersinia protein to a cluster in tab delimited GFF; PGL1_unique_Yersinia.sigfile - list of the longest protein in each cluster; PGL1_unique_Yersinia.summary - summary table of features of each of the clusters; PGL1_unique_Yersinia.table - summary table of each protein in the clusters. Within each cluster directory are the following files, where 'x' is the cluster name: PGL1_unique_Yersinia-x.faa - multifasta file of the proteins in the cluster; PGL1_unique_Yersinia-x.summary - summary of the properties of the proteins; PGL1_unique_Yersinia-x.matches - blast matches between the proteins of the cluster; PGL1_unique_Yersinia-x.muscle.fasta - muscle alignment of the proteins; PGL1_unique_Yersinia-x.muscle.fasta.gblo - gblocks output of muscle alignment (that is, auto-trimmed alignment); PGL1_unique_Yersinia-x.muscle.fasta.gblo.htm - as above in html format; PGL1_unique_Yersinia-x.muscle.tree - treefile from muscle alignment; PGL1_unique_Yersinia-x.sif - matches between proteins in simple interaction format for display on graphing software. [file gb-2010-11-1-r1-S15.zip › clusters/PGL1_unique_yersinia-CL1016/PGL1_unique_yersinia-CL1016.muscle.fasta.gblo.htm]

PGL1\_unique\_yersinia-CL1016.muscle.fasta


## Gblocks 0.91b Results

Processed file: **PGL1\_unique\_yersinia-CL1016.muscle.fasta**  
Number of sequences: **11**  
Alignment assumed to be: **Protein**  
New number of positions: **373** (selected positions are underlined in blue)

```
                         10        20        30        40        50        60
                 =========+=========+=========+=========+=========+=========+
yruck0001_1620   ---------------------------MKFSPRSHGIGIVFLLLYCSLCRS---------
ypseu0001X_4155  ----MGVPSHSRSSNSHLSDSKLSNSASSNSTSSNSVLSNSVLSNNVASSNISPNNASSS
ypest0001X_3480  ------------------------------------------------------------
yaldo0001_1480   -----------MNINTKIWISPLFYRVSKWSKLARHILLYGVLSSVSICCAASYS-----
ymoll0001_890    ----------------------------------------MLLLSLLLTSTASHG-----
yberc0001_1360   -----------------------------------------MLLSLLFTNTALLG-----
yinte0001_1640   ----------------------------------------MLLLVLLTSRAFS-------
yrohd0001_1910   ------------------------------------------------------------
yfred0001_1790   ------------------------------------------------------------
ykris0001_1530   MNLMGFYRFKIKNTFKINKLIKVKAGFWTAYSMAREPLFIFLLLSALLTIPFSIN-----
yente0001X_2530  ------------------------------------------------------------
                                                                             


                         70        80        90       100       110       120
                 =========+=========+=========+=========+=========+=========+
yruck0001_1620   ------EQSEPPVSLVFQNAPFSVVLQSLADYKNLNLVASANVGGNLSLRLVDVPWQQAL
ypseu0001X_4155  NASLPRTRSGGPVTLEFQDAPVSVILQALADYRQLNLITTTGVGGNLSLRLIEVPWEQAL
ypest0001X_3480  -------------------------LQALADYRQLNLITTTGVGGNLSLRLIEVPWEQAL
yaldo0001_1480   ------TGKGQLVSLEFQDAPATLVLQALADYQQLNLVATSGIGGNLSLRLNEVPWEQAL
ymoll0001_890    ------TKGGPPVTLAFQDAPVSVVLQALADYQQLNLVVAAGVSGNISLRLVDVPWEQAL
yberc0001_1360   ------AKGGPPVTLAFQDAPVSVVLQALADYQQLNLVVAAGVSGNISLRLVDVPWEQAL
yinte0001_1640   ------AKTGVPVTVEFHDAPITVVLQALADYQQLNLVIAAGVEGNLSLRLADVPWEQAL
yrohd0001_1910   -----------------------VVLQALADYQQLNLVIAAGVGGNLSLRLVDIPWEQAL
yfred0001_1790   -----------------------MVLQALADYQQLNLVIAADVGGNLSLRLVDIPWEQAL
ykris0001_1530   ------AKGGVPISLEFQDAPAPMVLQALADYQQLNLIIAADIGSNLSLRLVDVPWEQAL
yente0001X_2530  -----------------------VVLQALADYQQLNLVIASDIGANLSLRLVDVPWDQAL
                                         ####################################


                        130       140       150       160       170       180
                 =========+=========+=========+=========+=========+=========+
yruck0001_1620   DTVLRMGNLTVEREGNLLLVLTEQEVATRFLHDQGQREKKAKHQVLDRHSQVLLHAEAEE
ypseu0001X_4155  AIILRMGRLKAEREGTVMMVFTEQEIQER----QQRTKQQAAPEALANLTLALQYANAEQ
ypest0001X_3480  AIILRMGRLKAEREGTVMMVFTEQEIQER----QQRTKQQAAPEALANLTLALQYANAEQ
yaldo0001_1480   AITLRMSRLTVERDGAVMMVFTEQELQER----RQQAEQKALPETLSNLTLALQYADAQQ
ymoll0001_890    AIILRMGHLKAEREGAVMMVFTEQDIEER----QQRAEQKAAPDSMSSLTLALQHADAEH
yberc0001_1360   TIILRMGHLKVEREGAVMMVFTEQDIEER----QQRTKQKTEPDSLSNLTLALQHADAEQ
yinte0001_1640   AIILRMGRLKVEREGSVMMVFTEHEMQER----RQRAEQKNAPEPLNNFSLALQYADAEQ
yrohd0001_1910   TTVLRMSRLTVEREGAVMMVFTEQELEEK----RQRSQQKAAPESLSNLSIALQYADAEQ
yfred0001_1790   ATVLRMSRLKVEREGAIMMVFTEQDIEER----QQQAKQKAAPESLSNLTLALQYADAES
ykris0001_1530   AIVLRMSRLKVEREGAVMMVFTEQDVEDR----RLQAEQKMGPESLSNLSLALQYADAEQ
yente0001X_2530  AIVLRMSHLKVEREGAVMLVFTEQDSEER----QLRAEQKTAPESLSNLSIALQYANAEE
                 #############################    ###########################


                        190       200       210       220       230       240
                 =========+=========+=========+=========+=========+=========+
yruck0001_1620   IAANLNVNHGGLLSPQGRVFADKRTNRLLIRDTAESIAALKAWLSELDSPLQQVQLAAHI
ypseu0001X_4155  VADSLDPLQGGLLSPLGSVVADKRTNTLLIRDTPASLALLKNWLIEMDLPLQQVQLSAHI
ypest0001X_3480  VADSLDPLQGGLLSPLGSVVADKRTNTLLIRDTPASLALLKNWLIEMDLPLQQVQLSAHI
yaldo0001_1480   VADSLNLPEGGLLSPLGSVVVDKRTNTLLIRDTPASLALLKVWLAEMDLPLQQVQLAAHI
ymoll0001_890    VAESLE--EGGLLSPLGSVVADKRTNTLLIRDTPASLAILKGWLAEMDLPLQQIQLAAHI
yberc0001_1360   VADSLD--ESELLSPLGSVMVDKRTNTLLIRDTPASLAVLKGWLAEMDLPLQQVQLAAHI
yinte0001_1640   VADSLTLPEGGLLSPLGSVVVDKRTNTLLIRDTPASLALLKNWLAEMDLPLQQVQLAAHI
yrohd0001_1910   VADSLDLAEGGVLSPLGSVVVDKRTNTLLIRDTPASLALLQSWLVEMDLPLQQVQLAAHI
yfred0001_1790   VADSLNLAEGGLLSPLGSVVVDKRTNTLLIRDTPASLALLKSWLVEMDLPLQQVQLAAHI
ykris0001_1530   VADSLNLAEGGLLSPLGSVVVDKRTNTLLIRDTPASLALLKSWLAEMDLPLQQVQLAAHI
yente0001X_2530  VADSLNLAEGGVLSPLGSVVVDKRTNTLLIRDTPASLALLKNWLAEMDLPLQQVQLAAHI
                 ############################################################


                        250       260       270       280       290       300
                 =========+=========+=========+=========+=========+=========+
yruck0001_1620   VTISSESLHELGVRWGIPARDSGGSALRINNFNVGLPIQNSAITAGFNIARISGQLLDLE
ypseu0001X_4155  VTISSEDLQELGVRWGM-GEGKGNTALRINDFNVNLPLPNSAASVGFHVARIGGRLLELE
ypest0001X_3480  VTISSEDLQELGVRWGM-GEGKGNTALRINDFNVNLPLPNSAASVGFHVARIGGRLLELE
yaldo0001_1480   VTMSRENLHELGVRWGM-DEATQPRALRVNNFNVNLPLPNSALSAGFNVARIGGRLLELE
ymoll0001_890    VTMSRENLQELGVRWGM-GEPPHTTSLRVNDFNVNLPLANSAVSAGFNVARIGGRLLELE
yberc0001_1360   VTMSRENLQELGVRWGM-GESQHATSLRVNDFNVNLPLANSVVNAGFNVARIGGRLLELE
yinte0001_1640   VTMSREHLQELGVRWGM-DDAKQARSLRVNNFNVNLPLPNSALSAGFNVARIGGRLLELE
yrohd0001_1910   VTISRENLQELGVRWGM-GEAKQNSSLRMNDFNVNLPLPNRVISAGFNVARIGGRLLELE
yfred0001_1790   VTISRENLHELGVRWGM-GEGKPNTSLKMSDFNVNLPLPNSAINAGFNVARIGGRLLELE
ykris0001_1530   VTISRENLQELGVRWGM-GEPQPTSALRMSDFNVNLPLPTSAVSAGFNVARIGGRLLELE
yente0001X_2530  VTMSRENLQELGVRWGM-GDTKPTASLRMSDFNVNLPLPNSAVSAGFNVARIGGRLLELE
                 ################# ##########################################


                        310       320       330       340       350       360
                 =========+=========+=========+=========+=========+=========+
yruck0001_1620   LSALEQENQVEIIASPRLTTSHQQTASIKQGTDIPYSVSSGKNGGTTVEFKEAVLGMEVT
ypseu0001X_4155  LSALEQENQVDIIASPRLITSHQQTASIKQGSDIPYTVSRGKKEAAAIEFKEAVLGMEVT
ypest0001X_3480  LSALEQENQVDIIASPRLITSHQQTASIKQGSDIPYTVSRGKKEAAAIEFKEAVLGMEVT
yaldo0001_1480   LSALEQENQVDIIASPRLVTSHQQTASIKQGSDIPYTVSRGKKGVPTIEFKEAVLGMEVT
ymoll0001_890    LSALEQENQVDIIASPRLVASHQQTASIKQGSDIPYTVSRGEKGAASIEFKEAVLGMEVT
yberc0001_1360   LSALEQENQVDIIASPRLVASHLQTASIKQGSDIPYAVARGDKGAVSIEFKEAVLGMEVT
yinte0001_1640   LSALEQENQVDIIASPRLVTSHQQTASIKQGSDIPYTVSRGKKGGPTIEFKEAVLGMEVT
yrohd0001_1910   LSALEQENQIDIIASPHLVASHQQTASIKQGSDIPYTVSKGKKGISTVEFKEAVLGMEVT
yfred0001_1790   LSALEQENQVDIIASPRLVTSHQQTASIKQGSDIPYTVSGGKKGATTIEFKEAVLGMEVT
ykris0001_1530   LSALEQENQVDIIASPRLVTSHQQTASIKQGSDIPYTVSRGKKGTTTIEFKEAVLGMEVT
yente0001X_2530  LSALEQENQVDIIASPRLVTSHQQTASIKQGSDIPYTVSQGKKGATTIEFKEAVLGMEVT
                 ############################################################


                        370       380       390       400       410       420
                 =========+=========+=========+=========+=========+=========+
yruck0001_1620   PRILGAGKITLKLKISQNMPGMAIKRGENESLAIDKQEIKTQITVNDGETIVLGGIFQQK
ypseu0001X_4155  PKILRNGKIILDLKISQNMPGITIKRGESEMLLIDKQEIKTQVTVNDGETIVLGGIFQQK
ypest0001X_3480  PKILRNGKIILDLKISQNMPGITIKRGESEMLLIDKQEIKTQVTVNDGETIVLGGIFQQK
yaldo0001_1480   PKILRNGKITLNLKISQNMPGMAIKRGDSEALLIDKQEIKTQITVNDGETIVLGGIFQQK
ymoll0001_890    PKILRNGKITLNLKISQNMPGMTIKRGDTEALLIDKQEIKTQITVNDGETIVLGGIFQQK
yberc0001_1360   PKILRNGKITLNLKISQNMPGMTIKRGDTEALLIDKQEIKTQITVNDGETIVLGGIFQQK
yinte0001_1640   PKILRNGKITLNLKISQNMPGMAIKRGDSEALLIDKQEIKTQITVNDGETIVLGGIFQQK
yrohd0001_1910   PKILRNGKITLDLKISQNMPGMAMKRGDSETLLIDKQEIKTQITVNDGETIVLGGIFQQK
yfred0001_1790   PKILRNGKITLNLKISQNMPGMAMKRGDSETLLIDKQEIKTQITVNDGETIVLGGIFQQK
ykris0001_1530   PKILRNGKITLNLKISHNMPGMAMKRGDSETLLIDKQEIKTQITVNDGETIVLGGIFQQK
yente0001X_2530  PKILRNGKITLNLKISQNMPGMAMKRGDSETLLIDKQEIQTQITVNDGETIVLGGIFQQK
                 ############################################################


                        430       440       450       460
                 =========+=========+=========+=========+====
yruck0001_1620   NSRGANKVPVLSDIPLLGHLFKQTVNAKSRRELVIFITPRLISI
ypseu0001X_4155  KRQSVNKVPLLADIPLLGAMFRQDTQQQSRRELVIFITPKLISA
ypest0001X_3480  KRQSVNKVPLLADIPLLGAMFRQDTQQQSRRELVIFITPKLISA
yaldo0001_1480   NNQGINKVPILADIPLLGALFKQDVRAQTRRELVIFITPRLIGI
ymoll0001_890    NSQAVNKVPILADIPWLGALFKQDAQQQNRRELVIFITPRLISA
yberc0001_1360   NNQAVNKVPILADIPWLGALFKQDTKQQSRRELVIFITPRLISA
yinte0001_1640   NSQGVNKVPILADIPWLGALFKQDTQQQSRRELVIFITPRLISA
yrohd0001_1910   STQGVHKVPVLADIPWLGVLFKQDAQQQSRRELVIFITPKLINA
yfred0001_1790   SSQGVNKVPILGDIPWLGGLFKQDTQQQNRRELVIFITPRLISA
ykris0001_1530   NSQGVNKVPMLADIPWLGGA-----------------------V
yente0001X_2530  SSQGVNKVPLLADIPWLGGLFKQDTQQQSRRELVIFITPKLISA
                 ##########################################
```

```
Parameters used
Minimum Number Of Sequences For A Conserved Position: 6
Minimum Number Of Sequences For A Flanking Position: 9
Maximum Number Of Contiguous Nonconserved Positions: 8
Minimum Length Of A Block: 10
Allowed Gap Positions: With Half
Use Similarity Matrices: Yes
```

```
Flank positions of the 3 selected block(s)
Flanks: [85  149]  [154  257]  [259  462]  

New number of positions in PGL1_unique_yersinia-CLUSTERS.dir/PGL1_unique_yersinia-CL1016/PGL1_unique_yersinia-CL1016.muscle.fasta.gblo:  373  (80% of the original 464 positions)
```
