# Supplementary material for: Genomic characterization of the Yersinia genus
Source: Genome Biol. 2010 Jan 4;11(1):R1. doi: 10.1186/gb-2010-11-1-r1 (PMC2847712; doi:10.1186/gb-2010-11-1-r1)
Supplement: Additional file 15 — The top level directory consists of a directory called Additional_cluster_files and 5010 directories, one for each multi-protein cluster family. (This top level directory has been split into three data files for uploading purposes (Additional files 15, 16, 17).) Within the directory are the following files: PGL1_unique_Yersinia_unclustered.out - list of all protein singletons that MCL did not group into a cluster (see Materials and Methods); PGL1_Yersinia_unique_locus_tags.txt - names of the 11 locus tag prefixes used for each genome; PGL1_unique_Yersinia.gff - mapping each Yersinia protein to a cluster in tab delimited GFF; PGL1_unique_Yersinia.sigfile - list of the longest protein in each cluster; PGL1_unique_Yersinia.summary - summary table of features of each of the clusters; PGL1_unique_Yersinia.table - summary table of each protein in the clusters. Within each cluster directory are the following files, where 'x' is the cluster name: PGL1_unique_Yersinia-x.faa - multifasta file of the proteins in the cluster; PGL1_unique_Yersinia-x.summary - summary of the properties of the proteins; PGL1_unique_Yersinia-x.matches - blast matches between the proteins of the cluster; PGL1_unique_Yersinia-x.muscle.fasta - muscle alignment of the proteins; PGL1_unique_Yersinia-x.muscle.fasta.gblo - gblocks output of muscle alignment (that is, auto-trimmed alignment); PGL1_unique_Yersinia-x.muscle.fasta.gblo.htm - as above in html format; PGL1_unique_Yersinia-x.muscle.tree - treefile from muscle alignment; PGL1_unique_Yersinia-x.sif - matches between proteins in simple interaction format for display on graphing software. [file gb-2010-11-1-r1-S15.zip › clusters/PGL1_unique_yersinia-CL1017/PGL1_unique_yersinia-CL1017.muscle.fasta.gblo.htm]

PGL1\_unique\_yersinia-CL1017.muscle.fasta


## Gblocks 0.91b Results

Processed file: **PGL1\_unique\_yersinia-CL1017.muscle.fasta**  
Number of sequences: **11**  
Alignment assumed to be: **Protein**  
New number of positions: **407** (selected positions are underlined in blue)

```
                         10        20        30        40        50        60
                 =========+=========+=========+=========+=========+=========+
yruck0001_17690  MKRAVITGLGIVSSIGNNQQEVLAALQEGRSGITFAQEFKDAGMRSHIWGDVK-DLDV-T
ykris0001_40730  MKRAVITGLGIVSSIGNNQQEVLASLQEGRSGITFSQEFKDAGMRSHVWGDVKLAPETIT
yente0001X_3035  MKRAVITGLGIVSSIGNNQQEVLASLQEGRSGITFSQEFKDAGMRSHVWGDVKLAPEAIT
yaldo0001_24700  MKRAVITGLGIVSSIGNNQQEVLASLRKGRSGITFSQEFKDAGMRSHVWGAVKLESEP-K
yrohd0001_34150  MKRAVITGLGIVSSIGNNQQEVLAALQEGRSGITFAQEFKDAGMRSHVWGDVKLASEP-K
yfred0001_35130  MKRAVITGLGIVSSIGNNQQEVLASLQEGRSGITFAQEFKDAGMRSHVWGDVKLASEP-K
ymoll0001_23310  MKRAVITGMGIVSSIGNNQQEVLASLQEGRSGITFAQEFKDAGMRSHVWGAVKLASEP-K
yberc0001_22740  MKRAVITGLGIVSSIGNNQQEVLASLQEGRSGITFAQEFKDAGMRSHVWGDVKLAAEP-K
yinte0001_23710  MKRAVITGLGIVSSIGNNQQEVLASLQEGRSGITFAQEFKDAGMRSHVWGDVKLASEP-K
ypseu0001X_2956  MKRAVITGLGIVSSIGNNQQEVLASLQEGRSGITFAQEFKDAGMRSHVWGDVKLQSEP-K
ypest0001X_3044  MKRAVITGLGIVSSIGNNQQEVLASLQEGRSGITFAQEFKDAGMRSHVWGDVKLQSEP-K
                 ########################################################## #


                         70        80        90       100       110       120
                 =========+=========+=========+=========+=========+=========+
yruck0001_17690  SRIDRKVLRFMSDASVYAYLAMQEAIEDSKLADDQVSNFRSGLVVGSGGGSPRNQVAGSD
ykris0001_40730  ENIDRKVLRFMSDASVYAYLSMKQAIEDSGLTEEQVSNFRSGLVVGSGGGSPRNQVAGSD
yente0001X_3035  ENIDRKVLRFMSDASVYAYLSMKQAIEDSGLTEEQVSNFRSGLVVGSGGGSPRNQVAGSD
yaldo0001_24700  DLIDRKVLRFMSDASIYAYLAMQEAIADSKLTEEQISNFRSGLVVGSGGGSPRNQVAGSD
yrohd0001_34150  DLIDRKVLRFMSDASIYAYLAMQEAIADSGLAEDQVSNFRSGLVVGSGGGSPRNQVAGSD
yfred0001_35130  DLIDRKVLRFMSDASIYAYLAMQEAITDSGLAPEQVSNFRSGLVVGSGGGSPRNQVAGSD
ymoll0001_23310  DLIDRKVLRFMSDASIYAYLAMQEAIADSGLSDEQVSNFRSGLVVGSGGGSPRNQVAGSD
yberc0001_22740  DLIDRKVLRFMSDASIYAYLAMQEAIADSGLSDEQVSNFRSGLVVGSGGGSPRNQVAGSD
yinte0001_23710  DLIDRKVLRFMSDASIYAFLAMQEAIADSGLSDDQVSNFRSGLVVGSGGGSPRNQVAGSD
ypseu0001X_2956  DLIDRKVLRFMSDASIYAYLAMQEAIADSGLSDSQVSNFRSGLVVGSGGGSPRNQVAGSD
ypest0001X_3044  DLIDRKVLRFMSDASIYAYLAMQEAIADSGLSDSQVSNFRSGLVVGSGGGSPRNQVAGSD
                 ############################################################


                        130       140       150       160       170       180
                 =========+=========+=========+=========+=========+=========+
yruck0001_17690  GMRA-KGLRGVGPYMVTKAMASGVSACLATPFKIKGVNYSISSACATSAHCIGHAVELIQ
ykris0001_40730  GMRA-KGLRGVGPYMVTKAMASGVSACLATPFKIKGVNYSISSACATSAHCIGHALELIQ
yente0001X_3035  GMRA-KGLRGVGPYMVTKAMASGVSACLATPFKIKGVNYSISSACATSAHCIGHALELIQ
yaldo0001_24700  AMRTPRGLKGVGPYMVTKAMASGVSACLATPFKIKGVNYSISSACATSAHCIGHAVELIQ
yrohd0001_34150  AMRTPRGLKGVGPYMVTKAMASGVSACLATPFKIKGVNYSISSACATSAHCIGHALELIQ
yfred0001_35130  AMRTPRGLKGVGPYMVTKAMASGVSACLATPFKIKGVNYSISSACATSAHCIGHALELIQ
ymoll0001_23310  AMRTPRGLKGVGPYMVTKAMASGVSACLATPFKIKGVNYSISSACATSAHCIGHALELIQ
yberc0001_22740  AMRTPRGLKGVGPYMVTKAMASGVSACLATPFKIKGVNYSISSACATSAHCIGHALELIQ
yinte0001_23710  AMRTPRGLKGVGPYMVTKAMASGVSACLATPFKIKGVNYSISSACATSAHCIGHALELIQ
ypseu0001X_2956  AMRTPRGLKGVGPYMVTKAMASGVSACLATPFKIKGVNYSISSACATSAHCIGHALELIQ
ypest0001X_3044  AMRTPRGLKGVGPYMVTKAMASGVSACLATPFKIKGVNYSISSACATSAHCIGHALELIQ
                 ############################################################


                        190       200       210       220       230       240
                 =========+=========+=========+=========+=========+=========+
yruck0001_17690  LGKQDVVFAGGGEELCWEMACEFDAMGALSTKYNDTPEKASRTYDKDRDGFVIAGGGGMV
ykris0001_40730  LGKQDVVFAGGGEELCWEMSCEFDAMGALSTKYNETPAKASRTYDQDRDGFVIAGGGGMV
yente0001X_3035  LGKQDVVFAGGGEELCWEMSCEFDAMGALSTKYNETPDKASRTYDQYRDGFVIAGGGGMV
yaldo0001_24700  LGKQDVVFAGGGEELCWEMSCEFDAMGALSTKYNDTPEKASRTYDQDRDGFVIAGGGGMV
yrohd0001_34150  LGKQDVVFAGGGEELCWEMACEFDAMGALSTKYNETPAKASRTYDQDRDGFVIAGGGGMV
yfred0001_35130  LGKQDVVFAGGGEELCWEMACEFDAMGALSTKYNETPTKASRTYDQDRDGFVIAGGGGMV
ymoll0001_23310  LGKQDVVFAGGGEELCWEMSCEFDAMGALSTKYNDTPTKASRTYDKDRDGFVIAGGGGMV
yberc0001_22740  LGKQDVVFAGGGEELCWEMACEFDAMGALSTKYNETPTKASRTYDQDRDGFVIAGGGGMV
yinte0001_23710  LGKQDVVFAGGGEELCWEMACEFDAMGALSTKYNETPAKASRTYDQDRDGFVIAGGGGMV
ypseu0001X_2956  LGKQDVVFAGGGEELCWEMACEFDAMGALSTKYNDTPAKASRTYDQDRDGFVIAGGGGMV
ypest0001X_3044  LGKQDIVFAGGGEELCWEMACEFDAMGALSTKYNDTPAKASRTYDQDRDGFVIAGGGGMV
                 ############################################################


                        250       260       270       280       290       300
                 =========+=========+=========+=========+=========+=========+
yruck0001_17690  VVEELEHALARGAHIYAEIVGYGATSDGADMVAPSGEGAVRCMQMAMQGVDTPIDYMNVH
ykris0001_40730  VVEELEHALARGAHIYAEIVGYGATSDGADMVAPSGEGAVRCMKMAMEGVDTPIDYMNVH
yente0001X_3035  VVEELEHALARGAHIYAEIVGYGATSDGADMVAPSGEGAVRCMQMAMEGVDTPIDYMNVH
yaldo0001_24700  VVEELEHALARGAHIYAEIVGYGATSDGADMVAPSGEGAVRCMKMAMEGVDTPIDYMNVH
yrohd0001_34150  VVEELEHALARGAHIYAEIVGYGATSDGADMVAPSGEGAVRCMQMAMAGVDTPIDYMNVH
yfred0001_35130  VVEELEHALARGAHIYAEIVGYGATSDGADMVAPSGEGAVRCMQMAMQGVDTPIDYMNVH
ymoll0001_23310  VVEELEHALARGAHIYAEIVGYGATSDGADMVAPSGEGAVRCMQMAMQGVDTPIDYMNVH
yberc0001_22740  VVEELEHALARGAHIYAEIVGYGATSDGADMVAPSGEGAVRCMQMAMEGVDTPIDYMNVH
yinte0001_23710  VVEELEHALARGAHIYAEIVGYGATSDGADMVAPSGEGAVRCMQMAMAGVDTPIDYMNVH
ypseu0001X_2956  VVEELEHALARGAHIYAEIVGYGATSDGADMVAPSGEGAVRCMQMAMAGVDTPIDYMNVH
ypest0001X_3044  VVEELEHALARGAHIYAEIVGYGATSDGADMVAPSGEGAVRCMQMAMAGVDTPIDYMNVH
                 ############################################################


                        310       320       330       340       350       360
                 =========+=========+=========+=========+=========+=========+
yruck0001_17690  GTSTPVGDVKELGAIREIFGDNTPAISSTKAMTGHSLGAAGVHEAIFSLLMVEHGFIAPS
ykris0001_40730  GTSTPVGDVKELGAIREVFGDNTPAISSTKAMTGHSLGAAGVHEAIFSLLMVEHGFIAPS
yente0001X_3035  GTSTPVGDVKELGAIREVFGDNTPAISSTKAMTGHSLGAAGVHEAIFSLLMVEHGFIAPS
yaldo0001_24700  GTSTPVGDVKELGAIREVFGDNTPAISSTKAMTGHSLGAAGVHEAIYSLLMVEHGFIAPS
yrohd0001_34150  GTSTPVGDVKELGAIREVFGNDTPAISSTKAMTGHSLGAAGVHEAIFSLLMVEHGFIAPS
yfred0001_35130  GTSTPVGDVKELGAIREVFGNNTPAISSTKAMTGHSLGAAGVHEAIFSLLMVEHGFIAPS
ymoll0001_23310  GTSTPVGDVKELGAIREVFGDNTPAISSTKAMTGHSLGAAGVHEAIFSLLMVEHGFIAPS
yberc0001_22740  GTSTPVGDVKELGAIREVFGDNTPAISSTKAMTGHSLGAAGVHEAIFSLLMVEHGFIAPS
yinte0001_23710  GTSTPVGDVKELGAIREVFGDNTPAISSTKAMTGHSLGAAGVHEAIFSLLMVEHGFIAPS
ypseu0001X_2956  GTSTPVGDVKELGAIREVFGNNTPAISSTKAMTGHSLGAAGVHEAIFSLLMVEHGFIAPS
ypest0001X_3044  GTSTPVGDVKELGAIREVFGNNTPAISSTKAMTGHSLGAAGVHEAIFSLLMVEHGFIAPS
                 ############################################################


                        370       380       390       400
                 =========+=========+=========+=========+========
yruck0001_17690  INIETLDEKAEGMNIITEPTKRELTTVMSNSFGFGGTNATLVMRKYQK
ykris0001_40730  INIENLDEKAVGMNIVTEPTQRELTTVMSNSFGFGGTNATLVMRKYQK
yente0001X_3035  INIENLDEQAAGMNIVTEPTKRELNTVMSNSFGFGGTNATLVMRKYQK
yaldo0001_24700  INIDNLDEQAAGMDIITEPTERELTTVMSNSFGFGGTNATLVMRKYPK
yrohd0001_34150  INIDNLDEQAKGMNIITEPTKRELTTVMSNSFGFGGTNATLVMRKYQK
yfred0001_35130  INIENLDEQAAGMNIITEPTKRELTTVMSNSFGFGGTNATLVMRKYQK
ymoll0001_23310  INIDNLDEQAAGMNIVTEPTQRELTTVMSNSFGFGGTNATLVMRKYQK
yberc0001_22740  INIENLDEQAAGMNIVTKPTQRELTTVMSNSFGFGGTNATLVMRKYQQ
yinte0001_23710  INIENLDEKAAGMNIITEPTQRELTTVMSNSFGFGGTNATLVMRKYQK
ypseu0001X_2956  INIDNLDEQAQGMNIITETTQRELTTVMSNSFGFGGTNATLVMRKYQK
ypest0001X_3044  INIDNLDEQAQGMNIITETTQRELTTVMSNSFGFGGTNATLVMRKYQK
                 ################################################
```

```
Parameters used
Minimum Number Of Sequences For A Conserved Position: 6
Minimum Number Of Sequences For A Flanking Position: 9
Maximum Number Of Contiguous Nonconserved Positions: 8
Minimum Length Of A Block: 10
Allowed Gap Positions: With Half
Use Similarity Matrices: Yes
```

```
Flank positions of the 2 selected block(s)
Flanks: [1  58]  [60  408]  

New number of positions in PGL1_unique_yersinia-CLUSTERS.dir/PGL1_unique_yersinia-CL1017/PGL1_unique_yersinia-CL1017.muscle.fasta.gblo:  407  (99% of the original 408 positions)
```
