# Supplementary material for: Genomic characterization of the Yersinia genus
Source: Genome Biol. 2010 Jan 4;11(1):R1. doi: 10.1186/gb-2010-11-1-r1 (PMC2847712; doi:10.1186/gb-2010-11-1-r1)
Supplement: Additional file 15 — The top level directory consists of a directory called Additional_cluster_files and 5010 directories, one for each multi-protein cluster family. (This top level directory has been split into three data files for uploading purposes (Additional files 15, 16, 17).) Within the directory are the following files: PGL1_unique_Yersinia_unclustered.out - list of all protein singletons that MCL did not group into a cluster (see Materials and Methods); PGL1_Yersinia_unique_locus_tags.txt - names of the 11 locus tag prefixes used for each genome; PGL1_unique_Yersinia.gff - mapping each Yersinia protein to a cluster in tab delimited GFF; PGL1_unique_Yersinia.sigfile - list of the longest protein in each cluster; PGL1_unique_Yersinia.summary - summary table of features of each of the clusters; PGL1_unique_Yersinia.table - summary table of each protein in the clusters. Within each cluster directory are the following files, where 'x' is the cluster name: PGL1_unique_Yersinia-x.faa - multifasta file of the proteins in the cluster; PGL1_unique_Yersinia-x.summary - summary of the properties of the proteins; PGL1_unique_Yersinia-x.matches - blast matches between the proteins of the cluster; PGL1_unique_Yersinia-x.muscle.fasta - muscle alignment of the proteins; PGL1_unique_Yersinia-x.muscle.fasta.gblo - gblocks output of muscle alignment (that is, auto-trimmed alignment); PGL1_unique_Yersinia-x.muscle.fasta.gblo.htm - as above in html format; PGL1_unique_Yersinia-x.muscle.tree - treefile from muscle alignment; PGL1_unique_Yersinia-x.sif - matches between proteins in simple interaction format for display on graphing software. [file gb-2010-11-1-r1-S15.zip › clusters/PGL1_unique_yersinia-CL1018/PGL1_unique_yersinia-CL1018.muscle.fasta.gblo.htm]

PGL1\_unique\_yersinia-CL1018.muscle.fasta


## Gblocks 0.91b Results

Processed file: **PGL1\_unique\_yersinia-CL1018.muscle.fasta**  
Number of sequences: **11**  
Alignment assumed to be: **Protein**  
New number of positions: **360** (selected positions are underlined in blue)

```
                         10        20        30        40        50        60
                 =========+=========+=========+=========+=========+=========+
yruck0001_1640   MEKITVTLGVRSYPITIAAGLFNDPASFKPLKAGDQVMLVTNQTLAPLYLDSIQSVLEQS
yrohd0001_1930   MEKITVTLGERSYPITIAAGLFNDPASFEPLKAGDQVMLVTNQTLAPLYLDSLRAVLEQN
yaldo0001_1500   MEKITVTLGERSYPITIAAGLFNDPVSFKPLKVGDQVMLVTNQTLAPLYLDSIRKVLEQG
ypseu0001X_4153  MEKITVTLGERSYPITIAAGLFNDPASFKPLKAGDQVMLVTNQTLAPLYLDSLRAVLEHG
ypest0001X_3500  MEKITVTLGERSYPITIAAGLFNDPASFKPLKAGDQVMLVTNQTLAPLYLDSLRAVLEHG
ykris0001_1550   MEKITVTLGERSYPITIAAGLFKDPASFKPLKAGDQVMLVTNQTLAPLYLDSLRAVLEQS
yente0001X_2550  MEKITVTLGERSYPITIAAGLFNDPASFKPLKAGDQVMLVTNQTLAPLYLDSLRAVLEQG
yinte0001_1660   MEKITVTLGERSYPITIAAGLFNDSASFAPLKAGDQVMLVTNQTLAPLYLDSLRAVLEQS
ymoll0001_910    MEKITVTLGARSYPITIAAGLFNDPASFKPLKAGDQVMLVTNQTLAPLYLDSLRAVLEQS
yberc0001_1380   MEKITVTLGERSYPITIAAGLFNDPASFKPLKAGDQVMLVTNQTLAPLYLDSLRAVLEQS
yfred0001_1810   MEKITVTLGERSYPITIAAGLFNDPASFKPLKAGDQVMLVTNQTLAPLYLDSIRAVLEKS
                 ############################################################


                         70        80        90       100       110       120
                 =========+=========+=========+=========+=========+=========+
yruck0001_1640   GIQVDQVILPDGEQYKSLSVLEQVFSALLEKPHGRDTTLIALGGGVVGDLTGFAAACYQR
yrohd0001_1930   GIKVDQVILPDGEQYKSLSVLEQVFSALLEKPHGRDTTLVALGGGVVGDLTGFAAACYQR
yaldo0001_1500   GVQVDQVILPDGEQYKSLSVLEQVFSALLEKPHGRDTTLVALGGGVVGDLTGFAAACYQR
ypseu0001X_4153  GIKVDQVILPDGEQYKSLSVMEQVFSALLEKPHGRDTTLVALGGGVVGDLTGFAAACYQR
ypest0001X_3500  GIKVDQVILPDGEQYKSLSVMEQVFSALLEKPHGRDTTLVALGGGVVGDLTGFAAACYQR
ykris0001_1550   GIKVDQVILPDGEQYKSLSVLEQVFSALLEKPHGRDTTLVALGGGVVGDLTGFAAACYQR
yente0001X_2550  GIKVDQVILPDGEQYKSLSVLEQVFSALLEKPHGRDTTLVALGGGVVGDLTGFAAACYQR
yinte0001_1660   GIKVDQVILPDGEQYKSLSVLEQVFSALLEKPHGRDTTLVALGGGVVGDLTGFAAACYQR
ymoll0001_910    GIKVDQVILPDGEQYKSLSVLEQVFSALLEKPHGRDTTLVALGGGVVGDLTGFAAACYQR
yberc0001_1380   GIKVDQVILPDGEQYKSLSVLEQVFSALLEKPHGRDTTLVALGGGVVGDLTGFAAACYQR
yfred0001_1810   GIKVDQVILPDGEQYKSLSVLEQVFSALLEKPHGRDTTLVALGGGVVGDLTGFAAACYQR
                 ############################################################


                        130       140       150       160       170       180
                 =========+=========+=========+=========+=========+=========+
yruck0001_1640   GVRFIQVPTTLLSQVDSSVGGKTAVNHPLGKNMIGAFYQPASVIVDLNCLKTLPSRELAS
yrohd0001_1930   GVRFIQVPTTLLSQVDSSVGGKTAVNHPLGKNMIGAFYQPASVVVDLNCLKTLPPRELAS
yaldo0001_1500   GVRFIQVPTTLLSQVDSSVGGKTAVNHPLGKNMIGAFYQPASVVVDLNCLKTLPQRELAS
ypseu0001X_4153  GVRFIQVPTTLLSQVDSSVGGKTAVNHPLGKNMIGAFYQPASVVVDLNCLKTLPPRELAS
ypest0001X_3500  GVRFIQVPTTLLSQVDSSVGGKTAVNHPLGKNMIGAFYQPASVVVDLNCLKTLPPRELAS
ykris0001_1550   GVRFIQVPTTLLSQVDSSVGGKTAVNHPLGKNMIGAFYQPASVVVDLDCLKTLPPRELAS
yente0001X_2550  GVRFIQVPTTLLSQVDSSVGGKTAVNHPLGKNMIGAFYQPASVVVDLNCLKTLPSRELAS
yinte0001_1660   GVRFIQVPTTLLSQVDSSVGGKTAVNHPLGKNMIGAFYQPASVVVDLDCLKTLPSRELAS
ymoll0001_910    GVRFIQVPTTLLSQVDSSVGGKTAVNHPLGKNMIGAFYQPASVVIDLDCLKTLPPRELAS
yberc0001_1380   GVRFIQVPTTLLSQVDSSVGGKTAVNHPLGKNMIGAFYQPASVVVDLNCLKTLPPRELAS
yfred0001_1810   GVRFIQVPTTLLSQVDSSVGGKTAVNHPLGKNMIGAFYQPASVVVDLNCLKTLPPRELAS
                 ############################################################


                        190       200       210       220       230       240
                 =========+=========+=========+=========+=========+=========+
yruck0001_1640   GLAEVIKYGIILDSDFFVWLENNIDALLALDMEALGYCIRRCCELKAAVVAADEREENGL
yrohd0001_1930   GLAEVIKYGIILDADFFVWLEENIDALLELDMSALAYCIRRCCELKADVVAADEHETTGM
yaldo0001_1500   GLAEVIKYGIILDAAFFDWLENNIDALLALDMSALAYCIRRCCELKADVVAADEREESGM
ypseu0001X_4153  GLAEVIKYGIILDAAFFDWLENNIDALLALDMSALAYCIRRCCELKADVVAADEREESGA
ypest0001X_3500  GLAEVIKYGIILDAAFFDWLENNIDALLALDMSALAYCIRRCCELKADVVAADEREESGA
ykris0001_1550   GLAEVIKYGIILDAAFFAWLENNIDALLALDMSALAYCIRRCCELKADVVAADEREESGM
yente0001X_2550  GLAEVIKYGIILDAAFFEWLETNIDSLLALDMSALAYCIRRCCELKADVVAADEREESGM
yinte0001_1660   GLAEVIKYGIILDAAFFDWLENNIGALLALDMSALAYCIRRCCELKADVVAADEREESGM
ymoll0001_910    GLAEVIKYGIILDAAFFDWLEDNIDALLALEMSALAYCIRRCCELKADVVAADEREESGM
yberc0001_1380   GLAEVIKYGIILDAAFFDWLEENIDALLALDMSALAYCIRRCCELKADVVAADEREESGM
yfred0001_1810   GLAEVIKYGIILDAAFFDWLEANIDALLALDMSALAYCIRRCCELKADVVAADEREESGM
                 ############################################################


                        250       260       270       280       290       300
                 =========+=========+=========+=========+=========+=========+
yruck0001_1640   RALLNLGHTYGHAIEAEMGYGVWLHGEAVAVGMVMAAETAHIIGQFSAGDIERIKKLLLR
yrohd0001_1930   RALLNLGHTYGHAIEAEMGYGVWLHGEAVAAGMMMAAQTSYRLGQFSASDVERIKKLLLR
yaldo0001_1500   RALLNLGHTYGHAIEAEMGYGVWLHGEAVAAGMVMAAHTSRRLGQFSVEDVERIKKLLLR
ypseu0001X_4153  RALLNLGHTYGHAIEAEMGYGVWLHGEAVAAGMVMAAQTSRRLGQLSVSDVERIKKLLLR
ypest0001X_3500  RALLNLGHTYGHAIEAEMGYGVWLHGEAVAAGMVMAAQTSRRLGQLSVSDVERIKKLLLR
ykris0001_1550   RALLNLGHTYGHAIEAEMGYGVWLHGEAVAAGMMMAAHTSRRLGQLSDQDVERIKKLLLR
yente0001X_2550  RALLNLGHTYGHAIEAEMGYGVWLHGEAVAAGMMMAAHTSRRLGQFSAEDVERIKKLLLR
yinte0001_1660   RALLNLGHTYGHAIEAEMGYGVWLHGEAVAAGMMMAAHTSRRLGQFSASDVERIKKLLLR
ymoll0001_910    RALLNLGHTYGHAIEAEMGYGVWLHGEAVAAGMMMAAQTSRRLGQFSASDVERIKKLLLR
yberc0001_1380   RALLNLGHTYGHAIEAEMGYGVWLHGEAVAAGMMMAAHTSRRLGQLSASDVERIKKLILR
yfred0001_1810   RALLNLGHTYGHAIEAEMGYGVWLHGEAVAAGMMMAAHTSRRLGQFSARDVERIKKLLLR
                 ############################################################


                        310       320       330       340       350       360
                 =========+=========+=========+=========+=========+=========+
yruck0001_1640   AGLPVNGPAQMTPESYLPHMMRDKKVLAGELRLVLPTAIGKSEVRGGISHEIVLASITRC
yrohd0001_1930   AGLPVCGPQEMTPESYLPHMMRDKKVLAGELRLVLPTAIGKSEVRGGVAHEIVLASIADC
yaldo0001_1500   AGLPICGPQEMTPESYLPHMMRDKKVLAGELRLVLPTAIGTSEIRGGIAHDMVLASIADC
ypseu0001X_4153  AGLPVCGPKEMAPESYLPHMMRDKKVLAGELRLVLPTAIGKSEIRGGVAHDMVLASIADC
ypest0001X_3500  AGLPVCGPKEMAPESYLPHMMRDKKVLAGELRLVLPTAIGKSEIRGGVAHDMVLASIADC
ykris0001_1550   AGLPVSGPQEMTPESYLPHMMRDKKVLAGELRLVLPTAIGQSEVRGGVAHDMVLASITDC
yente0001X_2550  AGLPVSGPQKMTPESYLPHMMRDKKVLAGELRLVLPTAIGQSEIRSGIAHDMVLASIADC
yinte0001_1660   AGLPVCGPQEMTPESYLPHMMRDKKVLAGELRLVLPTAIGKSEIRSGIAHDMVLASITDC
ymoll0001_910    AGLPVCGPQEMTPESYLPHMMRDKKVLAGELRLVLPTAIGQSEIRSGIALDMVLASIADC
yberc0001_1380   AGLPVCGPQEMTPESYLPHMMRDKKVLAGELRLVLPTAIGQSEIRSGIAHDMVLASIADC
yfred0001_1810   AGLPVCGPQEMTPESYLPHMMRDKKVLAGELRLVLPTAIGQSEIRSGIAHDIVLASVADC
                 ############################################################


                 
                 ===
yruck0001_1640   LAG
yrohd0001_1930   Q--
yaldo0001_1500   Q--
ypseu0001X_4153  RP-
ypest0001X_3500  RP-
ykris0001_1550   Q--
yente0001X_2550  QL-
yinte0001_1660   QL-
ymoll0001_910    Q--
yberc0001_1380   QQ-
yfred0001_1810   Q--
```

```
Parameters used
Minimum Number Of Sequences For A Conserved Position: 6
Minimum Number Of Sequences For A Flanking Position: 9
Maximum Number Of Contiguous Nonconserved Positions: 8
Minimum Length Of A Block: 10
Allowed Gap Positions: With Half
Use Similarity Matrices: Yes
```

```
Flank positions of the 1 selected block(s)
Flanks: [1  360]  

New number of positions in PGL1_unique_yersinia-CLUSTERS.dir/PGL1_unique_yersinia-CL1018/PGL1_unique_yersinia-CL1018.muscle.fasta.gblo:  360  (99% of the original 363 positions)
```
