# Supplementary material for: Genomic characterization of the Yersinia genus
Source: Genome Biol. 2010 Jan 4;11(1):R1. doi: 10.1186/gb-2010-11-1-r1 (PMC2847712; doi:10.1186/gb-2010-11-1-r1)
Supplement: Additional file 16 — The top level directory consists of a directory called Additional_cluster_files and 5010 directories, one for each multi-protein cluster family. (This top level directory has been split into three data files for uploading purposes (Additional files 15, 16, 17.) Within the directory are the following files: PGL1_unique_Yersinia_unclustered.out - list of all protein singletons that MCL did not group into a cluster (see Materials and Methods); PGL1_Yersinia_unique_locus_tags.txt - names of the 11 locus tag prefixes used for each genome; PGL1_unique_Yersinia.gff - mapping each Yersinia protein to a cluster in tab delimited GFF; PGL1_unique_Yersinia.sigfile - list of the longest protein in each cluster; PGL1_unique_Yersinia.summary - summary table of features of each of the clusters; PGL1_unique_Yersinia.table - summary table of each protein in the clusters. Within each cluster directory are the following files, where 'x' is the cluster name: PGL1_unique_Yersinia-x.faa - multifasta file of the proteins in the cluster; PGL1_unique_Yersinia-x.summary - summary of the properties of the proteins; PGL1_unique_Yersinia-x.matches - blast matches between the proteins of the cluster; PGL1_unique_Yersinia-x.muscle.fasta - muscle alignment of the proteins; PGL1_unique_Yersinia-x.muscle.fasta.gblo - gblocks output of muscle alignment (that is, auto-trimmed alignment); PGL1_unique_Yersinia-x.muscle.fasta.gblo.htm - as above in html format; PGL1_unique_Yersinia-x.muscle.tree - treefile from muscle alignment; PGL1_unique_Yersinia-x.sif - matches between proteins in simple interaction format for display on graphing software. [file gb-2010-11-1-r1-S16.zip › clusters2/PGL1_unique_yersinia-CL1250/PGL1_unique_yersinia-CL1250.muscle.fasta.gblo.htm]

PGL1\_unique\_yersinia-CL1250.muscle.fasta


## Gblocks 0.91b Results

Processed file: **PGL1\_unique\_yersinia-CL1250.muscle.fasta**  
Number of sequences: **11**  
Alignment assumed to be: **Protein**  
New number of positions: **276** (selected positions are underlined in blue)

```
                         10        20        30        40        50        60
                 =========+=========+=========+=========+=========+=========+
yruck0001_5030   --------MKNQPIIKLYDGNLMPQLGLGVWKASHEEAQLAVTKALEIGYCSIDTAAIYK
ypseu0001X_3772  ----------MQPLIKLHDGRLMPQLGLGVWQASIQETELAVSKALEVGYRSIDTAAIYK
ypest0001X_8820  ----------MQPLIKLYDGRLMPQLGLGVWQASIQETELAVSKALEVGYRSIDTAAIYK
yaldo0001_6470   --------MATQPIIKLHDGNLMPQLGLGVWQASIEETQFAVSNALEVGYRSVDTAAIYK
yinte0001_6590   --------MATQPIIKLHDGNLMPQLGLGVWRASIEETQLAVSKALEVGYRSVDTAAIYK
ymoll0001_5800   --------MATQPIIKLHDGNLMPQLGLGVWQATIEETQLAVSKALEVGYRSIDTAAIYK
yberc0001_6500   --------MATQPIIKLHDGNLMPQLGLGVWQASIEETQLAVSKALEVGYRLVDTAAIYK
yrohd0001_6210   --------MATQPIIKLHDGNLMPQLGLGVWQASIEETQLAVSKALEVGYRSIDTAAIYK
ykris0001_4750   VILAEEINMATQPIIKLHDGNLMPQLGLGVWQASIEETQLAVTKALEVGYRSIDTAAIYK
yente0001X_5860  --------MATQPIIKLHDGNLMPQLGLGVWQASIEETQLAVSKALEVGYRSIDTAAIYK
yfred0001_41510  --------MATQPIIKLHDGNLMPQLGLGVWQASIEETQLAVSKALEVGYRSIDTAAIYK
                         ####################################################


                         70        80        90       100       110       120
                 =========+=========+=========+=========+=========+=========+
yruck0001_5030   NEEGVGKALRSATVPRKDLFVTTKLWNEDQSHPQQALEASLKKLQLDYIDLYLIHWPDPK
ypseu0001X_3772  NEEGVGKALKAAAVARDELFITTKLWNDDQHNPQQALETSLQKLQLDYIDLYLIHWPDPK
ypest0001X_8820  NEEGVGKALKAAAVARDELFITTKLWNDDQHNPQQALETSLQKLQLDYVDLYLIHWPDPK
yaldo0001_6470   NEEGVGKALKTAGIVREKLFITTKLWNDDQHLPKQALETSLEKLQLDYVDLYLIHWPDPK
yinte0001_6590   NEQGVGAALKAAGVARDELFITTKLWNDSQQDPKKALEESLNKLQLDYVDLYLIHWPNPT
ymoll0001_5800   NEVGVGKALKAAGVPRDELFITTKLWNDDQRYPKQGLKESLEKLQLDYVDLYLIHWPDPA
yberc0001_6500   NEVGVGHALKTAGVAREELFITTKLWNDGQRYPKQGLEESLEKLQLDYVDLYLIHWPDPA
yrohd0001_6210   NEEGVGKALKAANIARDEIFITTKLWNDSQRNPQQALEESLEKLQLDYVDLYLIHWPDPT
ykris0001_4750   NEEGVGQALKTTNIARDELFITTKLWNSNQNNPQQALEESLKKLQLDYVDLYLIHWPDPA
yente0001X_5860  NEEGVGQALKSTHIARDELFITTKLWNSDQDNPQQALEESLKKLQLDYVDLYLIHWPDPT
yfred0001_41510  NEVGVGQALKTANIPRDEIFITTKLWNDSQRNPKQALEESLEKLQLDYVDLYLIHWPDPT
                 ############################################################


                        130       140       150       160       170       180
                 =========+=========+=========+=========+=========+=========+
yruck0001_5030   QDRYVDAWRKLITLKEQGLIRSIGVCNFHIPHLQRLLDETGVAPTINQIELHPLLQQRQL
ypseu0001X_3772  QDHYVSAWRELVTLKEQGLIRSIGVCNFHIPHLQRLIDETGIAPTVNQIELHPLLQQRQL
ypest0001X_8820  QDHYVSAWRELVTLKEQGLIRSIGVCNFHIPHLQRLIDETGIAPTVNQIELHPLLQQRQL
yaldo0001_6470   QDRYVSAWRELLVLKEQGLARSVGVCNFQIPHLQRLIDETGIAPSVNQIELHPLLQQRQL
yinte0001_6590   QDHYVSAWRGLIKLKEQGLIRSIGVCNFHIPHLQRLIDETGVAPTVNQIELHPLLQQRQL
ymoll0001_5800   QDRYVSAWRELITLKEQGLVRSIGVCNFNIPHLQRLIDETGVAPTLNQIELHPLLQQRQL
yberc0001_6500   QDRYVSAWRELITLKEQGLVRSIGVCNFNIPHLQRLIDETGVAPTLNQIELHPLLQQRQL
yrohd0001_6210   QDNYVSAWRELAVLKKQGLIRSIGVCNFNIPHLQRLIDETGVAPTVNQIELHPLLQQRQI
ykris0001_4750   QDRYVSAWRELIALKEQGLIRSIGVCNFHIPHLQRLIDETGVAPTINQIELHPLLQQRQL
yente0001X_5860  QDRYVSAWRELIALKEQGLIRSIGVCNFNIPHLQRLIDETGVAPAVNQIELHPLLQQRQI
yfred0001_41510  QDRYVSAWRELIALKEQGLIRSVGVCNFTIPHLQRLIDETGIAPTVNQIELHPLLQQRQI
                 ############################################################


                        190       200       210       220       230       240
                 =========+=========+=========+=========+=========+=========+
yruck0001_5030   HAWNATHHIATESWSPLAQGGEGVFDQPLIHQLAQKYDKTPAQIVIRWHLDNGLIVIPKS
ypseu0001X_3772  HAWNATHHIATESWSPLAQGGKGVFDQEIIRKLAQQYNKTPAQIVIRWHLDSGLIVIPKS
ypest0001X_8820  HAWNATHHIATESWSPLAQGGKGVFDQEIIRKLAQQYNKTPAQIVIRWHLDSGLIVIPKS
yaldo0001_6470   HSWNATHHIATESWSPLAQGGEGVFDQTIIHQLAHKYNKSPAQIVIRWHLDNGLIVIPKS
yinte0001_6590   HSWNATHNIATESWSPLAQGGEGVFDQSVIRQLAQKYNKTPAQVVIRWHLDCGLIVIPKS
ymoll0001_5800   HAWNATHHIATESWSPLAQGGEGVFDQAVIRQLAQKYNKTPAQIVIRWHLDCGLIVIPKS
yberc0001_6500   HAWNATHHIATESWSPLAQGGEGVFDQPVIRQLAQKYNKTAAQIVIRWHLDCGLIVIPKS
yrohd0001_6210   HAWNATHHIATESWSPLAQGGKGVFDNPVIHQLAQKYHKTPAQIVIRWHLDCGLIVIPKS
ykris0001_4750   HAWNATHHIATESWSPLAQGGDGVFDQAVIRELAQKYGKTPAQIVIRWHLDSGLIVIPKS
yente0001X_5860  HAWNATHHIATESWSPLAQGGDGVFDQAVIRQLAQKYSKTPAQIVIRWHLDSGLIVIPKS
yfred0001_41510  HAWNATHHIATESWSPLAQGGDGVFDQAVIRQLAQKYSKTPAQIVIRWHLDSGLIVIPKS
                 ############################################################


                        250       260       270       280
                 =========+=========+=========+=========+=====
yruck0001_5030   VTPNRIRENFEVFDFKLEKDELSDIAKLDIGHRLGPDPDSF----
ypseu0001X_3772  VTPARIRENFEVFDFKLQKEELLAITKLDCGKRLGPDPEVFGSDR
ypest0001X_8820  VTPARIRENFEVFDFKLQKEELLAITKLDCGKRLGPDPEVFGSDR
yaldo0001_6470   VTPARIRENFDVFDFKLHKDELTTIAKLDSGKRLGPDPDAFGSDA
yinte0001_6590   VTPARIKENFEVFDFKLHKDELTAISKLDSNKRLGPDPDVFGSDN
ymoll0001_5800   VTPTRIRENFEVFDFKLHKDELTAISKLDCGKRLGPDPDIFGSDH
yberc0001_6500   VTPARIRENFEVFDFKLHKDELTAISKLDSGKRLGPDPDVFGSDR
yrohd0001_6210   VTPARIRENFEVFDFKLHKDELTAISKLDSGKRLGPDPDVFGSDS
ykris0001_4750   VTPARIKENFEVFDFKLHKDELTAISKLDSGKRLGPDPDAPRA--
yente0001X_5860  VTPARIRENFEVFDFKLHKDELTAISKLDSGKRLGPDPDVFGSDR
yfred0001_41510  VTPVRIRENFEVFDFKLHKDELTAISKLDSGKRLGPDPDVFGSDS
                 ############################################
```

```
Parameters used
Minimum Number Of Sequences For A Conserved Position: 6
Minimum Number Of Sequences For A Flanking Position: 9
Maximum Number Of Contiguous Nonconserved Positions: 8
Minimum Length Of A Block: 10
Allowed Gap Positions: With Half
Use Similarity Matrices: Yes
```

```
Flank positions of the 1 selected block(s)
Flanks: [9  284]  

New number of positions in PGL1_unique_yersinia-CLUSTERS.dir/PGL1_unique_yersinia-CL1250/PGL1_unique_yersinia-CL1250.muscle.fasta.gblo:  276  (96% of the original 285 positions)
```
