# Supplementary material for: Genomic characterization of the Yersinia genus
Source: Genome Biol. 2010 Jan 4;11(1):R1. doi: 10.1186/gb-2010-11-1-r1 (PMC2847712; doi:10.1186/gb-2010-11-1-r1)
Supplement: Additional file 16 — The top level directory consists of a directory called Additional_cluster_files and 5010 directories, one for each multi-protein cluster family. (This top level directory has been split into three data files for uploading purposes (Additional files 15, 16, 17.) Within the directory are the following files: PGL1_unique_Yersinia_unclustered.out - list of all protein singletons that MCL did not group into a cluster (see Materials and Methods); PGL1_Yersinia_unique_locus_tags.txt - names of the 11 locus tag prefixes used for each genome; PGL1_unique_Yersinia.gff - mapping each Yersinia protein to a cluster in tab delimited GFF; PGL1_unique_Yersinia.sigfile - list of the longest protein in each cluster; PGL1_unique_Yersinia.summary - summary table of features of each of the clusters; PGL1_unique_Yersinia.table - summary table of each protein in the clusters. Within each cluster directory are the following files, where 'x' is the cluster name: PGL1_unique_Yersinia-x.faa - multifasta file of the proteins in the cluster; PGL1_unique_Yersinia-x.summary - summary of the properties of the proteins; PGL1_unique_Yersinia-x.matches - blast matches between the proteins of the cluster; PGL1_unique_Yersinia-x.muscle.fasta - muscle alignment of the proteins; PGL1_unique_Yersinia-x.muscle.fasta.gblo - gblocks output of muscle alignment (that is, auto-trimmed alignment); PGL1_unique_Yersinia-x.muscle.fasta.gblo.htm - as above in html format; PGL1_unique_Yersinia-x.muscle.tree - treefile from muscle alignment; PGL1_unique_Yersinia-x.sif - matches between proteins in simple interaction format for display on graphing software. [file gb-2010-11-1-r1-S16.zip › clusters2/PGL1_unique_yersinia-CL1251/PGL1_unique_yersinia-CL1251.muscle.fasta.gblo.htm]

PGL1\_unique\_yersinia-CL1251.muscle.fasta


## Gblocks 0.91b Results

Processed file: **PGL1\_unique\_yersinia-CL1251.muscle.fasta**  
Number of sequences: **11**  
Alignment assumed to be: **Protein**  
New number of positions: **385** (selected positions are underlined in blue)

```
                         10        20        30        40        50        60
                 =========+=========+=========+=========+=========+=========+
yruck0001_5050   ----------------------MQNFTLHTPTKVVFGAGQIAELANQIPADARVLITYGG
yaldo0001_6480   ----------------------MQNFTLHTPTKVLFGTGQIAQLSKEIPANARILITYGG
yente0001X_5870  ----------------------MQNFTLHTPTKVLFGTGQIAQLTKEIPADARILITYGG
yrohd0001_6220   ----------------------MQNFTLHTPTKILFGTGQIAQLNKEIPADARILITYGG
yfred0001_41530  ----------------------MQNFTLHTPTKILFGTGQIAQLSKEIPADARILITYGG
yinte0001_6600   ----------------------MQNFTLHTPTKVLFGTGQIAQLSKEIPADARILITYGG
ykris0001_4760   ----------------------MQNFTLHTPTKVLFGTGQIAQLSKEIPANARILITYGG
ypseu0001X_3770  ----------------------MQNFTLHTPTKVLFGTGQIAQLTQEIPADARILITYGG
ypest0001X_8830  ----------------------MQNFTLHTPTKVLFGTGQIAQLTQEIPADARILITYGG
yberc0001_6510   ---------MATNVINNDGIKIMQNFTLHTPTKVLFGTGQIAQLSKEIPADARILITYGG
ymoll0001_5820   LSYGAKLPTMADNKIDNTGRQIMQNFTLHTPTKVLFGTGQIAQLNKEIPADARILITYGG
                                       ######################################


                         70        80        90       100       110       120
                 =========+=========+=========+=========+=========+=========+
yruck0001_5050   GSVKKNGVMDQVHQALKNHQFEEFGGIEPNPSYETLMKAVEMIRASHFDFLLAVGGGSVL
yaldo0001_6480   GSIKQNGVLDQVHQALKGFDFLEFGGIEPNPTYETLMKAVEVCRTEKINFLLAVGGGSVL
yente0001X_5870  GSIKQNGVLDQVHQALKGFDFLEFGGIEPNPTYETLMKAVEVCRKENITFLLAVGGGSVL
yrohd0001_6220   GSIKQNGVLDQVHQALKGFDFLEFGGIEPNPTYETLMKAIEICRAENINFLLAVGGGSVL
yfred0001_41530  GSIKQNGVLDQVHQALKGFDFIEFGGIEPNPTYETLMQAIEVCRKEKINFLLAVGGGSVL
yinte0001_6600   GSIKQNGVLDQVHQALKGFNFLEFGGIEPNPTYETLMKAVALCRAEKIDFLLAVGGGSVL
ykris0001_4760   GSIKQNGVLDQVHQALKGFDYLEFGGIEPNPTYETLMKAVEVCRTEKITFLLAVGGGSVL
ypseu0001X_3770  GSIKQNGVLDQVHQALKGFDFLEFGGIEPNPTYETLMKAVELCRTEGINFLLAVGGGSVL
ypest0001X_8830  GSIKQNGVLDQVHQALKGFDFLEFGGIEPNPTYETLMKAVELCRTEGINFLLAVGGGSVL
yberc0001_6510   GSIKQNGVLDQVHNALKGFDFLEFGGIEPNPTYETLMKAVELIRAEKINFLLAVGGGSVL
ymoll0001_5820   GSIKQNGVLDQVHDALKGFDFLEFGGIEPNPTYETLMKAVELIRAEKINFLLAVGGGSVL
                 ############################################################


                        130       140       150       160       170       180
                 =========+=========+=========+=========+=========+=========+
yruck0001_5050   DGTKFIAAAVNYPEDPWQILETTGSKITQAIPMGSVLTLPATGSETNNGAVVSRRSTGDK
yaldo0001_6480   DGTKFIAAAVSYPQDPWHILETTGRDIKQALPMGSVLTLPATGSEANNGAVISRRSTGDK
yente0001X_5870  DGTKFIAAAVNYPQDPWHILETTGSDIKQALPMGSVLTLPATGSEANNGAVISRRSTGDK
yrohd0001_6220   DGTKFIAAGVNYPQDPWHILETTGSDITEAIPMGSVLTLPATGSEANNGAVISRRSTGDK
yfred0001_41530  DGTKFIAAGVNYPQDPWHILETMGRNITEAIPMGSVLTLPATGSEANNGAVISRRSTGDK
yinte0001_6600   DGTKFIAAAVNYPQEPWHILQTTGSDIKEALPMGSVLTLPATGSEANNGAVISRRETGDK
ykris0001_4760   DGTKFIAAAVNYPQDPWHILETTGSDIKEALPMGSVLTLPATGSEANSGAVISRRSTGDK
ypseu0001X_3770  DGTKFIAAAVNYPQDPWHILETTGRDITEALPMGSVLTLPATGSEANNGAVISRRSTGDK
ypest0001X_8830  DGTKFIAAAVNYPQDPWHILETTGRDITEALPMGSVLTLPATGSEANNGAVISRRSTGDK
yberc0001_6510   DGTKFIAAAVNYPQDPWHILETTGSDITEALPMGSVLTLPATGSETNSGAVISRRSTGDK
ymoll0001_5820   DGTKFIAAAVNYPQDPWHILETTGSDIKEALPMGSVLTLPATGSETNSGAVISRRSTGDK
                 ############################################################


                        190       200       210       220       230       240
                 =========+=========+=========+=========+=========+=========+
yruck0001_5050   RHFFSPFVQPLFAILDPVVTYTLPQRQVANGVVDAFVHTIEQYLTYPVNAKVQDRFAEGL
yaldo0001_6480   QHFFSPHVQPLFAVLDPVVTYTLPSRQVTNGVVDAFIHTIEQYLTYPVDAKVQDRFAEGL
yente0001X_5870  QHFFSAHVQPLFAVLDPEVTYALPPRQVANGVVDAFVHTIEQYLTYPVDAKVQDRFAEGL
yrohd0001_6220   QHFFSAHVQPLFAVLDPAVTYTLPPRQVANGVVDAFVHTLEQYLTYPVDAKVQDRFAEGL
yfred0001_41530  QHFFSPHVQPLFAVLDPEVTYTLPPRQVANGVVDAFVHTIEQYLTYPVDAKVQDRFAEGL
yinte0001_6600   QHFFSPFVQPLFAVLDPVVTYTLPPRQVANGVVDAFIHTIEQYLTYPVDAKVQDRFAEGL
ykris0001_4760   QHFFSPHVQPLFAVLDPEVTYTLPPRQVANGVVDAFVHTIEQYLTYPVDAKVQDRFAEGL
ypseu0001X_3770  QHFFSPHVQPLFAVLDPVVTYTLPPRQVANGVVDAFVHTIEQYLTYPVDAKVQDRFAEGL
ypest0001X_8830  QHFFSPHVQPLFAVLDPVVTYTLPPRQVANGVVDAFVHTIEQYLTYPVDAKVQDRFAEGL
yberc0001_6510   QHFFSPHVQPLFAVLDPVVTYSLPPRQVANGVVDAFVHTIEQYLTYPVDAKVQDRFAEGL
ymoll0001_5820   QHFFSPHVQPLFAVLDPVVTYSLPPRQVANGVVDAFVHTIEQYLTYPVDAKVQDRFAEGL
                 ############################################################


                        250       260       270       280       290       300
                 =========+=========+=========+=========+=========+=========+
yruck0001_5050   LLTLIEEGPKALENPEDYDVRANIMWSATMALNGLIGAGVPQDWATHMLGHELTALHGLD
yaldo0001_6480   LLTLIEEGPKALAEPENYNVRANIMWSATMALNGLIGAGVPQDWATHMLGHELTACHGLD
yente0001X_5870  LLTLIEDGPKALSDPKNYHVRANIMWSATMALNGLIGAGVPQDWATHMLGHELTARHGLD
yrohd0001_6220   LLTLIEDGPKALSDPKNYNVRANIMWSATMALNGLIGAGVPQDWATHMLGHELTARHGLD
yfred0001_41530  LLTLIEDGPKALSDPKNYNVRANIMWSATMALNGLIGAGVPQDWATHMLGHELTARHGLD
yinte0001_6600   LLTLIEEGPKALAEPENYNVRANIMWSATMALNGLIGAGVPQDWATHMLGHELTARHGLD
ykris0001_4760   LLTLIEEGPKALTDPENYNVRANIMWSATMALNGLIGAGVPQDWATHMLGHELTARHGLD
ypseu0001X_3770  LLTLIEEGPKALKDPENYNVRANIMWSATMALNGLIGAGVPQDWATHMLGHELTARHGLD
ypest0001X_8830  LLTLIEEGPKALKDPENYNVRANIMWSATMALNGLIGAGVPQDWATHMLGHELTARHGLD
yberc0001_6510   LLTLIEDGPKALTDPENYNVRANIMWSATMALNGLIGAGVPQDWATHMLGHELTARHGLD
ymoll0001_5820   LLTLIEDGPKALTDPENYDVRANIMWSATMALNGLIGAGVPQDWATHMLGHELTARHGLD
                 ############################################################


                        310       320       330       340       350       360
                 =========+=========+=========+=========+=========+=========+
yruck0001_5050   HAQTLAIVLPSLLEAKKEQKRAKLLQYAERVWNLHAGSEDQRIDAAIAATRDFFERMGVP
yaldo0001_6480   HAQTLAVVLPALLAAKKEQKRAKLLQYADRVWGLRDGSEEQRIEAAIEATRKFFEDMGIA
yente0001X_5870  HAQTLAVVLPALLMAKKQQKRAKLLQYAERVWGLQGGSEEQRINAAIQATRDFFESMGVA
yrohd0001_6220   HAQTLAVVLPALLVAKKQQKRAKLLQYAERVWGLREGTEEQRIDAAIEATRHFFESMGVA
yfred0001_41530  HAQTLAVVLPALLVAKKQQKHAKLLQYAERVWGLHEGTEEQRIDAAIEATRHFFESMGVA
yinte0001_6600   HAQTLAVVLPALLVAKKQQKRAKLLQYAERVWGLHDGSEDQRIDAAIEATRHFFESMGVA
ykris0001_4760   HAQTLAVVLPALLVAKKQQKRAKLLQYAERVWNIHEGTEDQRIDAAIEATRHFFESMGVA
ypseu0001X_3770  HAQTLAVVLPALLVAKKQQKRAKLLQYAERVWGLREGSEDQRIDAAIEATRHFFESLGVA
ypest0001X_8830  HAQTLAVVLPALLVAKKQQKRAKLLQYAERVWGLREGSEDQRIDAAIEATRHFFESLGVA
yberc0001_6510   HAQTLAVVLPALLVAKKEQKRAKLLQYAERVWGLREGSEEQRIDAAIEATRHFFESMGVA
ymoll0001_5820   HAQTLAVVLPALLVAKKEQKRAKLLQYAERVWGLREGSEEQRIDAAIEATRHFFESMGVA
                 ############################################################


                        370       380       390       400
                 =========+=========+=========+=========+=======
yruck0001_5050   THLSDYGLDGSTIPSLINKLDEHGYHHLGEHGDITLEESKRIYEAAI
yaldo0001_6480   TRLSAYGLDGSSIPHLIKKLEEHGMTQLGEHRDITLADSKRIYEAAV
yente0001X_5870  TRLSAYKLDGSTIPDLIKKLEEHGMTALGEHSDITLADSKRIYQAAV
yrohd0001_6220   TRLSAYQLDGSSIPDLIKKLEEHGMTALGEHSDITLADSKRIYEAAV
yfred0001_41530  THLSAYKLDGSSIPDLIKKLEEHGMTALGEHGDITLADSKRIYEAAV
yinte0001_6600   TRLSAYKLDGSSIPDLIKKLEEHGMTQLGEHSDITLADSKRIYEAAV
ykris0001_4760   TRLSDYKLDGSTIPDLIKKLEEHGMTALGEHSDITLADSKRIYEAAV
ypseu0001X_3770  TRLSAYKLDGSSIPDLIKKLEEHGMTKLGEHSDITLADSKRIYEAAV
ypest0001X_8830  TRLSAYKLDGSSIPDLIKKLEEHGMTKLGEHSDITLADSKRIYEAAV
yberc0001_6510   TRLSAYKLDGSSIPDLIKKLEEHGMTQLGEHGDITLADSKRIYEAAV
ymoll0001_5820   TRLSAYKLDGSSIPDLIKKLEEHGMTQLGEHGDITLADSKRIYEAAV
                 ###############################################
```

```
Parameters used
Minimum Number Of Sequences For A Conserved Position: 6
Minimum Number Of Sequences For A Flanking Position: 9
Maximum Number Of Contiguous Nonconserved Positions: 8
Minimum Length Of A Block: 10
Allowed Gap Positions: With Half
Use Similarity Matrices: Yes
```

```
Flank positions of the 1 selected block(s)
Flanks: [23  407]  

New number of positions in PGL1_unique_yersinia-CLUSTERS.dir/PGL1_unique_yersinia-CL1251/PGL1_unique_yersinia-CL1251.muscle.fasta.gblo:  385  (94% of the original 407 positions)
```
