# Supplementary material for: Genomic characterization of the Yersinia genus
Source: Genome Biol. 2010 Jan 4;11(1):R1. doi: 10.1186/gb-2010-11-1-r1 (PMC2847712; doi:10.1186/gb-2010-11-1-r1)
Supplement: Additional file 16 — The top level directory consists of a directory called Additional_cluster_files and 5010 directories, one for each multi-protein cluster family. (This top level directory has been split into three data files for uploading purposes (Additional files 15, 16, 17.) Within the directory are the following files: PGL1_unique_Yersinia_unclustered.out - list of all protein singletons that MCL did not group into a cluster (see Materials and Methods); PGL1_Yersinia_unique_locus_tags.txt - names of the 11 locus tag prefixes used for each genome; PGL1_unique_Yersinia.gff - mapping each Yersinia protein to a cluster in tab delimited GFF; PGL1_unique_Yersinia.sigfile - list of the longest protein in each cluster; PGL1_unique_Yersinia.summary - summary table of features of each of the clusters; PGL1_unique_Yersinia.table - summary table of each protein in the clusters. Within each cluster directory are the following files, where 'x' is the cluster name: PGL1_unique_Yersinia-x.faa - multifasta file of the proteins in the cluster; PGL1_unique_Yersinia-x.summary - summary of the properties of the proteins; PGL1_unique_Yersinia-x.matches - blast matches between the proteins of the cluster; PGL1_unique_Yersinia-x.muscle.fasta - muscle alignment of the proteins; PGL1_unique_Yersinia-x.muscle.fasta.gblo - gblocks output of muscle alignment (that is, auto-trimmed alignment); PGL1_unique_Yersinia-x.muscle.fasta.gblo.htm - as above in html format; PGL1_unique_Yersinia-x.muscle.tree - treefile from muscle alignment; PGL1_unique_Yersinia-x.sif - matches between proteins in simple interaction format for display on graphing software. [file gb-2010-11-1-r1-S16.zip › clusters2/PGL1_unique_yersinia-CL1252/PGL1_unique_yersinia-CL1252.muscle.fasta.gblo.htm]

PGL1\_unique\_yersinia-CL1252.muscle.fasta


## Gblocks 0.91b Results

Processed file: **PGL1\_unique\_yersinia-CL1252.muscle.fasta**  
Number of sequences: **11**  
Alignment assumed to be: **Protein**  
New number of positions: **418** (selected positions are underlined in blue)

```
                         10        20        30        40        50        60
                 =========+=========+=========+=========+=========+=========+
yruck0001_5450   MPRPLNDRSFALNADNLRALPARFGCPVWAYDGDIIAERINQLRHFDVIRFAQKACSNIH
yaldo0001_7290   MPRALNDTSTALTAQNLMALPARFGCPVWAYDGDVIAERINQLRQFDVIRFAQKACSNIH
ypseu0001X_3403  MPRALNDRSSALTAQNLIALPERFGCPVWAYDGDIIAERINQLRHFDVIRFAQKACSNIH
ypest0001X_9980  MPRALNDRSSALTAQNLIALPERFGCPVWAYDGDIIAERINQLRHFDVIRFAQKACSNIH
yinte0001_8340   MPRTLYDTTSALTAQNLMALPERFGCPVWAYDGEIIAQRINQLRNFDVIRFAQKACSNIH
yrohd0001_8350   MPRALDDTSTALTAKNLIALPERFGCPVWAYDGDIITRKINQLRNFDVIRFAQKACSNIH
yfred0001_43520  MPHALNDLSSALTAQNLIALPERFGCPVWAYDGDIITQKINQLRSFDVIRFAQKACSNIH
yberc0001_7060   MPRALNDTSSALTAQNLIALPERFGCPVWAYDGDIITQRINQLRHFDVIRFAQKACSNIH
ymoll0001_7640   MPRALNDTSSALTAQNLIALPERFGCPVWAYDGDIIAQRINQLRHFDVIRFAQKACSNIH
ykris0001_6020   MPRALNDISTALTAQNLIALPERFGCPVWAYDGEVITQKINQLRHFDVIRFAQKACSNIH
yente0001X_9220  MPRALNDTSSALTAQNLIALPERFGCPVWAYDGEIITQKINQLRHFDVIRFAQKACSNIH
                 ############################################################


                         70        80        90       100       110       120
                 =========+=========+=========+=========+=========+=========+
yruck0001_5450   ILRLMREQGVKVDSVSLGEIERALLAGYQPGKEPAEIVFTADVLDQATLERVVELGIPVN
yaldo0001_7290   ILRLMREQGVKVDSVSLGEIERAICAGFQPGQEPAEIVFTADLLDEATLSRVTALDIPVN
ypseu0001X_3403  ILRLMREQGVKVDSVSLGEIERALHAGYQPGQEPAEIVFTADLLDQATLLRVTELNIPVN
ypest0001X_9980  ILRLMREQGVKVDSVSLGEIERALHAGYQPGQEPAEIVFTADLLDQATLLRVTELNIPVN
yinte0001_8340   ILRLMREQGVKVDSVSLGEIERAIHAGFQPGQEPAEIVFTADLLDQATLLRVTELNIPVN
yrohd0001_8350   ILRLMREQGVKVDSVSLGEIERALHAGFQPGQEPAEIVFTADLLDQPTLLRVTELNIPVN
yfred0001_43520  ILGLMRNQGVKVDSVSLGEIERALQAGFQPGQEPAEIVFTADLLDQATLLRVTELHIPVN
yberc0001_7060   ILRLMREQGVKVDSVSLGEIERALIAGFQPGQEPAEIVFTADLLDQATLLRVTELAIPVN
ymoll0001_7640   ILRLMREQGVKVDSVSLGEIERALVAGFQPGQEPAEIVFTADLLDQATLLRVTELRIPVN
ykris0001_6020   ILRLMREQGVKVDSVSLGEIERALQAGFLPGQEPAEIVFTADLLDHATLLRVTELNIPVN
yente0001X_9220  ILRLMHEQGVKVDSVSLGEIERALQAGFQPGQEPAEIVFTADLLDQATLLRVTELSIPVN
                 ############################################################


                        130       140       150       160       170       180
                 =========+=========+=========+=========+=========+=========+
yruck0001_5450   AGSIDMLTQLGEQAPGHPVWLRVNPGFGHGHSQKTNTGGENSKHGIWHHDLAAAITEIGK
yaldo0001_7290   AGSIDMLDQLGQTKRGHPVWLRINPGFGHGHSQKTNTGGENSKHGIWYKDLPQAIEKVQQ
ypseu0001X_3403  AGSIDMLDQLGQQAPGHPVWLRVNPGFGHGHSQKTNTGGENSKHGIWHEELPRALKKIEH
ypest0001X_9980  AGSIDMLDQLGQQAPGHPVWLRVNPGFGHGHSQKTNTGGENSKHGIWHEELPRALKKIEH
yinte0001_8340   AGSIDMLDQLGQHAPGHPVWLRINPGFGHGHSQKTNTGGENSKHGIWHQDLPQAIEKVKK
yrohd0001_8350   AGSIDMLDQLGQHAPGHPVWLRVNPGFGHGHSQKTNTGGENSKHGIWHEDLPHAINKVKQ
yfred0001_43520  AGSIDMLEQLGQHAPGHPVWLRVNPGFGHGHSQKTNTGGENSKHGIWHEDLPLAIKKIEQ
yberc0001_7060   AGSIDMLDQLGQQAPGHPVWLRVNPGFGHGHSQKTNTGGENSKHGIWYQDLPQAIEKIAQ
ymoll0001_7640   AGSIDMLDQLGQHAPGHPVWLRVNPGFGHGHSQKTNTGGENSKHGIWYQDLPQAIEKIAQ
ykris0001_6020   AGSIDMLDQLGQHAPGHPVWLRVNPGFGHGHSQKTNTGGENSKHGIWHEDLTQAIEKIQQ
yente0001X_9220  AGSIDMLDQLGQHASGHPVWLRVNPGFGHGHSQKTNTGGENSKHGIWHEDLPQAIAKIQQ
                 ############################################################


                        190       200       210       220       230       240
                 =========+=========+=========+=========+=========+=========+
yruck0001_5450   YSLKLVGIHMHIGSGVDYQHLEQVCDAMVRQVITLGQDISAISAGGGLSIPYQNDE-DII
yaldo0001_7290   YGLTLIGIHMHIGSGVDYQHLEQVCDAMVQQVIALGQDISAISAGGGLSIPYQFGDADKI
ypseu0001X_3403  YGLTLVGIHMHIGSGVDYQHLEQVCDAMVQQVITLGHDISAISAGGGLSIPYQFGD-DVI
ypest0001X_9980  YGLTLVGIHMHIGSGVDYQHLEQVCDAMVQQVITLGHDISAISAGGGLSIPYQFGD-DVI
yinte0001_8340   YGLTLVGVHMHIGSGVDYQHLEQVCDAMVQQVIALGQDISAISAGGGLSIPYQFGD-DEI
yrohd0001_8350   YGLTLVGIHMHIGSGVDYQHLEQVCAAMVKQVIDLGQDISAISAGGGLSIPYQFGD-DEI
yfred0001_43520  YGLTLVGIHMHIGSGVDYQHLEQVCDAMVQQVIDLGQDISAISAGGGLSIPYQFGD-DEI
yberc0001_7060   YGLTLVGIHMHIGSGVDYQHLEQVCDAMVEQVVTLGQDISAISAGGGLSIPYQFGD-DEI
ymoll0001_7640   YGLTLVGIHMHIGSGVDYQHLEQVCDAMVEQVITLGQDISAISAGGGLSIPYQVGD-DEI
ykris0001_6020   YGLTLVGIHMHIGSGVDYQHLEQVCDAMVQQVIALGQDISAISAGGGLSIPYQFGD-DEI
yente0001X_9220  YGLKLVGIHMHIGSGVDYQHLEQVCDAMVQQVIALGQDISAISAGGGLSIPYQFGD-DEI
                 ######################################################## ###


                        250       260       270       280       290       300
                 =========+=========+=========+=========+=========+=========+
yruck0001_5450   DTEHYYGLWNKARQQIAMHLGHPVSLEIEPGRFLVAESGVLVAQVRAVKNMGSRHYVLVD
yaldo0001_7290   DTQHYYGLWNDARERIAAHLGHPVSLEIEPGRFLVAESGVLIAQVRAVKNMGNRHYVLVD
ypseu0001X_3403  DTEHYYGLWNSARERIAAHLGHPVSLEIEPGRFLVAESGVLIAQVRAVKDMGRRHYVLVD
ypest0001X_9980  DTEHYYGLWNSARERIAAHLGHPVSLEIEPGRFLVAESGVLIAQVRAVKDMGRRHYVLVD
yinte0001_8340   DTEHYYGLWNKAREQIAAHLGHPVSLEIEPGRFLVAESGVLVAQVRAVKDMGRRHYVLVD
yrohd0001_8350   DTEHYYGLWNHAREQIAAHLGHPVSLEIEPGRFLVAESGVLIAQVRAVKEMGSRHYVLVD
yfred0001_43520  DTEHYYGLWNSARERIAAHLGHPVSLEIEPGRFLMAESGVLIAQVRAVKDMGRRHYVLVD
yberc0001_7060   DTEHYYGLWNSARERIAAHLGHPVSLEIEPGRFLVAESGVLIAQVRAVKEMGSRHYVLVD
ymoll0001_7640   DTEHYYGLWNSARERIAAHLGHPVSLEIEPGRFLVAESGVLIAQVRAVKEMGSRHYVLVD
ykris0001_6020   DTEHYYGLWNRAREKIAAYLGHAVSLEIEPGRFLVAESGVLIAQVRAVKNMGSRHYVLVD
yente0001X_9220  DTEHYYGLWNSARERIAAHLGHPVSLEIEPGRFLVAEAGVLIAQVRAVKNMGRRHYVLVD
                 ############################################################


                        310       320       330       340       350       360
                 =========+=========+=========+=========+=========+=========+
yruck0001_5450   AGFNDLMRPAMYGSYHHISVLPADGRDLSHEPLIDSVIAGPLCESGDIFTQQAGGGLETR
yaldo0001_7290   AGFNDLMRPALYGSYHHISLLPADGRDLNSDPLIDTVIAGPLCESGDVFTQEAGGALETR
ypseu0001X_3403  AGFNDLMRPAMYGSYHHISLLPADGRDLASAPLIDTVVAGPLCESGDVFTQQEGGGVETL
ypest0001X_9980  AGFNDLMRPAMYGSYHHISLLPADGRDLTSAPLIDTVVAGPLCESGDVFTQQEGGGVETL
yinte0001_8340   AGFNDLMRPAMYGSYHHISLLPADGRDLTSAPLIDTVVGGPLCESGDVFTQEAGGGLETR
yrohd0001_8350   AGFNDLMRPAMYGSYHHISLLPADGRSLVAEPLIETVVAGPLCESGDVFTQEAGGGLETR
yfred0001_43520  AGFNDLMRPAMYGSYHHISLLPADGRSLADEPLIETVVAGPLCESGDVFTQEAGGGLETR
yberc0001_7060   AGFNDLMRPAMYGSYHHISLLPADGRSLSAEPLIETVVGGPLCESGDVFTQEAGGGLETR
ymoll0001_7640   AGFNDLMRPAMYGSYHHISLLPADGRSLTAEPLIETVVGGPLCESGDVFTQEAGGGLETR
ykris0001_6020   AGFNDLMRPAMYGSYHHISLLPADGRLLAGEPLIDTVVAGPLCESGDVFTQEAGGGLETR
yente0001X_9220  AGFNDLMRPAMYGSYHHISVLPANGRSLANEPLIDTVVAGPLCESGDVFTQEAGGGLETR
                 ############################################################


                        370       380       390       400       410       420
                 =========+=========+=========+=========+=========+=========+
yruck0001_5450   TLPRPVIGDYLVFHDTGAYGASMSSNYNSRPLLPEVLFENGHPRLIRRRQTIEELVALEM
yaldo0001_7290   QLPNAHIGDYLVFHDTGAYGASMSSNYNSRPLLPEVLFERGVPRLIRRRQTIEELIALEL
ypseu0001X_3403  ALPAAVIGDYLVFHDTGAYGASMSSNYNSRPLLPEVLFEKGQPRLIRRRQTIEELIDLER
ypest0001X_9980  ALPAAVIGDYLVFHDTGAYGASMSSNYNSRPLLPEVLFEKGQPRLIRRRQTIEELIDLER
yinte0001_8340   ALPAAKIGDYLVFHDTGAYGASMSSNYNSRPLLPEVLFEQGQPRLIRRRQTIEELIALEQ
yrohd0001_8350   ALPAAKIGDYVVFHDTGAYGASMSSNYNSRPLLPEVLFEQGQPRLIRRRQTIEELIALEQ
yfred0001_43520  ALPMAKIGDYLVFHDTGAYGASMSSNYNSRPLLPEVLFEQGQPRLIRRRQTIEELIALEQ
yberc0001_7060   PLPAARIGDYLVFHDTGAYGASMSSNYNSRPLLPEVLFEQGQPRLIRRRQTIEELIALEL
ymoll0001_7640   ALPAARIGDYLVFHDTGAYGASMSSNYNSRPLLPEVLFEQGQPRLIRRKQTIEELIALEL
ykris0001_6020   ALPAAKIGDYLVFHDTGAYGASMSSNYNSRPLLPEVLFEQGQPRLIRRRQTIEELIALEL
yente0001X_9220  KLPAANIGDYLVFHDTGAYGASMSSNYNSRPLLPEVLFEQGQPRLIRRRQTIEEIIALEL
                 ########################################################### 


                 
                 =
yruck0001_5450   F
yaldo0001_7290   V
ypseu0001X_3403  V
ypest0001X_9980  V
yinte0001_8340   I
yrohd0001_8350   F
yfred0001_43520  F
yberc0001_7060   V
ymoll0001_7640   V
ykris0001_6020   I
yente0001X_9220  I
```

```
Parameters used
Minimum Number Of Sequences For A Conserved Position: 6
Minimum Number Of Sequences For A Flanking Position: 9
Maximum Number Of Contiguous Nonconserved Positions: 8
Minimum Length Of A Block: 10
Allowed Gap Positions: With Half
Use Similarity Matrices: Yes
```

```
Flank positions of the 2 selected block(s)
Flanks: [1  236]  [238  419]  

New number of positions in PGL1_unique_yersinia-CLUSTERS.dir/PGL1_unique_yersinia-CL1252/PGL1_unique_yersinia-CL1252.muscle.fasta.gblo:  418  (99% of the original 421 positions)
```
