# Supplementary material for: Genomic characterization of the Yersinia genus
Source: Genome Biol. 2010 Jan 4;11(1):R1. doi: 10.1186/gb-2010-11-1-r1 (PMC2847712; doi:10.1186/gb-2010-11-1-r1)
Supplement: Additional file 16 — The top level directory consists of a directory called Additional_cluster_files and 5010 directories, one for each multi-protein cluster family. (This top level directory has been split into three data files for uploading purposes (Additional files 15, 16, 17.) Within the directory are the following files: PGL1_unique_Yersinia_unclustered.out - list of all protein singletons that MCL did not group into a cluster (see Materials and Methods); PGL1_Yersinia_unique_locus_tags.txt - names of the 11 locus tag prefixes used for each genome; PGL1_unique_Yersinia.gff - mapping each Yersinia protein to a cluster in tab delimited GFF; PGL1_unique_Yersinia.sigfile - list of the longest protein in each cluster; PGL1_unique_Yersinia.summary - summary table of features of each of the clusters; PGL1_unique_Yersinia.table - summary table of each protein in the clusters. Within each cluster directory are the following files, where 'x' is the cluster name: PGL1_unique_Yersinia-x.faa - multifasta file of the proteins in the cluster; PGL1_unique_Yersinia-x.summary - summary of the properties of the proteins; PGL1_unique_Yersinia-x.matches - blast matches between the proteins of the cluster; PGL1_unique_Yersinia-x.muscle.fasta - muscle alignment of the proteins; PGL1_unique_Yersinia-x.muscle.fasta.gblo - gblocks output of muscle alignment (that is, auto-trimmed alignment); PGL1_unique_Yersinia-x.muscle.fasta.gblo.htm - as above in html format; PGL1_unique_Yersinia-x.muscle.tree - treefile from muscle alignment; PGL1_unique_Yersinia-x.sif - matches between proteins in simple interaction format for display on graphing software. [file gb-2010-11-1-r1-S16.zip › clusters2/PGL1_unique_yersinia-CL1253/PGL1_unique_yersinia-CL1253.muscle.fasta.gblo.htm]

PGL1\_unique\_yersinia-CL1253.muscle.fasta


## Gblocks 0.91b Results

Processed file: **PGL1\_unique\_yersinia-CL1253.muscle.fasta**  
Number of sequences: **11**  
Alignment assumed to be: **Protein**  
New number of positions: **297** (selected positions are underlined in blue)

```
                         10        20        30        40        50        60
                 =========+=========+=========+=========+=========+=========+
yruck0001_5060   MAAIDDIQQLMAEQVARLAPGNGLTPSAVPRVNILFSDVHHPRTPVMYTPSIVIIFQGKK
ypseu0001X_3769  MVDVIDIQAQMADKIARLAQGDGLTPSTVSGVKILYSTVHQPRTPVMYTPSVVIIFQGHK
ypest0001X_8840  MVDVIDIQAQMADKIARLAQGDGLTPSTVSGVKILYSTVHQPRTPVMYTPSVVIIFQGHK
yaldo0001_6530   MVEANDIQAQMAAKITRLAKGNSVTPSAVPRVKVFYSTVHQPRTPVMYTPSVVIIFQGHK
yrohd0001_6230   MVEITETQSQMAGMVARLAQGNGLTPSAVPKVQILYSTVRQPRTPVMYTPSVVILFQGHK
yfred0001_41550  MVEVTDIQAQMAGKIAHLAQGNGLTPSAVPKVSILYSTVHQPRTPVMYTPSVVIIFQGHK
ykris0001_4770   MVEVTDIQAQMAGMIARLAQGNGLTPSAVPKVKILYSTVHQPRTPVMYTPSVVIIFQGHK
yente0001X_5900  MVEVTDIQAQMAGMIAGLAQGNGLTPSTVPKVKILYSTVHQPRTPVMYTPSVVIIFQGHK
yinte0001_6610   MVEVNDIQAQMAEKITHLAQGNGLTPSAVPRVKILYSTVHQPRTPVMYTPSVVIIFQGHK
ymoll0001_5830   MVEVNDIQALMAEKIAHLAQGNGLTPSAVPRVKILYSTVHQPRTPVMYTPSVVIIFQGHK
yberc0001_6520   MVEVNDIQVLLAKKISHLAQGNGLTPSAVPRVKILYSTTHQPRTPVMYTPSVVIIFQGHK
                 ############################################################


                         70        80        90       100       110       120
                 =========+=========+=========+=========+=========+=========+
yruck0001_5060   VGYLGSKIFQYDPKNYLLLTVPLPFECETFASPEKPLAGLSIHVDSQMLQDLLVDIGDDE
ypseu0001X_3769  VGYLGSKVFQYDPKNYLILSVPLPFECETFASPEIPLAGISIQVDSQMLQDLMMTMGDDE
ypest0001X_8840  VGYLGSKVFQYDPKNYLILSVPLPFECETFASPEIPLAGISIQVDSQMLQDLMMTMGDDE
yaldo0001_6530   VGYLGSKVFQYDPKNYLILTVPLPFECETFASPEIPLAGVSIHIDSQMLQDLLIDIGDDE
yrohd0001_6230   VGYLGSKVFQYDPKNYLILTVPLPFECETFASPEIPLAGISIHIDSQMLQDLIIDIGEDE
yfred0001_41550  IGYLGSKVFQYDPKNYLILTVPLPFECETFASPEIPLAGIYIHIDNQMLQDLIIDIGDDE
ykris0001_4770   VGYLGSKVFQYDPKNYLILTVPLPFECETFASPEVPLAGISIHIDSQMLQDLIIDIGDDE
yente0001X_5900  VGYLGSKVFQYDPTNYLILTVPLPFECETFASPEVPLAGISIHIDSQMLQDLIIDIGDDE
yinte0001_6610   VGYLGTKVFQYDPKNYLILTVPLPFECETFASPETPLAGISINIDSQMLQDLLIDIGDDE
ymoll0001_5830   VGYLGSKVFRYDPKNYLILTVPLPFECETFASPEIPLAGISIHIDSQMLQDLLIDIGDDE
yberc0001_6520   VGYLGSKVFRYDPKNYLILTVPLPFECETFASPEIPLAGISIHIDSQMLQDLLIDIGDDE
                 ############################################################


                        130       140       150       160       170       180
                 =========+=========+=========+=========+=========+=========+
yruck0001_5060   LDKPRGNTNGVNAALLTEEMLCATERLLDVMENPLAARVLGPQIVREILFYVLSGPCGAP
ypseu0001X_3769  LEKPPGNTSGVNSAPLTEEMLCATERLLDVMSIARDARVLGPQIVREILYYVLCGSCGGA
ypest0001X_8840  LEKPPGNTSGVNSAPLTEEMLCATERLLDVMSIARDARVLGPQIVREILYYVLCGSCGGA
yaldo0001_6530   VGKPLGNSSGVNSAALTNEMLCATERLLDVMSKPRDAKVLGPQIVREIIYYVLCGQCGGA
yrohd0001_6230   LEKPSGNTSGVNSSVLTEEMLCATERLLDVMLNPRDARVLGPQIVREIIYYVLCGQCGGA
yfred0001_41550  LDKPFSNTSGVNSSALTEEMLCATERLMDVMSNPRDARVLGPQIVREIIYYVLCGQCGGA
ykris0001_4770   LDKPFGNTSGVNSSALTEEMLCATERLLDVMANPRDARVLGPQIVREIIYYVLCGQCGGA
yente0001X_5900  LDKPFGNTSGVNSSALTEEMLCATERLLDVMSNPRDARVLGPQIVREIIYYVLCGQCGGA
yinte0001_6610   LDKPFGNTSGVNSSVLTEEMLCATERLLDVMSNPRDARVLGPQIVREILYYVLCGQCGGA
ymoll0001_5830   LDKPFGNASGVNSSVLTDEMLCATERLLDVMSTPRDARVLGPQIVREILYYVLCGQCGGA
yberc0001_6520   LDKPFGNSSGVNSSALTDEMLCATERLLDVMSKPRDARVLGPQIVREILYYVLCGQCGGA
                 ############################################################


                        190       200       210       220       230       240
                 =========+=========+=========+=========+=========+=========+
yruck0001_5060   LQELVNRHGHFNQIARALRRIENQFAENLNVEQLAAEVNMSISAFHHNFKAVTNTSPLQY
ypseu0001X_3769  LQALVNRHGHFNQITKTLRRIENHFADNLSVEQLAAEVNMSVSAFHHNFKAVTNTSPLQY
ypest0001X_8840  LQALVNRHGHFNQITKTLRRIENHFADNLSVEQLAAEVNMSVSAFHHNFKAVTNTSPLQY
yaldo0001_6530   LQALVNRHGHFNQITKTLRRIENHFADNLTVEQLAADVNMSISAFHHNFKAVTNTSPLQY
yrohd0001_6230   LEALVNRHGHFNQITKTLRRIENQFADNLNVEQLAADVNMSVSAFHHNFKAVTNTSPLQY
yfred0001_41550  LQALVNRHGHFNQITKTLRRIENQFAENLNVEQLAADVNMSISAFHHNFKAVTNTSPLQY
ykris0001_4770   LQALVNRHGHFNQITKTLRRIENQFADNLNVEQLAADVNMSVSAFHHNFKAVTNTSPLQY
yente0001X_5900  LQALVNRHGHFNQITKTLRRIENQFADNLNVEQLAADVNMSVSAFHHNFKAVTNTSPLQY
yinte0001_6610   LQALVNRHGHFNQITKTLRRIENQFADNLNVEQLAADVNMSVSAFHHNFKAVTNTSPLQY
ymoll0001_5830   LQALVNRHGHFNQITKTLRRIEHQFADNLNVEQLAADVNMSVSAFHHNFKAVTNTSPLQY
yberc0001_6520   LQALVNRHGHFNQITKTLRRIEHQFADNLNVEQLAADVNMSVSAFHHNFKAVTNTSPLQY
                 ############################################################


                        250       260       270       280       290
                 =========+=========+=========+=========+=========+=========
yruck0001_5060   LKSYRLHRARVLMVHDGLKASTAAIRVGYESASQFSREFKRYFGHTPGDEVAKLRSNPV
ypseu0001X_3769  LKSYRLHRARMMMLHDGLKASTAAIKVGYESASQFSREFKRYFGHTPSEGVAKQRIN--
ypest0001X_8840  LKSYRLHRARMMMLHDGLKASTAAIKVGYESASQFSREFKRYFGHTPSEGVAKQRIN--
yaldo0001_6530   LKSYRLHRARMMMLHDGLKASTAAIKVGYESASQFSREFKRYFGHTPSEEVAKLRTN--
yrohd0001_6230   LKSYRLHRARMMILHDGLKASTAANKVGYESASQFSREFKRYFGLTPSEEVAKLRAN--
yfred0001_41550  LKSYRLHRARMMMLHDGLKASTAANKVGYESASQFSREFKRYFGVTPSEELAKLRAG--
ykris0001_4770   LKSYRLHRARMMMLHDGLKASTAANKVGYESASQFSREFKRYFGLTPSEEVAKLRTN--
yente0001X_5900  LKSYRLHRARMMILHDGLKASTAANKVGYESASQFSREFKRYFGLTPSEEVAKLRTN--
yinte0001_6610   LKSYRLHRARMMMLHDGLKASTAANKVGYESASQFSREFKRYFGLTPSEEVAKLRTN--
ymoll0001_5830   LKTYRLHRARMMMLHDGLKASTAANKVGYESASQFSREFKRYFGHTPSEEVAKLRTN--
yberc0001_6520   LKSYRLHRARMMMLHDGLKASTAANRVGYESASQFSREFKRYFGHTPSEEVAKLRAN--
                 #########################################################
```

```
Parameters used
Minimum Number Of Sequences For A Conserved Position: 6
Minimum Number Of Sequences For A Flanking Position: 9
Maximum Number Of Contiguous Nonconserved Positions: 8
Minimum Length Of A Block: 10
Allowed Gap Positions: With Half
Use Similarity Matrices: Yes
```

```
Flank positions of the 1 selected block(s)
Flanks: [1  297]  

New number of positions in PGL1_unique_yersinia-CLUSTERS.dir/PGL1_unique_yersinia-CL1253/PGL1_unique_yersinia-CL1253.muscle.fasta.gblo:  297  (99% of the original 299 positions)
```
