# Supplementary material for: Genomic characterization of the Yersinia genus
Source: Genome Biol. 2010 Jan 4;11(1):R1. doi: 10.1186/gb-2010-11-1-r1 (PMC2847712; doi:10.1186/gb-2010-11-1-r1)
Supplement: Additional file 16 — The top level directory consists of a directory called Additional_cluster_files and 5010 directories, one for each multi-protein cluster family. (This top level directory has been split into three data files for uploading purposes (Additional files 15, 16, 17.) Within the directory are the following files: PGL1_unique_Yersinia_unclustered.out - list of all protein singletons that MCL did not group into a cluster (see Materials and Methods); PGL1_Yersinia_unique_locus_tags.txt - names of the 11 locus tag prefixes used for each genome; PGL1_unique_Yersinia.gff - mapping each Yersinia protein to a cluster in tab delimited GFF; PGL1_unique_Yersinia.sigfile - list of the longest protein in each cluster; PGL1_unique_Yersinia.summary - summary table of features of each of the clusters; PGL1_unique_Yersinia.table - summary table of each protein in the clusters. Within each cluster directory are the following files, where 'x' is the cluster name: PGL1_unique_Yersinia-x.faa - multifasta file of the proteins in the cluster; PGL1_unique_Yersinia-x.summary - summary of the properties of the proteins; PGL1_unique_Yersinia-x.matches - blast matches between the proteins of the cluster; PGL1_unique_Yersinia-x.muscle.fasta - muscle alignment of the proteins; PGL1_unique_Yersinia-x.muscle.fasta.gblo - gblocks output of muscle alignment (that is, auto-trimmed alignment); PGL1_unique_Yersinia-x.muscle.fasta.gblo.htm - as above in html format; PGL1_unique_Yersinia-x.muscle.tree - treefile from muscle alignment; PGL1_unique_Yersinia-x.sif - matches between proteins in simple interaction format for display on graphing software. [file gb-2010-11-1-r1-S16.zip › clusters2/PGL1_unique_yersinia-CL1255/PGL1_unique_yersinia-CL1255.muscle.fasta.gblo.htm]

PGL1\_unique\_yersinia-CL1255.muscle.fasta


## Gblocks 0.91b Results

Processed file: **PGL1\_unique\_yersinia-CL1255.muscle.fasta**  
Number of sequences: **11**  
Alignment assumed to be: **Protein**  
New number of positions: **822** (selected positions are underlined in blue)

```
                         10        20        30        40        50        60
                 =========+=========+=========+=========+=========+=========+
ykris0001_29830  -----------------------MSEHQSDENYVDKAITP--------------------
ypseu0001X_2178  ---------LLTQSLLVTHISSAADNNNQDDYIFDDALVRGSSLGLGSIARFNKKNSYDA
ypest0001X_2172  ---------LLTQSLLVTHISSAADNNNQDDYIFDDALVRGSSLGLGSIARFNKKNSYDA
yaldo0001_15910  VRFAPWLSCLLTHSLLVSHVVSAADEDNQDEYIFEDALVRGSALGLGAISRFNKKDSYEA
yinte0001_38650  --------------------VSAADQSNQDEYVFEDALVRGSSLGLGSISRFNKKNAYQE
yberc0001_16110  --------------LLVSYSLSAADEHHQDEYVFEDALLRGSSLGLGSISRFNKKNSYEA
ymoll0001_13170  --------------------VSAAEEHHQDEYVFEDALLRGSSLGLGSISRFNKKDSYEA
yrohd0001_15760  VKFSPGFLCLLTQGLFVSHNVFAADESNQDEYVFEDALLRGSSLGLGSISRFNKKDSYEA
yfred0001_16110  ----------LTHGLLVSHIVSAAEENNQDEYVFEDALLRGSSLGLGSISRFNKKDSYEA
ykris0001_14510  --------------------VSAADESKQDEYVFEDALLRGSSLGLGSISRFNKKDSYEA
yente0001X_1629  --------------------VSAADESKQDEYVFEDALLRGSSLGLGAISRFNKKDSYEA
                                      #######################################


                         70        80        90       100       110       120
                 =========+=========+=========+=========+=========+=========+
ykris0001_29830  -----------------EVDFTQLLNKLQPLLAGGRL----------------NNAVDML
ypseu0001X_2178  GQYQVDMYMNNKFVDRLKMLFVDKDNSVEPCLSVAQLLQAGVKEEALKTADPKTPCLAFQ
ypest0001X_2172  GQYQVDMYMNNKFVDRLKMLFVDKDNSVEPCLSVAQLLQAGVKEEALKTADPKTPCLAFQ
yaldo0001_15910  GKYQVDLYMNNKFIDRVALDFIEKNDAVVPCLSVALLLQAGVTETALKDADPQDQCLPFK
yinte0001_38650  GKYQVDLYINNKFVDRVEIEFIAKNNDVIPCLSGAQLLQAGVTEEALKNSDPKDNCLDFR
yberc0001_16110  GKYQVDLYMNNKFVDRVELTFITKGDDVVPCLSGAQLLQAGVEESALKQANLEDNCLDLK
ymoll0001_13170  GKYQVDLYMNNKFVDRVELTFIPKGDDVIPCLSGAQLLQAGVEETALKNAHLEDNCLDFK
yrohd0001_15760  GKYQVDLFMNNSFIDRVELLFITKDGAVVPCLSLSQLLQAGVTDKALENVSGEDNCLDFK
yfred0001_16110  GKYHVDLFMNNTFVDRVELMFITKGDTVIPCLSVSQMLQAGVREDALEKVDHNDNCLDFK
ykris0001_14510  GKYQVDIYMNSKFVDRIELMFITKGDAVVPCLSVSQLLQAGVNENALENAKLEDNCLDFK
yente0001X_1629  GKYQVDLYMNNKFVDRIELIFITKDDAVVPCLSASQLLQAGVNDSVLKNANQEDDCLDFK
                 ############################################################


                        130       140       150       160       170       180
                 =========+=========+=========+=========+=========+=========+
ykris0001_29830  SLLS--------------------------------------------------------
ypseu0001X_2178  SILPASDFRFDHAKLRFDLSIPQKFVKNVPRGYVDPKNLTAGNTIGFSNYNLNQYHVDYN
ypest0001X_2172  SILPASDFRFDHAKLRFDLSIPQKFVKNVPRGYVDPKNLTAGNTIGFSNYNLNQYHVDYN
yaldo0001_15910  DILPASNYRFDNAKLRFDLSVPQLFVKKIPRGYVDPRNLTYGNTIGFSNYNLNQYHVGYN
yinte0001_38650  TILPASDYRFDYAKLRFDLSVPHLFVKKVPRGYVDPLNLTSGDTIGFSNYNLNQYHVGYN
yberc0001_16110  TILPTSDYHFDYAKLRFDLSVPQLFVKKMPRGYVDPRNLTSGETIGFSNYNFNQYHVGYN
ymoll0001_13170  TILPASDYHFDYAKLRFDLSVPQLFVKKMPRGYVDPRNLTSGDTIGFSNYNFNQYHVGYN
yrohd0001_15760  TILPASDYHFDYAKLRFDLSVPQLFVKNIPRGYVDPRNLTAGETIGFSNYNLNQYHVSYN
yfred0001_16110  TLLPASDYRFDYAKLRFDLSIPQLFVKNVPRGYVDPRNLTAGETIGFSNYNLNQYHVGYN
ykris0001_14510  TLLPASDYHFDYAKLRFDLSVPQLFVKHVPRGYVDPRNLTAGETIGFSNYNLNQYHVSYN
yente0001X_1629  TLLPASDYRFDYAKLRFDLSIPQLYVKNVPRGYVDPRNLTAGETIGFSNYNLNQYHVGYN
                 ############################################################


                        190       200       210       220       230       240
                 =========+=========+=========+=========+=========+=========+
ykris0001_29830  ------------------------------------------------------------
ypseu0001X_2178  KEGIKRTTNSTYLSLNSGINIGMWRFRQQGSLRYDASRGTNWTSNRLYSQRALPTIGSEI
ypest0001X_2172  KEGIKRTTNSTYLSLNSGINIGMWRFRQQGSLRYDASRGTNWTSNRLYSQRALPTIGSEI
yaldo0001_15910  KDGIKRVTDSTYLSLTNGINVGMWRFRQQGSLRYDPTRGANWTSNRFYSQRALPAIGSEV
yinte0001_38650  KDGIKRSTNSTYLSLTNGINAGMWRLRQQGSLRYDPTRGTNWTSNRLYSQRALPTMGSEV
yberc0001_16110  KDGIKRATDSSYLSLTNGINAGMWRFRQQGSLRYDQTRGANWTSNRLYSQRALPSIGSEV
ymoll0001_13170  KDGIKRATDSSYLSLTNGINAGMWRFRQQGSLRYDQTRGANWTSNRLYSQRALPSIGSEV
yrohd0001_15760  KDGIKRDTDSSYLSLNNGINAGMWRFRQQGSLRYDSTRGANWTSNRFYSQRALPSIGSEV
yfred0001_16110  KDGIKRTTNSSYLSLNNGINVGMWRFRQQGSLRYDDTRGANWTSNRLYSQRALPTIGSEI
ykris0001_14510  KEGIKRSTNSSYLSLNNGINAGMWRFRQQGSLRYDATRGADWTSNRLYSQRALPSIGSEI
yente0001X_1629  KDGIKRSTDSTYLSLNNGINAGMWRFRQQGSLRYDPTRGANWTSNRLYSQRALPSIGSEV
                 ############################################################


                        250       260       270       280       290       300
                 =========+=========+=========+=========+=========+=========+
ykris0001_29830  ------------------------------------------------------------
ypseu0001X_2178  TLGETFSSGQFFSSLGFLGVALSTDDRMLPESQRGYAPVVRGIARTNARVTVYQNNRSIY
ypest0001X_2172  TLGETFSSGQFFSSLGFLGVALSTDDRMLPESQRGYAPVVRGIARTNARVMVYQNNRSIY
yaldo0001_15910  TMGETFSAGQFFSSLGFSGVALSTDDRMLPESQRGYAPIVRGIARTNAKVTVYQNNRSIY
yinte0001_38650  TLGQTFSSGQFFSSLGFNGIALATDDRMLPESQRGYAPIVRGIARTNAKVTVYQNNRPIY
yberc0001_16110  TAGETFSAGQFFSSMGFTGIALATDDRMLPESQRGYAPVVRGIARTNAKVTVYQNNRSIY
ymoll0001_13170  TAGETFSSGQFFSSMGFTGIALATDDRMLPESQRGYAPVVRGIARTNAKVTVYQNSRPIY
yrohd0001_15760  TLGETFSSGQFFSSLGFSGVALSTDDRMLPESLRGYAPVVRGIARTNANVTVYQNNRAIY
yfred0001_16110  TVGETFSSGQFFSSLGFSGVALATDDRMLPESQRGYAPIVRGIARTNAKVTVYQNNRSIY
ykris0001_14510  TLGETFSSGQFFSSLGFSGVALSTDDRMLPESQRGYAPVVRGIARTNAKVTVYQNNRSIY
yente0001X_1629  TVGETFSSGQFFSSLGFSGIALSTDDRMLPESQRGYAPVIRGIARTNAKVTVYQNNRPIY
                 ############################################################


                        310       320       330       340       350       360
                 =========+=========+=========+=========+=========+=========+
ykris0001_29830  ------------------------------------------------------------
ypseu0001X_2178  QTTVSPGAFEFNDLSVTHFGGDLTVEINEADGSVSTFQVPFASVPESLRPGYSRYSFAAG
ypest0001X_2172  QTTVSPGAFEFNDLSVTHFGGDLTVEINEADGSVSTFQVPFASVPESLRPGYSRYSFAAG
yaldo0001_15910  QTTVSPGPFEFNDLSATNFGGDLTVEIQEADGNLSTFQVPFSSVPESLRPGYSRYSFAAG
yinte0001_38650  QTTVSPGPFEFNDLSATNFGGDLTIEIQEADGSVSTFQVPFSSVPESLRPGYSRYSFAAG
yberc0001_16110  QATVSPGPFEFDDLSATNFGGDLTVEIQEADGSLSTFQVPFSSVPESLRPGYSRYSFAAG
ymoll0001_13170  QATVSPGPFEFDDLSATNFGGDLTVEIQEADGSLSTFQVPFSSVPESLRPGYSRYSFAAG
yrohd0001_15760  QSTVSPGPFEFTDLSATNFGGDLTVEIQEADGSISTFQVPFSSVPESLRPGYSRYSFAAG
yfred0001_16110  QSTVSPGPFEFNDLSATNFGGDLTVEIQEADGSLSTFQVPFASVPESLRPGYSRYSFAAG
ykris0001_14510  QSTVSPGPFEFNDLSATNFGGDLTVEIQEADGSISTFQVPFSSVPESLRPGYSRYNFATG
yente0001X_1629  QSTVSPGAFEFNDLTATNFGGDLTVEIQEADGTLSTFQVPFSSVPESLRPGYSRYSFAAG
                 ############################################################


                        370       380       390       400       410       420
                 =========+=========+=========+=========+=========+=========+
ykris0001_29830  DVVDIADNALVEKLA---------------------------GVFESIV-AVG-------
ypseu0001X_2178  QVRDVGNNETFSELTYQQGISNAITANTGIRLASGYQAIMLGGVFTHYIGALGLNTTYSH
ypest0001X_2172  QVRDVGNNETFSELTYQQGISNAITANTGIRLASGYQAIMLGGVFTHYIGALGLNTTYSH
yaldo0001_15910  QVRDLGSNETFTELTYQRGISNAITGNTGVRLASGYQAVMLGGVYTHYIGALGLDTTYSH
yinte0001_38650  QVRDLNSSEVFSELTYQRGISNAITANTGLRMASGYQAIMLGGVFTHYIGALGLDATYSN
yberc0001_16110  KVRDQGNHELFSELTYQRGVSNAITANSGLRLAPGYQALMLGGVFTHYIGALGLDVTYSK
ymoll0001_13170  KVRDQGNHEVFSELTYQRGVSNAITANTGLRLAPGYQAAMLGGVFTHYIGALGLDVTYSK
yrohd0001_15760  QVRDIASHEMFGELTYQRGLSNAITANGGVRLASGYQAMMLGGVFTHYIGALGLDATYSN
yfred0001_16110  QVRDLASHEVFSELTYQRGISNAITANSGLRLASGYQAVMLGGVFTHYIGALGLDATYSD
ykris0001_14510  QVRDMGSHEVFSELTYQRGVSNAITANTGVRIASGYQAVMLGGVFTHYIGALGLDATYSH
yente0001X_1629  QVRDLSSQEMFGELTYQQGVSNAITANSGMRLASGYQAVMLGGVFTHYIGALGLDATYSH
                 ############################################################


                        430       440       450       460       470       480
                 =========+=========+=========+=========+=========+=========+
ykris0001_29830  ------------------------------------------------------------
ypseu0001X_2178  ARL------PDGEQ------QQGWMAKASFSRTFQPTNTTLSVAGYRYSTDGYRDLSDVL
ypest0001X_2172  ARL------PDGEQ------QQGWMAKASFSRTFQPTNTTLSVAGYRYSTDGYRDLSDVL
yaldo0001_15910  ATL------PEDDD------QSGWMARISFSRTFEPTNTTLSVAGYRYSTDGYRDLSDVL
yinte0001_38650  ARL------SDSND-SASERQSGWMARVSFSRMFETTNTTLSVAGYRYSTEGYRDLSDVL
yberc0001_16110  ASL------PDDEQ------QSGWMARAAFSRTFEATNTTLSVAGYRYSTEGYRDLSDVL
ymoll0001_13170  ASL------PDDEQ------QSGWMARAAFSRTFEATNTTLSVAGYRYSTEGYRDLSDVL
yrohd0001_15760  ARL------LDDEQ------QHGWMARASFSRTFSATNTTLSVAGYRYSTEGYRDLSDVL
yfred0001_16110  ARL------PNDEQ------QNGWMARASFSRRFETTDTTLSVAGYRYSTEGYRDLSDVL
ykris0001_14510  ASLPGNANSPDNSDSSNSTKQNGWMARASFSRTFEATNTTLSVAGYRYSTEGYRDLSDVL
yente0001X_1629  ASL------PSDTHSSENKSQNGWMARASFSRTFETTNTTLSVAGYRYSTEGYRDLSDVL
                 ###                 ########################################


                        490       500       510       520       530       540
                 =========+=========+=========+=========+=========+=========+
ykris0001_29830  ----------WESGNALRIANTELRLNQEAVNYRSLY-----------------------
ypseu0001X_2178  GVRATSNDSSWNSSTYRQRSRAEISLNQNFHRYGSLYLTASSQDYRDDRSRDSQLQLGYS
ypest0001X_2172  GVRATSNDSSWNSSTYRQRSRAEISLNQNFHRYGSLYLTASSQDYRDDRSRDSQLQLGYS
yaldo0001_15910  GMRATSDGKVWSSGTYRQRSRTEISLNQNLNSYGALYLTASSQDYRDDRKRDTQLQLGYA
yinte0001_38650  GVRAANNGKVWNSDTYRQRSRAEISLNQNLNSYGSLYLTASSQDYRNDRKRDSQLQLGYA
yberc0001_16110  GVRAATQGKVWSSGSYQQRSRAEISLNQNFNRYGSLYLTASSQDYRSDRKRDTQLQLGYA
ymoll0001_13170  GVRAANNGKVWSSGSYQQRSRAEISLNQSFNRYGSLYLTASSQDYRNDRKRDTQLQLGYA
yrohd0001_15760  GVRAASNGKVWNSGTYQQRSRAEISLNQNFNNYGSLYLTASSQDYRNARKRDTQLQLGYA
yfred0001_16110  GMRAANSGKVWSSGTYKQRSRAEISLNQNFNSYGSLYLTASSQDYRNDHKRDTQLQLGYA
ykris0001_14510  GIRAASHGKVWSSGTYQQRSRAEISLNQNLNNYGSLYLTASSQDYRNSRKRDTQLQLGYA
yente0001X_1629  GIRAASNGKVWSSGTYQQRSRAEISLNQNLNSYGSLYLTASSQDYRNSRERDTQLQLGYA
                 ############################################################


                        550       560       570       580       590       600
                 =========+=========+=========+=========+=========+=========+
ykris0001_29830  -SLFRD---------------------------------------RDTLV----------
ypseu0001X_2178  NTFWRNTSFNLAISQQKTGGA-NKIYFVDPGSGMPASNGANTLATRETVAQMSISFPLGG
ypest0001X_2172  NTFWRNTSFNLAISQQKTGGA-NKIYFVDPGSGMPASNGANTLATRETVAQMSISFPLGG
yaldo0001_15910  NTLWRNTSFNLAVSKQKTGGGNNETYFVDPGSGMPAANGANLLGSSETLVQMSISFPLGG
yinte0001_38650  NTLWRDTSLNIAVSRQKTGGVSNETYFVDPGSGMPAANGANILGTSETVVQMSVSFPLGG
yberc0001_16110  NTLWRDTSFNLAVSQQKTGGGSNEVYFVDPGSGMPAANGANTLGVSETLVQISISFPLGG
ymoll0001_13170  NTLWRDTSFNLAVSQQKTGGGSNEIYFVDPGSGMPAANGAKTLGVSETLVQMSISFPLGS
yrohd0001_15760  NTLWHNTSFNLSVSQQKTGGG-NETYFIDPGSGMPAANGASLLAINETVVQLAISFPLGG
yfred0001_16110  NTLWHNTSFNLAVSQQKTGGI-NETYFVDPGSGMPAANGAKILATNETVVQMSISFPLGG
ykris0001_14510  NTLWRNTSVNLAVSQQKTGGGNNETYFVDPGSGMPAANGANLLATNETVVQMSISFPLGG
yente0001X_1629  NTLWRNTSFNLAISKQKTGRGTNETYFVDPGSGMPAANGANFLATNETVVQMSVSFPLGG
                 ############################################################


                        610       620       630       640       650       660
                 =========+=========+=========+=========+=========+=========+
ykris0001_29830  ------------------------------------------------------------
ypseu0001X_2178  SSSAPYVSAGAVNSRTSGASYQTSLSGTMGSDQTAGYSVDVARNEPTNENTLSGSLQKQL
ypest0001X_2172  SSSAPYVSAGAVNSRTSGASYQTSLSGTMGSDQTAGYSVDVARNEPTNENTLSGSLQKQL
yaldo0001_15910  SPQAPYVSAGAVNSKTSGASYQTSLSGVMGADQSSSYSMDFARSEQTKENTFSGSLQKRL
yinte0001_38650  SAHAPYISAGAVNSKISGASYQTSLSGVMGEEQSASYSMDFARSEQTKENTFSGSLQKRL
yberc0001_16110  SPRAPYISAGAVNSQASGASYQTSLSGVMGDDQSASYSMDFARSEQNKENTFSGSLQKRL
ymoll0001_13170  SPRAPYISAGAVNSQTSGASYQTSLSGVMGDDQSASYSMDFARSEQTKENTFSGSLQKRL
yrohd0001_15760  SPQAPYVSAGAINSKASGASYQTSLSGVMGDDQSASYSMDFARSEQTKDNTFSGSLQKRL
yfred0001_16110  SPQAPYVSAGAVNSKVSGASYQTSLSGVMGDDQSASYSMDFARSEQTKENTFSGSLQKRL
ykris0001_14510  SPQAPYISAGAVNSRVSGASYQTSLAGVMGDDQSSSYSMDFARSEQTKENTFSGSLQKRL
yente0001X_1629  NPQAPYVSAGAVNSRISGASYQTSLAGVMGDDQSASYSMDFARSEQSKENTFSGSLQKRL
                 ############################################################


                        670       680       690       700       710       720
                 =========+=========+=========+=========+=========+=========+
ykris0001_29830  -------------------------------GITLL------------------------
ypseu0001X_2178  PTTSLSGSASRSPGYWQGSASARGSVAFHRGGVTLGPYLSDTFALIEAKGASGAKVMYGQ
ypest0001X_2172  PTTSLSGSASRSPGYWQGSASARGSVAFHRGGVTLGPYLSDTFALIEAKGASGAKVMYGQ
yaldo0001_15910  PATSLSGSLSRSPGYWQGSASARGAVAFHSGGVTLGPYLSDTFALIEAKGASGAKVMYGQ
yinte0001_38650  PSTSLSGSASSSPGYWQGSASARGAVAFHSGGVTLGPYLSDTFALIEAKGASGAKVMYGQ
yberc0001_16110  PVTSLSGSASSSPGYWQGSASARGSAAVHSGGITLGPYLSDTFALIEAKGASGAKVMYGQ
ymoll0001_13170  PVTSLSGSASSSPGYWQGSASARGSAAFHSGGVTLGPYLSDTFALIEAKGASGAKVMYGQ
yrohd0001_15760  PTTSLSGSASRSPGYWQGSASARGAIAFHSGGVTLGPYLSDTFALVEAKGASGAKVMYGQ
yfred0001_16110  PTTSLSGSASRSPGYWQGSASARGSVAFHRGGITLGPYLSDTFALVEAKGASGAKVMYGQ
ykris0001_14510  PSTSLSSSASRSPGYWQGSASARGAVAFHSGGVTLGPYLSDTFALIEAKGASGAKVMYGQ
yente0001X_1629  PVTSLSSSASLSPGYWQSSASTRGAIAFHRGGVTLGPYLSDTFALIEAKGASGAKVMYGQ
                 ############################################################


                        730       740       750       760       770       780
                 =========+=========+=========+=========+=========+=========+
ykris0001_29830  ------------------------------------------------------LRTLQ-
ypseu0001X_2178  GARIDRFGYALVPTLTPYRYNTLSLDPDGMDFNTELQDGERQIAPYAGSTVKVTFRTLNG
ypest0001X_2172  GARIDRFGYALVPTLTPYRYNTLSLDPDGMDFNTELQDGERQIAPYAGSTVKVTFRTLNG
yaldo0001_15910  GATIDRFGYALVPTLTPYRYNTITLDPDGMDFNTELQDGERQIAPYAGSSVKVTFRTLSG
yinte0001_38650  GSSIDRFGYALVPTLTPYRYNTITLSPDGMDFNTELQDGERQIAPYAGSSVKVIFRTLSG
yberc0001_16110  GARIDHFGYALVPTLTPYRYNTITLDPDGMDFNTELRDGERQIAPYAGSTVKVTFRTLSG
ymoll0001_13170  GARIDHFGYALVPTLTPYRYNTITLDPEGMDFNTELRDGERQIAPYAGSTVKVTFRTLSG
yrohd0001_15760  GAKIDSFGYALVPTLTPYRYNTITLNPDGMDFNTELQDAERQIAPYAGSAVKVTFRTLSG
yfred0001_16110  GAKIDSNGYALVPTLTPYRYNTITLDPDGMDFNTELQDGERQIAPYAGSATKVTFRTLSG
ykris0001_14510  GAKIDHFGYALVPTLTPYRYNTITLDPDGMDFNTELQDGERQIAPYAGSAVKVKFRTLSG
yente0001X_1629  GAKIDGFGYALVPTLTPYRYNTITLDPDGMDFNTELQDGERQIAPYAGSAVKVRFRTLSG
                 ############################################################


                        790       800       810       820       830       840
                 =========+=========+=========+=========+=========+=========+
ykris0001_29830  ------------------------------------------IIGQRINA----------
ypseu0001X_2178  YPALITIKMPDGSQLPMGTVVYNYNGK-----GTNDKNDIVGMVGQSSQAYLRAEELSGT
ypest0001X_2172  YPALITIKMPDGSQLPMGTVVYNYNGK-----GTNDKNDIVGMVGQSSQAYLRAEELSGT
yaldo0001_15910  YPVLITVKLADGSQLPMGTVVYSYKNG-----SNADKGSEIGMVGQGSQAYLRAPETNGT
yinte0001_38650  YPLLITVRLADGSQLPLGAVVYSTTTESG---KAAQQQNEVGMVGQASQAYVRAEGASGT
yberc0001_16110  YPVLINVKLADGSQLPMGTVIYSTNGSADSGHDDSKQEHEVGMVGQASQAYLRAASPSGT
ymoll0001_13170  YPVLINVKLANGGQLPMGTVVYSTDGSTESGHDDRSQAHEVGMVGQASQAYLRAENPRGT
yrohd0001_15760  YPLLINVKLADGSQLPMGAVVYNAGQKAD---GDNNQHQEVGMVGQGSQAYLRAEKLRGT
yfred0001_16110  YPLLITVKLADGGQLPMGTMVYRSESQADNDNGDNHQNREVGMVGQASQAYLRAEKTRGT
ykris0001_14510  YPLLVTVRLSDGSQVPMGAVVYSTGGTTDNKNGDTSPNLEIGMVGQASQAYLRAEHASGT
yente0001X_1629  YPLLISVKLADGSQVPMGAVVYTTSGPANNQDDNNRQDREVGMVGQASQAYLRAEQARGT
                 ######################                  ####################


                        850       860       870       880
                 =========+=========+=========+=========+=
ykris0001_29830  ----------------TKISPP-------------------
ypseu0001X_2178  LTLVWGESSKERCQLDYDLGKPTDNDKQLYKLDALCVAVQQ
ypest0001X_2172  LTLVWGESSKERCQLDYDLGKPTDNDKQLYKLDALCVAVQQ
yaldo0001_15910  LLLVWGESANERCQLNYSLGKP-DNDKQLYKLNALCVITQH
yinte0001_38650  LILVWGDAANERCQLDYDLGTP-NNDKQLYKLDALCVVTQH
yberc0001_16110  LMLVWGDAADERCQLDYNLGTP-NNDQQLYKLDALCVVTQP
ymoll0001_13170  LILAWGDAADERCKLDYDLGTP-KNDQQLYKLDALCVVTQT
yrohd0001_15760  LMLVWGESANERCQLDYDLGKP-DNDRQLYKLDALCVVSQH
yfred0001_16110  LLLVWGDAPNERCHLDYDLGKP-NNDRELYRLDALCVVTQH
ykris0001_14510  LLLVWGDAANESCQLDYDLGTP-NNDKQLYKLDALCVVAQH
yente0001X_1629  LLLVWGDTDNERCQLDYDLGTP-NKDKQLYKLDALCVVTQH
                 ######################  ################
```

```
Parameters used
Minimum Number Of Sequences For A Conserved Position: 6
Minimum Number Of Sequences For A Flanking Position: 9
Maximum Number Of Contiguous Nonconserved Positions: 8
Minimum Length Of A Block: 10
Allowed Gap Positions: With Half
Use Similarity Matrices: Yes
```

```
Flank positions of the 4 selected block(s)
Flanks: [22  423]  [441  802]  [821  862]  [865  880]  

New number of positions in PGL1_unique_yersinia-CLUSTERS.dir/PGL1_unique_yersinia-CL1255/PGL1_unique_yersinia-CL1255.muscle.fasta.gblo:  822  (93% of the original 881 positions)
```
