# Supplementary material for: Genomic characterization of the Yersinia genus
Source: Genome Biol. 2010 Jan 4;11(1):R1. doi: 10.1186/gb-2010-11-1-r1 (PMC2847712; doi:10.1186/gb-2010-11-1-r1)
Supplement: Additional file 16 — The top level directory consists of a directory called Additional_cluster_files and 5010 directories, one for each multi-protein cluster family. (This top level directory has been split into three data files for uploading purposes (Additional files 15, 16, 17.) Within the directory are the following files: PGL1_unique_Yersinia_unclustered.out - list of all protein singletons that MCL did not group into a cluster (see Materials and Methods); PGL1_Yersinia_unique_locus_tags.txt - names of the 11 locus tag prefixes used for each genome; PGL1_unique_Yersinia.gff - mapping each Yersinia protein to a cluster in tab delimited GFF; PGL1_unique_Yersinia.sigfile - list of the longest protein in each cluster; PGL1_unique_Yersinia.summary - summary table of features of each of the clusters; PGL1_unique_Yersinia.table - summary table of each protein in the clusters. Within each cluster directory are the following files, where 'x' is the cluster name: PGL1_unique_Yersinia-x.faa - multifasta file of the proteins in the cluster; PGL1_unique_Yersinia-x.summary - summary of the properties of the proteins; PGL1_unique_Yersinia-x.matches - blast matches between the proteins of the cluster; PGL1_unique_Yersinia-x.muscle.fasta - muscle alignment of the proteins; PGL1_unique_Yersinia-x.muscle.fasta.gblo - gblocks output of muscle alignment (that is, auto-trimmed alignment); PGL1_unique_Yersinia-x.muscle.fasta.gblo.htm - as above in html format; PGL1_unique_Yersinia-x.muscle.tree - treefile from muscle alignment; PGL1_unique_Yersinia-x.sif - matches between proteins in simple interaction format for display on graphing software. [file gb-2010-11-1-r1-S16.zip › clusters2/PGL1_unique_yersinia-CL1256/PGL1_unique_yersinia-CL1256.muscle.fasta.gblo.htm]

PGL1\_unique\_yersinia-CL1256.muscle.fasta


## Gblocks 0.91b Results

Processed file: **PGL1\_unique\_yersinia-CL1256.muscle.fasta**  
Number of sequences: **11**  
Alignment assumed to be: **Protein**  
New number of positions: **359** (selected positions are underlined in blue)

```
                         10        20        30        40        50        60
                 =========+=========+=========+=========+=========+=========+
yruck0001_5190   --------------------------MAHFTNGDTMQLIINSNLISKKEVNTKKTVSLIS
ypseu0001X_3757  MTTGIKIMMFMGVLFIKIDITPLSSHGIKIYNGGSMQLILNKDLINKKDNREKSAVVIIS
ypest0001X_8970  MTTGIKIMMFMGVLFIKIDITPLSNHGINIYNGGSMQLILNKDLINKKDNREKSAVVIIS
yberc0001_6640   -----------------------------------MQLILNKDLISRKEHKIKNTIVIIS
ymoll0001_5950   -----------------------------------MQLILNKELISRKESKVKNTITIIS
yaldo0001_6640   -----------------------------------MQLILNKDIINKKDNKTKNTIAIIS
yinte0001_6740   -----------------------------------MQLILSKDAINKKNNKIKNTVAIIS
ykris0001_4910   -----------------------------------MQLILNKELINRKDNKIKSNIVIVS
yente0001X_6020  -----------------------------------MQLILNKELINRKDSKTRNNIVVMS
yrohd0001_6340   -----------------------------------MQLITNKDLISKKDTKIKNTIAIIS
yfred0001_41660  -----------------------------------MQLILNKDLINKKDSKIKNTIAVIS
                                                    #########################


                         70        80        90       100       110       120
                 =========+=========+=========+=========+=========+=========+
yruck0001_5190   NRAEIIDRVSEQIRITDEFEIDEVRVDIFEKNTFHISTSSYGVIIDIAYHTDIDAIKNLI
ypseu0001X_3757  TRKGLKDKISEYVRLADIKNIKEIDDDIFSAASILIPDNVKGIIIDINENHDINTILDLM
ypest0001X_8970  TRKGLKDKISEYVRLADIKNIKEIDDDIFSAASILIPDNVKGIIIDINENHDINTILDLM
yberc0001_6640   ERNWVIEKISEKIRLTDINQIKSVEKDIFNVTAVNIPDQTVGVILDISDNGDVEDILNLI
ymoll0001_5950   TRKWLIEEISEKIRLTDFDNIKKIEQDIFNAVTINLSGQTVGVIIDIGNNCDVEDTVNLI
yaldo0001_6640   DRKWLSNDTSEKVRLADINDIKVIEENIFTMSKINLSDQIIGAIIDIGSNDEIISTLNVI
yinte0001_6740   NREWLREEISEKVRLADISDIKSFDEDIFIVSKINVSDKTIGVIIDIVNNGDVAKTLDLI
ykris0001_4910   TRKWVIEKVSEKIRLADINDIKEIDKDIFNLSTINTPEKTVGFIIDIGNNEDVEKTLSLI
yente0001X_6020  TRKWVIEEVSEKIRLANINDIKEIDKDIFNISAINIPDQTVGFIIDIGHNEDVDNTLNLI
yrohd0001_6340   KRKWLIDNVAEKIRLADMNNIKEIEKDILTVSSVNLPEQTIGIIIDIGNDHNIKNILDLI
yfred0001_41660  QRSWLIEKVSEKIRLADINNIKEVDKDIFSTLSINLHEQTIGVIIDIGYSDDIEKILNLI
                 ############################################################


                        130       140       150       160       170       180
                 =========+=========+=========+=========+=========+=========+
yruck0001_5190   KTHIPRVSWCIIIGDNDSISNSQRFIEQGLLYLQSASQIDGLAQRLQKGIQIESDRRAFF
ypseu0001X_3757  KVHTPRDCWCILVGDIDSISIAQQFIERGILYLHIQSQLADLTQNLLKGIQIESDRKAFF
ypest0001X_8970  KVHTPRDCWCILVGDIDSISIAQQFIERGILYLHIQSQLADLTQNLLKGIQIESDRKAFF
yberc0001_6640   KNHVPRDCWCVLVGDIDSITIAQQFTECGLLYLNIQSQSAELTQYLLKGIQIESERKAFF
ymoll0001_5950   KNNTPRDCWCVLVGDIDSISIAQQFTDHGLLYLNMQSQSAELTQHLLKGIQVESGRRAFF
yaldo0001_6640   KSHVPRNCWCILIGDIDSISIAQQFIHRGILYLNIQSQLSELTQHLLKGINIESERKAFF
yinte0001_6740   KSHIPRDCWCVLVGDIDSISIAQQFIQRGILYLNIKSQLSELTQNLLKGIPVESERKAFF
ykris0001_4910   KSNTPRDCWCVLVGDIDSISVAQKFTERGILYLNVQSQSIELTQHLLKGIPIEMERKAFF
yente0001X_6020  KSNTPRDCWCVLVGDIDSISIAQKFTERGLLYLNVQSQSVELTQHLLKGIPIEAERKAFF
yrohd0001_6340   KNCTPRECWCVLVGDIDSISIAQQFTEQGILYLNIQSQSAELTQLLLKGIRIESDRKAFF
yfred0001_41660  KSHTPRDCWCILVGDIDSISIAQQFTERGLLYLNIQSQSSEITQLLLKGIQIDLERKAFF
                 ############################################################


                        190       200       210       220       230       240
                 =========+=========+=========+=========+=========+=========+
yruck0001_5190   ISVFGCRGGVGTTLISFHLANAISEIKKSPTLLIQGNHGSQDIDLLSEKKIDQNTINYNK
ypseu0001X_3757  ISILGCRGGIGTTLLSYQLAHVITQIKKSPTLLLQGNQGSQDLDLITEKKMGEDITGYQK
ypest0001X_8970  ISILGCRGGIGTTLLSYQLAHVITQIKKSPTLLLQGNQGSQDLDLITEKKMGEDITGYQK
yberc0001_6640   ISVLGCKGGIGTTLLSYHLAHEVTQLRKSPTLLLQGSQGSQDLDLVTEKKMGAELTEYQK
ymoll0001_5950   ISVLGCKGGIGTTLMSYHLAHEMTQIKKSPTLLLQGNQGSQDLDLITEKKMSTELTEYKK
yaldo0001_6640   ISILGCKGGIGTTLLSYHLASTVTQIKQLPTLLLQGNQGSQDLDLVTEKKMVTDITELQK
yinte0001_6740   ISILGCKGGIGNTLLSYHLANAITQIKQSPTLLLQGNNGSQDLDLVTEKKMSTEITEYQN
ykris0001_4910   ISILGCKGGIGTTLLSYHFSCEITQIKKSPTLLLQGNQGSQDLDLVTEKKMTSEINEYHK
yente0001X_6020  VSILGCKGGIGTTLLSYHFAYEITQIKKSPTLLLQGNQGSQDIDLITEKKMVSGVNEYHK
yrohd0001_6340   ISVLGCKGGIGTTLLSFHLANEIAQIKKTPTLLLQGNQGSQDLDLVVEKKMSTEITEYYK
yfred0001_41660  ISVLGCRGGIGNTLLSYHLANEINQIKKSPTLLLQGNQGSQDLDLITEKKMNAELTEYQK
                 ############################################################


                        250       260       270       280       290       300
                 =========+=========+=========+=========+=========+=========+
yruck0001_5190   DLDLMLLKENSLDSINENNNKKHNFIIFDQSIHNTSTEELDEYIKQSHCIIILLDHSMVS
ypseu0001X_3757  NFDLMRGNERKLSEVNEHNNKKHNFIIFDQPIHNARKEKTLDYISNSNCIILLIDHSMMS
ypest0001X_8970  NFDLMRGNERKLSEVNEHNNKKHNFIIFDQPIHNARKEKTLDYISNSNCIILLIDHSMMS
yberc0001_6640   NLDLMLCKEKKLSDINFHKTKKHSFIVFDQPIYNTPKENLTDYIEYSNCIVLILDNSMIS
ymoll0001_5950   NFDLMLCKDKKLSDADIQKNKKHNFIIFDQSIHNATKENLADYIEHSNCIVLMLDNSMVS
yaldo0001_6640   NFFIMFCKEKEINNIDVNINNKHNFIVFDQPIHNTSKERLTDYIEYSNCIILLLDNSMMS
yinte0001_6740   NLSLMFCKEQKIADIDGKIEIKHNFIVFDQPIHNAPKEKFTDYIECSNCIIILLDNSMMS
ykris0001_4910   NIDIMLCKGNELSDIDIKIGRKHNYIVFDQSIHNSPKEKLTGYIEHSDCIIILLDNSMTS
yente0001X_6020  NIDIMLCKGNELSDI--QIGRKHNYIILDQPIHNSPKEKLTDYIEHSDCIIILLDNSMSS
yrohd0001_6340   NLNLMRCKENKLSEVDAKTNNKHNFIVFDQPIHNIPKENITDYIEQSNCIIILLDNSMMS
yfred0001_41660  NFDLMFCREKNFSEVDAQTNKKHNFIVFDQSVHNASKENITEYIEQSNCIIILFDNSMIS
                 ############################################################


                        310       320       330       340       350       360
                 =========+=========+=========+=========+=========+=========+
yruck0001_5190   VRVAKKMIKTLEKHRIENNHKVRFILCLNESRPLTSSMLSTQDIQSLLSKNIDEKINYIN
ypseu0001X_3757  VRVAKEFIDTLERFKRDNRQAIRLFICLNENRPITKDMLTTSDIQSLLGHSIDTAIPYIN
ypest0001X_8970  VRVAKEFIDTLERFKRDNRQAIRLFICLNENRPITKDMLTTSDIQSLLGHSIDTAIPYIN
yberc0001_6640   VRIAKEFIDTYSRFKRDNKRGIKLIICLNESRPVTKDMLNTSDVQSLLGRTIDIKIPYIS
ymoll0001_5950   VRVAKEFIDFYTRFKRDNKQSVKLIICVNESRPITKDMLDISDIQSLLGKTIDIKTPYIY
yaldo0001_6640   VRVAKNFIDIYDRFKRDNRQATRLLVCLNESRPITRDMLDTSDIPSLLGRKINIRIPYMH
yinte0001_6740   VRVAKGFIDIYERIKRDNRQATRLLVCLNESRPITRDMLDTSDVPSLLGRKIDIRIPFIH
ykris0001_4910   VRVAKDFIDIYDRFKRDNRQTTRLIVCLNESRPIAKNMLNTSDIQSLLGRKIDTQIPYIS
yente0001X_6020  VRVAKEFIDIYDRFKRDNRQVTRLIICLNECRPIAKNMLDTSDVQTLLGRKIDTRIPYIA
yrohd0001_6340   VRIAKEFIGIYDRFKRDNRQAIRLILCLNESRPVAKSMLATTDIESLLERKIDIHIPYIY
yfred0001_41660  VRVAKEFIGIYDRFKRDNRQAIRLLICLNESRPVTKNMLDTADIQSLLGRTIDIHIPYIY
                 ############################################################


                        370       380       390       400       410
                 =========+=========+=========+=========+=========+==
yruck0001_5190   KPREK-----------------------------------------------
ypseu0001X_3757  NAKNSLNSPDYFGRKKTKVVELAKQSLGININLSSHTNMWIREKLNK-LFK-
ypest0001X_8970  NAKNSLNSPDYFGRKKTKVVELAKQSLGININLSSHTNMWIREKLNK-LFK-
yberc0001_6640   KGSDSISHSNYFGRRKRSITELAKHALGIRINTSVYNKMWINKIIQL-LK--
ymoll0001_5950   KKNNSLNDQKYFGRRKKTITELASQTLGIRMDSSTYSGTLIKKIITS-LKK-
yaldo0001_6640   KTKESLSDQNYFGRKKTIIIELAKHTLGISIDLSHNRGSWISKIIKT-RGKI
yinte0001_6740   KTTESLSDKNYFGRKKILITELAKYTLGITIDLSNKRRSWLRKILKG-KGEK
ykris0001_4910   KTKESLVDKNYFGRSKVKIIDLAKNTLGINIHLSKNGKSWINKIAAS-LK--
yente0001X_6020  KTKESLVDQNYFGRNKIKINYLAKNTLGINTHVSNNRKSWINKIVAS-LK--
yrohd0001_6340   KTKESLSDQNYFGRKKNKIDTLVKNTLGIRVNSSLKNRIWINRVISSFIGKR
yfred0001_41660  KTKSTLSDQNYFGRKKQK----------------------------------
                 ##################################
```

```
Parameters used
Minimum Number Of Sequences For A Conserved Position: 6
Minimum Number Of Sequences For A Flanking Position: 9
Maximum Number Of Contiguous Nonconserved Positions: 8
Minimum Length Of A Block: 10
Allowed Gap Positions: With Half
Use Similarity Matrices: Yes
```

```
Flank positions of the 1 selected block(s)
Flanks: [36  394]  

New number of positions in PGL1_unique_yersinia-CLUSTERS.dir/PGL1_unique_yersinia-CL1256/PGL1_unique_yersinia-CL1256.muscle.fasta.gblo:  359  (87% of the original 412 positions)
```
