# Supplementary material for: Genomic characterization of the Yersinia genus
Source: Genome Biol. 2010 Jan 4;11(1):R1. doi: 10.1186/gb-2010-11-1-r1 (PMC2847712; doi:10.1186/gb-2010-11-1-r1)
Supplement: Additional file 16 — The top level directory consists of a directory called Additional_cluster_files and 5010 directories, one for each multi-protein cluster family. (This top level directory has been split into three data files for uploading purposes (Additional files 15, 16, 17.) Within the directory are the following files: PGL1_unique_Yersinia_unclustered.out - list of all protein singletons that MCL did not group into a cluster (see Materials and Methods); PGL1_Yersinia_unique_locus_tags.txt - names of the 11 locus tag prefixes used for each genome; PGL1_unique_Yersinia.gff - mapping each Yersinia protein to a cluster in tab delimited GFF; PGL1_unique_Yersinia.sigfile - list of the longest protein in each cluster; PGL1_unique_Yersinia.summary - summary table of features of each of the clusters; PGL1_unique_Yersinia.table - summary table of each protein in the clusters. Within each cluster directory are the following files, where 'x' is the cluster name: PGL1_unique_Yersinia-x.faa - multifasta file of the proteins in the cluster; PGL1_unique_Yersinia-x.summary - summary of the properties of the proteins; PGL1_unique_Yersinia-x.matches - blast matches between the proteins of the cluster; PGL1_unique_Yersinia-x.muscle.fasta - muscle alignment of the proteins; PGL1_unique_Yersinia-x.muscle.fasta.gblo - gblocks output of muscle alignment (that is, auto-trimmed alignment); PGL1_unique_Yersinia-x.muscle.fasta.gblo.htm - as above in html format; PGL1_unique_Yersinia-x.muscle.tree - treefile from muscle alignment; PGL1_unique_Yersinia-x.sif - matches between proteins in simple interaction format for display on graphing software. [file gb-2010-11-1-r1-S16.zip › clusters2/PGL1_unique_yersinia-CL1257/PGL1_unique_yersinia-CL1257.muscle.fasta.gblo.htm]

PGL1\_unique\_yersinia-CL1257.muscle.fasta


## Gblocks 0.91b Results

Processed file: **PGL1\_unique\_yersinia-CL1257.muscle.fasta**  
Number of sequences: **11**  
Alignment assumed to be: **Protein**  
New number of positions: **261** (selected positions are underlined in blue)

```
                         10        20        30        40        50        60
                 =========+=========+=========+=========+=========+=========+
yinte0001_27000  MNDNKDMISLKKRIKF---VQNKLDSISSAKDMTFADRIMLNNMDAYMSDLQAEQRALDI
ypest0001X_9000  --MNRKFLILFSLLIVAIGISGILLN--PDKEIP-ELLTQTEKRKERNIVLAQTRHDLT-
ypseu0001X_3755  --MNRKFLILFSLLIVAIGISGILLN--PDKEIP-ELLTQTEKRKERNIVLAQTRHDLT-
ykris0001_4940   --MNRKFILLLSLLIVVVGIIGILINIDNDEKKP-YNSSELDSEKEVNIALAQSTHDLP-
yente0001X_6050  --MNRKFILLLSLLIVAVGIVGILINTDNGQKNT-QDSTELNREQDTNIALAISTHDLS-
yaldo0001_6660   --MNRKFLLLFSLLIVAIGATGILINIGDDKDIP-NGFVDMKKEKEVSIVLAQSTHDIA-
yinte0001_6760   --MNRKFLLLFSILIVAIGAAGIMINTKHDEHTP-YNFVDSKKEKEVEIVLAQSTHDLS-
ymoll0001_5970   --MNRKFLLLLSLFIVAIGITGILSNNGDDENNPIGNLVEFNKEKEVSIVLAQSTRTLS-
yberc0001_6660   --MNRKLILLFSLIIIAIGAAGVLGNREDGSNTL-TNLVEPKKEKIATIMLAQSTRDLS-
yfred0001_41680  --MNRKFLLLFSILIIAIGITGILVGYGDDKKET-TRLAQQNNEKKVTILLAQAIRDLP-
yrohd0001_6380   -----------------------LSGYGDSNKKE-GELIKPNIEKEIAIVLAQATHDLP-
                   ################################   #####################  


                         70        80        90       100       110       120
                 =========+=========+=========+=========+=========+=========+
yinte0001_27000  RHPLLDFMELRLKGSIVDLGTVPLEIMGIITSNLAAMVQRATHKL---------------
ypest0001X_9000  AGTLISKEDYSLQNITVD------ESSDLVKNDLSHGHRIDGDSI----------ADDRT
ypseu0001X_3755  AGRLISKEDYSLQNIIVD------ESSDLVKNDLSHGHRIDSDSIDGDSIDGDRTADDRT
ykris0001_4940   SGTILRRQDYAIKKLSVK------ESSELVESNISNSTDINS------------------
yente0001X_6050  SGTILTANDYAIKNILVK------ESSELVKSNISSSAEINR------------------
yaldo0001_6660   SGTLLTQDDYSLKTIIVP------ESSTLIRSDISNSTNINN------------------
yinte0001_6760   SGTLLTQNDYALKVIMVP------ESSELIKSNISGKTDINS------------------
ymoll0001_5970   SGSILAKGDYALKTIIVP------ESSEWVKSDVSKIENINH------------------
yberc0001_6660   SGTILTKDDYALKTVMVP------ESSQLIKSDLSNPENIKS------------------
yfred0001_41680  SDTLLDKTDYAIKKIAVD------ESSDLIKSDISDIININS------------------
yrohd0001_6380   SGSLLTKMDYSIKKSAVP------QSSKLIKDDISAIPNIDS------------------
                 #################       ################                    


                        130       140       150       160       170       180
                 =========+=========+=========+=========+=========+=========+
yinte0001_27000  -SSGRDSHKVPYSIKSSLNMRLAELSPGSTRLGLTFSTGQCELVETVSSKAIKE-IINLL
ypest0001X_9000  HDHKLDGHLLKNNILAGSYIIDEMLISPDSREFSRLNLKHGEIIYKFYITEKNEYLLNTL
ypseu0001X_3755  HDHRLDGHLLKNNILAGSYITDEMLISPSSREFSRLNLKHGEIIYKFYITEKNEYLLNTL
ykris0001_4940   -------YLLKENVLSGSYLTKNMLISPNSDEFNHLILKKGNVIYKFNLKKQDEYLLDSL
yente0001X_6050  -------HLLKENVLAGSYITKDMLAIPDSDEFNHLVLKKGNVIYKFNLKKQDEYLLDSL
yaldo0001_6660   -------NLLKSNLLAGSYITKDLLVSPDSDEFLRLNLSKGDVIYKFNIKKHNEYLLDTL
yinte0001_6760   -------HLLKTNLLSGSYITPAVLASPDSNEFSYLNLKKGEVIYKFNINNKNGYLLDTL
ymoll0001_5970   -------HLLRSNVLAESYITHEMLVSPESNEFSRLILKKGEVIYKLEIKQLEEYLLDTL
yberc0001_6660   -------HLLKTNILSGSYITNEMLVSPESSEFDRLSLKKGEVIYKFDIEQQEQHLLDVL
yfred0001_41680  -------YLLKTNVVTGSYITKDMLVPPDSDEFIHLNLQKDHMIYKFDIKQQEDYLLNTL
yrohd0001_6380   -------YLLKHNILAGSYITQDMLVSPDSDEFNYHNLQQGQIIYKFNIKQQDEYLLDTL
                         ####################################################


                        190       200       210       220       230       240
                 =========+=========+=========+=========+=========+=========+
yinte0001_27000  DAVDATTMMNQVAEIGFNSAQSLKKIVEECDKNHIDFDLSWIGPFSDGNRKVSVNSTKIK
ypest0001X_9000  NPGDFLSFQLLTLETNKTKGMENGIAIDSKSMSSKQRQKYSLNNVIPDMPILSIKTYSPE
ypseu0001X_3755  NPGDVLSFQLLTLETNKTKGMENGIAIDSKSMNSKQRQKYSLNNVIPDMPILSIKTYSPE
ykris0001_4940   NIGDWLSFQLRTLETDKRKGMDNGTTINKKGMNDRQRQSYSLSKLIQKMEIVRIKKYSES
yente0001X_6050  NTGNRLSFQLITLETDKRKGMENGTTINKKGMNSRQRQNYSLNKLIQSMEIIRIKKYSKD
yaldo0001_6660   QVGDVLAFQLRLLETDKKSGMDNGISINTKDINDRKNQIYSLNKIIPAMRIVRIKKYLED
yinte0001_6760   QVGDSLSFQLRVLETDKNKGMSSGTVINTKELSDKKSQTFSLNEIIPAMRIIRVKKYSAD
ymoll0001_5970   SAGDLLSFQLRTLETDQRKGTENGISINTNEMNDRKKQSYSLTEIIPDMRIIRVNKYSTS
yberc0001_6660   NLGDTLSLQLRTLETDLRKGVENGIAINTQEMNDRKKQNYLLTEVIPNMRVIRIKKYSAN
yfred0001_41680  RIGDTISLQLRTLETDKKKGMGNGIVINTKKMSGISNKNYSLNDIISSMKVVRIKKHSAA
yrohd0001_6380   QIGDVVSFQLRALEIDNNKGMENGTVIESNSMNNRKKQSYSLNEIITGVKIIRIKKHSER
                 ############################################################


                        250       260       270       280       290       300
                 =========+=========+=========+=========+=========+=========+
yinte0001_27000  MLSDRLAATTISSPVIETIVGELASLSKYGKLEIEVDGEKIRASFPVDMLENIQKKHKVG
ypest0001X_9000  ELSVKNNKNNKTE---EYSLGYIEVIMKIQDLEFIHTVEKAGEVFLTPKSG----DHKRI
ypseu0001X_3755  ELSVKNNKNNKTE---AYALGYIEVIMKIQDLEFIHTVEKAGEVFLTPKSG----DHRRI
ykris0001_4940   ELSEINGKNQKTE---ETLSGYIEVIINMQDLDLIYLAEASGDIILTPSID--DEKHKSK
yente0001X_6050  ELLEINGKNRKTE---EMLIGYIDVIIDMQDLDLIYLAEISGEIILTPTID--GEDDKSK
yaldo0001_6660   ELSEKNKKNQKTE---ELLTGFIEVIVKRDELDIIHITEKSGDTFLTPSSN--DEKNKII
yinte0001_6760   ELSEKNNKNQKTE---EVVTGYISVMIKREELDIIHIAEKSGDIILAPSIV--SDNNQST
ymoll0001_5970   ELSEINNNNQKTK---DRLAGYIDVVISTDELDLIHIAEKAGDIFLIPSIKPRDQKYKSK
yberc0001_6660   ELSEENKKNQKTK---ISLVGYIEVIINTEELNIIHLAEKSGDIFLIPGTELRDEHHTSK
yfred0001_41680  ELSEQNRNNQKNN---EEIKGYIYVVMKTKDLDLIRLVENSGDIFLMPSYN-IRDNTKHN
yrohd0001_6380   ELSEKKYNNQKTE---NASAGYIDVIIKTEDLDVLHIVEKSGDIFLTPSYKPDDKESRAR
                 #############   #################################        ###


                        310       320       330
                 =========+=========+=========+
yinte0001_27000  KKLSLVVEVTDINNDNLGLHRKNYFVKSFN
ypest0001X_9000  DLDDIIPTLQTIRE-----------LRG--
ypseu0001X_3755  DLDDIIPTLQTIRE-----------LRG--
ykris0001_4940   YLHDILPELRTIRE-----------LRG--
yente0001X_6050  HLYDILPELRTIRE-----------LRG--
yaldo0001_6660   NLYDVIPKLRTTRE-----------LRG--
yinte0001_6760   NIYDIIPKLRITRE-----------LRG--
ymoll0001_5970   NLHDILPKLHTIRE-----------LRG--
yberc0001_6660   SLHDIFPKLRTIRE-----------LRG--
yfred0001_41680  NLYEIIPKLRTTRE-----------LRG--
yrohd0001_6380   NLYDVIPKLRTTRE-----------LRG--
                 ##############
```

```
Parameters used
Minimum Number Of Sequences For A Conserved Position: 6
Minimum Number Of Sequences For A Flanking Position: 9
Maximum Number Of Contiguous Nonconserved Positions: 8
Minimum Length Of A Block: 10
Allowed Gap Positions: With Half
Use Similarity Matrices: Yes
```

```
Flank positions of the 7 selected block(s)
Flanks: [3  34]  [38  58]  [61  77]  [85  100]  [129  253]  [257  289]  [298  314]  

New number of positions in PGL1_unique_yersinia-CLUSTERS.dir/PGL1_unique_yersinia-CL1257/PGL1_unique_yersinia-CL1257.muscle.fasta.gblo:  261  (79% of the original 330 positions)
```
