# Supplementary material for: Genomic characterization of the Yersinia genus
Source: Genome Biol. 2010 Jan 4;11(1):R1. doi: 10.1186/gb-2010-11-1-r1 (PMC2847712; doi:10.1186/gb-2010-11-1-r1)
Supplement: Additional file 16 — The top level directory consists of a directory called Additional_cluster_files and 5010 directories, one for each multi-protein cluster family. (This top level directory has been split into three data files for uploading purposes (Additional files 15, 16, 17.) Within the directory are the following files: PGL1_unique_Yersinia_unclustered.out - list of all protein singletons that MCL did not group into a cluster (see Materials and Methods); PGL1_Yersinia_unique_locus_tags.txt - names of the 11 locus tag prefixes used for each genome; PGL1_unique_Yersinia.gff - mapping each Yersinia protein to a cluster in tab delimited GFF; PGL1_unique_Yersinia.sigfile - list of the longest protein in each cluster; PGL1_unique_Yersinia.summary - summary table of features of each of the clusters; PGL1_unique_Yersinia.table - summary table of each protein in the clusters. Within each cluster directory are the following files, where 'x' is the cluster name: PGL1_unique_Yersinia-x.faa - multifasta file of the proteins in the cluster; PGL1_unique_Yersinia-x.summary - summary of the properties of the proteins; PGL1_unique_Yersinia-x.matches - blast matches between the proteins of the cluster; PGL1_unique_Yersinia-x.muscle.fasta - muscle alignment of the proteins; PGL1_unique_Yersinia-x.muscle.fasta.gblo - gblocks output of muscle alignment (that is, auto-trimmed alignment); PGL1_unique_Yersinia-x.muscle.fasta.gblo.htm - as above in html format; PGL1_unique_Yersinia-x.muscle.tree - treefile from muscle alignment; PGL1_unique_Yersinia-x.sif - matches between proteins in simple interaction format for display on graphing software. [file gb-2010-11-1-r1-S16.zip › clusters2/PGL1_unique_yersinia-CL1258/PGL1_unique_yersinia-CL1258.muscle.fasta.gblo.htm]

PGL1\_unique\_yersinia-CL1258.muscle.fasta


## Gblocks 0.91b Results

Processed file: **PGL1\_unique\_yersinia-CL1258.muscle.fasta**  
Number of sequences: **11**  
Alignment assumed to be: **Protein**  
New number of positions: **116** (selected positions are underlined in blue)

```
                         10        20        30        40        50        60
                 =========+=========+=========+=========+=========+=========+
yinte0001_12000  VNSLTIINKIVAYDYNNAIIYN-----INKPEKSVSLYAPTN-ECLYILLDQYPNVIPQN
yruck0001_5250   --MTKNNTENE-FELENGVIFSPARRYLHGPDAPPVILTENNIRFLQLLLN---GITDKE
yberc0001_6690   VAANMETNEIV-FKLEGTVLFSPAQRCLNGPNGAIAILTENNIRFLKLLLS---GVTEKE
ypseu0001X_3752  ----MEKNEIV-FKLEGAVLFSPEKRCLSGPGGTVAVLTENNLRFLQLLLN---GVTEKE
yinte0001_6790   ----MEKNEIM-FKLEGTVLFSPTQRCLNGPDGTVVILTENNLRFLQLLLN---GVTEKE
yaldo0001_6680   ----MEINEIV-FKLEGTVLFSPAQRCLNGPNGSIAILTENNLRFLQLLLN---GVTEKE
ymoll0001_5990   ----MENKETV-FKLERTVLFSPAQRCLNGPEGTVVILTENNIRFLQLLLS---GVTAKE
yrohd0001_6410   ----METKEIV-FKLEGIVLFSPSQRCLNGPDGSVVILTENNLRFLQLLLN---GVTEKE
yfred0001_41710  ----METNEIV-FKLEGTVLFSPSQRCLNGPDGSVVILTENNLRFLQLLLN---GITEKE
yente0001X_6080  ----METNEIV-FKLEGTVLFSPAQRCLNGPDGSVVILTENNLRFLQLLLN---GVTEKE
ykris0001_4970   ----MEINEIV-FKLEGTVLFSPTQRCLNGPGGSVVMLTENNLRFLQLLLN---GVTEKE
                             #######################################   ######


                         70        80        90       100       110       120
                 =========+=========+=========+=========+=========+=========+
yinte0001_12000  YFFEQAWEKQGLTTTNNNFYQHISMIRRAFEVVGLNGDIILTLPRRGLSLSKDLEITHEE
yruck0001_5250   SIINEVWKEQNGAISESSYYGQIYMLRKAFNQVGLNESLIHTIPRKGVRYIGSAMPLLVT
yberc0001_6690   QIINQVWKEQRGAVSESSYYGQLYMLRKAFVQVGLKESLIHTIPRKGVRYIGAVNQITTC
ypseu0001X_3752  EIINEVWKEQRGAISESSYYGQLYMLRKAFLQVGLKESLIHTIPRKGVRYTGSISQVAIN
yinte0001_6790   QIINQVWKEQRGAVSESSYYGQLYMLRKAFLQVGLKESLIHTIPRKGVRYTGSVSQIVIN
yaldo0001_6680   QIINQVWKEQRGAISESSYYGQLYMLRKAFVQVGLKESLIHTIPRKGVRYTGSVTQVTIN
ymoll0001_5990   QIINQVWREQRGAVSESSYYGQLYMLRKAFVQVGLKESLIHTIPRKGVRYTGSVEQIVIC
yrohd0001_6410   EIINQVWKEQRGAVSESSYYGQLYMLRKAFVQVGLKESLIHTIPRKGVRYTGSVSQIAAC
yfred0001_41710  KIINQVWKEQRGAVSESSYYGQLYMLRKAFLQVGLKESLIHTIPRKGVRYTGSVSQIEVC
yente0001X_6080  QIINQVWKEQRGAVSESSYYGQLYMLRKAFLQVGLKESLIHTIPRKGVRYTGSVSKIAVC
ykris0001_4970   KIINQVWKEQRGAVSESSYYGQLYMLRKAFLQVGLKESLIHTIPRKGVRYTGSVSQVTVS
                 #####################################################       


                        130       140       150       160       170       180
                 =========+=========+=========+=========+=========+=========+
yinte0001_12000  KKRQDKKEEIVIDETSNNATINNSMLRASLWVVVSILIITLFSILLYNKKSPYEKSIENY
yruck0001_5250   EANDILGK----------------------------------------------------
yberc0001_6690   DEATEQPQQSETNILA--------------------------------------------
ypseu0001X_3752  SNPENQQSSETANSVS--------IM----------------------------------
yinte0001_6790   DEAEKQMQADPHIIAV--------------------------------------------
yaldo0001_6680   QQPEIQQHSKEQKTME--------------------------------------------
ymoll0001_5990   NKKEQQQNSHNIQSITADNTLTDDTL----------------------------------
yrohd0001_6410   KEIESPQSGIDNTQVT--------TE----------------------------------
yfred0001_41710  KKPENPQDSDATEQIV--------AI----------------------------------
yente0001X_6080  KEPQEQQNSTDMQQMP--------AL----------------------------------
ykris0001_4970   NDSDEQQNNDDIQQIT--------TL----------------------------------
                                                                             


                        190       200       210       220       230       240
                 =========+=========+=========+=========+=========+=========+
yinte0001_12000  NHVEDIELCHLFSLSEGYDVSNVKEIIKNEKINCN-KVKNLYYTTYPIIKRESLIYCDVL
yruck0001_5250   NKHPLISTTTLHAVNLGDDEKTAAT--KMFRMNTYHRWKKIFFYSLTFLATCWLSSLTIL
yberc0001_6690   TAAPAPLALSPSMASVVAQVALPPT--KPGFLQSY-RWKKL-ISLLAFFSFCWLSFLSVL
ypseu0001X_3752  HDPAPLAVATIPTDAGTMTLSSPPT--KQSFLHSH-RWKKL-ISLLAVFSFCWLSFLSAL
yinte0001_6790   PNKEQLAPVTQPFKREASKISPVVA--QQSFLHSY-RWKKL-ISLLAFFSFCWLSFLSTL
yaldo0001_6680   PDMHQVGVATAPFNSTVIPVSKRVV--RQSFLQSY-RWKKL-ISLLAFFSFCWLSFLSAL
ymoll0001_5990   VENRQISTPSLPIKNAITLVSQPPV--KQSFLQSH-RWKKL-ISLLAFFSFCWLSFLSVL
yrohd0001_6410   PESSYIPMPSLPIKSAPTAVPHAQI--KHNFLQSH-GWKKL-ISLLAFFSFCWLSFLSVL
yfred0001_41710  TEQPQINTIPLPANSMAALANQTQSHTKQSFLQSY-RWKKL-ISLLALFSFCWLSFLSIL
yente0001X_6080  PDVSQLTTLPLPIENIAPQEFPAQN--KQSFLQSY-RWKKL-ISLLAFFSFCWLSFLSVL
ykris0001_4970   PELPQITTSSLPAESVTPRVPLANN--KQSFLQSN-GWKKL-ISLLAFFSFCWLSFLSVL
                                                           ##################


                        250       260
                 =========+=========+
yinte0001_12000  NNTPKNCISYLMIK------
yruck0001_5250   -------IIIYLNK------
yberc0001_6690   -------VIIILFHGDTPHP
ypseu0001X_3752  -------VIIILLKGE----
yinte0001_6790   -------IIIILFNGDRPHF
yaldo0001_6680   -------IIIILLNGDTP--
ymoll0001_5990   -------IIIILFNGNNSPH
yrohd0001_6410   -------IIIILFNGNNPAS
yfred0001_41710  -------VIIILFNGDKSNY
yente0001X_6080  -------IMIILFSGEKPNP
ykris0001_4970   -------IIIILFNGDTPNP
```

```
Parameters used
Minimum Number Of Sequences For A Conserved Position: 6
Minimum Number Of Sequences For A Flanking Position: 9
Maximum Number Of Contiguous Nonconserved Positions: 8
Minimum Length Of A Block: 10
Allowed Gap Positions: With Half
Use Similarity Matrices: Yes
```

```
Flank positions of the 3 selected block(s)
Flanks: [13  51]  [55  113]  [223  240]  

New number of positions in PGL1_unique_yersinia-CLUSTERS.dir/PGL1_unique_yersinia-CL1258/PGL1_unique_yersinia-CL1258.muscle.fasta.gblo:  116  (44% of the original 260 positions)
```
