# Supplementary material for: Genomic characterization of the Yersinia genus
Source: Genome Biol. 2010 Jan 4;11(1):R1. doi: 10.1186/gb-2010-11-1-r1 (PMC2847712; doi:10.1186/gb-2010-11-1-r1)
Supplement: Additional file 16 — The top level directory consists of a directory called Additional_cluster_files and 5010 directories, one for each multi-protein cluster family. (This top level directory has been split into three data files for uploading purposes (Additional files 15, 16, 17.) Within the directory are the following files: PGL1_unique_Yersinia_unclustered.out - list of all protein singletons that MCL did not group into a cluster (see Materials and Methods); PGL1_Yersinia_unique_locus_tags.txt - names of the 11 locus tag prefixes used for each genome; PGL1_unique_Yersinia.gff - mapping each Yersinia protein to a cluster in tab delimited GFF; PGL1_unique_Yersinia.sigfile - list of the longest protein in each cluster; PGL1_unique_Yersinia.summary - summary table of features of each of the clusters; PGL1_unique_Yersinia.table - summary table of each protein in the clusters. Within each cluster directory are the following files, where 'x' is the cluster name: PGL1_unique_Yersinia-x.faa - multifasta file of the proteins in the cluster; PGL1_unique_Yersinia-x.summary - summary of the properties of the proteins; PGL1_unique_Yersinia-x.matches - blast matches between the proteins of the cluster; PGL1_unique_Yersinia-x.muscle.fasta - muscle alignment of the proteins; PGL1_unique_Yersinia-x.muscle.fasta.gblo - gblocks output of muscle alignment (that is, auto-trimmed alignment); PGL1_unique_Yersinia-x.muscle.fasta.gblo.htm - as above in html format; PGL1_unique_Yersinia-x.muscle.tree - treefile from muscle alignment; PGL1_unique_Yersinia-x.sif - matches between proteins in simple interaction format for display on graphing software. [file gb-2010-11-1-r1-S16.zip › clusters2/PGL1_unique_yersinia-CL1259/PGL1_unique_yersinia-CL1259.muscle.fasta.gblo.htm]

PGL1\_unique\_yersinia-CL1259.muscle.fasta


## Gblocks 0.91b Results

Processed file: **PGL1\_unique\_yersinia-CL1259.muscle.fasta**  
Number of sequences: **11**  
Alignment assumed to be: **Protein**  
New number of positions: **0** (selected positions are underlined in blue)

```
                         10        20        30        40        50        60
                 =========+=========+=========+=========+=========+=========+
ypseu0001X_3602  ------------------------------------VLAEDTIIVERNRSQTDSYQATTS
ypest0001X_1162  ------------------------------------VLAEDTIIVERNRSQTDSYQATTS
yinte0001_28810  ------------------------------------------------------------
yfred0001_27640  ------------------------------------------------------------
yaldo0001_28650  ------------------------------------------------------------
yberc0001_26380  ------------------------------------------------------------
yaldo0001_28640  ------------------------------------------------------------
yaldo0001_28660  ------------------------------------------------------------
yruck0001_22100  MKIINIRRLHWFGLPLLFAIPTVQA-----ETAPQSEKTKMVVIGQTNEEESQSYQPTSS
ykris0001_27690  ------------------MGSAAQAVDSQAATKNSEEKNKIVVIGQQREADSQSYQPTTS
yente0001X_1070  MKIIKRQHMYWCSLPLLVMGPIAQAADSQTATKNSEEKTKIVVIGQQREADTQSYQPTSS
                                                                             


                         70        80        90       100       110       120
                 =========+=========+=========+=========+=========+=========+
ypseu0001X_3602  VTANRSPANIIDTPQNVTVVTHPVLEDYDVTNLGEALYFVSGITQSNTLGGTQDALIKRG
ypest0001X_1162  VTANRSPANIIDTPQNVTVVTHPVLEDYDVTNLGEALYFVSGITQSNTLGGTQDALIKRG
yinte0001_28810  ------------------------------------------------------------
yfred0001_27640  ------------------------------------------------------------
yaldo0001_28650  ------------------------------------------------------------
yberc0001_26380  ------------------------------------------------------------
yaldo0001_28640  ------------------------------------------------------------
yaldo0001_28660  ------------------------------------------------------------
yruck0001_22100  VTGTRTSTNLLNVPQAINVVPPQVLRDQAVRNIDEALYNVSGITQSNTLGGTQDALMKRG
ykris0001_27690  ITGTRTETNLLNVPQAVNVVPRQVLRDQAVRNIDEALYNVSGITQSNTLGGTQDAVMKRG
yente0001X_1070  VTGTRTETNLLNVPQAVNVVPRQVLRDQAARNIDEALYNVSGITQSNTLGGTQDAVMKRG
                                                                             


                        130       140       150       160       170       180
                 =========+=========+=========+=========+=========+=========+
ypseu0001X_3602  FGNNRDGSILHDGIRSIQARNFTPTSERVEVLKGPSSMLYGMNEPGGLINVISKKPQLDP
ypest0001X_1162  FGNNRDGSILHDGIRSIQARNFTPTSERVEVLKGPSSMLYGMNEPGGLINVISKKPQLDP
yinte0001_28810  ----------------------------VEVI----------------------------
yfred0001_27640  ------------------------------------------------------------
yaldo0001_28650  ------------------------------------------------------------
yberc0001_26380  ------------------------------------------------------------
yaldo0001_28640  ------------------------------------------------------------
yaldo0001_28660  ------------------------------------------------------------
yruck0001_22100  FGDNRDGSILRDGVRSAQARNFTPTTERIEVLKGPASMLYGMGEPGGVINVITKKPQLVQ
ykris0001_27690  FGDNRDGSILRDGVRSAQARNFTPTTERVEVLKGPASMLYGMGEPGGVINMITKKPQLVQ
yente0001X_1070  FGDNRDGSILRDGVRSAQARNFTPTTERVEVLKGPASMLYGMGEPGGVINMITKKPQLVQ
                                                                             


                        190       200       210       220       230       240
                 =========+=========+=========+=========+=========+=========+
ypseu0001X_3602  HVHLEANASSFKGGGGQLDVTGPLGESGFAGRLIIDHQETDYWRNFGRNRQTVVAPSLDW
ypest0001X_1162  HVHLEANASSFKGGGGQLDVTGPLGESGFAGRLIIDHQETDYWRNFGRNRQTVVAPSLDW
yinte0001_28810  ------------------------------------------------------------
yfred0001_27640  ------------------------------------------------------------
yaldo0001_28650  ------------------------------------------------------------
yberc0001_26380  ------------------------------------------------------------
yaldo0001_28640  ------------------------------------------------------------
yaldo0001_28660  ------------------------------------------------------------
yruck0001_22100  RNHIEGWGSSFNGGGGQLDVTGPIGQSGFAYRMIVDHDETDYWRNFGRNRQTVIAPSLMW
ykris0001_27690  QTHIEGWGSSFKGGGGQLDVTGPLGQSGFAYRMIVDHDETDYWRNFGRNRQTVVAPSVMW
yente0001X_1070  QTHIEGWGSSFKGGGGQLDVTGPLGQSGFAYRMIVDHDETDYWRNFGRNRQTVVAPAVMW
                                                                             


                        250       260       270       280       290       300
                 =========+=========+=========+=========+=========+=========+
ypseu0001X_3602  YGENTTVHLAWERMEYLVPFDRGTVIDPRTGKPVDTPRDRRFDESYNATRGDQDTVTFSI
ypest0001X_1162  YGENTTVHLAWERMEYLVPFDRGTVIDPRTGKPVDTPRDRRFDESYNATRGDQDTVTFSI
yinte0001_28810  ------------------------------------------------------------
yfred0001_27640  ------------------------------------------------------------
yaldo0001_28650  ------------------------------------------------------------
yberc0001_26380  ------------------------------------------------------------
yaldo0001_28640  ------------------------------------------------------------
yaldo0001_28660  ------------------------------------------------------------
yruck0001_22100  FGDTTTVRLAYEHMEYLTPFDRGTIIDSRTGKPVDTPRKRRFDEPFNATRGDQDNITLHI
ykris0001_27690  LGETTTVRVAYEHMEYLTPFDRGTIIDRRTGKPVDTPRDRRFDEAYNATRGDQDNITLQI
yente0001X_1070  FGDTTTVRVAYEHMEYLTPFDRGTIIDSRTGKPVNTPRDRRFDEAYNATRGDQDNITLQI
                                                                             


                        310       320       330       340       350       360
                 =========+=========+=========+=========+=========+=========+
ypseu0001X_3602  NHDLNEFWSTQLNYAFSRNSYSDNQARATAFNAETGVLTRQSDATANAKSHSQAVQWVVN
ypest0001X_1162  NHDLNEFWSTQLNYAFSRNSYSDNQARATAFNAETGVLTRQSDATANAKSHSQAVQWVVN
yinte0001_28810  ------------------------------------------------------------
yfred0001_27640  ------------------------------------------------------------
yaldo0001_28650  ------------------------------------------------------------
yberc0001_26380  ------------------------------------------------------------
yaldo0001_28640  ------------------------------------------------------------
yaldo0001_28660  ------------------------------------------------VSWAQAVQLTLM
yruck0001_22100  DQELNDDWKSALTYAYNRNRYSDNQARALTLNPESGVLTRKADATASAVSRAQAVQATLQ
ykris0001_27690  DQVLSDNWKSSLTYAYNRNRYSDNQARALLLNPITGVLTRQADATASAVSRAQAVQMTLN
yente0001X_1070  DQVLNDNWKSSLTYAYNRNRYSDNQARALLLDPITGVLSRQADATASAVSRAQAVQMTLN
                                                                             


                        370       380       390       400       410       420
                 =========+=========+=========+=========+=========+=========+
ypseu0001X_3602  GDLDWGFTHHQLMFGIDYEARRVFRGDMIRGSKNNGFNVYDPIYGTLPPSTAVSAADSDQ
ypest0001X_1162  GDLDWGFTHHQLMFGIDYEARRVFRGDMIRGSKNNGFNVYDPIYGTLPPSTAVSAADSDQ
yinte0001_28810  -------------------------------------------------ASTASAKGRDQ
yfred0001_27640  ------------------------------------------------------------
yaldo0001_28650  ------------------------------------------------------------
yberc0001_26380  ------------------------------------------------------------
yaldo0001_28640  ------------------------------------------------------------
yaldo0001_28660  GDLGLVGVGHQILVGFDFEDNRTYRGDMVRGKKNSNFNIYHPIYGLMPPPTAVSAKDSDQ
yruck0001_22100  GDLTWGSIGHQVLVGFDYEDNRTYRGDMIRGKKNSDFNIYDPVYDLMPPSTLVSAKDSDQ
ykris0001_27690  GDLDLGGMGHQMLLGFDYEDNRTYRGDMIRGKKDSDFNIYHPVYGLMPPSTAISAKDSDQ
yente0001X_1070  GDLDLGGMGHQMLFGFDYEDSRTYRGDMIRGKKNSNFNIYDPVYGLMPPSTAVSAKDSDQ
                                                                             


                        430       440       450       460       470       480
                 =========+=========+=========+=========+=========+=========+
ypseu0001X_3602  RENIDSRALFIQDSMRLNEHWLLLGGLRYDSFDVMAGKGRPFKKNTDSSDSRLVPRAGVV
ypest0001X_1162  RENIDSRALFIQDSMRLNEHWLLLGGLRYDSFDVMAGKGRPFKKNTDSSDSRLVPRAGVV
yinte0001_28810  RENLKSDGWFMQDSIELIDKWVVLTGLRYE------------------------------
yfred0001_27640  --------------------------------------------------------MGVV
yaldo0001_28650  ------------------------------------------------------------
yberc0001_26380  --------------LEFVKIWKYLS-----------------------------------
yaldo0001_28640  ------------------------------------------------------------
yaldo0001_28660  RENLKSYGWFMQDSIELTDKWGALAGLRYDHFDIFAGKGRPLVTNTDSSDSKLIPRTGVV
yruck0001_22100  RENLKSYGWFVQDAIELGDHWVFLAGLRYDSFDVFAGKGRPFVTNTNSSDSKLIPRTGVV
ykris0001_27690  RENLKSYGWFMQDSIELTDKWIVLAGLRYDRFDVFAGKGRPFITNTDSSDSKLVPRTGVV
yente0001X_1070  RENLKSYGWFMQDSIELTDKWIVLAGLRYDRFDVFAGKGRPFITNTDSSDSKLVPRAGVV
                                                                             


                        490       500       510       520       530       540
                 =========+=========+=========+=========+=========+=========+
ypseu0001X_3602  YNLNDWSSLYVSYTESFKPNVSIATAIDALPPEYGKSWEAGYKIDINNRVTGTLAVYDIH
ypest0001X_1162  YNLNDWSSLYVSYTESFKPNVSIATAIDALPPEYGKSWEAGYKIDINNRVTGTLAVYDIH
yinte0001_28810  ------------------------------------------------------------
yfred0001_27640  YKLMSGVFLYSSNSESFIPNFSITTQIDLLPPVQG-------------------------
yaldo0001_28650  ------------------------------------------------------------
yberc0001_26380  ------------------------------------------------------------
yaldo0001_28640  ------------------------------------------------------------
yaldo0001_28660  YKLTPEISWYGSYSESFKSNSSIATQIDSLPPEQGQSWKVSSKVE---------------
yruck0001_22100  YKLTPEVSLYGSYSESFKPNSSIATQISELPPEQGQSWELGSKVEISNGVTGTLALFDIT
ykris0001_27690  YKLTPEVSLYGSYSESFKPNSSIATQIDSLPPEQGQSWEIGSKVELVNGVTGTLALFDIA
yente0001X_1070  YKLTPEVSLYGSYSESFKPNSSIATQIDSLPPEQGQSWEVGSKVELVNGVTGTLALFDIA
                                                                             


                        550       560       570       580       590       600
                 =========+=========+=========+=========+=========+=========+
ypseu0001X_3602  KRNIMVSELVDGETVTRTAGKARSRGLELDMAGKVTDSLSLIGSYAYTDARLTEDPDNNG
ypest0001X_1162  KRNIMVSELVDGETVTRTAGKARSRGLELDMAGKVTDSLSLIGSYAYTDARLTEDPDNNG
yinte0001_28810  ------------------------------------------------------------
yfred0001_27640  ------------------------------------------------------------
yaldo0001_28650  ----MVNELVDGEAVTRTAGRVRSQGVEVDVSGQLTGNLSAIATYAYIDARVSEDPENKA
yberc0001_26380  ------------------------------------------------------------
yaldo0001_28640  ------------------------------------------------------------
yaldo0001_28660  ------------------------------------------------------------
yruck0001_22100  KRNVMVSELVGTETVTRTAGRVRSQGVELDVAGQLTDSVSAITSYAYIDARVTEDPVNTG
ykris0001_27690  KRNVMVNELVNGETITRTAGRVRSQGVEFDVAGQLTDSLSAIATYAYIDARVTEDPDNKG
yente0001X_1070  KRNVMVNELVNGETITRTAGRVRSQGVELDIAGQLTDNLSAIATYAYTDARVTEDPDNKG
                                                                             


                        610       620       630       640       650       660
                 =========+=========+=========+=========+=========+=========+
ypseu0001X_3602  NDLPNVARHTAALFLSRDFGSTSLISGDEVKAGIGARYVGKRAGDAANSFWLDNYTVADA
ypest0001X_1162  NDLPNVARHTAALFLSRDFGSTSLISGDEVKAGIGARYVGKRAGDAANSFWLDNYTVADA
yinte0001_28810  ------------------------------------------------------------
yfred0001_27640  ------------------------------------------------------------
yaldo0001_28650  NQMANVARNRASLFDSD-------------------------------------------
yberc0001_26380  ---------------------------VNIGIGAGARYIGSRAGDAANRFILDDYTVVDA
yaldo0001_28640  --------------LTQTLGFTGWQSGDNLRIGAGARYFGRRGGDVANSFTLDDYTAADT
yaldo0001_28660  ------------------------------------------------------------
yruck0001_22100  NRMANVARNSASLFLTKAMGSTGWHGGDDLRFGGGMRYVGKRAGDAANSFTLDDYTVADA
ykris0001_27690  NQMANVARNSASIFMTQALGSTGWQGGDDLRIGAGARYVGRRAGDAANSFTLDDYTVADA
yente0001X_1070  NQMTNVARNSASVFLTQALGSTGWQGGDDLRIGAGARYVGRRAGDAANSFTLDDYTVADA
                                                                             


                        670       680       690       700       710
                 =========+=========+=========+=========+=========+===
ypseu0001X_3602  FVAWKMPLSGYQLKWQLNVKNLFDKTYYPSSANNLRIAIGEPRQVVLQGSIDF
ypest0001X_1162  FIAWKMPLSGYQLKWQLNVKNLFDKTYYPSSANNLRIAIGEPRQVVLQGSIDF
yinte0001_28810  -----------------------------------------------------
yfred0001_27640  -----------------------------------------------------
yaldo0001_28650  --AWLYRLAEWR-----------------------------------------
yberc0001_26380  FVSYSLPINHYRVKWQLNVKKLFDKTYYPSSGGNLRIAVGEPRQFVLRASIDF
yaldo0001_28640  FISYSLPVNHYWVKWPLNVKNLFDKTYYLSSGGNLRVAVGEPR----------
yaldo0001_28660  -----------------------------------------------------
yruck0001_22100  FIAYTLPIQNYRVKWQLNVKNLFDKTYYPSSGNSYRVAIGEPRQFVLRANVDF
ykris0001_27690  FISYSLPINNYRVKWQLNVKNFFDKTYYPSSGGNLRVAVGEPRQFVLRASVDF
yente0001X_1070  FISYSLPISNYRVKWQLNVKNFLDKTYYPSSGGNLRVAVGEPRQFVLRASVDF
```

```
Parameters used
Minimum Number Of Sequences For A Conserved Position: 6
Minimum Number Of Sequences For A Flanking Position: 9
Maximum Number Of Contiguous Nonconserved Positions: 8
Minimum Length Of A Block: 10
Allowed Gap Positions: With Half
Use Similarity Matrices: Yes
```

```
Flank positions of the 0 selected block(s)
Flanks: 

New number of positions in PGL1_unique_yersinia-CLUSTERS.dir/PGL1_unique_yersinia-CL1259/PGL1_unique_yersinia-CL1259.muscle.fasta.gblo:  0  (0% of the original 713 positions)
```
