# Supplementary material for: Genomic characterization of the Yersinia genus
Source: Genome Biol. 2010 Jan 4;11(1):R1. doi: 10.1186/gb-2010-11-1-r1 (PMC2847712; doi:10.1186/gb-2010-11-1-r1)
Supplement: Additional file 16 — The top level directory consists of a directory called Additional_cluster_files and 5010 directories, one for each multi-protein cluster family. (This top level directory has been split into three data files for uploading purposes (Additional files 15, 16, 17.) Within the directory are the following files: PGL1_unique_Yersinia_unclustered.out - list of all protein singletons that MCL did not group into a cluster (see Materials and Methods); PGL1_Yersinia_unique_locus_tags.txt - names of the 11 locus tag prefixes used for each genome; PGL1_unique_Yersinia.gff - mapping each Yersinia protein to a cluster in tab delimited GFF; PGL1_unique_Yersinia.sigfile - list of the longest protein in each cluster; PGL1_unique_Yersinia.summary - summary table of features of each of the clusters; PGL1_unique_Yersinia.table - summary table of each protein in the clusters. Within each cluster directory are the following files, where 'x' is the cluster name: PGL1_unique_Yersinia-x.faa - multifasta file of the proteins in the cluster; PGL1_unique_Yersinia-x.summary - summary of the properties of the proteins; PGL1_unique_Yersinia-x.matches - blast matches between the proteins of the cluster; PGL1_unique_Yersinia-x.muscle.fasta - muscle alignment of the proteins; PGL1_unique_Yersinia-x.muscle.fasta.gblo - gblocks output of muscle alignment (that is, auto-trimmed alignment); PGL1_unique_Yersinia-x.muscle.fasta.gblo.htm - as above in html format; PGL1_unique_Yersinia-x.muscle.tree - treefile from muscle alignment; PGL1_unique_Yersinia-x.sif - matches between proteins in simple interaction format for display on graphing software. [file gb-2010-11-1-r1-S16.zip › clusters2/PGL1_unique_yersinia-CL1260/PGL1_unique_yersinia-CL1260.muscle.fasta.gblo.htm]

PGL1\_unique\_yersinia-CL1260.muscle.fasta


## Gblocks 0.91b Results

Processed file: **PGL1\_unique\_yersinia-CL1260.muscle.fasta**  
Number of sequences: **11**  
Alignment assumed to be: **Protein**  
New number of positions: **620** (selected positions are underlined in blue)

```
                         10        20        30        40        50        60
                 =========+=========+=========+=========+=========+=========+
yruck0001_6600   ----LEQAVSDKLLRPLDVQFARMIARDDEPLMQLAAAYLSAETGAGHVCLSLDRLHPDR
ypseu0001X_3383  MMTLLAQAARDRLLRPLDVQFSRMIAGDDDPRLQLAAAILSAEVGAGHVCLPLRYLQPEL
ypest0001X_1230  MMTLLAQAARDRLLRPLDVQFSRMIAGDDDPRLQLAAAILSAEVGAGHVCLPLRYLQPEL
yaldo0001_8620   MMALLAQAVRDHLLRPLDLQFSRMIAGDDDPQLQLAAAILSAEAGAGHVCLPISCLQPAQ
ymoll0001_7820   MMALLNQAVGDHLLRPLDLQFSRMIAGDNDPMLQLAAAILSAETGAGHVCLPLNYLQPDQ
yberc0001_6860   MIALLNQAVGNHLLRPLDLQFSLMIAGDNDPMLQLAAAILSAEAGAGHVCLPLSYLQPDQ
yinte0001_8540   MMALLDQAVRDHLLRPLDVQFSRMIAGDDGPMLQLAAAILSAEAGAGHVCLPLSYLQPEQ
yrohd0001_8560   MMRLLEQAVRDHLLRPLDIQFSRMIAGENDPMLQLAAAILSAEAGAGHVCLPLSYLQPNQ
yfred0001_43730  MMALLDQAVRDHLLRPLDVQFSRMIAGEDNPMLQLAAAILSAEAGAGHVCLPLSYLQPDE
ykris0001_8180   MMTLLEQAVRDHLLRPLDIQFSRMIAGDNAPMLQLAAAVLSAEAGAGHVCLPLSYLQPDQ
yente0001X_9440  MMTLLEQAVRDHLLRPLDVQFSRMIAGDNDPMLQLAAAVLSAEAGAGHVCLPLSYLQPDQ
                 ############################################################


                         70        80        90       100       110       120
                 =========+=========+=========+=========+=========+=========+
yruck0001_6600   LFGGRQPLLAQSLWQAAGKPELVQWQAGLRASDAVSDGSLPTPLVLQQDRLYLQRMWQCE
ypseu0001X_3383  LFGGRQPDLSLALWQAAGSPDKAQWLQALQNAPVVSDGSQPTPLVLQQERLYLQRMWQYE
ypest0001X_1230  LFGGRQPDLSLALWQAAGSPDKAQWLQALQNAPVVSDGSQPTPLVLQQERLYLQRMWQYE
yaldo0001_8620   FFGGRQPDLSQALWQAAGAPDKPRWLQVLQRSPAVSDGSQPTPLVLQNERLYLQRMWQYE
ymoll0001_7820   LFGGRQSALSQALWQAAGAPDESHWIAAFNNSAAVSDGSKPTPLVLQNGRLYLQRMWQYE
yberc0001_6860   LFGGRQSALSQALWQAAGAPDESCWIQAFNHSPAVSDGSQPTPLVLQNDRLYLQRMWQYE
yinte0001_8540   LFGGRQAGLSQTLWQAAGAPDLSRWIQVLNNSPAVSDGSLPTPLVLQNERLYLQRMWQYE
yrohd0001_8560   LLGGRHSELSQALWQAAGAPDTLQWSLALNNSAAISDGSQPTPLVLQNDRLYLQRMWQYE
yfred0001_43730  LFGGRQPSLSQALWQAAGSPNKLLWIQSLNSSPAVSDGARPTPLVLQNDRLYLQRMWQYE
ykris0001_8180   LFGGRQPALAQALWQAIGAPDQLHWMQTLNNSPAVSDGTLPTPLVLQNDRLYLQRMWQYE
yente0001X_9440  LFGGRQPALSQALWQAVGAPDQLHWIQKLNSSPAVSDGSQPTPLVLQNDRLYLQRMWQYE
                 ############################################################


                        130       140       150       160       170       180
                 =========+=========+=========+=========+=========+=========+
yruck0001_6600   GEVAEFISHAEANEVID------KEK----------LKDILNNLFGASSTEIDWQKVAAA
ypseu0001X_3383  GDVVQFIASDSV--FINRDSDFMTSKGIATSVDESLLRETLDALFGCAGSEVDWQKVAAA
ypest0001X_1230  GDVVQFIASDSV--FINRDSDFMTSKGIATSVDESLLRETLDALFGCAGSEVDWQKVAAA
yaldo0001_8620   GDVVRFIASDSLITATN------ESL----LIDEPLLKETLDRLFGVATREVDWQKVAAA
ymoll0001_7820   GDVVRFIASDSTAAWVN------EPR----EVNETLLRVTLDQLFGLAGDEVDWQKVAAA
yberc0001_6860   GDVVRFIASDSTAAWAN------EPR----EVNEALLRTTLDQLFGLAESEVDWQKVAAA
yinte0001_8540   GDVVRFIASDNTALFVN------EAR----DVNENLLTETLDRLFGRSGTEIDWQKVAAA
yrohd0001_8560   GDVVRFIASDNSATFIN------ETR----EVNETLLRETLDRLFGVAGTEVDWQKVAAA
yfred0001_43730  GDVVRFIASDNAASFIT------ETR----VVNETLLRETLDGLFGGTGPEVDWQKVAAA
ykris0001_8180   GDVVRFMASDNTASFIN------ETR----DVNEALLRETLDRLFGPASTEVDWQKLAAA
yente0001X_9440  GDVVRFIASDNMASLINETSDGSETR----DVNETLLRDTLDRLFGPAENEVDWQKVAAA
                 #################              #############################


                        190       200       210       220       230       240
                 =========+=========+=========+=========+=========+=========+
yruck0001_6600   VAVTRRISVISGGPGTGKTTTVAKLLTALVQLNEGRRLRIQLAAPTGKAAARLTESLGKA
ypseu0001X_3383  VAATRRISVISGGPGTGKTTTVAKLLTALIRLSQGQRLRIKLAAPTGKAAARLTESLGKA
ypest0001X_1230  VAATRRISVISGGPGTGKTTTVAKLLTALIRLSQGQRLRIKLAAPTGKAAARLTESLGKA
yaldo0001_8620   VAVTRRISVISGGPGTGKTTTVARLLVALIQLNSAQRLRIQLAAPTGKAAARLTESLGKA
ymoll0001_7820   VAATRRISVISGGPGTGKTTTVAKLLTALIQLNQGQRLRIQLAAPTGKAAARLTESLGNA
yberc0001_6860   VAATRRISVISGGPGTGKTTTVAKLLTALIQLSQGQRLRIQLAAPTGKAAARLTESLGNA
yinte0001_8540   VAATRRISVISGGPGTGKTTTVAKLLTALIQLSPGQRLRIQLAAPTGKAAARLTESLGNA
yrohd0001_8560   VAATRRISVISGGPGTGKTTTVAKLLTALIQLSQGQRLRIQLAAPTGKAAARLTESLGNA
yfred0001_43730  VAATRRISVISGGPGTGKTTTVAKLLTALIQLSQGQRLRIQLAAPTGKAAARLTESLGNA
ykris0001_8180   VAATRRISVISGGPGTGKTTTVAKLLTALIQLSQGQRLRIQLAAPTGKAAARLTESLGKA
yente0001X_9440  VAATRRISVISGGPGTGKTTTVAKLLTALIQLSKGQRLRIQLAAPTGKAAARLTESLGNA
                 ############################################################


                        250       260       270       280       290       300
                 =========+=========+=========+=========+=========+=========+
yruck0001_6600   SRQLPLSESERKLFPDQASTLHRLLGAQPNSQRLRYHQGNPLNLDVLVVDEASMVDLPMM
ypseu0001X_3383  IRQFSLTDDERKLFPDQASTLHRLLGVQPNSQRLRYHRGNPLNLDVLVVDEASMVDLPMM
ypest0001X_1230  IRQFFLTDDERKLFPDQASTLHRLLGVQPNSQRLRYHRGNPLNLDVLVVDEASMVDLPMM
yaldo0001_8620   IRQLALNDNERKLIPDQASTLHRLLGAQPNSQRLRFHQGNPLSLDVLVVDEASMVDLPMM
ymoll0001_7820   IRQLALTDDERRLFPDQASTLHRLLGAQPNSQRLRYHRGNPLNLDVLVVDEASMVDLPMM
yberc0001_6860   IRQLPLTDDERKLLPDQASTLHRLLGAQPNSQRLRYHRGNPLNLDVLVVDEASMVDLPMM
yinte0001_8540   IRQLTLTDAERKLLPDQASTLHRLLGAQPNSQRLRYHRGNPLNLDVLVVDEASMVDLPMM
yrohd0001_8560   IRQLPLTEADLKLFPEQASTLHRLLGAQPNSQRLRYHQGNPLNLDVLVVDEASMVDLPMM
yfred0001_43730  MRQLPLTDDERQLFPDQASTLHRLLGAQPNSQRLRYHRGNPLNLDVLVVDEASMVDLPMM
ykris0001_8180   IRQLSLTDDERKLFPDQASTLHRLLGAQPNSQRLRYHRGNPLNLDVLVVDEASMVDLPMM
yente0001X_9440  IRQLSLTDDERKLFPDQASTLHRLLGAQPNSQRLRYHRGNPLNLDVLVVDEASMVDLPMM
                 ############################################################


                        310       320       330       340       350       360
                 =========+=========+=========+=========+=========+=========+
yruck0001_6600   AKLIAALPAKAQVIFLGDRDQLASVEAGAVLGDICRLVESGYSPTRAEELSELTGC----
ypseu0001X_3383  ARLIAALPAKAKVIFLGDRDQLASVEAGAVLGDICRFAELGYSEQRAQQLTQLTGYLLTN
ypest0001X_1230  ARLIAALPAKAKVIFLGDRDQLASVEAGAVLGDICRFAELGYSEQRAQQLTQLTGYLLTN
yaldo0001_8620   ARLIAALPAKARVIFLGDRDQLASVEAGAVLGDICRFAELGYSDSRAEQLTRLTGY----
ymoll0001_7820   ARLIAALPAKAQVIFLGDRDQLASVEAGAVLGDICRFAEWGYSESRAEQLARLTGC----
yberc0001_6860   ARLIAALPAKAQVIFLGDRDQLASVEAGAVLGDICRFAEWGYSESRAEQLVRLTGCALTD
yinte0001_8540   ARLIAALPAKAQVIFLGDRDQLASVEAGAVLGDICRFAELGYSKARAEQLARLTGC----
yrohd0001_8560   ARLIAALPAKAQVIFLGDRDQLASVEAGAVLGDICRFAELGYSQVRAEQLTRLTGC----
yfred0001_43730  ARLIAALPAKAQVIFLGDRDQLASVEAGAVLGDICRFAELGYSAARAEQLARLTGC----
ykris0001_8180   ARLIAALPAKAQVIFLGDRDQLASVEAGAVLGDICRFAELGYSESRAEQLTRLTGC----
yente0001X_9440  ARLIAALPTKAQVIFLGDRDQLASVEAGAVLGDICRFAELGYSAPRAKQLARLTGC----
                 #######################################################     


                        370       380       390       400       410       420
                 =========+=========+=========+=========+=========+=========+
yruck0001_6600   -----------SLQGKVA-----EKTATIRDSLCLLRKSYRFDEQSGIGQLALAVNAGNP
ypseu0001X_3383  NALTSNVLTNKALANKAQPDETYSDSANVRDSLCLLRKSYRFDEKSGIGQLALAVNAGEY
ypest0001X_1230  NALTSNVLTNKALANKAQPDETYSDSANVRDSLCLLRKSYRFDEKSGIGQLALAVNAGEY
yaldo0001_8620   -----------ALSGEIPVGRSDKDATNVRDSLCLLRKSYRFDEKSGIGQLALAVNAGQY
ymoll0001_7820   -----------TLTGIIPIGDVPTDTVNVRDSLCLLRKSYRFDEKSGIGQLALAVNAGKY
yberc0001_6860   SRMIDSTLIDSTLTGIIPIGDVPKDTLNVRDSLCLLRKSYRFDEKSGIGQLALAVNAGKY
yinte0001_8540   -----------TLAGNIPIGAVPTDTVNVRDSLCLLRKSYRFDEKSGIGQLALAVNAGKY
yrohd0001_8560   -----------TLTGSIPIGDVQTDTVNVRDSLCLLRKSYRFDEKSGIGQLALAINAGRY
yfred0001_43730  -----------TLSGHIPIGDVQTDTVNVRDSLCLLRKSYRFDEKSGIGQLALAVNAGKY
ykris0001_8180   -----------TLAGNIPIGDVQTDTVNVRDSLCLLRKSYRFDEKSGIGQLALAVNAGRY
yente0001X_9440  -----------TLVGNIPIGEVETDTVNVRDSLCLLRKSYRFDEKSGIGQLALAVNAGRY
                             ################################################


                        430       440       450       460       470       480
                 =========+=========+=========+=========+=========+=========+
yruck0001_6600   KQALSVFQGNYADIERFPLSDTEDYQRLLQDSVAGYQHYLQRVAEEANPVAILTEFSRYQ
ypseu0001X_3383  RQALSVLNSAYSDVERFPLADEEDYQVLLEACAVGYQHYLERVAALAPAAEVLAAFGRYQ
ypest0001X_1230  RQALSVLNSAYSDVERFPLADEEDYQVLLEACAVGYQHYLERVAALAPAAEVLAAFGRYQ
yaldo0001_8620   REALSVLNGTYRDVERFPLVDTDDYQVLLEASMTGYQHYLQLAAAGMDASEVLAAFGRYQ
ymoll0001_7820   HHALSVLNGTYSDVECFSLADSDDYQVLLEECASGYQHYLQLASSGAHAVDVLAAFGRYQ
yberc0001_6860   HNALSVLSGTYSDIECFSLADSDDYQVLLEDCAASYQHYLQLATSGAHAVDVLAAFGRYQ
yinte0001_8540   RDALSVFKGAYSDVEHFSLTDSDDYQRLLEDCVAGYQHYLQLAATGAHAADVLAAFGRYQ
yrohd0001_8560   RDALSVLNGTYSDIKLLSLADADDYQVLLEDCAAGYQHYLQLATAGAPAAEVLAAFGRYQ
yfred0001_43730  RDALSVLSGTYSDIERFPLVDTDDYQVLLEDCAAGYQHYLQLATSGAHATDVLAAFGRYQ
ykris0001_8180   RDALSVLNGAYSDIENFSLADSDDYQVLLEDCAAGYRHYLELAAAGVHAVEVLAAFGRYQ
yente0001X_9440  RDALAVFNGTYSDIERFSLADSDDYQVLLEDCAAGYQHYLELAAAGADAVDVLAAFGRYQ
                 ############################################################


                        490       500       510       520       530       540
                 =========+=========+=========+=========+=========+=========+
yruck0001_6600   LLCALRTGPFGVGGLNERIEQMLNKKGLIRRIPGPSGRWYPGRPIMIDRNDSALGLFNGD
ypseu0001X_3383  LLCALRSGPFGVSGLNERIEQVLHRKGFIIRPSGPSGRWYVGRPVMIELNDSALGLFNGD
ypest0001X_1230  LLCALRSGPFGVSGLNERIEQVLHRKGFIIRPSGPSGRWYVGRPVMIELNDSALGLFNGD
yaldo0001_8620   LLCALRTGPFGVSGLNERIEQLLHRNRLIDRAPGPSGRWYVGRPVMIGLNDSALGLFNGD
ymoll0001_7820   LLCALRTGPFGVSGLNERIEQLLHRKRLIERASGPVGRWYVGRPVMIGLNDSALGLFNGD
yberc0001_6860   LLCALRTGPFGVSGLNERIEQLLHRKRLIERASGPAGRWYVGRPVMIGLNDSALGLFNGD
yinte0001_8540   LLCALRSGPFGVSGLNERIEQLLHHKRLIERAPGPTGRWYAGRPVMIGLNDSALGLFNGD
yrohd0001_8560   LLCALRSGPFGVSGLNERIEQLLHRKRLIERTPGPSGRWYLGRPVMIGLNDSALGLFNGD
yfred0001_43730  LLCALRTGPFGVSGLNERIEQLLHRQRFIERTPGPSGRWYVGRPVMIGLNDSALGLFNGD
ykris0001_8180   LLCALRSGPFGVSGLNERIEQLLHRKRLIERTPGPSGRWYVGRPVMIGLNDSALGLFNGD
yente0001X_9440  LLCALRAGPFGVSGLNERIEQLLHRKRLIERTPGPSGRWYVGRPVMIGLNDSALGLFNGD
                 ############################################################


                        550       560       570       580       590       600
                 =========+=========+=========+=========+=========+=========+
yruck0001_6600   IGIALYDADGELRVHFHLPDGSIKSVQPSRLPSHETAYVMTVHKSQGSEFEHTALILPNY
ypseu0001X_3383  IGIALHDNEGELRVYFQLPDGNIKSVQPSRLPSHETAYAMTVHKSQGSEFEHTALVLPNT
ypest0001X_1230  IGIALHDNEGELRVYFQLPDGNIKSVQPSRLPSHETAYAMTVHKSQGSEFEHTALVLPNT
yaldo0001_8620   IGIALLDCEGELRVHFQLPDGTIKSVQPSRLPSHETAYAMTVHKSQGSEFEHTALVLPGT
ymoll0001_7820   IGIALYDPEGELRVHFQLPDGSIKSVQPSRLPSHETAYAMTVHKSQGSEFEHTALVLPNT
yberc0001_6860   IGIALYDSEGELRVHFQLPDGNIKSVQPSRLPTHETAYAMTVHKSQGSEFEHTALVLPNT
yinte0001_8540   IGIALYDGEGELRVHFQLPDGNIKSVQPSRLPSHETAYAMTVHKSQGSEFEHTALVLPNT
yrohd0001_8560   IGIALNDPEGELRVHFQLPDGNIKSVQPSRLPSHETAYAMTVHKSQGSEFEHTALVLPNT
yfred0001_43730  IGIALNDSEGELRVYFQLPDGNIKSVQPSRLPSHETAYAMTVHKSQGSEFEHTALVLPNT
ykris0001_8180   IGIALYDSEGELRVHFQLPDGNIKSVQPSRLPSHETAYAMTVHKSQGSEFEHTALVLPNT
yente0001X_9440  IGIALLDPEGELRVHFQLPDGNIKSVQPSRLPSHETAYAMTVHKSQGSEFEHTALVLPNT
                 ############################################################


                        610       620       630       640       650
                 =========+=========+=========+=========+=========+======
yruck0001_6600   FLPVVTRELVYTAITRARKRLTLYCSDSVLTSAIRTPTQRRSGLVERLMQ------
ypseu0001X_3383  FMPVLTRELVYTAITRARQHLTLYCSDAVLSHAIRTPTLRLSGLVDRLNTLNRQ--
ypest0001X_1230  FMPVLTRELVYTAITRARQHLTLYCSDAVLSHAIRTPTLRLSGLVDRLNTLNRQ--
yaldo0001_8620   FMPVLTRELVYTAITRARERLTLYCNDTVLSSAIRTPTQRLSGLVDRLNEIRRVTK
ymoll0001_7820   FMPVLTRELVYTAITRARQRLTLYCSEAVLSNAIRTPTQRRSGLVDRLNELTK---
yberc0001_6860   FMPVLTRELVYTAITRARQRLTLYCSDTVLSHAIRTPTQRRSGLVDRLNELNK---
yinte0001_8540   FMPVLTRELVYTAITRARQRLTLYCSDTVLSNAIRTPTLRLSGLVDRLNALT----
yrohd0001_8560   FMPVLTRELVYTAITRARQRLTLYCSDGVLSQAIRTPTLRLSGLVERLNELKKLG-
yfred0001_43730  FMPVLTRELVYTAITRARQHLTLYCSDAVLGSAIRTPTLRLSGLVDRLNELKK---
ykris0001_8180   FMPVLTRELVYTAITRARQRLTLYCSDTVLSHAIRTPTHRVSGLVERLNQLT----
yente0001X_9440  FMPVLTRELVYTAITRARQRLTLYCSDTVLSHAIRTPTLRVSGLVDRLNQLK----
                 ###################################################
```

```
Parameters used
Minimum Number Of Sequences For A Conserved Position: 6
Minimum Number Of Sequences For A Flanking Position: 9
Maximum Number Of Contiguous Nonconserved Positions: 8
Minimum Length Of A Block: 10
Allowed Gap Positions: With Half
Use Similarity Matrices: Yes
```

```
Flank positions of the 3 selected block(s)
Flanks: [1  137]  [152  355]  [373  651]  

New number of positions in PGL1_unique_yersinia-CLUSTERS.dir/PGL1_unique_yersinia-CL1260/PGL1_unique_yersinia-CL1260.muscle.fasta.gblo:  620  (94% of the original 656 positions)
```
