# Supplementary material for: Genomic characterization of the Yersinia genus
Source: Genome Biol. 2010 Jan 4;11(1):R1. doi: 10.1186/gb-2010-11-1-r1 (PMC2847712; doi:10.1186/gb-2010-11-1-r1)
Supplement: Additional file 16 — The top level directory consists of a directory called Additional_cluster_files and 5010 directories, one for each multi-protein cluster family. (This top level directory has been split into three data files for uploading purposes (Additional files 15, 16, 17.) Within the directory are the following files: PGL1_unique_Yersinia_unclustered.out - list of all protein singletons that MCL did not group into a cluster (see Materials and Methods); PGL1_Yersinia_unique_locus_tags.txt - names of the 11 locus tag prefixes used for each genome; PGL1_unique_Yersinia.gff - mapping each Yersinia protein to a cluster in tab delimited GFF; PGL1_unique_Yersinia.sigfile - list of the longest protein in each cluster; PGL1_unique_Yersinia.summary - summary table of features of each of the clusters; PGL1_unique_Yersinia.table - summary table of each protein in the clusters. Within each cluster directory are the following files, where 'x' is the cluster name: PGL1_unique_Yersinia-x.faa - multifasta file of the proteins in the cluster; PGL1_unique_Yersinia-x.summary - summary of the properties of the proteins; PGL1_unique_Yersinia-x.matches - blast matches between the proteins of the cluster; PGL1_unique_Yersinia-x.muscle.fasta - muscle alignment of the proteins; PGL1_unique_Yersinia-x.muscle.fasta.gblo - gblocks output of muscle alignment (that is, auto-trimmed alignment); PGL1_unique_Yersinia-x.muscle.fasta.gblo.htm - as above in html format; PGL1_unique_Yersinia-x.muscle.tree - treefile from muscle alignment; PGL1_unique_Yersinia-x.sif - matches between proteins in simple interaction format for display on graphing software. [file gb-2010-11-1-r1-S16.zip › clusters2/PGL1_unique_yersinia-CL1261/PGL1_unique_yersinia-CL1261.muscle.fasta.gblo.htm]

PGL1\_unique\_yersinia-CL1261.muscle.fasta


## Gblocks 0.91b Results

Processed file: **PGL1\_unique\_yersinia-CL1261.muscle.fasta**  
Number of sequences: **11**  
Alignment assumed to be: **Protein**  
New number of positions: **317** (selected positions are underlined in blue)

```
                         10        20        30        40        50        60
                 =========+=========+=========+=========+=========+=========+
yruck0001_33330  ----------------------------------------------------------LL
ypseu0001X_3742  ---------------------------------------MFLYMTYIERKIRHSLFISLG
ypest0001X_9070  ---------------------------------------MFLYMTYIERKIRHSLFISLG
yente0001X_6220  ---------------------------MRGVKIERENAIMSFYIKYTQHKSWRSILPGLG
yaldo0001_6740   -------------------------------------------MLLISHKTSCFLLTTFA
yrohd0001_6520   ------------------------------------------MSLDIQHKISRSRFISFG
yfred0001_6570   ---------------------------------------MSLAIHHVQHKKFRSVLTGLG
ykris0001_5140   VVNYDLALTLSINVNVFINKSYLYIPPELGLKIERENAVMSFDINYIQHKKWRSTLTKLG
yinte0001_6930   ------------------------------------------------------------
yberc0001_38990  ------------------------------------------------------------
ymoll0001_36350  ---------------------------------------MSRYAPPISPKTARFVLLLLS
                                                                             


                         70        80        90       100       110       120
                 =========+=========+=========+=========+=========+=========+
yruck0001_33330  ALFTIFLLSGCKPADETVDVSPPAATSKNSEL-WPRIVRTAKGDIQISHPPRRIVSTSIT
ypseu0001X_3742  CLGLILSLSGCEPAEETNFHPFPIDENHSTSSPWSRTVDTVKGPVTLTHKPARIVSTSIT
ypest0001X_9070  CLGLILSLSGCEPAEETNFHPFPIDENHSTSSPWSRTVDTVKGPVTLTHKPARIVSTSIT
yente0001X_6220  LLGLIFCLSGCKPADDTTSEQAPSSSSQNATT-WTRTVETAKGPVTLTQQPKRIVSTSIT
yaldo0001_6740   CLCLILGLSGCKPAEESSI----STASENTSA-WSRTVETAKGPVTLTHQPQRIVSTSIT
yrohd0001_6520   FFALIFCLSGCKPAEETTGKPAPSSANDSTAT-WSRTVETAKGPVTLTHQPIRIVSTSIT
yfred0001_6570   ILSLILCLSACKPAEETTSKPASSATSENTST-WSRTVETATGPVTLTHQPTRIVSTSIT
ykris0001_5140   LFGLVLCLSACKPAEETKDQQAPSSGSEGTAA-WTRTVETAKGPVVLTHPPTRIVSTSIT
yinte0001_6930   -------------------------------------VETAKGPVTLIQQPTRIVSTSIT
yberc0001_38990  -LTLTFSLSGCKPAQEAET----SAAAKDTPA-WSRTVETAKGPVTLTSQPQRIVSTSIT
ymoll0001_36350  FLALTLGLSGCKPAQEAET----SAAAEDTPA-WSRTVETAKGPVTLTQQPKRIVSTSIT
                    ###########################   ###########################


                        130       140       150       160       170       180
                 =========+=========+=========+=========+=========+=========+
yruck0001_33330  MTGTLLAINAPLVGTGATVPNTTVADDQGFFTQWSKEAKARNLVPMYQTEPNAEAVASMA
ypseu0001X_3742  ITGTLLAINAPIIASSATEPNTTLADNKGFFTQWSDIARARHLIPMYQTEPNIKAVAKMR
ypest0001X_9070  ITGTLLAINAPIIASSATEPNTTLADNKGFFTQWSDIARARHLIPMYQTEPNIKAVAKMR
yente0001X_6220  ITGTLLAINAPVIASGATVPDTTVADNQGFFTQWSEVAQAKKLVPIYQTEPNAEAVAGMN
yaldo0001_6740   ITGTLLAINAPIIASGATMPNTTVADAQGFFTQWSDVAQARHLVPIYQTEPNAEAVAGMN
yrohd0001_6520   ITGTLLAINAPVIASGATVPDTTVADNQGFFTQWSDVAQSRKLVPMYHTEPNAEAVAGMD
yfred0001_6570   ITGTLLAINAPVIATGATVPDTTVADHQGFFTQWSDVAQAKKLVPMYQTEPNAEAVAGMN
ykris0001_5140   ITGTLLAINAPVIASGATVPDTTVADHQGFFTQWSDVAQAKKLVPMYQTEPNAEAVAGMN
yinte0001_6930   ITGTLLAINAPVIASGATAPNSTVADNQGFFTQWSDVAQTKNLVPIYQTEPNAEAVAGMN
yberc0001_38990  ITGTLLAINAPVIATGATAPNTTVADNQGFFIQWSDVAQAKKLVPLYQTEPNAEAVAGMN
ymoll0001_36350  ITGTLLAINAPVIATGATAPNTTVADKQGFFTQWSEVAQAKKLVPLYQTEPNAEAVAGMN
                 ############################################################


                        190       200       210       220       230       240
                 =========+=========+=========+=========+=========+=========+
yruck0001_33330  PDMIIISATGGDSALKLYEQLSAIAPTLVINYDDKSWQELAQVLGQATGHEADAERVITD
ypseu0001X_3742  PDLIIISATGDDSTLELYDQLSAIAPTLVINYDDKSWQELTLQLGQATGHEGDAEQVIDK
ypest0001X_9070  PDLIIISATGDDSTLELYDQLSAIAPTLVINYDDKSWQELTLQLGQATGHEGDAEQVIDK
yente0001X_6220  PDLIIISATGGDSALKLYEQLSVIAPTLVINYDDKSWQELAVLLGQATGHEADAQQVIAT
yaldo0001_6740   PDLIIISATGGDSALKLYEQLSAIAPTLVINYDDKSWQQLAILLGQATGHEADAEQVIAK
yrohd0001_6520   PDLIIISATGGDSALKLYEQLSVIAPTLVINYDDKSWQELALVLGQATGHEKDAQQVIDK
yfred0001_6570   PDLIIISATGGDSALKLYEQLSAIAPTLVINYDDKSWQELAIVLGQATGHEADAQQVIDN
ykris0001_5140   PDLIIISATGGDSALKLYEQLSAIAPTLVINYDDKSWQELAIVLGQATGHEADAQQIIEK
yinte0001_6930   PDLIIISATGGDSAVKLYEQLSAIAPTLVINYDDKSWQELAMILGQATGHETDAKEVIDK
yberc0001_38990  PDLIIISATGGDSALKLYEQLSAIAPTLVINYDDKSWQELAILLGQATGHESDATQVIDS
ymoll0001_36350  PDLIIISATGGDSALKLYEQLSTIAPTLVINYDDKSWQALAVILGQATGHESDATQVIDK
                 ############################################################


                        250       260       270       280       290       300
                 =========+=========+=========+=========+=========+=========+
yruck0001_33330  FSQQMEQVKSSITLPPQPVSAFVYQPGGQTANMWTENSAQGKLLQELGFRLAEIPEDVRG
ypseu0001X_3742  FARRLNEVKQKITLPPQPVSAFVYQEVSGSAKLWTENSAQGRLLLELGFTLANVPDTLKD
ypest0001X_9070  FARRLNEVKQKITLPPQPVSAFVYQEVSGSAKLWTENSAQGRLLLELGFTLANVPDTLKD
yente0001X_6220  FTHRLNEVKQNITLPPQPTSAFVYQAADSTANLWTENSAQGKLLQELGFTLAQVPDAVKG
yaldo0001_6740   FTRRVNEVKQNITLPPQPVSAFVYQAAGSTANLWTDSSAQGKLLQELGFTLANVPDAVKG
yrohd0001_6520   FANRLNEVKQNITLPPQPVSAFVYQSAGSTANLWTEFSAQGKLLQDLGFTLAKIPDAVKG
yfred0001_6570   FTRRLNEVKQNITLPPQPTSAFVYQAAGSTANLWTEDSAQGKLLQELGFTLAEVPDAVKG
ykris0001_5140   FTNRINEVKQNITLPPQPTSAFVYQAAGSTANLWTESSAQGKLLQELGFTLAEVPDAVKG
yinte0001_6930   FANRLNEVKQNITLPPQPTSAFVYQAAGSTANVWTEDSAQGKLLQALGFRLAEIPETVKG
yberc0001_38990  FANRLNEVKQNITLPPQPTSAFVYQAAGSTANLWTDNSAQGKLLQQLGFTLATIPDAVKG
ymoll0001_36350  FANRLNEVKQTITLPPQPTSAFVYQAAGNTANLWTENSAQGKLLQQLGFTLATIPDAVKG
                 ############################################################


                        310       320       330       340       350       360
                 =========+=========+=========+=========+=========+=========+
yruck0001_33330  NTSMGYRKDIVQLGGERLAEGLNGNTFLLFSGDHLAIEALKANQFLVHLTPLQQDRVYPM
ypseu0001X_3742  NTHTEHRKDIIQLGGDELAQGLNGKTILLFSGVHSAIDALKSNKFLTNLPAIQHDNVYAA
ypest0001X_9070  NTHTEHRKDIIQLGGDELAQGLNGKTILLFSGVHSAIDALKSNKFLTNLPAIQHDNVYAA
yente0001X_6220  NTSMGHRKDIIQLGGEKLAEGLNGETILLFSGDQPAIDALKSNKFLAHTPAIEHNRVYAA
yaldo0001_6740   NTSMGHRKDIIQLGGEKLAEGLNGETVLLFSGQQPAVDALKSNKFLAHIPAIEHHRVYAA
yrohd0001_6520   NTSMGHRKDIIQLGGEKLAEGLNGETILLFSGDQPAIDALKSNKFLAHIPAIEHHRVYAA
yfred0001_6570   NTSMGHRKDIIQLGGEKLAEGLNGETILLFSGDQPAIDALKSNKFLSHIPAIEHQRVYAA
ykris0001_5140   NTSMGHRKDIIQLGGEKLAEGLNGETILLFSGDQPAIDALKSNKFLAHIPAIEHDRVYAA
yinte0001_6930   NTSMGHRKDIVQLGGEKLAEGLSGETILLFSGDQPSVDALKSNKFLAHIPAIEHNRVYAV
yberc0001_38990  NTSMGHRKDIIQLGGEKLAEGLNGETILLFSGERPAVDALKSNQFLAHIPAIEHNRVYAV
ymoll0001_36350  NTSMGHRKDIIQLGGEKLAEGLNGETILLFSGDQPAVEALKSNQFLAHTPAVEHNRVYAV
                 ############################################################


                        370       380       390
                 =========+=========+=========+========
yruck0001_33330  GNDTFRLDYYSASNMLQRIKTLFNKD------------
ypseu0001X_3742  GYDTFRLDYYSANNLLTRIESMFKGKKDSEPTYHESRT
ypest0001X_9070  GYDTFRLDYYSANNLLTRIESMFKGKKDSEPTYHESRT
yente0001X_6220  GYDTFRLDYYSASKLLARLEEMFKAKS-----------
yaldo0001_6740   GYDTFRLDYYSASNFLTRIERMFKGSQ-----------
yrohd0001_6520   GYDTFRLDYYSASNLLARIEGMFKAKPPAL--------
yfred0001_6570   GYDTFRLDYYSASNLLARIEGMFKTKP-----------
ykris0001_5140   GYDTFRLDYYSASNLLARIEGMFKTKP-----------
yinte0001_6930   GYDTFRLDYYSASNLLARIEGMFTPPH-----------
yberc0001_38990  GYDTFRLDYYSASHLLARIEGMFKG-------------
ymoll0001_36350  GYDTFRLDYYSASNLLARIEGMFTAKP-----------
                 #######################
```

```
Parameters used
Minimum Number Of Sequences For A Conserved Position: 6
Minimum Number Of Sequences For A Flanking Position: 9
Maximum Number Of Contiguous Nonconserved Positions: 8
Minimum Length Of A Block: 10
Allowed Gap Positions: With Half
Use Similarity Matrices: Yes
```

```
Flank positions of the 2 selected block(s)
Flanks: [64  90]  [94  383]  

New number of positions in PGL1_unique_yersinia-CLUSTERS.dir/PGL1_unique_yersinia-CL1261/PGL1_unique_yersinia-CL1261.muscle.fasta.gblo:  317  (79% of the original 398 positions)
```
