# Supplementary material for: Genomic characterization of the Yersinia genus
Source: Genome Biol. 2010 Jan 4;11(1):R1. doi: 10.1186/gb-2010-11-1-r1 (PMC2847712; doi:10.1186/gb-2010-11-1-r1)
Supplement: Additional file 16 — The top level directory consists of a directory called Additional_cluster_files and 5010 directories, one for each multi-protein cluster family. (This top level directory has been split into three data files for uploading purposes (Additional files 15, 16, 17.) Within the directory are the following files: PGL1_unique_Yersinia_unclustered.out - list of all protein singletons that MCL did not group into a cluster (see Materials and Methods); PGL1_Yersinia_unique_locus_tags.txt - names of the 11 locus tag prefixes used for each genome; PGL1_unique_Yersinia.gff - mapping each Yersinia protein to a cluster in tab delimited GFF; PGL1_unique_Yersinia.sigfile - list of the longest protein in each cluster; PGL1_unique_Yersinia.summary - summary table of features of each of the clusters; PGL1_unique_Yersinia.table - summary table of each protein in the clusters. Within each cluster directory are the following files, where 'x' is the cluster name: PGL1_unique_Yersinia-x.faa - multifasta file of the proteins in the cluster; PGL1_unique_Yersinia-x.summary - summary of the properties of the proteins; PGL1_unique_Yersinia-x.matches - blast matches between the proteins of the cluster; PGL1_unique_Yersinia-x.muscle.fasta - muscle alignment of the proteins; PGL1_unique_Yersinia-x.muscle.fasta.gblo - gblocks output of muscle alignment (that is, auto-trimmed alignment); PGL1_unique_Yersinia-x.muscle.fasta.gblo.htm - as above in html format; PGL1_unique_Yersinia-x.muscle.tree - treefile from muscle alignment; PGL1_unique_Yersinia-x.sif - matches between proteins in simple interaction format for display on graphing software. [file gb-2010-11-1-r1-S16.zip › clusters2/PGL1_unique_yersinia-CL1262/PGL1_unique_yersinia-CL1262.muscle.fasta.gblo.htm]

PGL1\_unique\_yersinia-CL1262.muscle.fasta


## Gblocks 0.91b Results

Processed file: **PGL1\_unique\_yersinia-CL1262.muscle.fasta**  
Number of sequences: **11**  
Alignment assumed to be: **Protein**  
New number of positions: **734** (selected positions are underlined in blue)

```
                         10        20        30        40        50        60
                 =========+=========+=========+=========+=========+=========+
yruck0001_30310  -----------------------VGFRPFIWQLANKLRLSGEVCNDGSGVDIRLVQSVNI
yrohd0001_33210  VPEQEFVVDENGLSLRIKGKVQGVGFRPYIWQLAHRFGLYGDVSNDSAGVIVHLWQSPAV
yinte0001_39920  -------VDKNGLCLRVKGKVQGVGFRPYIWQLAHRFALRGDVSNDSAGVTVHLWQTPAV
ykris0001_5810   -------VEINGLCLRIKGKVQGVGFRPYVWQLAHRFGLHGDVSNDSAGVTVHLWQAPAA
ykris0001_5820   ------------------------------------------------------------
yaldo0001_6940   -------VDKNGLSLRIKGKVQGVGFRPYIWQLAHRFGLQGDVSNDSAGVTVHLWQAPAV
yente0001X_6440  -------VEKNGLSLRIKGKVQGVGFRPYIWQLAHRFGLQGDVSNDSAGVTVHLWQAPAV
yfred0001_6970   -------VDENGLRLRVKGKVQGVGFRPYIWQLAHRFGLCGDVSNDSAGVTIHLWQSPAV
ymoll0001_37160  -------VDENGLCLRVKGKVQGVGFRPYIWQLAHRFGLRGDVSNDSAGVTVHLWQSAAV
ymoll0001_37910  ------------------------------------------------------------
yberc0001_8370   -------VETNGLCLRVKGKVQGVGFRPYIWQLAHRYGLRGDVSNDSAGVTIHLWQSAAV
                                        #####################################


                         70        80        90       100       110       120
                 =========+=========+=========+=========+=========+=========+
yruck0001_30310  DLFIEELQRECPPLARIDQILFESFDWLTPPQDFTITTSQQSQMETQVTPDAATCPACLK
yrohd0001_33210  ADFLQALGPNCPPLAHIDSIITAPYHWAQPPQAFAIHHSGAGQMDTQIVPDAASCDACLA
yinte0001_39920  ADFLQALPQDCPPLAQIDSVTTSPYHWAQPPLDFVIHHSGAGQMDTQIVPDAATCDACLA
ykris0001_5810   VDFLQALPQDCPPLARIDSIDTAPYHWQQPPLEFVIHHSGAGQMDTQIVPDAATCDACLN
ykris0001_5820   ------------------------------------------------------------
yaldo0001_6940   ADFLQVLPQDCPPLAHIDSIDTTPYHWVRPPLDFVIQHSGAGQMDTQIVPDAATCDACLS
yente0001X_6440  ADFLQALPQDCPPLAHIDSIDTTPYHWAQPPLDFVIQHSGAGQMDTQIVPDAATCDACLS
yfred0001_6970   AGFLQALPLDCPPLAQIDSIVSAPYHWAQPPLGFVIHHSGAGQMDTQIVPDAATCNACLH
ymoll0001_37160  ADFLRALPQECPPLAQIDSITTAPYHWAPPPLDFVIHHSGAGQMDTQIVPDAATCDACLH
ymoll0001_37910  ------------------------------------------------------------
yberc0001_8370   ADFLRALPQECPPLAQIDSITTTPYHWGQPPLDFVIHHSGAGQMDTQIVPDAATCAACLH
                 ############################################################


                        130       140       150       160       170       180
                 =========+=========+=========+=========+=========+=========+
yruck0001_30310  DITQPDDRRFGYAFTNCTHCGPRFTIIHSMPYDRAATSMAAFTLCPACQQEYQNPADRRF
yrohd0001_33210  EMNDPSNRRYRYPFINCTHCGPRFTIIQRMPYDRPYTSMGKFPLCPACQAEYDHPADRRF
yinte0001_39920  EMNHPANRRYRYPFINCTHCGPRFTIIHRMPYDRPYTAMREFPLCAACQAEYDHPADRRF
ykris0001_5810   EMNDPANRRYRYPFINCTHCGPRFTIIHRMPYDRPDTAMGKFPLCAACQAEYDHPADRRF
ykris0001_5820   ------------------------------------------------------------
yaldo0001_6940   EMNNPTNRRYHYPFINCTHCGPRFTIIQRMPYDRPYTAMSKFPLCAACQAEYDHPADRRF
yente0001X_6440  EMNNPANRRYHYPFINCTHCGPRFTIIQRMPYDRPFTAMSKFPLCAACQREYDHPADRRF
yfred0001_6970   EMNDPANRRYRYPFINCTHCGPRFTIIRRMPYDRPYTAMGKFPLCAACQAEYDHPADRRF
ymoll0001_37160  EMNNPSDRRYRYPFINCTHCGPRFTIIHRMPYDRPDTAMGEFPLCAACQAEYDHPADRRF
ymoll0001_37910  ------------------------------------------------------------
yberc0001_8370   EMNDPADRRYRYPFINCTHCGPRFTIIHRMPYDRPDTAMGEFPLCAACQAEYDHPADRRF
                 ############################################################


                        190       200       210       220       230       240
                 =========+=========+=========+=========+=========+=========+
yruck0001_30310  HAQPVACPQCGPQISASRQNATILTQDADAIQSAVAALRSGKIVAIKGLGGFHLACDATQ
yrohd0001_33210  HAQPNACADCGPQLWLTGANGQAVAHGVSALQQAADALLAGQIVAIKGLGGFHLAVDATN
yinte0001_39920  HAQPNACADCGPQLWLTGREGQPIAHNFAALEQAAAALLAGDIVAVKGLGGFHLAVDATN
ykris0001_5810   HAQPNACADCGPQLWLAAANGQAVAHGFSALEQAAAALLAGEIVAVKGLGGFHLAVDATN
ykris0001_5820   ------------------------------------------------------------
yaldo0001_6940   HAQPNACADCGPQLWLTGADGQTMAQGFSALEQAAVALLAGDIVAVKGLGGFHLAVDATN
yente0001X_6440  HAQPNACADCGPQLWLTGTDGQALAQGFSALEQAAAALLAGDIVAVKGLGGFHLAVDATN
yfred0001_6970   HAQPNACAHCGPQLWLSDANGQTVAQDFAAVQQAAMALLAGEIVAVKGVGGFHLAVDATN
ymoll0001_37160  HAQPNACAVCGPQLWLSGADGQAVAHGFSAVEQAAQALLAGEIVAIKGLGGFHLAVDATH
ymoll0001_37910  ------------------------------------------------------------
yberc0001_8370   HAQPNACAACGPQLWLSEANGQAVAHGFSALEQAAQALLAGEIVAIKGLGGFHLAVDATN
                 ############################################################


                        250       260       270       280       290       300
                 =========+=========+=========+=========+=========+=========+
yruck0001_30310  QTAVVRLRERKQRPTKPLAVMLPSINWLAQCSDDHRSLALRDLLQSPAAPIVLTRHRASS
yrohd0001_33210  AGAVARLRQRKHRPSKPLAVMLPDINWLAACVQSSEMTALLRLLRSPAAPIVLVTKQPQS
yinte0001_39920  AAAVARLRQRKQRPSKPLAVMLPHADWLSRCVQSNDSAALLRVLRSPAAPIVLVSSRADS
ykris0001_5810   AAAVARLRERKHRPTKPLAVMLPDAGWLNSCVQAADNDALFRLLRSSAAPIVLVAKRPEG
ykris0001_5820   ------------------------------------------------------------
yaldo0001_6940   AVAVARLRERKHRPSKPLAVMLPDADWLQSCVSSADHTALLGLLRSPAAPIVLVAKQPES
yente0001X_6440  AAAVARLRERKHRPSKPLAVMLPDADWLQSCVSSADHTALLGLLRSPAAPIVLVAKQPES
yfred0001_6970   STAVARLRARKHRPSKPLAVMLPDASWLKTCVQSTDNDALLRLLRSPAAPIVLVAKQPNS
ymoll0001_37160  AAAVARLRDRKQRPSKPLAVMLPNVDWLMRCVKSGDEGALLRLLRSSAAPIVLVAKQPDS
ymoll0001_37910  ------------------------------------------------------------
yberc0001_8370   AAAVARLRRRKQRPSKPLAVMLPSEEWLTRCVQSADNDALLRLLRSPAAPIVLVAKQPDS
                 ############################################################


                        310       320       330       340       350       360
                 =========+=========+=========+=========+=========+=========+
yruck0001_30310  PLCAAIAPNLDEVGLMLPFTPLHHLLMQAMQTPLVMTSGNSADCAPVITNNDARRQLVDI
yrohd0001_33210  PVCAQIAPDLPEIGVMLPANPLQHLLLQQVARPLVMTSGNGSGKPPALSNEQALSALSDI
yinte0001_39920  PLCAALAPGLPEIGVMLPANPLQHLLLQQVGRPLVMTSGNGRGKPPALSNEQALGALRDI
ykris0001_5810   PLCAAVAPELPEIGIMLPANPLQHLLLQQVARPLVMTSGNGSGKPPALSNQQALSALSEI
ykris0001_5820   ------------------------------------------------------------
yaldo0001_6940   PLCAAVAPELPEIGVMLPANPLQHLLLQQVARPLVMTSGNGRGKPPALSNQQALSALSEI
yente0001X_6440  PLCAAVAPELPEIGVMLPATPLQHLLLQQVARPLVMTSGNGRGKPPALSNQQALSALSEI
yfred0001_6970   PLSAAVAPKLPEIGVMLPANPLQHLLLQQVARPLVMTSGNGSGKPPALSNEQALSALTDI
ymoll0001_37160  PLCAAVAPQLSEIGVMXLIN----------------------------------------
ymoll0001_37910  -------------------------LLQQVARPLVMTSGNGSGKPPALSNEQALSALSDI
yberc0001_8370   PLCAAVAPQLAEIGVMLAANPLQHLLLQQVARPLVMTSGNGSGKPPALSNEQALSALSDI
                 ############################################################


                        370       380       390       400       410       420
                 =========+=========+=========+=========+=========+=========+
yruck0001_30310  ADLWLLHNRDIVQRVDDSLVRWSPQGNEVLRRARGYVPDALRLPAGFACQPPLLALGGDL
yrohd0001_33210  ADHWLLHDRDIVQRADDSLVRFTAHGAEMLRRARGYVPDAFELPPGFSQQPAILALGADM
yinte0001_39920  ADHWLLHDRQIVQRADDSLVRLTGSGAEMLRRARGYVPDAFELPPGFSQQPAVLALGADM
ykris0001_5810   ADHWLLHDREIVQRADDSLVRLTPDGAEMLRRARGYVPDAFELPPGFSQQPAILALGADM
ykris0001_5820   ------------------------------------------------------------
yaldo0001_6940   ADHWLLHDREIVQRADDSLVRLTPHGADMLRRARGYVPDAFELPPGFSQQPAILALGADM
yente0001X_6440  ADHWLLHDREIVQRADDSLVRLTPRGAEMLRRARGYVPDAFELPPGFSQQPAILALGADM
yfred0001_6970   ADHWLLHDRDIVQRADDSLVRFTDSGAEMLRRARGYVPDAFELPPGFSQQPAILALGADM
ymoll0001_37160  ------------------------------------------------------------
ymoll0001_37910  ADHWLLHNRQIVQRADDSLVRFTEQGAEMLRRARGYVPDTFELPPGFSQQPAILALGADM
yberc0001_8370   ADHWLLHNRQIVQRADDSLVRFTAQGAEMLRRARGYVPDSCELPPGFSQQPAILALGADM
                 ############################################################


                        430       440       450       460       470       480
                 =========+=========+=========+=========+=========+=========+
yruck0001_30310  KNTFCLVKGNQAILSAHFGSLNRSDIARQQQQAIEHFQQLYHCTPRVIVHDAHPAYVSHQ
yrohd0001_33210  KNTFCLLRDTSAVLSQHLGDLADSDIAQQQQQLLALFCDIYHFTPQAIAVDAHPGYISHQ
yinte0001_39920  KNTFCLLRDGNAVLSQHLGDLEDRDIAQQQQQLVALFCDIYHFTPQTVVVDAHPGYVSHQ
ykris0001_5810   KNTFCLLRDRNAVLSQHLGDLEDRDIAQQQQQLLALFCDIYHFTPQAIVVDAHPGYVSHQ
ykris0001_5820   ------------------------------------------------------------
yaldo0001_6940   KNTFCLLRDSNAVLSQHLGDLDDRDIAQQQQQLLALFCDIYHFTPQAVVVDAHPAYVSHQ
yente0001X_6440  KNTFCLLRDSNAVLSQHLGDLDDRDIAQQQQQLLALFGDIYHFTPQVVVVDAHPAYVSHQ
yfred0001_6970   KNTFCLLRDSNAVLSQHLGDLADNDIAQQQQQLLALFCDIYHFTPQTVVVDAHPGYVSHQ
ymoll0001_37160  ------------------------------------------------------------
ymoll0001_37910  KNTFCLLRNHAAVLSQHLGDLADSDIAQQQQQLLALFCQIYHFTPQAVVVDAHPAYVSHQ
yberc0001_8370   KNTFCLLRNHTAVLSQHLGDLADSDIAQQQQQLLALFCQIYHFTPQAVVVDAHPAYVSHQ
                 ############################################################


                        490       500       510       520       530       540
                 =========+=========+=========+=========+=========+=========+
yruck0001_30310  RQQ----QPETTDVDVLHHHAHIAACLGEHQWPLDGSHVIGLALDGLGYAAPGALWGGEC
yrohd0001_33210  LGKLWAAQQNIPCVEVLHHHAHLAACLAEHGWPRQGGAVIGLALDGLGYGADGQLWGGEC
yinte0001_39920  LGKTLAAQWNIPCVDVLHHHAHLVACLAEHGWPREGGAVIGVALDGLGYGADGQLWGGEC
ykris0001_5810   FGKEWAAQQNIPCIAVLHHHAHLAACLAEHGWPRQGG-------GGYRFGAG---WAG--
ykris0001_5820   -----------------------------------------LALDGLGYGADGHLWGGEC
yaldo0001_6940   FGREWAAQRNIPCMEVLHHHAHLAACLAEHGWPRQAGEVIGLALDGLGYGANGQLWGGEC
yente0001X_6440  FGREWAAQHNIPCVEVLHHHAHLAACLAEHGWPRQGGAVIGLALDGLGYGVEGKLWGGEC
yfred0001_6970   FGKAWAAQQNIPCVEVLHHHAHLAACLAEHGWPREGGAVIGLALDGLGYGAEGQLWGGEC
ymoll0001_37160  ------------------------------------------------------------
ymoll0001_37910  WGKEWAAQQEIPCVAVLHHHAHLVACLAEHGWPREGGAVIGLALDGLGYGAEGQLWGGEC
yberc0001_8370   LGKQWAAQREIPCVAVLHHHAHLAACLAEHGWPREGGAVIGLALDGLGYGAEGQLWGGEC
                 ############################################################


                        550       560       570       580       590       600
                 =========+=========+=========+=========+=========+=========+
yruck0001_30310  FQVNYLHCEHLGGLPAVALAGGDRASQEPWRNLLAQWQAFVPNWQARPEAKVLLAHPWQP
yrohd0001_33210  LQVDYATCEYVGGLPAVALPGGDLASRQPWRNLLAQLQRFVPNWQQYPEAAAIPQPQGEI
yinte0001_39920  LRVDYTSCEYIGGLPAVALPGGDLASRQPWRNLLAQLQRYVPNWQHLPESAAIPQPEGRV
ykris0001_5810   ----------------------------VWR-----------------------------
ykris0001_5820   LLVDYVNCEYVGGLPAVALPGGDLASRQPWRNLLAQFQRFVPDWQNLPEAAAIPSPQGEV
yaldo0001_6940   LRVDYKTCEYIGGLPAVALPGGDLASRQPWRNLLAQLQRFVPNWQNLPESAAILQSQGRV
yente0001X_6440  LRVDYVNCEYVGGLPAVALPGGDLASRQPWRNLLAQFQRFVPNWQNLPEAAAIPQPQGEV
yfred0001_6970   LRVDYVNCEHIGGLPAVALPGGDLASRQPWRNFLAQLQRFVPRGHNLPEAAAIPQPQGEI
ymoll0001_37160  ------------------------------------------------------------
ymoll0001_37910  LRVDYSDCEYVGGLPAVAMPGGDLASRQPWRNLLAQWQRFVPHWQRLPEAAAIAQPQGAV
yberc0001_8370   LLVDYANCEYLGGLPAVALPGGDLASRQPWRNLLAQLQRFVPHWQQLPEAAAIAQPQGMM
                 ############################################################


                        610       620       630       640       650       660
                 =========+=========+=========+=========+=========+=========+
yruck0001_30310  LSKAIMAGVNSPLASSCGRLFDAVSTVLGCAPDTLSWEGEAACRLEALAHLADGVQHPIS
yrohd0001_33210  LARAIDRGINSPLASSAGRLFDAVAAALNCVPQAISWEGEAACRLEALAWQSTKTAPPVT
yinte0001_39920  LARAIERGINAPLASSTGRLFDAVAAALNLAPLSISWEGEAACQLEALARQSAVSVPPVT
ykris0001_5810   ------------------------------------------------------------
ykris0001_5820   LARAIARDINAPLASSAGRLFDAVAAALNIVSPTISWEGEAACLLEALAWQSSRTVPPVT
yaldo0001_6940   LVRAIERGINSPLASSTGRLFDAVAAALNIAPPSISWEGEAACLLEALAWQSSRTVPPVT
yente0001X_6440  LARAIARGINAPLASSAGRLFDAVAAALNIIPQAISWEGEAACLLEALAWQSSRTVPPVT
yfred0001_6970   LARAIVRGINAPLASSTGRLFDAVAAALNIVPHTISWEGEAACQLEALAWQSTRTVPPVT
ymoll0001_37160  ------------------------------------------------------------
ymoll0001_37910  LARAIERGINAPLASSTGRLFDAVAAALNIVPSSISWEGEAACLLEALAGQSAQSIPPVT
yberc0001_8370   LARAIERGINAPLASSTGRLFDAVAAALHIVPSSISWEGEAACLLEALAGQSAQTAPPVT
                 ############################################################


                        670       680       690       700       710       720
                 =========+=========+=========+=========+=========+=========+
yruck0001_30310  IPLTHCANGTFLDLATFWQQWLDWSAAPAARAFAFHDALAQGFAALAHYHSARTGIHTVA
yrohd0001_33210  LPL-H---HNKLDLATFWQQWLAYKAPPAERAYAFHFAFAQGFATLARQAAQRSGIKTIA
yinte0001_39920  MPL-C---DNQLDLATFWQQWLAYDATPAERAYAFHFALAQGFATLARQAAQYYRIDTIV
ykris0001_5810   ------------------------------------------------------------
ykris0001_5820   MPL-R---DNQLDLGTFWRQWLAFDAAPAERAYAFHFALAQGFATLARQAAQRFAVDTIA
yaldo0001_6940   MPL-R---DNQLDLATFWQQWLAYDATPAERAYAFHFALAQGFANLARQAAQRFGIETIA
yente0001X_6440  MPL-R---DNHLDLATFWRQWLAFDAAPAERAYAFHFALAQGFATLARQAAQRFSIETIA
yfred0001_6970   LPL-H---GNQLDLATFWQQWLAYDAEPAERAYAFHFALAHGFATLARQAAHDFGMDTIA
ymoll0001_37160  ------------------------------------------------------------
ymoll0001_37910  LPLGE---DNQLDLATFWQQWLAYDASPADRAYAFHFALAQGFAALARQAAQHFGIDTIA
yberc0001_8370   LPL-R---DNQLDLATFWQQWLAYDASPAERAYAFHFALAQGFAALARQAAQRFSIDTIA
                 ###     ####################################################


                        730       740       750       760       770       780
                 =========+=========+=========+=========+=========+=========+
yruck0001_30310  LAGGVLHNRLLRQRLHHHLAPLHVLMPHNIPAGDGGLALGQALIACARRQTAAPDAAHHT
yrohd0001_33210  LSGGVLHNRLLRELLRGQLHDFQLLIPQRLPAGDGGLALGQALTFPXXN-----------
yinte0001_39920  FSGGVLHNRLLRELLSAQLHDFQLLLPQRLPAGDGGLALGQALIAAASKCHK--------
ykris0001_5810   ------------------------------------------------------------
ykris0001_5820   FSGGVLHNRLLRQLLREQLHDFKLLMPQRLPAGDGGLALGQAVIAAARG-----------
yaldo0001_6940   FSGGVLHNRLLRELLSEQLHDFQLLMPQRLPAGDGGLALGQALIAAASKCDKC-------
yente0001X_6440  FSGGVLHNRLLRELLSEQLHDFQLLMPQRLPAGDGGLALGQALIAAART-----------
yfred0001_6970   FSGGVLHNQLLRELLCDQLHDFRLLMPQRLPAGDGGLALGQALIAAARGIT---------
ymoll0001_37160  ------------------------------------------------------------
ymoll0001_37910  FSGGVLHNRLLRELLCAQLSDFQLLLPQRLPAGDGGLALGQALIAAARGGDNRRIIKTP-
yberc0001_8370   FSGGVLHNRLLRKLLCAQLSDFQLLLPQRLPAGDGGLALGQALIVAARGVAGR-------
                 ##########################################                  


                 
                 ======
yruck0001_30310  TFSEIK
yrohd0001_33210  ------
yinte0001_39920  ------
ykris0001_5810   ------
ykris0001_5820   ------
yaldo0001_6940   ------
yente0001X_6440  ------
yfred0001_6970   ------
ymoll0001_37160  ------
ymoll0001_37910  ------
yberc0001_8370   ------
```

```
Parameters used
Minimum Number Of Sequences For A Conserved Position: 6
Minimum Number Of Sequences For A Flanking Position: 9
Maximum Number Of Contiguous Nonconserved Positions: 8
Minimum Length Of A Block: 10
Allowed Gap Positions: With Half
Use Similarity Matrices: Yes
```

```
Flank positions of the 2 selected block(s)
Flanks: [24  663]  [669  762]  

New number of positions in PGL1_unique_yersinia-CLUSTERS.dir/PGL1_unique_yersinia-CL1262/PGL1_unique_yersinia-CL1262.muscle.fasta.gblo:  734  (93% of the original 786 positions)
```
