# Supplementary material for: Genomic characterization of the Yersinia genus
Source: Genome Biol. 2010 Jan 4;11(1):R1. doi: 10.1186/gb-2010-11-1-r1 (PMC2847712; doi:10.1186/gb-2010-11-1-r1)
Supplement: Additional file 16 — The top level directory consists of a directory called Additional_cluster_files and 5010 directories, one for each multi-protein cluster family. (This top level directory has been split into three data files for uploading purposes (Additional files 15, 16, 17.) Within the directory are the following files: PGL1_unique_Yersinia_unclustered.out - list of all protein singletons that MCL did not group into a cluster (see Materials and Methods); PGL1_Yersinia_unique_locus_tags.txt - names of the 11 locus tag prefixes used for each genome; PGL1_unique_Yersinia.gff - mapping each Yersinia protein to a cluster in tab delimited GFF; PGL1_unique_Yersinia.sigfile - list of the longest protein in each cluster; PGL1_unique_Yersinia.summary - summary table of features of each of the clusters; PGL1_unique_Yersinia.table - summary table of each protein in the clusters. Within each cluster directory are the following files, where 'x' is the cluster name: PGL1_unique_Yersinia-x.faa - multifasta file of the proteins in the cluster; PGL1_unique_Yersinia-x.summary - summary of the properties of the proteins; PGL1_unique_Yersinia-x.matches - blast matches between the proteins of the cluster; PGL1_unique_Yersinia-x.muscle.fasta - muscle alignment of the proteins; PGL1_unique_Yersinia-x.muscle.fasta.gblo - gblocks output of muscle alignment (that is, auto-trimmed alignment); PGL1_unique_Yersinia-x.muscle.fasta.gblo.htm - as above in html format; PGL1_unique_Yersinia-x.muscle.tree - treefile from muscle alignment; PGL1_unique_Yersinia-x.sif - matches between proteins in simple interaction format for display on graphing software. [file gb-2010-11-1-r1-S16.zip › clusters2/PGL1_unique_yersinia-CL1263/PGL1_unique_yersinia-CL1263.muscle.fasta.gblo.htm]

PGL1\_unique\_yersinia-CL1263.muscle.fasta


## Gblocks 0.91b Results

Processed file: **PGL1\_unique\_yersinia-CL1263.muscle.fasta**  
Number of sequences: **11**  
Alignment assumed to be: **Protein**  
New number of positions: **393** (selected positions are underlined in blue)

```
                         10        20        30        40        50        60
                 =========+=========+=========+=========+=========+=========+
yruck0001_31660  ---MKKSQQSYDVVVVGGGMVGAATALGLAQSGWSVALLEHEAPVPFDSQSAPDLRISAI
ypseu0001X_1284  ---MNKSQPNYDVVVVGGGMVGAAAALGLAQIGWSVALLEHDAPAPFDKDSVPDLRVSAL
ypest0001X_2901  ---MNKSQPNYDVVVVGGGMVGAAAALGLAQIGWSVALLEHDAPAPFDKDSVPDLRVSAL
yaldo0001_22450  ---------------VGGGMVGAAAALGLAQQGWSVAVLEHEAPAPFDTESTPDLRVSAI
yinte0001_25950  MAKMNKSQPNYDVVVVGGGMVGAAAALGLAQTGWSVALLEHEAPAPFEADSVPDLRVSAI
yfred0001_22900  MAKMNKSQPNYDVVVVGGGMVGAAAALGLAQTGWSVALLEHEAPAEFDVNSAPDLRVSAI
yrohd0001_19330  MAKMNKSQPNYDVVVVGGGMVGAAAALGLAQAGWSVALLEHEAPAAFDANSAPDLRVSAI
ymoll0001_24660  ---MNKSQPNYDVVVVGGGMVGAAAALGLAQTGWSVALLEHEAPAPFETNSVPDLRVSAI
yberc0001_21680  ---MNKSQPNYDVVVVGGGMVGAAAALGLAQTGWSVALLEHEAPAPFDANSVPDLRVSAI
yente0001X_1281  MAKMDKSQPNYDVVVVGGGMVGAAAALGLAQTGWSVALLEHEAPQPFEAESVPDLRVSAI
ykris0001_22130  ---MDKSQPNYDVVVVGGGMVGAATALGLAQTGWSVALLEHEAPLPFEAESAPDLRVSAI
                    #########################################################


                         70        80        90       100       110       120
                 =========+=========+=========+=========+=========+=========+
yruck0001_31660  GCTSVGLLKQLGAWSRVQEMRYAPYRRLETWEMPGSTVVFDAASLALPELGFMVENRVLQ
ypseu0001X_1284  GCTSVALLKQLGAWPQVQQMRYAPYRRLETWEQPGSQVVFDAASLSLPELGFMVENRILQ
ypest0001X_2901  GCTSVALLKQLGAWPQVQQMRYAPYRRLETWEQPGSQVVFDAASLSLPELGFMVENRILQ
yaldo0001_22450  GCTSVSLLKQLGAWARVQQMRYAPYRRLETWEQPGSLVVFDAASLSLPELGFMVENRVLQ
yinte0001_25950  GCTSVSLLKQLGVWAQVQQMRYAPYRRLETWEQPGSQVVFDAASLSLPELGFMVENRILQ
yfred0001_22900  GCTSVSLLKQLNVWPRVQQMRYAPYRRLETWEQPGSHVIFDAASLSLPELGFMVENRILQ
yrohd0001_19330  GCTSVSLLKQLKVWPRVQQMRYAPYRRLETWEQPGSEVVFDAASLSLPELGFMVENRVLQ
ymoll0001_24660  GCTSVTLLKQLGAWSKVQQMRYAPYRRLETWEQPGSQVIFDAASLSLPELGFMVENRVLQ
yberc0001_21680  GCTSVALLKQLGVWSRVQQMRYAPYRRLETWEQPGSQVIFDAASLSLPELGFMVENRVLQ
yente0001X_1281  GCTSVSLLKQLGAWSQVQQMRYAPYRRLETWEQPGSQVVFDAASLSLPELGFMVENRVLQ
ykris0001_22130  GCTSVSLLKQLGVWSSVQQMRYAPYRRLETWEQPGSQVVFDAASLSLPELGFMVENRVLQ
                 ############################################################


                        130       140       150       160       170       180
                 =========+=========+=========+=========+=========+=========+
yruck0001_31660  LALWQQIETCANLTLLCPSRLQSMARVDRCWKLTLDTQEQIQARLVVGADGANSQVRRLA
ypseu0001X_1284  LALWQQFAECPNLTLLCPSRLQSMVRIDDYWKVTLNEQEEIQGRLVIGADGANSLVRRLA
ypest0001X_2901  LALWQQFAECPNLTLLCPSRLQSMVRIDDYWKVTLNEQEEIQGRLVIGADGANSLVRRLA
yaldo0001_22450  LALWQQMAECPNLTLLCPSRLQSMIRVDECWNITLEAQQEIQGRLVVGADGANSLVRRLA
yinte0001_25950  LALWQQITECPNLTLLCPSRLQTMVRVDDCWQITLDAQRQIQGRLVIGADGANSLVRQLA
yfred0001_22900  LALWQQMAECPNLTLLCPSRLQSMARVDECWKITLDAQQEIQGRLVIGADGANSLVRRLA
yrohd0001_19330  LALWQQMAECPNLALLCPSRLQTMVRIDECWNITLDAQQVIQGRLVIGADGANSLVRRLA
ymoll0001_24660  LALWQQMAECQNLTLLCPSRLQSMVRVDDYWKITLDAQQEIQGHLVVGADGANSLVRRLA
yberc0001_21680  LALWQQMAECHNLTLLCPSRLQTMVRVDDYWKITLDAQQEIQGHLVIGADGANSLVRRLV
yente0001X_1281  LALWQQMATCPNLTLLCPSRLQTMVRVDDYWKITLDAQREIQSHLVVGADGANSLVRRLA
ykris0001_22130  LALWQQMAECPNLTLLCPSRLQTMIRVDDYWKITLDAQQEIQSHLVIGADGANSLVRRLA
                 ############################################################


                        190       200       210       220       230       240
                 =========+=========+=========+=========+=========+=========+
yruck0001_31660  GIGTSGWQYRQSCMLISINTGAPQQDVTWQQFFPSGPRAFLPLFDHWASLVWYDSPQRIR
ypseu0001X_1284  GIGTSGWQYRQSCMLITVDTDVMQQDTTWQQFFPTGPRAFLPLFDHWASLVWYDSPQRIR
ypest0001X_2901  GIGTSGWQYRQSCMLITVETDVMQQDTTWQQFFPTGPRAFLPLFDHWASLVWYDSPQRIR
yaldo0001_22450  GIGTSGWQYRQSCMLITVETDSAQQDTTWQQFFPTGPRAFLPLFNNWASLVWYDNPQRIR
yinte0001_25950  GIGTSGWQYRQSCMLITVETDTAQQDTTWQQFFPTGPRAFLPLFGQWASLVWYDSPQRIR
yfred0001_22900  GIGTSGWQYRQSCMLITVETDTAQQDTTWQQFFPTGPRAFLPLFDNWASLVWYDSPQRIR
yrohd0001_19330  GIGTSGWQYRQSCLLITVATDTAQQDTTWQQFFPTGPRAFLPLFDNWASLVWYDSPQRIR
ymoll0001_24660  SIGTSGWQYRQSCMLITVETDTAQQDTTWQQFFPSGPRAFLPLFDHWASLVWYDSPQRIR
yberc0001_21680  GIGTSGWQYRHSCMLITVETDTPQQETTWQQFSPSGPRAFLPLFDRWGSLVWYDSPQRIR
yente0001X_1281  GIGTSGWQYRQSCMLITVETDTAQQDTTWQQFFPSGPRAFLPLFDNWASLVWYDSPQRIR
ykris0001_22130  GICTSGWQYRQSCMLMTVETDTAQQDTTWQQFFPSGPRAFLPLFDNWASLVWYDSPQRIR
                 ############################################################


                        250       260       270       280       290       300
                 =========+=========+=========+=========+=========+=========+
yruck0001_31660  QLQNMPLEQLNQEITRAFPDRLGRVNAVAAGSFPLTRRHAQRYVQAGLVLLGDAAHTINP
ypseu0001X_1284  QLQAMSMAQLSQEIAAFFPSRLGAVKAIAAGAFPLVRRHAQQYVKPGLVLLGDAAHTINP
ypest0001X_2901  QLQAMSMAQLSQEIAAFFPSRLGAVKAIAAGAFPLVRRHAQQYVKPGLVLLGDAAHTINP
yaldo0001_22450  QLQAMPMAQLNQAIAAAFPSRLGAVNAIAAGSFPLVRRHAQHYVQPGLVLLGDAAHTINP
yinte0001_25950  QLQALPMAQLNQEIAKAFPSRLGAVKAIAAGSFPLVRRHAQRYVQPGLVLLGDAAHTINP
yfred0001_22900  QLQALPMAQLNQEIAAAFPSRLGAVKAIAAGSFPLVRRHAQQYVQPGLVLLGDAAHTINP
yrohd0001_19330  QLQAMPMAQLNQEIAAAFPSRLGAVKAIAAGSFPLVRRHAQQYVQPGLVLLGDAAHTINP
ymoll0001_24660  QLQAMPMTQLNQEIAAAFPSRLGEVKAIAAGSFPLVRRHAQQYVQPGLVLLGDAAHTINP
yberc0001_21680  QLQAMPMVQLNQEIAAAFPSRLGAVKAIAAGSFPLVRRHAQHYVQPGLALLGDAAHTINP
yente0001X_1281  LLQALPLAQLNQEIAAAFPSRLGTVNAIATGSFPLVRRHAQQYVQPGLVLLGDAAHTINP
ykris0001_22130  QLQMMPMTQLNQEITAAFPSRLGAVKAIAAGSFPLVRRHAQQYVQPGLVLLGDAAHTINP
                 ############################################################


                        310       320       330       340       350       360
                 =========+=========+=========+=========+=========+=========+
yruck0001_31660  LAGQGVNLGYRDVDALLDVLSQARDLAEDWSSEAVLLRYQRKRRSDNLMMQSGMDLFYKA
ypseu0001X_1284  LAGQGVNLGYRDVDALLEVLSQARELAEPWHSEQVLLRYQRRRRTDNLMMQSGMDLFYTA
ypest0001X_2901  LAGQGVNLGYRDVDALLEVLSQARELAEPWHSEQVLLRYQRRRRTDNLMMQSGMDLFYTA
yaldo0001_22450  LAGQGVNLGYRDVDALLDVLHQARELAQPWYSEQVLLRYQRRRRTDNMMMQSGMDLFYTA
yinte0001_25950  LAGQGVNLGYRDVDALLEVLNQARERAEPWHSERVLLRYQRRRRTDNLIMQSGMDVFYTT
yfred0001_22900  LAGQGVNLGYRDVDALLEVLNQARERAELWHSEPVLLRYQRRRRTDNLIMQSGMDVFYTT
yrohd0001_19330  LAGQGVNLGYRDVDALLEVLNQAREQAEIWYSEPVLLRYQRRRRTDNLIMQSGMDVFYTT
ymoll0001_24660  LAGQGVNLGYRDVDALLNVLNQARELAEPWYSEQVLLRYQRRRRTDNLVMQSGMDLFYTA
yberc0001_21680  LAGQGVNLGYRDVDALLNVLNQAREVAEPWHSEQVLLRYQRRRRTDNLIMQSGMDLFYTA
yente0001X_1281  LAGQGVNLGYRDVDVLLEVLNQAREQAESWHSEQVLQRYQRRRRTDNLIMQSGMDVFYTA
ykris0001_22130  LAGQGVNLGYRDVDALLDVLNQAREQAESWHSEPVLLRYQRRRRTDNLIMQSGMDVFYTA
                 ############################################################


                        370       380       390
                 =========+=========+=========+======
yruck0001_31660  FSNDLPPVKMIRNLALIAAQRAGKLKEHALKYALGL
ypseu0001X_1284  FSNDLPAVKFARNLALMVAQRAGKLKEHALRYALGL
ypest0001X_2901  FSNDLPAVKFARNLALMVAQRAGKLKEHALRYALGL
yaldo0001_22450  FSNDLPPVKFVRNLALMAAQRAGKLKERALRYALGL
yinte0001_25950  FSNDLPPVKFVRNLALIAAQRAGKLKEHALRYALGL
yfred0001_22900  FSNNLPAVKFVRNLALITAQRAGKLKEYALRYALGL
yrohd0001_19330  FSNNLPAVKFVRNLALMAAQRAGKLKEHALKYALGL
ymoll0001_24660  FSNDLPPVRFVRNLALMAAQRAGKLKEQALKYALGL
yberc0001_21680  FSNDLPPVKFVRNLALMAAQRAGKLKEHALKYALGL
yente0001X_1281  FSNDLPAVKFARNLALMAAQRAGKLKEHALKYALGL
ykris0001_22130  FSNDLPVVKFARNLALMAAQRAGKLKEHALKYALGL
                 ####################################
```

```
Parameters used
Minimum Number Of Sequences For A Conserved Position: 6
Minimum Number Of Sequences For A Flanking Position: 9
Maximum Number Of Contiguous Nonconserved Positions: 8
Minimum Length Of A Block: 10
Allowed Gap Positions: With Half
Use Similarity Matrices: Yes
```

```
Flank positions of the 1 selected block(s)
Flanks: [4  396]  

New number of positions in PGL1_unique_yersinia-CLUSTERS.dir/PGL1_unique_yersinia-CL1263/PGL1_unique_yersinia-CL1263.muscle.fasta.gblo:  393  (99% of the original 396 positions)
```
