# Supplementary material for: Genomic characterization of the Yersinia genus
Source: Genome Biol. 2010 Jan 4;11(1):R1. doi: 10.1186/gb-2010-11-1-r1 (PMC2847712; doi:10.1186/gb-2010-11-1-r1)
Supplement: Additional file 16 — The top level directory consists of a directory called Additional_cluster_files and 5010 directories, one for each multi-protein cluster family. (This top level directory has been split into three data files for uploading purposes (Additional files 15, 16, 17.) Within the directory are the following files: PGL1_unique_Yersinia_unclustered.out - list of all protein singletons that MCL did not group into a cluster (see Materials and Methods); PGL1_Yersinia_unique_locus_tags.txt - names of the 11 locus tag prefixes used for each genome; PGL1_unique_Yersinia.gff - mapping each Yersinia protein to a cluster in tab delimited GFF; PGL1_unique_Yersinia.sigfile - list of the longest protein in each cluster; PGL1_unique_Yersinia.summary - summary table of features of each of the clusters; PGL1_unique_Yersinia.table - summary table of each protein in the clusters. Within each cluster directory are the following files, where 'x' is the cluster name: PGL1_unique_Yersinia-x.faa - multifasta file of the proteins in the cluster; PGL1_unique_Yersinia-x.summary - summary of the properties of the proteins; PGL1_unique_Yersinia-x.matches - blast matches between the proteins of the cluster; PGL1_unique_Yersinia-x.muscle.fasta - muscle alignment of the proteins; PGL1_unique_Yersinia-x.muscle.fasta.gblo - gblocks output of muscle alignment (that is, auto-trimmed alignment); PGL1_unique_Yersinia-x.muscle.fasta.gblo.htm - as above in html format; PGL1_unique_Yersinia-x.muscle.tree - treefile from muscle alignment; PGL1_unique_Yersinia-x.sif - matches between proteins in simple interaction format for display on graphing software. [file gb-2010-11-1-r1-S16.zip › clusters2/PGL1_unique_yersinia-CL1264/PGL1_unique_yersinia-CL1264.muscle.fasta.gblo.htm]

PGL1\_unique\_yersinia-CL1264.muscle.fasta


## Gblocks 0.91b Results

Processed file: **PGL1\_unique\_yersinia-CL1264.muscle.fasta**  
Number of sequences: **11**  
Alignment assumed to be: **Protein**  
New number of positions: **186** (selected positions are underlined in blue)

```
                         10        20        30        40        50        60
                 =========+=========+=========+=========+=========+=========+
yruck0001_6540   -MSLNDHLSAIHFDWPTRRCHRGFGLPEMMLAMMFGSVMVLSAVKVYPQLRQRLAVVYQH
yaldo0001_7140   ---------------------MGFSLPEVMLALSIGSLIVLSATQVFPKLLKQVSMLQHH
ypseu0001X_3389  MPLVVNVLSTPLNAQPTR--TAGFTLPEMMLALSFGSLIALSTAQVLPKLSQQISVLQQH
ypest0001X_1224  MPLVVNVLSTPLNAQPTR--TAGFTLPEMMLALSFGSLIALSTAQVLPKLSQQISVLQQH
ymoll0001_7770   MPRVIKKPIGPLSEHFWHKDISGFTLPEMMLALSIGSIIMLGSAQVFPKLRQQISTLQQH
yberc0001_6920   MP-----------EQTWHKSISGFTLPEMMLALSIGSMIVFGSAQIFPKLRQQISILQQH
yrohd0001_8500   --------VTATSGHLGHKYRAGFTLPEMMLALSIGSLIIMAATQTFPKLRRQISVLQQH
yente0001X_9390  MPEITKKLDVCVPSHFSHKYRAGFTLPEMMLALGVGSMIIMAATQVFPQLHKQISILQQH
yinte0001_8480   -----------VSARWGLKCITGFTLPEMMLALSIGSLIMLGATEVFPKLRKQISILQQH
ykris0001_8100   MLVTIKKPEAAVSGYFLHKCMTGFTLPEMMLALSVGSLIIMAATQTFPRLHKQIARLQQH
yfred0001_43670  --------------------MAGFTLPEMMLALSIGSLIIMAASQTFPKLRQQISYLQQH
                                       ######################################


                         70        80        90       100       110       120
                 =========+=========+=========+=========+=========+=========+
yruck0001_6540   HSLELSMQRVISSIEKDLRRAGFCNGQCQGTRFYLSQYKGEKADSCVIVAYDLNRNGHWE
yaldo0001_7140   YRLELVMRQVIAVLEKDIHRAGFCRGECLGNAVTTGNYLGEMKDSCVIVAYDLNRNGRWE
ypseu0001X_3389  YRLELVMNQAMGAMEKDLRRAGFCHGKCQGEAITIEHYLGETAHSCLIVAYDLNCNGRWE
ypest0001X_1224  YRLELVMNQAMGAMEKDLRRAGFCHGKCQGEAITIEHYLGETAHSCLIVAYDLNCNGRWE
ymoll0001_7770   YHLELVLSQAMATLEKDLRRAGFCHGECKGKAVTTQQYPGETTDSCLIVAYDLNRNGRWE
yberc0001_6920   YRLELALSQAMAMLEKDLRRAGFCHGECQGKAITTHHYPGEATDSCLIVAYDLNRNGRWE
yrohd0001_8500   YHLELVLNQAVTALEKDLRRAGFCHGECQGEAMTTHHYPGETANSCLIVAYDFNRNGRWE
yente0001X_9390  YHLELALSQVMAVLEKDLRRAGFCHGECQGEAIITASYPSEASNSCLIVAYDLNRNGRWE
yinte0001_8480   YYLELALSQVMAVLEKDLRRAGFCHGECQGTAVTTHHYPAEATNSCLIVAYDLNRNGRWE
ykris0001_8100   YHLELALSQTMAALEKDLRRAGFCHGECQGNAMTTQHYPGEVTNSCLIVAYDLNRNGRWE
yfred0001_43670  YHLELALSQVMAALEKDLRRAGFCHGECQGKAVTTHNYPGETTNSCLIVAYDLNHNGRWE
                 ############################################################


                        130       140       150       160       170       180
                 =========+=========+=========+=========+=========+=========+
yruck0001_6540   GIKHQKSEYFGYRLRKNQLEAARGERDCQSRGWEGLFETQKISITDFGIQREKPTLN--S
yaldo0001_7140   GAKHQESEYFGYRLRNRALEGQRGELNCHGSGWERLFDPQEVTVTHFSVNYLSRQPSDTG
ypseu0001X_3389  GAKHQESEYFGYRLRNKALESQRGELNCHGRGWEKLFDPRDVTVTHFSVTPLS------G
ypest0001X_1224  GAKHQESEYFGYRLRNKALESQRGELNCHGRGWEKLFDPRDVTVTHFSVTPLS------G
ymoll0001_7770   GEKHQESEYFGYRLRNKALETQRGELNCAGGGWERVFDPKEVTVTHFSVRLLSEPAS--A
yberc0001_6920   GEKHQESEYFGYRLRNKALEAQRGELNCVGGSWERVFDPKEVTITHFSISRLPEPTS--A
yrohd0001_8500   GEKHQESEYFGYRLRNKALESQRGELNCSGRGWEKLLDPQEVTVMHFSVELLPSQTL--G
yente0001X_9390  GAKHQESEYFGYRLRKKTLESQRGKLNCSGGGWEKLLDPKDITITHFSVSRLPQQTS--D
yinte0001_8480   GEKHQESEYFGYRLRNKALEGQRGELNCSGRGWEKLLDPREITITHFSISQLPQQTS--N
ykris0001_8100   GAKHQESEYFGYRLRNKALEGQRGELNCSGSGWEKLFDPQSITITHFSVTPLPQQTS--G
yfred0001_43670  GEKHPQSEYFGYRLHNKALEGQRGELNCSARGWEKLFDPQEITITHFSVNQLPRQVS--G
                 #########################################################  #


                        190       200       210
                 =========+=========+=========+
yruck0001_6540   TLLTLRLFGHIKQAPEIEQRLQHTVNGYNL
yaldo0001_7140   TFFSVRLAGQKTGNPAIRYQITHLVRGNNL
ypseu0001X_3389  QLFKLRLVGHKTGNPAIHHQVTYLIRGNNV
ypest0001X_1224  QLFKLRLVGHKTGNPAIHHQVTYLIRGNNV
ymoll0001_7770   QIYRVQLAGQSTSNAAVHHQLIYKIRGNNL
yberc0001_6920   QIYKVQLAGQSTGNATVHHQLIYMIRGNNL
yrohd0001_8500   KVYLVQLVGQKTGNPAIYHQITYRIRGSNL
yente0001X_9390  QVYRVQLAGQKRGNPTIHRQLTYTIRGDNL
yinte0001_8480   QVYRVQLAGQKTGNPVIHHQLTYTIRRNNL
ykris0001_8100   QVYSVQLAGQSTGNPAIHSQLSYTIRGNNL
yfred0001_43670  QIYRVQLAGQRKSHPAIHHQLTYTIRGNNL
                 ##############################
```

```
Parameters used
Minimum Number Of Sequences For A Conserved Position: 6
Minimum Number Of Sequences For A Flanking Position: 9
Maximum Number Of Contiguous Nonconserved Positions: 8
Minimum Length Of A Block: 10
Allowed Gap Positions: With Half
Use Similarity Matrices: Yes
```

```
Flank positions of the 2 selected block(s)
Flanks: [23  177]  [180  210]  

New number of positions in PGL1_unique_yersinia-CLUSTERS.dir/PGL1_unique_yersinia-CL1264/PGL1_unique_yersinia-CL1264.muscle.fasta.gblo:  186  (88% of the original 210 positions)
```
