# Supplementary material for: Genomic characterization of the Yersinia genus
Source: Genome Biol. 2010 Jan 4;11(1):R1. doi: 10.1186/gb-2010-11-1-r1 (PMC2847712; doi:10.1186/gb-2010-11-1-r1)
Supplement: Additional file 16 — The top level directory consists of a directory called Additional_cluster_files and 5010 directories, one for each multi-protein cluster family. (This top level directory has been split into three data files for uploading purposes (Additional files 15, 16, 17.) Within the directory are the following files: PGL1_unique_Yersinia_unclustered.out - list of all protein singletons that MCL did not group into a cluster (see Materials and Methods); PGL1_Yersinia_unique_locus_tags.txt - names of the 11 locus tag prefixes used for each genome; PGL1_unique_Yersinia.gff - mapping each Yersinia protein to a cluster in tab delimited GFF; PGL1_unique_Yersinia.sigfile - list of the longest protein in each cluster; PGL1_unique_Yersinia.summary - summary table of features of each of the clusters; PGL1_unique_Yersinia.table - summary table of each protein in the clusters. Within each cluster directory are the following files, where 'x' is the cluster name: PGL1_unique_Yersinia-x.faa - multifasta file of the proteins in the cluster; PGL1_unique_Yersinia-x.summary - summary of the properties of the proteins; PGL1_unique_Yersinia-x.matches - blast matches between the proteins of the cluster; PGL1_unique_Yersinia-x.muscle.fasta - muscle alignment of the proteins; PGL1_unique_Yersinia-x.muscle.fasta.gblo - gblocks output of muscle alignment (that is, auto-trimmed alignment); PGL1_unique_Yersinia-x.muscle.fasta.gblo.htm - as above in html format; PGL1_unique_Yersinia-x.muscle.tree - treefile from muscle alignment; PGL1_unique_Yersinia-x.sif - matches between proteins in simple interaction format for display on graphing software. [file gb-2010-11-1-r1-S16.zip › clusters2/PGL1_unique_yersinia-CL1265/PGL1_unique_yersinia-CL1265.muscle.fasta.gblo.htm]

PGL1\_unique\_yersinia-CL1265.muscle.fasta


## Gblocks 0.91b Results

Processed file: **PGL1\_unique\_yersinia-CL1265.muscle.fasta**  
Number of sequences: **11**  
Alignment assumed to be: **Protein**  
New number of positions: **151** (selected positions are underlined in blue)

```
                         10        20        30        40        50        60
                 =========+=========+=========+=========+=========+=========+
yruck0001_6530   ------------------------MKNRIQTAQQGFSLVEWLMVIALIGVIGSIGTQSWN
ypseu0001X_3390  MKIYSIKTGKLLPKAPLLRKQKVFIKQRSLIKQRGISLIELLVVITLVSVMTLWGGQSWH
ypest0001X_9860  --------------------------------------------------MTLWGGQSWH
yaldo0001_7150   ------------MKTNIRLNRTPLTQGYSIVKQQGITLIELLLVIALVGSMAVWASQSWH
yberc0001_6930   ------------MKIDIALNKIGFIKNNFLIKQKGISLIEVLLVIALVGVMATWGAQSWH
ymoll0001_7760   ------------MKIDIAPNKIGRINNNRLIKQKGISLIEVLLVIALVGVMATWGAQSWH
yrohd0001_8490   --------------------------------------------------MAAWGAQSWH
yente0001X_9380  ------------MKTNLRLNKLRLISGNIIKKQKGISLIELLLVIALAGIMTVWGAQSWH
yfred0001_43660  -----------------------------------MTLIELLLVIALVGSMVTWGAQRWH
yinte0001_8470   ------------MDTNIRLKRTAFIREYSLKKQKGISLIELLLVIVLVGITSLWGAQNWH
ykris0001_8090   -----------------------MHKNNILKKQNGISLIELLLVIALAGVMAVWGAQNWH
                                                      #######################


                         70        80        90       100       110       120
                 =========+=========+=========+=========+=========+=========+
yruck0001_6530   HYQQREKLLITARQLLIFLTHVQTDAFWLNRSALLWQSHSEHWCIGSGIQPTNGCSQQDG
ypseu0001X_3390  HYRQRAKLADSARQLLAFLTHLQAEANRNNRTAILWAQPDGQGCLGSGHKPTPPCTGLAG
ypest0001X_9860  HYRQRAKLADSARQLLAFLTHLQAEANRNNRTAILWAQPDGQGCLGSGHKPTPPCTGLAG
yaldo0001_7150   HYRQRERLADSARQLLGFLTHLQAQAHRSNRTALLWVQQSGQGCLGSGDKPILPCSLTTN
yberc0001_6930   QYRQRERLADSARQLLAFLTHLQVQANRSNDTALLWIQQHGQGCLGSGDKPTAPCSVLTG
ymoll0001_7760   HYRQREKLADSARQLLAYLTHLQVQVNRNNDTALLWIQKNRLGCLGSGNNPATPCSALAG
yrohd0001_8490   HYRQRERLADSARQLLAYLTYLQAGANRGNYTALLWVQPNGQGCLGSGDKPVIPCSSLAS
yente0001X_9380  HYRQRERLADSARQLLAFLTHLQAKANRSNSTTLLWIRQDGQGCLGSGNKPTIACSLLEE
yfred0001_43660  HYQQRERLADSARQLLAFLTHLQAEANRSNYTALLWVQPDGQGCLSSGDKPVMSCSSLAG
yinte0001_8470   HYRQRERLADSARQLLAFLTHLQAQANRRNNTELLWIQQNGQGCLGSGDKPATPCSALTD
ykris0001_8090   HYRQRERLADSARQLLAFLTHLQAKTNRSNNTALLWIQQEEQGCLGSGDKPATPCSSLDG
                 ############################################################


                        130       140       150       160       170       180
                 =========+=========+=========+=========+=========+=========+
yruck0001_6530   WLFSPEYPDITLAEYMPENMGFYGLRNTAQPGHITLANSAGRIRLVISNQGRMRLCSEQH
ypseu0001X_3390  SLFIPPYRDIAITLPLQKEMGFYGVRNTAQAGNIILSSPAGRIRLVISSRGRIRLCSEQQ
ypest0001X_9860  SLFIPPYRDIAIM-----------------------------------------------
yaldo0001_7150   TVFTPPYPDVVISSLMQKEMGFYGVRNTAQAGSIILSNPVGRIRLVISARGRMRLCSEGK
yberc0001_6930   RVFIPPYPDVAITMSLQKSIGFYGVRNTAQAGSILLNNPAGRIRLIISSRGRIRLCGEGQ
ymoll0001_7760   NVFILPYPDVTIAISLQKDVGFYGVRNTAQAGSILLNSPAGRIRLIISSRGRMRLCSEGQ
yrohd0001_8490   RVFIPPYPDVVITLSLNKDIGFFGVRNTAQAGNIQLSNPAGRIRLIISSRGRMRLCSEGQ
yente0001X_9380  GIFTPPYPDVSITISLNKEIGFYGIRNTAQAGNIMLSNPAGHIRLIISSRGRIRLCSEGQ
yfred0001_43660  PIFTPPYPDVSISISLKKEIGFFGVRNTAQAGNIILSSPAGRIRLVISSRGRMRLCSEEQ
yinte0001_8470   SVFIPPYPDVAIATSLQKEMGFFGVRNTAQAGNIMLSNPAGSIRLVISSRGRLRLCSEGQ
ykris0001_8090   SVFIPPYPDVSIAISLQKEMGFFGVRNTAQAGNIMLSSPAGRIRLIISSRGRMRLCSDEQ
                 ############################################################


                        190
                 =========+
yruck0001_6530   SQGRIPLCH-
ypseu0001X_3390  SMAGIHLCL-
ypest0001X_9860  ----------
yaldo0001_7150   ALGGIRLCQK
yberc0001_6930   SISGIHVC--
ymoll0001_7760   SISGIYVCQE
yrohd0001_8490   SIAGIHLCKS
yente0001X_9380  SIAGIHLCQK
yfred0001_43660  SVAGIHLCQ-
yinte0001_8470   PISGIHICQ-
ykris0001_8090   SIAGIHLC--
                 ########
```

```
Parameters used
Minimum Number Of Sequences For A Conserved Position: 6
Minimum Number Of Sequences For A Flanking Position: 9
Maximum Number Of Contiguous Nonconserved Positions: 8
Minimum Length Of A Block: 10
Allowed Gap Positions: With Half
Use Similarity Matrices: Yes
```

```
Flank positions of the 1 selected block(s)
Flanks: [38  188]  

New number of positions in PGL1_unique_yersinia-CLUSTERS.dir/PGL1_unique_yersinia-CL1265/PGL1_unique_yersinia-CL1265.muscle.fasta.gblo:  151  (79% of the original 190 positions)
```
