# Supplementary material for: Genomic characterization of the Yersinia genus
Source: Genome Biol. 2010 Jan 4;11(1):R1. doi: 10.1186/gb-2010-11-1-r1 (PMC2847712; doi:10.1186/gb-2010-11-1-r1)
Supplement: Additional file 16 — The top level directory consists of a directory called Additional_cluster_files and 5010 directories, one for each multi-protein cluster family. (This top level directory has been split into three data files for uploading purposes (Additional files 15, 16, 17.) Within the directory are the following files: PGL1_unique_Yersinia_unclustered.out - list of all protein singletons that MCL did not group into a cluster (see Materials and Methods); PGL1_Yersinia_unique_locus_tags.txt - names of the 11 locus tag prefixes used for each genome; PGL1_unique_Yersinia.gff - mapping each Yersinia protein to a cluster in tab delimited GFF; PGL1_unique_Yersinia.sigfile - list of the longest protein in each cluster; PGL1_unique_Yersinia.summary - summary table of features of each of the clusters; PGL1_unique_Yersinia.table - summary table of each protein in the clusters. Within each cluster directory are the following files, where 'x' is the cluster name: PGL1_unique_Yersinia-x.faa - multifasta file of the proteins in the cluster; PGL1_unique_Yersinia-x.summary - summary of the properties of the proteins; PGL1_unique_Yersinia-x.matches - blast matches between the proteins of the cluster; PGL1_unique_Yersinia-x.muscle.fasta - muscle alignment of the proteins; PGL1_unique_Yersinia-x.muscle.fasta.gblo - gblocks output of muscle alignment (that is, auto-trimmed alignment); PGL1_unique_Yersinia-x.muscle.fasta.gblo.htm - as above in html format; PGL1_unique_Yersinia-x.muscle.tree - treefile from muscle alignment; PGL1_unique_Yersinia-x.sif - matches between proteins in simple interaction format for display on graphing software. [file gb-2010-11-1-r1-S16.zip › clusters2/PGL1_unique_yersinia-CL1266/PGL1_unique_yersinia-CL1266.muscle.fasta.gblo.htm]

PGL1\_unique\_yersinia-CL1266.muscle.fasta


## Gblocks 0.91b Results

Processed file: **PGL1\_unique\_yersinia-CL1266.muscle.fasta**  
Number of sequences: **11**  
Alignment assumed to be: **Protein**  
New number of positions: **264** (selected positions are underlined in blue)

```
                         10        20        30        40        50        60
                 =========+=========+=========+=========+=========+=========+
yruck0001_6520   MKQYLDLMKKVLDEGTPKADRTGTGTLSIFGHQMRFNLQDGFPLVTTKRCHLRSIIHELL
yinte0001_8460   MKQYLDLMKKVLEEGTPKADRTGTGTVSIFGHQMRFNLQDGFPLVTTKRCHLRSIIHELL
yente0001X_9370  MKQYLDLMKKVLEEGTTKDDRTGTGTLSIFGHQMRFNLQDGFPLVTTKRCHLRSIIHELL
ypseu0001X_3391  MKQYLDLMKKVLEEGTPKADRTGTGTLSIFGHQMRFNLQDGFPLVTTKRCHLRSIIHELL
ypest0001X_9870  MKQYLDLMKKVLEEGTPKADRTGTGTLSIFGHQMRFNLQDGFPLVTTKRCHLRSIIHELL
ykris0001_8080   MKQYLDLMKKVLEEGTPKADRTGTGTVSIFGHQMRFNLQDGFPLVTTKRCHLRSIIHELL
yfred0001_43650  MKQYLDLMKKVLAEGTAKDDRTGTGTVSIFGHQMRFNLQDGFPLVTTKRCHLRSIIHELL
yaldo0001_7160   MKQYLDLMKKVLEEGTPKADRTGTGTLSIFGHQMRFNLQDGFPLVTTKRCHLRSIIHELL
yrohd0001_8480   MKQYLDLMKKVLEEGTPKDDRTGTGTVSIFGHQMRFNLQDGFPLVTTKKCHLRSIIHELL
yberc0001_6940   MKQYLDLMKKVLEEGTPKDDRTGTGTVSIFGHQMRFNLQDGFPLVTTKRCHLRSIIHELL
ymoll0001_7750   MKQYLDLMKKVLEEGTPKDDRTGTGTVSIFGHQMRFNLQDGFPLVTTKRCHLRSIIHELL
                 ############################################################


                         70        80        90       100       110       120
                 =========+=========+=========+=========+=========+=========+
yruck0001_6520   WFLNGDTNIGYLKENNVSIWDEWADENGDLGPVYGKQWRAWGAADGRQIDQLSKVVEQLK
yinte0001_8460   WFLNGDTNIGYLKENNVSIWDEWADENGDLGPVYGKQWRAWGAADGRKIDQLSNVVKQLK
yente0001X_9370  WFLNGDTNIGYLKENNVSIWDEWADENGDLGPVYGKQWRAWGAADGRQIDQLSKVVQQLK
ypseu0001X_3391  WFLNGDTNIAYLKENNVSIWDEWADENGDLGPIYGKQWRAWGAADGRKIDQLSNVVNQLK
ypest0001X_9870  WFLNGDTNIAYLKENNVSIWDEWADENGDLGPIYGKQWRAWGAADGRKIDQLSNVVNQLK
ykris0001_8080   WFLNGDTNIAYLKENNVSIWDEWADENGDLGPVYGKQWRAWGAADGRQIDQLSKVVQQLK
yfred0001_43650  WFLNGDTNIAYLKENNVSIWDEWADENGDLGPVYGKQWRAWGAADGRKIDQLSNVVQQLK
yaldo0001_7160   WFLNGDTNIAYLKENNVSIWDEWADENGSLGPVYGKQWRAWGAADGRQIDQLSNVVKQLK
yrohd0001_8480   WFLNGDTNIAYLKENNVSIWDEWADENGDLGPVYGKQWRAWGAADGRKIDQLSNVVQQLK
yberc0001_6940   WFLNGDTNIGYLKENSVSIWDEWADENGDLGPVYGKQWRAWGAADGRQIDQLSNVVKQLK
ymoll0001_7750   WFLNGDTNIGYLKENSVSIWDEWADENGDLGPVYGKQWRAWGAADGRQIDQLSNVVKQLK
                 ############################################################


                        130       140       150       160       170       180
                 =========+=========+=========+=========+=========+=========+
yruck0001_6520   QDPDSRRIIVSAWNVGELDQMALAPCHAFFQFYVADGKLSCQLYQRSCDIFLGLPFNIAS
yinte0001_8460   QDPDSRRIIVSAWNVGELEQMALAPCHAFFQFYVADGKLSCQLYQRSCDVFLGLPFNIAS
yente0001X_9370  QDPNSRRIIVSAWNVGELDQMALAPCHAFFQFYVADGKLSCQLYQRSCDVFLGLPFNIAS
ypseu0001X_3391  QDPDSRRIIVSAWNVGELDQMALAPCHAFFQFYVADGKLSCQLYQRSCDVFLGLPFNIAS
ypest0001X_9870  QDPDSRRIIVSAWNVGELDQMALAPCHAFFQFYVADGKLSCQLYQRSCDVFLGLPFNIAS
ykris0001_8080   QDPDSRRIIVSAWNVGELDQMALAPCHAFFQFYVADGKLSCQLYQRSCDVFLGLPFNIAS
yfred0001_43650  QDPNSRRIIVSAWNVGELDQMALAPCHAFFQFYVAEGKLSCQLYQRSCDVFLGLPFNIAS
yaldo0001_7160   QDPDSRRIIVSAWNVGELDQMALAPCHAFFQFYVADGKLSCQLYQRSCDVFLGLPFNIAS
yrohd0001_8480   QDPNSRRIIVSAWNVGELDQMALAPCHAFFQFYVADGKLSCQLYQRSCDVFLGLPFNIAS
yberc0001_6940   QDPNSRRIIVSAWNVGELDQMALAPCHAFFQFYVADGKLSCQLYQRSCDVFLGLPFNIAS
ymoll0001_7750   QDPNSRRIIVSAWNVGELDQMALAPCHAFFQFYVADGKLSCQLYQRSCDVFLGLPFNIAS
                 ############################################################


                        190       200       210       220       230       240
                 =========+=========+=========+=========+=========+=========+
yruck0001_6520   YALLVHMMAQQCHLEVGDFVWTGGDTHLYNNHMEQTQLQLSREPRALPKLVIKRKPESLF
yinte0001_8460   YALLVHMMAQQCELDVGDFVWTGGDTHLYSNHIEQTNLQLSREPRALPKLIIKRKPASLF
yente0001X_9370  YALLVHMMAQQCDLAVGDFVWTGGDTHLYSNHIEQTHLQLSREPRALPKLIIKSKPASLF
ypseu0001X_3391  YALLVHMMAQQCDLAVGDFVWTGGDTHLYSNHIDQTHLQLSREPRALPKLVIKRKPDSLF
ypest0001X_9870  YALLVHMMAQQCDLAVGDFVWTGGDTHLYSNHIDQAHLQLSREPRVLPKLVIKRKPDSLF
ykris0001_8080   YALLVHMMAQQCDLEVGDFVWTGGDTHLYSNHIDQTNLQLSREPRALPKLIIKRKPDSLF
yfred0001_43650  YALLIHMMAQQCDLAVGDFVWTGGDTHLYSNHIEQTQLQLSREPRALPKLIIKRKPDSLF
yaldo0001_7160   YALLVHMMAQQCDLDVGDFVWTGGDTHLYSNHIEQTHLQLSREPRALPKLIIKRKPDSLF
yrohd0001_8480   YALLVHMMAQQCDLEVGDFVWTGGDTHLYSNHIEQTHLQLSREPRALPKLIIKRKPDSLF
yberc0001_6940   YALLIHMMAQQCDLDVGDFVWTGGDTHLYSNHIEQTHLQLSREPRALPKLIIKRKPDSLF
ymoll0001_7750   YALLIHMMAQQCDLEVGDFVWTGGDTHLYSNHIEQTHLQLSREPRALPKLIIKRKPDSLF
                 ############################################################


                        250       260
                 =========+=========+====
yruck0001_6520   DYHFEDFEIEGYDPHPAIKAPVAI
yinte0001_8460   DYRFEDFDIEGYDPHPGIKAPIAI
yente0001X_9370  DYRFEDFEIEGYDPHPGIKAPIAI
ypseu0001X_3391  DYHFDDFDIEGYDPHPGIKAPIAI
ypest0001X_9870  DYHFDDFDIEGYDPHPGIKAPIAI
ykris0001_8080   DYRFEDFEIEGYDPHPGIKAPIAI
yfred0001_43650  DYRFEDFEIEGYDPHPGIKAPIAI
yaldo0001_7160   DYRFDDFEIEGYDPHPGIKAPIAI
yrohd0001_8480   DYRFDDFEIEGYDPHPGIKAPIAI
yberc0001_6940   DYHFDDFEIEGYDPHPGIKAPIAI
ymoll0001_7750   DYHFDDFEIEGYDPHPGIKAPIAI
                 ########################
```

```
Parameters used
Minimum Number Of Sequences For A Conserved Position: 6
Minimum Number Of Sequences For A Flanking Position: 9
Maximum Number Of Contiguous Nonconserved Positions: 8
Minimum Length Of A Block: 10
Allowed Gap Positions: With Half
Use Similarity Matrices: Yes
```

```
Flank positions of the 1 selected block(s)
Flanks: [1  264]  

New number of positions in PGL1_unique_yersinia-CLUSTERS.dir/PGL1_unique_yersinia-CL1266/PGL1_unique_yersinia-CL1266.muscle.fasta.gblo:  264  (100% of the original 264 positions)
```
