# Supplementary material for: Genomic characterization of the Yersinia genus
Source: Genome Biol. 2010 Jan 4;11(1):R1. doi: 10.1186/gb-2010-11-1-r1 (PMC2847712; doi:10.1186/gb-2010-11-1-r1)
Supplement: Additional file 16 — The top level directory consists of a directory called Additional_cluster_files and 5010 directories, one for each multi-protein cluster family. (This top level directory has been split into three data files for uploading purposes (Additional files 15, 16, 17.) Within the directory are the following files: PGL1_unique_Yersinia_unclustered.out - list of all protein singletons that MCL did not group into a cluster (see Materials and Methods); PGL1_Yersinia_unique_locus_tags.txt - names of the 11 locus tag prefixes used for each genome; PGL1_unique_Yersinia.gff - mapping each Yersinia protein to a cluster in tab delimited GFF; PGL1_unique_Yersinia.sigfile - list of the longest protein in each cluster; PGL1_unique_Yersinia.summary - summary table of features of each of the clusters; PGL1_unique_Yersinia.table - summary table of each protein in the clusters. Within each cluster directory are the following files, where 'x' is the cluster name: PGL1_unique_Yersinia-x.faa - multifasta file of the proteins in the cluster; PGL1_unique_Yersinia-x.summary - summary of the properties of the proteins; PGL1_unique_Yersinia-x.matches - blast matches between the proteins of the cluster; PGL1_unique_Yersinia-x.muscle.fasta - muscle alignment of the proteins; PGL1_unique_Yersinia-x.muscle.fasta.gblo - gblocks output of muscle alignment (that is, auto-trimmed alignment); PGL1_unique_Yersinia-x.muscle.fasta.gblo.htm - as above in html format; PGL1_unique_Yersinia-x.muscle.tree - treefile from muscle alignment; PGL1_unique_Yersinia-x.sif - matches between proteins in simple interaction format for display on graphing software. [file gb-2010-11-1-r1-S16.zip › clusters2/PGL1_unique_yersinia-CL1267/PGL1_unique_yersinia-CL1267.muscle.fasta.gblo.htm]

PGL1\_unique\_yersinia-CL1267.muscle.fasta


## Gblocks 0.91b Results

Processed file: **PGL1\_unique\_yersinia-CL1267.muscle.fasta**  
Number of sequences: **11**  
Alignment assumed to be: **Protein**  
New number of positions: **290** (selected positions are underlined in blue)

```
                         10        20        30        40        50        60
                 =========+=========+=========+=========+=========+=========+
yruck0001_6510   ----------------------------MYLVGFVFAMWLAVRRANKPGSGWTKDEVENL
ypseu0001X_3392  MSNSYLAFPKFDPVIFSIGPVSLHWYGLMYLVGFVFAMWLAVRRANKPGSGWTKEEVENL
ypest0001X_9880  MSNSYLAFPKFDPVIFSIGPVSLHWYGLMYLVGFVFAMWLAVRRANKPGSGWTKEEVENL
yaldo0001_7170   MINSYLAFPKFDPVIFSIGPVSLHWYGLMYLVGFVFAMWLAVRRANKPGSGWTKEEVENL
yrohd0001_8470   MSNSYLAFPKFDPVIFSLGPVSLHWYGLMYLVGFVFAMWLAVRRANKPGSGWTKEEVENL
ymoll0001_7740   MSNSYLAFPKFDPVIFSIGPVSLHWYGLMYLVGFVFAMWLAVRRANKPGSGWTKEEVENL
yente0001X_9360  MSNSYLAFPKFDPVIFSIGPVSLHWYGLMYLVGFVFAMWLAVRRANKPGSGWTKEEVENL
yinte0001_8450   MSNSYLAFPKFDPVIFSIGPVSLHWYGLMYLVGFVFAMWLAVRRANKPGSGWTKEEVENL
ykris0001_8070   MSNSYLAFPKFDPVIFSIGPVSLHWYGLMYLVGFVFAMWLAVRRANKPGSGWTKEEVENL
yfred0001_43640  MSNSYLAFPKFDPVIFSIGPVSLHWYGLMYLVGFVFAMWLAVRRANKPGSGWTKEEVENL
yberc0001_6950   MSNSYLAFPKFDPVIFSIGPVSLHWYGLMYLVGFVFAMWLAVRRANKPGSGWTKEEVENL
                 ############################################################


                         70        80        90       100       110       120
                 =========+=========+=========+=========+=========+=========+
yruck0001_6510   LYAGFVGVFVGGRLGYVLFYNLPMFLDNPLYLFKVWDGGMSFHGGLVGVICVMLWFAHRT
ypseu0001X_3392  LYAGFLGVFIGGRVGYVLFYNLPMFLDNPLYLFKVWDGGMSFHGGLIGVICVMLWFARRT
ypest0001X_9880  LYAGFLGVFIGGRVGYVLFYNLPMFLDNPLYLFKVWDGGMSFHGGLIGVICVMLWFARRT
yaldo0001_7170   LYAGFLGVFVGGRVGYVLFYNLPMFLDNPLYLFKVWDGGMSFHGGLIGVICVMLWFARRT
yrohd0001_8470   LYAGFLGVFVGGRVGYVLFYNLPLFLDNPLYLFKVWDGGMSFHGGLIGVICVMMWFARRT
ymoll0001_7740   LYAGFLGVFVGGRVGYVLFYNLPVFLDNPLYLFKVWDGGMSFHGGLIGVICVMLWFARRT
yente0001X_9360  LYAGFLGVFVGGRVGYVLFYNLPLFLDNPLYLFKVWDGGMSFHGGLIGVICVMLWFARRT
yinte0001_8450   LYAGFLGVFVGGRVGYVLFYNLPMFLDNPLYLFKVWDGGMSFHGGLIGVICVMLWFARRT
ykris0001_8070   LYAGFLGVFVGGRVGYVLFYNLPMFLDNPLYLFKVWDGGMSFHGGLIGVICVMLWFARRT
yfred0001_43640  LYAGFLGVFVGGRVGYVLFYNLPMFLDNPLYLFKVWDGGMSFHGGLIGVICVMLWFARRT
yberc0001_6950   LYAGFLGVFVGGRVGYVLFYNLPMFLDNPLYLFKVWDGGMSFHGGLIGVICVMLWFARRT
                 ############################################################


                        130       140       150       160       170       180
                 =========+=========+=========+=========+=========+=========+
yruck0001_6510   KRHFFQVADFMAPLIPFGLGAGRLGNFINGELWGRVTTDTPWAMLFPASRSEDIALAAAD
ypseu0001X_3392  KRNFFQVADFIAPLIPFGLGAGRLGNFINAELWGRVTTDTPWAMLFPTSRNTDIAIVAAD
ypest0001X_9880  KRNFFQVADFIAPLIPFGLGAGRLGNFINAELWGRVTTDTPWAMLFPTSRNTDIAIVAAD
yaldo0001_7170   KRNFFQVADFIAPLIPFGLGAGRLGNFINGELWGRVTTDTPWAMLFPTSRSEDIAMVAAD
yrohd0001_8470   KRHFFQVSDFIAPLIPFGLGAGRLGNFINGELWGRVTTDAPWAMLFPTSRGEDIAIVAAD
ymoll0001_7740   KRNFFQVADFMAPLIPFGLGAGRLGNFINGELWGRVTTDTPWAMLFPTSRGEDIAIVAAD
yente0001X_9360  KRHFFQVADFIAPLIPFGLGAGRLGNFINGELWGRVTTDTPWAMLFPTSRGEDIAIVAAD
yinte0001_8450   KRHFFQVADFIAPLIPFGLGAGRLGNFINGELWGRVTTDTPWAMLFPTSRGEDIAIVAAD
ykris0001_8070   KRHFFQVADFMAPLIPFGLGAGRLGNFINGELWGRVTTDTPWAMLFPTSRGEDIAIVAAD
yfred0001_43640  KRHFFQVSDFMAPLIPFGLGAGRLGNFINGELWGRVTTDTPWAMLFPTSRGEDIAIVAAD
yberc0001_6950   KRHFFQVADFMAPLIPFGLGAGRLGNFINGELWGRVTTDTPWAMLFPTSRGEDIAIVAAD
                 ############################################################


                        190       200       210       220       230       240
                 =========+=========+=========+=========+=========+=========+
yruck0001_6510   P-QLQSILFQYGVLPRHPSQLYEMILEGVVLFIILNLFIRKPRPMGSVSGLFLIGYGFFR
ypseu0001X_3392  PAKWQAIFNQYGVLPRHPSQLYEMILEGVVLFIILNVFIRKPRPMGSVSGLFLIGYGTFR
ypest0001X_9880  PAKWQAIFNQYGVLPRHPSQLYEMILEGVVLFIILNVFIRKPRPMGSVSGLFLIGYGTFR
yaldo0001_7170   PAKWQAIFNQYGVLPRHPSQLYEMILEGVVLFIILNLFIRKPRPMGSVSGLFLIGYGVFR
yrohd0001_8470   PAKWQAIFNQYGVLPRHPSQLYEMILEGVVLFIILNLFIRKPRPMGSVSGLFLIGYGAFR
ymoll0001_7740   PAKWQAIFNQYGVLPRHPSQLYEMLLEGVVLFIILNLFIRKPRPMGSVSGLFLIGYGAFR
yente0001X_9360  PAKWQAIFNQYGVLPRHPSQLYEMILEGVVLFIILNLFIRKPRPMGSVSGLFLIGYGAFR
yinte0001_8450   PAKWQAIFNQYGVLPRHPSQLYEMILEGVVLFIILNLFIRKPRPMGSVSGLFLIGYGAFR
ykris0001_8070   PAKWQAIFNQYGVLPRHPSQLYEMILEGVVLFIILNLFIRKPRPMGSVSGLFLIGYGAFR
yfred0001_43640  PAKWQAIFNQYGVLPRHPSQLYEMLLEGVVLFIILNLFIRKPRPMGSVSGLFLIGYGTFR
yberc0001_6950   PAKWQAIFNQYGVLPRHPSQLYEMLLEGVVLFIILNLFIRKPRPMGSVSGLFLIGYGVFR
                 ############################################################


                        250       260       270       280       290
                 =========+=========+=========+=========+=========+
yruck0001_6510   IVVECFRQPDAQLGLFDGVISMGQILSVPMIVAGIIMMIWAYRRPQQQLS
ypseu0001X_3392  IIVECFRQPDEQLGLFEGMISMGQILSVPMILAGIIMMIWAYRRPTQKLS
ypest0001X_9880  IIVECFRQPDEQLGLFEGMISMGQILSVPMILAGIIMMIWAYRRPTQKLS
yaldo0001_7170   IVVEYFRQPDAQLGLFDGVISMGQLLSVPMILAGIIMMIWAYRRPAQQLS
yrohd0001_8470   IIVECFRQPDAQLGLFDGVISMGQILSLPMILAGIIMMIWAYRRPTQQLS
ymoll0001_7740   IIVEYFRQPDAQLGLFDGVISMGQILSIPMILAGIIMMIWAYRRPAQQLS
yente0001X_9360  IIVECFRQPDAQLGLFDGVISMGQILSVPMILAGIIMMIWAYRRPAQQLS
yinte0001_8450   IIVECFRQPDAQLGLFDGVISMGQILSVPMILAGIIMMIWAYRRPAQQLS
ykris0001_8070   IIVECFRQPDAQLGLFDGVISMGQILSVPMILAGIIMMIWAYRRPAQQLS
yfred0001_43640  IIVECFRQPDAQLGLFDGVISMGQILSVPMILAGIIMMIWAYRRPAQQLS
yberc0001_6950   IIVECFRQPDAQLGLFDGVISMGQILSVPMILAGIIMMIWAYRRPAQQLS
                 ##################################################
```

```
Parameters used
Minimum Number Of Sequences For A Conserved Position: 6
Minimum Number Of Sequences For A Flanking Position: 9
Maximum Number Of Contiguous Nonconserved Positions: 8
Minimum Length Of A Block: 10
Allowed Gap Positions: With Half
Use Similarity Matrices: Yes
```

```
Flank positions of the 1 selected block(s)
Flanks: [1  290]  

New number of positions in PGL1_unique_yersinia-CLUSTERS.dir/PGL1_unique_yersinia-CL1267/PGL1_unique_yersinia-CL1267.muscle.fasta.gblo:  290  (100% of the original 290 positions)
```
