# Supplementary material for: Genomic characterization of the Yersinia genus
Source: Genome Biol. 2010 Jan 4;11(1):R1. doi: 10.1186/gb-2010-11-1-r1 (PMC2847712; doi:10.1186/gb-2010-11-1-r1)
Supplement: Additional file 16 — The top level directory consists of a directory called Additional_cluster_files and 5010 directories, one for each multi-protein cluster family. (This top level directory has been split into three data files for uploading purposes (Additional files 15, 16, 17.) Within the directory are the following files: PGL1_unique_Yersinia_unclustered.out - list of all protein singletons that MCL did not group into a cluster (see Materials and Methods); PGL1_Yersinia_unique_locus_tags.txt - names of the 11 locus tag prefixes used for each genome; PGL1_unique_Yersinia.gff - mapping each Yersinia protein to a cluster in tab delimited GFF; PGL1_unique_Yersinia.sigfile - list of the longest protein in each cluster; PGL1_unique_Yersinia.summary - summary table of features of each of the clusters; PGL1_unique_Yersinia.table - summary table of each protein in the clusters. Within each cluster directory are the following files, where 'x' is the cluster name: PGL1_unique_Yersinia-x.faa - multifasta file of the proteins in the cluster; PGL1_unique_Yersinia-x.summary - summary of the properties of the proteins; PGL1_unique_Yersinia-x.matches - blast matches between the proteins of the cluster; PGL1_unique_Yersinia-x.muscle.fasta - muscle alignment of the proteins; PGL1_unique_Yersinia-x.muscle.fasta.gblo - gblocks output of muscle alignment (that is, auto-trimmed alignment); PGL1_unique_Yersinia-x.muscle.fasta.gblo.htm - as above in html format; PGL1_unique_Yersinia-x.muscle.tree - treefile from muscle alignment; PGL1_unique_Yersinia-x.sif - matches between proteins in simple interaction format for display on graphing software. [file gb-2010-11-1-r1-S16.zip › clusters2/PGL1_unique_yersinia-CL1268/PGL1_unique_yersinia-CL1268.muscle.fasta.gblo.htm]

PGL1\_unique\_yersinia-CL1268.muscle.fasta


## Gblocks 0.91b Results

Processed file: **PGL1\_unique\_yersinia-CL1268.muscle.fasta**  
Number of sequences: **11**  
Alignment assumed to be: **Protein**  
New number of positions: **745** (selected positions are underlined in blue)

```
                         10        20        30        40        50        60
                 =========+=========+=========+=========+=========+=========+
yruck0001_6500   MLTRLREVVEKVAMAASLTDALELLVNETCLAMNTEVCSIYLADNDRRCYYLMATRGLKK
ypest0001X_9890  --MRLREVVEKVAMATGLTEALELLVNETCLAMDTEVCSIYLADNDRRCYYLMATRGLKK
ypseu0001X_3393  --MRLREVVEKVAMATGLTEALELLVNETCLAMDTEVCSIYLADNDRRCYYLMATRGLKK
yberc0001_6960   --MRLREIVEKVAMATSLTDALELLVNETCLSMDTEVCSIYLADNDRRCYYLMATRGLKK
ymoll0001_7730   --MRLREIVEKVAMATSLTDALELLVNETCVAMDTEVCSIYLADNDRRCYYLMATRGLKK
yaldo0001_7180   MLTRLREIVEKVAMATSLTDALELLVNETCLAMDTEVCSIYLADNDRRCYYLMATRGLKK
yrohd0001_8460   --MRLREIVEKVAMATSLTDALELLVNETCLAMDTEVCSIYLADNDRRCYYLMATRGLKK
yfred0001_43630  MLTRLREIVEKVAMATSLTDALELLVNETCLAMDTEVCSIYLADNDRRCYYLMATRGLKK
yinte0001_8440   --MRLREIVEKVAMATSLADALELLVNETCLAMDTEVCSIYLADNDRRCYYLMATRGLKK
yente0001X_9350  MLTRLREIVEKVAMATSLTDALELLVNETCLAMDTEVCSIYLADNDRRCYYLMATRGLKK
ykris0001_8060   MLTRLREIVEKVAMATSLTDALELLVNETCLAMDTEVCSIYLADNDRRCYYLMATRGLKK
                    #########################################################


                         70        80        90       100       110       120
                 =========+=========+=========+=========+=========+=========+
yruck0001_6500   PRGRTITLAFDEGVVGLVGRLAEPINLADAQSHPSFKYVPQVKEDRFRAFLGVPIIYRRQ
ypest0001X_9890  PRGRTITLAFDEGIVGLVGRLAEPINLADAQSHPSFKYVPQVKEERYRAFLGVPIIYRRQ
ypseu0001X_3393  PRGRTITLAFDEGIVGLVGRLAEPINLADAQSHPSFKYVPLVKEERYRAFLGVPIIYRRQ
yberc0001_6960   PRGRIITLAFDEGIVGLVGRLAEPINLADAQSHPSFKYVPQVKEDRFRAFLGVPIIYRRQ
ymoll0001_7730   PRGRIITLAFDEGIVGLVGRLAEPINLADAQSHPSFKYVPQVKEDRFRAFLGVPIIYRRQ
yaldo0001_7180   PRGRTITLAFDEGIVGLVGRLAEPINLADAQSHPSFKYVPQVKEDRFRAFLGVPIIYRRQ
yrohd0001_8460   PRGRTITLAFDEGIVGLVGRLAEPINLADAQSHPSFKYVPQVKEDRFRAFLGVPIIYRRQ
yfred0001_43630  PRGRTITLAFDEGIVGLVGRLAEPINLADAQSHPSFKYVPQVKEDRFRAFLGVPIIYRRQ
yinte0001_8440   PRGRTITLAFDEGIVGLVGRLAEPINLADAQSHPSFKYVPQVKEDRFRAFLGVPIIYRRQ
yente0001X_9350  PRGRTITLAFDEGIVGLVGRLAEPINLADAQSHPSFKYVPQVKEDRFRAFLGVPIIYRRQ
ykris0001_8060   PRGRTITLAFDEGIVGLVGRLAEPINLADAQSHPSFKYVPQVKEDRFRAFLGVPIIYRRQ
                 ############################################################


                        130       140       150       160       170       180
                 =========+=========+=========+=========+=========+=========+
yruck0001_6500   LLGVLVVQQREHRQFDESEESFMVTLATQLAAILSQSQLNAIFGQYRQTRIRALAAAPGV
ypest0001X_9890  LLGVLVVQQREHRQFDESEESFMVTLATQLAGILSQSQLNAIFGQYRQTRIRALAAAPGV
ypseu0001X_3393  LLGVLVVQQREHRQFDESEESFMVTLATQLAGILSQSQLNAIFGQYRQTRIRALAAAPGV
yberc0001_6960   LLGVLVVQQREHRQFDESEESFMVTLATQLAGILSQSQLNAIFGQYRQTRIRALAAAPGV
ymoll0001_7730   LLGVLVVQQREHRQFDESEESFMVTLATQLAGILSQSQLNAIFGQYRQTRIRALAAAPGV
yaldo0001_7180   LLGVLVVQQREHRQFDESEESFMVTLATQLAGILSQSQLNAIFGQYRQTRIRALAAAPGV
yrohd0001_8460   LLGVLVVQQREHRQFDESEESFMVTLATQLAGILSQSQLNAIFGQYRQTRIRALAAAPGV
yfred0001_43630  LLGVLVVQQREHRQFDESEESFMVTLATQLAGILSQSQLNAIFGQYRQTRIRALAAAPGV
yinte0001_8440   LLGVLVVQQREHRQFDESEESFMVTLATQLAGILSQSQLNAIFGQYRQTRIRALAAAPGV
yente0001X_9350  LLGVLVVQQREHRQFDESEESFMVTLATQLAGILSQSQLNAIFGQYRQTRIRALAAAPGV
ykris0001_8060   LLGVLVVQQREHRQFDESEESFMVTLATQLAGILSQSQLNAIFGQYRQTRIRALAAAPGV
                 ############################################################


                        190       200       210       220       230       240
                 =========+=========+=========+=========+=========+=========+
yruck0001_6500   AVAEGWQDTSQPSLDLVYKASTLDSVRERERLTQALEEAAAEFRRFSKRFTASSQKESAA
ypest0001X_9890  AVAEGWQDISQPSLDLVYEASTLDSTLERERLTQALEEAAAEFRRFSKRFAASSQKESAA
ypseu0001X_3393  AVAEGWQDISQPSLDLVYEASTLDSTLERERLTQALEEAAAEFRRFSKRFAASSQKESAA
yberc0001_6960   AVAEGWQDTSQPSLDLVYEASTLDTVQERERLTQALEEAGAEFRRFSKRFAASSQKESAA
ymoll0001_7730   AVAEGWQDTSQPSLDLVYEASTLDTVQERERLTQALEEAAAEFRRFSKRFAASSQKESAA
yaldo0001_7180   AVAEGWQDTSQPSLDLVYEASTLDTAQERERLTQALEEAAAEFRRFSKRFAASSQKESAA
yrohd0001_8460   AVAEGWQDTSQPSLDLVYEASTLDPALERERLTQALEEAAAEFRRFSKRFAASSQKESAA
yfred0001_43630  AVAEGWQDTSQPSLDLVYEASTLDPVLERERLTQALEEAAAEFRRFSKRFAASSQKESAA
yinte0001_8440   AVAEGWQDTSQPSLDLVYEASTLDTVQERERLTQALEEAAAEFRRFSKRFAASSQKESAA
yente0001X_9350  AVAEGWQDTSQPSLDLVYEASTLDTAQERERLTQALEEAAAEFRRFSKRFAASSQKESAA
ykris0001_8060   AVAEGWQDTSQPSLDLVYEASTLDTAQERERLTQALEEAAAEFRRFSKRFAASSQKESAA
                 ############################################################


                        250       260       270       280       290       300
                 =========+=========+=========+=========+=========+=========+
yruck0001_6500   IFDLYSHLLNDARLKRELFAEIDAGSVAEWAVKQVVEQFAAQFASLQDTYMRERAGDLRA
ypest0001X_9890  IFDLYSHLLNDARLKRELFAQIDAGAVAEWAVKQVVEAFAAQFASLQDTYMRERASDLRA
ypseu0001X_3393  IFDLYSHLLNDARLKRELFAQIDAGAVAEWAVKQVVEAFAAQFASLQDTYMRERASDLRA
yberc0001_6960   IFDLYSHLLNDARLKRELFAQIDAGSVAEWAVKQVVEQFAAQFANLQDTYMRERASDLRA
ymoll0001_7730   IFDLYSHLLNDARLKRELFAQIDAGSVAEWAVKQVVEQFAAQFANLQDTYMRERASDLRA
yaldo0001_7180   IFDLYSHLLNDARLKRELFAQIDTGSVAEWAVKQVVEQFAAQFASLQDTYMRERASDLRA
yrohd0001_8460   IFDLYSHLLNDARLKRELFAQIDAGSVAEWAVKQVVEQFAAQFASLQDTYMRERGSDLRA
yfred0001_43630  IFDLYSHLLNDARLKRELFAQIDAGSVAEWAVKQVVEQFAAQFASLQDTYMRERASDLRA
yinte0001_8440   IFDLYSHLLNDARLKRELFAQIDAGSVAEWAVKQVVEQFAAQFASLQDTYMRERASDLRA
yente0001X_9350  IFDLYSHLLNDARLKRELFAQIDAGSVAEWAVKQVVEQFAAQFASLQDTYMRERASDLRA
ykris0001_8060   IFDLYSHLLNDARLKRELFAQIDAGSVAEWAVKQVVEQFAAQFASLQDTYMRERASDLRA
                 ############################################################


                        310       320       330       340       350       360
                 =========+=========+=========+=========+=========+=========+
yruck0001_6500   LGQRLLFHLDDSTSGTSQWPERFILVADELTATLLAEVPQDRLAGVVVRDGAANSHAAIL
ypest0001X_9890  LGQRLLFHLDDSTSGASQWPERFILVADELTATLLAEVPQDRLAGVVVRDGAANSHAAIL
ypseu0001X_3393  LGQRLLFHLDDSTSGASQWPERFILVADELTATLLAEVPQDRLAGVVVRDGAANSHAAIL
yberc0001_6960   LGLRLLFHLDDSTSGASQWPERFILVADELTATLLAEVPQDRLVGVVVRDGAANSHAAIL
ymoll0001_7730   LGLRLLFHLDDSTSGASQWPERFILVADELTATLLAEVPQDRLVGVVVRDGAANSHAAIL
yaldo0001_7180   LGQRLLFHLDDSTSGASQWPARFILVADELTATLLAEVPQDRLAGVVVRDGAANSHAAIL
yrohd0001_8460   LGQRLLFHLDDSTSGASQWPERFILVADELTATLLAEVPQDRLVGVVVRDGAANSHAAIL
yfred0001_43630  LGQRLLFHLDDSTSGASQWPERFILVADELTATLLAEVPQDRLAGVVVRDGAANSHAAIL
yinte0001_8440   LGQRLLFHLDDSTSGASQWPERFILVADELTATLLAEVPQDRLAGVVVRDGAANSHAAIL
yente0001X_9350  LGQRLLFHLDDSTSGASQWPERFILVADELTATLLAEVPQDRLAGVVVRDGAANSHAAIL
ykris0001_8060   LGQRLLFHLDDSTSGASQWPERFILVADELTATLLAEVPQDRLAGVVVRDGAANSHAAIL
                 ############################################################


                        370       380       390       400       410       420
                 =========+=========+=========+=========+=========+=========+
yruck0001_6500   VRAMGIPTVMGADIQPSLLSQRLLIVDGYRGEILVDPEPVLVQEYQRLITEEIELSKLAE
ypest0001X_9890  VRAMGIPTVMGADIQPALLSQRLLIVDGYRGEVLVDPEPVLVKEYQRLVTEEIELSKLAE
ypseu0001X_3393  VRAMGIPTVMGADIQPALLSQRLLIVDGYRGEVLVDPEPVLVKEYQRLVTEEIELSKLAE
yberc0001_6960   VRAMGIPTVMGADIQPALLSQRLLIVDGYRGEVLVDPEPVLIKEYQRLVTEEIELSKLAE
ymoll0001_7730   VRAMGIPTVMGADIQPALLNQRLLIVDGYRGEVLVDPEPVLVKEYQRLVTEEIELSKLAE
yaldo0001_7180   VRAMGIPTVMGADIQPALLNQRLLIVDGYRGEVLVDPEPVLVKEYQRLVTEEIELSKLAE
yrohd0001_8460   VRAMGIPTVMGADIQPALLNQRLLIVDGYRGEVLVDPEPVLVKEYQRLVTEEIELSKLAE
yfred0001_43630  VRAMGIPTVMGADIQPALLNQRLLIVDGYRGEVLVDPEPVLVKEYQRLVTEEIELSKLAE
yinte0001_8440   VRAMGIPTVMGADIQPALLNQRLLIVDGYRGEVLVDPEPVLVKEYQRLVTEEIELSKLAE
yente0001X_9350  VRAMGIPTVMGADIQPALLNQRLLIVDGYRGEVLVDPEPVLVKEYQRLVTEEIELSKLAE
ykris0001_8060   VRAMGIPTVMGADIQPALLNQRLLIVDGYRGEVLVDPEPVLVKEYQRLVTEEIELSKLAE
                 ############################################################


                        430       440       450       460       470       480
                 =========+=========+=========+=========+=========+=========+
yruck0001_6500   DDVEQPAELKSGERIQVLLNAGLSPEHEQLLGGRVDGVGLYRTEIPFMLQSGFPSEDEQV
ypest0001X_9890  DDVEQPAALKSGERIQVMLNAGLSPEHEQLLGGRVDGVGLYRTEIPFMLQSGFPSEEEQV
ypseu0001X_3393  DDVEQPAALKSGERIQVMLNAGLSPEHEQLLGGRVDGVGLYRTEIPFMLQSGFPSEEEQV
yberc0001_6960   DEVEQPAALKSGERIQVMLNAGLSPEHEKLLGGRVDGVGLYRTEIPFMLQSGFPSEEEQV
ymoll0001_7730   DDVEQPAALKSGERIQVMLNAGLSPEHEKLLGGRVDGVGLYRTEIPFMLQSGFPSEEEQV
yaldo0001_7180   DDVEQPAALKSGERIQVLLNAGLSPEHEQLLGGRVDGVGLYRTEIPFMLQSGFPSEEEQV
yrohd0001_8460   DDVEQPAALKSGERVQVMLNAGLSPEHEQLLGGRVDGVGLYRTEIPFMLQSGFPSEEEQV
yfred0001_43630  DDVEQPAALKSGERIQVMLNAGLSPEHEQLLGGRVDGVGLYRTEIPFMLQSGFPSEEEQV
yinte0001_8440   DDVEQPAALKSGERIQVMLNAGLSPEHEQLLGGRVDGVGLYRTEIPFMLQSGFPSEEEQV
yente0001X_9350  DDVEQPAALKSGERIQVMLNAGLSPEHEQLLGGRVDGVGLYRTEIPFMLQSGFPSEEEQV
ykris0001_8060   DDVEQPAALKSGERIQVMLNAGLSPEHEQLLGGRVDGVGLYRTEIPFMLQSGFPSEEEQV
                 ############################################################


                        490       500       510       520       530       540
                 =========+=========+=========+=========+=========+=========+
yruck0001_6500   AQYQGMLQLYPQKPVTLRTLDIGADKQLPYMPISEENPCLGWRGIRVTLDQPEIFLIQVR
ypest0001X_9890  AQYQGMLQLYPQKPVTLRTLDIGADKQLPYMPISEENPCLGWRGIRITLDQPEIFLIQVR
ypseu0001X_3393  AQYQGMLQLYPQKPVTLRTLDIGADKQLPYMPISEENPCLGWRGIRITLDQPEIFLIQVR
yberc0001_6960   AQYQGMLQLYPNKSVTLRTLDIGADKQLPYMPISEENPCLGWRGIRVTLDQPEIFLIQVR
ymoll0001_7730   SQYQGMLQLYPNKSVTLRTLDIGADKQLPYMPISEENPCLGWRGIRVTLDQPEIFLIQVR
yaldo0001_7180   AQYQGMLQLYPHKPVTLRTLDIGADKQLPYMPISEENPCLGWRGIRVTLDQPEIFLIQVR
yrohd0001_8460   AQYQGMLQLYPHKPVTLRTLDIGADKQLPYMPISEENPCLGWRGIRVTLDQPEIFLIQVR
yfred0001_43630  AQYQGMLQLYPNKPVTLRTLDIGADKQLPYMPISEENPCLGWRGIRVTLDQPEIFLIQVR
yinte0001_8440   AQYQGMLQLYPNKPVTLRTLDIGADKQLPYMPISEENPCLGWRGIRVTLDQPEIFLIQVR
yente0001X_9350  AQYQGMLQLYPNKPVTLRTLDIGADKQLPYMPISEENPCLGWRGIRVTLDQPEIFLIQVR
ykris0001_8060   TQYQGMLQLYPNKPVTLRTLDIGADKQLPYMPISEENPCLGWRGIRVTLDQPEIFLIQVR
                 ############################################################


                        550       560       570       580       590       600
                 =========+=========+=========+=========+=========+=========+
yruck0001_6500   AMLRANAGTGNLGILLPMITSLEEVDEAKRLIDRAGREVQEVLGYELPKPRLGVMIEVPA
ypest0001X_9890  AMLRANAGTGNLGILLPMITSIEEVDEAKRLIDRAGREVEEMLGYVLPQPRLGVMIEVPS
ypseu0001X_3393  AMLRANAGTGNLGILLPMITSIEEVDEAKRLIDRAGREVEEMLGYVLPQPRLGVMIEVPS
yberc0001_6960   AMLRANAGTGNLGILLPMITSLEEVDEAKRLIDRAGREVQEVLGYELPQPKLGVMIEVPA
ymoll0001_7730   AMLRANASTGNLGILLPMITSLEEVDEAKRLIDRAGREVQEVLGYGLPQPKLGVMIEVPA
yaldo0001_7180   AMLRANAGTGNLGILLPMITSLEEVDEAKRLIDRAGREVQEVLGYELPQPKLGVMIEVPA
yrohd0001_8460   AMLRANAGTGNLGILLPMITSLEEVDEAKRLIDRAGREVEEVLGYALPQPKLGVMVEVPA
yfred0001_43630  AMLRANAGTGNLGILLPMITSLEEVDEAKRLIDRAGREVQEVLGYELPQPKLGVMIEVPA
yinte0001_8440   AMLRANAGTGNLGILLPMITSLEEVDEAKRLIDRAGREVQEVLGYELPQPKLGVMIEVPA
yente0001X_9350  AMLRANAGTGNLGILLPMITSLEEVDEAKRLIDRAGREVQEVLGYELPQPKLGVMVEVPA
ykris0001_8060   AMLRANAGTGNLGILLPMITSLEEVDEAKRLIDRAGREVQELLGYALPQPKLGVMVEVPA
                 ############################################################


                        610       620       630       640       650       660
                 =========+=========+=========+=========+=========+=========+
yruck0001_6500   MIFMLPYLKSRVDFISVGTNDLTQYLLAVDRNNTRVASLYDSLHPAMLQVLRQILVQATD
ypest0001X_9890  MIFMLPYLTSRVDFISVGTNDLTQYLLAVDRNNTRVASLYDSLHPAMLQVLRQILMQATE
ypseu0001X_3393  MIFMLPYLTSRVDFISVGTNDLTQYLLAVDRNNTRVASLYDSLHPAMLQVLRQILMQATE
yberc0001_6960   MIFMLPYLKSRVDFISVGTNDLTQYLLAVDRNNTRVASLYDSLHPAVLQVLSHILTQATQ
ymoll0001_7730   MIFMLPYLKSRVDFISVGTNDLTQYLLAVDRNNTRVASLYDSLHPAVLQVLSHILTQATQ
yaldo0001_7180   MIFMLPYLKSRVDFISVGTNDLTQYLLAVDRNNTRVASLYDNLHPAMLQVLKHILTQATQ
yrohd0001_8460   MIFMLPYLKSRVDFISVGTNDLTQYLLAVDRNNTRVASLYDSLHPAMLQVLNHILTQATQ
yfred0001_43630  MIFMLPYLKSRVDFISVGTNDLTQYLLAVDRNNTHVASLYDSLHPAMLQVLNHILTQATQ
yinte0001_8440   MIFMLPYLKSRVDFISVGTNDLTQYLLAVDRNNTRVASLYDSLHPAMLQVLSHILTQATL
yente0001X_9350  MIFMLPYLKSRVDFISVGTNDLTQYLLAVDRNNTRVASLYDSLHPAMLQVLSHILAQATQ
ykris0001_8060   MIFMLPYLKSRVDFISVGTNDLTQYLLAVDRNNTRVASLYDSLHPAMLQVLSHILTQATQ
                 ############################################################


                        670       680       690       700       710       720
                 =========+=========+=========+=========+=========+=========+
yruck0001_6500   SGLQVSLCGEMAGDPMGALLLVGLGYRQLSMNGRSVARIKYLLRNVDLVEAEALAERVLT
ypest0001X_9890  SGLQVSLCGEMAGDPMGALLLVGLGYRNLSMNGRSVARIKYLLRNIELADAQVLAARVLT
ypseu0001X_3393  SGLQVSLCGEMAGDPMGALLLVGLGYRNLSMNGRSVARIKYLLRNIELADAQALAARVLT
yberc0001_6960   AGLQVSLCGEMAGDPMGALLLVGLGYRNLSMNGRSVARIKYLLRNIDLADAQALAERVLT
ymoll0001_7730   SGLQVSLCGEMAGDPMGALLLVGLGYRNLSMNGRSVARIKYLLRNIDLADAQALAERVLT
yaldo0001_7180   SGLQVSLCGEMAGDPMGALLLVGLGYRNLSMNGRSVARIKYLLRNIELADAEALVERVLA
yrohd0001_8460   SGLQVSLCGEMAGDPMGALLLVGLGYRNLSMNGRSVARIKYLLRNIDLADAQALAERVLA
yfred0001_43630  SGLQVSLCGEMAGDPMGALLLVGLGYRNLSMNGRSVARIKYLLRNIELADAQALAERVLT
yinte0001_8440   SGLQVSLCGEMAGDPMGALLLVGLGYRNLSMNGRSVARIKYLLRNIDLADATALAERVLS
yente0001X_9350  SGLQVSLCGEMAGDPMGALLLVGLGYRNLSMNGRSVARIKYLLRNIDLVDAQALAERVLN
ykris0001_8060   SGLQVSLCGEMAGDPMGALLLVGLGYRNLSMNGRSVARIKYLLRNIDLVDAQALAERVLS
                 ############################################################


                        730       740
                 =========+=========+========
yruck0001_6500   AQMTTDVRHLTAAFMERRGLGGLIRGGK
ypest0001X_9890  AQMTTDVRHLTAAFMERRGLGGLIRGGK
ypseu0001X_3393  AQMTTDVRHLTAAFMERRGLGGLIRGGK
yberc0001_6960   AQMTTDVRHLTAAFMERRGLGGLIRGGK
ymoll0001_7730   AQMTTDVRHLTAAFMERRGLGGLIRGGK
yaldo0001_7180   AQMTTDVRHLTAAFMERRGLGGLIRGGK
yrohd0001_8460   AQMTTDVRHLTAAFMERRGLGGLIRGGK
yfred0001_43630  AQMTTDVRHLTAAFMERRGLGGLIRGGK
yinte0001_8440   AQMTTDVRHLTAAFMERRGLGGLIRGGK
yente0001X_9350  AQMTTDVRHLTAAFMERRGLGGLIRGGK
ykris0001_8060   AQMTTDVRHLTAAFMERKGLGGLIRGGK
                 ############################
```

```
Parameters used
Minimum Number Of Sequences For A Conserved Position: 6
Minimum Number Of Sequences For A Flanking Position: 9
Maximum Number Of Contiguous Nonconserved Positions: 8
Minimum Length Of A Block: 10
Allowed Gap Positions: With Half
Use Similarity Matrices: Yes
```

```
Flank positions of the 1 selected block(s)
Flanks: [4  748]  

New number of positions in PGL1_unique_yersinia-CLUSTERS.dir/PGL1_unique_yersinia-CL1268/PGL1_unique_yersinia-CL1268.muscle.fasta.gblo:  745  (99% of the original 748 positions)
```
