# Supplementary material for: Genomic characterization of the Yersinia genus
Source: Genome Biol. 2010 Jan 4;11(1):R1. doi: 10.1186/gb-2010-11-1-r1 (PMC2847712; doi:10.1186/gb-2010-11-1-r1)
Supplement: Additional file 16 — The top level directory consists of a directory called Additional_cluster_files and 5010 directories, one for each multi-protein cluster family. (This top level directory has been split into three data files for uploading purposes (Additional files 15, 16, 17.) Within the directory are the following files: PGL1_unique_Yersinia_unclustered.out - list of all protein singletons that MCL did not group into a cluster (see Materials and Methods); PGL1_Yersinia_unique_locus_tags.txt - names of the 11 locus tag prefixes used for each genome; PGL1_unique_Yersinia.gff - mapping each Yersinia protein to a cluster in tab delimited GFF; PGL1_unique_Yersinia.sigfile - list of the longest protein in each cluster; PGL1_unique_Yersinia.summary - summary table of features of each of the clusters; PGL1_unique_Yersinia.table - summary table of each protein in the clusters. Within each cluster directory are the following files, where 'x' is the cluster name: PGL1_unique_Yersinia-x.faa - multifasta file of the proteins in the cluster; PGL1_unique_Yersinia-x.summary - summary of the properties of the proteins; PGL1_unique_Yersinia-x.matches - blast matches between the proteins of the cluster; PGL1_unique_Yersinia-x.muscle.fasta - muscle alignment of the proteins; PGL1_unique_Yersinia-x.muscle.fasta.gblo - gblocks output of muscle alignment (that is, auto-trimmed alignment); PGL1_unique_Yersinia-x.muscle.fasta.gblo.htm - as above in html format; PGL1_unique_Yersinia-x.muscle.tree - treefile from muscle alignment; PGL1_unique_Yersinia-x.sif - matches between proteins in simple interaction format for display on graphing software. [file gb-2010-11-1-r1-S16.zip › clusters2/PGL1_unique_yersinia-CL1269/PGL1_unique_yersinia-CL1269.muscle.fasta.gblo.htm]

PGL1\_unique\_yersinia-CL1269.muscle.fasta


## Gblocks 0.91b Results

Processed file: **PGL1\_unique\_yersinia-CL1269.muscle.fasta**  
Number of sequences: **11**  
Alignment assumed to be: **Protein**  
New number of positions: **221** (selected positions are underlined in blue)

```
                         10        20        30        40        50        60
                 =========+=========+=========+=========+=========+=========+
yruck0001_6470   --------------------------------MSAYSQSPAPPVDEHQLFERAQRLAGFT
yberc0001_6980   --------------------------------MSVYSLPPAPPTDEHQLFQRAQALSGFT
ykris0001_8040   --------------------------------MSVYSLPPAPPTDEHQLFQRAEALSGFT
yaldo0001_7210   --------------------------------MSVYSLPPAPPTNEHQLFQRAEALSGFT
ypest0001X_9910  --------------------------------MSVYSLPPAPPSDEHQLFQRAQALSGFT
ypseu0001X_3395  --------------------------------MSVYSLPPAPPSDEHQLFQRAQALSGFT
yente0001X_9320  --------------------------------MSVYSLPPAPPTDEHQLFQRAEALSGFT
yrohd0001_8430   MLLLISAVLDNNTSTVTFVSACFLTDFSRFPAMSVYSLPPAPPTDEHQLFQRAQALSGFT
yinte0001_8420   --------------------------------MSVYSLPPAPPTDEHQLFQRAQALSGFT
ymoll0001_7710   --------------------------------MSVYSLPPAPPTDEHQLFQRAQALSGFT
yfred0001_43600  --------------------------------MSVYSLPPAPPTDEHQLFQRAQALSGFT
                                                 ############################


                         70        80        90       100       110       120
                 =========+=========+=========+=========+=========+=========+
yruck0001_6470   LGELATRAGWPIPVDLKRDKGWVGMLLEFYLGANAGSKPEQDFANIGIELKTIPINGQGK
yberc0001_6980   LGELAARAQWLIPADLKRVKGWVGMLLEFYLGASAGSKPEQDFADIGIELKTIPISGQGK
ykris0001_8040   LGELAIRAGWNIPADLKRVKGWVGMLLEFYLGASAGSKPEQDFADIGIELKTIPISAQGK
yaldo0001_7210   LGELATRAGWNIPADLKRVKGWVGMLLEFYLGASAGSKPEQDFADIGIELKTIPISAQGK
ypest0001X_9910  LGELATRAQWVIPADLKRVKGWVGMLLEFYLGASAGSKPEQDFADIGIELKTIPISAQGK
ypseu0001X_3395  LGELATRAQWVIPADLKRVKGWVGMLLEFYLGASAGSKPEQDFADIGIELKTIPISAQGK
yente0001X_9320  LGELASKAGWNIPADLKRIKGWVGMLLEFYLGASAGSKPEQDFADIGIELKTIPISAQGK
yrohd0001_8430   LGELASQAQWVIPADLKRVKGWVGMLLEFYLGASAGSKPEQDFADIGIELKTIPISAQGK
yinte0001_8420   LGELAARAQWVIPADLKRVKGWVGMLLEFYLGASAGSKPEQDFADIGIELKTIPISAQGK
ymoll0001_7710   LGELATRAQWVIPADLKRVKGWVGMLLEFYLGASAGSKPEQDFADIGIELKTIPISGQGK
yfred0001_43600  LGELATRAQWVIPADLKRVKGWVGMLLEFYLGASAGSKPEQDFADIGIELKTIPISAQGK
                 ############################################################


                        130       140       150       160       170       180
                 =========+=========+=========+=========+=========+=========+
yruck0001_6470   PLETTFVCVAPLTGNSGVTWESSHVRHKLARVLWIPVEGERQIPLAERRIGAPLLWSPNT
yberc0001_6980   PLETTFVCVAPLTGNSGITWENSHVRHKLARVLWVPVEGERHIPLAERRVGAPLLWSPNA
ykris0001_8040   PLETTFVCVAPLTGNSGITWESSHVRHKLARVLWVPVEGERQIPLAQRRVGAPLLWSPNE
yaldo0001_7210   PLETTFVCVAPLTGNSGVTWESSHVRHKLARVLWVPVEGERLIPLAQRRVGAPLLWSPSA
ypest0001X_9910  PLETTFVCVAPLTGNSGVTWESSHVRHKLARVLWVPVEGERHIPLAERRVGAPLLWSPNV
ypseu0001X_3395  PLETTFVCVAPLTGNSGVTWENSHVRHKLARVLWVPVEGERHIPLAERRVGAPLLWSPNV
yente0001X_9320  PLETTFVCVAPLTGNSGITWESSHVRHKLARVLWVPVEGERQIPLAQRRVGAPLLWSPNE
yrohd0001_8430   PLETTFVCVAPLTGNSGVTWESSHVRHKLARVLWVPVEGERQIPLAERRVGAPLLWSPNE
yinte0001_8420   PLETTFVCVAPLSGNSGITWESSHVRHKLARVLWVPVEGERQIPLAERRVGAPLLWSPNA
ymoll0001_7710   PLETTFVCVAPLTGNSGITWENSHVRHKLARVLWVPVEGERQIPLAERRVGAPLLWSPNA
yfred0001_43600  PLETTFVCVAPLTGNSGITWESSHVRHKLTRVLWVPVEGERQIPLAERRVGAPLLWSPNE
                 ############################################################


                        190       200       210       220       230       240
                 =========+=========+=========+=========+=========+=========+
yruck0001_6470   EEEERLRRDWEELMDLIVLGKVESITARHGEVLQLRPKAANSRALTEAVGEKGQPIMTLP
yberc0001_6980   QEDELLRRDWEELMDLIVLGRVETITARHGEVLQLRPKAANSRALTEAIGEQGQPIMTLP
ykris0001_8040   EEEELLRCDWEELMDLIVLGKVETITARHGEVLQLRPKAANSRALTEAIGEHGQPIMTLP
yaldo0001_7210   QEEELLRRDWEELMDLIVLGKVESITARHGEVLQLRPKAANSRALTEAIGEHGQPIMTLP
ypest0001X_9910  EEEELLRRDWEELMDLIVLGKVESITARHGQVLQLRPKAANSRALTEAIGEFGQPIMTLP
ypseu0001X_3395  EEEELLRRDWEELMDLIVLGKVESITARHGQVLQLRPKAANSRALTEAIGEFGQPIMTLP
yente0001X_9320  EEEELLRRDWEELMDLIVLGKVETITARHGEVLQLRPKAANSRALTEAIGEHGQPIMTLP
yrohd0001_8430   EEEELLRRDWEELMDLIVLGKVETITARHGQVLQLRPKAANSRALTEAIGEQGQPIMTLP
yinte0001_8420   QEEELLRRDWEELMDLIVLGKVETITARHGEVLQLRPKAANSRALTEAIGEKGQPIMTLP
ymoll0001_7710   EEEELLRRDWEELMDLIVLGKVESITARHGEVLQLRPKAANSRALTEAIGEQGQPIMTLP
yfred0001_43600  EEEELLRRDWEELMDLIVLGKVETITARHGEVLQLRPKAANSRALTEAIGEQGQPIMTLP
                 ############################################################


                        250       260
                 =========+=========+=====
yruck0001_6470   RGFYLKKTFTGPL-LARHFLL----
yberc0001_6980   RGFYLKKPLPDLCWLATSYCKISPK
ykris0001_8040   RGFYLKKPLPARC-SLGTFCFNRRI
yaldo0001_7210   RGFYLKKTFTGPM-LARHFLL----
ypest0001X_9910  RGFYLKKTLTAPM-LARHFLL----
ypseu0001X_3395  RGFYLKKTLTAPM-LARHFLL----
yente0001X_9320  RGFYLKKTFTGPM-LARHFLL----
yrohd0001_8430   RGFYLKKTFTGPM-LARHFLL----
yinte0001_8420   RGFYLKKTFTGPM-LARHFLL----
ymoll0001_7710   RGFYLKKTFTGPM-LARHFLL----
yfred0001_43600  RGFYLKKTFTGPM-LARHFLL----
                 #############
```

```
Parameters used
Minimum Number Of Sequences For A Conserved Position: 6
Minimum Number Of Sequences For A Flanking Position: 9
Maximum Number Of Contiguous Nonconserved Positions: 8
Minimum Length Of A Block: 10
Allowed Gap Positions: With Half
Use Similarity Matrices: Yes
```

```
Flank positions of the 1 selected block(s)
Flanks: [33  253]  

New number of positions in PGL1_unique_yersinia-CLUSTERS.dir/PGL1_unique_yersinia-CL1269/PGL1_unique_yersinia-CL1269.muscle.fasta.gblo:  221  (83% of the original 265 positions)
```
