# Supplementary material for: Genomic characterization of the Yersinia genus
Source: Genome Biol. 2010 Jan 4;11(1):R1. doi: 10.1186/gb-2010-11-1-r1 (PMC2847712; doi:10.1186/gb-2010-11-1-r1)
Supplement: Additional file 16 — The top level directory consists of a directory called Additional_cluster_files and 5010 directories, one for each multi-protein cluster family. (This top level directory has been split into three data files for uploading purposes (Additional files 15, 16, 17.) Within the directory are the following files: PGL1_unique_Yersinia_unclustered.out - list of all protein singletons that MCL did not group into a cluster (see Materials and Methods); PGL1_Yersinia_unique_locus_tags.txt - names of the 11 locus tag prefixes used for each genome; PGL1_unique_Yersinia.gff - mapping each Yersinia protein to a cluster in tab delimited GFF; PGL1_unique_Yersinia.sigfile - list of the longest protein in each cluster; PGL1_unique_Yersinia.summary - summary table of features of each of the clusters; PGL1_unique_Yersinia.table - summary table of each protein in the clusters. Within each cluster directory are the following files, where 'x' is the cluster name: PGL1_unique_Yersinia-x.faa - multifasta file of the proteins in the cluster; PGL1_unique_Yersinia-x.summary - summary of the properties of the proteins; PGL1_unique_Yersinia-x.matches - blast matches between the proteins of the cluster; PGL1_unique_Yersinia-x.muscle.fasta - muscle alignment of the proteins; PGL1_unique_Yersinia-x.muscle.fasta.gblo - gblocks output of muscle alignment (that is, auto-trimmed alignment); PGL1_unique_Yersinia-x.muscle.fasta.gblo.htm - as above in html format; PGL1_unique_Yersinia-x.muscle.tree - treefile from muscle alignment; PGL1_unique_Yersinia-x.sif - matches between proteins in simple interaction format for display on graphing software. [file gb-2010-11-1-r1-S16.zip › clusters2/PGL1_unique_yersinia-CL1270/PGL1_unique_yersinia-CL1270.muscle.fasta.gblo.htm]

PGL1\_unique\_yersinia-CL1270.muscle.fasta


## Gblocks 0.91b Results

Processed file: **PGL1\_unique\_yersinia-CL1270.muscle.fasta**  
Number of sequences: **11**  
Alignment assumed to be: **Protein**  
New number of positions: **346** (selected positions are underlined in blue)

```
                         10        20        30        40        50        60
                 =========+=========+=========+=========+=========+=========+
yruck0001_6450   --------------MLGLGTMTFGEQNSEADAHAQLDYAIAAGVNLIDTAEMYPVPPRPE
yaldo0001_7240   MQYHRIPHSTLEVSLLGLGTMTFGEQNSEADAHAQLDYAVAAGINLIDTAEMYPVPPKPE
ypseu0001X_3396  MQYHRIPHSSLEVSLLGLGTMTFGEQNSEADAHAQLDYAVAAGINLIDTAEMYPVPPRPE
ypest0001X_9920  MQYHRIPHSSLEVSLLGLGTMTFGEQNSEADAHAQLDYAVAAGINLIDTAEMYPVPPRPE
ykris0001_8020   MQYHRIPHSSLEVSLLGLGTMTFGEQNSEADAHEQLDYAVAAGINLIDTAEMYPVPPKPE
yrohd0001_8410   MQYHRIPHSSLEVSRLGLGTMTFGEQNSEADAHAQLDYAVAAGINLIDTAEMYPVPPKPE
yente0001X_9300  MQYHRIPHSSLEVSLLGLGTMTFGEQNSEADAHAQLDYAVAAGINLIDTAEMYPVPPKPE
yfred0001_43580  MQYHRIPHSSLEVSLLGLGTMTFGEQNSEADAHAQLDYAVAAGVNLIDTAEMYPVPPKPE
yinte0001_8400   MQYHRIPHSSLEVSLLGLGTMTFGEQNSEADAHAQLDYAVAAGINLIDTAEMYPVPPRPE
yberc0001_7000   MQYHRIPHSSLEVSLLGLGTMTFGEQNSEADAHAQLDYAVAAGINLIDTAEMYPVPPRPE
ymoll0001_7690   MQYHRIPHSSLEVSLLGLGTMTFGEQNSEADAHAQLDYAVAAGINLIDTAEMYPVPPRPE
                 ############################################################


                         70        80        90       100       110       120
                 =========+=========+=========+=========+=========+=========+
yruck0001_6450   TQGLTEQYIGSWLKARGCREKIILASKVSGPSRGNDQPIRPGMALDRKNIRAALDASLKR
yaldo0001_7240   TQGLTEQYIGSWIKARGSRDKIILASKVSGPSRGNDQPIRPNMALDRQNIRKALEDSLKR
ypseu0001X_3396  TQGLTEQYIGRWIKARGCREKIILASKVSGPSRGDDQPIRPNMALDRKNIRIALEESLKR
ypest0001X_9920  TQGLTEQYIGRWIKARGCREKIILASKVSGPSRGDDQPIRPNMALDRKNIRIALEESLKR
ykris0001_8020   TQGLTEQYIGSWIKARGSRDKIILASKVSGPSRGSDQPIRPNMALDRKNIREALDASLKR
yrohd0001_8410   TQGLTEQYIGNWIKARGCRDKIILASKISGPSRGSDQPIRPNMALDRKNIRIALEDSLRR
yente0001X_9300  TQGLTEQYIGSWIKARGSRDKIILASKVSGPSRGNDKPIRPNMALDRKNIRVALEDSLRR
yfred0001_43580  TQGLTEQYIGSWIKARGSRDKIILASKVSGPSRGSDKPIRPNMALDRKNIRAALDESLKR
yinte0001_8400   TQGLTEQYIGNWIKARGERDKIILASKVSGPSRGSDQPIRPNMALDRKNIRIALEDSLRR
yberc0001_7000   TQGLTEQYIGNWIKARGSRDKIILASKVSGPSRGSDQPIRPNMALDRKNIRIALEDSLRR
ymoll0001_7690   TQGLTEQYIGNWIKARGSRDKIILASKVSGPSRGSDQPIRPNMALDRKNIRIALEDSLRR
                 ############################################################


                        130       140       150       160       170       180
                 =========+=========+=========+=========+=========+=========+
yruck0001_6450   LNTDYLDLYQLHWPQRETNCFGKLNYRYSNDTATVTLLETLEALNEQVRAGKIRYIGVSN
yaldo0001_7240   LNTDYIDLYQLHWPQRETNCFGKLNYRYSENTVPVTLLETLEALNEQVRAGKIRYIGVSN
ypseu0001X_3396  LNTDYLDIYQLHWPQRETNCFGKLNYRYSEQTAVVTLLETLEALNEQVRAGKIRYIGVSN
ypest0001X_9920  LNTDYLDIYQLHWPQRETNCFGKLNYRYSEQTAVVTLLETLEALNEQVRAGKIRYIGVSN
ykris0001_8020   LNTDYIDLYQLHWPQRETNCFGKLNYRYSEQTAVITLLETLEALNEQVRAGKIRYIGVSN
yrohd0001_8410   LNTDYLDIYQLHWPQRETNCFGKLNYHYSEQTAVVTLLETLEALTEQVRAGKIRYIGVSN
yente0001X_9300  LNTDYLDIYQLHWPQRETNCFGKLNYRYSEQTAVVTLLETLEALNEQVRAGKIRYIGVSN
yfred0001_43580  LNTDYLDLYQLHWPQRETNCFGKLNYRYSEQTAVVTLLETLEALNEQVRAGKIRYIGVSN
yinte0001_8400   LNTDYIDIYQLHWPQRETNCFGKLNYRYSEHTAVVTLLETLEALNEQVRAGKIRYIGVSN
yberc0001_7000   LNTDYLDIYQLHWPQRETNCFGKLNYRYSEQTAVVTLLETLEALNEQVRAGKIRYIGVSN
ymoll0001_7690   LNTDYLDIYQLHWPQRDTNCFGKLNYRYSEQTAVVTLLETLEALNEQVRAGKIRYIGVSN
                 ############################################################


                        190       200       210       220       230       240
                 =========+=========+=========+=========+=========+=========+
yruck0001_6450   ETPWGVMRYLQLAEKHDLPRIVSIQNPYSLLNRSFEVGLAEISQHEGIELLAYSSLAFGT
yaldo0001_7240   ETPWGVMRYLKLADKHDLPRIVSIQNPYSLLNRSFEVGLAEISQHEGVELLAYSSLAFGT
ypseu0001X_3396  ETPWGVMRYLQLAEKHDLPRIVSIQNPYSLLNRSFEVGLAEISQHEGVELLAYSSLAFGT
ypest0001X_9920  ETPWGVMRYLQLAEKHDLPRIVSIQNPYSLLNRSFEVGLAEISQHEGVELLAYSSLAFGT
ykris0001_8020   ETPWGVMRYLHLAEKHDLPRIVSIQNPYSLLNRSFEVGLAEISQHEGVELLAYSSLAFGT
yrohd0001_8410   ETPWGVMRYLQLAEKHDLPRIVSIQNPYSLLNRSFEVGLAEISQHEGVELLAYSSLAFGT
yente0001X_9300  ETPWGVMRYLQLAEKHDLPRIVSIQNPYSLLNRSFEVGLAEISQHEGVELLAYSSLAFGT
yfred0001_43580  ETPWGVMRYLQLAEKHDLPRIVSIQNPYSLLNRSFEVGLAEISQHEGVELLAYSSLAFGT
yinte0001_8400   ETPWGVMRYLQLAEKHDLPRIVSIQNPYSLLNRSFEVGLAEISQHEGVELLAYSSLAFGT
yberc0001_7000   ETPWGVMRYLQLAEKHDLPRIVSIQNPYSLLNRSFEVGLAEISQHEGVELLAYSSLAFGT
ymoll0001_7690   ETPWGVMRYLQLAEKHDLPRIVSIQNPYSLLNRSFEVGLAEISQHEGIELLAYSSLAFGT
                 ############################################################


                        250       260       270       280       290       300
                 =========+=========+=========+=========+=========+=========+
yruck0001_6450   LSGKYLNGAKPADARNTLFSRFTRYSSPQAQLAIAEYVVLAQKHGLDPAQMALAFVRQQP
yaldo0001_7240   LSGKYLNGAQPASARNTLYSRFTRYTGSQAQLAVAEYVALAQRYGLDPAQMALAFVRQQP
ypseu0001X_3396  LSGKYLNGAKPAGARNTLFSRFTRYSGPQTQLAVAEYVSLAKHHGLDPAQMALAFVRQQP
ypest0001X_9920  LSGKYLNGAKPAGARNTLFSRFTRYSGPQTQLAVAEYVSLAKHHGLDPAQMALAFVRQQP
ykris0001_8020   LSGKYLNGAQPAGARNTLFSRFTRYTGPQAQLAIAEYVALAKRHGLDPAQMALAFVRQQP
yrohd0001_8410   LSGKYLNGAQPAGARNTLYSRFTRYTGPQAQLAVAEYVALAKRHGLDPAQMALAFVRQQP
yente0001X_9300  LSGKYLNGAQPAGARNTLFSRFTRYTGPQAQLATAEYVALAKRHGLDPAQMALAFVRQQP
yfred0001_43580  LSGKYLNGAQPAGARNTLFSRFTRYTGPQAQLAIAEYVALAKRHGLDPAQMALAFVRQQP
yinte0001_8400   LSGKYLNGAKPAAARNTLFSRFTRYTGPQSQLAVAEYVALAKRHGLDPAQMALAFVRQQP
yberc0001_7000   LSGKYLNGAKPAGARNTLFSRFTRYTGSQPQLAVAEYVALAKRHGLDPAQMALAFVRQQP
ymoll0001_7690   LSGKYLNGAKPAGARNTLFSRFTRYTGPQSQLAVAEYVALAKRHGLDPAQMALAFVRQQP
                 ############################################################


                        310       320       330       340
                 =========+=========+=========+=========+======
yruck0001_6450   FVASTLLGATTLPQLQSNLDSLNIVLDEEILNELEEIHTRYTFPAP
yaldo0001_7240   FVASTLLGATSMAQLKSNIDSQDVILSQEVLTALEEIHTRFTFPAP
ypseu0001X_3396  FVASTLLGATSLEQLKSNIDSQNIVLSQEVLDALEAIHTRYTFPAP
ypest0001X_9920  FVASTLLGATSLEQLKSNIDSQNIVLSQEVLDALEAIHTRYTFPAP
ykris0001_8020   FVASTLLGATTLEQLKSNIDSQDVVLAPEILKALEEIHTRFTFPAP
yrohd0001_8410   FVASTLLGATTLEQLKSNIDSQQVVLSQEVLHALEEIHTRFTFPAP
yente0001X_9300  FVASTLLGATTLEQLKSNIDSQDVVLSQEVLNALEEIHTRFTFPAP
yfred0001_43580  FVASTLLGATTLEQLKSNIDSQNVVLSQEVLNALEEIHTRFTFPAP
yinte0001_8400   FVASTLLGATTLEQLKSNIDSQNLVLDQEVLNALEEIHTRFTFPAP
yberc0001_7000   FVASTLLGATTLEQLKSNIDSQNVVLSQEVLDALEEIHTRFTFPAP
ymoll0001_7690   FVASTLLGATSLEQLKSNIDSQNVVLSQEVLEALEEIHTRFTFPAP
                 ##############################################
```

```
Parameters used
Minimum Number Of Sequences For A Conserved Position: 6
Minimum Number Of Sequences For A Flanking Position: 9
Maximum Number Of Contiguous Nonconserved Positions: 8
Minimum Length Of A Block: 10
Allowed Gap Positions: With Half
Use Similarity Matrices: Yes
```

```
Flank positions of the 1 selected block(s)
Flanks: [1  346]  

New number of positions in PGL1_unique_yersinia-CLUSTERS.dir/PGL1_unique_yersinia-CL1270/PGL1_unique_yersinia-CL1270.muscle.fasta.gblo:  346  (100% of the original 346 positions)
```
