# Supplementary material for: Genomic characterization of the Yersinia genus
Source: Genome Biol. 2010 Jan 4;11(1):R1. doi: 10.1186/gb-2010-11-1-r1 (PMC2847712; doi:10.1186/gb-2010-11-1-r1)
Supplement: Additional file 16 — The top level directory consists of a directory called Additional_cluster_files and 5010 directories, one for each multi-protein cluster family. (This top level directory has been split into three data files for uploading purposes (Additional files 15, 16, 17.) Within the directory are the following files: PGL1_unique_Yersinia_unclustered.out - list of all protein singletons that MCL did not group into a cluster (see Materials and Methods); PGL1_Yersinia_unique_locus_tags.txt - names of the 11 locus tag prefixes used for each genome; PGL1_unique_Yersinia.gff - mapping each Yersinia protein to a cluster in tab delimited GFF; PGL1_unique_Yersinia.sigfile - list of the longest protein in each cluster; PGL1_unique_Yersinia.summary - summary table of features of each of the clusters; PGL1_unique_Yersinia.table - summary table of each protein in the clusters. Within each cluster directory are the following files, where 'x' is the cluster name: PGL1_unique_Yersinia-x.faa - multifasta file of the proteins in the cluster; PGL1_unique_Yersinia-x.summary - summary of the properties of the proteins; PGL1_unique_Yersinia-x.matches - blast matches between the proteins of the cluster; PGL1_unique_Yersinia-x.muscle.fasta - muscle alignment of the proteins; PGL1_unique_Yersinia-x.muscle.fasta.gblo - gblocks output of muscle alignment (that is, auto-trimmed alignment); PGL1_unique_Yersinia-x.muscle.fasta.gblo.htm - as above in html format; PGL1_unique_Yersinia-x.muscle.tree - treefile from muscle alignment; PGL1_unique_Yersinia-x.sif - matches between proteins in simple interaction format for display on graphing software. [file gb-2010-11-1-r1-S16.zip › clusters2/PGL1_unique_yersinia-CL1271/PGL1_unique_yersinia-CL1271.muscle.fasta.gblo.htm]

PGL1\_unique\_yersinia-CL1271.muscle.fasta


## Gblocks 0.91b Results

Processed file: **PGL1\_unique\_yersinia-CL1271.muscle.fasta**  
Number of sequences: **11**  
Alignment assumed to be: **Protein**  
New number of positions: **386** (selected positions are underlined in blue)

```
                         10        20        30        40        50        60
                 =========+=========+=========+=========+=========+=========+
yruck0001_6440   MNQPAVAINKPLLSRGMMAVLGAQFFSAFGDNALLFVTLAVIKQQLYPDWSQPILQMAFV
ypseu0001X_3399  ----------------MVAVLCAQFFSAFGDNALLFATLALIKQQLYPDWSQPILQMAFV
ypest0001X_9940  ----------------MVAVLCAQFFSAFGDNALLFATLALIKQQLYPDWSQPILQMAFV
yaldo0001_7250   MNQQVLT-DKPLLSRGMVAVLCAQFFSAFGDNALLFATLALIKQQLYPDWSQPILQMAFV
yinte0001_8390   MSQQALS-DKPLLSRGMIAVLCAQFFSAFGDNALLFATLALIKQQLYPDWSQPILQMAFV
yrohd0001_8400   MSQPVIT-DKPLLSRGMMAVLGAQFFSAFGDNALLFATLALIKQQLYPDWSQPILQMAFV
yfred0001_43570  MSQQALA-EKPLLSRGMIAVLCAQFFSAFGDNALLFATLALIKQQLYPDWSQPILQMAFV
yberc0001_7020   MSQQAIA-DKPLLSRGMIAVLCAQFFSAFGDNALLFATLALIKQQLYPDWSQPILQMAFV
yente0001X_9280  MSQQALA-DKPLLSRGMIAVLCAQFFSAFGDNALLFATLALIKQQLYPDWSQPILQMAFV
ymoll0001_7680   MSQQIIA-DKPLLSRGMVAVLCAQFFSAFGDNALLFATLALIKQQLYPDWSQPILQMAFV
ykris0001_8010   --------------------------------------LALIKQQLYPDWSQPILQMAFV
                                 ############################################


                         70        80        90       100       110       120
                 =========+=========+=========+=========+=========+=========+
yruck0001_6440   ATYIVLAPFVGQFADSFAKGRVMMTANGLKLAGALVIFFGFNPFLGYTLVGIGAAAYSPA
ypseu0001X_3399  ATYIVLAPFVGQIADGFAKGRVMMVANGLKLAGALVICFGLNPFLGYSLVGVGAAAYSPA
ypest0001X_9940  ATYIVLAPFVGQIADGFAKGRVMMVANGLKLAGALVICFGLNPFLGYSLVGVGAAAYSPA
yaldo0001_7250   ATYIILAPFVGQVADSFAKGRVMMFANGLKLAGALVICFGLNPFLGYSLVGIGAAAYSPA
yinte0001_8390   ATYIILAPFVGQVADSFAKGRVMMVANGLKLAGALVICFGLNPFLGYSLVGVGAAAYSPA
yrohd0001_8400   ATYIILAPFVGQFADSFAKGRVMMVANGLKLAGALVICFGFNPFLGYSLVGVGAAAYSPA
yfred0001_43570  ATYIILAPFVGQFADSFAKGRVMMVANGLKLAGALVICFGFNPFLGYSLVGVGAAAYSPA
yberc0001_7020   ATYIILAPFVGQFADSFAKGRVMMVANGLKLAGALVICFGFNPFLGYSLVGVGAAAYSPA
yente0001X_9280  ATYIVLAPFVGQFADSFAKGRVMMVANGLKLAGALVICFGFNPFLGYSLVGVGAAAYSPA
ymoll0001_7680   ATYIILAPFVGQFADSFAKGRVMMVANGLKLAGALVICFGFNPFLGYSLVGVGAAAYSPA
ykris0001_8010   ATYIILAPFVGQFADSFAKGRVMMVANGLKLAGALVICFGFNPFLGYSLVGVGAAAYSPA
                 ############################################################


                        130       140       150       160       170       180
                 =========+=========+=========+=========+=========+=========+
yruck0001_6440   KYGILGEMTSGDKLVKANGLMEASTIAAILMGSVAGGVLADWNLSVALGLCVLVYSAAVV
ypseu0001X_3399  KYGILGEITSGEQLVKANGMMEASTIAAILLGSVAGGILADWHLMAALGVCALVYAIAVI
ypest0001X_9940  KYGILGEITSGEQLVKANGMMEASTIAAILLGSVAGGILADWHLMAALGVCALVYAIAVI
yaldo0001_7250   KYGILGEITSGEQLVKANGMMEASTIAAILLGSVAGGVLADWHLMAALGVCALVYAIAVV
yinte0001_8390   KYGILGEITSGEQLVKANGMMEASTIAAILLGSVAGGVLADWHLIAALSVCALVYAIAVV
yrohd0001_8400   KYGILGEITSGEQLVKANGMMEASTIAAILLGSVAGGVLADWHLSVALGVCALVYAIAVV
yfred0001_43570  KYGILGEITSGDQLVKANGMMEASTIAAILLGSVAGGVLADWHLVVALGVCALVYAIAVV
yberc0001_7020   KYGILGEITSGEQLVKANGMMEASTIAAILLGSVAGGVLADWNLMAALGACALVYAIAVV
yente0001X_9280  KYGILGEITSGEQLVKANGMMEASTIAAILLGSVAGGVLADWHLGVALGVCALVYAIAVV
ymoll0001_7680   KYGILGEITSGEQLVKANGMMEASTIAAILLGSVAGGVLADWHLMVALGVCALVYAIAVV
ykris0001_8010   KYGILGEITSGEQLVKANGMMEASTIAAILLGSVAGGVLADWHLGAALAVCALVYAIAVV
                 ############################################################


                        190       200       210       220       230       240
                 =========+=========+=========+=========+=========+=========+
yruck0001_6440   ANLFIPRLSAARGGLSWWPTTMSRSFFAASKTLWRNGETRFSLVGTSLFWGAGVTLRFLL
ypseu0001X_3399  ANLFIPRLAAARSGASWRPRAMTGSFFTACRLLWQDSETRFSLAGTSLFWGAGVTLRFLL
ypest0001X_9940  ANLFIPRLAAARSGASWRPRAMTGSFFTACRLLWQDSETRFSLAGTSLFWGAGVTLRFLL
yaldo0001_7250   ANLFIPRLSAARSGRSWQPRAMTGNFLSACQILWRDGETRFSLAGTSLFWGAGVTLRFLL
yinte0001_8390   ANLFIPHLAAARSGSSWRPRAMTGSFFAACRILWRDGETRFSLAGTSLFWGAGVTLRFLL
yrohd0001_8400   ANMFIPRLAAARAGSSWRPGAMTGSFFAACRILWRDGETRFSLAGTSLFWGAGVTLRFLL
yfred0001_43570  ANMFIPRLAAARSGHSWRPTAMTGSFFAACRILWRDGETRFSLAGTSLFWGAGVTLRFLL
yberc0001_7020   ANLFIPRLAAARVGSSWRPRAMTSSFFTACRLLWRDGETRFSLAGTSLFWGAGVTLRFLL
yente0001X_9280  ANMFIPKLAAARSGSSWRPRAMTGSFFTACLVLWRDGEARFSLAGTSLFWGAGVTLRFLL
ymoll0001_7680   ANLFIPRLAAARSGSSWRPRAMTGSFFTACRLLWRDGETRFSLAGTSLFWGAGVTLRFLL
ykris0001_8010   ANMFIPRLAAARSGSSWRPRAMTGSFFAACRILWRDGETRFSLAGTSLFWGAGVTLRFLL
                 ############################################################


                        250       260       270       280       290       300
                 =========+=========+=========+=========+=========+=========+
yruck0001_6440   VLWVPVALGIIDNATPTVLNATVAIGIVIGAAAAARFVTLKTVKRCMPAGILIGVAVAIF
ypseu0001X_3399  VLWVPVALGIADNATPTLLNAMVAIGIVVGAGAAARFVTLKTVKRCLPAGVLIGVMVTIF
ypest0001X_9940  VLWVPVALGIADNATPTLLNAMVAIGIVVGAGAAARFVTLKTVKRCLPAGVLIGVMVTIF
yaldo0001_7250   VLWVPVALGIADNATPTLLNAMVAVGIVLGAGAAARFVTLKTVKRCLPAGVLIGVAVAIF
yinte0001_8390   VLWVPVALGIADNATPTLLNAMVAIGIVIGAGAAARFVTLKTVKRCLPAGVLIGVVVATF
yrohd0001_8400   VLWVPIALGITDNATPTLLNAMVAIGIVVGAGAAARFVTLKTVKRCLPAGVLIGVFVAIF
yfred0001_43570  VLWVPIALGITDNATPTLLNAMVAIGIVLGAGAAARFVTLKTVKRCLPAGVLIGVVVAIF
yberc0001_7020   VLWVPIALGITDNTTPTLLNAMVAIGIVVGAGAAARFVTLKTVKRCLPAGVLIGGVVAIF
yente0001X_9280  VLWVPIALGITDNATPTLLNAMVAIGIVVGAGAAARFVTLKTVKRCLPAGVLIGVAVAIF
ymoll0001_7680   VLWVPIALGIADNATPTLLNAMVAIGIVVGAGAAARFVTLKTVKRCLPAGVLIGVMVAIF
ykris0001_8010   VLWVPIALGITDNTTPTLLNAMVAIGIVVGAGAAARFVTLKTVKRCLPAGVLIGVAVAIF
                 ############################################################


                        310       320       330       340       350       360
                 =========+=========+=========+=========+=========+=========+
yruck0001_6440   SLQYTMPMAYLFLVSIGILGGFFVVPLNALLQERGKKSVGAGNAIAVQNLGENTAMLLML
ypseu0001X_3399  SLQNSMPMAYLLLIIIGILGGFFVVPLNALLQERGKHSVGAGNAIAVQNLGENTAMLFML
ypest0001X_9940  SLQNSMPMAYLLLIIIGILGGFFVVPLNALLQERGKHSVGAGNAIAVQNLGENTAMLFML
yaldo0001_7250   SLQHSMPMAYLLLIIIGILGGFFVVPLNALLQERGKNSVGAGNAIAVQNLGENTAMLLML
yinte0001_8390   SLQHSMPMAYLLLIIIGILGGFFVVPLNALLQERGRQSVGAGNAIAVQNLGENTAMLLML
yrohd0001_8400   ALQHSMPMAYLLLIIIGVLGGFFVVPLNALLQERGKHSVGAGNAIAVQNLGENTAMLLML
yfred0001_43570  ALQHSMPMAYLLLIIIGILGGFFVVPLNALLQERGKQSVGAGNAIAVQNLGENTAMLLML
yberc0001_7020   ALQHSMPMAYLLLIIIGILGGFFVVPLNALLQERGKQSVGAGNAIAVQNLGENTAMLLML
yente0001X_9280  ALQHSMPMAYLLLIIIGILGGFFVVPLNALLQERGKNSVGAGNAIAVQNLGENTAMLLML
ymoll0001_7680   ALQHSMPMAYLLLIIIGILGGFFVVPLNALLQERGKQSVGAGNAIAVQNLGENTAMLLML
ykris0001_8010   ALQHSMPMAYLLLIIIGILGGFFVVPLNALLQERGKNSVGAGNAIAVQNLGENTAMLVML
                 ############################################################


                        370       380       390       400
                 =========+=========+=========+=========+=======
yruck0001_6440   GLYSVVIKLGAPVVAVGVGFGVIFALAITVLWIWQWEQERKIS----
ypseu0001X_3399  GLYSLVVKLGAPVVAVGVGFGVVFALAIALLWGWQWRQQRQKTRQPE
ypest0001X_9940  GLYSLVVKLGAPVVAVGVGFGVVFALAIALLWGWQWRQQRQKTRQPE
yaldo0001_7250   GLYSVVVKLGVPVVAVGIGFGVIFALAIALLWGWQWRQQRRKVAK--
yinte0001_8390   GLYSVVVKLGVPVVVVGVGFGVIFALAIALLWCWQWQQLRRKAAK--
yrohd0001_8400   GLFSVVVKLGVPVIAVGVGFGVVFALAISLLWFWQWRQLRHKVTK--
yfred0001_43570  GLFSVVVKLGVPVIAVGVGFGVVFALAIALLWGWQWRQLRRKTAE--
yberc0001_7020   GLFSMVVKLGVPVVAVGVGFGVIFALAIALLWGWQWWQLRHKTVE--
yente0001X_9280  GLFSVVVKLGVPVIAVGVGFGVIFALAIALLWGWQWRQQRQKTAE--
ymoll0001_7680   GLFSVVVKLGVPVIAVGVGFGVIFALAIALLWGWQWRQLRSKAVE--
ykris0001_8010   GLFSVVVKLGVPVVAVGVGFGVIFALAIALLWGWQWRQQRRKAV---
                 ##########################################
```

```
Parameters used
Minimum Number Of Sequences For A Conserved Position: 6
Minimum Number Of Sequences For A Flanking Position: 9
Maximum Number Of Contiguous Nonconserved Positions: 8
Minimum Length Of A Block: 10
Allowed Gap Positions: With Half
Use Similarity Matrices: Yes
```

```
Flank positions of the 1 selected block(s)
Flanks: [17  402]  

New number of positions in PGL1_unique_yersinia-CLUSTERS.dir/PGL1_unique_yersinia-CL1271/PGL1_unique_yersinia-CL1271.muscle.fasta.gblo:  386  (94% of the original 407 positions)
```
