# Supplementary material for: Genomic characterization of the Yersinia genus
Source: Genome Biol. 2010 Jan 4;11(1):R1. doi: 10.1186/gb-2010-11-1-r1 (PMC2847712; doi:10.1186/gb-2010-11-1-r1)
Supplement: Additional file 16 — The top level directory consists of a directory called Additional_cluster_files and 5010 directories, one for each multi-protein cluster family. (This top level directory has been split into three data files for uploading purposes (Additional files 15, 16, 17.) Within the directory are the following files: PGL1_unique_Yersinia_unclustered.out - list of all protein singletons that MCL did not group into a cluster (see Materials and Methods); PGL1_Yersinia_unique_locus_tags.txt - names of the 11 locus tag prefixes used for each genome; PGL1_unique_Yersinia.gff - mapping each Yersinia protein to a cluster in tab delimited GFF; PGL1_unique_Yersinia.sigfile - list of the longest protein in each cluster; PGL1_unique_Yersinia.summary - summary table of features of each of the clusters; PGL1_unique_Yersinia.table - summary table of each protein in the clusters. Within each cluster directory are the following files, where 'x' is the cluster name: PGL1_unique_Yersinia-x.faa - multifasta file of the proteins in the cluster; PGL1_unique_Yersinia-x.summary - summary of the properties of the proteins; PGL1_unique_Yersinia-x.matches - blast matches between the proteins of the cluster; PGL1_unique_Yersinia-x.muscle.fasta - muscle alignment of the proteins; PGL1_unique_Yersinia-x.muscle.fasta.gblo - gblocks output of muscle alignment (that is, auto-trimmed alignment); PGL1_unique_Yersinia-x.muscle.fasta.gblo.htm - as above in html format; PGL1_unique_Yersinia-x.muscle.tree - treefile from muscle alignment; PGL1_unique_Yersinia-x.sif - matches between proteins in simple interaction format for display on graphing software. [file gb-2010-11-1-r1-S16.zip › clusters2/PGL1_unique_yersinia-CL1272/PGL1_unique_yersinia-CL1272.muscle.fasta.gblo.htm]

PGL1\_unique\_yersinia-CL1272.muscle.fasta


## Gblocks 0.91b Results

Processed file: **PGL1\_unique\_yersinia-CL1272.muscle.fasta**  
Number of sequences: **11**  
Alignment assumed to be: **Protein**  
New number of positions: **332** (selected positions are underlined in blue)

```
                         10        20        30        40        50        60
                 =========+=========+=========+=========+=========+=========+
yaldo0001_38840  -----MIMAKARLHDDAMVQLLMEDPEFAQVYLHQALLDIDEEGGQEAFLM---------
ymoll0001_9150   ---MQEVAKKAGVSKATVSRVLSGKGYVSEATKKQVYTAIEEAGYRPNLLARNLATNKSE
yberc0001_37040  ---MQEVAKKAGVSKATVSRVLSGKGYVSEATKEQVYKAIEEAGYRPNLLARNLATNKSE
yinte0001_24330  ---MQEVAKKAGVSKATVSRVLSGKGYVSEVTKAQVYKAIEESGYRPNLLARNLATNKSQ
yrohd0001_37830  ---MQEVAKKAGVSKATVSRVLSGKGYVSPATKDQVFKAIDETGYRPNLLARNLATNKSE
yfred0001_25650  ---MQEVAKKAGVSKATVSRVLSGKGYVSETTKEQVFKAIEETGYRPNLLARNLATNKSE
yente0001X_3069  ---MQEVAKKAGVSKATVSRVLSGKGYVSDATKESVFKAIEETGYRPNLLARNLATNKSE
ykris0001_25450  ---MQEVAKKAGVSKATVSRVLSGKGYVSDATKNQVFKAIEETGYRPNLLARNLATNKSE
yinte0001_8350   ------------------------------------------------------------
yaldo0001_7280   MVTMLDVSLRAGVSKATVSRVLNGTGQVKESTRKAVFNAMDELGYRPNFLAQSLANKSSN
yfred0001_43530  MITMLDVSLRAGVSKATVSRVLNGTGQVKESTREAVFKAMDELGYRPNFLAQSLANKSSN
                    #########################################################


                         70        80        90       100       110       120
                 =========+=========+=========+=========+=========+=========+
yaldo0001_38840  ------------------------------------------------ALRRVVEARGG-
ymoll0001_9150   CIGFVVTNTLYNGNYFNEILSQAAKKLESSGRQLVLVDGKHSAEEEQAAIQFLLDLRCDA
yberc0001_37040  CIGFVVTNTLYNGNYFNEILSQAAKKLEDSGRQLVLVDGKHSAEEEQAAIQFLLDLRCDA
yinte0001_24330  CIGLVVTNTLYNGNYFNEILSQAAKKLENNGRQLVLVDGKHSAEEEQEAIQFLLDLRCDA
yrohd0001_37830  CIGFVVTNTLYSGSYFNEILSQAAQKLESNGRQLVLVDGKHSAQEEQEAIQFLLDLRCDA
yfred0001_25650  CIGLVVTNTLYNGSYFNEILSQAAQKLEQNGRQLVLVDGKHSAEEEQEAIQFLLDLRCDA
yente0001X_3069  CIGLVVTNTLYNGSYFNEILSQAAQKLEKNGRQLVLVDGKHSAEEEQEAIQFLLDLRCDA
ykris0001_25450  CIGLVVTNTLYNGSYFNEILSQAAQKLENNGRQLVLVDGKHSAEEEQEAIQFLLDLRCDA
yinte0001_8350   --------------------------------------MHNCRD-----IRQLCHKNCP-
yaldo0001_7280   SIGLVVSN--FDGPYFGRLLRRAAKLTEASGKHLIVTDGHDTPEGEQQAVRLLADRRCDA
yfred0001_43530  SIGLVVSN--FEGPYFGRLLHQAAKLIEASGKHLIVTDGHDTPEGEQQAVQLLADRRCDA
                 ############################################################


                        130       140       150       160       170       180
                 =========+=========+=========+=========+=========+=========+
yaldo0001_38840  ------------------------------------------------------------
ymoll0001_9150   IIIYPRFLTVDAMDLIIDKHKQPIMVVNRKLRKNHSHSIFCDHKGSSYHATKYLIEQGHR
yberc0001_37040  IIIYPRFLTVDAMDLIIDKHKQPIMVVNRKLRKNHSHSIFCDHKGSSYNATNYLIEQGHR
yinte0001_24330  IIIYPRFLTVDDMDVIIDKYKQPIMVVNRKLRKNHSHCIFCDHKGSSYNATKYLIERGHQ
yrohd0001_37830  IIIYPRFLTVDALDLIIDKYKQPIMVVNRKLRKNHSHCISCDHKGSSYSATQYLIEHGHQ
yfred0001_25650  IIIYPRFLTVDDIDLIIDKYKQPIMVVNRKLRKNHSHCIFCDHKGSSYTATQYLIERGHQ
yente0001X_3069  IIIYPRFLTVDAMDLIIDQYKQPIMVVNRKLRKNHSHCIFCDHKGSSYNATKYLIDQGHR
ykris0001_25450  IIIYPRFLTVDAMDLIIDKYKQPIMVVNRKLRKNYSHCIFCDHKGSSYNATKYLIDQGHQ
yinte0001_8350   ------------------------------------------------------------
yaldo0001_7280   IILYTRFMSETDLMQLLSSLPTPLMVINRDLPQLRERCVFFEQQQAAFDAVTYLIQQGHR
yfred0001_43530  IILYTRSMSEADLMQLLANVPTPLMVINRDLPQAREHCVFFEQQQAAFNAVNYLIEQGHR
                 ############################################################


                        190       200       210       220       230       240
                 =========+=========+=========+=========+=========+=========+
yaldo0001_38840  -----MASVAKKAGVSRETLYRTL-------------------SPSGNPTLKTLLS----
ymoll0001_9150   DIAFITGSLDSPTAIERLSGYKEALIAHSLPVRDTLIAQGKWTPHSGSAAVESLLAHQLP
yberc0001_37040  DIAFITGSLDSPTAIERLSGYKAALMANSLIVRETLIAPGRWTPQSGFAAVESLLASQLP
yinte0001_24330  DIAFITGSLDSPTAIERLSGYKEALRACSLPLRDSLIIQGKWTPLSGATAVESLLASQQP
yrohd0001_37830  DIAFITGSLDSPTAIERLSGYKAALIDFSLPVRDELIVQGKWTPTSGAAAVELLLTHKSP
yfred0001_25650  DIAFITGSLDSPTAIERLSGYKAALEASSLPVRDKLIAQGKWTPASGAAAIEYLLSSKLP
yente0001X_3069  DIAFITGSLDSPTAIERLSGYKTALAAFSLPIPDKLIAQGKWTPASGATAVESLLASQLP
ykris0001_25450  DIAFITGSLDSPTAIERLSGYKAALTEFSLPVQDKLIVQGKWTPASGATAIESLLTSQLP
yinte0001_8350   ---------TTSTAQSRLAGYQQALVTHRIALNEDLIIHGDSGVPDGYQGCQAL------
yaldo0001_7280   DIACITGPITTPTAQSRLAGYQQALAEQGITLDKDLIAYGDSSVPGGYDGCQVLLARNIP
yfred0001_43530  DIACITGPIATSTAQSRLAGYQQALTRHGIPLNDELIAHGENGVPDGYKGCQTLLARNIK
                 ############################################################


                        250       260       270       280       290       300
                 =========+=========+=========+=========+=========+=========+
yaldo0001_38840  ----------------------------VVSATGFQFSHIASITA---------------
ymoll0001_9150   LSAILASNDDMAIGAIKKLNEVGIRVPDEISIIGFDNIPLAPFLSPALSSIKDPVSSMVN
yberc0001_37040  FSAIIASNDDMAIGAMKKLTEVGIRVPEEISIIGFDNIPLAPFLSPSLSSIKDPVSSMIN
yinte0001_24330  FSAIIASNDDMAIGAMKRLNEMGINIPEAVSLIGFDNIPIAPFLSPSLSSIKDPVSGMIN
yrohd0001_37830  FSAIIASNDDMAIGAMKKLNESGINVPEEISIIGFDNILIAPFLLPPLSSIKDPVSDMIN
yfred0001_25650  FSAVIASNDDMAIGAMKRLNEVGMKIPEDISIIGFDNIPLAPFLLPPLSSIKDPVSGMIN
yente0001X_3069  FSAIIASNDDMAIGAMKKLNEAGLKVPEDVSIIGFDNIPIAPFLLPPLSSIKDPVSGMIN
ykris0001_25450  FSAIIASNDDMAIGAMKKLNEAGLKIPEDISIIGFDNIPIAPFLLPPLSSIKDPVSGMIN
yinte0001_8350   ---------KLIAGSIKCK-----------------------------------------
yaldo0001_7280   FSALFANNDDMTIGAMKALNQAGKRLPQDVSLFGFDDEPSAPYLQPALSTVYLPIDAMIE
yfred0001_43530  FSALFIYNDDMAIGAMKALSQAGKKLPEDVSLFGFDDEPTAPYLQPSLSTVYLPIDAMIE
                 ############################################################


                        310       320       330       340
                 =========+=========+=========+=========+=====
yaldo0001_38840  ---------------------------------------------
ymoll0001_9150   EVINRLISMLDGGYLSNENLFQSDLILRESVSHGPFFSQKTI---
yberc0001_37040  EVINRLISMLDGGYLSNENRFQSDLMLRDSVSYGPFFSPKTS---
yinte0001_24330  DVINRLISMLDGGYLSTENIFQSELIVRDSVGDGPYLGQSALNHQ
yrohd0001_37830  EVINRLISMLDGGYLSNENTFQSDLIIRDSVANGPFYQIKAK---
yfred0001_25650  EVIHRLISMLDGGYLSQENIFQSDLIIRDSVGDGPFLTR------
yente0001X_3069  EVINRLISMLDGGYLSNENIFQSELFIRDSVGKGPFFTENKVK--
ykris0001_25450  EVINRLISMLDGGYLSNENIFQSELLIRDSVSKGPFFNSLKAK--
yinte0001_8350   ---------------------------------------------
yaldo0001_7280   AAITQALKLIAGQEVHPLAPFIGELKLRESVVPGPYYGQS-----
yfred0001_43530  AAITQALRLIAGKKVLPLTPFTGELKLRESVVPGPYYC-------
                 ###################################
```

```
Parameters used
Minimum Number Of Sequences For A Conserved Position: 6
Minimum Number Of Sequences For A Flanking Position: 9
Maximum Number Of Contiguous Nonconserved Positions: 8
Minimum Length Of A Block: 10
Allowed Gap Positions: With Half
Use Similarity Matrices: Yes
```

```
Flank positions of the 1 selected block(s)
Flanks: [4  335]  

New number of positions in PGL1_unique_yersinia-CLUSTERS.dir/PGL1_unique_yersinia-CL1272/PGL1_unique_yersinia-CL1272.muscle.fasta.gblo:  332  (96% of the original 345 positions)
```
