# Supplementary material for: Genomic characterization of the Yersinia genus
Source: Genome Biol. 2010 Jan 4;11(1):R1. doi: 10.1186/gb-2010-11-1-r1 (PMC2847712; doi:10.1186/gb-2010-11-1-r1)
Supplement: Additional file 16 — The top level directory consists of a directory called Additional_cluster_files and 5010 directories, one for each multi-protein cluster family. (This top level directory has been split into three data files for uploading purposes (Additional files 15, 16, 17.) Within the directory are the following files: PGL1_unique_Yersinia_unclustered.out - list of all protein singletons that MCL did not group into a cluster (see Materials and Methods); PGL1_Yersinia_unique_locus_tags.txt - names of the 11 locus tag prefixes used for each genome; PGL1_unique_Yersinia.gff - mapping each Yersinia protein to a cluster in tab delimited GFF; PGL1_unique_Yersinia.sigfile - list of the longest protein in each cluster; PGL1_unique_Yersinia.summary - summary table of features of each of the clusters; PGL1_unique_Yersinia.table - summary table of each protein in the clusters. Within each cluster directory are the following files, where 'x' is the cluster name: PGL1_unique_Yersinia-x.faa - multifasta file of the proteins in the cluster; PGL1_unique_Yersinia-x.summary - summary of the properties of the proteins; PGL1_unique_Yersinia-x.matches - blast matches between the proteins of the cluster; PGL1_unique_Yersinia-x.muscle.fasta - muscle alignment of the proteins; PGL1_unique_Yersinia-x.muscle.fasta.gblo - gblocks output of muscle alignment (that is, auto-trimmed alignment); PGL1_unique_Yersinia-x.muscle.fasta.gblo.htm - as above in html format; PGL1_unique_Yersinia-x.muscle.tree - treefile from muscle alignment; PGL1_unique_Yersinia-x.sif - matches between proteins in simple interaction format for display on graphing software. [file gb-2010-11-1-r1-S16.zip › clusters2/PGL1_unique_yersinia-CL1273/PGL1_unique_yersinia-CL1273.muscle.fasta.gblo.htm]

PGL1\_unique\_yersinia-CL1273.muscle.fasta


## Gblocks 0.91b Results

Processed file: **PGL1\_unique\_yersinia-CL1273.muscle.fasta**  
Number of sequences: **11**  
Alignment assumed to be: **Protein**  
New number of positions: **285** (selected positions are underlined in blue)

```
                         10        20        30        40        50        60
                 =========+=========+=========+=========+=========+=========+
ykris0001_26620  -------MIKRATVSDVAALAKVSSATVS------NVLGNRPGKVSAKTRQRVLDAIT-A
yruck0001_34740  ------------------------------------------------------------
ypseu0001X_2270  -------MYRRLLLAAAVTAAMCSAVQAAPLVVGFSQIGSESGWRSAETKVAKQEAEKRG
ypest0001X_4215  -------MYRRLLLAAAVTAAMCSAVQAAPLVVGFSQIGSESGWRSAETKVAKQEAEKRG
yaldo0001_33600  MASEEINMYRRLLLAAAVTAAMCSAVQAAPLVVGFSQIGSESGWRSAETKVSKQEAEKRG
yrohd0001_30640  --------------------------------VGFSQIGSESGWRSAETKVAKQEAEKRG
yfred0001_30740  -------MYRRLLLAAAVTAAMCSAVQAAPLVVGFSQIGSESGWRSAETKVSKQEAEKRG
ymoll0001_33080  ---------------------MCSAVQAAPLVVGFSQIGSESGWRSAETKVSKQEAEKRG
yberc0001_32530  --------------------------------VGFSQIGSESGWRSAETKVSKQEAEKRG
yinte0001_35510  ---------------------MCSAAQAAPLVVGFSQIGSESGWRSAETKVSKQEAEKRG
yente0001X_4178  --------------------------------VGFSQIGSESGWRSAETKVSKQEAEKRG
                                                 ############################


                         70        80        90       100       110       120
                 =========+=========+=========+=========+=========+=========+
ykris0001_26620  LNYTYNENAATLRSNRSNIVGLVIHDLSNPYYTELIAKINRKLTDHGFATILACSDENLD
yruck0001_34740  ------------------------------------------------------------
ypseu0001X_2270  ITLKIADAQQKQENQIKAVRSFIAQGVDAIFIAPVVA--------TGWTPVLQ------E
ypest0001X_4215  ITLKIADAQQKQENQIKAVRSFIAQGVDAIFIAPVVA--------TGWTPVLQ------E
yaldo0001_33600  ITLKIADAQQKQENQIKAVRSFIAQGVDAIFIAPVVA--------TGWTPVLQ------E
yrohd0001_30640  ITLKIADAQQKQENQIKAVRSFIAQGVDAIFIAPVVA--------TGWTPVLQ------E
yfred0001_30740  ITLKIADAQQKQENQIKAVRSFIAQGVDAIFIAPVVA--------TGWTPVLQ------E
ymoll0001_33080  ITLKIADAQQKQENQIKAVRSFIAQGVDAIFIAPVVA--------TGWTPVLQ------E
yberc0001_32530  ITLKIADAQQKQENQIKAVRSFIAQGVDAIFIAPVVA--------TGWTPVLQ------E
yinte0001_35510  ITLKIADAQQKQENQIKAVRSFIAQGVDAIFIAPVVA--------TGWTPVLQ------E
yente0001X_4178  ITLKIADAQQKQENQIKAVRSFIAQGVDAIFIAPVVA--------TGWTPVLQ------E
                 #####################################                      #


                        130       140       150       160       170       180
                 =========+=========+=========+=========+=========+=========+
ykris0001_26620  SQQSYLQLMQRHNALAILLCPTWDTTSEHLQAWNALSPTITFLRPVASADIDFIGINNYQ
yruck0001_34740  ------------------------------------------------------------
ypseu0001X_2270  AKEAKIPVFLLDRMIEVNDPSLYTAAVASDSVYEGKVAGEWLLQDVVGKPCNVVELQ---
ypest0001X_4215  AKEAKIPVFLLDRMIEVNDPSLYTAAVASDSVYEGKVAGEWLLQDVAGKPCNVVELQ---
yaldo0001_33600  AKEAKIPVFLLDRMIEVNDPSLYTAAVASDSVYEGKVAGEWLLKDVAGKPCNVVELQ---
yrohd0001_30640  AKEAKIPVFLLDRMIEVNDPSLYTAAVASDSVYEGKVAGEWLLKDVAGKPCNVVELQ---
yfred0001_30740  AKEAKIPVFLLDRMIEVNDPSLYTAAVASDSVYEGKVAGEWLLKEVAGKPCNVVELQ---
ymoll0001_33080  AKEAKIPVFLLDRMIEVNDPSLYTAAVASDSVYEGKVAGEWLLKDVAGKPCNVVELQ---
yberc0001_32530  AKEAKIPVFLLDRMIEVNDPSLYTAAVASDSVYEGKVAGEWLLKDVAGKPCNVVELQ---
yinte0001_35510  AKEAKIPVFLLDRMIEVNDPSLYTAAVASDSVYEGKVAGEWLLKDVAGKPCNVVELQ---
yente0001X_4178  AKEAKIPVFLLDRMIEVNDPSLYTAAVASDSVYEGKVAGEWLLKDVAGKPCNVVELQ---
                 #########################################################   


                        190       200       210       220       230       240
                 =========+=========+=========+=========+=========+=========+
ykris0001_26620  AAYDITRRLISDGHKHLGFIGGSLESRLRRQRIQGWKDAHDDAGLHYAEE-HILDCAS--
yruck0001_34740  ------------------------------------------------------------
ypseu0001X_2270  ---------------------GTVGSSVAINRKKGFAD-----GIASAPGVKIIRSQSGD
ypest0001X_4215  ---------------------GTVGSSVAINRKKGFAD-----GIASAPGVKIIRSQSGD
yaldo0001_33600  ---------------------GTVGSSVAINRKKGFAD-----GIAFAPNVKIIRSQSGD
yrohd0001_30640  ---------------------GTVGSSVAINRKKGFAD-----GIASAPNVKIIRSQSGD
yfred0001_30740  ---------------------GTVGSSVAINRKKGFAD-----GIASAPNVKIIRSQSGD
ymoll0001_33080  ---------------------GTVGSSVAINRKKGFAD-----GIASAPNVKIIRSQSGD
yberc0001_32530  ---------------------GTVGSSVAINRKKGFAD-----GIASAPNVKIIRSQSGD
yinte0001_35510  ---------------------GTVGSSVAINRKKGFAD-----GIASAPNVKIIRSQSGD
yente0001X_4178  ---------------------GTVGSSVAINRKKGFAD-----GIASAPNVKIIRSQSGD
                                      #################     #################


                        250       260       270       280       290       300
                 =========+=========+=========+=========+=========+=========+
ykris0001_26620  -SMSAGAKATAKILDI---APQITALVCYQDVVAFGAINAIHYMGRKPGRDIAVTGFDGL
yruck0001_34740  ------------------------------------------------------------
ypseu0001X_2270  FTRSKGKEVMESFIKAEQNGKNICAVYAHNDDMAIGAIQAIKEAGLKPGSDIKIVSIDGV
ypest0001X_4215  FTRSKGKEVMESFIKAEQNGKNICAVYAHNDDMAIGAIQAIKEAGLKPGSDIKIVSIDGV
yaldo0001_33600  FTRSKGKEVMESFIKAEQNGKNICAVYAHNDDMAIGAIQAIKEAGLKSGSEIKIVSIDGV
yrohd0001_30640  FTRSKGKEVMESFIKAEQNGKNICAVYAHNDDMAIGAIQAIKEAGLKPGSEIKIVSIDGV
yfred0001_30740  FTRSKGKEVMESFIKAEQNGKNICAVYAHNDDMAIGAIQAIKEAGLKPGSEIKIVSIDGV
ymoll0001_33080  FTRSKGKEVMESFIKAEQNGKNICAVYAHNDDMAIGAIQAIKEAGLKPGTEIKIVSIDGV
yberc0001_32530  FTRSKGKEVMESFIKAEQNGKNICAVYAHNDDMAIGAIQAIKEAGLKPGTEIKIVSIDGV
yinte0001_35510  FTRSKGKEVMESFIKAEQNGKNICAVYAHNDDMAIGAIQAIKEAGLKPGSEIKIVSIDGV
yente0001X_4178  FTRSKGKEVMESFIKAEQNGKNICAVYAHNDDMAIGAIQAIKEAGLKPGSEIKIVSIDGV
                 ############################################################


                        310       320       330       340       350       360
                 =========+=========+=========+=========+=========+=========+
ykris0001_26620  ADAEGYLPSLTTAEVQIDV-----LTEHIVARLLSRLNDRDQSSMATLLHAHIHWRNSTR
yruck0001_34740  ------------------------MAVPTVDGLIMRKKGGIQPSKFIQIKFRLLQSGKSV
ypseu0001X_2270  PDI---FKAMSSGEANATVELTPNMAGPALDALIVLKKDGTQPPKFIQTESRLLQPDTAK
ypest0001X_4215  PDI---FKAMSSGEANATVELTPNMAGPALDALIVLKKDGTQPPKFIQTESRLLQPDTAK
yaldo0001_33600  PDI---FKAMSSGEANATVELTPNMAGPAFDALIALKKDGTQPPKFIQTESRLLQPDTAK
yrohd0001_30640  PDI---FKAMSSGEANATVELTPNMAGPAFDALIALKKDGTQPPKFIQTESRLLQPDTAK
yfred0001_30740  PDI---FKAMSSGEANATVELTPNMAGPAFDALIALKKDGTQPPKFIQTESRLLQPDTAK
ymoll0001_33080  PDI---FKAMSSGEANATVELTPNMAGPAFDALIALKKDGTQPPKFIQTESRLLQPDTAK
yberc0001_32530  PDI---FKAMSSGEANATVELTPNMAGPAFDALIALKKDGTQPPKFIQTESRLLQPDTAK
yinte0001_35510  PDI---FKAMSSGEANATVELTPNMAGPAFDALIALKKDGTQPPKFIQTESRLLQPDTAK
yente0001X_4178  PDI---FKAMSSGEANATVELTPNMAGPAFDALIALKKDGTQPPKFIQTESRLLQPDTAK
                 ###   ######################################################


                        370
                 =========+=
ykris0001_26620  AH---------
yruck0001_34740  K----------
ypseu0001X_2270  QEYELKKSLGY
ypest0001X_4215  QEYELKKSLGY
yaldo0001_33600  QEYESKKSLGY
yrohd0001_30640  QEYESKKSLGY
yfred0001_30740  QEYESKKSLGY
ymoll0001_33080  QEYESKKSLGY
yberc0001_32530  QEYESKKSLGY
yinte0001_35510  QEYESKKSLGY
yente0001X_4178  QEYESKKSLGY
                 ###########
```

```
Parameters used
Minimum Number Of Sequences For A Conserved Position: 6
Minimum Number Of Sequences For A Flanking Position: 9
Maximum Number Of Contiguous Nonconserved Positions: 8
Minimum Length Of A Block: 10
Allowed Gap Positions: With Half
Use Similarity Matrices: Yes
```

```
Flank positions of the 5 selected block(s)
Flanks: [33  97]  [120  177]  [202  218]  [224  303]  [307  371]  

New number of positions in PGL1_unique_yersinia-CLUSTERS.dir/PGL1_unique_yersinia-CL1273/PGL1_unique_yersinia-CL1273.muscle.fasta.gblo:  285  (76% of the original 371 positions)
```
