# Supplementary material for: Genomic characterization of the Yersinia genus
Source: Genome Biol. 2010 Jan 4;11(1):R1. doi: 10.1186/gb-2010-11-1-r1 (PMC2847712; doi:10.1186/gb-2010-11-1-r1)
Supplement: Additional file 16 — The top level directory consists of a directory called Additional_cluster_files and 5010 directories, one for each multi-protein cluster family. (This top level directory has been split into three data files for uploading purposes (Additional files 15, 16, 17.) Within the directory are the following files: PGL1_unique_Yersinia_unclustered.out - list of all protein singletons that MCL did not group into a cluster (see Materials and Methods); PGL1_Yersinia_unique_locus_tags.txt - names of the 11 locus tag prefixes used for each genome; PGL1_unique_Yersinia.gff - mapping each Yersinia protein to a cluster in tab delimited GFF; PGL1_unique_Yersinia.sigfile - list of the longest protein in each cluster; PGL1_unique_Yersinia.summary - summary table of features of each of the clusters; PGL1_unique_Yersinia.table - summary table of each protein in the clusters. Within each cluster directory are the following files, where 'x' is the cluster name: PGL1_unique_Yersinia-x.faa - multifasta file of the proteins in the cluster; PGL1_unique_Yersinia-x.summary - summary of the properties of the proteins; PGL1_unique_Yersinia-x.matches - blast matches between the proteins of the cluster; PGL1_unique_Yersinia-x.muscle.fasta - muscle alignment of the proteins; PGL1_unique_Yersinia-x.muscle.fasta.gblo - gblocks output of muscle alignment (that is, auto-trimmed alignment); PGL1_unique_Yersinia-x.muscle.fasta.gblo.htm - as above in html format; PGL1_unique_Yersinia-x.muscle.tree - treefile from muscle alignment; PGL1_unique_Yersinia-x.sif - matches between proteins in simple interaction format for display on graphing software. [file gb-2010-11-1-r1-S16.zip › clusters2/PGL1_unique_yersinia-CL1274/PGL1_unique_yersinia-CL1274.muscle.fasta.gblo.htm]

PGL1\_unique\_yersinia-CL1274.muscle.fasta


## Gblocks 0.91b Results

Processed file: **PGL1\_unique\_yersinia-CL1274.muscle.fasta**  
Number of sequences: **11**  
Alignment assumed to be: **Protein**  
New number of positions: **294** (selected positions are underlined in blue)

```
                         10        20        30        40        50        60
                 =========+=========+=========+=========+=========+=========+
yruck0001_5460   ---------------MPAISLRQIEIFHAVMTTGNLTEAAVLLQTSQPTVSRELARFEQL
ypseu0001X_3404  ---------------MPAISLRQIEIFHGVMTTGNLTEAALLLQTSQPTVSRELARFEQL
ypest0001X_9990  ---------------MPAISLRQIEIFHGVMTTGNLTEAALLLQTSQPTVSRELARFEQL
yaldo0001_7300   MIWVIFFSRPRWSVVMAAISLRQIEIFHGVMTTGNLTEAALLLQTSQPTVSRELARFEQL
yrohd0001_8330   ---------------MPAISLRQIEIFHAVMTTGNLTEAAQLLQTSQPTVSRELARFEKL
yfred0001_43510  ------------------------------MTTGNLTEAALLLQTSQPTVSRELARFEQL
ykris0001_6030   --------------VMPAISLRQIEIFHAVMTTGNLTEAALLLQTSQPTVSRELARFEQL
yente0001X_9210  ------------------------------MTTGNLTEAALLLQTSQPTVSRELARFEQL
yinte0001_8320   ------------------------------MTTGNLTEAALLLQTSQPTVSRELARFEQL
yberc0001_7070   --------------VMPAISLRQIEIFHGVMTTGNLTEAALLLQTSQPTVSRELARFEQL
ymoll0001_7630   ---------------MPAISLRQIEIFHGVMTTGNLTEAASLLQTSQPTVSRELARFEQL
                                               ##############################


                         70        80        90       100       110       120
                 =========+=========+=========+=========+=========+=========+
yruck0001_5460   IQLNLFDRLRGRLYPTAQGLRLFEEVQRSYYGLDRIVQAAESIRQFQQAQLSIACLPVFS
ypseu0001X_3404  VQLTLFERVRGRLYPTVQGLRLFEEVQRSYYGLDRIKQAAEGIRQFQHAQLSIACLPVFS
ypest0001X_9990  VQLTLFERVRGRLYPTVQGLRLFEEVQRSYYGLDRIKQAAEGIRQFQHAQLSIACLPVFS
yaldo0001_7300   VQLRLFDRVRGRLYPTVQGLRLFEEVQRSYYGLDRIKQAAQGIRQFQHAQLSIACLPVFS
yrohd0001_8330   VQLTLFDRLRGRLYPTVQGLRLFEEVQRSYYGLDRIKQAAEGIRQFRQAQLSIACLPVFS
yfred0001_43510  VQLKLFDRVRGRLYPTVQGLRLFEEVQRSYYGLDRIKQAAEGIRQFQHAQLSIACLPVFS
ykris0001_6030   VQLKLFDRVRGRLYPTVQGLRLFEEVQRSYYGLDRIKQAAEGIRQFQHAQLSIACLPVFS
yente0001X_9210  VQLKLFDRVRGRLYPTVQGLRLFEEVQRSYYGLDRIKQAAEGIRQFQHAQLSIACLPVFS
yinte0001_8320   VQLTLFDRVRGRLYPTVQGLRLFEEVQRSYYGLDRIRQAAEGIRQFQQAQLSIACLPVFS
yberc0001_7070   VQLKLFDRVRGRLYPTVQGLRLFEEVQRSYYGLDRIRQAAEGIRQFQQAQLSIACLPVFS
ymoll0001_7630   VQLKLFDRVRGRLYPTVQGLRLFEEVQRSYYGLDRIRQAAEGIRQFRQAQLSIACLPVFS
                 ############################################################


                        130       140       150       160       170       180
                 =========+=========+=========+=========+=========+=========+
yruck0001_5460   QSLLPAVCKTFIDSYPEVSLNVIPQESPLLEEWLSAQHHDLGITENLQTPAGTERQTLMT
ypseu0001X_3404  QSLLPAVCKPFIDRYPEVSLNVIPQESPLLEEWLSAQRHDLGFTENTQTPAGTQRHTLMT
ypest0001X_9990  QSLLPAVCKPFIDRYPEVSLNVIPQESPLLEEWFSAQRHDLGLTENIQTPAGTQRHTLMT
yaldo0001_7300   QSLLPAVCKPFMDRYPEVSLNVIPQESPLLEEWLSAQRHDLGLTENAQTPAGTVRHPLMT
yrohd0001_8330   QSLLPGVCKPFIDLYPEVSLSVIPQESPLLEEWLSAQRHDLGLTENTQTPAGTVRHTLMA
yfred0001_43510  QSLLPGVCRPFIDRYPEVSLSVIPQESPLLEEWLSAQRHDLGLTENTHTPAGTVRHTLMT
ykris0001_6030   QSLLPAVCKPFIDRYPEVSLSVIPQESPLLEEWLSAQRHDLGLTENTQTPAGTVRYALMT
yente0001X_9210  QSLLPAVCKPFIDRYPEVSLSVIPQESPLLEEWLSAQRHDLGLTENTQTPAGTVRHALMT
yinte0001_8320   QSLLPAVCKPFIDRYPEVSLNIIPQESPLLEEWLSAQRHDLGLTENTQTPAGTLRHPLMT
yberc0001_7070   QSLLPAVCKPFIESYPEVSLSVIPQESPLLEEWLSAQRYDLGLTENTQTPAGTLRHALMT
ymoll0001_7630   QSLLPAVCKPFIASYPEVSLSVIPQESPLLEEWLSAQRHDLGLTENTQTPAGTLRHALMT
                 ############################################################


                        190       200       210       220       230       240
                 =========+=========+=========+=========+=========+=========+
yruck0001_5460   LNEVCVLPAEHPLTKKMVLTPQDFSGENFISLSVTDSYRQLLDNLFNEKGINRRLVMETH
ypseu0001X_3404  VNEVCVLPADHPLREKSVLTPQDFNGENFVSLSVTDSYRQLLDNLFAEEKVTRRLVMETH
ypest0001X_9990  VNEVCVLPADHPLREKSVLTPQDFNGENFVSLSVTDSYRQLLDNLFAEEKVTRRLVMETH
yaldo0001_7300   LNEVCVLPHDHPLQSKSVLTPQDFAGENFISLSVTDSYRQLLDSLFTDEKINRRLVMETH
yrohd0001_8330   VNEVCVLPRHHPLQHKAVLTPQDFQGENFISLSVTDSYRQLLDNLFAEQGINRRLVLETH
yfred0001_43510  VNEVCVLPNDHPLQHKTVLTPQDFQGENFISLSVTDSYRQLLDNLFTEQGINRRLVLETH
ykris0001_6030   VNEVCVLPSDHPLREKTVLTPHDFQGENFISLSVTDSYRQLLDNLFTEQGINRRLVLETH
yente0001X_9210  VNEVCVLPCGHPLQEKAVLTPQDFQGENFISLSVTDSYRQLLDNLFTEQGINRRLVLETH
yinte0001_8320   VNEICVLPSDHPLRDKSVLTPQDFQGENFISLSVTDSYRQLLDKLFIEQGISRRLVLETH
yberc0001_7070   VNEVCVLPGDHPLREKSVLTPQDFHGENFISLSVTDSYRQLLDNLFTEQGISRRLVLETH
ymoll0001_7630   VNEVCVLPSDHPLREKSVLTPQDFQGENFISLSVTDSYRQLLDNLFAEQGIHRRLVLETH
                 ############################################################


                        250       260       270       280       290       300
                 =========+=========+=========+=========+=========+=========+
yruck0001_5460   SAASVCAMVREGVGVSIVNPLTALDYAENNHEKGICVRRFSINVPFTVSLIQPLHRPSSM
ypseu0001X_3404  SAASVCAMVREGVGVSIVNPLTALDYLSKGRGSGVCVRPFSVDIPFTVSLIQPLHRPSST
ypest0001X_9990  SAASVCAMVREGVGVSIVNPLTALDYLSKGRGSGVCVRPFSVDIPFTVSLIQPLHRPSST
yaldo0001_7300   SAASICAMVREGVGVSIVNPLTALDYISKGAGEGVCVRPFSIEIPFTISLIQPIHRPSSA
yrohd0001_8330   SAASVCAMVQEGVGVSIVNPLTALDYLSKAGAEGVCVRPFSVEIPFTVSLIQPIHRPSSS
yfred0001_43510  SAASVCAMVREGVGVSIVNPLTALDYISKGAGEGVCVRPFSVEIPFTISLIKPIHRPSST
ykris0001_6030   SAASVCAMVREGVGVSIVNPLTALDYIGKGAGDGVCVRRFSVEIPFTISLIQPIHRPSSS
yente0001X_9210  SAASVCAMVREGVGVSIVNPLTALDYIGAGVNEGVCIRPFSVDIPFTISLIQPIHRPSSS
yinte0001_8320   SAASVCAMVREGVGVSIVNPLTALDYISKGSAEGVCVRPFSVDIPFTISLIQPKHRPSST
yberc0001_7070   SAASVCAMVREGVGVSIVNPLTALDYISKGMEEGVCVRPFSVEIPFTISLIQPIHRPSST
ymoll0001_7630   SAASVCAMVREGVGVSIVNPLTALEYFSKGGDEGVCVRPFSVEIPFTISLIQPIHRPSST
                 ############################################################


                        310       320       330
                 =========+=========+=========+==
yruck0001_5460   LVDTFISHLKQQVTTLQTRLTAIITG------
ypseu0001X_3404  LVDTFVGHLKQQTTLFQQRLATVIAPAC----
ypest0001X_9990  LVDTFVGHLKQQTTLFQQRLATVIAPAC----
yaldo0001_7300   LVDTFIEHLKQQAVTFQNRLAAVIAH------
yrohd0001_8330   LVDTFIEHLKQQAITFQQRLAAVIAPQ-----
yfred0001_43510  LVDTFIEHLKQQAITFQQRLTAVIAPQY----
ykris0001_6030   LVDTFIEHLKQQAITFQQRLAAVIAQQY----
yente0001X_9210  LVDTFIEHLKQQAITFQQRLAAVIAQHNSSLN
yinte0001_8320   LVDTFIEHLKQQAITFQQRLTQVITQQY----
yberc0001_7070   LVDTFIEHLKQQAITFQQRLAKVITQQ-----
ymoll0001_7630   LVETFIEHLKQQAITFQQRLATVIAQ------
                 ########################
```

```
Parameters used
Minimum Number Of Sequences For A Conserved Position: 6
Minimum Number Of Sequences For A Flanking Position: 9
Maximum Number Of Contiguous Nonconserved Positions: 8
Minimum Length Of A Block: 10
Allowed Gap Positions: With Half
Use Similarity Matrices: Yes
```

```
Flank positions of the 1 selected block(s)
Flanks: [31  324]  

New number of positions in PGL1_unique_yersinia-CLUSTERS.dir/PGL1_unique_yersinia-CL1274/PGL1_unique_yersinia-CL1274.muscle.fasta.gblo:  294  (88% of the original 332 positions)
```
