# Supplementary material for: Genomic characterization of the Yersinia genus
Source: Genome Biol. 2010 Jan 4;11(1):R1. doi: 10.1186/gb-2010-11-1-r1 (PMC2847712; doi:10.1186/gb-2010-11-1-r1)
Supplement: Additional file 17 — The top level directory consists of a directory called Additional_cluster_files and 5010 directories, one for each multi-protein cluster family. (This top level directory has been split into three data files for uploading purposes (Additional files 15, 16, 17.) Within the directory are the following files: PGL1_unique_Yersinia_unclustered.out - list of all protein singletons that MCL did not group into a cluster (see Materials and Methods); PGL1_Yersinia_unique_locus_tags.txt - names of the 11 locus tag prefixes used for each genome; PGL1_unique_Yersinia.gff - mapping each Yersinia protein to a cluster in tab delimited GFF; PGL1_unique_Yersinia.sigfile - list of the longest protein in each cluster; PGL1_unique_Yersinia.summary - summary table of features of each of the clusters; PGL1_unique_Yersinia.table - summary table of each protein in the clusters. Within each cluster directory are the following files, where 'x' is the cluster name: PGL1_unique_Yersinia-x.faa - multifasta file of the proteins in the cluster; PGL1_unique_Yersinia-x.summary - summary of the properties of the proteins; PGL1_unique_Yersinia-x.matches - blast matches between the proteins of the cluster; PGL1_unique_Yersinia-x.muscle.fasta - muscle alignment of the proteins; PGL1_unique_Yersinia-x.muscle.fasta.gblo - gblocks output of muscle alignment (that is, auto-trimmed alignment); PGL1_unique_Yersinia-x.muscle.fasta.gblo.htm - as above in html format; PGL1_unique_Yersinia-x.muscle.tree - treefile from muscle alignment; PGL1_unique_Yersinia-x.sif - matches between proteins in simple interaction format for display on graphing software. [file gb-2010-11-1-r1-S17.zip › clusters3/PGL1_unique_yersinia-CL3002/PGL1_unique_yersinia-CL3002.muscle.fasta.gblo.htm]

PGL1\_unique\_yersinia-CL3002.muscle.fasta


## Gblocks 0.91b Results

Processed file: **PGL1\_unique\_yersinia-CL3002.muscle.fasta**  
Number of sequences: **7**  
Alignment assumed to be: **Protein**  
New number of positions: **330** (selected positions are underlined in blue)

```
                         10        20        30        40        50        60
                 =========+=========+=========+=========+=========+=========+
yruck0001_33940  MNNILSLIGRNKTLFTQDLAKNKNELASIVSESRFLVLGGAGSIGQAVTKEIFKRNPK--
ypest0001X_2773  MNAMSLLSVEVSNFTFVGLFKNDNVNIVSIHRVLLLLEELMSQLPEKM-SAVVCHGPQDY
ypseu0001X_2858  MNAMSLLSVEVSNFTFVDLFKNDNVNIVSIHRVLLLLEELMSQLPEKM-SAVVCHGPQDY
ypseu0001X_9210  -----------------------------MSHENLKGAQMAEGIPETM-RAVVAYGPRDY
ypest0001X_3634  ------------------VCDKKPLKESSMSHENLKGAQMAEGIPETM-RAVVAYGPRDY
yberc0001_38820  -----------------------------MYNECSKDAQMLENIPKNM-RAVVAYGPGDY
ymoll0001_28010  -----------------------------MYNERSIDAQMVAGIPEKM-RAVVAYGPGDY
                                                                  ###########


                         70        80        90       100       110       120
                 =========+=========+=========+=========+=========+=========+
yruck0001_33940  KLHVIDISENNMVELVRDIRSQFGYILGDFQTFALDIGSLEYDAFIKADGQYDYVLNLSA
ypest0001X_2773  RLEHVPTPVPKAKELVIKVKG-CGICAGDCKCKN---GAKMF---WGET---PWV-----
ypseu0001X_2858  RLEHVPTPVPKAKELVIKVKG-CGICAGDCKCKN---GAKMF---WGET---PWV-----
ypseu0001X_9210  RFERVPVPTIDAKEILVKVEG-CGICAGDTKAFE---GAPSF---WGDDKQPAYI-----
ypest0001X_3634  RFERVPVPTIDAKEILVKVEG-CGICAGDTKAFE---GAPSF---WGDDKQPAYI-----
yberc0001_38820  RLEIVSVPTIDAKEILVKVEA-CGICAGDTKAFG---GAPSF---WGDKTQPAYI-----
ymoll0001_28010  RLQIVSVPTIDAKEILVKVEG-CGICAGDTKAFG---GAPSF---WGDDKQPAYI-----
                 ##################### ###########            ##########     


                        130       140       150       160       170       180
                 =========+=========+=========+=========+=========+=========+
yruck0001_33940  LKHVRSEKDPFTLMRMIEVNILNTNKTIEQSFETGVKKYFCVSTDKAANPVNMMGASKRI
ypest0001X_2773  -------KPPVVPGHEFYGHIVALGEGTESKYKLGER----VIAEQI---VPCWDCRYCN
ypseu0001X_2858  -------KPPVVPGHEFYGHIVALGEGTESKYKIGER----VIAEQI---VPCWDCRYCN
ypseu0001X_9210  -------KAPMIPGHEFIGHVVGLGADVE-GFELGDR----VTSEQI---VPCWGCRFCN
ypest0001X_3634  -------KAPMIPGHEFIGHVVGLGADVE-GFELGDR----VTSEQI---VPCWGCRFCN
yberc0001_38820  -------KAPMIPGHEFIGHVVGLGAEVE-GFELGDR----VTSEQI---VPCWECRFCN
ymoll0001_28010  -------KAPMIPGHEFIGHVVGLGAEVE-GFKLGDR----VTSEQI---VPCWECRFCN
                        ######################                     ##########


                        190       200       210       220       230       240
                 =========+=========+=========+=========+=========+=========+
yruck0001_33940  MEMFLMRNSEKI-----DISTARFAN-VAFSDGSLLHGFEQRLSKQQP-LVAPNDIKRYF
ypest0001X_2773  SGSYWMCETHNIYGFQKDVAEGGMAEYMRFSENAIVHKIPESLSHEDAVLIEPMACAIHT
ypseu0001X_2858  SGSYWMCETHNIYGFQKDVAEGGMAEYMRFSENAIVHKIPESLSHEDAVLIEPMACAIHT
ypseu0001X_9210  HGQYWMCEKHDLYGFQSNV-NGAMAEYLKFTKEAINYHVPDHLPLEKAILIEPYACAFHA
ypest0001X_3634  HGQYWMCEKHDLYGFQSNV-NGAMAEYLKFTKEAINYHVPDHLPLEKAILIEPYACAFHA
yberc0001_38820  RGQYWMCEKHDLYGFQHNV-NGAMAEYMKFTKEAINYRVPDDLPLEKAILIEPYACSFHA
ymoll0001_28010  RGQYWMCEKHDLYGFQHNV-NGAMAEYMKFTKEAINYHVPADLPLEKAILIEPYACSFHA
                 ################### ########################################


                        250       260       270       280       290       300
                 =========+=========+=========+=========+=========+=========+
yruck0001_33940  VTPQES--GELCLMS-------CIFGENRDIFFPKLSESLHLITFADIAER--YLRQRGY
ypest0001X_2773  VARGDIQLDDVVVLAGAGPLGLCMVPVAR------LKTPKKLIVIDTIEERLALAKAFGA
ypseu0001X_2858  VARGDIQLDDVVVLAGAGPLGLCMVQVAR------LKTPKKLIVIDTIEERLALAKAFGA
ypseu0001X_9210  VQRAKIKLGDVVVLAGSGTLGLGMIGAIK------KSGAAKLIVLDLSDDRLALAKKFGA
ypest0001X_3634  VQRAKIKLGDVVVLAGSGTLGLGMIGAIK------KSGAAKLIVLDLSDDRLALAKKFGA
yberc0001_38820  VQRANIKLGDVVVLAGAGTLGLGMVGAIK------KSGPGKLIVLDLSDDRLALAKKFGA
ymoll0001_28010  VQRANIKLGDVVVLAGAGTLGLGMVGAIK------KSGPGKLIVLDLSDDRLALAKKFGA
                 #############################      #########################


                        310       320       330       340       350       360
                 =========+=========+=========+=========+=========+=========+
yruck0001_33940  QPYLCQTEDEARELAKTLPAQGQWPCLFTSSDTTGEKDFEEFYTSKEILDMERFDNLGII
ypest0001X_2773  DVVINPLKEDADKIVKSL--TGGYGCDVYIEATGAPIGVTQGLQM--IRKLGRFVEFSVF
ypseu0001X_2858  DVVINPLKEDADKIVKSL--TGGYGCDVYIEATGAPIGVTQGLQM--IRKLGRFVEFSVF
ypseu0001X_9210  DLVLNPSRDDVNTLIKEM--TGGYGCDIYIDATGAQKAVEQGLTL--LRKLGTFVEFSVF
ypest0001X_3634  DLVLNPSRDDVNTLIKEM--TGGYGCDIYIDATGAQKAVEQGLTL--LRKLGTFVEFSVF
yberc0001_38820  DLVLNPSKDDVISIIKEM--TDGYGCDIYIEATGAQKAVEQGLTL--LRKLGTFVEFSVF
ymoll0001_28010  DLVLNPTKDDVISIIKEM--TDGYGCDIYIEATGAQKAVEQGLTL--LRKLGTFVEFSVF
                 ##################  #########################  #############


                        370       380       390       400       410       420
                 =========+=========+=========+=========+=========+=========+
yruck0001_33940  QNEPDYDPSRLEH---------------FEKEIMRMTTALEWTKNDIVKLF----FEMIP
ypest0001X_2773  GQETTVDWSIIGDRKELDIRGAHLAPYSYEIATDLFERGLVTSKGIVTHSYSLDDWEKAF
ypseu0001X_2858  GQETTVDWSIIGDRKELDIRGAHLAPYSYEIATDLFERGLVTSKGIVTHSYSLDDWEKAF
ypseu0001X_9210  KDPVTVDWSIISDRKELDVLGSHLGPYCFPLVIEGISNGDLPTEGVVTHILPLEKYAEGI
ypest0001X_3634  KDPVTVDWSIISDRKELDVLGSHLGPYCFPLVIEGISNGDLPTEGVVTHILPLEKYAEGI
yberc0001_38820  KEPVTVDWSIISDRKELDVLGSHLGPYCYPLVIKGICNGDLPTDGVVTHILPLERFAEGF
ymoll0001_28010  KDPVTVDWSIISDRKELDVLGSHLGPYCYPLVIEGICNGDLPTEGVVTHILPLEKFAEGF
                 ############################################################


                        430
                 =========+=========
yruck0001_33940  DFCHKETGKY---LDSKM-
ypest0001X_2773  ELAD-STNSIKVILVP---
ypseu0001X_2858  ELAD-STNSIKVILVP---
ypseu0001X_9210  ELVKRGVDSLKVVLNPNLT
ypest0001X_3634  ELVKRGVDSLKVVLNPNLT
yberc0001_38820  EMVTKGIGSLKVVLNPNIQ
ymoll0001_28010  EMVTKGVGSLKVVLNPNIQ
                 ################
```

```
Parameters used
Minimum Number Of Sequences For A Conserved Position: 4
Minimum Number Of Sequences For A Flanking Position: 5
Maximum Number Of Contiguous Nonconserved Positions: 8
Minimum Length Of A Block: 10
Allowed Gap Positions: With Half
Use Similarity Matrices: Yes
```

```
Flank positions of the 9 selected block(s)
Flanks: [50  81]  [83  93]  [106  115]  [128  149]  [171  199]  [201  269]  [276  318]  [321  345]  [348  436]  

New number of positions in PGL1_unique_yersinia-CLUSTERS.dir/PGL1_unique_yersinia-CL3002/PGL1_unique_yersinia-CL3002.muscle.fasta.gblo:  330  (75% of the original 439 positions)
```
