# Supplementary material for: Genomic characterization of the Yersinia genus
Source: Genome Biol. 2010 Jan 4;11(1):R1. doi: 10.1186/gb-2010-11-1-r1 (PMC2847712; doi:10.1186/gb-2010-11-1-r1)
Supplement: Additional file 17 — The top level directory consists of a directory called Additional_cluster_files and 5010 directories, one for each multi-protein cluster family. (This top level directory has been split into three data files for uploading purposes (Additional files 15, 16, 17.) Within the directory are the following files: PGL1_unique_Yersinia_unclustered.out - list of all protein singletons that MCL did not group into a cluster (see Materials and Methods); PGL1_Yersinia_unique_locus_tags.txt - names of the 11 locus tag prefixes used for each genome; PGL1_unique_Yersinia.gff - mapping each Yersinia protein to a cluster in tab delimited GFF; PGL1_unique_Yersinia.sigfile - list of the longest protein in each cluster; PGL1_unique_Yersinia.summary - summary table of features of each of the clusters; PGL1_unique_Yersinia.table - summary table of each protein in the clusters. Within each cluster directory are the following files, where 'x' is the cluster name: PGL1_unique_Yersinia-x.faa - multifasta file of the proteins in the cluster; PGL1_unique_Yersinia-x.summary - summary of the properties of the proteins; PGL1_unique_Yersinia-x.matches - blast matches between the proteins of the cluster; PGL1_unique_Yersinia-x.muscle.fasta - muscle alignment of the proteins; PGL1_unique_Yersinia-x.muscle.fasta.gblo - gblocks output of muscle alignment (that is, auto-trimmed alignment); PGL1_unique_Yersinia-x.muscle.fasta.gblo.htm - as above in html format; PGL1_unique_Yersinia-x.muscle.tree - treefile from muscle alignment; PGL1_unique_Yersinia-x.sif - matches between proteins in simple interaction format for display on graphing software. [file gb-2010-11-1-r1-S17.zip › clusters3/PGL1_unique_yersinia-CL3003/PGL1_unique_yersinia-CL3003.muscle.fasta.gblo.htm]

PGL1\_unique\_yersinia-CL3003.muscle.fasta


## Gblocks 0.91b Results

Processed file: **PGL1\_unique\_yersinia-CL3003.muscle.fasta**  
Number of sequences: **7**  
Alignment assumed to be: **Protein**  
New number of positions: **184** (selected positions are underlined in blue)

```
                         10        20        30        40        50        60
                 =========+=========+=========+=========+=========+=========+
ypseu0001X_4287  ----------------MLKPYPFLKQDTYAWCLSIGLPVIWIPFAIFFPTEIALGLYMVL
yfred0001_40500  ----------------MLKPYPFLKQDTYAWCLSIGLPVIWGLSAIFLPQKVALGLYMLC
yruck0001_33620  ----------------MLKPYPLSKQDGYAWALAIVPPLLWAPLSIFFSKEIALGFYLVL
yberc0001_40440  ----------------MLKPYPLSKQDSYAWALAIVPPLLWAPFAIFFPKEIALGFYLVL
yinte0001_41680  ----------------MLKPYPLSKQDSYAWALTIVLPLLWAPFAIFFPKEIALGFYLVL
yruck0001_32380  ----------------MLKPYPFLKQDTYAWCLSIGLPILWVPFAIFCPKEIALGLYMVF
yrohd0001_22360  MLFLSSGFCAAGREAIMLKPYPLSKQDGYAWALAIILPILWVPFVIFFPKEIALGLYMML
                                 ############################################


                         70        80        90       100       110       120
                 =========+=========+=========+=========+=========+=========+
ypseu0001X_4287  SLIWVLLDRLNLMKQEITPPSMGWFLLPIVYLRQRDERQGKLWRLLQVWLICTVLSAVAG
yfred0001_40500  SLVWVLLDRLNLMKQEITAPSLMWFLLPMVYLRQRDERQGKPWRLLQVWLICTVLSAVAG
yruck0001_33620  SLVWTLLDRLSLMKQEKVPPSFWYFLLPIVYLRQRDERQGKPWRLLQVWLMCTVLSAIAG
yberc0001_40440  SLVWTLLDRLSLMKQEKVPPSFWYFLLPMVYLRQRDERQGKPWRLLQVWLICTVLSVIAG
yinte0001_41680  SLVWTLLDRLRLMKQEKVPPSFWYFLLPMVYLRQRDERQGKPWRLLQVWLICTVLSVIAG
yruck0001_32380  SLIWVLLDRLSLMKQEKLAPSFLYFLLPMVYLRQRDERQGKPWRLLQVWLICTVLSAVAG
yrohd0001_22360  SLIWVLLDRFRLMKQDKFSPSFLYFLLPMVYLRQRDERQGKPWRLLQVWLLCTVLSVIAG
                 ############################################################


                        130       140       150       160       170       180
                 =========+=========+=========+=========+=========+=========+
ypseu0001X_4287  NHFKTQSGTERLAQSACPVVTKILQRQGIEEHCIRITDIKEEVAGRFYQAQALLNTGSKE
yfred0001_40500  NHFKTQSGTEQLAQSACPVVTKILQRQGIEEHCIRITGIREEVAERFYQAQALLNTGSKE
yruck0001_33620  EQFKKQSRTERLARSACSTVTQLMKQEGIDERCIRVTDLKEEVSGRFYQAQALMNTGNKE
yberc0001_40440  EQFKKQSGTEQLAQSACSIVTQLMKQEGIDERCIRVTDLKEEVSGRFYRAQALMSTGNKE
yinte0001_41680  EQLKKQSGTEQLAQSACSIVTQLMKQEGIDERCIRVTDLKEEVSGRFYRAQALMNTGNKE
yruck0001_32380  NHFKKQSGTEQLAKSACSTVSQIMQREGIDERCIRVTDLKEEVSGRFYRAQALMNTGNKE
yrohd0001_22360  EQFKKQSGTEQLAQSACSTVTQIMQREGIDERCIRVTDLKEEVSGRFYRALALMNTGNKE
                 ############################################################


                        190       200
                 =========+=========+=
ypseu0001X_4287  PLTIEVRSGGNIYVTLTDSE-
yfred0001_40500  PLTIEVRSGGNIYVTLTDLE-
yruck0001_33620  PMTIEVR-GRDIYVVLPEWEE
yberc0001_40440  PMTIEVR-GRDIYVVLPEWEE
yinte0001_41680  PMTIEVR-GRDIYVVLPEWEE
yruck0001_32380  PMTIEVR-GRDIYVVLPELGE
yrohd0001_22360  PLTIEVR-GRDIYVVLPEVGE
                 ####### #############
```

```
Parameters used
Minimum Number Of Sequences For A Conserved Position: 4
Minimum Number Of Sequences For A Flanking Position: 5
Maximum Number Of Contiguous Nonconserved Positions: 8
Minimum Length Of A Block: 10
Allowed Gap Positions: With Half
Use Similarity Matrices: Yes
```

```
Flank positions of the 2 selected block(s)
Flanks: [17  187]  [189  201]  

New number of positions in PGL1_unique_yersinia-CLUSTERS.dir/PGL1_unique_yersinia-CL3003/PGL1_unique_yersinia-CL3003.muscle.fasta.gblo:  184  (91% of the original 201 positions)
```
