# Supplementary material for: Genomic characterization of the Yersinia genus
Source: Genome Biol. 2010 Jan 4;11(1):R1. doi: 10.1186/gb-2010-11-1-r1 (PMC2847712; doi:10.1186/gb-2010-11-1-r1)
Supplement: Additional file 17 — The top level directory consists of a directory called Additional_cluster_files and 5010 directories, one for each multi-protein cluster family. (This top level directory has been split into three data files for uploading purposes (Additional files 15, 16, 17.) Within the directory are the following files: PGL1_unique_Yersinia_unclustered.out - list of all protein singletons that MCL did not group into a cluster (see Materials and Methods); PGL1_Yersinia_unique_locus_tags.txt - names of the 11 locus tag prefixes used for each genome; PGL1_unique_Yersinia.gff - mapping each Yersinia protein to a cluster in tab delimited GFF; PGL1_unique_Yersinia.sigfile - list of the longest protein in each cluster; PGL1_unique_Yersinia.summary - summary table of features of each of the clusters; PGL1_unique_Yersinia.table - summary table of each protein in the clusters. Within each cluster directory are the following files, where 'x' is the cluster name: PGL1_unique_Yersinia-x.faa - multifasta file of the proteins in the cluster; PGL1_unique_Yersinia-x.summary - summary of the properties of the proteins; PGL1_unique_Yersinia-x.matches - blast matches between the proteins of the cluster; PGL1_unique_Yersinia-x.muscle.fasta - muscle alignment of the proteins; PGL1_unique_Yersinia-x.muscle.fasta.gblo - gblocks output of muscle alignment (that is, auto-trimmed alignment); PGL1_unique_Yersinia-x.muscle.fasta.gblo.htm - as above in html format; PGL1_unique_Yersinia-x.muscle.tree - treefile from muscle alignment; PGL1_unique_Yersinia-x.sif - matches between proteins in simple interaction format for display on graphing software. [file gb-2010-11-1-r1-S17.zip › clusters3/PGL1_unique_yersinia-CL3004/PGL1_unique_yersinia-CL3004.muscle.fasta.gblo.htm]

PGL1\_unique\_yersinia-CL3004.muscle.fasta


## Gblocks 0.91b Results

Processed file: **PGL1\_unique\_yersinia-CL3004.muscle.fasta**  
Number of sequences: **7**  
Alignment assumed to be: **Protein**  
New number of positions: **293** (selected positions are underlined in blue)

```
                         10        20        30        40        50        60
                 =========+=========+=========+=========+=========+=========+
yruck0001_4250   -----------------------------------------------------MATYTGY
yrohd0001_35770  ------------------------------------------------------------
yinte0001_9570   -------MPTAGTKRNNHATSTAHYLSDQQRARIKQLSRSWLWRLELPTWVLIGVIYTGW
ymoll0001_39610  --------------------------------------------LELPTWILIGVIYTGW
yfred0001_8680   MPRMSLNMPTADSKISHYVAGASYYLNEQQRARVKQLSRSWMWRLELPTWALIGVIYTGW
yente0001X_1411  -------MPAADTKFNHRGAGAAYYLSEQQRARIKLLSRTWVWRLELPTWILIGVIYTGW
ykris0001_9650   -------MSSTDTKLNHREAGAAYYLSERQRARIKLLSRSWVWRLELPTWILITIIYTGW
                                                             ################


                         70        80        90       100       110       120
                 =========+=========+=========+=========+=========+=========+
yruck0001_4250   MI------------------------------------------------------FPHC
yrohd0001_35770  -----------------------------------------------LGMAALA------
yinte0001_9570   FAGVIYWQQLGPVLGSVLLILLSAWYMSLQHELIHGHPTRWRWINQLFGTLPFAVWYPYG
ymoll0001_39610  FVTVVYWQQVGVVMGSVLLILLSTWYMSLQHELIHGHPTRWRWVNQLLGTLPLAVWYPYG
yfred0001_8680   FACVIYWQQLGTVLGSVLLILLSTWYMSLQHELIHGHPTRWRWVNQLLGTLPLAIWYPYG
yente0001X_1411  FACVIYWQQLGTVLGSILLILFTAWYMSLQHELIHGHPTRWRGVNQLLGTLPLAVWYPYG
ykris0001_9650   FACVIYWQQLGPVLGSTLLILLSAWYMSLQHELIHGHPTRWRRVNQLLGTLPLAVWYPYG
                 ############################################################


                        130       140       150       160       170       180
                 =========+=========+=========+=========+=========+=========+
yruck0001_4250   L-----------------------------------------------------------
yrohd0001_35770  ------------------------------------------------------------
yinte0001_9570   LYRDSHIQHHRDEDLTHPEKDPECYYYTATQWQYFHILLKGVIRLSNTFVGRLTIGPLLD
ymoll0001_39610  LYRDSHIQHHCDENLTHPEKDPESYYYTGEQWKYLSPLFKRIVRASNTFLGRVVLGPALD
yfred0001_8680   LYRDSHIQHHKNEDLTHPEKDPECYYYTAEQWRHFPSLFKSIIRISNTFIGRLVVGPALD
yente0001X_1411  LYRDSHIQHHKDEDLTHPEKDPECYYYTAKQWQHFPQLFKGIVRISNTFSGRLILGPALD
ykris0001_9650   LYRDSHIQHHKDEDLTHPEKDPECYYYTAEQWQRFPPLFKSIVRINNTFIGRLILGPALD
                 ############################################################


                        190       200       210       220       230       240
                 =========+=========+=========+=========+=========+=========+
yruck0001_4250   --------------------------LELNSSILFFLIFYSIKAFFTAIIICHMALNLAD
yrohd0001_35770  ------------------------------ELVLHWVDNYGISAVFSVLVVSYPAQSL--
yinte0001_9570   IINTLRSAGRAFMSGDRPAIFMWLIHLGLLYGLLYWMAGYGISAVFYLLVISYPALSLTK
ymoll0001_39610  IIDTIRSAARAFLSGDRQAIAMWLVHLGLLIGLFHWMASYGISTLFYLLVVSYPALSLTK
yfred0001_8680   IIDTIRSASRAFITGDRPAIIMWLVHFGLLSLLLYWMVGYGISVMFTILVIGYSALSLTK
yente0001X_1411  IIATIRSTIRAFITGDHPAIAMWLAHFGLLGLLFYWMAGYGISAIFYILVISYPALSLTK
ykris0001_9650   IIDTIRNAISAFITGDRPAMAMWLVHFGLLGLLFHWMAGYGISAIFYILVISYPALSLTK
                 ############################################################


                        250       260       270       280       290       300
                 =========+=========+=========+=========+=========+=========+
yruck0001_4250   ------------------------------------------------------------
yrohd0001_35770  --------------------------------------HH--------------------
yinte0001_9570   VRSFFEHRAAQESSARSVLNEAAWPWRLLFLNLNYHLVHHDLPALPWYGLRQIYLENRVQ
ymoll0001_39610  VRSFFEHRAVENSAARSTLNEAAWPWRLLFLNLNYHLVHHDLPSLPWYGLRQIYLENRVE
yfred0001_8680   VRSFFEHRAAENSAARSTLNEAAWPWRLLFLNLNYHLVHHDLPTLPWYGLRQIYLESRVQ
yente0001X_1411  VRSFFEHRAAENSAARSTLNEAAWPWRLLFLNLNYHLVHHDLPALPWYGLRRIYLESRVQ
ykris0001_9650   IRSFFEHRAAENSAARSTLNEAAWPWRLLFLNLNYHLVHHDLPALPWYGLRQIYLENRVQ
                 ############################################################


                        310       320       330       340       350       360
                 =========+=========+=========+=========+=========+=========+
yruck0001_4250   -------------------YAYE-------------------------------------
yrohd0001_35770  ------------------------------------------------------------
yinte0001_9570   YQQRSGQFVVNGYTQWFDAFAFTALEIEVHPFYDCENKTVAAEERSGVTKSSHHRSAST-
ymoll0001_39610  YQHRSEQFVVHGYNQWIEAHAFTALEIEVHPFYDSGNRALVGQEQHGVIEN-RRHAPSIE
yfred0001_8680   YQHRSEQFVVNGYTQWLETFAFTALEIEIHPFYESENKTPISELLHKRGNRADRQTDSSQ
yente0001X_1411  YQHRSEQFVVNGYTEWFEAFVFTALEIEIHPFYDSENKAPAEERLHTVIDRGNGHTESV-
ykris0001_9650   YQHRSEQFVVNGYTEWFEAFVFTALEIETHPFYDSENKALTERIQDTVIDSGDRRSESV-
                 #####################################                       


                 
                 ===
yruck0001_4250   ---
yrohd0001_35770  ---
yinte0001_9570   ---
ymoll0001_39610  SS-
yfred0001_8680   VAR
yente0001X_1411  ---
ykris0001_9650   ---
```

```
Parameters used
Minimum Number Of Sequences For A Conserved Position: 4
Minimum Number Of Sequences For A Flanking Position: 5
Maximum Number Of Contiguous Nonconserved Positions: 8
Minimum Length Of A Block: 10
Allowed Gap Positions: With Half
Use Similarity Matrices: Yes
```

```
Flank positions of the 1 selected block(s)
Flanks: [45  337]  

New number of positions in PGL1_unique_yersinia-CLUSTERS.dir/PGL1_unique_yersinia-CL3004/PGL1_unique_yersinia-CL3004.muscle.fasta.gblo:  293  (80% of the original 363 positions)
```
