# Supplementary material for: Genomic characterization of the Yersinia genus
Source: Genome Biol. 2010 Jan 4;11(1):R1. doi: 10.1186/gb-2010-11-1-r1 (PMC2847712; doi:10.1186/gb-2010-11-1-r1)
Supplement: Additional file 17 — The top level directory consists of a directory called Additional_cluster_files and 5010 directories, one for each multi-protein cluster family. (This top level directory has been split into three data files for uploading purposes (Additional files 15, 16, 17.) Within the directory are the following files: PGL1_unique_Yersinia_unclustered.out - list of all protein singletons that MCL did not group into a cluster (see Materials and Methods); PGL1_Yersinia_unique_locus_tags.txt - names of the 11 locus tag prefixes used for each genome; PGL1_unique_Yersinia.gff - mapping each Yersinia protein to a cluster in tab delimited GFF; PGL1_unique_Yersinia.sigfile - list of the longest protein in each cluster; PGL1_unique_Yersinia.summary - summary table of features of each of the clusters; PGL1_unique_Yersinia.table - summary table of each protein in the clusters. Within each cluster directory are the following files, where 'x' is the cluster name: PGL1_unique_Yersinia-x.faa - multifasta file of the proteins in the cluster; PGL1_unique_Yersinia-x.summary - summary of the properties of the proteins; PGL1_unique_Yersinia-x.matches - blast matches between the proteins of the cluster; PGL1_unique_Yersinia-x.muscle.fasta - muscle alignment of the proteins; PGL1_unique_Yersinia-x.muscle.fasta.gblo - gblocks output of muscle alignment (that is, auto-trimmed alignment); PGL1_unique_Yersinia-x.muscle.fasta.gblo.htm - as above in html format; PGL1_unique_Yersinia-x.muscle.tree - treefile from muscle alignment; PGL1_unique_Yersinia-x.sif - matches between proteins in simple interaction format for display on graphing software. [file gb-2010-11-1-r1-S17.zip › clusters3/PGL1_unique_yersinia-CL3006/PGL1_unique_yersinia-CL3006.muscle.fasta.gblo.htm]

PGL1\_unique\_yersinia-CL3006.muscle.fasta


## Gblocks 0.91b Results

Processed file: **PGL1\_unique\_yersinia-CL3006.muscle.fasta**  
Number of sequences: **7**  
Alignment assumed to be: **Protein**  
New number of positions: **278** (selected positions are underlined in blue)

```
                         10        20        30        40        50        60
                 =========+=========+=========+=========+=========+=========+
yfred0001_22980  -------MANWLDNSR--------------------------------------------
yinte0001_21860  -------MANWLDHSR--------------------------------------------
yente0001X_3419  ------------------------------------------------------------
yrohd0001_19430  ---VNLAMANWLDHSR--------------------------------------------
yfred0001_34180  -----MTVTIKLNDARREEYRRQG------------------------------------
ypseu0001X_3674  VNLEQQEVRFWLEGERIKFRAPEGVMTGAVIASLKALKPQLVAILAVREQQETLPIDTEN
ypest0001X_9800  VNLEQQEVRFWLEGERIKFRAPEGVMTGAVMASLKALKPQLVAILAVREQQETLPIDTEN
                                                                             


                         70        80        90       100       110       120
                 =========+=========+=========+=========+=========+=========+
yfred0001_22980  ----------------------------------------DDALVIAICGTTEITLHQFR
yinte0001_21860  ----------------------------------------DDDLVIAMCGDKIITLSQFR
yente0001X_3419  ------------------------------------------------------------
yrohd0001_19430  ----------------------------------------DDALVIAMCGTKSITLNQLR
yfred0001_34180  --------------------------------------YWGDASLADYWHHSVKVAPEKI
ypseu0001X_3674  RYAAFPLSHVQAAYFVGRQSVFEYGGVGCHGYFEILAPCWDKAKLECVWEQMIIRHDMLR
ypest0001X_9800  RFVAFPLSHVQAAYFVGRQSVFEYGGVGCHGYFEILAPCWDKAKLECIWEQMIIRHDMLR
                                                                      #######


                        130       140       150       160       170       180
                 =========+=========+=========+=========+=========+=========+
yfred0001_22980  YDVTSLFNKITLLPNERWALC-----------------FENSYW--FTV-----------
yinte0001_21860  CDVTYLFNKINFLPNQRWALC-----------------FENSYW--FTV-----------
yente0001X_3419  ---------MAIFSTSRWALC-----------------FENSYW--FTI-----------
yrohd0001_19430  CDVAYLSHKMTLLPNMRWALC-----------------FENSYW--FTV-----------
yfred0001_34180  AVIDNQGASYTYAALDRAAACLAAYLLDCGIQPGDRVAFQLPGWSEFTL-----------
ypseu0001X_3674  AIITPNGEQRILPSVAPFVIEQVDVSSETVRQAMSHRVYQTDKWPLFDLKISHQNGKDCL
ypest0001X_9800  AIITPNGEQRILPSVAPFVIEQVDVSSETVRQAMSHRVYQTDKWPLFDLKISHQNGKDCL
                 #####################                                       


                        190       200       210       220       230       240
                 =========+=========+=========+=========+=========+=========+
yfred0001_22980  ---------------ALLATLYCQ-------KTPVIFGHAREAVLKEQQHEF-----DG-
yinte0001_21860  ---------------ALLATLYCQ-------KTPVIFGHAREAVLKEQLHEF-----DG-
yente0001X_3419  ---------------ALLATLYCK-------KKPIIFGHAREAVLKEQLHQF-----DG-
yrohd0001_19430  ---------------ALLATLYCK-------KTPVIFGHAREAVLKEQLHEF-----DG-
yfred0001_34180  ---------------IYLACLKTGAV-----SVPLLPAY-RETELIWTLNKS-----QAR
ypseu0001X_3674  HFSIDLLIADFLSVQILLAELFKGYLGQTFPTAPLTASF-RDVICYERQQQSGRAYGDAR
ypest0001X_9800  HFSIDLLIADFLSVQILLAELFKGYLGQTFPAAPLTASF-RDVICYERQQQSGRAYGDAR
                                                #####################        


                        250       260       270       280       290       300
                 =========+=========+=========+=========+=========+=========+
yfred0001_22980  ------------------------------MLTDQ-------------------LLHLDC
yinte0001_21860  ------------------------------MLTDQ-------------------LLNLAC
yente0001X_3419  ------------------------------MLTDQ-------------------LLNLNC
yrohd0001_19430  ------------------------------MLTDQ-------------------QLNLEC
yfred0001_34180  VLFV------------------------PTLFKNTDPVAMITPLRSQLP-----YLQQVV
ypseu0001X_3674  QYWFSRLPTLPACPQLPTCVQLPTGSPSPAMQTNASEIPRFVRYRHQLAPSCWRRLQQGC
ypest0001X_9800  QYWFSRLPTLPACPQLPTCVQLPTGSPSPAMQTNASEIPRFVRYRHQLAPSCWRRLQQGC
                                                                        #####


                        310       320       330       340       350       360
                 =========+=========+=========+=========+=========+=========+
yfred0001_22980  QNIRITDHASVEAIYH------------------------------PLPDWSP-------
yinte0001_21860  PSVTVASHAAIGADYH------------------------------PLPDWPS-------
yente0001X_3419  PTVMVADHAPVDIICH------------------------------PLPDWPS-------
yrohd0001_19430  PRIMVTGHVSPEAIYS------------------------------PLPNWPS-------
yfred0001_34180  AVDKLAPATSLPSLSQ------------------------------LLRDYSP-------
ypseu0001X_3674  QQQGVSPSAFLLTVFSEVIGRWSENRHFTLNLTVMNRPNIHKDIDKLVGDFTSVSLLEVD
ypest0001X_9800  QQQGVSPSAFLLTVFSEVIGRWSENRHFTLNLTVMNRPNIHKDIDKLVGDFTSVSLLEVD
                 #########                                                   


                        370       380       390       400       410       420
                 =========+=========+=========+=========+=========+=========+
yfred0001_22980  --------------------------------------------NAALILFTSGSTGT--
yinte0001_21860  --------------------------------------------AASLILFTSGSTGA--
yente0001X_3419  --------------------------------------------DASLILFTSGSTGA--
yrohd0001_19430  --------------------------------------------DASLILFTSGSTGA--
yfred0001_34180  LENPVKVH----------------------------------GDELAVVLFTSGTEGV--
ypseu0001X_3674  LRSPKTVYERVKQIQKQLWQDLEHRTFSGIEVMREWGRQQGVGHARMPIVFTSALVGEGK
ypest0001X_9800  LRSPKTVYERVKQIQKQLWQDLEHRTFSGIEVMREWGRQQGVGHARMPIVFTSALVGEGK
                                                                             


                        430       440       450       460       470       480
                 =========+=========+=========+=========+=========+=========+
yfred0001_22980  --------------------------------------------------PKAIVKS---
yinte0001_21860  --------------------------------------------------PKAIVKS---
yente0001X_3419  --------------------------------------------------PKAVVKS---
yrohd0001_19430  --------------------------------------------------PKAVVKS---
yfred0001_34180  --------------------------------------------------PKGVMLT---
ypseu0001X_3674  NQVQEAISETQLDLVFDYGISQTPQVWIDCQIMVLQGRLQLNWDVMADVFPKGVVAAMFS
ypest0001X_9800  NQVQEAISETQLDLVFDYGISQTPQVWIDCQIMVLQGRLQLNWDVMADVFPKGVVAAMFS
                                                                             


                        490       500       510       520       530       540
                 =========+=========+=========+=========+=========+=========+
yfred0001_22980  --------VECLDIESHWLAEQWNPCFDKKDNPLIVASVSHQHLYGLTFRIFLPLSLGIP
yinte0001_21860  --------VASLDTESHWLAVQWGQRFDRKSNPLIVASVSHQHLYGLTFRIFLPLSLGIP
yente0001X_3419  --------IASLDIESHWLAAKWGQYFDKKSNPLIIASVSHQHLYGLTFRLFLPLSLGIP
yrohd0001_19430  --------IEGLDIESHWLAMQWGRYIDRANNPLIVASVSHQHLYGLTFRIFLPLSLGIP
yfred0001_34180  --------------HNNVLASERAYCATLNLN------------WMDT--ILMPAPLGHA
ypseu0001X_3674  TFIDSVERIDSVECIDSIECIDRVKCLCMEEVDTGEALCDSPLFWHSA--LSLPLPESQQ
ypest0001X_9800  TFIDSVERIDSVECIDSIECIDRVKCLCMEEVGTGEALCDSPLFWHSA--LSLPLPESQQ
                         ##################################################  


                        550       560       570       580       590       600
                 =========+=========+=========+=========+=========+=========+
yfred0001_22980  FQAELM--------------TYHEQLQLLPK-----------------------------
yinte0001_21860  FQAKLI--------------GYHEQLQSLPE-----------------------------
yente0001X_3419  FQAELI--------------GYHEQLQQLPE-----------------------------
yrohd0001_19430  FQAELI--------------GYHEQLQFLPE-----------------------------
yfred0001_34180  T-------------------GFLH------------------------------------
ypseu0001X_3674  QRRKQVNATEKTLTPRCLHDGILARAAQQPQAIALVDPQHSLTYAALIARAQALAAKLNS
ypest0001X_9800  QRRKQVNATEKTLTPRCLHDGILARAAQQPQAIALVDPQHCLTYAALITRAQALAAKLNS
                                     ##########                              


                        610       620       630       640       650       660
                 =========+=========+=========+=========+=========+=========+
yfred0001_22980  ----------------SCSPIFISSPAFLKRIDTKLAPILCRQIFTAGG-----------
yinte0001_21860  ----------------SCSPIFISSPAFLKRMDTKLSPISCRQIFSAGG-----------
yente0001X_3419  ----------------LFSPIFISSPAFLKRIDTKLPPIQCQQIFSAGG-----------
yrohd0001_19430  ----------------CCSPIFISSPAFLKRLDVKLPPIQCQQIFSAGG-----------
yfred0001_34180  ----------------GVTAPFIIGARSVL-LDIFN-PIDCLTLLAREKCTCVMGATP--
ypseu0001X_3674  HQRYAVLMEKRHEQVVAVLGIFIAGSAYVP-VDIHQPPARILTILSDAAVSGVVTASPQA
ypest0001X_9800  HQRYAVLMEKRHEQVVAVLGIFIAGSAYVP-VDIHQPPARILTILSDAAVSGVVTASPQA
                                     ##########################              


                        670       680       690       700       710       720
                 =========+=========+=========+=========+=========+=========+
yfred0001_22980  ------------------------------------------------------------
yinte0001_21860  ------------------------------------------------------------
yente0001X_3419  ------------------------------------------------------------
yrohd0001_19430  ------------------------------------------------------------
yfred0001_34180  ------------------------------------------------------FVYDLL
ypseu0001X_3674  CLADAHFREINLSLLAQIPDAIEAKILPLPHDLAYVIYTSGSTGQPKGVMISHDAAYNTL
ypest0001X_9800  CLADAHFREINLSLLAQIPDAIEAKILPLPHDLAYVIYTSGSTGQPKGVMISHDAAYNTL
                                                                             


                        730       740       750       760       770       780
                 =========+=========+=========+=========+=========+=========+
yfred0001_22980  ---------------------PLSFH----------------------------------
yinte0001_21860  ---------------------PLSYH----------------------------------
yente0001X_3419  ---------------------PLNFH----------------------------------
yrohd0001_19430  ---------------------PLSFH----------------------------------
yfred0001_34180  CTVQQQ---------------PYDIS----------------------------------
ypseu0001X_3674  ADMQQRMALTPDDRVLALARLSFDLSVFDIFGVLGAGGALIFPDEGDRQNPARWAHDIAQ
ypest0001X_9800  ADMQQRMALTPDDRVLALARLSFDLSVFDIFGVLGAGGALIFPDEGDLQNPARWAHDIAQ
                                                                             


                        790       800       810       820       830       840
                 =========+=========+=========+=========+=========+=========+
yfred0001_22980  ----------------------------DAQSTRNALGILP-------------------
yinte0001_21860  ----------------------------DAQSTLNTLGILP-------------------
yente0001X_3419  ----------------------------DAQATLKILGVLP-------------------
yrohd0001_19430  ----------------------------DAQSTLAVLGILP-------------------
yfred0001_34180  ----------------------------SLRFFLCGGTTIP-------KQITRDCLQAGI
ypseu0001X_3674  HQITLWNSVPAQMKMLTDYLRAEQITLPSLRYILLSGDWIPVNLPPAIAQIAPHCTQ---
ypest0001X_9800  HQITLWNSVPAQMKMLTDYLRAEQITLPSLRYILLSGDWIPVNLPPAIAQIAPHCTQ---
                                                                             


                        850       860       870       880       890       900
                 =========+=========+=========+=========+=========+=========+
yfred0001_22980  --TEIYGATET---------------------GLIAYRQQSEQQQAWQ----FFSGVTLD
yinte0001_21860  --TEIYGATET---------------------GLIAYRHQSAPEQAWQ----FFSGVSLE
yente0001X_3419  --TEIYGATET---------------------GLIAYRQQLEQLQPWQ----FFSGVTLA
yrohd0001_19430  --TEIYGATET---------------------GLIAHRQQFEPQQPWQ----FFSGITLD
yfred0001_34180  KLLSVYGATESSPHAVVKLDDP--LSRVVNTDGTAVLGVEIKVVDKARKTIPYGEEGEEA
ypseu0001X_3674  --LALGGATEAAIWSNYWRIDPQITYPVSIPYGVPLTNQQFRVVNPWGEDCPDWGAGELL
ypest0001X_9800  --LALGGATEAAIWSNYWRIDPQITYPVSIPYGVPLTNQQFRVVNPWGEDCPDWGAGELL
                                                 ################    ########


                        910       920       930       940       950       960
                 =========+=========+=========+=========+=========+=========+
yfred0001_22980  MNSDKTFTVY--------SDLISESNG---VLMSDVIELFDDKRSFYLVGRKDRIVKIEE
yinte0001_21860  INSDKTFTVY--------SALIPESTG---VPMSDIIELSDNRQSFYLLGRQDRIVKIEE
yente0001X_3419  INNDNTFTVY--------SALIPESTG---MPMSDIIELSDNGQSFYLAGRQDRIVKIEE
yrohd0001_19430  INSDNTFTVY--------SALIPESTG---MPMSDIIELSDDGQGFHLLGRQDRIVKIEE
yfred0001_34180  SRGPNVFVGYLGE--PELTAQALDEEG--WYYSGDLCRMDEEGYIKIT-GRKKDIIVRGG
ypseu0001X_3674  IGGRGVAQGYWQDEAKTQAHFFMDTQGLRWYRTGDLGRYTSEGVIEFL-GRRDHQIKVRG
ypest0001X_9800  IGGRGVAQGYWQDEAKTQAHFFIDTQGLRWYRTGDLGRYTSEGVIEFL-GRRDHQIKVRG
                 ##########                      ############################


                        970       980       990      1000      1010      1020
                 =========+=========+=========+=========+=========+=========+
yfred0001_22980  KRVSLTEIEQRLISLPDIADATVL------------------------------------
yinte0001_21860  KRVSLTEIEQRLISLPEIADATVL------------------------------------
yente0001X_3419  KRVSLTEIEQRLTSLPDIAEATVL------------------------------------
yrohd0001_19430  KRVSLTEIEQRLLLLPDIADATVL------------------------------------
yfred0001_34180  ENISSREVEEILLLHPLVQDVGV-------------------------------------
ypseu0001X_3674  YRVETGEIEAQLLKLPEVAQAVVMQTTQAIQTEQAEQATQIAQATQAAQ-----------
ypest0001X_9800  YRVETGEIEAQLLKLPEVAQAVVMQTTQAIQTEQAEQATQIAQATQIAQATQATQATQAT
                 ########################                                    


                       1030      1040      1050      1060      1070      1080
                 =========+=========+=========+=========+=========+=========+
yfred0001_22980  ------------------------------------------------------------
yinte0001_21860  ------------------------------------------------------------
yente0001X_3419  ------------------------------------------------------------
yrohd0001_19430  ------------------------------------------------------------
yfred0001_34180  ------------------------------------------------------------
ypseu0001X_3674  ----------------------------------------IAQATPTTHTELQAYIEVAQ
ypest0001X_9800  QATQAAQAAQTAQTTQTTQTTQTAQATQIAQTTQATPTTPTTQATPTTHTELQAYIEAAQ
                                                                             


                       1090      1100      1110      1120      1130      1140
                 =========+=========+=========+=========+=========+=========+
yfred0001_22980  ------------------------------------------------------------
yinte0001_21860  ------------------------------------------------------------
yente0001X_3419  ------------------------------------------------------------
yrohd0001_19430  ------------------------------------------------------------
yfred0001_34180  ------------------------------------------------------------
ypseu0001X_3674  VAAASDYTQSLREQGRTALAHLQSAAQQAGDALDRPCIDRLFSLLDKVALMQMVKALSDM
ypest0001X_9800  VAAASDYTQSLREQGRTALAHLQSAAQQAGDALDRPCIDRLFSLLDKVALMQMVKALSDM
                                                                             


                       1150      1160      1170      1180      1190      1200
                 =========+=========+=========+=========+=========+=========+
yfred0001_22980  ------------------------------------------------------------
yinte0001_21860  ------------------------------------------------------------
yente0001X_3419  ------------------------------------------------------------
yrohd0001_19430  ------------------------------------------------------------
yfred0001_34180  ------------------------------------------------------------
ypseu0001X_3674  QTVNTHHAVTLDAIMEKGQIASVNRQLLRRWLRALTQHQYLQQINDTYRLISVVAEQEIR
ypest0001X_9800  QTVNTHHAVTLDAIMEKGQIASVNRQLLRRWLRALTQHQYLQQINDTYRLISVVAEQEIR
                                                                             


                       1210      1220      1230      1240      1250      1260
                 =========+=========+=========+=========+=========+=========+
yfred0001_22980  ------------------------------------------VLTQNERVNIAAV-----
yinte0001_21860  ------------------------------------------VLTHNERVNIAAV-----
yente0001X_3419  ------------------------------------------VLTQNERVSIAAV-----
yrohd0001_19430  ------------------------------------------LLTQNERVNIAAV-----
yfred0001_34180  ------------------------------------------VAMPDERLGERACAY---
ypseu0001X_3674  LCWQQCEQLITQLDDNRGLLTYLERSSQCLPELLQGKEDPLNLLFPDGRLDVATQAYQNN
ypest0001X_9800  LCWQQCEQLITQLDDNRGLLTYLERSSQCLPELLQGKEDPLNLLFPDGRLDVATQAYQNN
                                                            ###########      


                       1270      1280      1290      1300      1310      1320
                 =========+=========+=========+=========+=========+=========+
yfred0001_22980  --------VVLTDDGKEK------------------------------------FSSATM
yinte0001_21860  --------VVLTDMGKEQ------------------------------------VAAASL
yente0001X_3419  --------VVLTDYGKEQ------------------------------------LSTATV
yrohd0001_19430  --------VVLTDAGKEQ------------------------------------LCTETM
yfred0001_34180  --------VVLRDPDVPL------------------------------------TFAAMI
ypseu0001X_3674  LISQFMNRLLLKAAEQKVKNQAQGRVLKVFEIGAGVGGTSNVLIPLFAEGNAEYTFTDIS
ypest0001X_9800  LISQFMNRLLLKAAEQKVKNQAQGRVLKVFEIGAGVGGTSNVLIPLFAEGNAEYTFTDIS
                                                                             


                       1330      1340      1350      1360      1370      1380
                 =========+=========+=========+=========+=========+=========+
yfred0001_22980  ASFNQKLRSDLRHW----------------------------------------------
yinte0001_21860  GFLTQSLRSTLRHW----------------------------------------------
yente0001X_3419  GSFTQNLRSSLRNW----------------------------------------------
yrohd0001_19430  SSFNQKLRSALRNW----------------------------------------------
yfred0001_34180  DFFRSRRVAKYKY-----------------------------------------------
ypseu0001X_3674  PFFLNEARKRYQHYDFIHYQLFDFNQSPVQQGLNCGQYDLVVAANVLHNAIIARHGLNNL
ypest0001X_9800  PFFLNEARKRYQHYDFIHYQLFDFNQSPVQQGLDCGQYDLVVAANVLHNAIIARHGLNNL
                                                                             


                       1390      1400      1410      1420      1430      1440
                 =========+=========+=========+=========+=========+=========+
yfred0001_22980  ------------------------------------------------------------
yinte0001_21860  ------------------------------------------------------------
yente0001X_3419  ------------------------------------------------------------
yrohd0001_19430  ------------------------------------------------------------
yfred0001_34180  ------------------------------------------------------------
ypseu0001X_3674  RQLLAPGGWLLIIEATRDNYQLMTSMEFKQGLTAFEDERLALDSPFLPQQNWIHALQDVG
ypest0001X_9800  RQLLAPGGWLLIIEATRDNYQLMTSMEFKQGLTAFEDERLALDSPFLPQQNWIHALQDVG
                                                                             


                       1450      1460      1470      1480      1490      1500
                 =========+=========+=========+=========+=========+=========+
yfred0001_22980  ---------------------------------------------LEPASLPRRLRVIES
yinte0001_21860  ---------------------------------------------LDPVSLPRRLRVIDV
yente0001X_3419  ---------------------------------------------LEPVSLPRRLRVIDV
yrohd0001_19430  ---------------------------------------------LEPASLPRRVRVIDV
yfred0001_34180  ---------------------------------------------------PERLELVDH
ypseu0001X_3674  AEMLWAYPPTDDVLHKMGQSFIFAQFNSQRMSCEPDALLAYLQQQLPDYMVPAKLVILDK
ypest0001X_9800  AEMLWAYPPTDDVLHKMGQSFIFAQFNSQRMSCEPDALLAYLQQQLPDYMVPAKLVILDK
                                                              ###############


                       1510      1520      1530      1540      1550      1560
                 =========+=========+=========+=========+=========+=========+
yfred0001_22980  IPVNPQGKRDYARLQEL-------------------------------------------
yinte0001_21860  IPLNPQGKRDYARLQEL-------------------------------------------
yente0001X_3419  MPVNLQGKRDYALLQEL-------------------------------------------
yrohd0001_19430  IPVNPQGKRDYARLQEL-------------------------------------------
yfred0001_34180  LPRTASGKIKKFLLREL-------------------------------------------
ypseu0001X_3674  LPVSANGKIDRKQLPRLPESQQKQAPALQDTDSPLEKTLLALGRTLIGNNAMGVDDDFFT
ypest0001X_9800  LPVSANGKIDRKQLPRLPESQQRQAPALQDTDSPLEKTLLALGRTLIGNNAMGVDDDFFT
                 #################                                           


                       1570      1580      1590      1600      1610      1620
                 =========+=========+=========+=========+=========+=========+
yfred0001_22980  ------------------------------------------------------------
yinte0001_21860  ------------------------------------------------------------
yente0001X_3419  ------------------------------------------------------------
yrohd0001_19430  ------------------------------------------------------------
yfred0001_34180  ------------------------------------------------------------
ypseu0001X_3674  SGGDSLLITQWINQVREVLGEDKVPWEGCLRQVLQQPTARALAAYLRNIREEEPESQRPQ
ypest0001X_9800  SGGDSLLITQWINQVREVLGEDKVPWEGCLRQVLQQPTARALAAYLRNIREEEPESQRPQ
                                                                             


                       1630      1640      1650      1660      1670      1680
                 =========+=========+=========+=========+=========+=========+
yfred0001_22980  ------------------------------------------------------------
yinte0001_21860  ------------------------------------------------------------
yente0001X_3419  ------------------------------------------------------------
yrohd0001_19430  ------------------------------------------------------------
yfred0001_34180  ------------------------------------------------------------
ypseu0001X_3674  MAVTCLKESAGLKERSGLKERSEEPVVILHEGSGTVLPYLSLVDKLSGPIYGVNVTDSAA
ypest0001X_9800  MAVTCLKESACLKERSGLKERSEEPVVILHEGSGTVLPYLSLVDKLSGPIYGVNVTDSAA
                                                                             


                       1690      1700      1710      1720      1730      1740
                 =========+=========+=========+=========+=========+=========+
yfred0001_22980  FL----------------------------------------------------------
yinte0001_21860  FL----------------------------------------------------------
yente0001X_3419  FL----------------------------------------------------------
yrohd0001_19430  FL----------------------------------------------------------
yfred0001_34180  LL----------------------------------------------------------
ypseu0001X_3674  FLSIPAERLICQLASTYLPEIQKVGDQCHLVGYCMGGLLAFEIARQALENGRPVSSLTII
ypest0001X_9800  FLSIPAERLICQLASTYLPEIQKVGDQCHLVGYCMGGLLAFEIARQALENGRPVSSLTII
                                                                             


                       1750      1760      1770      1780      1790      1800
                 =========+=========+=========+=========+=========+=========+
yfred0001_22980  ------------------------------------------------------------
yinte0001_21860  ------------------------------------------------------------
yente0001X_3419  ------------------------------------------------------------
yrohd0001_19430  ------------------------------------------------------------
yfred0001_34180  ------------------------------------------------------------
ypseu0001X_3674  SSYQIPYHINDPLMIDLVMSWTLGVEPPWNLLKADLEGIYHAILADKPAVITVNAVVERA
ypest0001X_9800  SSYQIPYHINDPLMIDLVMSWTLGVEPPWNLLKADLEGIYHAILADKPAVITVNAVVERA
                                                                             


                       1810      1820      1830      1840      1850      1860
                 =========+=========+=========+=========+=========+=========+
yfred0001_22980  ------------------------------------------------------------
yinte0001_21860  ------------------------------------------------------------
yente0001X_3419  ------------------------------------------------------------
yrohd0001_19430  ------------------------------------------------------------
yfred0001_34180  ------------------------------------------------------------
ypseu0001X_3674  KANGLTALANEYACLQQQSNEVRLSQLLTAIEQHSTASSHVSRQPFDVFRHSIQGVCQYQ
ypest0001X_9800  KANGLTALANEYACLQQQSNEVRLSQLLTAIEQHSTASSHVSRQPFDVFRHSIQGVCQYQ
                                                                             


                       1870      1880      1890      1900      1910      1920
                 =========+=========+=========+=========+=========+=========+
yfred0001_22980  ------------------------------------------------------------
yinte0001_21860  ------------------------------------------------------------
yente0001X_3419  ------------------------------------------------------------
yrohd0001_19430  ------------------------------------------------------------
yfred0001_34180  ------------------------------------------------------------
ypseu0001X_3674  PNYFAGDMTFVCQQGDTYLLPALQQDMTAFWQTYCLGQINRLTLPGDHFTCLSADNISPL
ypest0001X_9800  PNYFAGDMTFVCQQGDTYLLPALQQDMTAFWQTYCLGQINRLTLPGDHFTCLSADNISPL
                                                                             


                       1930
                 =========+==
yfred0001_22980  ------------
yinte0001_21860  ------------
yente0001X_3419  ------------
yrohd0001_19430  ------------
yfred0001_34180  ------------
ypseu0001X_3674  IQHLEQCKGVRG
ypest0001X_9800  IQHLEQCKGVRG
```

```
Parameters used
Minimum Number Of Sequences For A Conserved Position: 4
Minimum Number Of Sequences For A Flanking Position: 5
Maximum Number Of Contiguous Nonconserved Positions: 8
Minimum Length Of A Block: 10
Allowed Gap Positions: With Half
Use Similarity Matrices: Yes
```

```
Flank positions of the 11 selected block(s)
Flanks: [114  141]  [212  232]  [296  309]  [489  538]  [561  570]  [621  646]  [873  888]  [893  910]  [933  984]  [1244  1254]  [1486  1517]  

New number of positions in PGL1_unique_yersinia-CLUSTERS.dir/PGL1_unique_yersinia-CL3006/PGL1_unique_yersinia-CL3006.muscle.fasta.gblo:  278  (14% of the original 1932 positions)
```
