# Supplementary material for: Genomic characterization of the Yersinia genus
Source: Genome Biol. 2010 Jan 4;11(1):R1. doi: 10.1186/gb-2010-11-1-r1 (PMC2847712; doi:10.1186/gb-2010-11-1-r1)
Supplement: Additional file 17 — The top level directory consists of a directory called Additional_cluster_files and 5010 directories, one for each multi-protein cluster family. (This top level directory has been split into three data files for uploading purposes (Additional files 15, 16, 17.) Within the directory are the following files: PGL1_unique_Yersinia_unclustered.out - list of all protein singletons that MCL did not group into a cluster (see Materials and Methods); PGL1_Yersinia_unique_locus_tags.txt - names of the 11 locus tag prefixes used for each genome; PGL1_unique_Yersinia.gff - mapping each Yersinia protein to a cluster in tab delimited GFF; PGL1_unique_Yersinia.sigfile - list of the longest protein in each cluster; PGL1_unique_Yersinia.summary - summary table of features of each of the clusters; PGL1_unique_Yersinia.table - summary table of each protein in the clusters. Within each cluster directory are the following files, where 'x' is the cluster name: PGL1_unique_Yersinia-x.faa - multifasta file of the proteins in the cluster; PGL1_unique_Yersinia-x.summary - summary of the properties of the proteins; PGL1_unique_Yersinia-x.matches - blast matches between the proteins of the cluster; PGL1_unique_Yersinia-x.muscle.fasta - muscle alignment of the proteins; PGL1_unique_Yersinia-x.muscle.fasta.gblo - gblocks output of muscle alignment (that is, auto-trimmed alignment); PGL1_unique_Yersinia-x.muscle.fasta.gblo.htm - as above in html format; PGL1_unique_Yersinia-x.muscle.tree - treefile from muscle alignment; PGL1_unique_Yersinia-x.sif - matches between proteins in simple interaction format for display on graphing software. [file gb-2010-11-1-r1-S17.zip › clusters3/PGL1_unique_yersinia-CL3008/PGL1_unique_yersinia-CL3008.muscle.fasta.gblo.htm]

PGL1\_unique\_yersinia-CL3008.muscle.fasta


## Gblocks 0.91b Results

Processed file: **PGL1\_unique\_yersinia-CL3008.muscle.fasta**  
Number of sequences: **7**  
Alignment assumed to be: **Protein**  
New number of positions: **72** (selected positions are underlined in blue)

```
                         10        20        30        40        50        60
                 =========+=========+=========+=========+=========+=========+
yruck0001_29460  VNLFPENVSALFPVTLEPCLPGDDGDTDTPAIQLDEPDNLTLCQLLTPLVSHFYYCSMTR
yruck0001_19180  ---------------------------------------MTKNHLAPPLSPAYLLNQCTL
ypest0001X_3199  ---------------------MKIIYITFASAVLFNSPSLAYNPLFSSFPPIYWVYNSPT
ypseu0001X_3225  ---------------------MKIIYITFASAVLFSSPSLAYNPLFSSFPPIYWVYNSPT
ykris0001_24160  -------------------------------------MKIGYI----TFTSAMLFCLSTA
yfred0001_25170  -------------------------------------MQISYIALIGTLLTSFFATAQP-
yinte0001_23310  ----------------------------------------------MILANIMLINLSVM
                                                                             


                         70        80        90       100       110       120
                 =========+=========+=========+=========+=========+=========+
yruck0001_29460  HEAGYDCDVVLLPLTPL------TEHLKAIKQSTPQVPLTERLLKAFRRWANINVELLRL
yruck0001_19180  PFFCHEQDRPAAPLFIP--PLLASNNGKLEIQRQNPAWINKNL--S----DLYSVQ----
ypest0001X_3199  ATTWLKLENQFNPLYLMTSSFWFTQHGNSAQQLTNLALITSNGAVS----GNFSIE----
ypseu0001X_3225  ATTWLKLENQFNPLYLMTRSFWFTQHGNSAQQLTNLALITSNGAVS----GNFSIE----
ykris0001_24160  A----------QPLIAS-------------GLLIKQISLASSLTVS----NDFSVH----
yfred0001_25170  -----------PPLDYL--------HVNLMSFSKNPSS------------DNFSVY----
yinte0001_23310  A----------QPITLE-------------DLRVKPILINNSHILT----DNFSVH----
                                                                             


                        130       140       150       160       170       180
                 =========+=========+=========+=========+=========+=========+
yruck0001_29460  TLPRRQQNFGIEQQIWGHESAVHSPAIRRRARKLTDDCCDFCGYTSKHNALIFRNSNPED
yruck0001_19180  ---------GIKSEYF--------------------------------------------
ypest0001X_3199  ---------GVQTNYW--------------------------------------------
ypseu0001X_3225  ---------GVQTNYW--------------------------------------------
ykris0001_24160  ---------GVQPSYW--------------------------------------------
yfred0001_25170  ---------GIQTNYW--------------------------------------------
yinte0001_23310  ---------GVQTHYW--------------------------------------------
                                                                             


                        190       200       210       220       230       240
                 =========+=========+=========+=========+=========+=========+
yruck0001_29460  TADTNLGVACPVCACSRHLNNLGANDGVMVYLPELSPADISHLLRTVSIARQQGDKRQKQ
yruck0001_19180  ---------------------LVNGRGQI----KVEITTLAPLLITAYLFDINGKEQAQT
ypest0001X_3199  ---------------------LDRGQGQI----EITATTHEHLLITAYLFDVNGQEKAQT
ypseu0001X_3225  ---------------------LDRGQGQI----EITATTHEHLLITAYLFDVNGQEKAQT
ykris0001_24160  ---------------------LKDGQGQI----EMTIATHDHLLITGYLYDVEGHEKARA
yfred0001_25170  ---------------------LENSQGEI----PMTVTTQENLLITVYIYDQYNNEKAQT
yinte0001_23310  ---------------------LENSSGEI----PMTVTTLEHLLITTYLYDQYDDEKTQT
                                                  ###########################


                        250       260       270       280       290       300
                 =========+=========+=========+=========+=========+=========+
yruck0001_29460  GATTILRWLAEHRAEAEAFWGTCHPGEFGQALLQARDRLREDLQQRLR--HIALIPNPEL
yruck0001_19180  EE-----WVNNQSKELMLALENLSHGRYRLVI----KALDADGRSAIKVHHISLI-----
ypest0001X_3199  AT-----LINNRTKNLILLLDNIVAGNYRLVI----KALATDHQTAIKTFHITLA-----
ypseu0001X_3225  AT-----LINNRTKNLILLLDNIVAGNYRLVI----KALATDHQTAIKTFHITLA-----
ykris0001_24160  ET-----LINNRTKDFSLPLEGIAAGNYKLVI----KALNTEHQSAIKNFNIALA-----
yfred0001_25170  AT-----LINNRAKGMTLLLEDFSAGNYRLVV----TSLNTQHQLVIKTYYMALT-----
yinte0001_23310  AT-----LIDNRSKNMTLFLVNLPPGNYRLVV----TGLATDHQISSKTFHMTLA-----
                 ##     #########################    ##################      


                        310       320
                 =========+=========+======
yruck0001_29460  ISGNITADSIKPSTWLSLLNQYRSQN
yruck0001_19180  ----------------NRSMTTPS--
ypest0001X_3199  ----------------DIVPQTR---
ypseu0001X_3225  ----------------DIVPQTR---
ykris0001_24160  ----------------NFAEN-----
yfred0001_25170  ----------------NMTSEDLSY-
yinte0001_23310  ----------------NQTSGYDSSY
```

```
Parameters used
Minimum Number Of Sequences For A Conserved Position: 4
Minimum Number Of Sequences For A Flanking Position: 5
Maximum Number Of Contiguous Nonconserved Positions: 8
Minimum Length Of A Block: 10
Allowed Gap Positions: With Half
Use Similarity Matrices: Yes
```

```
Flank positions of the 3 selected block(s)
Flanks: [214  242]  [248  272]  [277  294]  

New number of positions in PGL1_unique_yersinia-CLUSTERS.dir/PGL1_unique_yersinia-CL3008/PGL1_unique_yersinia-CL3008.muscle.fasta.gblo:  72  (22% of the original 326 positions)
```
