# Supplementary material for: Genomic characterization of the Yersinia genus
Source: Genome Biol. 2010 Jan 4;11(1):R1. doi: 10.1186/gb-2010-11-1-r1 (PMC2847712; doi:10.1186/gb-2010-11-1-r1)
Supplement: Additional file 17 — The top level directory consists of a directory called Additional_cluster_files and 5010 directories, one for each multi-protein cluster family. (This top level directory has been split into three data files for uploading purposes (Additional files 15, 16, 17.) Within the directory are the following files: PGL1_unique_Yersinia_unclustered.out - list of all protein singletons that MCL did not group into a cluster (see Materials and Methods); PGL1_Yersinia_unique_locus_tags.txt - names of the 11 locus tag prefixes used for each genome; PGL1_unique_Yersinia.gff - mapping each Yersinia protein to a cluster in tab delimited GFF; PGL1_unique_Yersinia.sigfile - list of the longest protein in each cluster; PGL1_unique_Yersinia.summary - summary table of features of each of the clusters; PGL1_unique_Yersinia.table - summary table of each protein in the clusters. Within each cluster directory are the following files, where 'x' is the cluster name: PGL1_unique_Yersinia-x.faa - multifasta file of the proteins in the cluster; PGL1_unique_Yersinia-x.summary - summary of the properties of the proteins; PGL1_unique_Yersinia-x.matches - blast matches between the proteins of the cluster; PGL1_unique_Yersinia-x.muscle.fasta - muscle alignment of the proteins; PGL1_unique_Yersinia-x.muscle.fasta.gblo - gblocks output of muscle alignment (that is, auto-trimmed alignment); PGL1_unique_Yersinia-x.muscle.fasta.gblo.htm - as above in html format; PGL1_unique_Yersinia-x.muscle.tree - treefile from muscle alignment; PGL1_unique_Yersinia-x.sif - matches between proteins in simple interaction format for display on graphing software. [file gb-2010-11-1-r1-S17.zip › clusters3/PGL1_unique_yersinia-CL3010/PGL1_unique_yersinia-CL3010.muscle.fasta.gblo.htm]

PGL1\_unique\_yersinia-CL3010.muscle.fasta


## Gblocks 0.91b Results

Processed file: **PGL1\_unique\_yersinia-CL3010.muscle.fasta**  
Number of sequences: **6**  
Alignment assumed to be: **Protein**  
New number of positions: **0** (selected positions are underlined in blue)

```
                         10        20        30        40        50        60
                 =========+=========+=========+=========+=========+=========+
ymoll0001_25140  --------MQRNLLKYLKSFTGKIRKCQEEAVEKFFKFKKKNIRDKACLINLPTGAGKTG
yaldo0001_1280   ------------------------------------------------------------
yruck0001_1440   MLLWIQSQPLRVISIIFAGSSLAYYMLMTTLAIYLHSYLKLTVATVGTIIFGYVFISRLA
yaldo0001_1270   ------------------------------------------------------------
yaldo0001_1260   ------------------------------------------------------------
yente0001X_2220  ------------------------------------------------------------
                                                                             


                         70        80        90       100       110       120
                 =========+=========+=========+=========+=========+=========+
ymoll0001_25140  VISLISHLSKERNILIICHRRAVKEQLYKEVSSRFFRVTLNDPDIKLKNTFKNINNLNEE
yaldo0001_1280   ----------------------------------MRLWKRPQPMQPIAIIFVGS------
yruck0001_1440   KVILGPWFDRLSLRTSLTFSLSIAAAGFFLAATDNLIGILAPWCLCIAGIGISSVVLLTQ
yaldo0001_1270   ------------------------------------MGKFASWCLCIAGVGVSSVVLLTQ
yaldo0001_1260   ------------------------------------------------------------
yente0001X_2220  ----------------------------------MRFWKRPQPMLPIATIFV--------
                                                                             


                        130       140       150       160       170       180
                 =========+=========+=========+=========+=========+=========+
ymoll0001_25140  GIYISTFQKLSMLSPEDLDKTQSFFDLIIIDEGHSEPSPVWREIVRQSDAIKVVITATPY
yaldo0001_1280   ------------------------------------------------------------
yruck0001_1440   SCIAR--QKQALLNEVHAPADYSFIYVLMHSSALVAPAIGFTIMRHFPGGLYWIIGGFYL
yaldo0001_1270   SFIAS--QKQLRSHNAASSADDSFLYILMNGSALIAP-----------------------
yaldo0001_1260   ------------------------------------------------------------
yente0001X_2220  ------------------------------------------------------------
                                                                             


                        190       200       210       220       230       240
                 =========+=========+=========+=========+=========+=========+
ymoll0001_25140  RNDLFELNVDLDDYFIFTFKQAISDKIITEPNFIQVNSMEKMLQEVQIFLAKNENIKCII
yaldo0001_1280   ------------------------------------------------------------
yruck0001_1440   LLLLF------------------CRLFLPEPQLPPPATTNKAVITDFLTAFRKQHYCRFL
yaldo0001_1270   ------------------------------------------------------------
yaldo0001_1260   ------------------------------------------------------------
yente0001X_2220  --------------------------------------------------FKNRNYCRFL
                                                                             


                        250       260       270       280       290       300
                 =========+=========+=========+=========+=========+=========+
ymoll0001_25140  KCKDANDIYLYHESISKKFKTASIHETFRNDEASGKFKSVNSALKSDNIRVLIHQHKLDE
yaldo0001_1280   --------------------------------------SLAYYVLMTTFSTYLHEH-L--
yruck0001_1440   LINSLLWMLYAQLFSTIPLYIREILGEKSNLTLFYMVEALTVMMLQHWVSTYINQH-IQA
yaldo0001_1270   -----------------------------------------------VIGFEILRY-WPQ
yaldo0001_1260   -------------------------------------------VLQHWVSLYINQH-IPA
yente0001X_2220  LVNSLLWMLHAQLYSTIPLYTRETLDDESHLTLFFMVEALCVMVLQHWVNLYINQH-IPS
                                                                             


                        310       320       330       340       350       360
                 =========+=========+=========+=========+=========+=========+
ymoll0001_25140  GVDFPEAKLLVLTYQVGSGRELVQTIGRVVRNYNSIKPMIIDLASSSNERMWQSYRVFDD
yaldo0001_1280   --RFPVTTVSAIMFGY----------------------ILVSRLSKVALGPW--------
yruck0001_1440   --YFHQVALVIFSLSF----------------------LLIYYAHSMVLILL--------
yaldo0001_1270   GLYWIIGGLYILLLVY----------------------CLAYISGAECKPLP--------
yaldo0001_1260   --YYPAVALTLFSVSF----------------------FLIYLAQDRVLILW--------
yente0001X_2220  --YYPAVTLMLFSVSF----------------------FLIYLAQDRVLILW--------
                                                                             


                        370       380       390       400       410       420
                 =========+=========+=========+=========+=========+=========+
ymoll0001_25140  YISTPSGSKGFIKSLSTTNLIKGFLDNFPEYSYFSSRFRERLDLQSINANDISIPLASVC
yaldo0001_1280   -------------------------------------F----------------------
yruck0001_1440   ---------------AGCLFAFALMIFMPCSNAVNAAFAEP-------------------
yaldo0001_1270   ---------------APHLVLADWL----------KPFKNR-------------------
yaldo0001_1260   ---------------AAGVFSLALMVYMPSADANNAAFADH-------------------
yente0001X_2220  ---------------AAGVFSLALMVYMPSADANNAAFADH-------------------
                                                                             


                        430       440       450       460       470       480
                 =========+=========+=========+=========+=========+=========+
ymoll0001_25140  FIEKGPNYSTPLLLDKIYWELHTQ-GSLVKQIENDHDVSIYLYISFNSSRYLSDKLFFEP
yaldo0001_1280   -----DRLTFRSGLIFA--------------------LGIVNY-----------------
yruck0001_1440   -----GRFATYFGLVSLSATLGDSLGSA---------LGMTGFNLLLQSGMLRDYFLWLA
yaldo0001_1270   ------NYCRFLLVNSLLWMLHAQLYST---------IPLYTRETLDDESNLTLFFYRGS
yaldo0001_1260   -----DRFATYFGLLSLSATLGDSLGNI---------LGMMLLAFLLQSGNVSLYFLWLS
yente0001X_2220  -----GSFATYFGLLSLSVTLGDSLGNM---------LGMRLLAFLLQSGNVSQYFLWLA
                                                                             


                        490       500       510       520       530       540
                 =========+=========+=========+=========+=========+=========+
ymoll0001_25140  KLEIIIIKELSNSIAIFDSSGGKYANRIDLNLVNPININRLTALAAVTKAREIKEAHSRA
yaldo0001_1280   ----------TPNVRHSTK-----------------------------------------
yruck0001_1440   G---I-----TVALAFLRR--------------NPYNCLL--------------------
yaldo0001_1270   A---I-----CHGVAALGQ--------------SLY------------------------
yaldo0001_1260   GLAAI-----TAVLAIFCR--------------NPYNCH---------------------
yente0001X_2220  G---I-----TAVLAIFYR--------------NPYNCPL--------------------
                                                                             


                        550       560
                 =========+=========+
ymoll0001_25140  IGTAKNRPEAISLKGKNLEI
yaldo0001_1280   --------------------
yruck0001_1440   --------------------
yaldo0001_1270   --------------------
yaldo0001_1260   --------------------
yente0001X_2220  --------------------
```

```
Parameters used
Minimum Number Of Sequences For A Conserved Position: 4
Minimum Number Of Sequences For A Flanking Position: 5
Maximum Number Of Contiguous Nonconserved Positions: 8
Minimum Length Of A Block: 10
Allowed Gap Positions: With Half
Use Similarity Matrices: Yes
```

```
Flank positions of the 0 selected block(s)
Flanks: 

New number of positions in PGL1_unique_yersinia-CLUSTERS.dir/PGL1_unique_yersinia-CL3010/PGL1_unique_yersinia-CL3010.muscle.fasta.gblo:  0  (0% of the original 560 positions)
```
