# Supplementary material for: Genomic characterization of the Yersinia genus
Source: Genome Biol. 2010 Jan 4;11(1):R1. doi: 10.1186/gb-2010-11-1-r1 (PMC2847712; doi:10.1186/gb-2010-11-1-r1)
Supplement: Additional file 17 — The top level directory consists of a directory called Additional_cluster_files and 5010 directories, one for each multi-protein cluster family. (This top level directory has been split into three data files for uploading purposes (Additional files 15, 16, 17.) Within the directory are the following files: PGL1_unique_Yersinia_unclustered.out - list of all protein singletons that MCL did not group into a cluster (see Materials and Methods); PGL1_Yersinia_unique_locus_tags.txt - names of the 11 locus tag prefixes used for each genome; PGL1_unique_Yersinia.gff - mapping each Yersinia protein to a cluster in tab delimited GFF; PGL1_unique_Yersinia.sigfile - list of the longest protein in each cluster; PGL1_unique_Yersinia.summary - summary table of features of each of the clusters; PGL1_unique_Yersinia.table - summary table of each protein in the clusters. Within each cluster directory are the following files, where 'x' is the cluster name: PGL1_unique_Yersinia-x.faa - multifasta file of the proteins in the cluster; PGL1_unique_Yersinia-x.summary - summary of the properties of the proteins; PGL1_unique_Yersinia-x.matches - blast matches between the proteins of the cluster; PGL1_unique_Yersinia-x.muscle.fasta - muscle alignment of the proteins; PGL1_unique_Yersinia-x.muscle.fasta.gblo - gblocks output of muscle alignment (that is, auto-trimmed alignment); PGL1_unique_Yersinia-x.muscle.fasta.gblo.htm - as above in html format; PGL1_unique_Yersinia-x.muscle.tree - treefile from muscle alignment; PGL1_unique_Yersinia-x.sif - matches between proteins in simple interaction format for display on graphing software. [file gb-2010-11-1-r1-S17.zip › clusters3/PGL1_unique_yersinia-CL3014/PGL1_unique_yersinia-CL3014.muscle.fasta.gblo.htm]

PGL1\_unique\_yersinia-CL3014.muscle.fasta


## Gblocks 0.91b Results

Processed file: **PGL1\_unique\_yersinia-CL3014.muscle.fasta**  
Number of sequences: **6**  
Alignment assumed to be: **Protein**  
New number of positions: **419** (selected positions are underlined in blue)

```
                         10        20        30        40        50        60
                 =========+=========+=========+=========+=========+=========+
yaldo0001_27860  MSKFIILVIDSFGVGAMPDAAQIRPQDVEANTCGHILQTYPQMRLPHLEKLGLMNALHLA
yfred0001_26800  MSQFIVLVIDSFGVGAMPDVSEVRPQDKEANTCAHILQIYPKLRLPNLEKLGLLNALHIG
ykris0001_26820  MSKFIVLVIDSFGVGAMPDVAEVRPQDIEANTCAHILQTYPELRLPNLEKMGLINALHIG
yente0001X_1157  MSKFIVLVIDSFGVGAMPDVSDVRPQDLGANTCAHILQAYPELRLATLEKLGLLNALHIG
yberc0001_25590  MSKFIVLVIDSFGVGAMPDVAEVRPQDIAANTCAHILQTYPDMRLPHLEKLGLINALHIG
ymoll0001_25950  MSKFIVLVIDSFGVGAMPDVAEVRPQDITANTCAHILQTYPEMHLPHLEKLGLLNALHIG
                 ############################################################


                         70        80        90       100       110       120
                 =========+=========+=========+=========+=========+=========+
yaldo0001_27860  APHFTDSVMQENPQACFGVARLQHVGGDTFMGHQEIMGTLPCAPRLMPFSRVKARVANAL
yfred0001_26800  SPDFTDSVMQDNPHASFGVALLQHEGGDTFMGHQEIMGTLPRAPLSMPFSTVKSRVADAL
ykris0001_26820  SPDFHASVMQDNPEASFGVALLQHEGGDTFMGHQEIMGTLPRSPLSMPFSSVKTRVADAL
yente0001X_1157  SPDFQDSVMQDNPLASFGVALLQHEGGDTFMGHQEIMGTLPRTPLSMPFSTVKSRVADAL
yberc0001_25590  SPDFNESIMQERAQASFGVALLQHEGGDTFMGHQEIMGTLPRSPLSMPFSTVKSRVADAL
ymoll0001_25950  SPDFKESIMQESTQASFGVALLQHEGGDTFMGHQEIMGTLPRAPLSMPFSTVKSQVADAL
                 ############################################################


                        130       140       150       160       170       180
                 =========+=========+=========+=========+=========+=========+
yaldo0001_27860  IRQGYQVEERGAPDDSSELRFLWVNDAVAIGDNLEADLGQVFNICANLNAINFEQVENMG
yfred0001_26800  RQQGYQVEERGAPDDSSNLQFLWVNDCVAIGDNLEADLGQVFNICANLSVIDFSQVEKIG
ykris0001_26820  RQQGYQVEEHSAPNDSSNLQFLWVNNCVAIGDNLEADLGQVFNICANLDAINFEQVEKIG
yente0001X_1157  RQQGYQVEEHSAPDDSSDLQFLWVNNCVAIGDNLEADLGQVFNICANLNAIDFEQVEKIG
yberc0001_25590  RQQGYQVEQRSAPEESSHLQLLWVNNAVAIGDNLEADLGQVFNICANLSAVDFELVEKIG
ymoll0001_25950  RQQGYQVEPRSAVGDSSELQLLWVNDAVAIGDNLEADLGQVFNICANLSAIDFASVEKIG
                 ############################################################


                        190       200       210       220       230       240
                 =========+=========+=========+=========+=========+=========+
yaldo0001_27860  RIVRRCVEVNRVIAYGGLLMDSHEIIRSAEVKQQQYIGINSPKSGVYDNGFQVIHLGYGV
yfred0001_26800  RIVRSCVEVNRVIAYGGRLINSLAIISAAEVKQQDYIGINSPKSGVYDNGFQVIHLGYGV
ykris0001_26820  RIVRGCVEVNRVIAYGGVLMNSHAIISAAEVKQQHYIGINSPKSGVYDNGFQVIHLGYGV
yente0001X_1157  RIVRGCVEVNRVIAYGGQLMNSQAIISAAEVKQQHYIGINSPKSGVYDTGFQVIHLGYGV
yberc0001_25590  RIVRSCVAVNRVIAYGGQLVDSHKIISAAEVKQQQYIGINSPKSGVYDSGFQVIHLGYGV
ymoll0001_25950  RIVRHCVAVNRVIAYGGQLADSQAIIDAVEVKQQQYIGINSPKSGVYDRGFQVIHLGYGV
                 ############################################################


                        250       260       270       280       290       300
                 =========+=========+=========+=========+=========+=========+
yaldo0001_27860  DADVQVPHQLDQVRVPTILVGKVADIVANCAGRSYPQRVDSQTILDITLAEVQRPKSAFI
yfred0001_26800  DTDIQVPHQLEKVNISTVLIGKVADIVANPAGRNYPQLVDSQVILDITLAEVQRAESAFI
ykris0001_26820  DADVQVPHQLEKVNIPTVLVGKVADIVANPAGRSYQQLVDSQTILDITLEEVQRAGSAFI
yente0001X_1157  DADVQVPHQLEKVNIPTVLVGKVADIVANPAGRSYQQLVDSQVILDITLAEVQRAGSAFI
yberc0001_25590  DAAVQVPHQLEQVNVPTVLVGKVADIVANPTGRNYQQLVDSQTILDITLAEVQRAGSAFI
ymoll0001_25950  DAEVQVPHQLEKVNIPTVLVGKVADIVANPAGENYQQLVDSKTIFDITLTEIQRAGSAFI
                 ############################################################


                        310       320       330       340       350       360
                 =========+=========+=========+=========+=========+=========+
yaldo0001_27860  CANIQETDLAGHAQDVERYAERLQLVDSMLGKIMAAMTIDDCLVVMADHGNDPTIGHSKH
yfred0001_26800  CSNIQETDLAGHGQDVERYAERLQLVDTMLGKITAAMAAGDCLVVMADHGNDPTIGHSKH
ykris0001_26820  CTNIQETDLAGHGQDVARYAERLQLVDNMLGKITAAMVQGDCLVVMADHGNDPTIGHSKH
yente0001X_1157  CTNIQETDLAGHGQDVARYAERLQLVDNMLGKITAAMAPGDCLVVMADHGNDPTIGHSKH
yberc0001_25590  CTNIQETDLAGHGQDVERYAERLELVDNMLGKIMAAMATGDCLVIMADHGNDPTIGHSKH
ymoll0001_25950  CTNIQETDLAGHGQDVARYAERLQLVDHMLGEMQAAMAAGDCLVVMADHGNDPTIGHSKH
                 ############################################################


                        370       380       390       400       410       420
                 =========+=========+=========+=========+=========+=========+
yaldo0001_27860  TREHVPVLVYRIN-----QTATQLGIRQTMSDIGATVCEFFAAKAPQNGRSFWKQLAEPR
yfred0001_26800  TREQVPLLVYHAGFNHSRPQGIQLGIRKTMSDVGATVCEFFAAPAPQNGHSFWQQLTNAS
ykris0001_26820  TREQVPLLVYHAGMAQSPQKAIRLGTRETMSDVGATVCEFFAAIAPQNGHSFWRQLTDAS
yente0001X_1157  TREQVPLLVYHPGMKHSPQKAIHLGIRETMSDVGATACEFFAAIAPQNGHSFWRQLTEAS
yberc0001_25590  TREQVPLLVYHAGMTDTQPQAVHLGIRETMSDVGATVCEFFAAPAPQNGHSFWQQLINAS
ymoll0001_25950  TREQVPLLVYLAGVTNAQPQAVRLGIRKTMSDVGATVCEFFAAPAPQNGHSFWQQLIRAR
                 ########################################################### 


                 
                 ==
yaldo0001_27860  N-
yfred0001_26800  K-
ykris0001_26820  K-
yente0001X_1157  K-
yberc0001_25590  K-
ymoll0001_25950  QE
```

```
Parameters used
Minimum Number Of Sequences For A Conserved Position: 4
Minimum Number Of Sequences For A Flanking Position: 5
Maximum Number Of Contiguous Nonconserved Positions: 8
Minimum Length Of A Block: 10
Allowed Gap Positions: With Half
Use Similarity Matrices: Yes
```

```
Flank positions of the 1 selected block(s)
Flanks: [1  419]  

New number of positions in PGL1_unique_yersinia-CLUSTERS.dir/PGL1_unique_yersinia-CL3014/PGL1_unique_yersinia-CL3014.muscle.fasta.gblo:  419  (99% of the original 422 positions)
```
