# Supplementary material for: Genomic characterization of the Yersinia genus
Source: Genome Biol. 2010 Jan 4;11(1):R1. doi: 10.1186/gb-2010-11-1-r1 (PMC2847712; doi:10.1186/gb-2010-11-1-r1)
Supplement: Additional file 17 — The top level directory consists of a directory called Additional_cluster_files and 5010 directories, one for each multi-protein cluster family. (This top level directory has been split into three data files for uploading purposes (Additional files 15, 16, 17.) Within the directory are the following files: PGL1_unique_Yersinia_unclustered.out - list of all protein singletons that MCL did not group into a cluster (see Materials and Methods); PGL1_Yersinia_unique_locus_tags.txt - names of the 11 locus tag prefixes used for each genome; PGL1_unique_Yersinia.gff - mapping each Yersinia protein to a cluster in tab delimited GFF; PGL1_unique_Yersinia.sigfile - list of the longest protein in each cluster; PGL1_unique_Yersinia.summary - summary table of features of each of the clusters; PGL1_unique_Yersinia.table - summary table of each protein in the clusters. Within each cluster directory are the following files, where 'x' is the cluster name: PGL1_unique_Yersinia-x.faa - multifasta file of the proteins in the cluster; PGL1_unique_Yersinia-x.summary - summary of the properties of the proteins; PGL1_unique_Yersinia-x.matches - blast matches between the proteins of the cluster; PGL1_unique_Yersinia-x.muscle.fasta - muscle alignment of the proteins; PGL1_unique_Yersinia-x.muscle.fasta.gblo - gblocks output of muscle alignment (that is, auto-trimmed alignment); PGL1_unique_Yersinia-x.muscle.fasta.gblo.htm - as above in html format; PGL1_unique_Yersinia-x.muscle.tree - treefile from muscle alignment; PGL1_unique_Yersinia-x.sif - matches between proteins in simple interaction format for display on graphing software. [file gb-2010-11-1-r1-S17.zip › clusters3/PGL1_unique_yersinia-CL3015/PGL1_unique_yersinia-CL3015.muscle.fasta.gblo.htm]

PGL1\_unique\_yersinia-CL3015.muscle.fasta


## Gblocks 0.91b Results

Processed file: **PGL1\_unique\_yersinia-CL3015.muscle.fasta**  
Number of sequences: **6**  
Alignment assumed to be: **Protein**  
New number of positions: **136** (selected positions are underlined in blue)

```
                         10        20        30        40        50        60
                 =========+=========+=========+=========+=========+=========+
yente0001X_3808  ---------------------MKINVTDKPNPQDEEYVIDGLWAHNDKTQKVDIHPLFLT
ykris0001_31870  MILQAVVLNVLITEIGRYVPKMNINVTDKPNPQDEEFVIDGLWAHNDKTQPVDIHPLFLT
yrohd0001_3420   ---------------------MKINVTDTPNPQDEEFVIDSLWAHNNKTEVVDIHPLFLT
yfred0001_2810   ---------------------MNINVTDTPNPADEEYVINGLWAHNNKTEVVDIHPLLLT
yaldo0001_3760   ---------------------MNINVTDAPNPQDEEFVIDSLWAHNNKTEVVDIHPLFLT
yinte0001_3840   ---------------------MNINVTDTPNPQDEEYVIDSLWAHNNKTEAVDIHPLFLT
                                      #######################################


                         70        80        90       100       110       120
                 =========+=========+=========+=========+=========+=========+
yente0001X_3808  VTDDDGKIVAGLVARTWWGGLEIQYLWVSDDYRKSGYGRQLMEKAEEEALKRGCHMAYVD
ykris0001_31870  VTDNDGKIVAGLVARTWWGGLEIQYLWVSDEYRKSGYGRQLMEKAEEEALKRECHMAYVD
yrohd0001_3420   ITDEQGKIVAGLVARTWWGGLEVQYLWVSDEHRNSGYGRQLMEQAEAEALQRGCHMAYVD
yfred0001_2810   VTDDNGKIVAGLVARTWWGGLEVQYLWVSDEYRKSGYGRQLMEQAEEEAIKRGCHMAYVD
yaldo0001_3760   VTGDDGKIVAGLVARTWWGGLEVQYLWVSDEYRKSGYGRHLMEKAEEEALKRGCHMAYVD
yinte0001_3840   VTHDNGKIVAGLVARTWWGGLEVQYLWVSDEYRKSGYGRQLMEKAEEEAIKRGCHMAYVD
                 ############################################################


                        130       140       150
                 =========+=========+=========+=======
yente0001X_3808  TFDFQAKGFYEKLGYRVYGNLGGYAKKHTRHYLAKEI
ykris0001_31870  TFDFQAKGFYEKLGYRAYGNLGGYAKKHTRHYLAKEI
yrohd0001_3420   TFDFQARGFYEKLGYRIYGNLGGYAKKHTRHYLAKEI
yfred0001_2810   TFDFQARGFYEKLGYSVYGNLGGYAKKHTRFYLAKEL
yaldo0001_3760   TFDFQARGFYEKLGYRIYGNLGGYAKKHTRHYLAKEI
yinte0001_3840   TFDFQARGFYEKLGYRVYGDLGGYAKKYTRHYLAKEI
                 #####################################
```

```
Parameters used
Minimum Number Of Sequences For A Conserved Position: 4
Minimum Number Of Sequences For A Flanking Position: 5
Maximum Number Of Contiguous Nonconserved Positions: 8
Minimum Length Of A Block: 10
Allowed Gap Positions: With Half
Use Similarity Matrices: Yes
```

```
Flank positions of the 1 selected block(s)
Flanks: [22  157]  

New number of positions in PGL1_unique_yersinia-CLUSTERS.dir/PGL1_unique_yersinia-CL3015/PGL1_unique_yersinia-CL3015.muscle.fasta.gblo:  136  (86% of the original 157 positions)
```
