# Supplementary material for: Genomic characterization of the Yersinia genus
Source: Genome Biol. 2010 Jan 4;11(1):R1. doi: 10.1186/gb-2010-11-1-r1 (PMC2847712; doi:10.1186/gb-2010-11-1-r1)
Supplement: Additional file 17 — The top level directory consists of a directory called Additional_cluster_files and 5010 directories, one for each multi-protein cluster family. (This top level directory has been split into three data files for uploading purposes (Additional files 15, 16, 17.) Within the directory are the following files: PGL1_unique_Yersinia_unclustered.out - list of all protein singletons that MCL did not group into a cluster (see Materials and Methods); PGL1_Yersinia_unique_locus_tags.txt - names of the 11 locus tag prefixes used for each genome; PGL1_unique_Yersinia.gff - mapping each Yersinia protein to a cluster in tab delimited GFF; PGL1_unique_Yersinia.sigfile - list of the longest protein in each cluster; PGL1_unique_Yersinia.summary - summary table of features of each of the clusters; PGL1_unique_Yersinia.table - summary table of each protein in the clusters. Within each cluster directory are the following files, where 'x' is the cluster name: PGL1_unique_Yersinia-x.faa - multifasta file of the proteins in the cluster; PGL1_unique_Yersinia-x.summary - summary of the properties of the proteins; PGL1_unique_Yersinia-x.matches - blast matches between the proteins of the cluster; PGL1_unique_Yersinia-x.muscle.fasta - muscle alignment of the proteins; PGL1_unique_Yersinia-x.muscle.fasta.gblo - gblocks output of muscle alignment (that is, auto-trimmed alignment); PGL1_unique_Yersinia-x.muscle.fasta.gblo.htm - as above in html format; PGL1_unique_Yersinia-x.muscle.tree - treefile from muscle alignment; PGL1_unique_Yersinia-x.sif - matches between proteins in simple interaction format for display on graphing software. [file gb-2010-11-1-r1-S17.zip › clusters3/PGL1_unique_yersinia-CL3016/PGL1_unique_yersinia-CL3016.muscle.fasta.gblo.htm]

PGL1\_unique\_yersinia-CL3016.muscle.fasta


## Gblocks 0.91b Results

Processed file: **PGL1\_unique\_yersinia-CL3016.muscle.fasta**  
Number of sequences: **6**  
Alignment assumed to be: **Protein**  
New number of positions: **582** (selected positions are underlined in blue)

```
                         10        20        30        40        50        60
                 =========+=========+=========+=========+=========+=========+
yaldo0001_40850  MRYWAEVITPKLGQLPSIINVGTFDDDNAAGSSDSVSNGIISLTQLQGALNGIDSGELAF
yfred0001_13920  -----------------------------------------------------------M
ymoll0001_21250  MHHW------------------------------QCASTLFRPLLLSTIISNLFWSGFSV
yberc0001_20900  MHSL------------------------------SCASKLFRPLLLSTIISNILLPGFSI
yente0001X_2945  MHNS------------------------------QHASKLFRPLFLTVAISNIIWPEFTM
yrohd0001_12850  ------------------------------------------------------------
                                                                             


                         70        80        90       100       110       120
                 =========+=========+=========+=========+=========+=========+
yaldo0001_40850  GSHAQFIMGKLDFDDVPYVPAQSPRTGRVDLVSVAVHELAHGLGISNMVSDPHVRDTFTP
yfred0001_13920  AGSSDLFYT----------------STKIDPQSFNLSDVIATD-----------------
ymoll0001_21250  AEDRKLTYS----------------STITDPSSIALSDAIKGD-----------------
yberc0001_20900  AEERKYTYS----------------SAISGPSSVALSDAISGD-----------------
yente0001X_2945  AQTREFTYS----------------ATIPGPSSVALSDAIAGD-----------------
yrohd0001_12850  ------------------------------------------------------------
                                                                             


                        130       140       150       160       170       180
                 =========+=========+=========+=========+=========+=========+
yaldo0001_40850  VFDTQPFGSWASHLRDDHGNPARPGQVILCHGCSNPWDPQGFDVRLDKGYFTGRYVDEVL
yfred0001_13920  -------GNYSANISVD--NPNPALEVYIAQAETD-----------DENYHIGDYFVNIH
ymoll0001_21250  -------GNYNATVNVD--NPNGSLEVYIGQTETN-----------NKTYDVGDYSVNIQ
yberc0001_20900  -------GNYKATVNVD--NPSSSLEIYLGQTETA-----------NKAYDIGDYSVEIN
yente0001X_2945  -------GNYNATINVD--NPDKPLEVYFGQTETL-----------QKTYDIGNYSVDIY
yrohd0001_12850  ------------------------------------------------------------
                        ##########                                           


                        190       200       210       220       230       240
                 =========+=========+=========+=========+=========+=========+
yaldo0001_40850  AGAMPGVPVKTLVDDGSVD-NNYMSHIELKNSMMSHQNYRNYTTFMEAELALLQDMGYQI
yfred0001_13920  G-------------NNILNPEGYTA-----------------------------------
ymoll0001_21250  G-------------QNNTAASPYTA-----------------------------------
yberc0001_20900  G-------------QNTVD-NPYTA-----------------------------------
yente0001X_2945  G-------------QNTTD-SSYTV-----------------------------------
yrohd0001_12850  ------------------------------------------------------------
                                                                             


                        250       260       270       280       290       300
                 =========+=========+=========+=========+=========+=========+
yaldo0001_40850  DRRNFFGFSLYGDGQTLTNRNGYFLRNQQGDGYLAGQYNTAMLGLGLHVYGSNNRIFQQA
yfred0001_13920  ----FFGLSA-QKNNTISLNNFSFINSLVV-GDGH-NNSTALLAANGSNITINGKVYINS
ymoll0001_21250  ----FFGLAA-DRNNTVSLNSFSFNNNLMV-GDAHHHNSTALIASNGSKVTINGKVYINS
yberc0001_20900  ----FFGLAV-DKNNTVSLNNFSYDNKLKIGGDGHHHNSTALMVSNGSEVTINGKVYINS
yente0001X_2945  ----FFGLAV-DSNNAVSINNFSYNNNLKVGGDNHHHNSTALIASDGSKVTIDGKVYINS
yrohd0001_12850  ------------------------------------------------------------
                              ###############################################


                        310       320       330       340       350       360
                 =========+=========+=========+=========+=========+=========+
yaldo0001_40850  DLLTQGAGGAGIRIDGQNNTLNIEPGTRVYADGLNGRGIIFAYGKQH--NLIQRGDIQAL
yfred0001_13920  -LVEMDTSGTGTATTANNGLYAKDEGTSITAN--SGDIYINTYTKNFLELLEHNNTYPEG
ymoll0001_21250  -LVEMETSGASSATAANNGLYATGAGSTITAN--SGDVYINTYGKNFFEVLTQNGTYPSG
yberc0001_20900  -LVEMDTSGTGNTTVANNGLYATGVGSTITAN--SGDVYINTYAKDFLEALNQNDTYPSG
yente0001X_2945  -LVEMDTSGSETASIANNGLYATGMGSTITAN--NGDVYINTYAKNFLELLGENASYTGG
yrohd0001_12850  --------------MANNGLYATGVGSQITAN--NGDIYINTYAKNFLELLGENASYTEG
                  ###############################   #########################


                        370       380       390       400       410       420
                 =========+=========+=========+=========+=========+=========+
yaldo0001_40850  GANGVAISFDFGNNLLGNKADYRGSWIHTVGWYVAALLPELQGALVDNADISGRVAGKGA
yfred0001_13920  GAKSDAISGKYGGQVTVNQTG------------------QYRLNVLGNMDLGDDAAEGGG
ymoll0001_21250  SSKSDAVSGKHGGQVTINQTG------------------NYQVNLLGNLDLGDDFVNRG-
yberc0001_20900  SSKSDAVSGKRGGQVTINETG------------------NYQVNLLGNLDLGSEF-GTGS
yente0001X_2945  GAKSDAVSGKHGGQVIINETG------------------QYQVNLLGNLDLGNAF-GLGS
yrohd0001_12850  ------------------------------------------------------------
                 ##################                             #############


                        430       440       450       460       470       480
                 =========+=========+=========+=========+=========+=========+
yaldo0001_40850  AIYISPNALVGNINILNGARLEGDIYSDYNQRDAYGQQRLT---QLSFGRMANAQGLATD
yfred0001_13920  GITA----------VFNGA----DSYWHGHEVNFYDTTSNAWIGALDVTLMNKAHWIPDE
ymoll0001_21250  SITA----------LLNGA----NSYWHGTEVNTYDAANNLWAGNLDVTLMNKAQWIPDA
yberc0001_20900  SITA----------VLNGS----DSYWHGTEANTYNEATNTWAGKLDVTLMNKAQWIPDA
yente0001X_2945  SITV----------VLNGS----DSYWHGTEANEYNADSNTWAGILDVTLMNKAQWIPDA
yrohd0001_12850  ------------------------------------------------------------
                 ##                     #####################################


                        490       500       510       520       530       540
                 =========+=========+=========+=========+=========+=========+
yaldo0001_40850  QSDPNFSLNYRGNIEGINNLALSARGGKTSLNGEHQIYSMYIAPGATLSGNGGYTLNQEG
yfred0001_13920  LNAEISALNLQK--DGVVNLHGFNLHTNKSQNESVKIYD--------LKGN-------NG
ymoll0001_21250  VNAEISALNLQK--EGVVNLHGLNLHTQKSQNESVKIYD--------LKGS-------EG
yberc0001_20900  VNAEISALNLQK--DGVVNLHGLNLHTNKSQNESVKIYD--------LKGN-------DG
yente0001X_2945  VNAEISALTLQN--GGTVNLHGFNLHTNKSQNEGVKVYD--------LKGS-------DG
yrohd0001_12850  ------------------------------------------------------------
                 ###########    ########################                     


                        550       560       570       580       590       600
                 =========+=========+=========+=========+=========+=========+
yaldo0001_40850  RFVNDGILAPGNSLGKINVTGAYQQSDNGQLLLEVDGRGGHDTLVVDGHAQFNGQLTFVP
yfred0001_13920  IFLID-----------VNTSKTDGQCKNGSDFIEVVSSS-------TGGSHYIEALNANK
ymoll0001_21250  IFLID-----------VNTSKTDDQHKNGSDFIEVVSSS-------TGGTHYIEALNVNK
yberc0001_20900  IFLID-----------VNTSKTDDQRKNGSDFIEVVSSS-------TGGTHYIEALNVNK
yente0001X_2945  IFLID-----------VNISKTDDQRKNDSDFIEVVSSS-------TGGSHYIEALNVDK
yrohd0001_12850  ------------------------------------------------------------
                                  ######################       ##############


                        610       620       630       640       650       660
                 =========+=========+=========+=========+=========+=========+
yaldo0001_40850  QSDWYAANWRLDSQDLLKTASHSGEFSVVNSLLRSPTLTLQTTSLGEEGWQLSMLRASNA
yfred0001_13920  FADLSEDIWVADAANNITFKPYE-QIDITNEYVYDYKPILRSD-----------IKPGDP
ymoll0001_21250  LAGLSDDIWVADAANNVSFKAYD-QIDANNEYVYDYTPILRSD-----------IKEGDP
yberc0001_20900  LADLREDIWVADAANNVSFKAYD-QIDITNEYVYDYKPLLRSD-----------IKEGDP
yente0001X_2945  LANLSEDIWVADAASNVSFKADD-QIDVNNEYVYDYKPIIRSD-----------IRDGDP
yrohd0001_12850  ------------------------------------------------------------
                 ####################                                        


                        670       680       690       700       710       720
                 =========+=========+=========+=========+=========+=========+
yaldo0001_40850  YSQYAPNDNARQVGQALDKIVSIADSDIQPLYRTLDFSSADGGSISNVLQQLSPAAYSAM
yfred0001_13920  ASQYGTNWYITKIDKQLS-----APSHTVMANASLNYATATA--------RLEIDSLNKR
ymoll0001_21250  ASQYGTNWYITGVETQLS-----AGGDTAMANASLNYATATA--------RLEIDSLNKR
yberc0001_20900  VSQYGTNWYITGVDKKLS-----AGSDTALANASVNYATATA--------RIEIDNLNKR
yente0001X_2945  LSQYGTNWYISGIDKKLS-----AVSDTVLANAGVNYATATA--------RLEIDSLNKR
yrohd0001_12850  ------------------------------------------------------------
                  #################     ###################        ##########


                        730       740       750       760       770       780
                 =========+=========+=========+=========+=========+=========+
yaldo0001_40850  FGSSLNREQQITRIISGPNPVATPTPLAEGEWHSFAIPFGGSFWQQRQGGSVGYDARSYG
yfred0001_13920  LGELRNDQQE------------------YGLW----LRYKGGEMKSKHGSYFKNQYHFYQ
ymoll0001_21250  LGELRSDQQE------------------NGLW----LRYKGGEMKSDEGSYFKNRYNFYQ
yberc0001_20900  LGELRSDQQE------------------NGLW----LRYKGGELKSHEGSYFKNRYNFYQ
yente0001X_2945  LGELRNDQQE------------------NGIW----LRYKGGEMKSKDTSHFKNQYDFYQ
yrohd0001_12850  ------------------------------------------------------------
                 #########                           ########################


                        790       800       810       820       830       840
                 =========+=========+=========+=========+=========+=========+
yaldo0001_40850  MVFGAEKQNEQDRNWVYGLHGA-VSGQSVTVKSPETANGKTTAFNLGIQARYGVERPEGM
yfred0001_13920  LGYDNKDENEHGI-WTKGIAAHYLDGKS----EFTYGSGDNKSYGGTIYGSW--QRHEKQ
ymoll0001_21250  LGYDHKDEYENGI-WTTGFAAHYLKGKS----TFDSGSGDNKSYGASLYGSW--NRPEKQ
yberc0001_20900  LGYDHKDENENGI-WTTGLAGHYLDGKS----TFDQGSGDNKSYGASIYGSW--NRPEKQ
yente0001X_2945  LGYDNKDEYENGI-WTKGIAAHYLDGKS----EFDYGSGDNKSYGGSLYSSW--NRPDQQ
yrohd0001_12850  ------------------------------------------------------------
                 ############# ##############     ###################   #####


                        850       860       870       880       890       900
                 =========+=========+=========+=========+=========+=========+
yaldo0001_40850  -YLFGNGRFGIEDSWLD--RSIHVETYGASHHASWTGLTGSLMAGGGYRWALNDNASAGP
yfred0001_13920  DYVDLVLKYSHLKNHFDYQNTFGAGGYGAGSNWSWSASAEY-----GREFSLDNGNFIEP
ymoll0001_21250  DYVDLVLKYSHLKSNFDYQNRLGTVGHGGVSNGAWSASAEY-----GREFSIGNNNFIEP
yberc0001_20900  DYVDLVLKYSHLKSNFDYQNTLGTAGYGSASNGAWSASAEY-----GREFSIGSGNFIEP
yente0001X_2945  DYVDLVFKYSHLKSNFDYRNTLGTGGHGTGSNSSWSVSAEY-----GREFSLDNGNFIEP
yrohd0001_12850  ------------------------------------ASAEY-----GREFSIGNGNFIEP
                 #########################################     ##############


                        910       920       930       940       950       960
                 =========+=========+=========+=========+=========+=========+
yaldo0001_40850  VASLNYTTLHRPGVTESGNEGSRLMLGSETFDSLRSSIGGNGHWILPLASGAAIAADLQI
yfred0001_13920  QGQLVYTYINKANYTTSS--GLEVNQ-----DNINSVIGRA---------GVRVGHRFED
ymoll0001_21250  QGQLVYTHIDQAGYTTSS--GLQVLQ-----DNINSVIGRA---------GVRVGHRFEE
yberc0001_20900  QGQLVYTHINKADYTTSS--GLKVQQ-----DNINSVIGRA---------GVRVGHRFDE
yente0001X_2945  QGQLVYTYLNKADYSTNS--GLLVSQ-----DNINSVIGRA---------GVRVGHRFEG
yrohd0001_12850  QGQLVYTHINKANYATSS--GLQVNQ-----DNINSVIGRA---------GMRVGHRFEE
                 ##################             ##########         ##########


                        970       980       990      1000      1010      1020
                 =========+=========+=========+=========+=========+=========+
yaldo0001_40850  TWDHEL-LDGDVMQQASFANYRSTGFSAKNKVSGRDSLGIKAGMSYKINTDVELGIGIES
yfred0001_13920  NSNNDIYLKADLLHE--FAGDRSVTVLGKDTTLTTKQGGDDSWITYGIGTNIQLTKENNS
ymoll0001_21250  NSNNDIYLKADLLHE--FAGDRHITLQGKDAMLVSQQEGKDSWITYGVGANIQLTEENNS
yberc0001_20900  NSNNDIYLKADLLHE--FAGDRNVTIQGKDATLVSKQDGKDSWIAYGVGTNIQLTEDNNS
yente0001X_2945  KSNNDIYLKADLLRE--FAGDRNVTIRGKDTVLVDNQNGKDNWIVYGIGTNIQFTEDNNS
yrohd0001_12850  NSNNDIYLKADLLHE--FASDRNVTVRGKDAVLVNNQDGKDSWITYGIGTNIQLTEDNNS
                 ###############  ###########################################


                       1030      1040
                 =========+=========+========
yaldo0001_40850  DVFRSGYDSVAGNLSVAWR--------F
yfred0001_13920  RFYLDVEKSAGGDVNTNWQVNAGLRWEW
ymoll0001_21250  RFYLDVEKSSGGDINTQWQVNAGLRWEF
yberc0001_20900  RFYLDVEKSSGGDINTNWQVNAGLRWEF
yente0001X_2945  RFYLDLEKSSGGDINTQWQVNAGLRWEW
yrohd0001_12850  RFYLDLEKSSGGNINTNWQVNAGLRWEW
                 ###########################
```

```
Parameters used
Minimum Number Of Sequences For A Conserved Position: 4
Minimum Number Of Sequences For A Flanking Position: 5
Maximum Number Of Contiguous Nonconserved Positions: 8
Minimum Length Of A Block: 10
Allowed Gap Positions: With Half
Use Similarity Matrices: Yes
```

```
Flank positions of the 20 selected block(s)
Flanks: [128  137]  [254  300]  [302  332]  [336  378]  [408  422]  [444  491]  [496  519]  [558  579]  [587  620]  [662  678]  [684  702]  [711  729]  [757  793]  [795  808]  [814  832]  [836  881]  [887  918]  [932  941]  [951  975]  [978  1047]  

New number of positions in PGL1_unique_yersinia-CLUSTERS.dir/PGL1_unique_yersinia-CL3016/PGL1_unique_yersinia-CL3016.muscle.fasta.gblo:  582  (55% of the original 1048 positions)
```
