# Supplementary material for: Genomic characterization of the Yersinia genus
Source: Genome Biol. 2010 Jan 4;11(1):R1. doi: 10.1186/gb-2010-11-1-r1 (PMC2847712; doi:10.1186/gb-2010-11-1-r1)
Supplement: Additional file 17 — The top level directory consists of a directory called Additional_cluster_files and 5010 directories, one for each multi-protein cluster family. (This top level directory has been split into three data files for uploading purposes (Additional files 15, 16, 17.) Within the directory are the following files: PGL1_unique_Yersinia_unclustered.out - list of all protein singletons that MCL did not group into a cluster (see Materials and Methods); PGL1_Yersinia_unique_locus_tags.txt - names of the 11 locus tag prefixes used for each genome; PGL1_unique_Yersinia.gff - mapping each Yersinia protein to a cluster in tab delimited GFF; PGL1_unique_Yersinia.sigfile - list of the longest protein in each cluster; PGL1_unique_Yersinia.summary - summary table of features of each of the clusters; PGL1_unique_Yersinia.table - summary table of each protein in the clusters. Within each cluster directory are the following files, where 'x' is the cluster name: PGL1_unique_Yersinia-x.faa - multifasta file of the proteins in the cluster; PGL1_unique_Yersinia-x.summary - summary of the properties of the proteins; PGL1_unique_Yersinia-x.matches - blast matches between the proteins of the cluster; PGL1_unique_Yersinia-x.muscle.fasta - muscle alignment of the proteins; PGL1_unique_Yersinia-x.muscle.fasta.gblo - gblocks output of muscle alignment (that is, auto-trimmed alignment); PGL1_unique_Yersinia-x.muscle.fasta.gblo.htm - as above in html format; PGL1_unique_Yersinia-x.muscle.tree - treefile from muscle alignment; PGL1_unique_Yersinia-x.sif - matches between proteins in simple interaction format for display on graphing software. [file gb-2010-11-1-r1-S17.zip › clusters3/PGL1_unique_yersinia-CL3017/PGL1_unique_yersinia-CL3017.muscle.fasta.gblo.htm]

PGL1\_unique\_yersinia-CL3017.muscle.fasta


## Gblocks 0.91b Results

Processed file: **PGL1\_unique\_yersinia-CL3017.muscle.fasta**  
Number of sequences: **6**  
Alignment assumed to be: **Protein**  
New number of positions: **177** (selected positions are underlined in blue)

```
                         10        20        30        40        50        60
                 =========+=========+=========+=========+=========+=========+
yfred0001_6220   VGLYWGSQRIIDQERRRFTLDFSTLVGYVNEQEVFLRNLRSENQRLSTLPLLHVASFYEE
yaldo0001_4540   ------MQAIL-------------------------------------------SSWFIQ
yinte0001_4620   ----MIMQSIQ-------------------------------------------SSWFIQ
yfred0001_3550   ----MIMQAIL-------------------------------------------SSWFIE
ypest0001X_5310  ------MQAIL-------------------------------------------SSWFIQ
ypseu0001X_4950  ------MQAIL-------------------------------------------SSWFIQ
                                                                       ######


                         70        80        90       100       110       120
                 =========+=========+=========+=========+=========+=========+
yfred0001_6220   KKLTDARGRLFVGRESRVSMPFSVVCENETGCPDLKETFSSLGSYLADFYSAFWAASYFP
yaldo0001_4540   GMIKATSDMWRKGWDER-----------NGGNISLRLLAEEVEAYRSDFYS---------
yinte0001_4620   GMIKATSDMWRKGWDER-----------NGGNISLRLLAEEVEPYRGDFAA---------
yfred0001_3550   GMIKATSDMWRKGWDER-----------NGGNISLRLLAEEVEPYRRDFYP---------
ypest0001X_5310  GMIKATSDMWHKGWDER-----------NGGNISLRLLAEEVEPYRRDFYQ---------
ypseu0001X_4950  GMIKATSDMWHKGWDER-----------NGGNISLRLLAEEVEPYRRDFYQ---------
                 #################           ######################          


                        130       140       150       160       170       180
                 =========+=========+=========+=========+=========+=========+
yfred0001_6220   ATTVFFVDGADEMSISVPAINVNAGYEPINIETYHATTEAVRHKLRGNELNGCGSEQHRQ
yaldo0001_4540   --------------------------QPRTVELTQ-------------------------
yinte0001_4620   --------------------------QPRNVELTQ-------------------------
yfred0001_3550   --------------------------QPRHVELTQ-------------------------
ypest0001X_5310  --------------------------QPRKVELTQ-------------------------
ypseu0001X_4950  --------------------------QPRKVELTQ-------------------------
                                                                             


                        190       200       210       220       230       240
                 =========+=========+=========+=========+=========+=========+
yfred0001_6220   KDSEVIWFRAKTLSDQLIGLVSAGLPQGVWKNSLIHTECIYAATLLNRSRLGVLEKRLNP
yaldo0001_4540   -----------------------------------------------------------P
yinte0001_4620   -----------------------------------------------------------P
yfred0001_3550   -----------------------------------------------------------P
ypest0001X_5310  -----------------------------------------------------------P
ypseu0001X_4950  -----------------------------------------------------------P
                                                                             


                        250       260       270       280       290       300
                 =========+=========+=========+=========+=========+=========+
yfred0001_6220   APEHSFWLQHSHERWLTHRKYGLLLGEDNIPNFESTGLHYTLTGIVLKLSDQSGDWTGIY
yaldo0001_4540   APELA-------NCWF------LVTGS---------------------------------
yinte0001_4620   APELA-------NSWF------LVTGS---------------------------------
yfred0001_3550   APELA-------NSWF------LVTGS---------------------------------
ypest0001X_5310  APELA-------NSWF------LVTGS---------------------------------
ypseu0001X_4950  APELA-------NSWF------LVTGS---------------------------------
                                                                             


                        310       320       330       340       350       360
                 =========+=========+=========+=========+=========+=========+
yfred0001_6220   RVSYGSFFRNNVWLPISTLVLLMISVAGCFVYMRWYNRRVVAPAQEAQREILASEAFNRT
yaldo0001_4540   ----GKFFRNVELNPAENLVLLQVSGDGMAYHIHW-------------------------
yinte0001_4620   ----GKFFRNVELNPEDNLVLLQISNDGMAYHIHW-------------------------
yfred0001_3550   ----GKFFRNVELNPAENLVLLQVSNDGMAYHIHW-------------------------
ypest0001X_5310  ----GKFFRNVELNPAENLVLLQVSNDGMAYHIHW-------------------------
ypseu0001X_4950  ----GKFFRNVELNPAENLVLLQVSNDGMAYDIHW-------------------------
                     ###############################                         


                        370       380       390       400       410       420
                 =========+=========+=========+=========+=========+=========+
yfred0001_6220   LIETAPVALCLIDRSEATLIFANALALDWLGVDEEARPHQNDAIKTLFSQLQNVEQGGAI
yaldo0001_4540   ------------------------------GLTQGGLP-TSELAAHFQSHVVRIQVSGGS
yinte0001_4620   ------------------------------GLPQGGLP-TSELAAHFQSHIVRMQVSGGS
yfred0001_3550   ------------------------------GLTQGGLP-TSELAAHFQSHIVRMQVSDGS
ypest0001X_5310  ------------------------------GLTQGGLP-TSELAAHFQSHIVRMQVSGGT
ypseu0001X_4950  ------------------------------GLTQGGLP-TSELAAHFQSHIVRMQVSGGT
                                                        #####################


                        430       440       450       460       470       480
                 =========+=========+=========+=========+=========+=========+
yfred0001_6220   ERLTLPDARIVYVVYAPTRYRQQAVILCAFTDVSAHAEMEKHLTWAKQAADEANDAKSTF
yaldo0001_4540   DRVIMH---------------------CHATN----------------------------
yinte0001_4620   NRVIMH---------------------CHATN----------------------------
yfred0001_3550   NRVIMH---------------------CHATN----------------------------
ypest0001X_5310  NRVIMH---------------------CHATN----------------------------
ypseu0001X_4950  NRVIMH---------------------CHATN----------------------------
                 ######                                                      


                        490       500       510       520       530       540
                 =========+=========+=========+=========+=========+=========+
yfred0001_6220   LATMSHEIRTPLYGALGTLELLSLTQLNNQQRQYVDRIESASQMLLQIISDILDISKIEA
yaldo0001_4540   --------------------LIALS--------YVQKLDNASFTRLLW------------
yinte0001_4620   --------------------LIALS--------YVQKLDNASFTRLLW------------
yfred0001_3550   --------------------LIALS--------YVQKLDNASFTRLLW------------
ypest0001X_5310  --------------------LIALS--------YVQKLENASFTRLLW------------
ypseu0001X_4950  --------------------LIALS--------YVQKLENASFTRLLW------------
                                                  ###############            


                        550       560       570       580       590       600
                 =========+=========+=========+=========+=========+=========+
yfred0001_6220   GQLQLDKSEFNPRELVQECTGTYAGMAHRKGLLLFSIIATDIPERVIGDPARIRQILNNL
yaldo0001_4540   -------------EGSTEC------------LVVFP------------------------
yinte0001_4620   -------------EGSTEC------------LVVFP------------------------
yfred0001_3550   -------------EGSTEC------------LVVFP------------------------
ypest0001X_5310  -------------EGSTEC------------LVVFP------------------------
ypseu0001X_4950  -------------EGSTEC------------LVVFP------------------------
                                                                             


                        610       620       630       640       650       660
                 =========+=========+=========+=========+=========+=========+
yfred0001_6220   ISNAIKFTEIGTVVVRLSQLERSSLSAKFLLEVCDSGVGIDKAEQEKLFTPFYMIDAERN
yaldo0001_4540   -----------------------------------DGIGI---------VPWM-------
yinte0001_4620   -----------------------------------DGIGI---------VPWM-------
yfred0001_3550   -----------------------------------DGIGI---------VPWM-------
ypest0001X_5310  -----------------------------------DGIGI---------VPWM-------
ypseu0001X_4950  -----------------------------------DGIGI---------VPWM-------
                                                                             


                        670       680       690       700       710       720
                 =========+=========+=========+=========+=========+=========+
yfred0001_6220   MAGGAGLGMSICARLAELMDTKIQLRSEPQIGSQFSVELNTELVEAGDTLTPQLNGISVL
yaldo0001_4540   VPGTDGIGSKTAEQMRE-------------------------------------------
yinte0001_4620   VPGTDGIGSQTAEQMRE-------------------------------------------
yfred0001_3550   VPGTDGIGAQTAEQMRE-------------------------------------------
ypest0001X_5310  VPGTDGIGTQTAEQMRE-------------------------------------------
ypseu0001X_4950  VPGTDGIGTQTAEQMRE-------------------------------------------
                 #################                                           


                        730       740       750       760       770       780
                 =========+=========+=========+=========+=========+=========+
yfred0001_6220   VRTPHPELTENICNWLKRWGAEVVTETDALLKTERQTIVLNIVRRVTDIPKGWPWLRLDL
yaldo0001_4540   -----------------------------------HSLVL------------WPF-----
yinte0001_4620   -----------------------------------HSLVL------------WPF-----
yfred0001_3550   -----------------------------------HSLVL------------WPF-----
ypest0001X_5310  -----------------------------------HSLVL------------WPF-----
ypseu0001X_4950  -----------------------------------HSLVL------------WPF-----
                                                                             


                        790       800       810       820       830       840
                 =========+=========+=========+=========+=========+=========+
yfred0001_6220   SLSGEAVASSDVDAYNLSSIGFGLERLHHGVQCTKSPVPTLPHFNLRILVAEDNPLNQVT
yaldo0001_4540   ----------------------------HGI-----------------------------
yinte0001_4620   ----------------------------HGI-----------------------------
yfred0001_3550   ----------------------------HGI-----------------------------
ypest0001X_5310  ----------------------------HGI-----------------------------
ypseu0001X_4950  ----------------------------HGI-----------------------------
                                                                             


                        850       860       870       880       890       900
                 =========+=========+=========+=========+=========+=========+
yfred0001_6220   LKGQLEKLGCEVTIADDGEEALALWDISPHDMVMTDVNMPYLNGYELARKLRNEGVTAPI
yaldo0001_4540   -------FGSGPTLDDA-------------------------------------------
yinte0001_4620   -------FGSGPTLDDA-------------------------------------------
yfred0001_3550   -------FGSGPTLDDA-------------------------------------------
ypest0001X_5310  -------FGSGPTLDDA-------------------------------------------
ypseu0001X_4950  -------FGSGPTLDDA-------------------------------------------
                        ##########                                           


                        910       920       930       940       950       960
                 =========+=========+=========+=========+=========+=========+
yfred0001_6220   IGVTANAMRDEERRCVEAGMNAWLVKPIELKELAELLRKHVQPGWIDDGGNENTFILPEP
yaldo0001_4540   FGLIDTAEKSAE----------IMVKVLSM------------------GGKQQT------
yinte0001_4620   FGLIDTAEKSAE----------IMVKVLSM------------------GGKQQT------
yfred0001_3550   FGLIDTAEKSAE----------IMVKVLSM------------------GGKRQT------
ypest0001X_5310  FGLIDTAEKSAE----------IMVKVLSM------------------GGKKQT------
ypseu0001X_4950  FGLIDTAEKSAE----------IMVKVLSM------------------GGKKQT------
                 ############                                                


                        970       980       990      1000      1010      1020
                 =========+=========+=========+=========+=========+=========+
yfred0001_6220   NVLEKHRSIFLASMKEDLQHLEDGIAQKDADSLIMTLHRMRGALVLAQFRELASEMEMLE
yaldo0001_4540   --ISREQLIALA----------------------------------ARF-----------
yinte0001_4620   --ISREQLIALA----------------------------------ARF-----------
yfred0001_3550   --ISREQLIALA----------------------------------ARF-----------
ypest0001X_5310  --ISREQLIALA----------------------------------ARF-----------
ypseu0001X_4950  --ISREQLIALA----------------------------------ARF-----------
                   ##########                                                


                       1030      1040      1050
                 =========+=========+=========+====
yfred0001_6220   QQMQESHLNEESLADVVAMAAEISQLLVQIESTP
yaldo0001_4540   --------------DVTPMAAALDV---------
yinte0001_4620   --------------GVTPLAAALDV---------
yfred0001_3550   --------------DVTPLAAALDV---------
ypest0001X_5310  --------------DVTPMAAALDA---------
ypseu0001X_4950  --------------DVTPMAAALDA---------
                               ##########
```

```
Parameters used
Minimum Number Of Sequences For A Conserved Position: 4
Minimum Number Of Sequences For A Flanking Position: 5
Maximum Number Of Contiguous Nonconserved Positions: 8
Minimum Length Of A Block: 10
Allowed Gap Positions: With Half
Use Similarity Matrices: Yes
```

```
Flank positions of the 10 selected block(s)
Flanks: [55  77]  [89  110]  [305  335]  [400  426]  [514  528]  [661  677]  [848  857]  [901  912]  [963  972]  [1035  1044]  

New number of positions in PGL1_unique_yersinia-CLUSTERS.dir/PGL1_unique_yersinia-CL3017/PGL1_unique_yersinia-CL3017.muscle.fasta.gblo:  177  (16% of the original 1054 positions)
```
