# Supplementary material for: Genomic characterization of the Yersinia genus
Source: Genome Biol. 2010 Jan 4;11(1):R1. doi: 10.1186/gb-2010-11-1-r1 (PMC2847712; doi:10.1186/gb-2010-11-1-r1)
Supplement: Additional file 17 — The top level directory consists of a directory called Additional_cluster_files and 5010 directories, one for each multi-protein cluster family. (This top level directory has been split into three data files for uploading purposes (Additional files 15, 16, 17.) Within the directory are the following files: PGL1_unique_Yersinia_unclustered.out - list of all protein singletons that MCL did not group into a cluster (see Materials and Methods); PGL1_Yersinia_unique_locus_tags.txt - names of the 11 locus tag prefixes used for each genome; PGL1_unique_Yersinia.gff - mapping each Yersinia protein to a cluster in tab delimited GFF; PGL1_unique_Yersinia.sigfile - list of the longest protein in each cluster; PGL1_unique_Yersinia.summary - summary table of features of each of the clusters; PGL1_unique_Yersinia.table - summary table of each protein in the clusters. Within each cluster directory are the following files, where 'x' is the cluster name: PGL1_unique_Yersinia-x.faa - multifasta file of the proteins in the cluster; PGL1_unique_Yersinia-x.summary - summary of the properties of the proteins; PGL1_unique_Yersinia-x.matches - blast matches between the proteins of the cluster; PGL1_unique_Yersinia-x.muscle.fasta - muscle alignment of the proteins; PGL1_unique_Yersinia-x.muscle.fasta.gblo - gblocks output of muscle alignment (that is, auto-trimmed alignment); PGL1_unique_Yersinia-x.muscle.fasta.gblo.htm - as above in html format; PGL1_unique_Yersinia-x.muscle.tree - treefile from muscle alignment; PGL1_unique_Yersinia-x.sif - matches between proteins in simple interaction format for display on graphing software. [file gb-2010-11-1-r1-S17.zip › clusters3/PGL1_unique_yersinia-CL3019/PGL1_unique_yersinia-CL3019.muscle.fasta.gblo.htm]

PGL1\_unique\_yersinia-CL3019.muscle.fasta


## Gblocks 0.91b Results

Processed file: **PGL1\_unique\_yersinia-CL3019.muscle.fasta**  
Number of sequences: **6**  
Alignment assumed to be: **Protein**  
New number of positions: **0** (selected positions are underlined in blue)

```
                         10        20        30        40        50        60
                 =========+=========+=========+=========+=========+=========+
yaldo0001_6520   ------------------------------------------------LGINPNQGIDLS
yaldo0001_6500   ------------------------------------------------------------
yruck0001_28180  ----------MTTAALSITAVAANAADPGCAGEEMPLLGGVVLTCATDLGIKPNQGFDIS
yente0001X_5890  VKYSRTLLSLLITGALSLTALNTHAAEPGCAGEEMPLLSNAVLTCATDLGINPNQEIDLS
yaldo0001_6510   ------------------------------------------------------------
yaldo0001_6490   ------------------------------------------------------------
                                                                             


                         70        80        90       100       110       120
                 =========+=========+=========+=========+=========+=========+
yaldo0001_6520   AEVSAALSDGKSLFLPAGSYLIGSDIVLNEKI----------------------------
yaldo0001_6500   ------------------------------------------------------------
yruck0001_28180  SEVSAALSDGKSIFFPAGNYLIGSDIVLKDKNSLVGSESGVTIFRGVKPNKSISIGNDTY
yente0001X_5890  AEVSTALSDGKSIFFPAGSYLIGSDMVLNEKNSLVGSEAGVTILRGINPEKAISIGNKTY
yaldo0001_6510   ----------------------------------VGSESGVTILRGVKPDGSISIGNKTY
yaldo0001_6490   ------------------------------------------------------------
                                                                             


                        130       140       150       160       170       180
                 =========+=========+=========+=========+=========+=========+
yaldo0001_6520   ------------------------------------------------------------
yaldo0001_6500   ------------------------------------------------------------
yruck0001_28180  GTIINQLTIKNIIFDNATVNFYGNKRNIEIINNAFINTVSEAQQLSVSHNAFTIHGNVFL
yente0001X_5890  GSPVNQLTIKNIIFDNATVNFYGNKKNITIINNAFINTNSKDEQLTVSHHPFIIHGNVLL
yaldo0001_6510   GIPVNQLTIKNIIFDNATVNFYGNK-----------------------------------
yaldo0001_6490   ------------------------------------------------------------
                                                                             


                        190       200       210       220       230       240
                 =========+=========+=========+=========+=========+=========+
yaldo0001_6520   ------------------------------------------------------------
yaldo0001_6500   ------------------------------------MNYYDIGTFNIINKIKRSAQENNL
yruck0001_28180  RDKNHPGVGFSTYRNNNAKIENNVVGDISDKNLLLTLNYYDIGTFNVINKIKKSAKNKEL
yente0001X_5890  RDKNHPGLGIGTYRNTKTKIENNVIGDISDKNILLTLNYYDVGTFNVINKIKDSAKNQNL
yaldo0001_6510   ------------------------------------------------------------
yaldo0001_6490   ------------------------------------------------------------
                                                                             


                        250       260       270       280       290       300
                 =========+=========+=========+=========+=========+=========+
yaldo0001_6520   ------------------------------------------------------------
yaldo0001_6500   SVSDKQGYFVAGWYATDGLKNSIFRNNIVSGNTLDCLDINGETDKAKCSKNMTRDHAIYI
yruck0001_28180  TVLDEQGYFVAGWYATDGLKNSVFRNNVISGNTLDCLDVSGETDKAKCK--MTRDHVIYI
yente0001X_5890  NVLDEQGYFIAGWYATDGLKDSVFRNNVISGNTLDCLDVSGETEKAKCT--MTRDHVIYI
yaldo0001_6510   ------------------------------------------------------------
yaldo0001_6490   ------------------------------------------------------------
                                                                             


                        310       320       330       340       350       360
                 =========+=========+=========+=========+=========+=========+
yaldo0001_6520   ------------------------------------------------------------
yaldo0001_6500   KQYNNVEIVNNYFSGWPLDAAGNLKFRNASHLYFVGNYLNKTEFNARPYDGNETLNMDNT
yruck0001_28180  KQYNNVDVVNNYFSGWPLDAAGNIKFRNASHLYFAGNYLNKTEFNARPYDNSETLKMDNT
yente0001X_5890  KQYNNVDVVNNYFSGWPLDAAGNLKFRNASHLYFAGNYLNKTEFNARPYNNSDTLNMDNT
yaldo0001_6510   ------------------------------------------------------------
yaldo0001_6490   ------------------------------------------------------------
                                                                             


                        370       380       390       400       410       420
                 =========+=========+=========+=========+=========+=========+
yaldo0001_6520   ------------------------------------------------------------
yaldo0001_6500   FIFNNTLQDTMIGYWQNFTDTDDKYINANNFVVFNNLFLADDQSAKRISSTLRSTHGEFL
yruck0001_28180  FIFNNVLQETAIGYWQNFEDTDEKYIDARNFVVFDNVFSAEDQTASRINTSWRSTHGEFL
yente0001X_5890  FIFNNMLKDSMIGYWQNFEDRDDYYINAKNFVVFDNLFMAEDQTAKRISSTWRSTHGEFL
yaldo0001_6510   ------------------------------------------------------------
yaldo0001_6490   ------------------------------------------------------------
                                                                             


                        430       440       450       460       470       480
                 =========+=========+=========+=========+=========+=========+
yaldo0001_6520   --------------------------------------------VW--------------
yaldo0001_6500   EANNRYADQTPVVTQE-FQSVDIATAKERLPVEKIALLALKPIPLWKK------------
yruck0001_28180  EANNRYTDQTPVLANHLFQSVDITTAKERFPANKSALLAVKPIPLWNKIGNIGGQDLQEN
yente0001X_5890  EANNRYTDQTSVLTGD-FQMVDIASAKALLPADKTALLAVKPIPLWKKMGDISGQDLQDN
yaldo0001_6510   ------------------------------------------------------------
yaldo0001_6490   ---------------------------------------------VEKVGNVSGPDLQDN
                                                                             


                        490       500       510       520       530       540
                 =========+=========+=========+=========+=========+=========+
yaldo0001_6520   ------------------------------------------------------------
yaldo0001_6500   ------------------------------------------------------------
yruck0001_28180  QLVRLDIEVEGYPSQFVVYEPNSSSDYSTYRWAAKLTALFNEKIKGACAGILTSKVTTDN
yente0001X_5890  QLVRLDIEAEGYPSQFVVYSPDSKYHYPGHRWAAKLTALFNEKIKGACAGVLTSNVTTNN
yaldo0001_6510   ------------------------------------------------------------
yaldo0001_6490   QLARLDIEVEGYPSQFVVYAPDRRDNYPTHHWAAKLTALFNEKIKGACAGVLTSNVTTDN
                                                                             


                        550       560       570       580       590       600
                 =========+=========+=========+=========+=========+=========+
yaldo0001_6520   ------------------------------------------------------------
yaldo0001_6500   ------------------------------------------------------------
yruck0001_28180  SCKFMTPMGSKYLNDIYTTEGGAATYTINIINKYLEVGYISGSKIELGQSVKLAVTFEDG
yente0001X_5890  SCKFMAPKGSSYLNNVYTTEGGAATYTTKIIDKYLEVGYISGSKIQLGQTVKLAVTFEDG
yaldo0001_6510   ------------------------------------------------------------
yaldo0001_6490   SCKFMAQKGSSYLNDIYTTEGGPATYTTKIIDKYLEVGYISGSKIQLGQTVRLAVTFEDG
                                                                             


                        610       620       630       640       650       660
                 =========+=========+=========+=========+=========+=========+
yaldo0001_6520   -------------------WVLSLA-----------------------------------
yaldo0001_6500   ------------------------------------------------------------
yruck0001_28180  THKEVTYTPDHEYRTAAHRWTAELAHKINTSIPGLCAGQYTESKGQNNGIAQCKNVIPSN
yente0001X_5890  THKEVAYTPDDEYRTAGHRWTTELAHKINANIPGLCSGKYTESTEQGNGIAKCGNVVPSG
yaldo0001_6510   ------------------------------------------------------------
yaldo0001_6490   THKEVTYTPDNEYRTAGHRWTTELARKINTNIPGLCAGKYTESKAQDNGISECGYVVPSG
                                                                             


                        670       680
                 =========+=========+===
yaldo0001_6520   -----------------------
yaldo0001_6500   -----------------------
yruck0001_28180  SSYLNKIYTVNGQSAKIDISIES
yente0001X_5890  SSYLNKIYTVNGQSAKVAISIN-
yaldo0001_6510   -----KIY---------------
yaldo0001_6490   SSYLNKIYTVNGQAAKVDISISN
```

```
Parameters used
Minimum Number Of Sequences For A Conserved Position: 4
Minimum Number Of Sequences For A Flanking Position: 5
Maximum Number Of Contiguous Nonconserved Positions: 8
Minimum Length Of A Block: 10
Allowed Gap Positions: With Half
Use Similarity Matrices: Yes
```

```
Flank positions of the 0 selected block(s)
Flanks: 

New number of positions in PGL1_unique_yersinia-CLUSTERS.dir/PGL1_unique_yersinia-CL3019/PGL1_unique_yersinia-CL3019.muscle.fasta.gblo:  0  (0% of the original 683 positions)
```
