# Supplementary material for: Genomic characterization of the Yersinia genus
Source: Genome Biol. 2010 Jan 4;11(1):R1. doi: 10.1186/gb-2010-11-1-r1 (PMC2847712; doi:10.1186/gb-2010-11-1-r1)
Supplement: Additional file 17 — The top level directory consists of a directory called Additional_cluster_files and 5010 directories, one for each multi-protein cluster family. (This top level directory has been split into three data files for uploading purposes (Additional files 15, 16, 17.) Within the directory are the following files: PGL1_unique_Yersinia_unclustered.out - list of all protein singletons that MCL did not group into a cluster (see Materials and Methods); PGL1_Yersinia_unique_locus_tags.txt - names of the 11 locus tag prefixes used for each genome; PGL1_unique_Yersinia.gff - mapping each Yersinia protein to a cluster in tab delimited GFF; PGL1_unique_Yersinia.sigfile - list of the longest protein in each cluster; PGL1_unique_Yersinia.summary - summary table of features of each of the clusters; PGL1_unique_Yersinia.table - summary table of each protein in the clusters. Within each cluster directory are the following files, where 'x' is the cluster name: PGL1_unique_Yersinia-x.faa - multifasta file of the proteins in the cluster; PGL1_unique_Yersinia-x.summary - summary of the properties of the proteins; PGL1_unique_Yersinia-x.matches - blast matches between the proteins of the cluster; PGL1_unique_Yersinia-x.muscle.fasta - muscle alignment of the proteins; PGL1_unique_Yersinia-x.muscle.fasta.gblo - gblocks output of muscle alignment (that is, auto-trimmed alignment); PGL1_unique_Yersinia-x.muscle.fasta.gblo.htm - as above in html format; PGL1_unique_Yersinia-x.muscle.tree - treefile from muscle alignment; PGL1_unique_Yersinia-x.sif - matches between proteins in simple interaction format for display on graphing software. [file gb-2010-11-1-r1-S17.zip › clusters3/PGL1_unique_yersinia-CL3020/PGL1_unique_yersinia-CL3020.muscle.fasta.gblo.htm]

PGL1\_unique\_yersinia-CL3020.muscle.fasta


## Gblocks 0.91b Results

Processed file: **PGL1\_unique\_yersinia-CL3020.muscle.fasta**  
Number of sequences: **6**  
Alignment assumed to be: **Protein**  
New number of positions: **394** (selected positions are underlined in blue)

```
                         10        20        30        40        50        60
                 =========+=========+=========+=========+=========+=========+
yinte0001_6660   ---------MIKNRLLHVKVRLLVSL--------IKNEQGAILFPFIIFLPLIIGLIFFS
yente0001X_5950  ---------MLIQPDA-IHKFNHFTL-------FKKNEQGTILISFMIILPFFIALIFIT
ykris0001_4830   ----------LTNTDI-IRKFNRLTQ-------FKKNEHGAILVSFIIIFPFFIALTFII
yaldo0001_6580   ---------MFKNKAVRFKQYS-VGI-------FIKDEIGAILWPFIIFLPLFIGLLYLS
ymoll0001_5880   MDI-MYRIFMLKNRSI-LRKYGLLSL-------FIKNDKGAILLPFVIFLPIFIGLLFLS
yberc0001_6570   MIIKLIRKFMLTNRSI-FRPGNLLSLFIIISRKFIKNDKGAILLPFIIILPFFIALLFLS
                                                  ###########################


                         70        80        90       100       110       120
                 =========+=========+=========+=========+=========+=========+
yinte0001_6660   FELAHFLQKKTKLSDAMEQATLALTVENNNSTPSAAQITKNAEIVSSYAQAYLPAETFST
yente0001X_5950  FEISHYLQRKAKLSDAIEQATLALTIENN-AIPDEPQQIKNNALVLSYANAYLPSKEFSV
ykris0001_4830   LEVSIFLQKKAKLSDAIEQATLALTVEND-GIPNAAQQTKNRELVLSYANAYLPSEGFSD
yaldo0001_6580   FEISHYLQKAAKLSDAIEQATLALTIENNTNNPDETQTEKNISLVNAYARAYLPSESFSA
ymoll0001_5880   FEISQFLQKKAKLSDAIEQATLALTVENN-DIPDANQSQKNRDLVTHYATAYLPSEKFST
yberc0001_6570   FEISQLLQKKAKLSDAIEQATLALTVEND-DLPDELQMRKNVDLVSNFSSAYLPLEHFSV
                 #############################   ############################


                        130       140       150       160       170       180
                 =========+=========+=========+=========+=========+=========+
yinte0001_6660   PTINIIYNNGRIEYGAEINMSYSAKFLSNIQVTNLSTIINATDRGAARKNIISAPIEKTD
yente0001X_5950  PIININDNTYYLEYNAAVTMAYPAKFLTQTSLTNAITDINITDNGVAIKNKAIEASDLTD
ykris0001_4830   PIINIDDNTNYLGYNAAVTMTYPVEFLGRSPLTNSISNIQTTDNGEAIKNKTIEVSEPTD
yaldo0001_6580   PVIDIISHPNYIEYRAATTLNYTPKFLTKELITNIDRRIIVSDNGVAIKNKFTSPGEITD
ymoll0001_5880   PIIDISNNKGHLLYKAETTMSYPAQFLANSPLAN--TKISIADSGAARKDVAVGPSELTD
yberc0001_6570   PEIDIKNNCGQLTYNAKITMSYFANFLSKTAMTNAITTIGTEDNGAAIKQVSTIQDKATD
                 ############################################################


                        190       200       210       220       230       240
                 =========+=========+=========+=========+=========+=========+
yinte0001_6660   VVFVADYSNSMDEYFYHDEN--EPKKIVALREIFNRLNDNVLKNKNIHTIGFIPFSWGTK
yente0001X_5950  VIFVADYSGSMLYNFDVNEP-NDHERINALRSAFRKLHDIIMNNSNINAIGYIPFSWGTK
ykris0001_4830   VVFVADYSGSMLLSFSDDVSIKNGERINALRSAFRILHNTIKNNSNVNTIGFIPFGSGTK
yaldo0001_6580   VVFVVDYSVSMDGNF-GDEK--KTTKIQELRRIFEDLNNTILKNNNTHTIGFVPFSWGTK
ymoll0001_5880   VVFVVDYSSSMINPFHGTYG--SRSKIDELRDIFYKLNGNILKNDNINTIGFIPFSWGIK
yberc0001_6570   VIFVADYSGSMNEGFHGKVP--RGEKINALRDVFNRLNGSILKNSNINLIGFVPFSWGTK
                 ###############          ###################################


                        250       260       270       280       290       300
                 =========+=========+=========+=========+=========+=========+
yinte0001_6660   NRVENGTRIIEYCHLPFVPKKHSPNGDYLRKYILSGLKQFPGLERLEHIDHIEYAKINDE
yente0001X_5950  RIVFENQQQKIYCHFPFSSKIYKPKGNYL----SDEIKKSSNALLLLDY-----------
ykris0001_4830   RKVSENGENKEYCHLPFSPKIYKPNGDYL----SENAEATKNAWTFLDV-----------
yaldo0001_6580   KIIGKGIHRKIYCHFPFVPKTPMPPSYYL-----GDLKSYNPAKELTDA-----------
ymoll0001_5880   KIVGTGQQSKTYCHFPYAPIKHKSTGDYLRQYTASNLKQFLAPENFNYVDNIEYGELSNR
yberc0001_6570   RIVIENSQEKKYCHFPFVPKQYRADNNYFRQYTVSGLKKFPGLEGLTDIDKINYGELTLG
                 ######################################                      


                        310       320       330       340       350       360
                 =========+=========+=========+=========+=========+=========+
yinte0001_6660   IYSNTKNQI-DELNIEDAESAYTFLFRSRHIIQPLTQFDIIEENIDYDATINSILHNSAG
yente0001X_5950  ----------------------------------------IGDIIDYDKTIESITGNA--
ykris0001_4830   ----------------------------------------IGDHIDYKKTIMSITENV--
yaldo0001_6580   ----------------------------------------VKNNIDYDETIKSITANY--
ymoll0001_5880   NNRVNYLKIKDEISHSKSNLANEFMIKTHYINKYYIISNILTSNIDYDKTINLMSKKY--
yberc0001_6570   EYNTLTNVIKNMAKQEYRNKALEFLRITLNIPTYMQQMIFITTTIDYDATIKSINSDA--
                                                             #############   


                        370       380       390       400       410       420
                 =========+=========+=========+=========+=========+=========+
yinte0001_6660   TPPKTINIPINDILNTYNCLYQRNSYSLNENH-SNDIINDMIEMVPAGGTLISSGILSAN
yente0001X_5950  ---QPIDIPMSDVRTKNVCLQASNAYSLEQEQ-YINNIDNIIKMEPYGWTLISSGILSAN
ykris0001_4830   ---QPIDIPMRDIKHKEICLSGTNSYSLEREQ-FDYSIENIIEMAPLGGTLISSGILSAN
yaldo0001_6580   ---NFINIPIDDIKPSSFCLKGSDAYTLRSDDITNDNIQENIEHEVNGLTLISSGILVAN
ymoll0001_5880   ---KSIDIPIDDVLDGNICLSSSTTYSLEFNKVSDESITESLATEPVGLTLVSSGILAAN
yberc0001_6570   ---QYIDIPLDDIINESICLNNSNAYSLDSHNSHDDLIDKMIAMSPLGQTLVSSGILYAN
                      #######################         #######################


                        430       440       450       460       470       480
                 =========+=========+=========+=========+=========+=========+
yinte0001_6660   NLFNENRSNDNKKLMIILSDG-----------------NDSFEKENKKIKG-FMSRKTSS
yente0001X_5950  NLFKKEANNRHRKLMIILSDG-----------------VDTYQDNFLPNKGLFISKTLVE
ykris0001_4830   NIFKETADNGHKKLMIILSDG-----------------MDSYNSTMLPNKGFFISKTLID
yaldo0001_6580   DIFRKDSKNK-DKLMIIISDG-----------------NDQEISSDLTQE--KITKTLIE
ymoll0001_5880   NLFK-EANDKNKKLMIVLSDGEDSDNTTTFDKDGNLIAVDDYIKNDEDRKPFRITKNLID
yberc0001_6570   TLFKKESNNSNNKLMVIISDG-----------------IDVFINDTTIQQSIYISKTLID
                 #####################                                #######


                        490       500       510       520       530       540
                 =========+=========+=========+=========+=========+=========+
yinte0001_6660   KKVCAKKLKR---------MESP-----------WLSSLLDIYLYTTPRHQDI-------
yente0001X_5950  KGMCERVISSGIQMAFIAIAYSPDDDVNEPEYINWRQCVGKDNYYEAHNADELMRDIQQA
ykris0001_4830   EGMCEMIIKNGIQMAFIAIAYSPENNVNAPEYINWKQCVGEDNYYEAHNAHELELELQQA
yaldo0001_6580   KGMCERIKENNIRMVFIGIAYTVKE-------IKWEDCVGKRNYYEAQNAHELEADLRQA
ymoll0001_5880   KGMCEAIAANKIRMVFIAIGYTPVNDAYSPTYIDWEKCVGKDNFYLAKDAHELEADLQQA
yberc0001_6570   KGMCERIKENNIKMVFIAIKDGSNETNEPANYIDWKKCVGEDNYYYVSDAHELEAALRQS
                 ############################################################


                        550
                 =========+======
yinte0001_6660   -------SIGKSV---
yente0001X_5950  ISKSATSEVGRNTPKK
ykris0001_4830   VSVSATSEVGRNTPKQ
yaldo0001_6580   LGTIEASEVGRNIPKN
ymoll0001_5880   LGGENIRDVGRNTPKH
yberc0001_6570   LTTTSSEVVGRNIPKH
                 ###############
```

```
Parameters used
Minimum Number Of Sequences For A Conserved Position: 4
Minimum Number Of Sequences For A Flanking Position: 5
Maximum Number Of Contiguous Nonconserved Positions: 8
Minimum Length Of A Block: 10
Allowed Gap Positions: With Half
Use Similarity Matrices: Yes
```

```
Flank positions of the 7 selected block(s)
Flanks: [34  89]  [93  195]  [206  278]  [345  357]  [366  388]  [398  441]  [474  555]  

New number of positions in PGL1_unique_yersinia-CLUSTERS.dir/PGL1_unique_yersinia-CL3020/PGL1_unique_yersinia-CL3020.muscle.fasta.gblo:  394  (70% of the original 556 positions)
```
