# Supplementary material for: Genomic characterization of the Yersinia genus
Source: Genome Biol. 2010 Jan 4;11(1):R1. doi: 10.1186/gb-2010-11-1-r1 (PMC2847712; doi:10.1186/gb-2010-11-1-r1)
Supplement: Additional file 17 — The top level directory consists of a directory called Additional_cluster_files and 5010 directories, one for each multi-protein cluster family. (This top level directory has been split into three data files for uploading purposes (Additional files 15, 16, 17.) Within the directory are the following files: PGL1_unique_Yersinia_unclustered.out - list of all protein singletons that MCL did not group into a cluster (see Materials and Methods); PGL1_Yersinia_unique_locus_tags.txt - names of the 11 locus tag prefixes used for each genome; PGL1_unique_Yersinia.gff - mapping each Yersinia protein to a cluster in tab delimited GFF; PGL1_unique_Yersinia.sigfile - list of the longest protein in each cluster; PGL1_unique_Yersinia.summary - summary table of features of each of the clusters; PGL1_unique_Yersinia.table - summary table of each protein in the clusters. Within each cluster directory are the following files, where 'x' is the cluster name: PGL1_unique_Yersinia-x.faa - multifasta file of the proteins in the cluster; PGL1_unique_Yersinia-x.summary - summary of the properties of the proteins; PGL1_unique_Yersinia-x.matches - blast matches between the proteins of the cluster; PGL1_unique_Yersinia-x.muscle.fasta - muscle alignment of the proteins; PGL1_unique_Yersinia-x.muscle.fasta.gblo - gblocks output of muscle alignment (that is, auto-trimmed alignment); PGL1_unique_Yersinia-x.muscle.fasta.gblo.htm - as above in html format; PGL1_unique_Yersinia-x.muscle.tree - treefile from muscle alignment; PGL1_unique_Yersinia-x.sif - matches between proteins in simple interaction format for display on graphing software. [file gb-2010-11-1-r1-S17.zip › clusters3/PGL1_unique_yersinia-CL3021/PGL1_unique_yersinia-CL3021.muscle.fasta.gblo.htm]

PGL1\_unique\_yersinia-CL3021.muscle.fasta


## Gblocks 0.91b Results

Processed file: **PGL1\_unique\_yersinia-CL3021.muscle.fasta**  
Number of sequences: **6**  
Alignment assumed to be: **Protein**  
New number of positions: **112** (selected positions are underlined in blue)

```
                         10        20        30        40        50        60
                 =========+=========+=========+=========+=========+=========+
ypseu0001X_3701  MKTKKKAFSMKMRVFLLLISGVIAVFLFSRETILKKMGYQPATVSPSFVRGNKAVQFVEI
ypest0001X_9510  ---------------------MAAVFLFSRESVLKQMGYQPATVSQSFTRGNKAVQFVEI
ykris0001_5640   MNTQKKTFSMKVWGLLLLIA-LVTVVVIYHDVIWEKLNNKPIAISKSFIRGNKSVQFVEI
yrohd0001_6940   MNTKKKTLSMKVWGILLFIV-LAAIAAIYHKTILAKLKSEPVVVSKSFIRGNKAVQFVEI
yaldo0001_6810   ------------------------------------------------------------
yente0001X_6300  -----------------------------------------VAASKSFVRGDKAIQFVEL
                                                             ################


                         70        80        90       100       110       120
                 =========+=========+=========+=========+=========+=========+
ypseu0001X_3701  KNMVITLKDNNAERYLQLELGMATGDDKDIKNVIAMVPVIQATTVNLLSNMDYQAVRHTS
ypest0001X_9510  KNMVITLKDNNTERYLQLELGMATGDDKDIKNVIAMVPVIQATTVNLLSSMDYQAVRHTS
ykris0001_5640   KNMIITLKDIKTERYLQLELGMATGDDNDIKKVIAMVPVIQSATVNLLSHMDYQAVRNTS
yrohd0001_6940   KNMIITLKDNKSERYLQLELGVATGDDNDVKKVIAMVPVIQSATVNLLSNMDYQAVRSIS
yaldo0001_6810   --MIITLDDNKSERYLQLELGIATGDDDDVKRVIAMVPVIRAATVSLLSNRDYQTVRQIS
yente0001X_6300  KNMLITLDDNKSERYLQLE-----------------------------------------
                 ############################################################


                        130       140       150
                 =========+=========+=========+======
ypseu0001X_3701  ILDLRRQLMNEYKQDFAGLNVPMPFDDVVISRMVFQ
ypest0001X_9510  ILDLRRQLMNEYKQDFAGLNVPMPFDDVVISRMVFQ
ykris0001_5640   IVDLRRQLMNEYKKDFEKLNAPMPFDDVVISRMVFQ
yrohd0001_6940   IADLRRQLMSEYKIDFENLNSPMPFDDVVISRMVFQ
yaldo0001_6810   ITDLRRQLMDEYRKEFEYLNSEMPFDGVVISRMVFQ
yente0001X_6300  ------------------------------------
                 ####################################
```

```
Parameters used
Minimum Number Of Sequences For A Conserved Position: 4
Minimum Number Of Sequences For A Flanking Position: 5
Maximum Number Of Contiguous Nonconserved Positions: 8
Minimum Length Of A Block: 10
Allowed Gap Positions: With Half
Use Similarity Matrices: Yes
```

```
Flank positions of the 1 selected block(s)
Flanks: [45  156]  

New number of positions in PGL1_unique_yersinia-CLUSTERS.dir/PGL1_unique_yersinia-CL3021/PGL1_unique_yersinia-CL3021.muscle.fasta.gblo:  112  (71% of the original 156 positions)
```
