# Supplementary material for: Genomic characterization of the Yersinia genus
Source: Genome Biol. 2010 Jan 4;11(1):R1. doi: 10.1186/gb-2010-11-1-r1 (PMC2847712; doi:10.1186/gb-2010-11-1-r1)

Proteins conserved in pathogenic species but missing from non-pathogens.

| ***Y. enterocolitica* 8081** | **Length (aa)** | **Description** | ***Y. pestis* CO92** | ***Y. pseudotuberculosis* IP32953** | **Notes** |
| --- | --- | --- | --- | --- | --- |
| YE0393 | 106 | primosomal replication protein n | YPO3538 | YPTB0439 |  |
| YE0445 | 468 | probable outer membrane efflux lipoprotein (ileB) | YPO3481 | YPTB0493 |  |
| YE0759 | 66 | Hypothetical protein | YPO3367* | YPTB0763 | *In the same location as YPO3367 but ortholog in CO92 called on the opposite strand |
| YE2612 | 434 | putative salicylate synthetase | YPO1916 | YPTB1601 |  |
| YE2613 | 426 | putative signal transducer | YPO1915 | YPTB1600 |  |
| YE2614 | 600 | inner membrane ABC-transporter YbtQ | YPO1914 | YPTB1599 |  |
| YE2615 | 600 | lipoprotein inner membrane ABC-transporter | YPO1913 | YPTB1598 |  |
| YE2616 | 319 | transcriptional regulator YbtA | YPO1912 | YPTB1597 |  |
| YE2617 | 2035 | yersiniabactin biosynthetic protein | YPO1911 | YPTB1596 |  |
| YE2618 | 3161 | yersiniabactin biosynthetic protein | YPO1910 | YPTB1595 |  |
| YE2619 | 366 | yersiniabactin biosynthetic protein YbtU | YPO1909 | YPTB1594 |  |
| YE2620 | 267 | yersiniabactin biosynthetic protein YbtT | YPO1908 | YPTB1593 |  |
| YE2621 | 525 | yersiniabactin siderophore biosynthetic protein | YPO1907 | YPTB1592 |  |
| YE2622 | 673 | pesticin/yersiniabactin receptor protein | YPO1906 | YPTB1591 |  |
| YE3032 | 108 | Hypothetical protein | - | YPTB1208 | No locus assigned in Y.pestis CO92 but present in other Y. pestis |
| YE3036 | 215 | Putative uncharacterized protein | YPO2821 | YPTB1040 | Truncated in CO92 |
| YE3368 | 78 | Hypothetical protein | YPO0876 | YPTB3119 |  |
| YE3390 | 145 | Hypothetical protein | YPO0904 | YPTB3179 |  |
| YE3910 | 101 | 30S ribosomal protein S14 | YPO0222a | YPTB3685 |  |

Decline in number of unique genes as more genomes are added. Each value is the average of 8 random genome permutations.

       
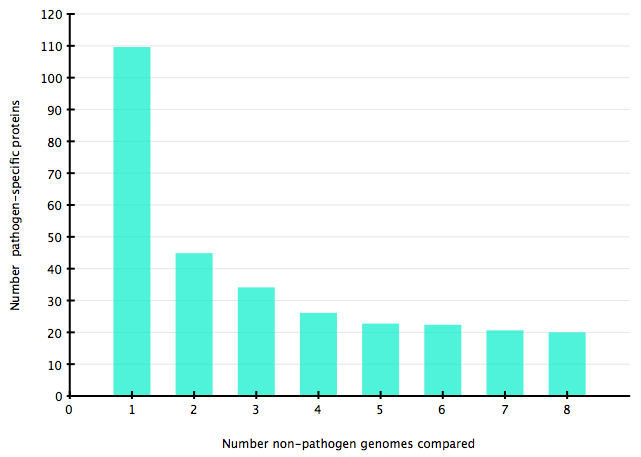

Supplement: Additional file 21 — A curve showing the rate of decline in number of this set as more non-pathogen genomes are added is also included. [file gb-2010-11-1-r1-S21.doc]
