# Supplementary material for: Genomic characterization of the Yersinia genus
Source: Genome Biol. 2010 Jan 4;11(1):R1. doi: 10.1186/gb-2010-11-1-r1 (PMC2847712; doi:10.1186/gb-2010-11-1-r1)
Supplement: Additional file 22 — Phylogeny of TTSS component YscN in Yersinia and other enterobacteria species. [file gb-2010-11-1-r1-S22.doc]

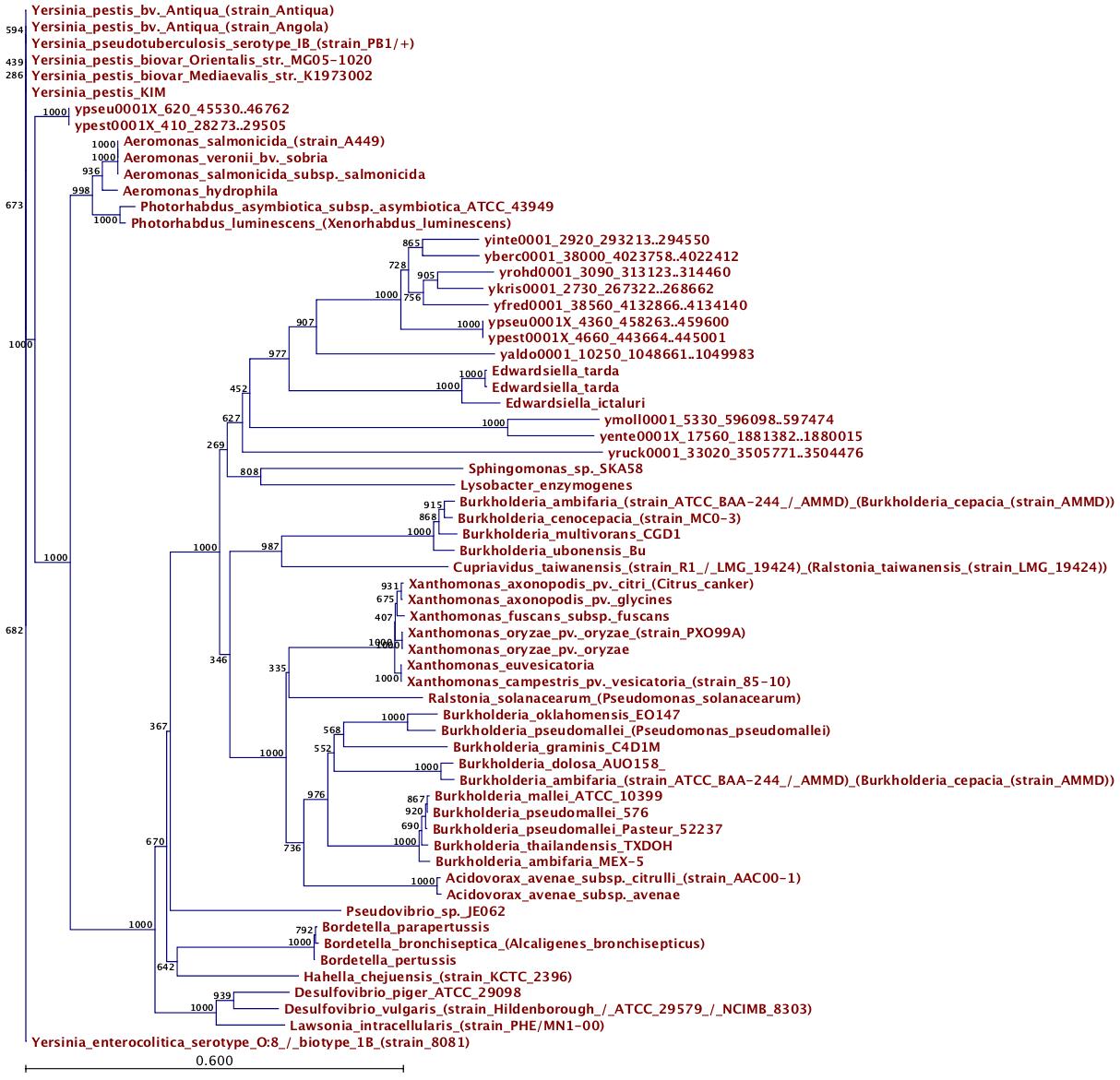


YscN orthologs from the 8 *Yersinia* genomes in this study were grouped with Y. pestis YscN UniRef50 group. All proteins within 25 amino acids length to the 439 YscN protein of Y. pestis were aligned using ClustalW and trimmed manually. A neighbor joining tree using PHYLIP was constructed. Plasmid and chromosomal clusters of Yersinia form separate branches and the Y. enterocolitica/ Y. mollareti branch are further from other enterocolitical like strains than the Y. pestis/ Y. pseudotuberculosis *ysa* ortholog.
