# Supplementary material for: Genomic characterization of the Yersinia genus
Source: Genome Biol. 2010 Jan 4;11(1):R1. doi: 10.1186/gb-2010-11-1-r1 (PMC2847712; doi:10.1186/gb-2010-11-1-r1)
Supplement: Additional file 24 — Calculations for the estimation of Π from aligned Yersinia core genomes. [file gb-2010-11-1-r1-S24.doc]

**Whole Genome-Based Nucleotide Diversity**

**# pairwise comparisons** 55

**# sites considered** 1,495,930

**# mismatches** 22,539,297

**π** 0.273946909280514

**5%** 0.0136973454640257

**# mismatches caused by 1 sequencing error** 10

**# mismatches needed to change pi by 5%** 1126965

**# sequencing errors needed** 112696

**bermuda model** 0.9999

**# sequencing errors assuming bermuda model** 150

**change in π** 1.82E-05

**% change in π 0.01%**

**genome size (# sites considered) needed to create 5% change in π** 1.13E+09
